# Tyrosine bioconjugation with hypervalent iodine†

**DOI:** 10.1039/d2sc04558c

**Published:** 2022-10-12

**Authors:** Nina Declas, John R. J. Maynard, Laure Menin, Natalia Gasilova, Sebastian Götze, Jakob L. Sprague, Pierre Stallforth, Stefan Matile, Jerome Waser

**Affiliations:** Laboratory of Catalysis and Organic Synthesis, Institut des Sciences et Ingénierie Chimique, Ecole Polytechnique Fédérale de Lausanne CH-1015 Lausanne Switzerland jerome.waser@epfl.ch; Department of Organic Chemistry, University of Geneva 1211 Geneva Switzerland stefan.matile@unige.ch; Institut des Sciences et Ingénierie Chimique, Ecole Polytechnique Fédérale de Lausanne, EPFL 1015 Lausanne Switzerland; Department of Paleobiotechnology, Leibniz Institute for Natural Product Research and Infection Biology, Hans Knöll Institute (HKI) 07745 Jena Germany; Department of Microbial Pathogenicity Mechanisms, Leibniz Institute for Natural Product Research and Infection Biology, Hans Knöll Institute (HKI) 07745 Jena Germany

## Abstract

Hypervalent iodine reagents have recently emerged as powerful tools for late-stage peptide and protein functionalization. Herein we report a tyrosine bioconjugation methodology for the introduction of hypervalent iodine onto biomolecules under physiological conditions. Tyrosine residues were engaged in a selective addition onto the alkynyl bond of ethynylbenziodoxolones (EBX), resulting in stable vinylbenziodoxolones (VBX) bioconjugates. The methodology was successfully applied to peptides and proteins and tolerated all other nucleophilic residues, with the exception of cysteine. The generated VBX were further functionalized by palladium-catalyzed cross-coupling and azide–alkyne cycloaddition reactions. The method could be successfully used to modify bioactive natural products and native streptavidin to enable thiol-mediated cellular uptake.

## Abstract 2

A tyrosine bioconjugation for the introduction of hypervalent iodine onto biomolecules is described. The transformation was applied to peptides and proteins and was used to modify native streptavidin to enable thiol-mediated cellular uptake.

## Graphical abstract

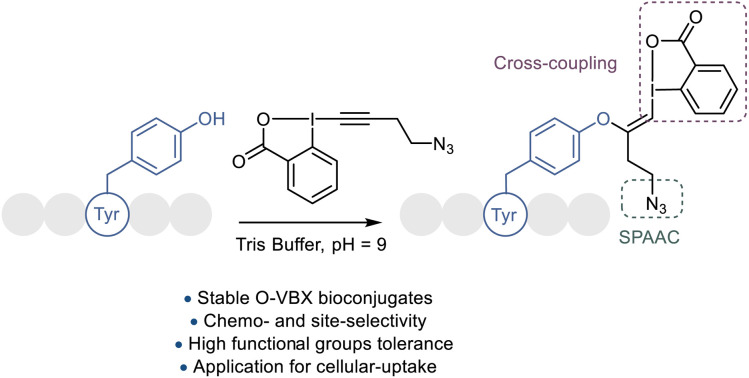

## Introduction

**Scheme 1:**
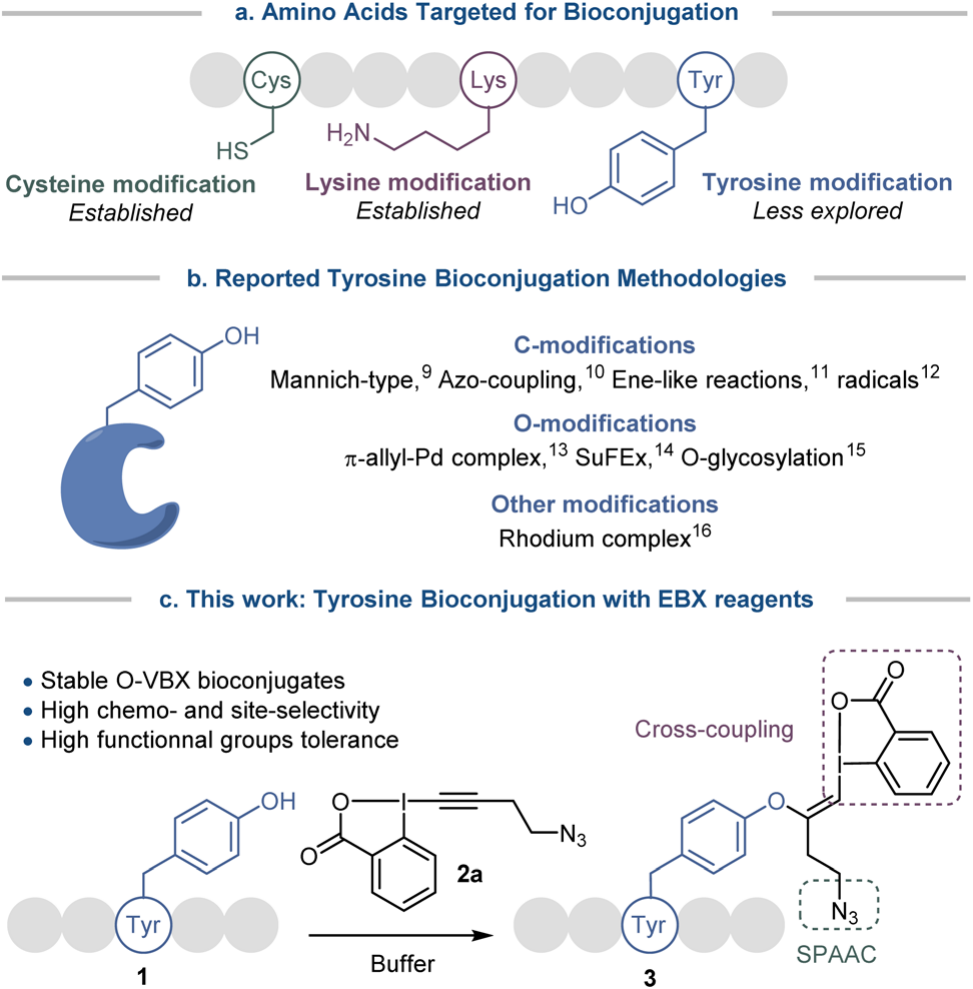
(a) Amino acids targeted for bioconjugation. (b) Reported tyrosine bioconjugation methodologies. (c) Development of a tyrosine-selective bioconjugation method with hypervalent iodine reagents.

Site-specific chemical modification of peptides and proteins is becoming increasingly important in research and industry for monitoring cellular events or designing therapeutics, targeting ligands and molecular probes.^1^ However the number of chemical transformations that are suitable for efficient biomolecule functionalization is limited by the stringent conditions required (mild and aqueous conditions, high selectivity, low toxicity) and developing chemo- and site-selective transformations remains a prominent challenge.^2^ Traditional methods for bioconjugation target nucleophilic lysine and cysteine residues (Scheme 1a).^2^ However, functionalization of the abundant lysine tends to lack selectivity. Modification of the rarer cysteine often requires cleavage of disulfide bonds, which play an important role in the folding and stability of proteins,^3^ or the expression of non-natural proteins with selectively introduced cysteines. To overcome these limitations and expand the functionalization toolbox, less exploited amino acids such as methionine,^4^ tryptophan,^5^ histidine^6^ and tyrosine^7^ have been investigated.

Tyrosine bioconjugation in particular shows promising results (Scheme 1b).^7^ Among natural amino acids, it is an interesting residue for site-selective modification, as it is relatively rare on protein surfaces,^8^ and is often buried within the protein structure due to the amphiphilicity of the phenol moiety. Several elegant methods have been reported for C-modification of tyrosine, such as three-component Mannich-type reactions,^9^ or reactions with aryldiazonium salts,^10^ diazodicarboxamides^11^ and radicals.^12^ O-functionalization has also been described, with for example reaction with electrophilic π-allyl palladium complexes,^13^ SuFEx reagents^14^ and glycosylation reactions.^15^ Functionalization has also been achieved *via* dearomatization−rearomatization strategies,^16^ or formation of a rhodium arene complex.^17^

All these strategies have advantages, but also inherent limitations, such as low site- or chemo-selectivity with competitive amino acids, side reactions, the use of expensive or toxic transition metals or the stability of the reagents and the bioconjugates. Therefore the development of alternative methods is still highly desirable. With their low toxicity, high functional group tolerance and stability in biocompatible media, hypervalent iodine reagents have recently emerged as powerful tools for late-stage peptide and protein functionalization.^18^ In 2019, our group reported the synthesis of vinylbenziodoxolone reagents (VBX) by stereoselective addition of phenols, sulfonamides and thiols nucleophiles onto ethynylbenziodoxolones (EBX).^19,20^ The VBX were shown to be bench-stable and the enhanced reactivity of the hypervalent bond allowed their use as electrophiles in palladium-catalyzed cross-couplings at room temperature. During this work,^19^ an N- and C-protected tyrosine could be used as a nucleophile, but the reaction conditions developed for small organic molecules were not compatible with peptides and proteins.

Moving from protected amino-acids to native peptides is challenging, as they contain multiple reactive nucleophilic functionalities and require aqueous buffers for solubilization, which are attenuating the reactivity of the residues and could degrade the reagents. Formation of S-VBX reagents from cysteine under physiological conditions was previously reported by our group.^20^ As a harder and less nucleophilic residue, tyrosine usually displays a lower reactivity with electrophiles than the soft and highly nucleophilic cysteine. Indeed, no tyrosine functionalization had been observed under the conditions developed for S-VBX formation. Nevertheless, free cysteine residues are occurring only rarely in peptides and proteins, most of them form disulfide bonds, which usually do not react with EBX reagents.^20^ It should be therefore possible to selectively target tyrosine if adequate conditions could be developed and other nucleophilic amino acids would not interfere. In this work, we report a tyrosine-selective modification of peptides with EBX 2a under mild and biocompatible conditions (Scheme 1c). With the exception of cysteine, all nucleophilic residues present in peptides were tolerated, and the method could be used for both synthetic peptides and natural products. Preliminary results indicate that the method can also be used with proteins with surface accessible tyrosine residues. The stable O-VBX conjugates were then used as a platform for further orthogonal modifications *via* a palladium-catalyzed Suzuki–Miyaura cross-coupling and a metal-free Strain-Promoted Alkyne–Azide Cycloaddition (SPAAC), providing a wide range of labelled biomolecules. Using this methodology, native streptavidin was modified to enable thiol-mediated cellular uptake.

## Results and discussion

### Optimization of the labelling on peptide tetramer 1a

We started our study with the functionalization of tetramer AFYA-NH_2_ (1a) as model substrate. AFYA-NH_2_ (1a) was easily obtained by solid-phase peptide synthesis (SPPS) and allowed a good detection by HPLC. Although 1a did not contain nucleophilic side chains, the presence of the free N-terminus was already expected to give an impression of the selectivity of the method towards other nucleophiles. We chose the azide-functionalized EBX JW-RF-010 (2a) as labeling reagent,^21^ as the azide group can be used for further functionalization *via* SPAAC.^20^ By simple dissolution of the peptide in non-degassed Tris buffer (10 mM, pH 8.2), and then addition of a solution of EBX 2a in DMSO, the O-VBX bioconjugate 3a could be obtained in 76% HPLC-yield after 24 hours of reaction at 37 °C (Table 1, entry 1). The reaction was clean and the only side products observed were N_3_-VBX 4 (ref. 22) and 2-iodobenzoic acid (5), both originating from EBX decomposition. No reaction on the potentially nucleophilic N-terminus was observed.

**Table 1:** Optimization of the labelling of tetramer 1a with JW-RF-010 (2a)^a^

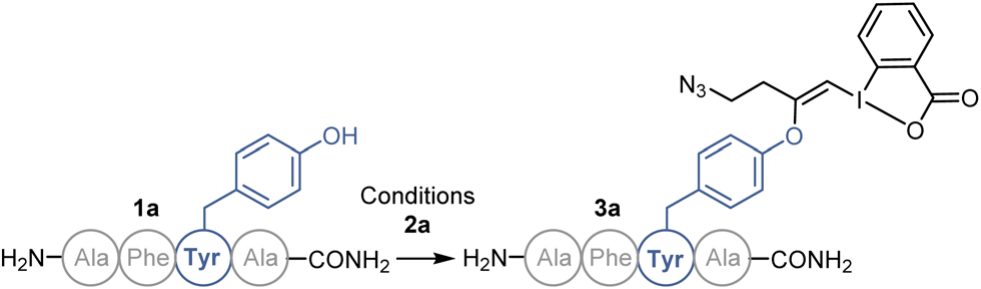				
Entry	Buffer (Molarity)	pH	*T* (°C)	Yield^b^
1	Tris (10 mM)	8.2	37	76%
2	Tris (1 mM)	8.2	37	52%
3	Tris (100 mM)	8.2	37	96%
4	Tris (1 M)	8.2	37	90%
5	PB (100 mM)	8.2	37	86%
6	HEPES (100 mM)	8.2	37	87%
7	10× PBS	8.2	37	86%
8	Tris (100 mM)	7.0	37	24%
**9**	**Tris (100 mM)**	**9.0**	**37**	**>99%**
10	Tris (100 mM)	9.0	rt	98%
11	Tris (100 mM)	9.0	50	70%
12	Tris (100 mM)	9.0	90	50%
13^c^	Tris (100 mM)	9.0	37	58%
14^d^	Tris (100 mM)	9.0	37	80%

a Conditions: AFYA-NH_2_1a (1.0 μmol), N_3_-EBX 2a (3.0 equiv), Buffer (2% v/v DMSO), 2 mM, 24 h.

b HPLC-MS yields are given. The yields were approximated as the ratio of Aprod/Atotal where Aprod = area in mAU of the product peak and Atotal = area in mAU of all peptides products (product, starting material, and side products if present).

c Reaction time: 1 h.

d Reaction time: 4 h.


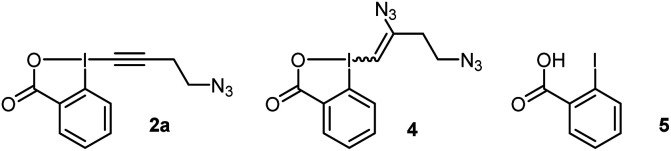
Decreasing the buffer molarity to 1 mM slowed down the bioconjugate formation, whereas increasing to 100 mM allowed to reach 96% HPLC-yield (entries 2–3). Under higher molarity, solubility issues of the peptide and higher EBX decomposition were observed (entry 4). The reaction displayed high tolerance towards buffer (entries 5–7), but additional side products were detected in PB, HEPES and PBS buffers.^23^ The pH was found to be an important factor for the efficiency of the labelling. At pH close to physiological pH (7.0), only 24% of O-VBX was formed (entry 8), whereas under more basic conditions (pH 9.0), quantitative peptide functionalization could be achieved (entry 9). Performing the reaction at room temperature slightly slowed down the labelling (entry 10). At higher temperature, the labelling remained efficient, but higher EBX decomposition was observed (entries 11–12). The developed methodology required 24 hours to reach full conversion of the peptide to the corresponding labelled product. However, decent conversion could be obtained in shorter time. After 1 hour, almost 60% conversion was achieved (entry 13), and up to 80% yield was obtained in 4 hours (entry 14).

### Scope of peptides

**Scheme 2:**
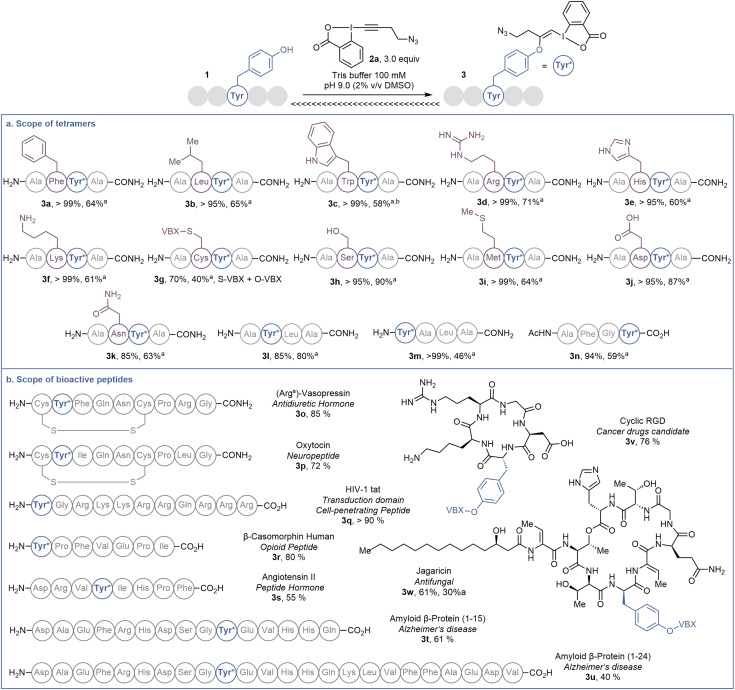
(a) Scope of tetramers on 20.0 μmol. (b) Scope of bioactive peptides on 1.0 μmol or 1.0 mg. Tyr* = Tyr modified with EBX. HPLC-MS yields are given, determined as indicated in Scheme 1. ^*a*^Isolated yield. ^*b*^Obtained as a 1 : 2 mixture with 4.

In order to investigate the functional group tolerance of the labelling method, tetrapeptides 1 containing various unprotected natural amino acids were then engaged in the reaction (Scheme 2a). Reproducibility of the reaction on larger scale (20 instead of 1 μmol) was first confirmed by full conversion of AFYA-NH_2_ (1a) to the corresponding O-VBX 3a, which was isolated in 64% yield following HPLC purification and characterized by ^1^H, ^13^C and 2-dimensional NMR spectroscopy.^24^ A sterically hindered leucine residue did not affect the reactivity and O-VBX bioconjugate 3b was obtained in 65% isolated yield. High chemoselectivity was achieved in the presence of tryptophan, however peptide 3c could only be isolated as a 1 : 2 mixture with N_3_-VBX 4. No side reactions occurred in the presence of the nucleophilic and basic arginine (3d), histidine (3e) and lysine (3f) residues.

When cysteine-containing tetramer 1g was submitted to the reaction conditions, fast addition of the cysteine residue was observed,^20^ followed by reaction of the tyrosine, leading to a doubly labelled S- and O-VBX product 3g in 40% isolated yield. Due to the inherent higher nucleophilicity of thiols, the functionalization of tyrosine in the presence of free cysteine has usually not been reported,^9,12*c*,15,25^ except for a few exceptions with selected biomolecules.^13^ Serine (3h), methionine (3i) and aspartic acid (3j) did not affect the reactivity, whereas a slightly lower yield due to incomplete conversion was observed for asparagine (3k). Finally, the position of tyrosine in the tetramer was varied (1l–1n). All peptides were successfully labelled to give products 3l–n, including N- and C-terminal tyrosine residues (3m and 3n).

The promising functional group tolerance and high chemoselectivity observed on tetramers drove us to apply the methodology to more complex peptides (Scheme 2b). The chemoselectivity of the peptides labelling was confirmed by MS/MS analysis (see ESI† for details). The neurohypophysial hormones vasopressin (1o) and oxytocin (1p), both containing a disulfide bond, were successfully functionalized at the tyrosine position (3o and 3p) with high conversion and no damage to the macrocyclic structure. High labelling conversion was also obtained at the N-terminal tyrosine of HIV-1-tat (1q) and β-casomorphin human (1r) peptides (VBXs 3q and 3r). Lower efficiency was observed on angiotensin II (1s), as well as on bigger amyloid β-proteins 1t and 1u, with however still high chemoselectivity towards the tyrosine residue (VBXs 3s, 3t and 3u). The cyclopentapeptide RGD (1v), containing an Arg–Gly–Asp motif and commonly used in targeted therapy for its property to bind specifically to integrin receptor on cell surface,^26^ was successfully labelled to give VBX 3v in 76% yield. Jagaricin (1w) is a cyclic lipopeptide natural product with promising antifungal properties.^27^ Despite the diversity of chemical structures present including threonine, histidine, glutamine and sensitive dehydrothreonine units, jagaricin (1w) was selectively labelled on the tyrosine residue with EBX 2a in 61% HPLC-MS yield and could be isolated in 30% yield (3wa).^28^

### Preliminary results on proteins

**Scheme 3:**
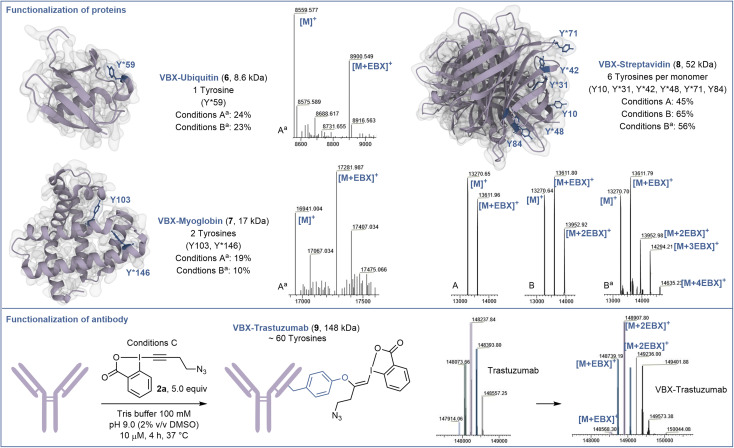
Functionalization of proteins and antibody. Conditions A: Protein (10.0 nmol), N_3_-EBX 2a (10.0 equiv), 100 μM in Tris buffer (100 mM, pH 9.0), 72 h. Conditions B: Protein (10.0 nmol), N_3_-EBX 2a (50.0 equiv), 100 μM in Tris buffer (100 mM, pH 9.0), 24 h. Conditions C: Trastuzumab (1.0 nmol), N_3_-EBX 2a (5.0 equiv), 10 μM in Tris buffer (100 mM, pH 9.0), 4 h. The ratio indicated corresponds to the signal intensity of all the functionalized products mass over the sum of the intensities of all other products with a threshold of 5% intensity. ^*a*^Denaturing conditions: Tris buffer (100 mM, pH 9.0, 6.0 M GdmHCl).

Next, the capacity of EBX 2a to label tyrosine on proteins was assessed (Scheme 3). When the small regulatory native protein Ubiquitin (8.6 kDa) was submitted to the reaction conditions, nearly no reaction was observed. This confirmed that only accessible tyrosine can be functionalized and no other residues react. However, with denaturing conditions (6.0 M GdmHCl) to gain access to the buried tyrosine together with a higher excess of EBX 2a (10 equiv) and a longer reaction time (72 hours) (conditions A^a^), modified ubiquitin 6 was obtained in 24% yield.

Selective modification of residue Y59 was confirmed by targeted top-down mass spectrometry analysis (see ESI† for details). Higher conversion could be obtained using 50 equiv of EBX 2a, but without increase in yield of 6 due to side reactions (conditions B^a^). The same conditions A^a^ were then applied to the hemeprotein myoglobin (17 kDa) containing 2 tyrosine residues. Chemo- and site-selectivity was achieved on the more reactive tyrosine Y146 to give 7 in 19% yield. Finally, we studied the functionalization of the tetrameric recombinant protein streptavidin (52 kDa), which contains 6 tyrosine residues per monomer (13 kDa), which are more accessible. Under native conditions (conditions A), 10 equiv of EBX 2a allowed to mono-label streptavidin on the most accessible tyrosine Y31, Y42, Y48 or Y71 to give 8. This result reflects the reactivity and accessibility of these amino acids compared to Y10 and Y84, which were not modified. Increasing the amount of EBX 2a to 50 equiv allowed to doubly modify the protein after 24 hours of incubation (conditions B). Again, only Y31, Y42, Y48 and Y71 were functionalized. Finally, under denaturing conditions, the 4 reactive tyrosine residues could be labelled (conditions B^a^). Interestingly, when another mutant streptavidin (66 kDa) was used, a better conversion was observed (see ESI†).

The incomplete conversion observed indicated that the tyrosines of the protein investigated so far were not fully accessible. We therefore decided to investigate the functionalization of trastuzumab (148 kDa), a monoclonal antibody used to treat breast cancer,^29^ containing a high number of tyrosine residues including some with high accessibility and reactivity.^30^ Gratefully, and despite the glycans present on the heavy chains, under mild conditions without denaturation, trastuzumab could be efficiently labelled with an average of 2 EBX and complete conversion after 4 hours to give 9. A longer reaction time and higher excess of EBX allowed to reach a higher degree of bioconjugation (see ESI† for details).

### Scope of EBX and further functionalization

**Scheme 4:**
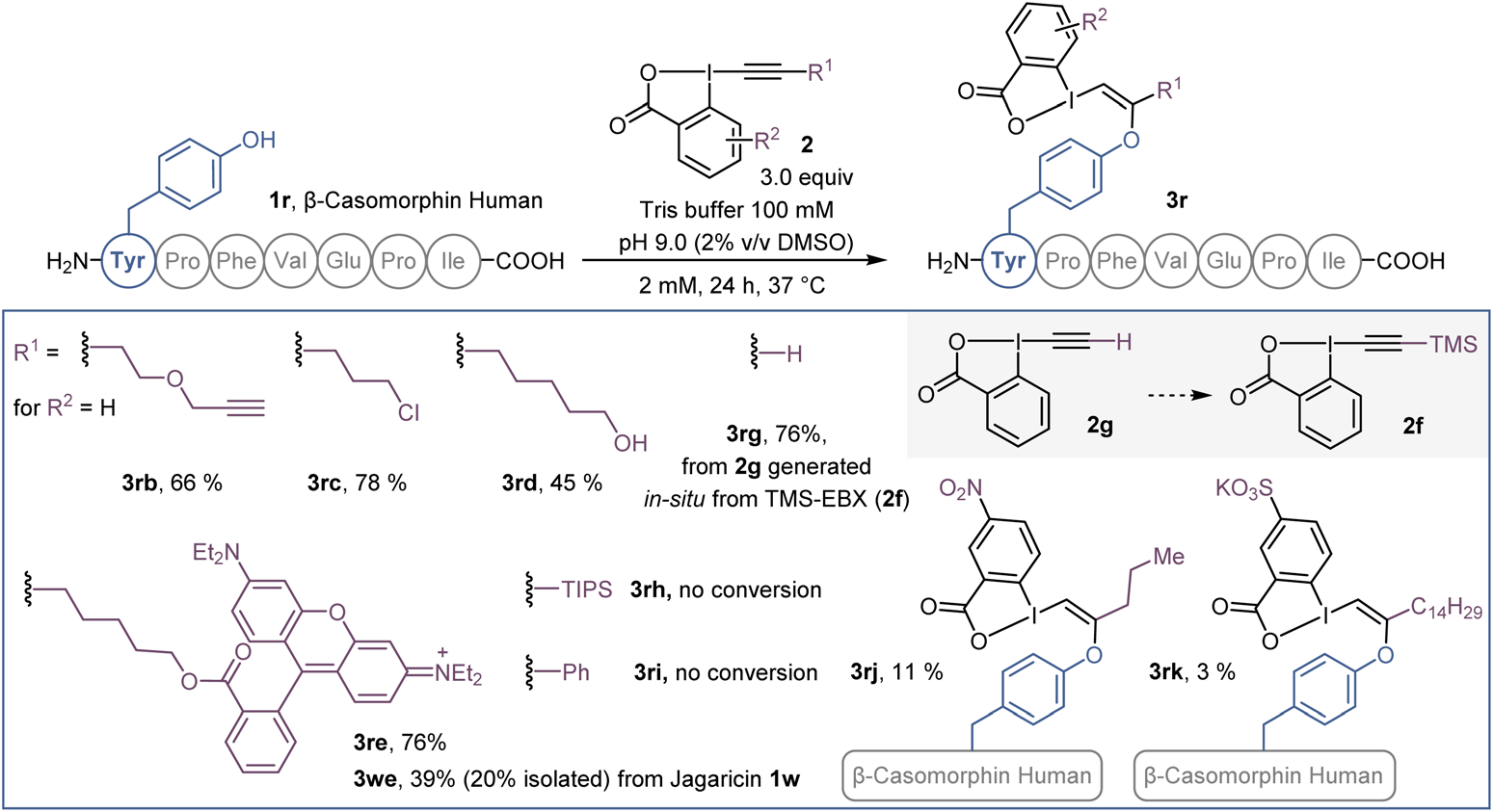
Scope of EBX reagents on β-casomorphin human peptide 1r and jagaricin (1w). HPLC-MS yields are given as indicated in Scheme 1.

Beside azide-bearing 2a, other hypervalent iodine reagents could be used as labelling reagents under the same reaction conditions (Scheme 4). EBX with alkyne (2b), chlorine (2c) and alcohol (2d) functional groups as well as a rhodamine fluorophore (2e), were successfully added to the N-terminal tyrosine of β-casomorphin human peptide (3r) to give VBXs 3rb–3re. In addition, jagaricin 3w, was labeled with a fluorescent rhodamine 2e and the isolated O-VBX (3we) could be used as a fluorescent stain in *Candida albicans* cells (see ESI† for microscopy images). Interestingly, TMS-EBX (2f) led to unsubstituted bioconjugate 3rg. Such a result was previously observed on cysteine,^31^ and is due to a fast and complete desilylation of TMS-EBX (2f) to give the free EBX (2g). However, unlike with cysteine, where the generated S-VBX underwent a slow rearrangement into the ethynylated product, the unsubstituted O-VBX 3rg remained stable over time. During screening of EBX reagents, some limitations were observed. TIPS- and Ph-EBX (2h and 2i) did not provide the expected labelled product 3rh and 3ri. No reaction occurred, which could be due to the low solubility of these reagents in aqueous media. Similarly, low conversion was obtained with *p*-NO_2_ substituted EBX 2j to give product 3rj. When the water soluble EBX 2k was used,^32^ no improvement was observed and less than 5% of bioconjugate 3rk was formed.^33^

**Scheme 5:**
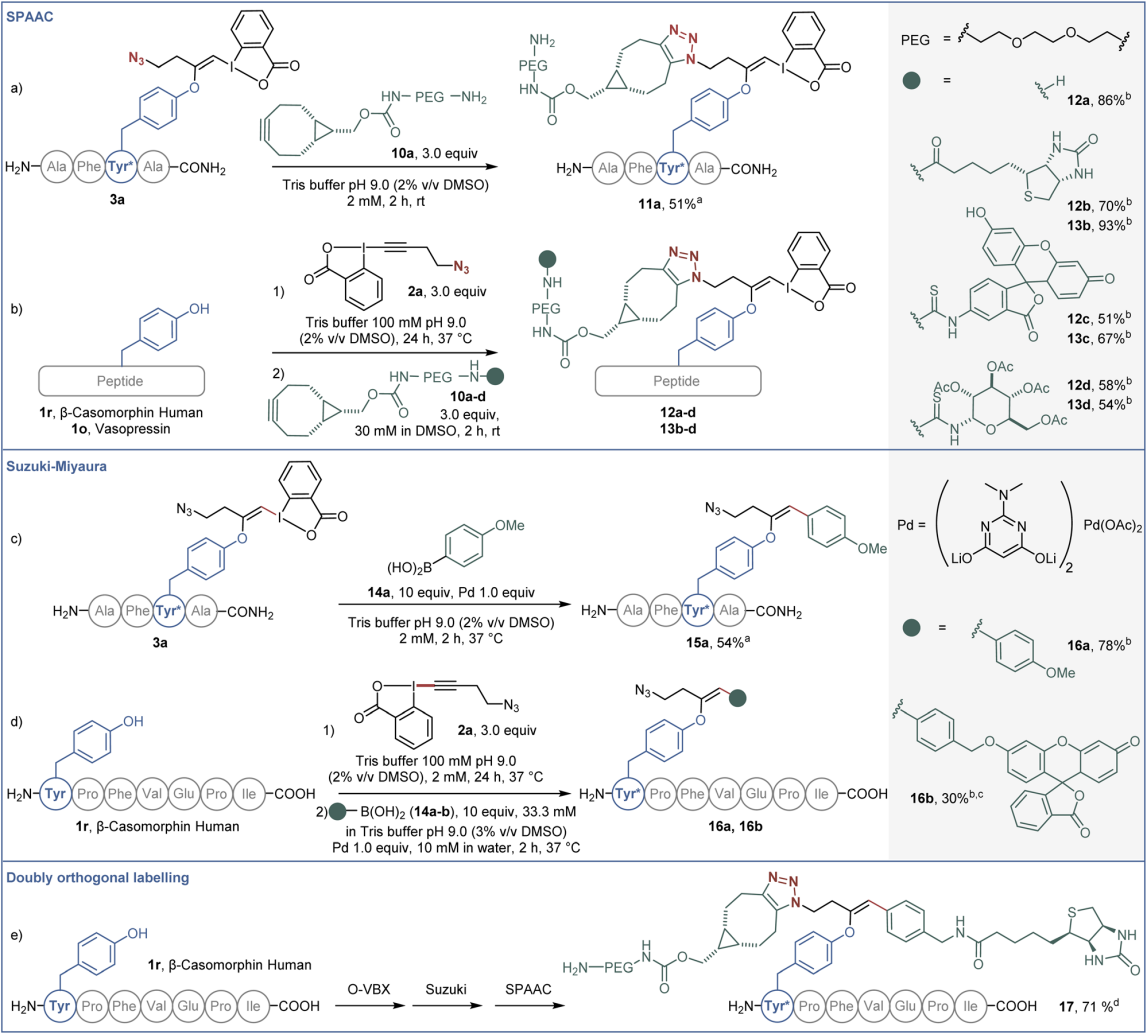
(a) SPAAC functionalization on O-VBX 3a, 20.0 μmol. (b) One-pot two-step labeling/SPAAC functionalization of peptides. Reaction on vasopressin (1o) (0.762 μmol) and β-casomorphin human peptide (1r) (1.0 μmol). (c) Suzuki–Miyaura functionalization on O-VBX 3a, 20.0 μmol. (d) One-pot two-step labeling/Suzuki–Miyaura functionalization of β-casomorphin human peptide (1.0 μmol). (e) One-pot three-step labeling/Suzuki–Miyaura/SPAAC functionalization of β-casomorphin human peptide (1.0 μmol). ^*a*^Isolated yield is given. ^*b*^HPLC-UV yield over two steps is given. ^*c*^10.0 equiv of Pd and 20% of DMSO were used. The reaction time was 4 hours. ^*d*^HPLC-UV yield over three steps is given.

The potential of the obtained bioconjugates for further functionalization was then investigated. We first optimized a copper-free SPAAC,^34^ taking advantage of the azide moiety on the labelled AFYA-NH_2_ tetramer 3a (Scheme 5a). In Tris buffer, an excess of PEG-functionalised bicyclononyne (BCN) 10a efficiently reacted with the azide functional group of the O-VBX 3a, leading to 11a in 51% isolated yield. With this result in hand, we then extended the click reaction to more complex peptides using a one-pot two-step procedure. β-Casomorphin human (1r) was successfully labelled with 10a to give 12a in 86% HPLC-yield. Both β-casomorphin human (1r) and vasopressin (1o) peptides were successfully labelled with N_3_-EBX (2a), followed by cycloaddition with BCN 10b–d functionalized with biotin, a fluorophore or a carbohydrate derivative (bioconjugates 12b–d and 13b–d).

In addition, the hypervalent iodine handle displayed high reactivity for cross-coupling reactions under mild conditions. Our group previously reported a Suzuki–Miyaura cross-coupling of the hypervalent iodine bond of S-VBX with boronic acids in aqueous media.^20^ Under similar conditions, labelled AFYA-NH_2_ tetramer 3a was successfully coupled with boronic acid 14a in 54% isolated yield (Scheme 5b). Coupling of *p*-methoxyphenyl boronic acid (14a) was also successful with β-casomorphin peptide (1r) using a one-pot two-step procedure. A fluorescein-substituted boronic acid 14b also reacted with the *in situ* generated O-VBX 3r, but a higher loading of palladium was required, as well as a higher ratio of DMSO to allow solubilization of the coupling partner.

Finally, both reactive handles were successfully employed in a labeling/Suzuki–Miyaura/SPAAC one-pot three-step process to afford doubly-functionalized β-casomorphin peptide 17. A biotin unit was installed using the palladium catalyzed reaction on the hypervalent iodine moiety, whereas the PEG unit was introduced by the click reaction.

### Application to the thiol-mediated cellular uptake of streptavidin

**Fig. 1:**
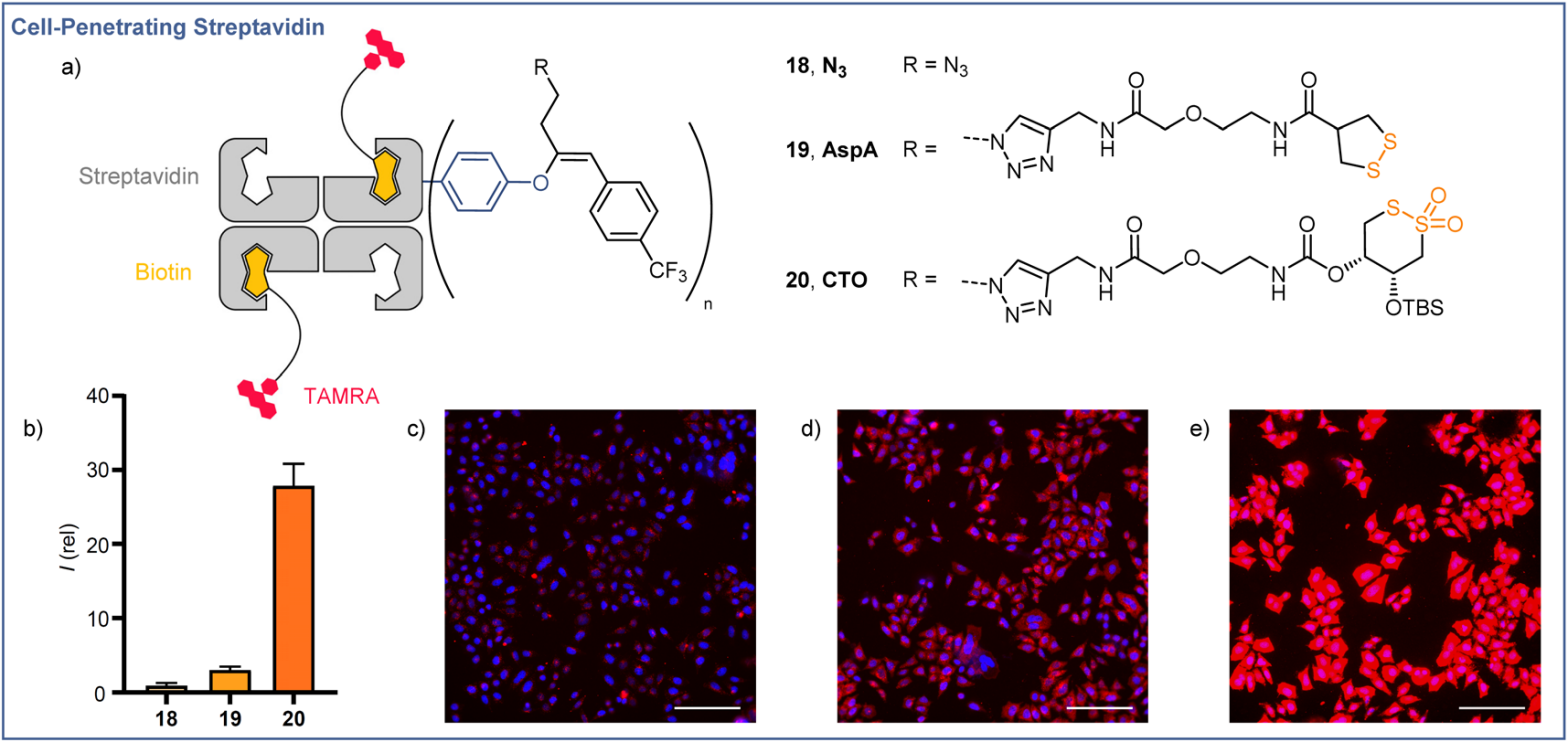
Cellular uptake of streptavidin using O-VBX. (a) Schematic representations of fluorescent biotin-streptavidin complexes used in uptake studies. (b) Relative fluorescence intensities of live HeLa MZ cells in SDCM images following incubation with 10 μM N_3_, AspA, or CTO for 6 h. Images of HeLa cells incubated with 18 N_3_ (c), 19 AspA (d), and 20 CTO (e) at 10 μM for 6 h (Blue = Hoechst 33342, Red = TAMRA). Scale bars: 150 μm. Brightness and contrast were adjusted equally in all images.

The cellular membrane provides a barrier to the passage of large, hydrophilic molecules, such as proteins, into the cytosol. Thiol-mediated uptake, postulated to function by transporter units engaging in dynamic covalent exchange cascades with cellular thiols and disulfides, has been employed previously for the efficient cytosolic delivery of streptavidin.^35–39^ Using the orthogonal reactivities offered by the EBX modification developed here, we were able to functionalize streptavidin (66 kDa) with both a transporter for thiol-mediated uptake, *via* CuAAC ‘click’ chemistry with the azide handle, and a lipophilic CF_3_ functionalized aromatic unit,^40^*via* Suzuki coupling with the hypervalent iodine motif. The functionalized proteins were still easily complexed with 2 equiv of a red fluorescent TAMRA-biotin derivative (Fig. 1a), and uptake of the complexes was studied in HeLa MZ cells. Compared with the protein after Suzuki coupling, but prior to click functionalization (18, R = N_3_), streptavidin functionalized with asparagusic acid (19, R = AspA) showed ∼3-fold increase in cellular uptake whereas streptavidin functionalized with a cyclic thiosulfonate (20, R = CTO), gave ∼28-fold increase in cellular uptake *versus* the N_3_ control (Fig. 1b). These results were in accordance with the relative reactivities of the two transporter motifs in thiol-disulfide exchange, and previous studies with small molecule fluorophore cargoes.^41^

Structure–activity relationships for cyclic thiosulfonate transporters and thiol-mediated uptake inhibitors revealed a strong dependence in either case on the presence of hydrophobic directing groups to target aprotic environments such as the cellular membrane and proteins, in which the proticity-dependent cascade exchange of cyclic thiosulfonates is enhanced.^41^ The ∼9-fold increase in uptake efficiency observed between AspA and CTO complexes exceeds the ∼3-fold difference seen with small molecule fluorophore cargoes, the greater differential perhaps resulting from the additional hydrophobic directing group provided by the CF_3_-functionalised aromatic unit, which is known to be more important for CTO transporters.^41^

In the case of AspA and CTO, a diffuse pattern of fluorescence was observed in spinning disk confocal microscopy (SDCM) images (Fig. 1d and e). In line with previous reports, this implied delivery to the cytosol rather than entrapment within endosomes following endocytosis, whereas N_3_ complex 18 showed a punctate fluorescence indicative of endocytosis (Fig. 1c).^42,43^ These results indicate the potential of readily accessible, broadly stable cyclic thiosulfonates as transporters for the efficient cytosolic delivery of proteins *via* thiol-mediated uptake, and highlight the possibilities offered by the dual, orthogonal functionalizations available following EBX-tyrosine modification.

## Conclusions

In summary, we have reported a chemo- and site-selective tyrosine bioconjugation methodology using hypervalent iodine reagents. Under mild and biocompatible conditions, tyrosine residues reacted with EBX reagents to generate stable O-VBX bioconjugates. The high functional group tolerance of the conjugation method allowed to selectively label a broad range of bioactive peptides, including the natural product jagaricin (3w). Preliminary results were obtained with selected proteins and an antibody. The generated O-VBX conjugates could be further functionalized orthogonally by palladium-catalyzed Suzuki–Miyaura cross-coupling and azide–alkyne cycloaddition, providing a wide range of labelled biomolecules. The potential of the doubly-orthogonal functionalization was demonstrated in a cellular uptake experiment, which further establishes that the functionalized streptavidin retains its structural integrity and activity to bind biotin, a crucial feature for applications in chemical biology. This work further highlights the potential of hypervalent iodine reagents in biomolecule labeling. Moreover, the high tyrosine selectivity described compares well with existing methods for O-functionalization of tyrosine residues, in which labelling of other nucleophilic amino acids such as lysine and histidine has often been reported.^14^

## Data availability

Experimental procedures, compound characterization and microscopy images are provided within the ESI† and raw data is available at zenodo.org: https://doi.org/10.5281/zenodo.7074420.

## Author contributions

N. D. developed and optimized the bioconjugation methodology, examined the scope of the reaction on peptides and proteins, investigated functionalization of the bioconjugates and prepared the related part of the manuscript and experimental parts. J. R. J. M. performed the cellular uptake experiments and prepared the related part of the manuscript and experimental parts. L. M. and N. G. performed the mass analysis and prepared the related experimental part. S. G. performed structural analysis studies on the jagaricin conjugates 3w and 3we and determined the biological activity of 3w. J. L. S. performed the microscopy experiments with 3we. P. S. supervised the experiments on the jagaricin conjugates and proofread the manuscript. S. M. supervised the cellular uptake experiments and proofread the manuscript. J. W. supervised the bioconjugation work, proofread the manuscript and experimental part and coordinated the research project.

## Conflicts of interest

There are no conflicts to declare.

## Supplementary Material

SC-013-D2SC04558C-s001
