# Correction: Tyrosine bioconjugation with hypervalent iodine

**DOI:** 10.1039/d2sc90252d

**Published:** 2022-12-15

**Authors:** Nina Declas, John R. J. Maynard, Laure Menin, Natalia Gasilova, Sebastian Götze, Jakob L. Sprague, Pierre Stallforth, Stefan Matile, Jerome Waser

**Affiliations:** a Laboratory of Catalysis and Organic Synthesis, Institut des Sciences et Ingénierie Chimique, Ecole Polytechnique Fédérale de Lausanne CH-1015 Lausanne Switzerland jerome.waser@epfl.ch; b Department of Organic Chemistry, University of Geneva 1211 Geneva Switzerland stefan.matile@unige.ch; c Institut des Sciences et Ingénierie Chimique, Ecole Polytechnique Fédérale de Lausanne, EPFL 1015 Lausanne Switzerland; d Department of Paleobiotechnology, Leibniz Institute for Natural Product Research and Infection Biology, Hans Knöll Institute (HKI) 07745 Jena Germany; e Department of Microbial Pathogenicity Mechanisms, Leibniz Institute for Natural Product Research and Infection Biology, Hans Knöll Institute (HKI) 07745 Jena Germany

## Abstract

Correction for ‘Tyrosine bioconjugation with hypervalent iodine’ by Nina Declas *et al.*, *Chem. Sci.*, 2022, **13**, 12808–12817, https://doi.org/10.1039/D2SC04558C.

The authors regret that due to a technical issue with the ChemDraw file, part of the reaction conditions in Scheme 2 on page 12811 of the original article were not visible. A revised Scheme 2 with full reaction conditions is shown below:

**Scheme 2:**
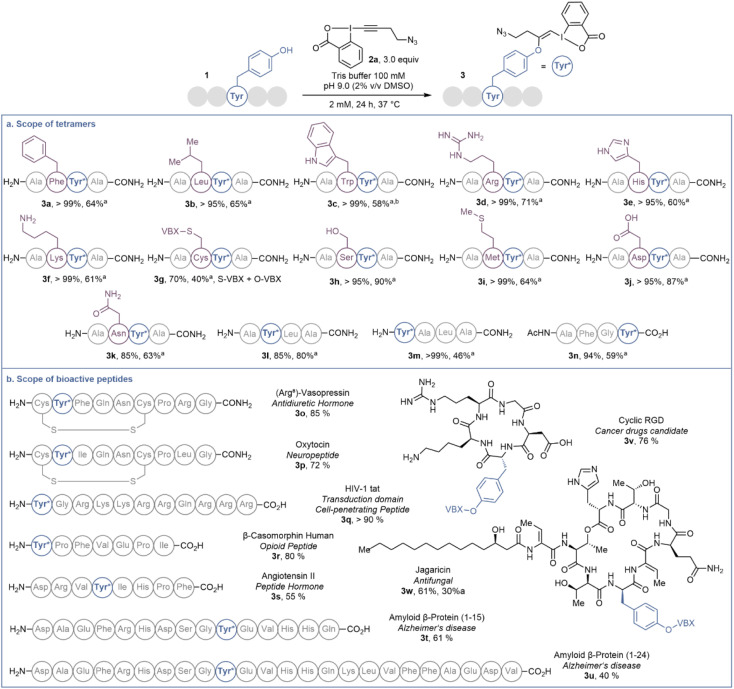
(a) Scope of tetramers on 20.0 μmol. (b) Scope of bioactive peptides on 1.0 μmol or 1.0 mg. Tyr* = Tyr modified with EBX. HPLC-MS yields are given, determined as indicated in Scheme 1. ^*a*^Isolated yield. ^*b*^Obtained as a 1 : 2 mixture with 4.

The Royal Society of Chemistry apologises for these errors and any consequent inconvenience to authors and readers.

## Supplementary Material

