# Supplementary material for: Tyrosine bioconjugation with hypervalent iodine
Source: Chem Sci. 2022 Oct 12;13(43):12808–17. doi: 10.1039/d2sc04558c (PMC9645396; doi:10.1039/d2sc04558c)

Electronic Supplementary Material (ESI) for Chemical Science.
This journal is © The Royal Society of Chemistry 2022

Tyrosine Bioconjugation with Hypervalent Iodine

Nina Declas, John R. J. Maynard, Laure Menin, Natalia Gasilova, Sebastian Götze, Jakob L. Sprague, Pierre Stallforth, Stefan Matile and Jerome Waser

Supporting Information (202 pages)

Table of Contents

1. General procedures 3
2. HPLC-MS and preparative HPLC information 4
   a. HPLC-MS analysis 4
   b. Preparative HPLC 4
3. Synthesis of hypervalent iodine reagents (EBX) 5
   a. Synthesis of alkyne substrates 5
   b. Synthesis of ethynylbenziodoxolone reagents 8
4. Synthesis of tetramers 17
5. Scope of tetramers 26
6. Scope of peptides 55
7. Protein bioconjugation 73
8. Scope of EBX on β-Casomorphin 91
9. Click chemistry 112
   c. Synthesis of Click reagents 112
   d. SPAAC on isolated O-VBX 117
   e. One pot-two steps SPAAC: 119
10. Suzuki reaction 134
   a. Synthesis of ligands and preparation of palladium complexes 134
   b. Synthesis of boronic acid 135
   c. Suzuki on isolated O-VBX: 136
   d. One pot-two steps Suzuki: 138
11. Labelling/SPAAC/Suzuki 143
12. Jagaricin fluorescent microscopy experiment 146
13. Cellular uptake experiments 147
14. Stability evaluation of O-VBX 157
15. Full mass spectrum for proteins 158
16. NMR spectrum of the synthesized compounds 165

1. General procedures

All reactions using anhydrous conditions were performed with oven-dried glassware, under an atmosphere of nitrogen, unless stated otherwise. Tetrahydrofuran, acetonitrile, diethyl ether and dichloromethane (DCM) were dried by passage over activated alumina, under nitrogen atmosphere, on an Innovative Technology Solvent Delivery System (water content < 10 ppm, Karl-Fischer titration). Dichloroethane and ethanol were purchased from Acros and trifluoroethanol was purchased from Fluorochem. DMSO was purchased from Sigma-Aldrich. All the Fmoc-protected amino acids and Rink Amide MBHA resin were purchased from GL Biochem or Bachem. 1-[Bis(dimethylamino)methylene]-1H-1,2,3-triazolo[4,5-b]pyridinium 3-oxide hexafluorophosphate (HATU, Bachem) and N,N-diisopropylethylamine (DIPEA, Iris Biotech GmbH) were used as received. Vasopressin, oxytocin, HIV-1 tat, angiotensin, β-casomorphin, amyloid β-protein and cyclic RGD were purchased from Bachem in lyophilized form. Ubiquitin was purchased from Bio-Techne and Myoglobin from Sigma Aldrich in lyophilized form. All the other reagents were purchased from ABCR, Acros, AlfaAesar, Apollo Scientific, Fluorochem, Fluka, Roth, Sigma-Aldrich and TCI and were used as such. For flash chromatography, distilled technical grade solvents were used. Chromatographic purification was performed as flash chromatography using Macherey-Nagel silica 40-63, 60 Å, using the solvents indicated as eluent with 0.1 – 0.5 bar pressure. TLC was performed on Merck silica gel 60 F254 TLC aluminum or glass plates and visualized with UV light or permanganate stain. Melting points were measured on a Büchi B-540 melting point apparatus using open glass capillaries. ^{1}H-NMR spectra were recorded on a Bruker DPX-400 400 MHz spectrometer in $CDCl_3$, DMSO-d_6, CD_3OD, or D_2O. All signals are reported in ppm with the internal $CHCl_3$ signal at 7.26 ppm, the internal DMSO signal at 2.50 ppm and CD_3OD as 3.31 ppm as standard. The data is being reported as: s = singlet, d = doublet, t = triplet, q = quadruplet, qi = quintet, m = multiplet or unresolved, br = broad signal, app = apparent, coupling constant(s) in Hz, integration, interpretation.^{13}C-NMR spectra were recorded with 1H-decoupling on a Brucker DPX-400 100 MHz spectrometer in $CDCl_3$, DMSO-d_6 or CD_3OD. All signals are reported in ppm with the internal $CHCl_3$ signal at 77.16 ppm or the internal DMSO signal at 39.52 ppm as standard. Spectra were fully assigned using COSY, HSQC, HMBC and ROESY. Infrared spectra were recorded on a JASCO FT-IR B4100 spectrophotometer with an ATR PRO410-S and a ZnSe prisma and are reported as cm^{-1} (w = weak, m = medium, s = strong, br = broad). High-resolution mass spectrometry measurements were performed by the mass spectrometry service of ISIC at the EPFL on LTQ Orbitrap ELITE ETD and Exploris 240 instruments (Thermofisher) and Xevo G2-S QTOF (Waters).

2. HPLC-MS and preparative HPLC information

a. HPLC-MS analysis

HPLC-MS measurements were performed on an Agilent 1290 Infinity HPLC system with a G4226a 1290 Autosampler, a G4220A 1290 Bin Pump and a G4212A 1290 DAD detector, connected to a 6130 Quadrupole LC/MS, coupled with a Waters XBridge C18 column (250 x 4.6 mm, 5 µm). Water:acetonitrile 95:5 (solvent A) and water:acetonitrile 5:95 (solvent B), each containing 0.1% formic acid, were used as the mobile phase, at a flow rate of 0.6 $mL.min^{-1}$. The gradient was programmed as follows:

Method 1: 100% A for 5 minutes and then a gradient to 100% B in 20 minutes, plus 5 minutes of 100% B.

Method 1 was used for HPLC-MS analysis unless noted otherwise.

The column temperature was set up to 25 °C. Low-resolution mass spectrometric measurements were acquired using the following parameters: positive electrospray ionization (ESI), temperature of drying gas = 350 °C, flow rate of drying gas = 12 L. min^{-1}, pressure of nebulizer gas = 60 psi, capillary voltage = 2500 V and fragmentor voltage = 70 V.

b. Preparative HPLC

Preparative RP-HPLC were performed on an Agilent 1260 HPLC system with a G2260A 1260 Prep ALS Autosampler, a G1361a 1260 Prep Pump, a G1365C 1260 MWD detector and a G1364B 1260 FC-PS collector, coupled with a Waters XBridge semi-preparative C18 column (19 x 150 mm, 5 µm). Water (solvent A) and water:acetonitrile 5:95 (solvent B), each containing 0.1% TFA, were used as the mobile phase at a flow rate of 20 $mL.min^{-1}$. The gradient was programmed as follows: 100% A isocratic for 5 minutes followed by 100% A to 100% B in 20 minutes then isocratic for 5 minutes.

3. Synthesis of hypervalent iodine reagents (EBX)

a. Synthesis of alkyne substrates

(4-Iodo-but-1-yn-1-yl)trimethylsilane (**S2**)

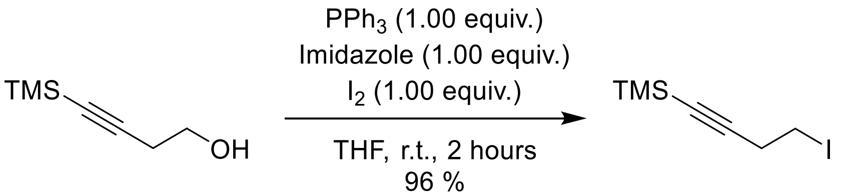

S1 **S2**

Following a slightly modified procedure,[1] triphenylphosphine (PPh_3, 37.9 g, 145 mmol, 1.00 equiv.) was added to a cooled solution of 4-(trimethylsilyl)but-3-yn-1-ol (**S1**) (20.6 g, 145 mmol, 1.00 equiv.) in tetrahydrofuran (545 mL) at 0 °C. Upon dissolution, imidazole (9.84 g, 145 mmol, 1.00 equiv.) was added, followed by iodine (I_2, 36.7 g, 145 mmol, 1.00 equiv.). The resulting mixture was then allowed to warm to room temperature and was stirred for 2 hours. It was then diluted with diethyl ether (400 mL) and washed with 10% aqueous sodium thiosulfate (400 mL). The aqueous layer was extracted with additional portions of diethyl ether (2 x 150 mL) and the combined organic layers were washed with brine (400 mL), dried over magnesium sulfate, filtered and concentrated *in vacuo*. The resulting white suspension was filtered through a plug of silica, eluting with pentane, to afford pure (4-iodo-but-1-yn-1-yl)trimethylsilane (**S2**) (34.9 g, 138 mmol, 96% yield) as a colorless oil.

^{1}H NMR (400 MHz, $CDCl_3$) δ 3.20 (t, *J* = 7.5 Hz, 2H, CH_2CH_2I), 2.78 (t, *J* = 7.5 Hz, 2H, CH_2CH_2I), 0.14 (s, 9H, TMS). **^{13}C NMR** (101 MHz, $CDCl_3$) δ 105.1, 86.9, 25.3, 1.1, 0.1.

Spectroscopic data was consistent with the values reported in literature.[2]

(4-Azidobut-1-yn-1-yl)trimethylsilane (S3)

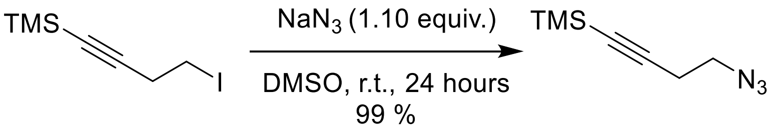

S2 **S3**

Following a slightly modified procedure,[3] (4-iodobut-1-yn-1-yl)trimethylsilane (**S2**) (34.9 g, 138 mmol, 1.00 equiv.) was added to a 0.5 M solution of sodium azide in dimethyl sulfoxide (NaN_3, 304 mL, 152 mmol, 1.10 equiv.). The reaction mixture was stirred for 24 hours at room temperature, then slowly poured into a mixture of ice:water (800 mL). The aqueous layer was extracted with diethyl ether (3 x 300 mL) and the combined organic layers were washed with water (2 x 200 mL), brine (200 mL), dried over magnesium sulfate, filtered and concentrated under reduced pressure. The light yellow crude liquid was purified through a plug of silica,

---

[1] Rodier, F.; Rajzmann, M.; Parrain, J. L.; Chouraqui, G.; Commeiras, L. *Chem. Eur. J.* **2013**, *19*, 2467.
[2] Berkessel, A.; Kramer, J.; Mummy, F.; Neudorfl, J. M.; Haag, R. *Angew. Chem. Int. Ed.* **2013**, *52*, 739.
[3] Frei, R.; Wodrich, M. D.; Hari, D. P.; Borin, P.-A.; Chauvier, C.; Waser J. *J. Am. Chem. Soc.* **2014**, *136*, 16563.

eluting with pentane, to afford pure (4-azidobut-1-yn-1-yl)trimethylsilane (**S3**) (22.8 g, 136 mmol, 99% yield) as a colorless liquid.

^{1}H NMR (400 MHz, $CDCl_3$) δ 3.37 (t, *J* = 6.8 Hz, 2H, $CH_2CH_2N_3$), 2.52 (t, *J* = 6.8 Hz, 2H, $CH_2CH_2N_3$), 0.15 (s, 9H, TMS). **^{13}C NMR** (101 MHz, $CDCl_3$) δ 102.8, 87.3, 49.8, 21.1, 0.0.

Spectroscopic data was consistent with the values reported in literature.[4]

Trimethyl(4-(prop-2-yn-1-yloxy)but-1-yn-1-yl)silane (S4)

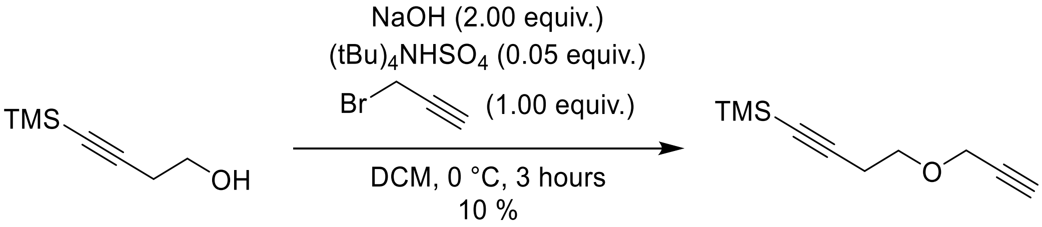

S1 **S4**

Following a reported procedure,[4] 4-(trimethylsilyl)but-3-yn-1-ol (**S1**) (4.00 g, 28.1 mmol, 1.00 equiv.) was dissolved in dichloromethane (60 mL) and the solution was cooled down at 0 °C. Then, tetrabutylammonium hydrogensulfate ((*t*Bu)$_4$NHSO$_4$, 0.477 g, 1.41 mmol, 0.05 equiv.) was added, followed by sodium hydroxide (NaOH, 2.25 g, 56.2 mmol, 2.00 equiv.). The reaction mixture was stirred at this temperature for 5 minutes and then propargyl bromide (3.03 mL, 28.1 mmol, 1.00 equiv.) was added. The resulting yellow reaction mixture was stirred for 4 hours at 0 °C and quenched with water (60 mL) keeping the internal temperature at 0 °C. The aqueous layer was extracted with dichloromethane (60 mL), then the combined organic layers were dried over magnesium sulfate, filtered and concentrated *in vacuo*. The crude yellow oil was purified by column chromatography (SiO_2, Pentane:Ethyl acetate 99:1) affording pure trimethyl(4-(prop-2-yn-1-yloxy)but-1-yn-1-yl)silane (**S4**) (1.44 g, 8.00 mmol, 29% yield) as a colorless liquid.

R_f 0.22 (Pentane:Ethyl acetate 99:1). **^{1}H NMR** (400 MHz, $CDCl_3$) δ 4.19 (dd, *J* = 6.9, 2.4 Hz, 2H, $CCCH_2O$), 3.66 (q, *J* = 7.1 Hz, 2H, OCH_2), 2.53 (t, *J* = 7.2, 2H, OCH_2CH_2), 2.44 (dt, *J* = 3.2, 2.4 Hz, 1H, CC*H*), 0.14 (s, 9H, TMS). **^{13}C NMR** (101 MHz, $CDCl_3$) δ 103.3, 86.1, 79.6, 74.7, 68.3, 58.3, 21.3, 0.2.

Spectroscopic data was consistent with the values reported in literature.[4]

7-(Trimethylsilyl)hept-6-yn-1-ol (S6)

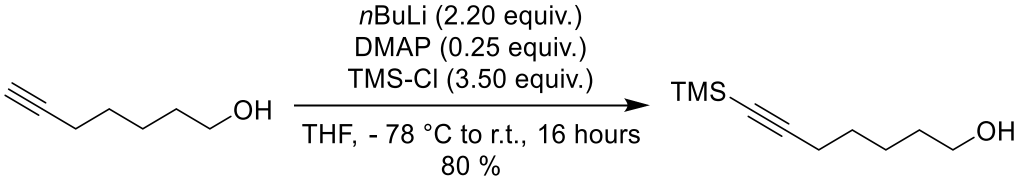

S5 **S6**

[4] Diaz, L.; Bujons, J.; Casas, J.; Llebaria, A.; Delgado, A. *J. Med. Chem.* **2010**, *53*, 5248.

Following a reported procedure,[5] hept-6-yn-1-ol (**S5**) (5.00 g, 44.6 mmol, 1.00 equiv.) was dissolved in tetrahydrofuran (150 mL) and the solution was cooled down at - 78 °C. A cooled 2.5 M solution of *n*-butyllithium in hexanes (*n*BuLi, 39.2 mL, 98.0 mmol, 2.20 equiv.) was added dropwise, followed by 4-(dimethylamino)pyridine (DMAP, 1.36 g, 11.1 mmol, 0.25 equiv.). After stirring for 1 hour at this temperature, trimethylsilyl chloride (TMS-Cl, 20.4 mL, 156 mmol, 3.50 equiv.) was added dropwise. The mixture was then allowed to warm to room temperature. After 2 hours of stirring, the reaction was quenched with a 1.0 N aqueous hydrochloric acid (50 mL) and vigorously stirred at room temperature over 30 minutes. The mixture was then diluted with ethyl acetate (200 mL) and the layers were separated. The aqueous phase was extracted with additional portions of ethyl acetate (3 x 50 mL). The combined organic layers were collected, washed with a solution of saturated aqueous sodium bicarbonate (100 mL), brine (50 mL), dried over magnesium sulfate, filtered and concentrated *in vacuo*. After purification by column chromatography (SiO_2, Pentane:Ethyl acetate 4:1), 7-(trimethylsilyl)hept-6-yn-1-ol (**S6**) (6.58 g, 35.7 mmol, 80% yield) was obtained as a colorless oil.

R_f 0.32 (Pentane:Ethyl acetate 4:1). **^{1}H NMR** (400 MHz, $CDCl_3$) δ 3.65 (td, *J* = 6.5, 1.0 Hz, 2H, C*H_2*OH), 2.24 (td, *J* = 7.0, 1.0 Hz, 2H, CCC*H_2*), 1.63-1.51 (m, 4H, C*H_2*), 1.51-1.41 (m, 2H, C*H_2*), 0.14 (s, 9H, TMS). **^{13}C NMR** (101 MHz, $CDCl_3$) δ 107.4, 84.6, 62.9, 32.3, 28.6, 25.1, 19.9, 0.3.

Spectroscopic data was consistent with the values reported in literature.[6]

Trimethylsilyl(triisopropylsilyl)acetylene (S8)

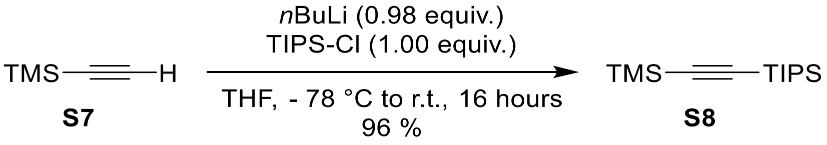

Following a reported procedure,[7] a cooled 2.5 M solution of *n*-butyllithium in hexanes (*n*BuLi, 86.0 mL, 209 mmol, 0.98 equiv.) was added dropwise to a stirred solution of trimethylsilylacetylene (**S7**) (30.3 mL, 213 mmol, 1.00 equiv.) in tetrahydrofuran (330 mL) at - 78 °C. After stirring for 2 hours at this temperature, tri*iso*propyl chloride (TIPS-Cl, 45.6 mL, 213 mmol, 1.00 equiv.) was added dropwise. The mixture was then allowed to warm to room temperature. After stirring overnight, the reaction was treated with a saturated aqueous solution of ammonium chloride (300 mL) and was extracted with diethyl ether (2 x 300 mL). The combined organic phase were dried over magnesium sulfate, filtered and concentrated under reduced pressure. The crude product was passed through a short plug of silica, eluting with pentane to afford trimethylsilyl(tri*iso*propylsilyl)acetylene (52.5 g, 206 mmol, 96% yield) as a colorless liquid (**S8**).

^{1}H NMR (400 MHz, $CDCl_3$) δ 1.08 (m, 21H, TIPS), 0.18 (s, 9H, TMS). **^{13}C NMR** (101 MHz, $CDCl_3$) δ 116.3, 110.3, 18.7, 11.2, 0.2.

Spectroscopic data was consistent with the values reported in literature.[8]

---

[5] Peixoto, P. A.; Richard, J. A.; Severin, R.; Chen, D. Y. *Org. Lett.* **2011**, *13*, 5724.
[6] Rodier, F.; Rajzmann, M.; Parrain, J. L.; Chouraqui, G.; Commeiras, L. *Chem. Eur. J.* **2013**, *19*, 2467.
[7] Helal, C J.; Magriotis, P. A.; Corey, E. J. *J. Am. Chem. Soc.* **1996**, *118*, 10938.
[8] Brand, J. P.; Waser, J. *Synthesis* **2012**, *44*, 1155.

b. Synthesis of ethynylbenziodoxolone reagents

(4-Azidobut-1-ynyl)-1,2-benziodoxol-3(1H)-one (2a)

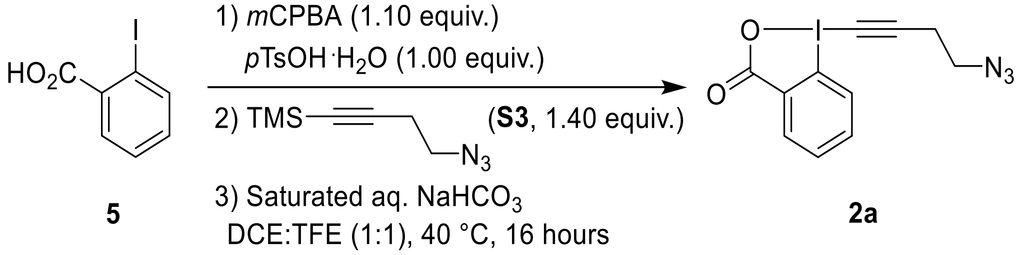

Following a reported procedure,[9] 2-iodobenzoic acid (**5**) (24.1 g, 97.0 mmol, 1.00 equiv.), *para*-toluene sulfonic acid monohydrate (*p*TsOH·H_2O, 18.5 g, 97.0 mmol, 1.00 equiv.) and *meta*-chloroperoxybenzoic acid (*m*CPBA-77%, 23.9 g, 107 mmol, 1.10 equiv.) were dissolved in a mixture of dichloroethane (81 mL) and 2,2,2-trifluoroethanol (81 mL). After 1 hour stirring at 40 °C, (4-azidobut-1-yn-1-yl)trimethylsilane (**S3**) (22.7 g, 136 mmol, 1.40 equiv.) was added in one portion. The reaction mixture was stirred for an additional 14 hours at the same temperature, then the resulting suspension was filtered and the volatiles were removed under reduced pressure. The resultant residue was dissolved in dichloromethane (1000 mL) and treated with a solution of saturated aqueous sodium bicarbonate (1000 mL). The mixture was vigorously stirred for 1 hour, then the two layers were separated and the aqueous layer was extracted with additional portions of dichloromethane (3 x 500 mL). The organic layers were combined, dried over magnesium sulfate; filtered and concentrated under reduced pressure. Purification by column chromatography (SiO_2, Ethyl acetate) afforded (4-azidobut-1-ynyl)-1,2-benziodoxol-3(1H)-one (**2a**) (5.23 g, 15.3 mmol, 16% yield) as a white solid.

R_f 0.47 (Ethyl acetate:Methanol 9:1). **^{1}H NMR** (400 MHz, $CDCl_3$) δ 8.37 (d, *J* = 7.5 Hz, 1H, Ar*H*), 8.21 (d, *J* = 7.5 Hz, 1H, Ar*H*), 7.80-7.70 (m, 2H, Ar*H*), 3.56 (t, *J* = 6.5 Hz, 2H, $CH_2CH_2N_3$), 2.86 (t, *J* = 6.5 Hz, 2H, $CH_2CH_2N_3$). **^{13}C NMR** (101 MHz, $CDCl_3$) δ 167.2, 134.9, 132.3, 131.6, 131.4, 126.8, 115.8, 104.5, 49.4, 42.7, 21.5.

Spectroscopic data was consistent with the values reported in literature.[10]

4-(Prop-2-yn-1-yloxy- but-1-yn-1-yl)-1,2-benziodoxol-3(1H)-one (2b)

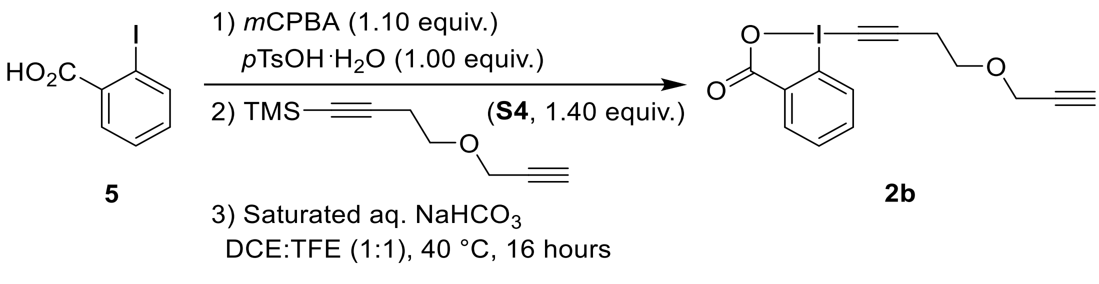

Following a reported procedure,[9] 2-iodobenzoic acid (**5**) (1.75 g, 7.06 mmol, 1.00 equiv.), *para*-toluene sulfonic acid monohydrate (*p*TsOH·H_2O, 1.34 g, 7.06 mmol, 1.00 equiv.) and *meta*-chloroperoxybenzoic acid (*m*CPBA-77%, 1.74 g, 7.77 mmol, 1.10 equiv.) were dissolved in a

[9] Frei, R.; Wodrich, M. D.; Hari, D. P.; Borin, P.-A.; Chauvier, C.; Waser J. *J. Am. Chem. Soc*. **2014**, *136*, 16563.
[10] Abegg, D.; Frei, R.; Cerato, L.; Hari, D. P.; Wang, C.; Waser, J.; Adibekian, A. *Angew. Chem. Int. Ed.* **2015**, *54*, 10852.

mixture of dichloroethane (5.9 mL) and 2,2,2-trifluoroethanol (5.9 mL). After 1 hour stirring at 40 °C, trimethyl(4-(prop-2-yn-1-yloxy)but- 1-yn-1-yl)silane (**S4**) (1.78 g, 9.88 mmol, 1.40 equiv.) was added in one portion. The reaction mixture was stirred for an additional 14 hours at the same temperature, then the resulting suspension was filtered and the volatiles were removed under reduced pressure. The resultant residue was dissolved in dichloromethane (25 mL) and treated with a solution of saturated aqueous sodium bicarbonate (25 mL). The mixture was vigorously stirred for 1 hour, then the two layers were separated and the aqueous layer was extracted with additional portions of dichloromethane (3 x 20 mL). The organic layers were combined, dried over magnesium sulfate; filtered and concentrated under reduced pressure. Purification by column chromatography (SiO_2, Ethyl acetate) afforded 4-(prop-2-yn-1-yloxy-but-1-yn-1-yl)-1,2-benziodoxol-3(1H)-one (**2b**) (177 mg, 0.500 mmol, 7% yield) as a white solid.

Rf 0.10 (Ethyl acetate). **^{1}H NMR** (400 MHz, $CDCl_3$) δ 8.40 (dd, *J* = 7.0, 2.2 Hz, 1H, Ar*H*), 8.26 (dd, *J* = 8.1, 1.1 Hz, 1H, Ar*H*), 7.83-7.70 (m, 2H, Ar*H*), 4.25 (d, *J* = 2.4 Hz, 2H, OCH_2CC), 3.78 (t, *J* = 6.3 Hz, 2H, OCH_2CH_2), 2.90 (t, *J* = 6.3 Hz, 2H, OCH_2CH_2), 2.49 (t, *J* = 2.4 Hz, 1H, CC*H*). **^{13}C NMR** (101 MHz, $CDCl_3$) δ 166.6, 135.0, 132.5, 131.7, 131.5, 126.5, 115.8, 105.9, 79.2, 75.3, 67.4, 58.6, 41.5, 21.9.

Spectroscopic data was consistent with the values reported in literature.[9]

(5-Chloropent-1-ynyl)-1,2-benziodoxol-3(1*H*)-one (2c)

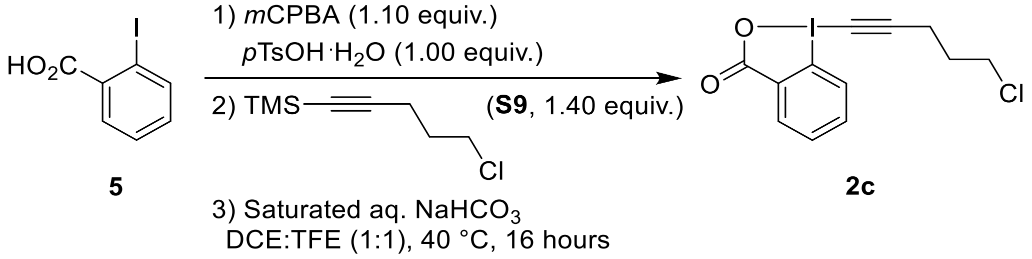

Following a reported procedure,[9] 2-iodobenzoic acid (**5**) (1.18 g, 4.76 mmol, 1.00 equiv.), *para*-toluene sulfonic acid monohydrate (*p*TsOH·H_2O, 0.905 g, 4.76 mmol, 1.00 equiv.) and *meta*-chloroperoxybenzoic acid (*m*CPBA-77%, 1.17 g, 5.24 mmol, 1.10 equiv.) were dissolved in a mixture of dichloroethane (4.0 mL) and 2,2,2-trifluoroethanol (4.0 mL). After 1 hour stirring at 40 °C, (5-chloropent-1-yn-1-yl)trimethylsilane (**S9**) (1.16 g, 6.66 mmol, 1.40 equiv.) was added in one portion. The reaction mixture was stirred for an additional 14 hours at the same temperature, then the resulting suspension was filtered and the volatiles were removed under reduced pressure. The resultant residue was dissolved in dichloromethane (40 mL) and treated with a solution of saturated aqueous sodium bicarbonate (40 mL). The mixture was vigorously stirred for 1 hour, then the two layers were separated and the aqueous layer was extracted with additional portions of dichloromethane (3 x 20 mL). The organic layers were combined, dried over magnesium sulfate; filtered and concentrated under reduced pressure. Purification by column chromatography (SiO_2, Ethyl acetate) afforded (5-chloropent-1-ynyl)-1,2-benziodoxol-3(1*H*)-one (**2c**) (481 mg, 1.38 mmol, 29% yield) as a white solid.

R_f 0.15 (Ethyl acetate). **^{1}H NMR** (400 MHz, $CDCl_3$) δ 8.43-8.39 (m, 1H, Ar*H*), 8.20-8.15 (m, 1H, Ar*H*), 7.80-7.73 (m, 2H, Ar*H*), 3.71 (t, *J* = 6.1 Hz, 2H, ClCH_2CH_2), 2.83 (t, *J* = 6.9 Hz, 2H, CCCH_2CH_2), 2.17-2.08 (m, 2H, ClCH_2CH_2). **^{13}C NMR** (101 MHz, $CDCl_3$) δ 167.0, 134.7, 132.8, 131.9, 131.6, 126.6, 115.8, 106.9, 43.7, 41.0, 30.9, 18.1.

Spectroscopic data was consistent with the values reported in literature.[9]

5-Pentanolethynyl-1,2-benziodoxol-3(1H)-one (2d)

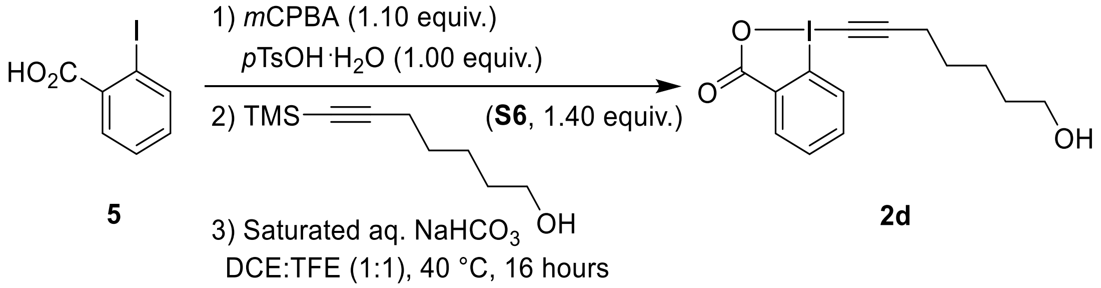

5 **2d**

Following a reported procedure,[9] 2-iodobenzoic acid (**5**) (7.69 g, 31.0 mmol, 1.00 equiv.), *para*-toluene sulfonic acid monohydrate (*p*TsOH·H_2O, 5.90 g, 31.0 mmol, 1.00 equiv.) and *meta*-chloroperoxybenzoic acid (*m*CPBA-77%, 7.64 g, 34.1 mmol, 1.10 equiv.) were dissolved in a mixture of dichloroethane (25.8 mL) and 2,2,2-trifluoroethanol (25.8 mL). After 1 hour stirring at 40 °C, 7-(trimethylsilyl)hept-6-yn-1-ol (**S6**) (8.00 g, 43.4 mmol, 1.40 equiv.) was added in one portion. The reaction mixture was stirred for an additional 14 hours at the same temperature, then the resulting suspension was filtered and the volatiles were removed under reduced pressure. The resultant residue was dissolved in dichloromethane (500 mL) and treated with a solution of saturated aqueous sodium bicarbonate (500 mL). The mixture was vigorously stirred for 1 hour, then the two layers were separated and the aqueous layer was extracted with additional portions of dichloromethane (3 x 150 mL). The organic layers were combined, dried over magnesium sulfate; filtered and concentrated under reduced pressure. Purification by column chromatography (SiO_2, Ethyl acetate:Methanol 95:5) afforded 5-pentanolethynyl-1,2-benziodoxol-3(1H)-one (**2d**) (1.60 g, 4.30 mmol, 14% yield) as a white solid.

R_f 0.24 (Ethyl acetate:Methanol 9:1). **^{1}H NMR** (400 MHz, $CDCl_3$) δ 8.33 (dd, *J* = 7.2, 2.0 Hz, 1H, Ar*H*), 8.15 (d, *J* = 8.0 Hz, 1H, Ar*H*), 7.79-7.64 (m, 2H, Ar*H*), 3.66 (t, *J* = 5.9 Hz, 2H, C*H₂*OH), 2.59 (t, *J* = 6.9 Hz, 2H, CCC*H₂*), 1.73-1.49 (m, 7H, C*H₂* and O*H*). **^{13}C NMR** (101 MHz, $CDCl_3$) δ 167.2, 134.8, 132.2, 131.6, 131.4, 126.6, 115.6, 109.8, 62.2, 38.7, 32.0, 27.9, 25.2, 20.5.

Spectroscopic data was consistent with the values reported in literature.[9]

Rhodamine-EBX (2e)

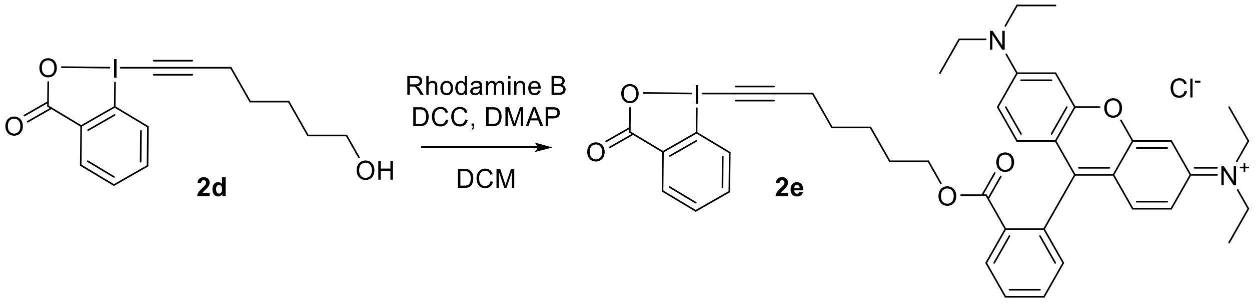

Following a slightly modified procedure,[11] to a solution of 1-(7-hydroxyhept-1-ynyl)-1lambda3,2-benziodoxol-3-one **2d** (20.0 mg, 55.8 µmol, 1.00 equiv), rhodamine B (32.1 mg,

[11] J. Zhu, H. Sun, C. E. Callmann, M. P. Thompson, C. Battistella, M. T. Proetto, A. S. Carlini, N. C. Gianneschi, *Chem. Commun.* **2020**, *56*, 6778–6781.

67.0 μmol, 1.20 equiv) and DMAP (680 μg, 5.60 μmol, 0.100 equiv) in 0.40 mL of DCM, was added slowly a solution of DCC (15.0 mg, 72.6 μmol, 1.30 equiv) in 0.20 mL of DCM at 0 °C in an ice bath. The reaction was stirred for 30 minutes and then the ice bath was removed, and the reaction left to stir for another 6 hours. The mixture was filtered and purified by column chromatography (95:5 DCM: MeOH) to give EBX **2e** (23.0 mg, 29.3 μmol, 53% yield) as a dark purple amorphous solid.

^{1}H NMR (400 MHz, $CDCl_3$) δ 8.33 (dt, *J* = 7.3, 1.8 Hz, 1H, ArH), 8.23 (dd, *J* = 7.9, 1.3 Hz, 1H, ArH), 8.15 (dd, *J* = 8.0, 1.2 Hz, 1H, ArH), 7.81 – 7.62 (m, 4H, ArH), 7.26 (m, 1H, ArH), 7.07 (dd, *J* = 9.5, 5.1 Hz, 2H, ArH), 6.95 (dd, *J* = 9.4, 2.4 Hz, 2H, ArH), 6.81 (d, *J* = 2.5 Hz, 2H, ArH), 4.05 (t, *J* = 6.5 Hz, 2H, CH_2OCO), 3.65 (qd, *J* = 7.4, 6.7, 4.0 Hz, 8H, NC$H_2$$CH_3$), 2.54 (t, *J* = 7.0 Hz, 2H, ICCCH_2), 1.63 – 1.45 (m, 4H, C$H_2$$CH_2$OCO), 1.33 – 1.28 (m, 14H, CH_2, NCH_2CH_3). **^{13}C NMR** (101 MHz, $CDCl_3$) δ 167.2, 165.2, 158.9, 157.8, 155.7 *(2 C under this peak)*, 134.7, 133.6, 133.2, 132.2, 132.1, 131.4, 131.4 *(2 C under this peak)*, 131.2, 130.5, 130.3, 130.1, 126.8, 116.7, 114.5 *(2 C under this peak)*, 113.6 *(2 C under this peak)*, 108.4, 96.5 *(2 C under this peak)*, 65.4, 50.9, 46.3 *(4 C under this peak)*, 28.0, 27.7, 25.2, 20.5, 12.8 *(4 C under this peak)*. *1 C is under the solvent peak.* **HRMS** (ESI/QTOF) m/z: $[M]^+$ Calcd for $C_{42}H_{44}IN_2O_5^+$ 783.2289; Found 783.2300.

2-Iodosylbenzoic acid (26)

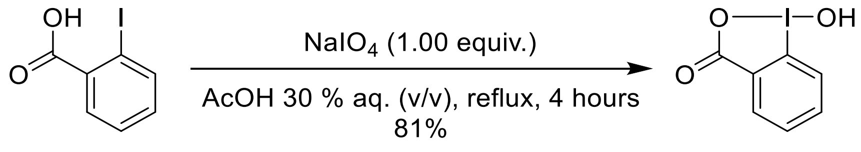

5 **S10**

Following a reported procedure,[12] sodium periodate ($NaIO_4$, 77.2 g, 361 mmol, 1.00 equiv.) and 2-iodobenzoic acid (**5**) (89.5 g, 361 mmol, 1.00 equiv.) were suspended in 30% aqueous acetic acid solution (700 mL). The vigorously stirred mixture was heated and refluxed under air for 4 hours. The reaction mixture was then diluted with cold water (500 mL) and allowed to cool to room temperature. The mixture was stirred at room temperature for 45 minutes, then poured into water (1.5 L). The crude product was collected by filtration, washed with a mixture of ice:water (3 x 300 mL) and cold acetone (3 x 300 mL). After air-drying overnight, 2-iodosylbenzoic acid (**S10**) (74.3 g, 281 mmol, 78% yield) was recovered as a white solid.

^{1}H NMR (400 MHz, DMSO-d_6) δ 8.01 (dd, *J* = 7.5, 1.5 Hz, 1H, Ar*H*), 7.96 (ddd, *J* = 8.5, 7.2, 1.6 Hz, 1H, Ar*H*), 7.85 (dd, *J* = 8.2, 0.9 Hz, 1H, Ar*H*), 7.70 (td, *J* = 7.3, 1.0 Hz, 1H, Ar*H*). **^{13}C NMR** (101 MHz, DMSO-d_6) δ 167.7, 134.5, 131.5, 131.1, 130.4, 126.3, 120.4.

Spectroscopic data was consistent with the values reported in literature.[12]

1-[(Trimethylsilyl)ethynyl]-1,2-benziodoxol-3(1*H*)-one (TMS-EBX, 2f)

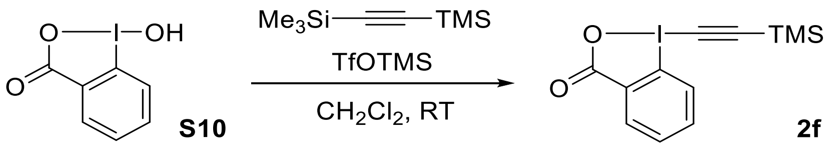

S10 **2f**

---

[12] Kraszkiewicz, L.; Skulski, L. *Arkivoc* **2003**, *6*, 120.

Following a slight modification of the reported procedure,[3] trimethylsilyl triflate (5.54 mL, 30.7 mmol, 1.10 equiv) was added to a suspension of 2-iodosylbenzoic acid **S10** (7.36 g, 28.0 mmol, 1.00 equiv) in CH_2Cl_2 (85 mL) at RT. The resulting yellow mixture was stirred for 1 h, followed by the dropwise addition of bis(trimethylsilyl)acetylene (6.98 mL, 30.7 mmol, 1.10 equiv). The resulting suspension was stirred for 6 h at RT, during this time a white solid was formed. A saturated solution of $NaHCO_3$ was then added and the mixture was stirred vigorously until completely solubilization of the white solid. The two layers were separated and the combined organic extracts were washed with sat. $NaHCO_3$, dried over $MgSO_4$, filtered and evaporated under reduce pressure. Recrystallization from acetonitrile (5 mL) afforded 1-[(trimethylsilyl)ethynyl]-1,2-benziodoxol-3(1*H*)-one **2f** (7.17 g, 20.8 mmol, 74%) as a colorless solid. **Mp:** 143-145°C (dec); **^{1}H NMR**(400 MHz, Chloroform-*d*) δ 8.42 (dd, *J*= 6.4, 1.9 Hz, 1 H; Ar*H*), 8.19 (m, 1 H; Ar*H*), 7.78 (m, 2 H; Ar*H*), 0.32 (s, 9 H; TMS); **^{13}C NMR** (100 MHz, $CDCl_3$) *δ* 166.4, 134.9, 132.6, 131.7, 131.4, 126.1, 117.2, 115.4, 64.2, -0.5; **IR** v 3389 (w), 2967 (w), 1617 (s), 1609 (s), 1562 (m), 1440 (w), 1350 (m), 1304 (w), 1254 (w), 1246 (w), 1112 (w), 1008 (w), 852 (s), 746 (m), 698 (m), 639 (m). The characterization data for compound **2b** corresponded to the reported values.[13]

1-[(Tri*iso*propylsilyl)ethynyl]-1,2-benziodoxol-3(1H)-one (2h)

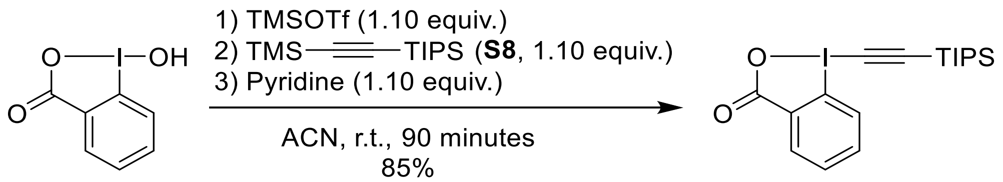

S10 **2h**

Following a reported procedure,[8] a cooled solution of trimethylsilyl trifluoromethanesulfonate (TMSOTf, 19.9 mL, 110 mmol, 1.10 equiv.) was added dropwise to a stirred suspension of 2-iodosylbenzoic acid (**S10**) (26.4 g, 100.0 mmol, 1.00 equiv.) in acetonitrile (350 mL) at 0 °C. The mixture was then allowed to warm to room temperature and was stirred for 15 minutes. Then trimethylsilyl(tri*iso*propylsilyl)acetylene (**S8**) (28.0 g, 110 mmol, 1.10 equiv.) was added dropwise to the reaction mixture. After 30 minutes, pyridine (9.8 mL, 122 mmol, 1.10 equiv.) was added dropwise and, 15 minutes later, the reaction mixture was concentrated under reduced pressure. The collected solid was dissolved in dichloromethane (250 mL) and washed with a 1.0 N aqueous hydrochloric acid (150 mL). The aqueous layer was extracted with dichloromethane (250 mL), then the combined organic layers were washed with a saturated aqueous sodium bicarbonate (2 x 250 mL), dried over magnesium sulfate, filtered and concentrated *in vacuo*. The resulting solid was then recrystallized from acetonitrile and washed with hexanes (2 x 40 mL) to yield pure TIPS-EBX (**2h**) (32.1 g, 74.9 mmol, 75% yield) as white crystals.

^{1}H NMR (400 MHz, $CDCl_3$) δ 8.43 (dd, *J* = 5.9, 3.3 Hz, 1H, Ar*H*), 8.29 (dd, *J* = 6.0, 3.3 Hz, 1H, Ar*H*), 7.76 (dd, *J* = 5.9, 3.3 Hz, 2H, Ar*H*), 1.33-1.05 (m, 21H, TIPS). **^{13}C NMR** (101 MHz, $CDCl_3$) δ 166.4, 134.5, 132.3, 131.4, 131.4, 126.1, 115.6, 113.9, 64.7, 18.4, 11.1.

Spectroscopic data was consistent with the values reported in literature.[8]

[13] Hari, D. P.; Waser, J. *J. Am. Chem. Soc*. **2016**, *138* (7), 2190–2193.

1-Phenylethynyl-1,2-benziodoxol-3(1*H*)-one (2i)

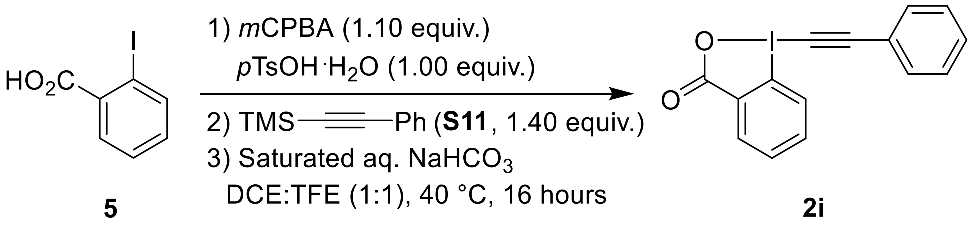

Following a modified procedure,[14] 2-iodobenzoic acid (**5**) (1.21 g, 4.87 mmol, 1.00 equiv.), *para*-toluene sulfonic acid monohydrate (*p*TsOH·H_2O, 0.926 g, 4.87 mmol, 1.00 equiv.) and *meta*-chloroperoxybenzoic acid (*m*CPBA-77%, 1.20 g, 5.36 mmol, 1.10 equiv.) were dissolved in a mixture of dichloroethane (4.0 mL) and 2,2,2-trifluoroethanol (4.0 mL). After 1 hour stirring at 40 °C, trimethyl(phenylethynyl)silane (**S11**) (1.19 g, 6.82 mmol, 1.40 equiv.) was added in one portion. The reaction mixture was stirred for an additional 14 hours at the same temperature, then the resulting suspension was filtered and the volatiles were removed under reduced pressure. The resultant residue was dissolved in dichloromethane (40 mL) and treated with a solution of saturated aqueous sodium bicarbonate (40 mL). The mixture was vigorously stirred for 1 hour, then the two layers were separated and the aqueous layer was extracted with additional portions of dichloromethane (3 x 20 mL). The organic layers were combined, dried over magnesium sulfate; filtered and concentrated under reduced pressure. The resulting solid was then recrystallized from acetonitrile and washed with acetonitrile (2 x 20 mL) to yield pure 1-phenylethynyl-1,2-benziodoxol-3(1*H*)-one (**2i**) (648 mg, 1.86 mmol, 38% yield) as a white solid.

^{1}H NMR (400 MHz, $CDCl_3$) δ 8.45-8.41 (m, 1H, Ar*H*), 8.28-8.23 (m, 1H, Ar*H*), 7.81-7.75 (m, 2H, Ar*H*), 7.62-7.59 (m, 2H, Ar*H*), 7.51-7.41 (m, 3H, Ar*H*). **^{13}C NMR** (101 MHz, $CDCl_3$) δ 166.7, 135.0, 133.0, 132.6, 131.7, 131.5, 130.9, 128.9, 126.4, 120.7, 116.3, 106.7, 50.3.

Spectroscopic data was consistent with the values reported in literature.[14]

2-Iodosyl-5-nitrobenzoic acid (S12) and 2-iodosyl-3-nitrobenzoic acid (S13)

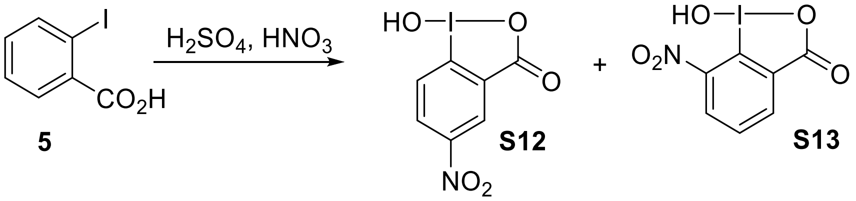

Following a reported procedure,[15] fuming nitric acid (3.3 mL) was acid to 2-iodobenzoic acid (**5**) (5.0 g, 20 mmol, 1 equiv) in concentrated H_2SO_4 (6.7 mL). The reaction was equipped with a cooler and a nitrous vapor trap and was heated at 100°C for 1 h. The reaction mixture was then poured in ice-water and filtered. The resulting solid was refluxed in water (50 mL) and filtered. A second crop of precipitate was filtered from the mother liquors. Both solids were combined, washed with acetone (10 mL) and dried under vacuum to afford **S12** (2.19 g, 7.10 mmol, 36 %). The mother liquors were reduced to one third and then kept at 4°C, the resulting precipitate was filtered, washed with acetone (10 mL) and dried under vacuum to afford **S13** (630 mg, 2.04 mmol, 10 %). **S12**: ^{1}H NMR (400 MHz, DMSO) δ 8.73 (dd, 1H, *J* = 8.8, 2.6 Hz,

[14] Brand, J. P.; Chevalley, C.; Scopelliti, R.; Waser, J. *Chem. - Eur. J.* **2012**, *18*, 5655.
[15] Morrison, G. F.; Hooz, J. *J. Org. Chem.* **1970,** *35*, 1196.

ArH), 8.58 (d, 1H, *J* = 2.4 Hz, ArH), 8.54 (br s, 1H, OH), 8.11 (d, 1H, *J* = 8.8 Hz, ArH). **S13**: ^{1}H NMR (400 MHz, DMSO) δ 7.92 (dd, 1 H, *J* = 7.9, 1.5 Hz), 7.79 (m, 1 H), 7.67 (m, 1 H).

Spectroscopic data was consistent with the values reported in literature.[13]

5-nitro-1-(pent-1-yn-1-yl)-1,2-benziodoxol-3(1H)-one (2j)

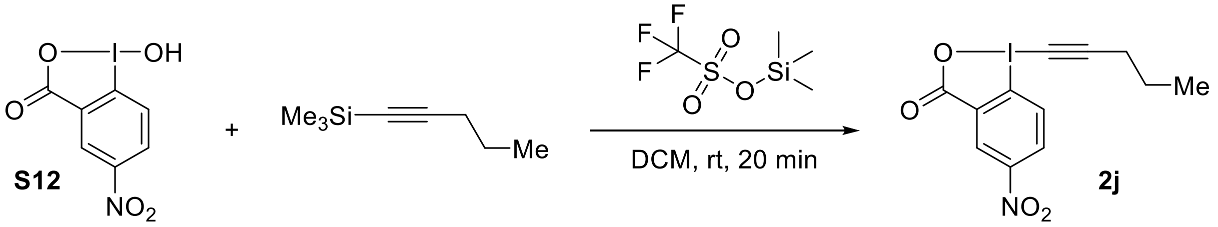

Following a slightly modified procedure,[16] to a solution of BI-OH (**S12**) (250 mg, 809 µmol, 1.00 equiv) in dry DCM (8.00 mL) in a flame-dried round-bottom flask, was added trimethylsilyl trifluoromethanesulfonate (198 mg, 161 µL, 890 µmol, 1.10 equiv) dropwise at room temperature and the reaction mixture was stirred for 1.5 h. After this time, trimethyl(pent-1-ynyl)silane (125 mg, 163 µL, 890 µmol, 1.10 equiv) was added and the mixture was stirred for 6 h at room temperature. The reaction mixture was then quenched with saturated aqueous $NaHCO_3$ solution and extracted with DCM (3 times). The combined organic extracts were dried over $MgSO_4$, filtered and concentrated under reduced pressure. The residue was purified by column chromatography (DCM:MeOH 100:0 to 80:20) to give EBX **2j** (70.0 mg, 195 µmol, 24% yield) as an off-white solid.

^{1}H NMR (400 MHz, DMSO) δ 8.68 – 8.60 (m, 2H, ArH), 8.52 – 8.44 (m, 1H, ArH), 2.69 (t, *J* = 7.1 Hz, 2H, C*H₂*), 1.65 (h, *J* = 7.3 Hz, 2H, C*H₂*), 1.04 (t, *J* = 7.3 Hz, 3H, C*H₃*). **^{13}C NMR** (101 MHz, DMSO) δ 164.4, 150.2, 134.3, 129.5, 128.6, 124.8, 122.7, 109.1, 39.9, 21.6, 21.2, 13.4. **HRMS** (ESI/QTOF) m/z: $[M + H]^+$ Calcd for $C_{12}H_{11}INO_4^+$ 359.9727; Found 359.9711.

Preparation of (S15)

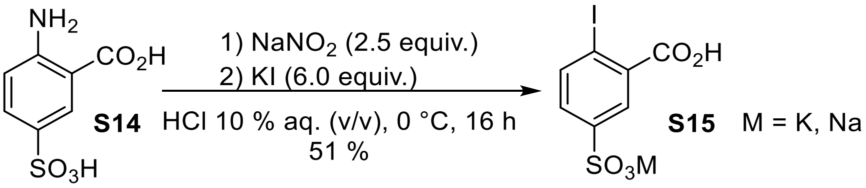

Following a reported procedure,[17] 2-amino-5-sulfobenzoic acid **S14** (4.34 g, 20.0 mmol, 1.0 equiv.) was suspended in a 10% aqueous hydrochloric acid solution (100 mL) and cooled to 0 °C. A cooled solution of sodium nitrite ($NaNO_2$, 3.45 g, 50.0 mmol, 2.5 equiv.) in water (18 mL) was slowly added over a period of 45 minutes. After an additional 30 minutes stirring at this temperature, a cooled solution of potassium iodide (KI, 19.9 g, 120 mmol, 6.0 equiv.) in water (75 mL) was slowly added over a period of 1 hour at 0 °C. The resulting dark solution was allowed to warm to room temperature and stirred for 16 hours. Then, the reaction was slowly quenched by small portions of sodium bisulfite (around 14 g) until the solution persistently turned as a light-yellow[18] suspension. The resulting suspension was filtered, washed with

---

[16] Huang, H.; Zhang, G.; Gong, L.; Zhang, S.; Chen, Y. *J. Am. Chem. Soc*. **2014**, *136*, 2280−2283.
[17] A. Kommreddy, M. S. Bowsher, M. R. Gunna, K. Botha, T. K. Vinod,*Tetrahedron Lett.* **2008**, *49*, 4378.
[18] a) T. Harschneck, S. Hummel, S. Kirsch, P. Klahn, *Chem. Eur. J.* **2012**, *18*, 1187; b) A. Bredenkamp, F. Mohr, S. Kirsch, *Synthesis*, **2015**, *47*, 1937.

acetone (3 x 100 mL) and dichloromethane (50 mL) to afford a yellow pale solid. The collected solid was then recrystallized from water and washed with cold water (2 x 50 mL), acetone (2 x 50 mL) and dichloromethane (2 x 50 mL) to yield pure **S15** (3.71 g, 10.1 mmol, 51% yield) as a pale-yellow solid.

^{1}H NMR (400 MHz, DMSO-*d*$_6$) δ 7.95 (d, *J* = 8.1 Hz, 1H, ArH), 7.90 (d, *J* = 2.0 Hz, 1H, ArH), 7.41 (dd, *J* = 8.1, 2.1 Hz, 1H, ArH). **^{13}C NMR** (101 MHz, DMSO-*d*$_6$) δ 167.9, 147.8, 140.5, 136.4, 129.5, 127.3, 94.8.

Spectra data was consistent with the values reported in literature.[17]

Preparation of (S16)

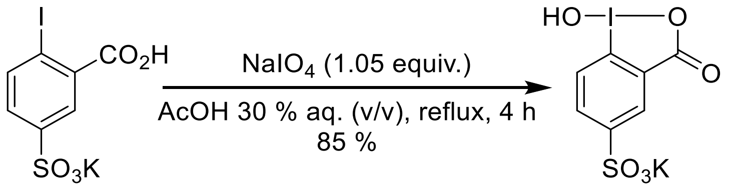

S15 **S16**

Following a reported procedure,[19] **S15** (1.75 g, 8.17 mmol, 1.00 equiv.) and sodium periodate ($NaIO_4$, 2.85 g, 7.78 mmol, 1.05 equiv.) were suspended in 30% aqueous acetic acid solution (14 mL). The vigorously stirred mixture was heated and refluxed under air for 4 h. The reaction mixture was allowed to cool to room temperature and placed under vacuum. The resulting precipitate was filtered and washed with acetone (3 x 100 mL) and dichloromethane (100 mL). The collected solid was dissolved in methanol, filtered and concentrated under pressure to afford pure potassium 2-iodosyl-5-sulfobenzoate, **S16** (2.52 g, 6.59 mmol, 85% yield) as a white solid.

^{1}H NMR (400 MHz, DMSO-*d*$_6$) δ 8.18 (d, *J* = 1.8 Hz, 1H, ArH), 8.12 (dd, *J* = 8.3, 1.9 Hz, 1H, ArH), 7.80 (d, *J* = 8.3 Hz, 1H, ArH). **^{13}C NMR** (101 MHz, DMSO-*d*$_6$) δ 167.5 (C=O), 151.1 (ArC), 132.1 (ArC), 130.7 (ArC), 128.5 (ArC), 126.3 (ArC), 119.1 (ArC). **HRMS** (ESI/QTOF) m/z: [M-K]$^-$ Calcd for $C_7H_4IO_6S^-$ 342.8779; Found 342.8779.

Spectroscopic data was consistent with the values reported in literature.[19]

Preparation of $C_{14}H_{29}$-EBX-SO_3M (2k)

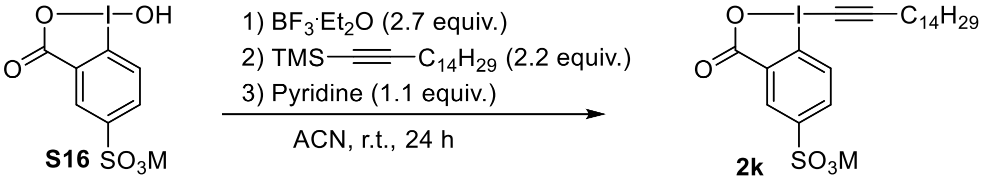

S16 **2k**

Following a reported procedure,[19] a flame-dried 25 mL round-bottomed flask under nitrogen was charged with potassium **S16** (0.50 g, 1.30 mmol, 1.0 equiv.) and acetonitrile (10.0 mL). A cooled solution of boron trifluoride etherate ($BF_3 \cdot Et_2O$, 0.43 mL, 3.5 mmol, 2.7 equiv.) was added dropwise at room temperature and the reaction was stirred for 2 h. Hexadec-1-yn-1-yltrimethylsilane (0.843 g, 2.86 mmol, 2.2 equiv.) was then slowly added and the resulting mixture was stirred for an additional 18 h. Then, pyridine (115 μL, 1.43 mmol, 1.1 equiv.) was

[19] A. Kumar Mishra, R. Tessier, D. Prasad Hari, J. Waser, *Angew. Chem. Int. Ed.* **2021**, *60*, 17963 – 17968.

added dropwise and the reaction mixture was stirred for 2 h. The resulting precipitate was filtered and washed with acetonitrile (3 x 15 mL) and pentane (3 x 10 mL). The crude mixture was purified by RP-HPLC using C18 column (gradient: 100 H_2O to 95% ACN/H_20 for 30 mins) to obtain 60% pure **2k** (0.45 g, 0.78 mmol,) as a white solid. HPLC-Gradient: Method 7. Yield for **2k** calculated based on K salt.

R_f 0.30 (dichloromethane:methanol, 9:1). **^{1}H NMR** (400 MHz, DMSO-*d6*) δ 8.29 (d, *J* = 2.1 Hz, 1H, ArH), 8.18 (d, *J* = 8.5 Hz, 1H, ArH), 8.02 (dd, *J* = 8.4, 2.1 Hz, 1H, ArH), 2.67 (t, *J* = 7.0 Hz, 2H, CH_2), 1.59 (q, *J* = 7.1 Hz, 2H, CH_2), 1.49 – 1.12 (m, 22H, 11×CH_2), 0.85 (t, *J* = 8.0 Hz , 3H, CH_3). **^{13}C NMR** (101 MHz, DMSO-*d6*) *δ* 166.3 (C=O), 152.0 (ArC), 132.5 (ArC), 132.0 (ArC), 128.6 (ArC), 127.3 (ArC), 115.7 (CC), 108.6 (CC), 31.7 (CH_2), 29.5 (5×CH_2), 29.4 (CH_2), 29.2 (CH_2), 28.9 (CH_2), 28.7 (CH_2), 28.1 (CH_2), 22.5 (CH_2), 20.1 (CH_2), 14.4 (CH_3). **HRMS** (ESI/QTOF) m/z: $[M-K]^-$ Calcd for $C_{23}H_{32}IO_5S^-$ 547.1021; Found 547.1019.

Spectroscopic data was consistent with the values reported in literature.[19]

4. Synthesis of tetramers

Solid-Phase Peptide Synthesis (SPPS):

Peptides were synthesized on an MultiPep RSi parallel peptide synthesizer (Intavis) using standard Fmoc SPPS-chemistry and Rink Amide MBHA resin (0.337 mmol/g resin, 0.05 mmol scale). Each coupling cycle was initiated by Fmoc deprotection on the Rink Amide MBHA resin, achieved by shaking the resin with 800 µL of 20% v/v piperidine in dimethylformamide (DMF) at 400 rpm, over 5 minutes twice. Then the resin was washed with DMF (6000 µL x7). The coupling was carried out by shaking Rink Amide MBHA resin with a Fmoc-protected monomer (4.0 equiv.), 2-(1H-benzotriazol-1-yl)-1,1,3,3-tetramethyluronium hexafluorophosphate (HBTU, 4.0 equiv.), hydroxybenzotriazole (HOBt, 4.0 equiv.) and *N*-Methylmorpholine (6.0 equiv.), in DMF (1.3 mL), at 400 rpm, over 30 minutes twice. Alternatively, HATU (4.0 equiv.) instead of the HBTU and HOBt combination was used for the coupling. Capping using Cap Mixture (5% v/v Ac_2O and 6% v/v 2,6-lutidine in DMF) was carried out at the end of each cycle, followed by a DMF wash (6000 µL x7). The synthesis was finished by deprotection of Fmoc using 20% v/v piperidine in dimethylformamide at 400 rpm, over 5 minutes two times. The N-terminus was either left unprotected or was acylated or fluoresceinated. Acetylation of the N-terminal was achieved by incubating the resin with Cap Mixture three times. Fluoresceination was achieved by shaking Rink Amide MBHA resin with a 5(6)-carboxyfluorescein (2.0 equiv.), 2-(1H-benzotriazol-1-yl)-1,1,3,3-tetramethyluronium hexafluorophosphate (HATU, 2.0 equiv.) and *N*-Methylmorpholine (3.0 equiv.), in DMF (1.3 mL), at 400 rpm, over 30 minutes. Next, washing steps were performed with dimethylformamide (5 x 3 mL). Finally, resin was dried with dichloromethane (5 x 3 mL).

Peptide cleavage and deprotection:

Peptides were deprotected and cleaved from the resin by treatment with 2.5% v/v water and 2.5% v/v Triisopropyl silane in neat trifluoroacetic acid (5 mL). The resulting mixture was shaken for 2 hours, at room temperature. The resin was removed by filtration and peptides were precipitated in cold diethyl ether (50 mL), followed by a 2 hours incubation at -20 °C. Peptides were pelleted by centrifugation at 4000 rpm, for 5 minutes. Finally, the mother liquors were carefully removed.

Peptide purification and analysis:

Peptides were dissolved in water with a minimum amount of organic co-solvent (acetonitrile, dimethylformamide or dimethyl sulfoxide). Peptides were then purified on preparative RP-HPLC with a gradient of 5 minutes of solvent A (100% Water with 0.1% TFA) and then 20 minutes gradient to 100% solvent B (5% Water/AcCN with 0.1% TFA). Fractions containing the desired peptide were lyophilized. Peptides were obtained as TFA salts, one molecule of TFA was assumed for every basic amino acid residue - Lys (K), Arg (R) and His (H). The purity was assessed by HPLC-MS analysis. At the same time, low-resolution mass spectrometric measurements were also acquired. High-resolution mass spectrometry measurements of the purified peptide were performed by ESI on a Thermo Orbitrap Elite.

MS/MS fragmentation:

The regioselectivity of the bioconjugation was confirmed using MS/MS analysis using a LTQ Orbitrap Elite instrument (Thermofisher). The precursor ion was selected in the LTQ using an isolation window of 3 *m/z* and submitted to fragmentation using CID, HCD and ETD activation techniques. The spectra were analyzed using the Apm2S tool available at ms.epfl.ch or

eln.epfl.ch.[20] For the calculations, the experimental spectra were compared to theoretical peaks using a peak threshold set to 0.5%, a mass error of 5 ppm, a minimal similarity for the isotopic pattern of 70% and a zone set to -0.5 to 4.5 u relative to the calculated monoisotopic mass. The linker was added as a variable modification and fragment ions (mainly b, y and a but also neutral losses) with and without linker were matched. ETD fragmentation, being known to better preserve labile modifications, was also performed and in this case, c and z fragments were searched. The fragments containing the bioconjugate mass are indicated in **bold**.

[20] a) J. S. Desport, G. Frache, L. Patiny *Ref Rapid Commun Mass Spectrom.* **2020**, *e8652*; b) D. Ortiz, N Gasilova, F. Sepulveda, L. Patiny, P. J. Dyson, L. Menin, *Ref Rapid Commun Mass Spectrom.* **2020.**

AFYA-NH$_2$ (1a)

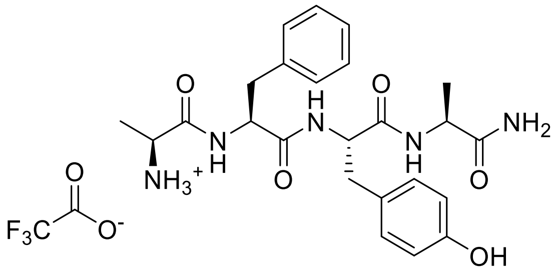

^{1}H NMR (400 MHz, MeOD) δ 7.30 – 7.15 (m, 5H, Ar*H*), 7.10 – 7.02 (m, 2H, Ar*H*), 6.73 – 6.65 (m, 2H, Ar*H*), 4.63 (dd, *J* = 9.1, 5.5 Hz, 1H, C*H*-Tyr), 4.53 (dd, *J* = 8.1, 6.2 Hz, 1H, C*H*-Tyr), 4.29 (q, *J* = 7.2 Hz, 1H, C*H*-Ala-C-Term), 3.83 (q, *J* = 7.0 Hz, 1H, C*H*-Ala-N-Term), 3.11 (dd, *J* = 14.0, 5.6 Hz, 1H, CH_2-Phe), 3.03 (dd, *J* = 13.9, 6.2 Hz, 1H, CH_2-Tyr), 2.87 (td, *J* = 14.0, 8.7 Hz, 2H, CH_2-Tyr, CH_2-Phe), 1.42 (d, *J* = 7.1 Hz, 3H, CH_3), 1.34 (d, *J* = 7.2 Hz, 3H, CH_3). **^{13}C NMR** (101 MHz, MeOD) δ 177.2, 172.9 *(2 C under this peak)*, 170.9, 157.3, 138.2, 131.5, 130.2, 129.5, 128.8, 127.9, 116.3, 56.3, 56.2, 50.1, 50.0, 38.7, 38.0, 18.3, 17.7.[21] **HRMS** (nanochip-ESI/LTQ-Orbitrap) m/z: $[M + H]^+$ Calcd for $C_{24}H_{32}N_5O_5^+$ 470.2398; Found 470.2392.

ALYA-NH$_2$ (1b)

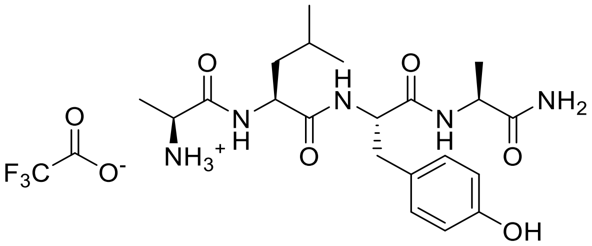

^{1}H NMR (400 MHz, MeOD) δ 7.10 – 7.02 (m, 2H, Ar*H*), 6.73 – 6.65 (m, 2H, Ar*H*), 4.53 (dd, *J* = 8.3, 6.1 Hz, 1H, C*H*-Tyr), 4.39 (dd, *J* = 8.0, 7.1 Hz, 1H, C*H*-Leu), 4.31 (q, *J* = 7.1 Hz, 1H, C*H*-Ala-C-Term), 3.90 (q, *J* = 7.0 Hz, 1H, C*H*-Ala-N-Term), 3.06 (dd, *J* = 14.0, 6.1 Hz, 1H, CH_2-Tyr), 2.87 (dd, *J* = 14.0, 8.3 Hz, 1H, CH_2-Tyr), 1.66 – 1.56 (m, 1H, $CH_2CH(CH_3)_2$), 1.55 – 1.50 (m, 2H, $CH_2CH(CH_3)_2$), 1.45 (d, *J* = 7.1 Hz, 3H, CH_3), 1.34 (d, *J* = 7.2 Hz, 3H, CH_3), 0.94 (d, *J* = 6.5 Hz, 3H, $CH_2CH(CH_3)_2$), 0.91 (d, *J* = 6.4 Hz, 3H, $CH_2CH(CH_3)_2$). **^{13}C NMR** (101 MHz, MeOD) δ 175.7, 172.7, 171.6, 169.5, 155.9, 130.0, 127.4, 114.9, 54.6, 52.0, 48.7 (*2 C under this peak*), 40.5, 36.5, 24.4, 21.9, 20.5, 16.9, 16.3.21[21] **HRMS** (ESI/QTOF) m/z: $[M + H]^+$ Calcd for $C_{21}H_{34}N_5O_5^+$ 436.2554; Found 436.2553.

[21] ^{13}C NMR peaks of TFA were not resolved.

AWYA-NH_2 (1c)

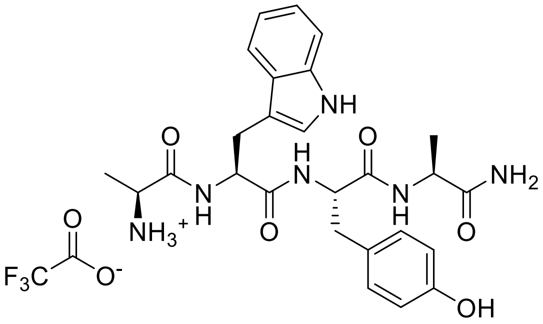

^{1}H NMR (400 MHz, MeOD) δ 7.60 (dt, *J* = 7.9, 1.1 Hz, 1H, Ar*H*), 7.33 (dt, *J* = 8.1, 0.9 Hz, 1H, Ar*H*), 7.14 – 7.06 (m, 2H, Ar*H*), 7.02 (ddd, *J* = 8.0, 7.1, 1.0 Hz, 1H, Ar*H*), 6.99 – 6.92 (m, 2H, Ar*H*), 6.69 – 6.63 (m, 2H, Ar*H*), 4.68 (dd, *J* = 7.9, 6.1 Hz, 1H, C*H*-Tyr), 4.52 – 4.44 (m, 1H, C*H*-Trp), 4.26 (q, *J* = 7.2 Hz, 1H, C*H*-Ala-C-Term), 3.83 (q, *J* = 7.0 Hz, 1H, C*H*-Ala-N-Term), 3.24 (ddd, *J* = 14.7, 6.1, 0.9 Hz, 1H, CH_2-Trp), 3.16 – 3.07 (m, 1H, CH_2-Trp), 2.90 (dd, *J* = 13.9, 6.2 Hz, 1H, CH_2-Tyr), 2.81 (dd, *J* = 13.9, 7.8 Hz, 1H, CH_2-Tyr), 1.39 (d, *J* = 7.0 Hz, 3H, CH_3), 1.30 (d, *J* = 7.2 Hz, 3H, CH_3). **^{13}C NMR** (101 MHz, MeOD) δ 177.2, 173.3, 172.8, 170.9, 157.4, 138.1, 131.5, 128.7, 128.6, 124.7, 122.6, 119.9, 119.3, 116.3, 112.4, 110.6, 56.1, 55.8, 50.1, 49.6, 37.8, 28.9, 18.3, 17.6.[21] **HRMS** (ESI/QTOF) m/z: $[M + H]^+$ Calcd for $C_{26}H_{33}N_6O_5^+$ 509.2507; Found 509.2515.

ARYA-NH_2 (1d)

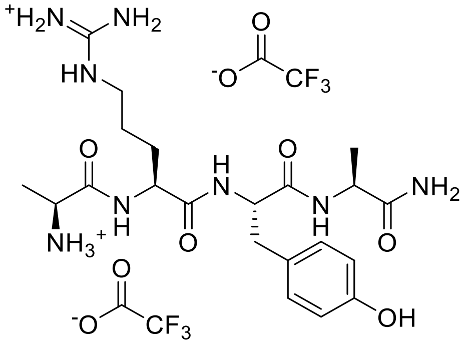

^{1}H NMR (400 MHz, MeOD) δ 7.13 – 7.04 (m, 2H, Ar*H*), 6.74 – 6.65 (m, 2H, Ar*H*), 4.57 (dd, *J* = 8.6, 5.9 Hz, 1H, C*H*-Tyr), 4.35 (dd, *J* = 7.4, 6.3 Hz, 1H, C*H*-Arg), 4.29 (q, *J* = 7.1 Hz, 1H, C*H*-Ala-C-Term), 3.94 (q, *J* = 7.0 Hz, 1H, C*H*-Ala-N-Term), 3.17 (t, *J* = 7.0 Hz, 2H, $CH_2CH_2CH_2NH$), 3.07 (dd, *J* = 14.0, 5.9 Hz, 1H, CH_2-Tyr), 2.86 (dd, *J* = 14.0, 8.6 Hz, 1H, CH_2-Tyr), 1.86 – 1.73 (m, 1H, $CH_2CH_2CH_2NH$), 1.73 – 1.63 (m, 1H, $CH_2CH_2CH_2NH$), 1.58 (tdd, *J* = 11.2, 8.3, 4.6 Hz, 2H, $CH_2CH_2CH_2NH$), 1.45 (d, *J* = 7.1 Hz, 3H, CH_3), 1.35 (d, *J* = 7.2 Hz, 3H, CH_3). **^{13}C NMR** (101 MHz, MeOD) δ 177.2, 173.2, 173.1, 171.0, 158.6, 157.4, 131.4, 128.7, 116.3, 56.1, 54.4, 50.2, 50.1, 41.9, 38.0, 30.3, 26.0, 18.4, 17.6.[21] **HRMS** (ESI/QTOF) m/z: $[M + H]^+$ Calcd for $C_{21}H_{35}N_8O_5^+$ 479.2725; Found 479.2731.

AHYA-NH_2 (1e)

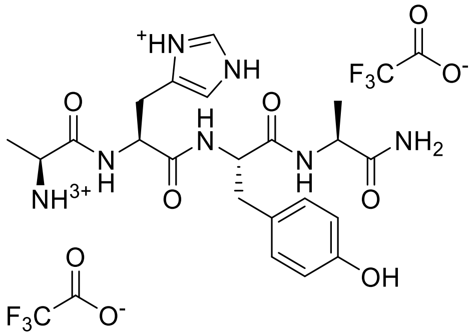

^{1}H NMR (400 MHz, MeOD) δ 8.77 (d, J = 1.4 Hz, 1H, ArH), 7.31 (d, J = 1.4 Hz, 1H, ArH), 7.13 – 7.05 (m, 2H, ArH), 6.74 – 6.66 (m, 2H, ArH), 4.70 (t, J = 6.5 Hz, 1H, CH-Tyr), 4.54 (dd, J = 8.8, 5.9 Hz, 1H, CH-His), 4.31 (q, J = 7.2 Hz, 1H, CH-Ala-C-Term), 3.93 (q, J = 7.1 Hz, 1H, CH-Ala-N-Term), 3.27 – 2.99 (m, 3H, CH_2-His, CH_2-Tyr), 2.86 (dd, J = 14.1, 8.9 Hz, 1H, CH_2-Tyr), 1.43 (d, J = 7.1 Hz, 3H, CH_3), 1.36 (d, J = 7.2 Hz, 3H, CH_3). **^{13}C NMR** (101 MHz, MeOD) δ 177.2, 173.6, 171.3, 171.0, 157.5, 135.1, 131.3, 130.3, 128.6, 118.7, 116.3, 56.4, 53.5, 50.3, 50.1, 37.9, 28.3, 18.2, 17.6.[21] **HRMS** (nanochip-ESI/LTQ-Orbitrap) m/z: $[M + H]^+$ Calcd for $C_{21}H_{30}N_7O_5^+$ 460.2303; Found 460.2308.

AKYA-NH_2 (1f)

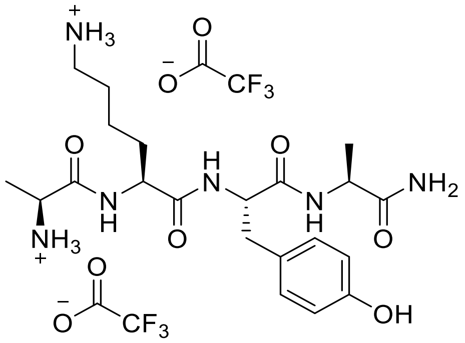

^{1}H NMR (400 MHz, MeOD) δ 7.22 – 6.98 (m, 2H, ArH), 6.82 – 6.56 (m, 2H, ArH), 4.56 (dd, J = 8.6, 6.0 Hz, 1H, CH-Tyr), 4.39 – 4.21 (m, 2H, CH-Lys, CH-Ala-C-Term), 3.93 (q, J = 7.0 Hz, 1H, CH-Ala-N-Term), 3.07 (dd, J = 14.1, 5.9 Hz, 1H, CH_2-Tyr), 2.97 – 2.81 (m, 3H, CH_2-Tyr, $CH_2CH_2CH_2CH_2NH_2$), 1.77 (ddt, J = 13.3, 9.9, 6.5 Hz, 1H, $CH_2CH_2CH_2CH_2NH_2$), 1.65 (qdd, J = 8.2, 5.6, 3.3 Hz, 3H, $CH_2CH_2CH_2CH_2NH_2$), 1.45 (d, J = 7.0 Hz, 3H, CH_3), 1.35 (d, J = 7.1 Hz, 5H, $CH_2CH_2CH_2CH_2NH_2$, CH_3). **^{13}C NMR** ^{13}C NMR (101 MHz, MeOD) δ 177.2, 173.3, 173.2, 170.9, 157.4, 131.4, 128.7, 116.3, 56.1, 54.7, 50.2, 50.1, 40.4, 38.0, 32.6, 28.1, 23.5, 18.4, 17.6.[21] **HRMS** (ESI/QTOF) m/z: $[M]^+$ Calcd for $C_{21}H_{36}N_6O_5^{+2}$ 226.1368; Found 226.1371.

ALYA-NH2 (1g)

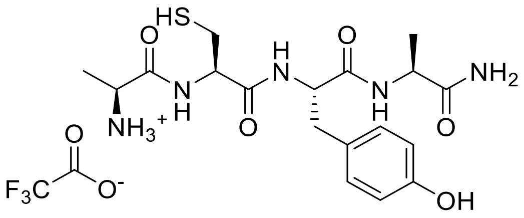

^{I1}H NMR (400 MHz, MeOD) δ 7.10 – 7.04 (m, 2H, Ar*H*), 6.74 – 6.66 (m, 2H, Ar*H*), 4.55 (dd, *J* = 8.6, 5.9 Hz, 1H, C*H*-Tyr), 4.48 (dd, *J* = 7.1, 5.9 Hz, 1H, C*H*-Cys), 4.31 (q, *J* = 7.2 Hz, 1H, C*H*-Ala-C-Term), 3.94 (q, *J* = 7.0 Hz, 1H, C*H*-Ala-N-Term), 3.07 (dd, *J* = 14.0, 5.9 Hz, 1H, C*H2*-Tyr), 2.92 – 2.82 (m, 2H, C*H2*-Tyr, C*H2*-Cys), 2.78 (dd, *J* = 13.9, 7.1 Hz, 1H, C*H2*-Cys), 1.45 (d, *J* = 7.1 Hz, 3H, C*H3*), 1.34 (d, *J* = 7.2 Hz, 3H, C*H3*). **^{13}C NMR** (101 MHz, MeOD) δ 177.4, 173.0, 171.6, 171.0, 157.4, 131.4, 128.7, 116.3, 57.1, 56.3, 50.1, 49.7, 37.8, 26.9, 18.3, 17.7.[21] **HRMS** (nanochip-ESI/LTQ-Orbitrap) m/z: $[M + H]^+$ Calcd for $C_{18}H_{28}N_5O_5S^+$ 426.1806; Found 426.1815.

ASYA-NH2 (1h)

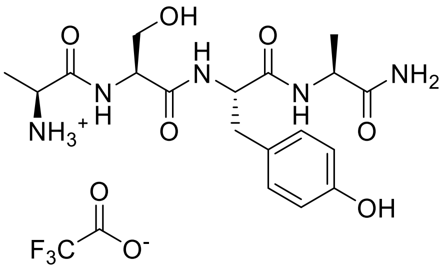

^{1}H NMR (400 MHz, MeOD) δ 7.12 – 7.04 (m, 2H, Ar*H*), 6.74 – 6.66 (m, 2H, Ar*H*), 4.55 (dd, *J* = 8.4, 5.4 Hz, 1H, C*H*-Tyr), 4.47 (t, *J* = 6.1 Hz, 1H, C*H*-Ser), 4.30 (q, *J* = 7.2 Hz, 1H, C*H*-Ala-C-Term), 3.94 (q, *J* = 7.0 Hz, 1H, C*H*-Ala-N-Term), 3.80 (dd, *J* = 10.8, 5.9 Hz, 1H, C*H2*-Ser), 3.73 (dd, *J* = 10.8, 6.3 Hz, 1H, C*H2*-Ser), 3.09 (dd, *J* = 14.2, 5.4 Hz, 1H, C*H2*-Tyr), 2.88 (dd, *J* = 14.2, 8.4 Hz, 1H, C*H2*-Tyr), 1.46 (d, *J* = 7.0 Hz, 3H, C*H3*), 1.33 (d, *J* = 7.2 Hz, 3H, C*H3*). **^{13}C NMR** (101 MHz, MeOD) δ 177.3, 173.1, 172.1, 171.1, 157.4, 131.4, 128.7, 116.3, 63.0, 56.5, 56.4, 50.2, 50.1, 37.6, 18.2, 17.6.[21] **HRMS** (ESI/QTOF) m/z: $[M + Na]^+$ Calcd for $C_{18}H_{27}N_5NaO_6^+$ 432.1854; Found 432.1855.

AMYA-NH_2 (1i)

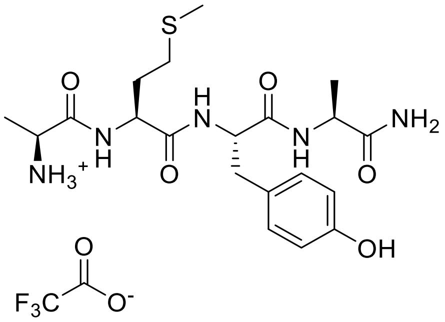

^{1}H NMR (400 MHz, MeOD) δ 7.11 – 7.03 (m, 2H, Ar*H*), 6.73 – 6.65 (m, 2H, Ar*H*), 4.60 – 4.51 (m, 1H, C*H*-Tyr), 4.46 (dd, *J* = 8.6, 5.6 Hz, 1H, C*H*-Met), 4.32 (qd, *J* = 7.2, 5.2 Hz, 1H, C*H*-Ala-C-Term), 3.92 (q, *J* = 7.0 Hz, 1H, C*H*-Ala-N-Term), 3.06 (dd, *J* = 14.0, 6.0 Hz, 1H, C*H₂*-Tyr), 2.92 – 2.82 (m, 1H, CH_2-Tyr), 2.57 – 2.39 (m, 2H, $CH_2CH_2SCH_3$), 2.07 – 1.96 (m, 1H, $CH_2CH_2SCH_3$), 2.07 (s, 3H, SCH_3), 1.88 (dtd, *J* = 14.2, 8.7, 5.8 Hz, 1H, $CH_2CH_2SCH_3$), 1.46 (d, *J* = 7.1 Hz, 3H, CH_3), 1.34 (d, *J* = 7.2 Hz, 3H, CH_3). **^{13}C NMR** (101 MHz, MeOD) δ 177.2, 173.1, 173.0, 171.0, 157.4, 131.4, 128.7, 116.3, 56.1, 54.1, 50.2, 50.1, 37.9, 32.8, 30.9, 18.3, 17.6, 15.2.[21] **HRMS** (ESI/QTOF) m/z: $[M + H]^+$ Calcd for $C_{20}H_{32}N_5O_5S^+$ 454.2119; Found 454.2117.

ADYA-NH_2 (1j)

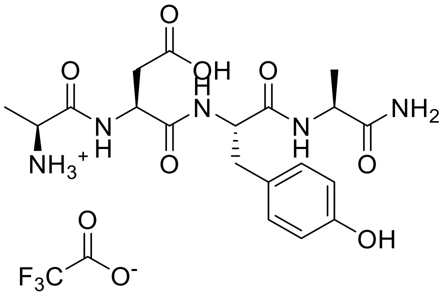

^{1}H NMR (400 MHz, MeOD) δ 7.12 – 7.03 (m, 2H, Ar*H*), 6.74 – 6.66 (m, 2H, Ar*H*), 4.71 (t, *J* = 7.0 Hz, 1H, C*H*-Asp), 4.50 (dd, *J* = 8.8, 5.2 Hz, 1H, C*H*-Tyr), 4.31 (q, *J* = 7.2 Hz, 1H, C*H*-Ala-C-Term), 3.86 (q, *J* = 7.0 Hz, 1H, C*H*-Ala-N-Term), 3.16 – 3.06 (m, 1H, CH_2-Tyr), 2.93 – 2.80 (m, 2H, CH_2-Tyr, CH_2-Asp), 2.69 (dd, *J* = 17.0, 6.9 Hz, 1H, CH_2-Asp), 1.39 (d, *J* = 7.1 Hz, 3H, CH_3), 1.36 (d, *J* = 7.2 Hz, 3H, CH_3). **^{13}C NMR** (101 MHz, MeOD) δ 176.0, 172.6, 171.6, 171.2, 169.4, 156.0, 129.9, 127.5, 114.9, 55.2, 49.8, 48.9, 48.7, 36.0, 35.2, 16.5, 16.2.[21] **HRMS** (ESI/QTOF) m/z: $[M + H]^+$ Calcd for $C_{19}H_{28}N_5O_7^+$ 438.1983; Found 438.1980.

ANYA-NH$_2$ (1k)

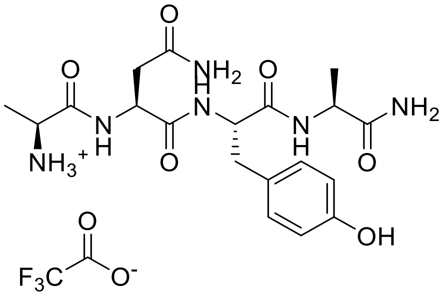

^{1}H NMR (400 MHz, MeOD) δ 7.14 – 7.04 (m, 2H, Ar*H*), 6.73 – 6.66 (m, 2H, Ar*H*), 4.72 (t, *J* = 6.9 Hz, 1H, C*H*-Asn), 4.48 (dd, *J* = 9.0, 5.0 Hz, 1H, C*H*-Tyr), 4.31 (q, *J* = 7.2 Hz, 1H, C*H*-Ala-C-Term), 3.87 (q, *J* = 7.0 Hz, 1H, C*H*-Ala-N-Term), 3.16 – 3.07 (m, 1H, CH_2-Tyr), 2.90 – 2.75 (m, 2H, CH_2-Tyr, CH_2-Asn), 2.68 – 2.58 (m, 1H, CH_2-Asn), 1.37 (d, *J* = 7.2 Hz, 3H, CH_3) , 1.38 (d, *J* = 7.1 Hz, 3H, CH_3). **^{13}C NMR** (101 MHz, MeOD) δ 176.2, 173.1, 171.8, 171.6, 169.4, 155.9, 129.9, 127.5, 114.9, 55.3, 50.0, 49.0, 48.7, 36.2, 36.0, 16.6, 16.2.[21] **HRMS** (nanochip-ESI/LTQ-Orbitrap) m/z: [M + H]$^+$ Calcd for $C_{19}H_{29}N_6O_6^+$ 437.2143; Found 437.2156.

AYLA-NH$_2$ (1l)

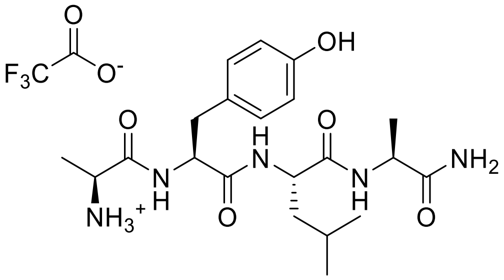

^{1}H NMR (400 MHz, MeOD) δ 7.13 – 7.05 (m, 2H, Ar*H*), 6.73 – 6.65 (m, 2H, Ar*H*), 4.61 (dd, *J* = 9.3, 5.5 Hz, 1H, C*H*-Tyr), 4.38 (dd, *J* = 8.0, 6.9 Hz, 1H, C*H*-Leu), 4.32 (q, *J* = 7.1 Hz, 1H, C*H*-Ala-C-Term), 3.85 (q, *J* = 7.0 Hz, 1H, C*H*-Ala-N-Term), 3.09 (dd, *J* = 14.1, 5.5 Hz, 1H, CH_2-Tyr), 2.83 (dd, *J* = 14.1, 9.3 Hz, 1H, CH_2-Tyr), 1.73 – 1.62 (m, 1H, CH_2CH(CH$_3$)$_2$), 1.62 – 1.55 (m, 2H, CH_2CH(CH$_3$)$_2$, CH$_2$C*H*(CH$_3$)$_2$), 1.47 (d, *J* = 7.1 Hz, 3H, CH_3), 1.36 (d, *J* = 7.1 Hz, 3H, CH_3), 0.95 (d, *J* = 6.3 Hz, 3H, CH$_2$CH(CH_3)$_2$), 0.91 (d, *J* = 6.3 Hz, 3H, CH$_2$CH(CH_3)$_2$). **^{13}C NMR** (101 MHz, MeOD) δ 175.9, 172.6, 172.0, 169.5, 156.0, 129.9, 127.38, 114.9, 55.0, 51.7, 48.6, 48.6, 40.4, 36.4, 24.3, 22.1, 20.5, 17.0, 16.2.[21] **HRMS** (nanochip-ESI/LTQ-Orbitrap) m/z: [M + H]$^+$ Calcd for $C_{21}H_{34}N_5O_5^+$ 436.2554; Found 436.2561.

YALA-NH$_2$ (1m)

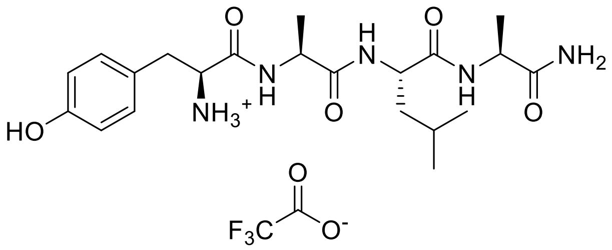

^{1}H NMR (400 MHz, MeOD) δ 7.16 – 7.08 (m, 2H, Ar*H*), 6.81 – 6.73 (m, 2H, Ar*H*), 4.48 – 4.37 (m, 2H, C*H*-Ala, C*H*-Leu), 4.34 (q, *J* = 7.1 Hz, 1H, C*H*-Ala-C-Term), 4.04 (dd, *J* = 8.6, 5.1 Hz, 1H, C*H*-Tyr), 3.19 (dd, *J* = 14.5, 5.2 Hz, 1H, C*H*$_2$-Tyr), 2.91 (dd, *J* = 14.5, 8.7 Hz, 1H, C*H*$_2$-Tyr), 1.81 – 1.67 (m, 1H, C*H*$_2$CH(CH$_3$)$_2$), 1.67 – 1.56 (m, 2H, CH$_2$C*H*(CH$_3$)$_2$, C*H*$_2$CH(CH$_3$)$_2$), 1.40 (d, *J* = 7.1 Hz, 3H, C*H*$_3$), 1.37 (d, *J* = 7.1 Hz, 3H, C*H*$_3$), 0.98 (d, *J* = 6.5 Hz, 3H, CH$_2$CH(C*H*$_3$)$_2$), 0.94 (d, *J* = 6.4 Hz, 3H, CH$_2$CH(C*H*$_3$)$_2$). **^{13}C NMR** (101 MHz, MeOD) δ 177.3, 174.5, 174.2, 169.5, 158.3, 131.6, 125.9, 116.9, 55.7, 53.2, 50.4, 49.9, 41.7, 37.8, 25.9, 23.5, 22.0, 18.5, 18.2.[21] **HRMS** (nanochip-ESI/LTQ-Orbitrap) m/z: $[M + H]^+$ Calcd for $C_{21}H_{34}N_5O_5^+$ 436.2554; Found 436.2560.

Ac-AFGY (1n)

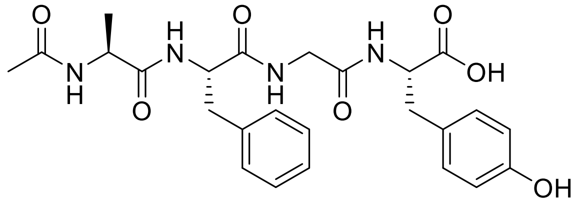

^{1}H NMR (400 MHz, MeOD) δ 7.31 – 7.15 (m, 5H, Ar*H*), 7.09 – 7.01 (m, 2H, Ar*H*), 6.74 – 6.65 (m, 2H, Ar*H*), 4.59 (dd, *J* = 8.4, 5.1 Hz, 1H, C*H*-Phe), 4.50 (dd, *J* = 9.4, 5.5 Hz, 1H, C*H*-Tyr), 4.19 (q, *J* = 7.2 Hz, 1H, C*H*-Ala-N-Term), 3.97 (d, *J* = 16.8 Hz, 1H, C*H*$_2$-Gly), 3.66 (d, *J* = 16.8 Hz, 1H, C*H*$_2$-Gly), 3.23 (dd, *J* = 14.0, 5.5 Hz, 1H, C*H*$_2$-Tyr), 3.15 – 3.05 (m, 1H, C*H*$_2$-Phe), 2.96 (ddd, *J* = 14.0, 11.5, 8.9 Hz, 2H, C*H*$_2$-Tyr, C*H*$_2$-Phe), 1.94 (s, 3H, Ac-C*H*$_3$), 1.19 (d, *J* = 7.2 Hz, 3H, C*H*$_3$). **^{13}C NMR** (101 MHz, MeOD) δ 173.8, 173.1, 172.4, 172.4, 169.9, 155.9, 137.2, 130.0, 128.9, 128.1, 127.6, 126.3, 114.8, 54.9, 54.1, 49.5, 41.8, 36.5, 36.2, 21.0, 16.1. **HRMS** (ESI/QTOF) m/z: $[M + H_{-1}]^-$ Calcd for $C_{25}H_{29}N_4O_7^-$ 497.2042; Found 497.2053.

5. Scope of tetramers

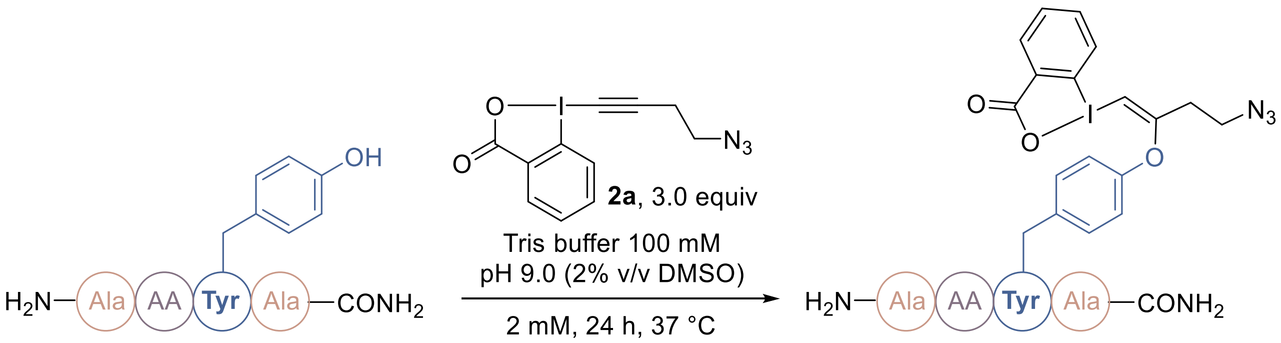

General procedure:

To a solution of tetramers **1** (20.0 µmol, 1.00 equiv) in 100 mM Tris buffer pH 9.0 (9.80 mL), in a 25 mL round bottom flask, was added a 300 mM solution of N_3-EBX **2a** in DMSO (200 µL, 20.5 mg, 60.0 µmol, 3.00 equiv). The 2.00 mM solution was stirred at 37 °C for 24 h. No effort was made to exclude oxygen. The reaction was analyzed by HPLC-MS. The crude material was lyophilized and purified by reverse phase HPLC (water 0.1 % TFA to 95:5 ACN:water 0.1 % TFA).

Abbreviations for HPLC:

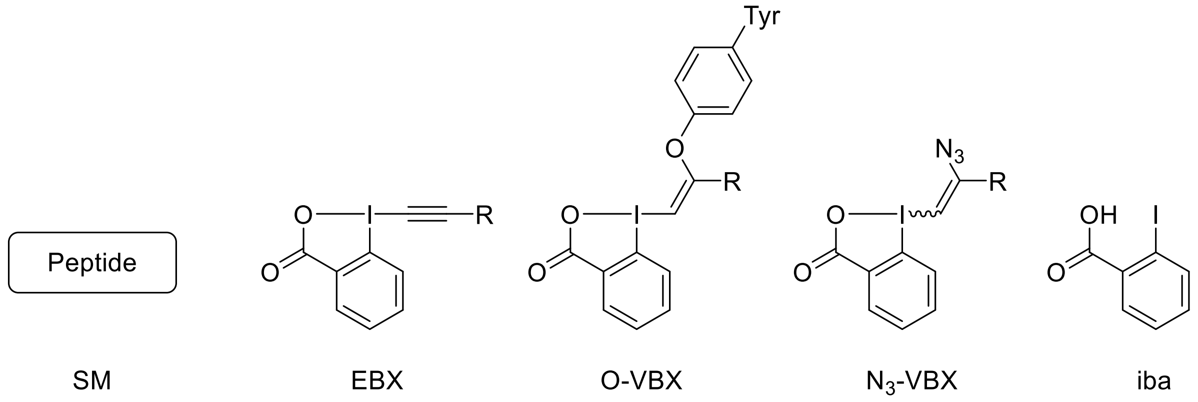

O-VBX of AFYA-NH$_2$ (3a)

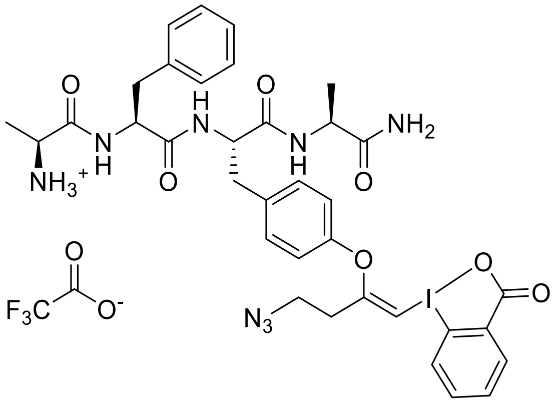

Starting from AFYA-NH$_2$ (**1a**) (11.7 mg, 20.0 µmol), O-VBX of AFYA-NH$_2$ (**3a**) (11.8 mg, 12.8 µmol, 64% yield) was obtained, as a white solid after lyophilization (retention time 8.6 min).

^{1}H NMR (400 MHz, MeOD) δ 8.32 – 8.25 (m, 1H, Ar*H*), 8.03 – 7.96 (m, 1H, Ar*H*), 7.83 – 7.69 (m, 2H, Ar*H*), 7.30 – 7.14 (m, 7H, Ar*H*), 7.00 – 6.92 (m, 2H, Ar*H*), 6.49 (d, *J* = 0.7 Hz, 1H, C*H*-I), 4.56 (dd, *J* = 9.4, 5.4 Hz, 1H, C*H*-Phe), 4.51 (dd, *J* = 8.5, 5.7 Hz, 1H, C*H*-Tyr), 4.30 (q, *J* = 7.1 Hz, 1H, C*H*-Ala-C-Term), 3.81 (q, *J* = 7.0 Hz, 1H, C*H*-Ala-N-Term), 3.59 (t, *J* = 6.3 Hz, 2H, $CH_2CH_2N_3$), 3.19 – 3.04 (m, 2H, CH_2-Tyr, CH_2-Phe), 2.96 – 2.81 (m, 4H, CH_2-Tyr, CH_2-Phe, $CH_2CH_2N_3$), 1.36 (d, *J* = 3.8 Hz, 3H, CH_3), 1.35 (d, *J* = 3.9 Hz, 3H, CH_3). **^{13}C NMR** (101 MHz, MeOD) δ 177.1, 172.9, 172.3, 171.0, 170.4, 168.3, 153.9, 138.2, 135.9, 135.9, 133.6, 133.3, 132.4, 132.0, 130.2, 129.5, 129.1, 127.9, 120.4, 114.5, 82.5, 56.3, 55.9, 50.0, 38.6, 38.0, 33.0, 18.5, 17.7 *2 aliphatic carbon signals are not resolved (under the solvent peak)*.[21] **HRMS** (nanochip-ESI/LTQ-Orbitrap) m/z: $[M + H]^+$ Calcd for $C_{35}H_{40}IN_8O_7^+$ 811.2059; Found 811.2054.

HPLC-UV chromatogram (214 nm) of AFYA-NH$_2$ (**1a**):

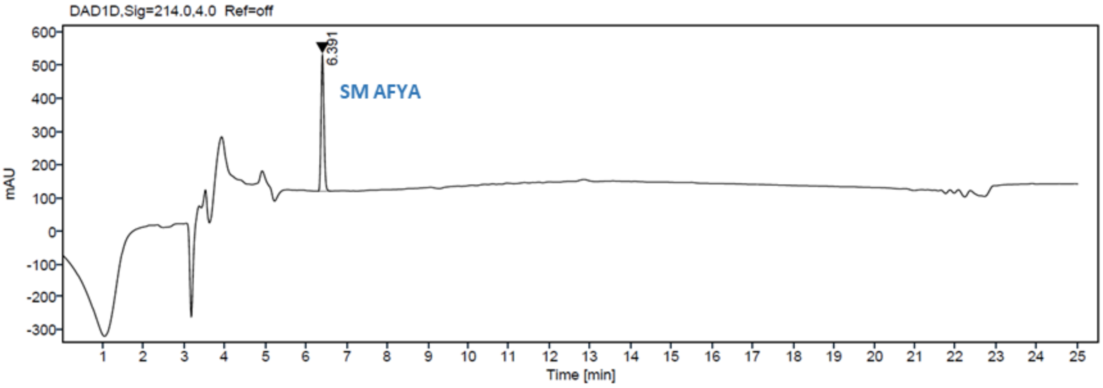

HPLC-UV chromatogram (214 nm) of crude of the reaction:

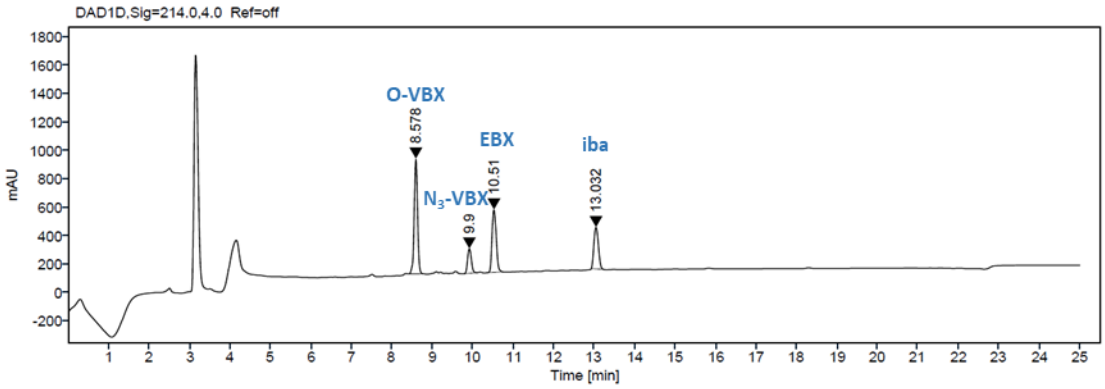

HPLC-MS chromatogram of crude of the reaction:

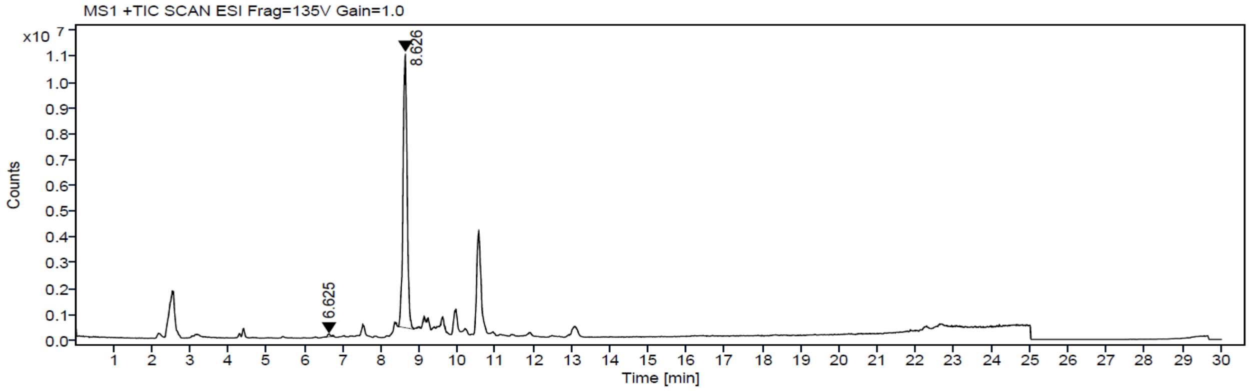

HPLC-UV chromatogram (214 nm) of O-VBX of AFYA-NH_2 (**3a**):

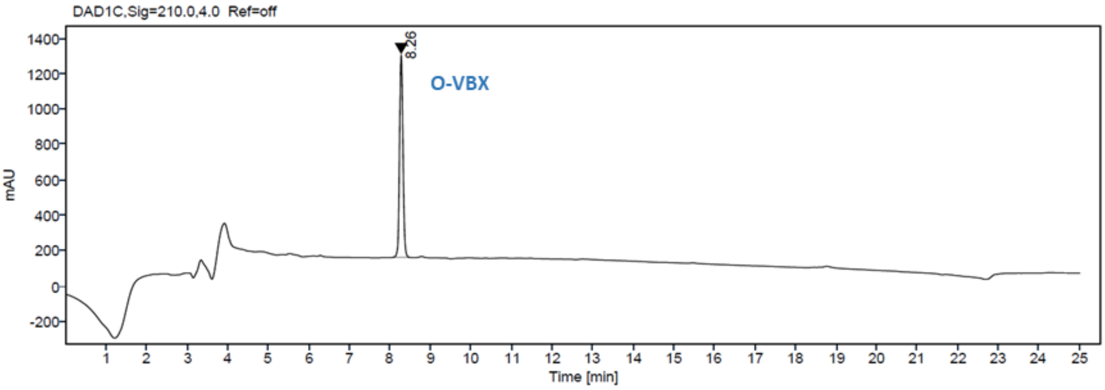

Relative ratio based on reverse phase HPLC-UV chromatogram at 214 nm: > 99%

Relative ratio based on reverse phase HPLC-MS chromatogram: > 99%

O-VBX of ALYA-NH_2 (3b)

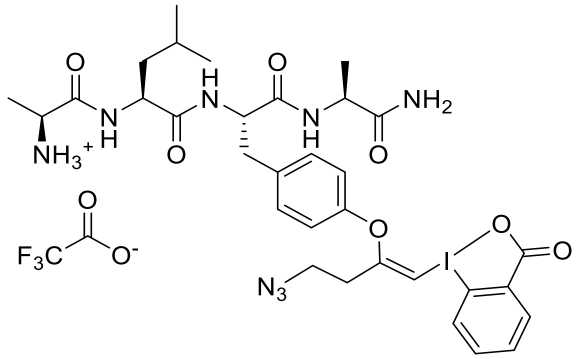

Starting from ALYA-NH_2 (**1b**) (11.0 mg, 20.0 µmol), O-VBX of ALYA-NH_2 (**3b**) (11.8 mg, 13.0 µmol, 65% yield) was obtained, as a white solid after lyophilization (retention time 8.3 min).

^{1}H NMR (400 MHz, MeOD) δ 8.32 – 8.25 (m, 1H, Ar*H*), 8.03 – 7.97 (m, 1H, Ar*H*), 7.83 – 7.70 (m, 2H, Ar*H*), 7.30 – 7.21 (m, 2H, Ar*H*), 7.00 – 6.91 (m, 2H, Ar*H*), 6.50 (d, *J* = 0.8 Hz, 1H, C*H*-I), 4.56 (dd, *J* = 8.6, 5.7 Hz, 1H, C*H*-Tyr), 4.39 – 4.27 (m, 2H, C*H*-Leu, C*H*-Ala-C-Term), 3.89 (q, *J* = 7.0 Hz, 1H, C*H*-Ala-N-Term), 3.61 (t, *J* = 6.3 Hz, 2H, $CH_2CH_2N_3$), 3.21 – 3.11 (m, 1H, CH_2-Tyr), 2.99 – 2.87 (m, 3H, CH_2-Tyr, $CH_2CH_2N_3$), 1.58 (m, 1H, $CH_2CH(CH_3)_2$), 1.48 (m, 2H, $CH_2CH(CH_3)_2$), 1.39 (d, *J* = 7.0 Hz, 3H, CH_3), 1.35 (d, *J* = 7.1 Hz, 3H, CH_3), 0.88 (dd, *J* = 11.7, 6.5 Hz, 6H, $CH_2CH(CH_3)_2$). **^{13}C NMR** (101 MHz, MeOD) δ 177.0, 174.1, 172.5, 170.9, 170.3, 168.2, 154.0, 135.8, 135.7, 133.7, 133.6, 132.4, 132.0, 129.1, 120.3, 114.5, 83.1, 55.5, 53.4, 50.0, 49.9, 49.5, 41.9, 37.9, 32.9, 25.8, 23.3, 21.9, 18.5, 17.7.[21] **HRMS** (ESI/QTOF) m/z: [M + H]$^+$ Calcd for $C_{32}H_{42}IN_8O_7^+$ 777.2216; Found 777.2218.

HPLC-UV chromatogram (214 nm) of ALYA-NH_2 (**1b**):

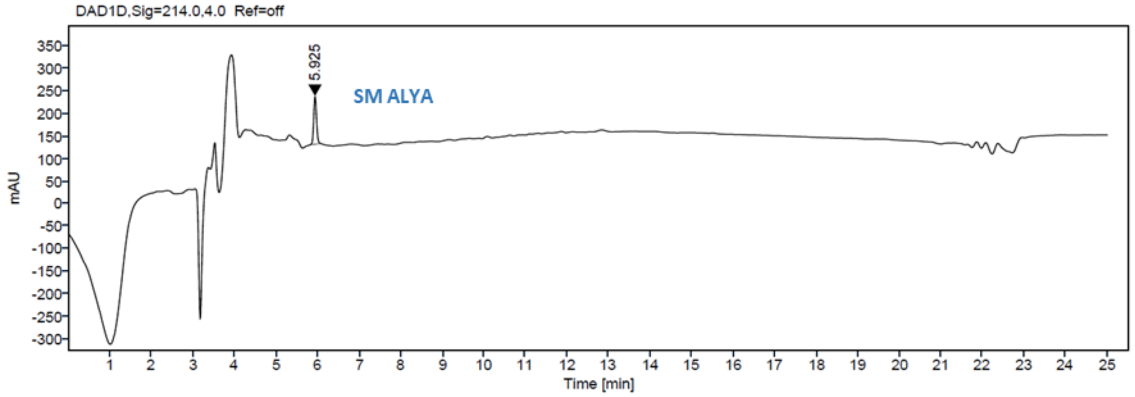

HPLC-UV chromatogram (214 nm) of crude of the reaction:

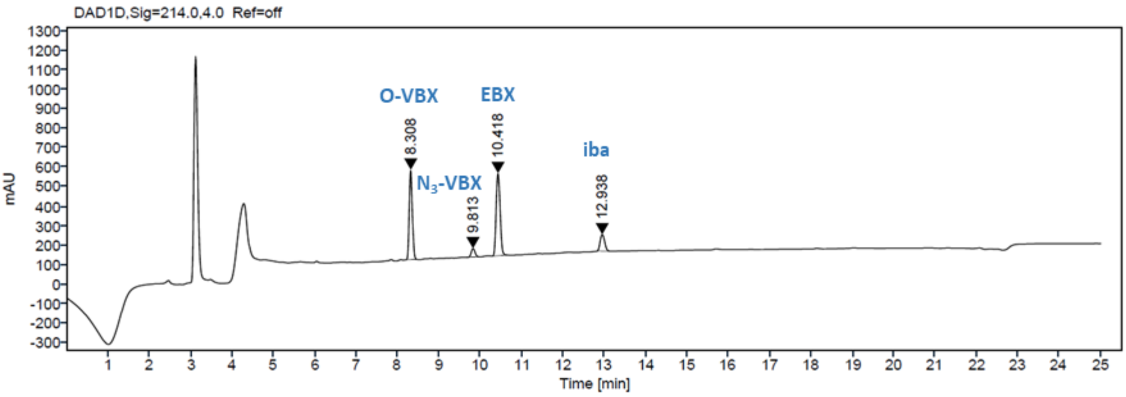

HPLC-MS chromatogram of crude of the reaction:

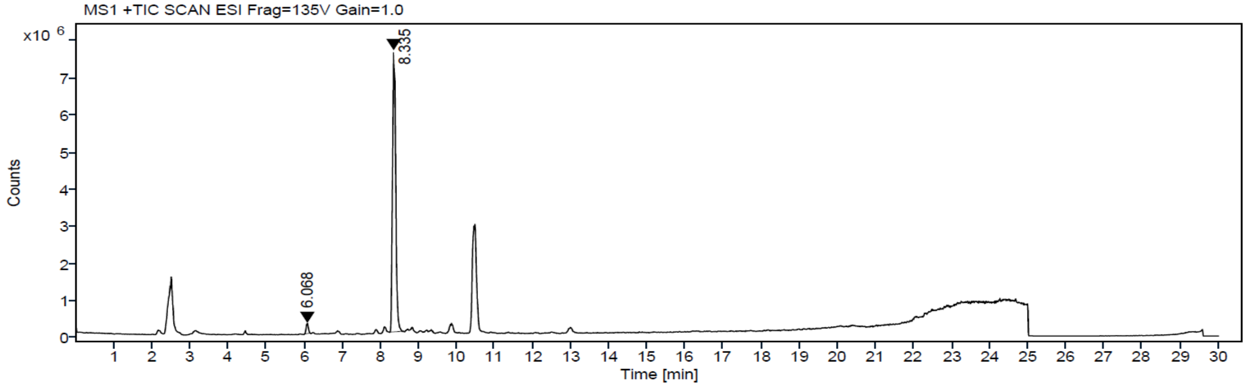

HPLC-UV chromatogram (214 nm) of O-VBX of ALYA-NH_2 (**3b**):

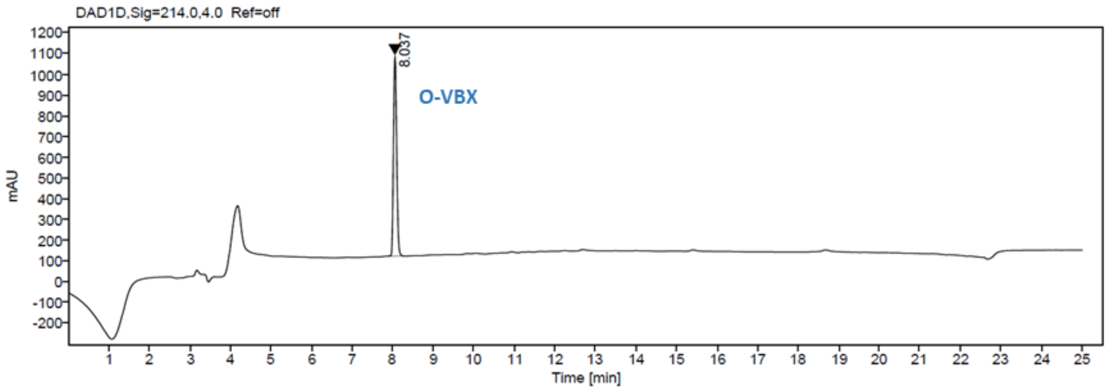

Relative ratio based on reverse phase HPLC-UV chromatogram at 214 nm: > 95%

Relative ratio based on reverse phase HPLC-MS chromatogram: > 95%

O-VBX of AWYA-NH$_2$ (3c)

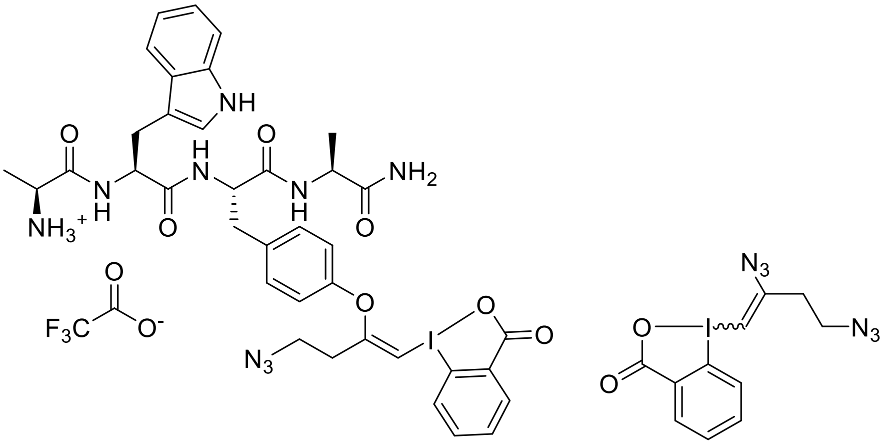

Starting from AWYA-NH$_2$ (**1c**) (12.5 mg, 20.0 µmol), O-VBX of AWYA-NH$_2$ (**3c**) in a 1 : 2 mixture with N$_3$-VBX (**4**) (20.0 mg of the mixture, 11.6 µmol, 58% yield) was obtained, as a white solid after lyophilization (retention time 9.0 min).

^{1}H NMR (400 MHz, MeOD) δ 8.28 (dd, J = 7.4, 1.8 Hz, 1H, ArH), 7.98 (dd, J = 8.0, 1.1 Hz, 1H, ArH), 7.82 – 7.69 (m, 2H, ArH), 7.57 (dt, J = 7.9, 1.0 Hz, 1H, ArH), 7.32 (dt, J = 8.2, 1.0 Hz, 1H, ArH), 7.18 – 7.14 (m, 2H, ArH), 7.12 – 7.05 (m, 2H, ArH), 6.99 (ddd, J = 7.9, 7.0, 1.0 Hz, 1H), 6.95 – 6.87 (m, 2H, ArH), 6.47 (s, 1H, CH-I), 4.62 (dd, J = 8.3, 5.9 Hz, 1H, CH-Tyr), 4.49 (dd, J = 8.4, 5.6 Hz, 1H, CH-Trp), 4.28 (q, J = 7.1 Hz, 1H, CH-Ala-C-Term), 3.86 (m, 1H, CH-Ala-N-Term), 3.54 (t, J = 6.3 Hz, 2H, CH$_2$CH_2N$_3$), 3.24 – 3.18 (m, 1H, CH_2-Trp), 3.13 – 3.02 (m, 2H, CH_2-Tyr, CH_2-Trp), 2.92 – 2.83 (m, 3H, CH_2CH$_2$N$_3$, CH_2-Tyr), 1.34 (dd, J = 12.9, 7.1 Hz, 6H, CH_3). **^{13}C NMR** (101 MHz, MeOD) δ 177.1, 173.3, 172.3, 171.0, 170.4, 168.3, 153.9, 138.1, 136.0, 135.9, 133.7, 133.1, 132.4, 132.2, 132.0, 129.2, 128.6, 124.7, 122.6, 120.4, 119.9, 119.3, 114.5, 112.4, 110.7, 82.2, 55.8, 55.8, 50.0, 49.7, 49.5, 37.8, 32.9, 28.9, 18.4, 17.7. [21] **HRMS** (ESI/QTOF) m/z: [M + H]$^+$ Calcd for $C_{37}H_{41}IN_9O_7^+$ 850.2168; Found 850.2149.

HPLC-UV chromatogram (214 nm) of AWYA-NH$_2$ (**1c**):

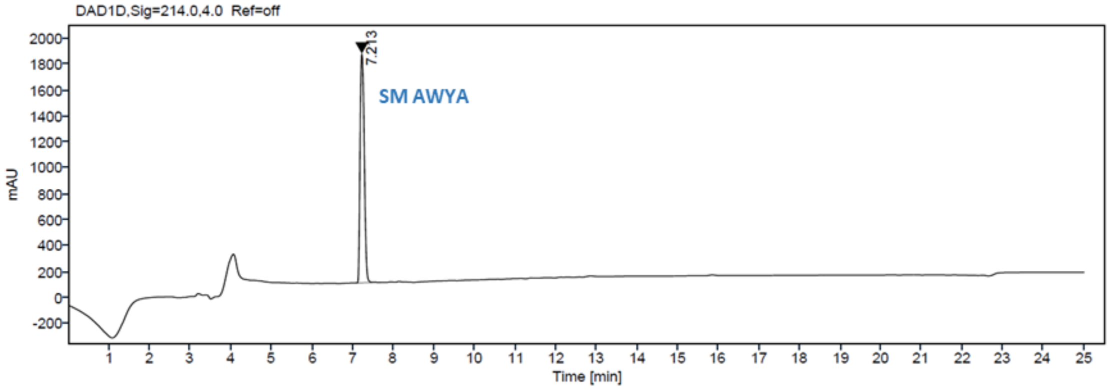

HPLC-UV chromatogram (214 nm) of crude of the reaction:

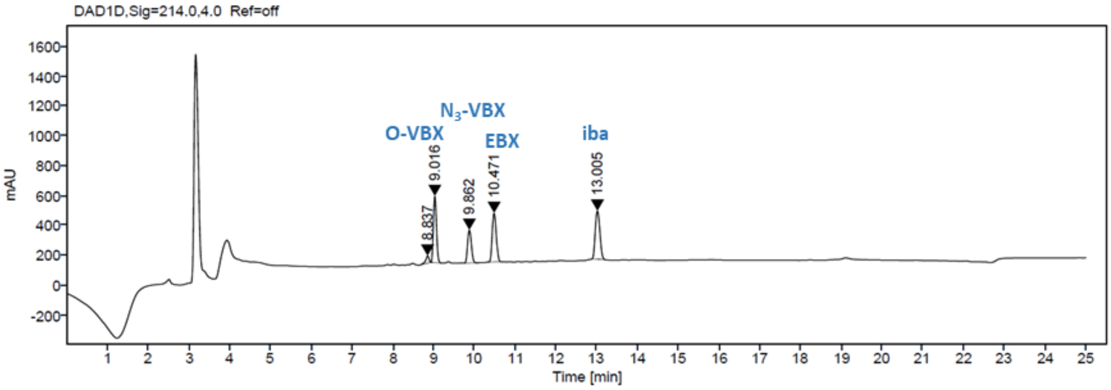

HPLC-MS chromatogram of crude of the reaction:

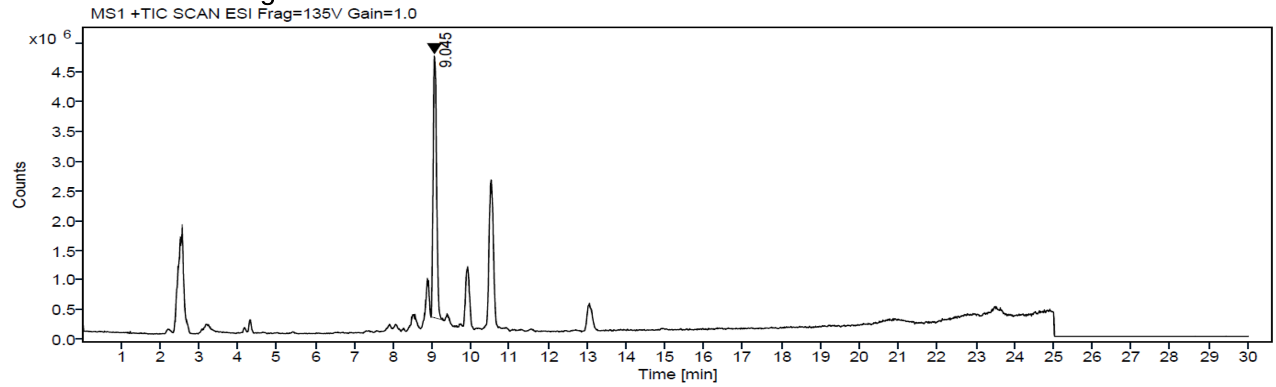

HPLC-UV chromatogram (214 nm) of O-VBX of AWYA-NH_2 (**3c**):

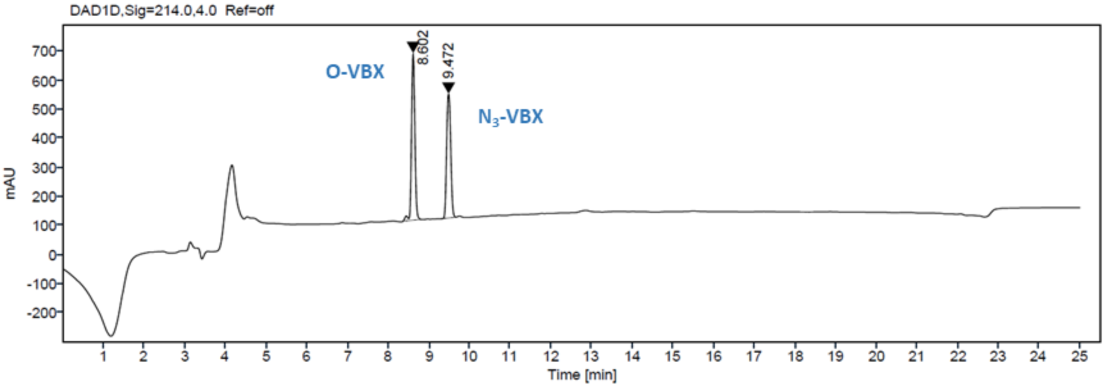

Relative ratio based on reverse phase HPLC-UV chromatogram at 214 nm: > 99%

Relative ratio based on reverse phase HPLC-MS chromatogram: > 99%

N_3-VBX (**4**)

^{1}H NMR (400 MHz, DMSO) δ 8.22 – 8.11 (m, 1H), 7.74 – 7.65 (m, 3H), 6.37 (d, J = 1.1 Hz, 1H), 3.83 (t, J = 6.5 Hz, 2H), 3.18 – 3.10 (m, 2H). **^{13}C NMR** (101 MHz, DMSO) δ 166.6, 153.2, 140.5, 134.2, 131.9, 130.5, 127.9, 114.1, 85.3, 48.2, 32.0. **HRMS** (nanochip-ESI/LTQ-Orbitrap) m/z: $[M + H]^+$ Calcd for $C_{11}H_{10}IN_6O_2^+$ 384.9904; Found 384.9902.

O-VBX of ARYA-NH$_2$ (3d)

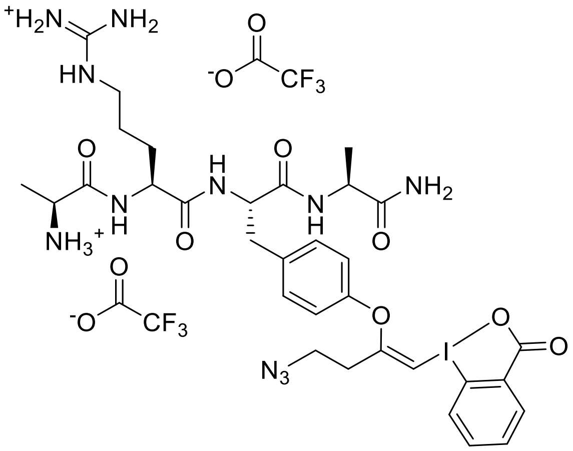

Starting from ARYA-NH$_2$ (**1d**) (14.1 mg, 20.0 μmol), O-VBX of ARYA-NH$_2$ (**3d**) (14.9 mg, 14.2 μmol, 71% yield) was obtained, as a white solid after lyophilization (retention time 6.6 min).

^{1}H NMR (400 MHz, MeOD) δ 8.31 – 8.25 (m, 1H, Ar*H*), 8.02 – 7.95 (m, 1H, Ar*H*), 7.82 – 7.69 (m, 2H, Ar*H*), 7.30 – 7.22 (m, 2H, Ar*H*), 7.00 – 6.92 (m, 2H, Ar*H*), 6.50 (s, 1H, C*H*-I), 4.59 (dd, *J* = 8.6, 5.4 Hz, 1H, C*H*-Tyr), 4.36 – 4.25 (m, 2H, C*H*-Arg, C*H*-Ala-C-Term), 4.00 – 3.88 (m, 1H, C*H*-Ala-N-Term), 3.62 (t, *J* = 6.3 Hz, 2H, $CH_2CH_2N_3$), 3.17 (td, *J* = 7.8, 6.9, 3.2 Hz, 3H, $CH_2CH_2CH_2NH$, CH_2-Tyr), 3.00 – 2.87 (m, 3H, CH_2-Tyr, $CH_2CH_2N_3$), 1.80 (m, 1H, $CH_2CH_2CH_2NH$), 1.74 – 1.53 (m, 3H, $CH_2CH_2CH_2NH$), 1.40 (d, *J* = 7.1 Hz, 3H, CH_3), 1.35 (d, *J* = 7.1 Hz, 3H, CH_3). **^{13}C NMR** (101 MHz, MeOD) δ 177.1, 173.1, 172.6, 171.0, 170.4, 168.1, 158.6, 154.1, 135.7, 135.6, 133.9, 133.5, 132.4, 132.0, 129.0, 120.3, 114.7, 82.9, 55.6, 54.2, 50.1, 49.7, 49.5, 41.8, 38.0, 33.2, 30.2, 26.1, 18.5, 17.7.[21] **HRMS** (ESI/QTOF) m/z: [M + H]$^+$ Calcd for $C_{32}H_{43}IN_{11}O_7^+$ 820.2386; Found 820.2390.

HPLC-UV chromatogram of ARYA-NH$_2$ (**1d**):

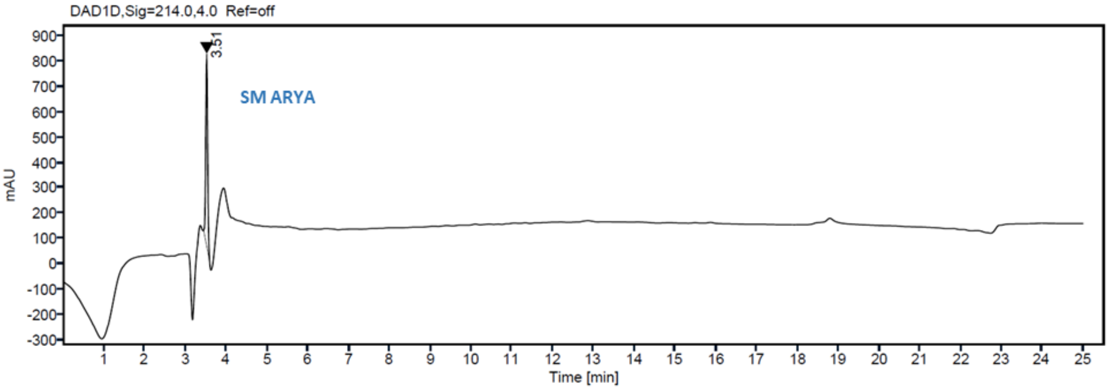

HPLC-MS chromatogram of ARYA-NH_2 (**1d**):

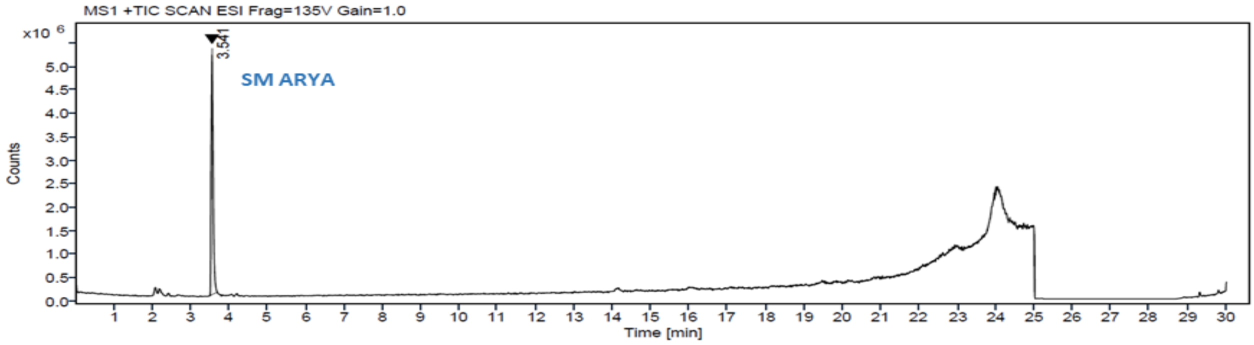

HPLC-UV chromatogram (214 nm) of crude of the reaction:

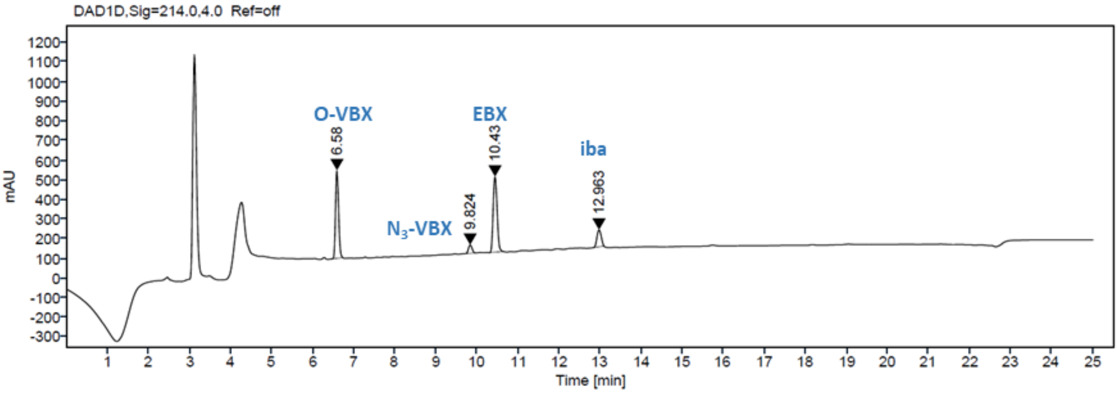

HPLC-MS chromatogram of crude of the reaction:

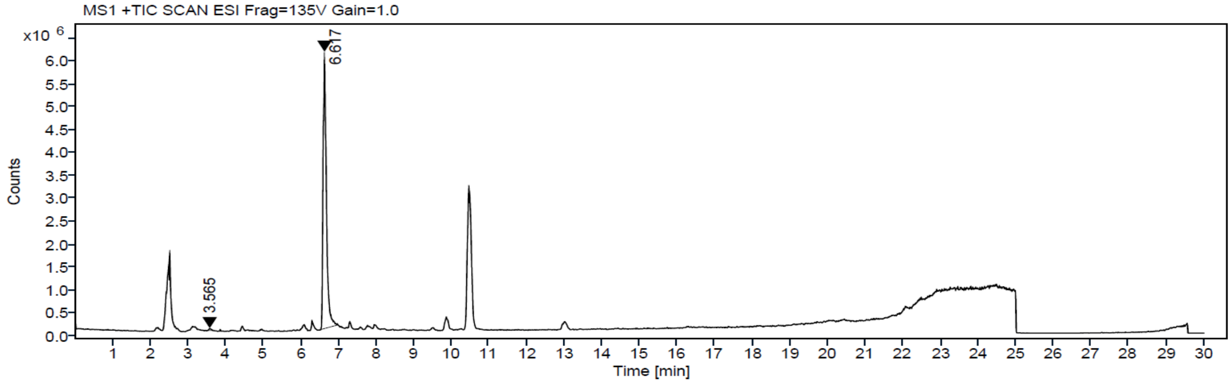

HPLC-UV chromatogram (214 nm) of O-VBX of ARYA-NH_2 (**3d**):

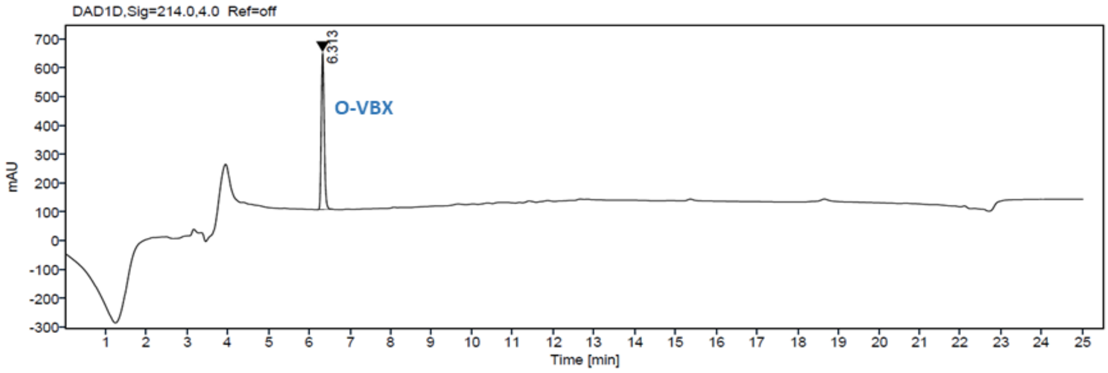

Relative ratio based on reverse phase HPLC-UV chromatogram at 214 nm: > 99%

Relative ratio based on reverse phase HPLC-MS chromatogram: > 99%

O-VBX of AHYA-NH2 (3e)

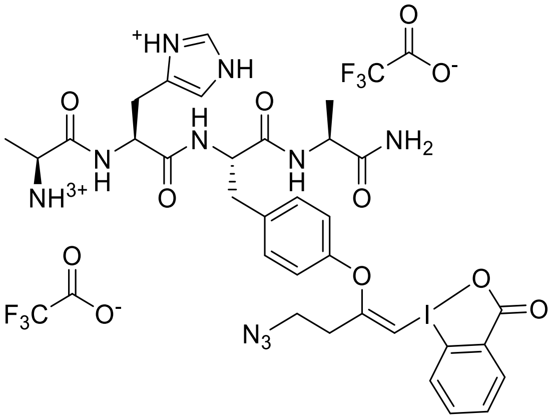

Starting from AHYA-NH_2 (**1e**) (13.7 mg, 20.0 μmol), O-VBX of AHYA-NH_2 (**3e**) (12.4 mg, 12.1 μmol, 60% yield) was obtained, as a white solid after lyophilization (retention time 6.6 min).

^{1}H NMR (400 MHz, MeOD) δ 8.79 (d, *J* = 1.4 Hz, 1H, Ar*H*), 8.28 (dd, *J* = 7.4, 1.9 Hz, 1H, Ar*H*), 7.98 (dd, *J* = 8.1, 1.1 Hz, 1H, Ar*H*), 7.85 – 7.67 (m, 2H, Ar*H*), 7.32 (d, *J* = 1.4 Hz, 1H, Ar*H*), 7.30 – 7.21 (m, 2H, Ar*H*), 7.01 – 6.93 (m, 2H, Ar*H*), 6.47 (s, 1H, C*H*-I), 4.69 (t, *J* = 6.7 Hz, 1H, C*H*-Tyr), 4.53 (dd, *J* = 8.7, 5.5 Hz, 1H, C*H*-His), 4.32 (q, *J* = 7.2 Hz, 1H, C*H*-Ala-C-Term), 3.92 (q, *J* = 7.0 Hz, 1H, C*H*-Ala-N-Term), 3.64 (t, *J* = 6.3 Hz, 2H, $CH_2CH_2N_3$), 3.27 – 3.07 (m, 3H, CH_2-Tyr, CH_2-His), 2.94 (q, *J* = 7.5, 6.5 Hz, 3H, CH_2-Tyr, $CH_2CH_2N_3$), 1.38 (m, 6H, CH_3). **^{13}C NMR** (101 MHz, MeOD) δ 177.1, 172.7, 171.4, 171.1, 170.5, 168.3, 154.2, 135.8, 135.7, 135.1, 133.6 (*2 carbon signals under this peak*), 132.4, 132.0, 130.4, 129.0, 120.6, 118.7, 114.8, 81.8, 56.0, 53.5, 50.2, 50.1, 49.3, 37.8, 33.6, 28.1, 18.4, 17.6. [21] **HRMS** (nanochip-ESI/LTQ-Orbitrap) m/z: $[M + H]^+$ Calcd for $C_{32}H_{38}IN_{10}O_7^+$ 801.1964; Found 801.1983.

HPLC-UV chromatogram of AHYA-NH_2 (**1e**):

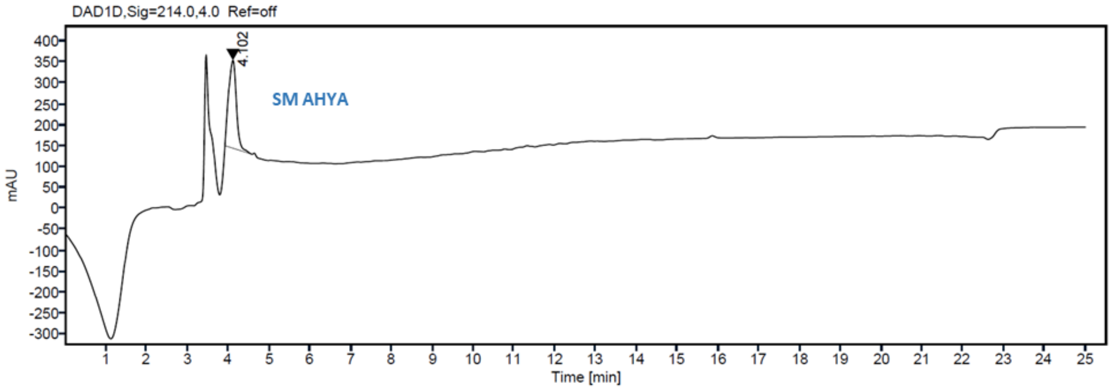

HPLC-MS chromatogram of AHYA-NH_2 (**1e**):

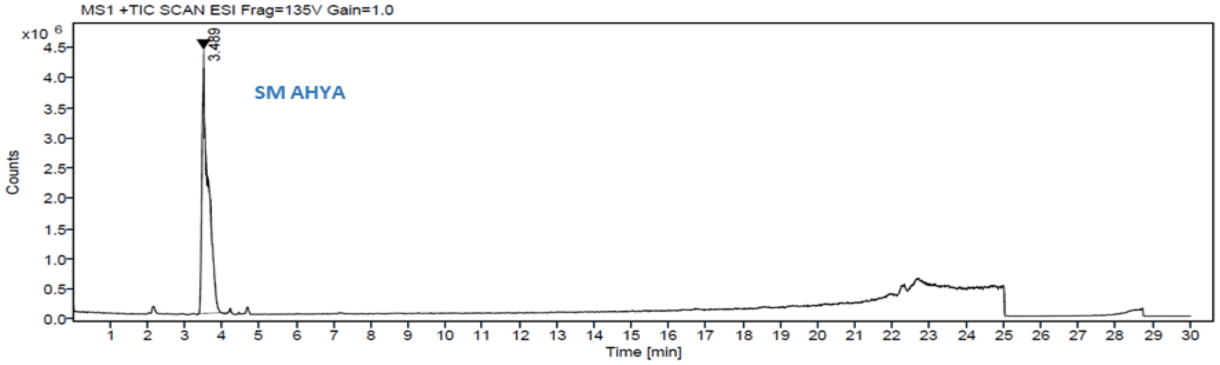

HPLC-UV chromatogram (214 nm) of crude of the reaction:

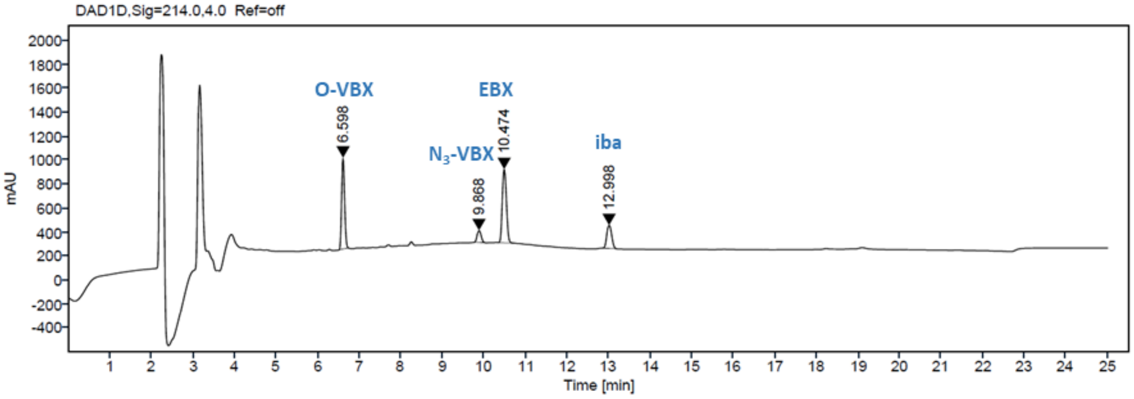

HPLC-MS chromatogram of crude of the reaction:

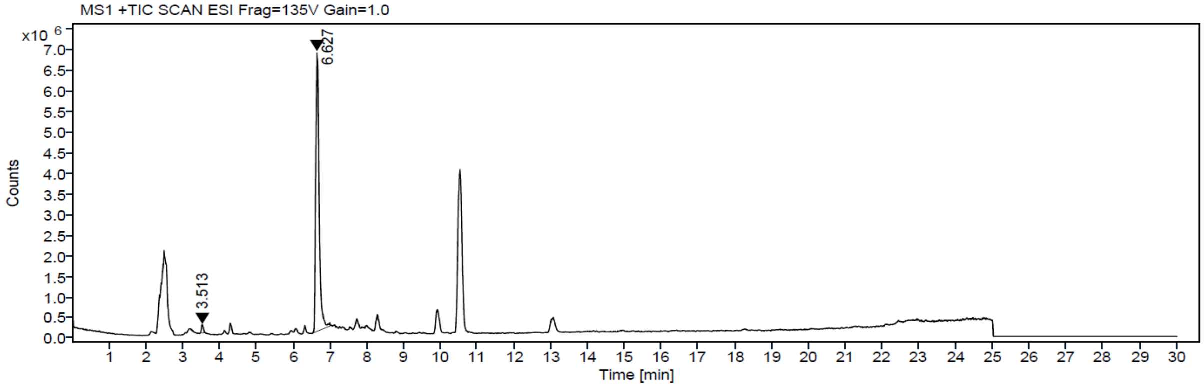

HPLC-UV chromatogram (214 nm) of O-VBX of AHYA-NH_2 (**3e**):

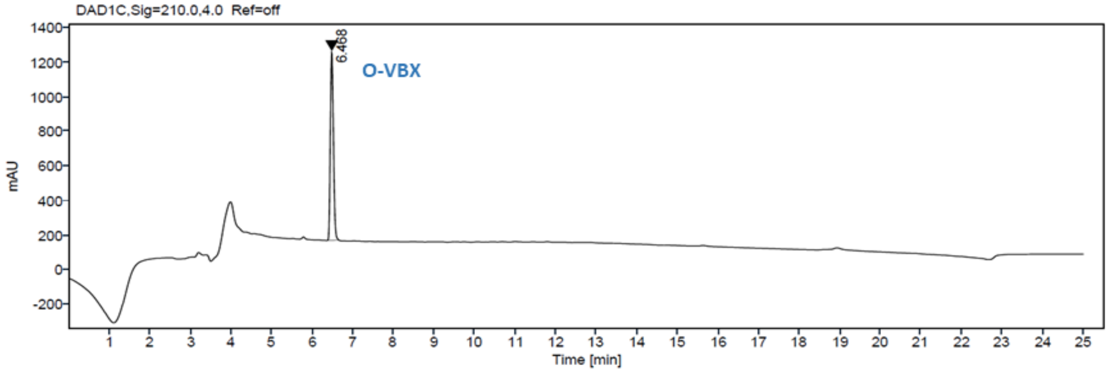

Relative ratio based on reverse phase HPLC-UV chromatogram at 214 nm: > 99%

Relative ratio based on reverse phase HPLC-MS chromatogram: > 95%

O-VBX of AKYA-NH_2 (3f)

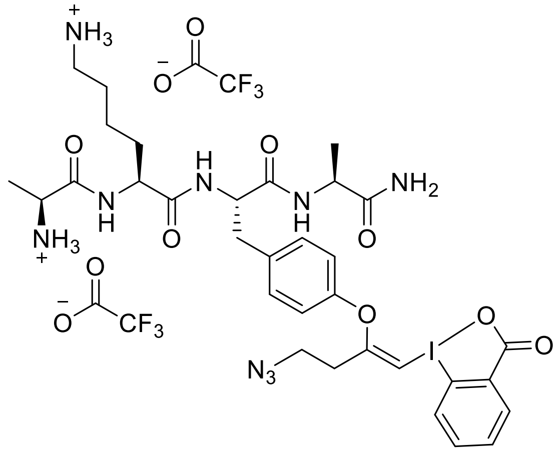

Starting from AKYA-NH_2 (**1f**) (13.6 mg, 20.0 μmol), O-VBX of AKYA-NH_2 (**3f**) (12.5 mg, 12.3 μmol, 61% yield) was obtained, as a white solid after lyophilization (retention time 6.6 min).

^{1}H NMR (400 MHz, MeOD) δ 8.32 – 8.25 (m, 1H, Ar*H*), 8.15 (dd, *J* = 10.3, 7.3 Hz, 1H, N*H*), 8.03 – 7.96 (m, 1H, Ar*H*), 7.83 – 7.70 (m, 2H, Ar*H*), 7.30 – 7.22 (m, 2H, Ar*H*), 7.00 – 6.92 (m, 2H, Ar*H*), 6.51 (d, *J* = 0.7 Hz, 1H, C*H*-I), 4.65 – 4.55 (m, 1H, C*H*-Tyr), 4.37 – 4.21 (m, 2H, C*H*-Lys, C*H*-Ala-C-Term), 3.92 (q, *J* = 7.0 Hz, 1H, C*H*-Ala-N-Term), 3.62 (t, *J* = 6.3 Hz, 2H, $CH_2CH_2N_3$), 3.24 – 3.11 (m, 1H, CH_2-Tyr), 2.92 (tt, *J* = 5.8, 4.3 Hz, 5H, CH_2-Tyr, $CH_2CH_2CH_2CH_2NH_2$, $CH_2CH_2N_3$), 1.80 – 1.54 (m, 4H, $CH_2CH_2CH_2CH_2NH_2$), 1.41 (m, 2H, $CH_2CH_2CH_2CH_2NH_2$), 1.41 (d, *J* = 7.1 Hz, 3H, C*H*$_3$), 1.35 (d, *J* = 7.2 Hz, 3H, C*H*$_3$). **^{13}C NMR** (101 MHz, MeOD) δ 177.1, 173.4, 172.7, 171.0, 170.4, 168.2, 154.0, 135.8, 135.7, 133.6 (*2 carbon signals under this peak*), 132.4, 132.0, 129.1, 120.3, 114.6, 83.0, 55.6, 54.6, 50.2, 50.1, 40.4, 38.0, 33.0, 32.5, 28.0, 23.6, 18.6, 18.5, 17.7. [21] **HRMS** (ESI/QTOF) m/z: $[M + H]^+$ Calcd for $C_{32}H_{43}IN_9O_7^+$ 792.2325; Found 792.2303.

HPLC-UV chromatogram of AKYA-NH_2 (**1f**):

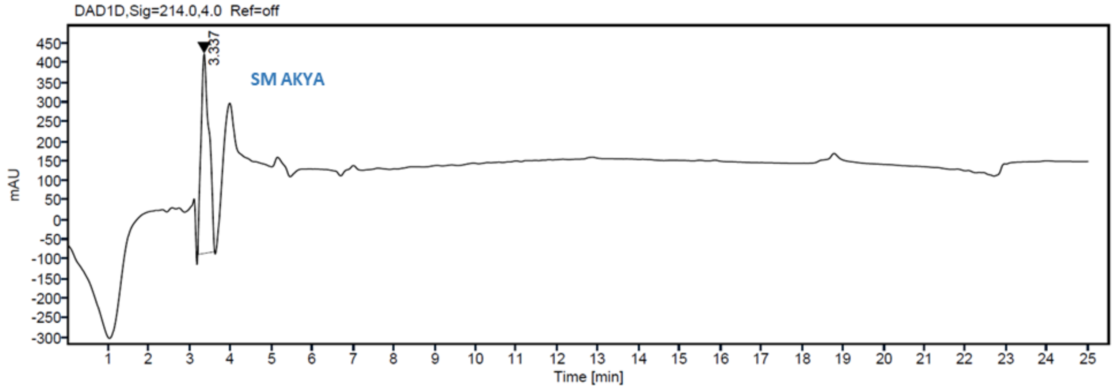

HPLC-MS chromatogram of AKYA-NH_2 (**1f**):

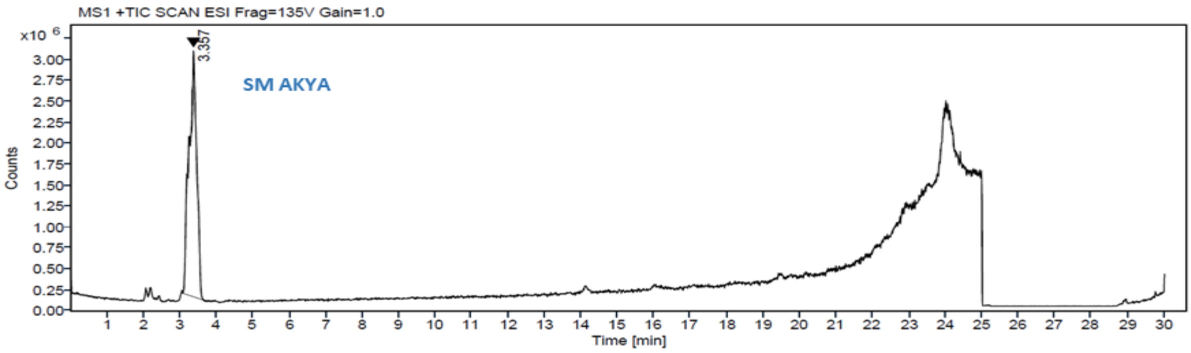

HPLC-UV chromatogram (214 nm) of crude of the reaction:

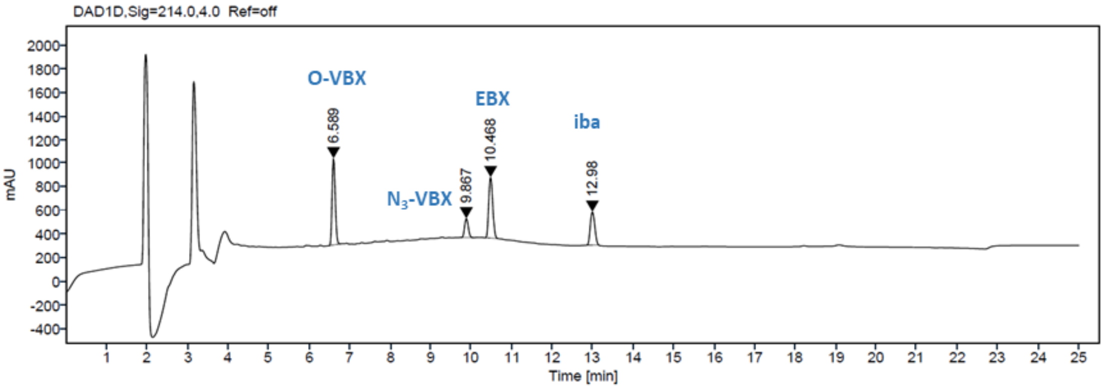

HPLC-MS chromatogram of crude of the reaction:

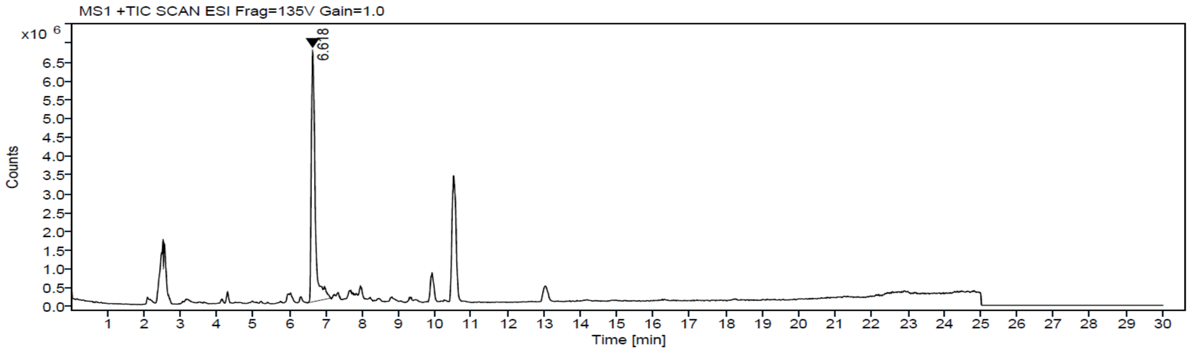

HPLC-UV chromatogram (214 nm) of O-VBX of AKYA-NH_2 (**3f**):

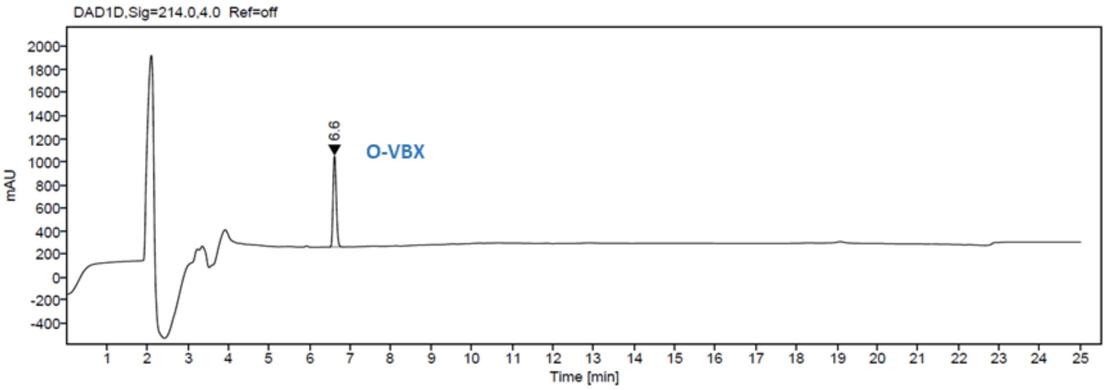

Relative ratio based on reverse phase HPLC-UV chromatogram at 214 nm: > 99%

Relative ratio based on reverse phase HPLC-MS chromatogram: > 99%

O-VBX of ACYA-NH$_2$ (3g)

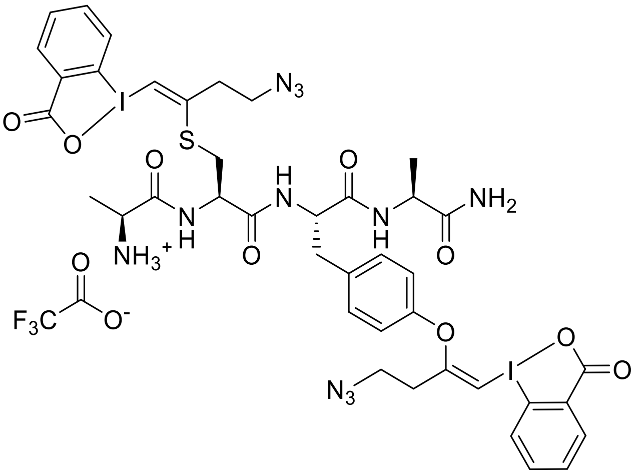

Starting from ACYA-NH$_2$ (**1g**) (10.8 mg, 20.0 µmol), O-VBX of ACYA-NH$_2$ (**3g**) (9.80 mg, 8.18 µmol, 40% yield) was obtained, as a white solid after lyophilization (retention time 8.3 min).

^{1}H NMR (400 MHz, MeOD) δ 8.29 (td, J = 7.3, 2.0 Hz, 3H, ArH, NH), 7.99 (dd, J = 8.0, 1.2 Hz, 1H, NH), 7.85 – 7.63 (m, 6H, ArH), 7.29 – 7.22 (m, 2H, ArH), 7.11 (s, 1H, SCCH-I), 6.97 – 6.91 (m, 2H, ArH), 6.50 (d, J = 0.9 Hz, 1H, OCCH-I), 4.63 (dd, J = 9.5, 5.0 Hz, 1H, CH-Tyr), 4.42 (dd, J = 7.8, 6.7 Hz, 2H, CH-Cys, CH-Ala-C-Term), 3.83 (q, J = 7.2 Hz, 1H, CH-Ala-N-Term), 3.75 (t, J = 6.1 Hz, 2H, SCCH$_2$CH_2N$_3$), 3.65 – 3.57 (m, 3H, NH_2, OCCH$_2$CH_2N$_3$), 3.24 – 3.03 (m, 6H, NH_2, CH_2-Tyr, OCCH_2CH$_2$N$_3$), 2.99 (t, J = 6.0 Hz, 1H, CH_2-Cys), 2.95 – 2.79 (m, 3H, CH_2-Cys, SCCH_2CH$_2$N$_3$), 1.38 (d, J = 7.2 Hz, 3H, CH_3), 1.28 (d, J = 7.1 Hz, 3H, CH_3). **^{13}C NMR** (151 MHz, MeOD) δ 177.3, 172.4, 171.0, 170.4, 170.3, 170.3, 168.1, 159.9, 154.0, 135.8, 135.7, 133.9, 133.8, 133.7, 133.6, 132.3, 132.3, 132.0, 132.0, 129.1, 129.0, 120.4, 114.6, 112.7, 104.6, 83.0, 55.4, 54.8, 50.9, 50.2, 50.0, 49.6, 38.2, 37.0, 33.7, 33.0, 18.5, 17.8.[21] **HRMS** (ESI/QTOF) m/z: [M + H]$^+$ Calcd for $C_{40}H_{44}I_2N_{11}O_9S^+$ 1108.1128; Found 1108.1108.

HPLC-UV chromatogram (214 nm) of ACYA-NH$_2$ (**1g**):

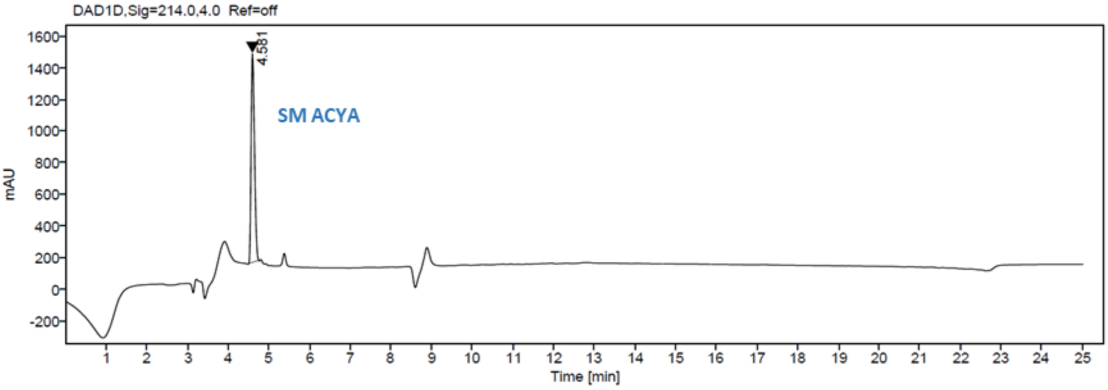

HPLC-UV chromatogram (214 nm) of crude of the reaction:

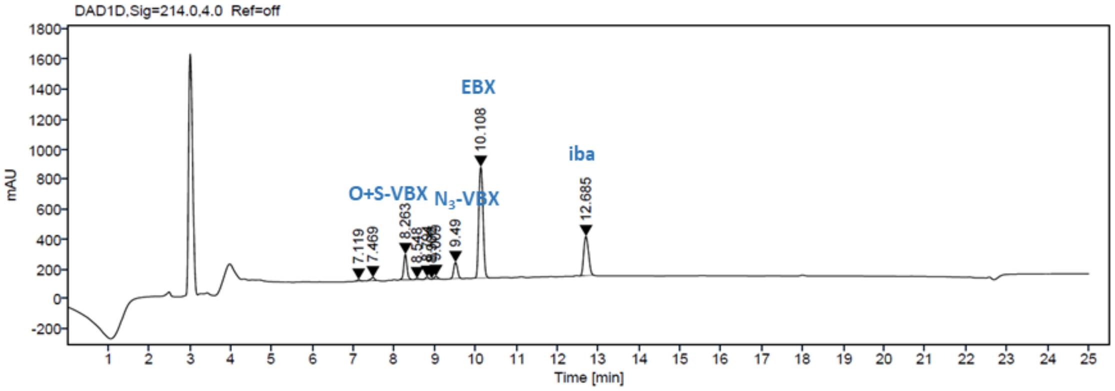

HPLC-MS chromatogram of crude of the reaction:

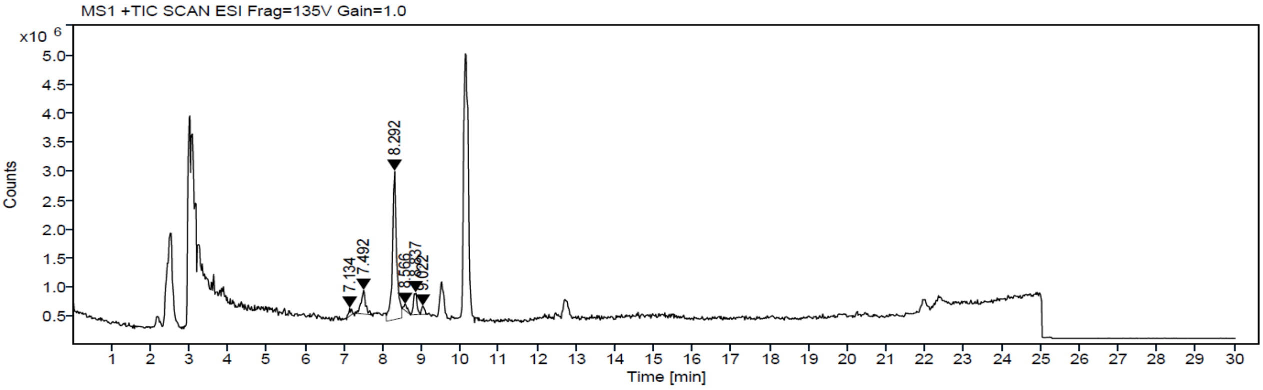

HPLC-UV chromatogram (214 nm) of O-VBX of ACYA-NH_2 (**3g**):

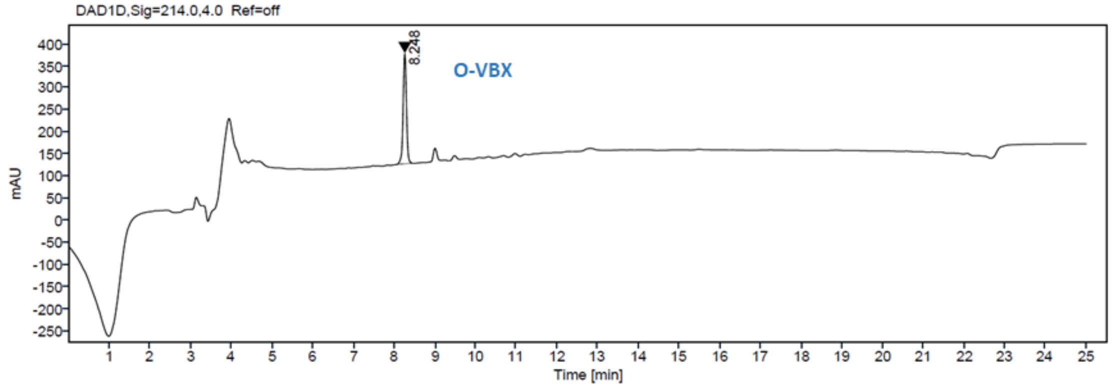

Relative ratio based on reverse phase HPLC-UV chromatogram at 214 nm: 70%

Relative ratio based on reverse phase HPLC-MS chromatogram: 70%

O-VBX of ASYA-NH_2 (3h)

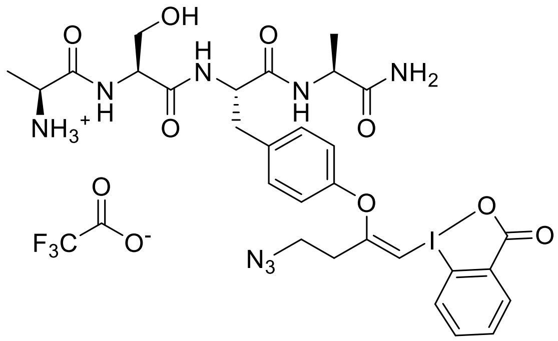

Starting from ASYA-NH_2 (**1h**) (10.5 mg, 20.0 µmol), O-VBX of ASYA-NH_2 (**3h**) (15.5 mg, 17.9 µmol, 90% yield) was obtained, as a white solid after lyophilization (retention time 7.5 min).

^{1}H NMR (400 MHz, MeOD) δ 8.30 (dd, *J* = 7.4, 1.9 Hz, 1H, Ar*H*), 8.01 (dd, *J* = 8.0, 1.1 Hz, 1H, Ar*H*), 7.84 – 7.71 (m, 2H, Ar*H*), 7.31 – 7.23 (m, 2H, Ar*H*), 7.00 – 6.92 (m, 2H, Ar*H*), 6.51 (s, 1H, C*H*-I), 4.58 (dd, *J* = 8.6, 5.2 Hz, 1H, C*H*-Tyr), 4.43 (t, *J* = 6.0 Hz, 1H, C*H*-Ser), 4.31 (q, *J* = 7.2 Hz, 1H, C*H*-Ala-C-Term), 3.94 (q, *J* = 7.0 Hz, 1H, C*H*-Ala-N-Term), 3.79 (dd, *J* = 10.8, 5.9 Hz, 1H, C*H*₂-Ser), 3.71 (dd, *J* = 10.8, 6.3 Hz, 1H, CH_2-Ser), 3.60 (t, *J* = 6.3 Hz, 2H, $CH_2CH_2N_3$), 3.19 (dd, *J* = 14.1, 5.2 Hz, 1H, CH_2-Tyr), 2.99 – 2.88 (m, 3H, CH_2-Tyr, $CH_2CH_2N_3$), 1.42 (d, *J* = 7.0 Hz, 3H, CH_3), 1.33 (d, *J* = 7.2 Hz, 3H, CH_3). **^{13}C NMR** (101 MHz, MeOD) δ 177.2, 172.6, 172.1, 171.1, 170.4, 168.4, 154.0, 136.0, 135.8, 133.7, 133.1, 132.3, 132.0, 129.3, 120.4, 114.4, 82.6, 62.9, 56.5, 56.0, 50.1, 49.7, 49.5, 37.7, 32.8, 18.3, 17.6. [21] **HRMS** (ESI/QTOF) m/z: $[M + H]^+$ Calcd for $C_{29}H_{36}IN_8O_8^+$ 751.1695; Found 751.1706.

HPLC-UV chromatogram (214 nm) of ASYA-NH_2 (**1h**):

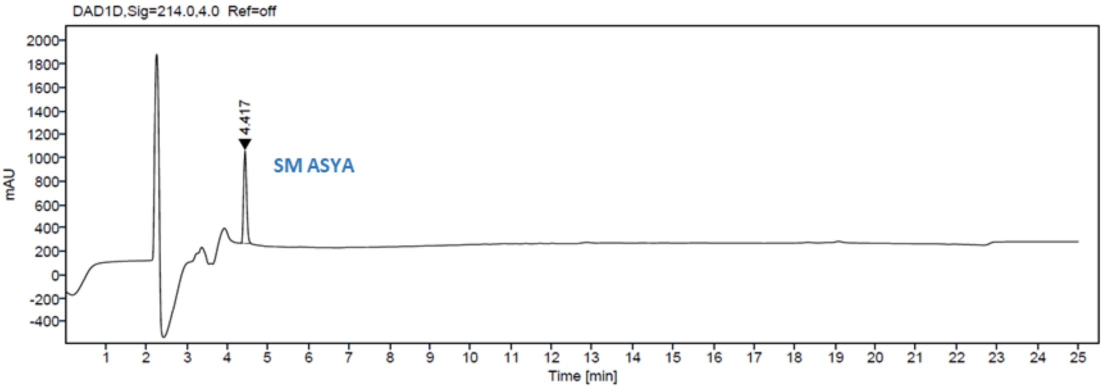

HPLC-UV chromatogram (214 nm) of crude of the reaction:

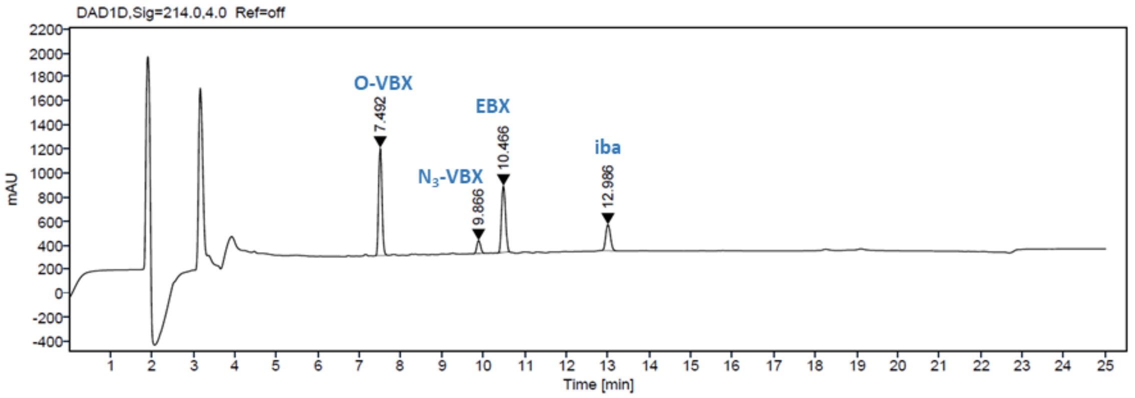

HPLC-MS chromatogram of crude of the reaction:

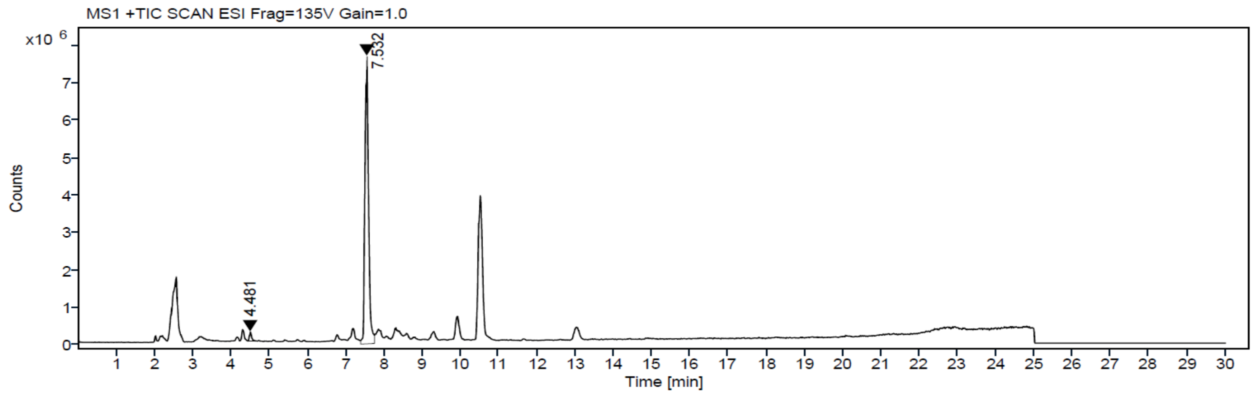

HPLC-UV chromatogram (214 nm) of O-VBX of ASYA-NH_2 (**3h**):

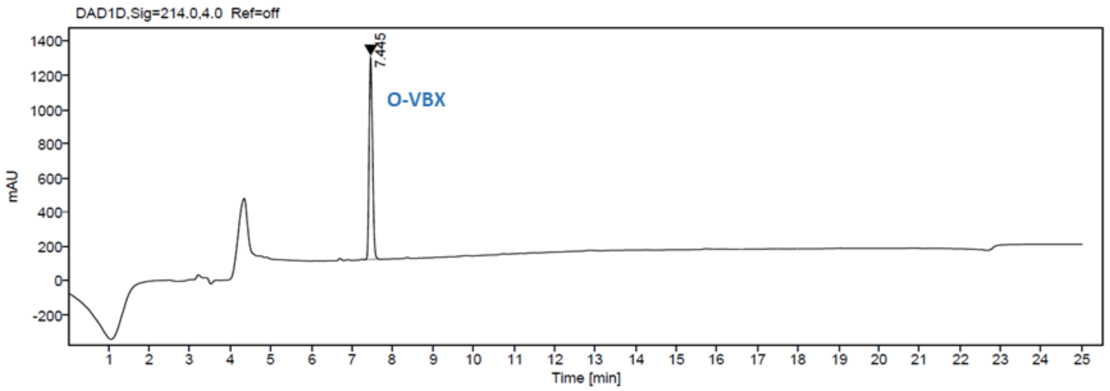

Relative ratio based on reverse phase HPLC-UV chromatograms at 214 nm: > 99%

Relative ratio based on reverse phase HPLC-MS chromatograms: > 95%

O-VBX of AMYA-NH$_2$ (3i)

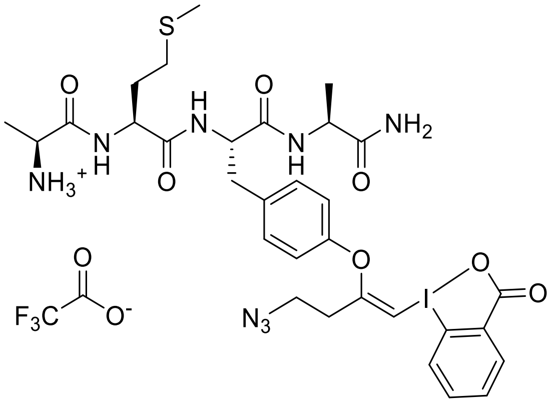

Starting from AMYA-NH$_2$ (**1i**) (11.4 mg, 20.0 µmol), O-VBX of AMYA-NH$_2$ (**3i**) (11.0 mg, 12.9 µmol, 64% yield) was obtained, as a white solid after lyophilization (retention time 8.1 min).

^{1}H NMR (400 MHz, MeOD) δ 8.29 (dd, *J* = 7.4, 1.9 Hz, 1H, Ar*H*), 8.00 (dd, *J* = 8.0, 1.3 Hz, 1H, Ar*H*), 7.83 – 7.70 (m, 2H, Ar*H*), 7.29 – 7.21 (m, 2H, Ar*H*), 6.99 – 6.91 (m, 2H, Ar*H*), 6.50 (d, *J* = 0.8 Hz, 1H, C*H*-I), 4.57 (dd, *J* = 8.8, 5.6 Hz, 1H, C*H*-Tyr), 4.40 (dd, *J* = 8.7, 5.5 Hz, 1H, C*H*-Met), 4.33 (q, *J* = 7.1 Hz, 1H, C*H*-Ala-C-Term), 3.92 (q, *J* = 7.1 Hz, 1H, , C*H*-Ala-N-Term), 3.61 (t, *J* = 6.3 Hz, 2H, $CH_2CH_2N_3$), 3.21 – 3.11 (m, 1H, CH_2-Tyr), 2.98 – 2.87 (m, 3H, $CH_2CH_2N_3$, CH_2-Tyr), 2.43 (qdd, *J* = 13.3, 9.0, 6.1 Hz, 2H, $CH_2CH_2SCH_3$), 2.03 (s, 3H, $CH_2CH_2SCH_3$), 1.97 (dddd, *J* = 14.4, 9.1, 6.7, 5.5 Hz, 1H, $CH_2CH_2SCH_3$), 1.85 (dtd, *J* = 14.3, 8.8, 5.7 Hz, 1H, $CH_2CH_2SCH_3$), 1.41 (d, *J* = 7.0 Hz, 3H, CH_3), 1.35 (d, *J* = 7.1 Hz, 3H, CH_3). **^{13}C NMR** (101 MHz, MeOD) δ 177.1, 173.1, 172.5, 171.1, 170.4, 168.2, 154.0, 135.8, 135.8, 133.6, 133.5, 132.4, 132.0, 129.1, 120.4, 114.5, 82.9, 55.7, 54.1, 50.1, 50.0, 37.9, 32.9, 32.8, 30.9, 18.5, 17.6, 15.2. *1 aliphatic carbon signal is not resolved (under the solvent peak)*. [21] **HRMS** (ESI/QTOF) m/z: [M + H]$^+$ Calcd for $C_{31}H_{40}IN_8O_7S^+$ 795.1780; Found 795.1783.

HPLC-UV chromatogram (214 nm) of AMYA-NH$_2$ (**1i**):

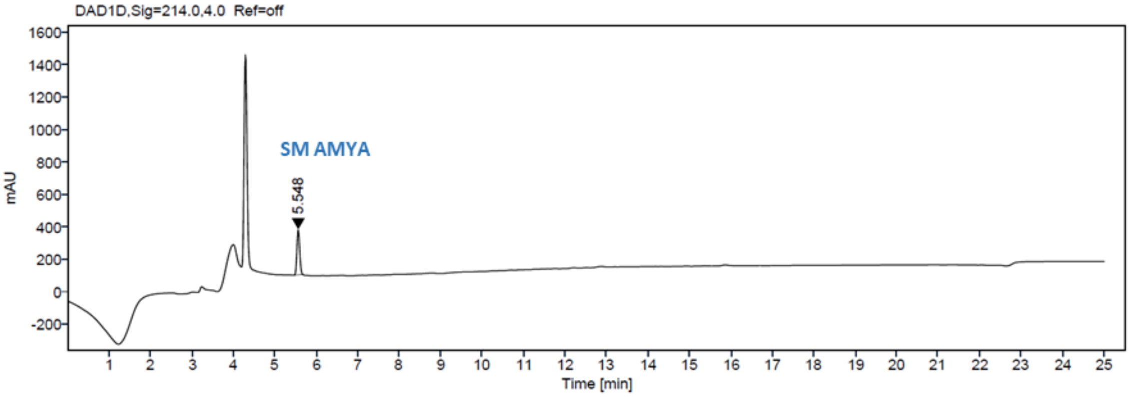

HPLC-UV chromatogram (214 nm) of crude of the reaction:

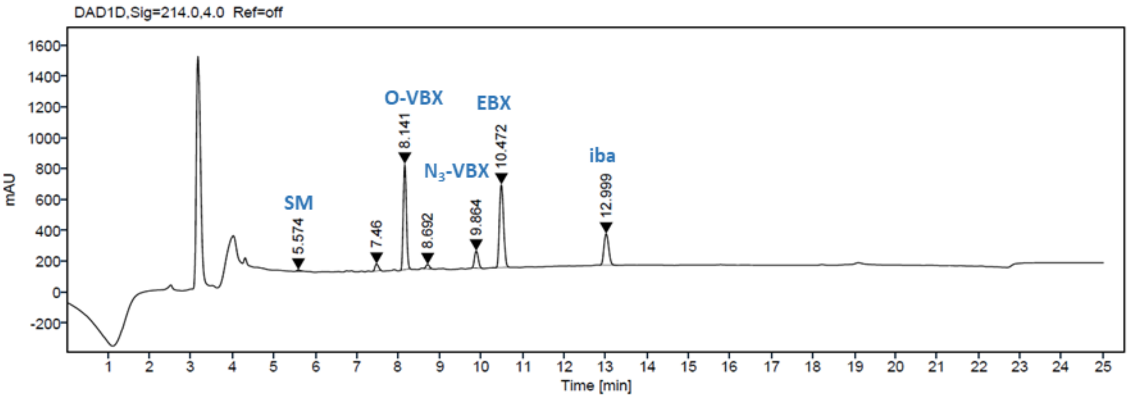

HPLC-MS chromatogram of crude of the reaction:

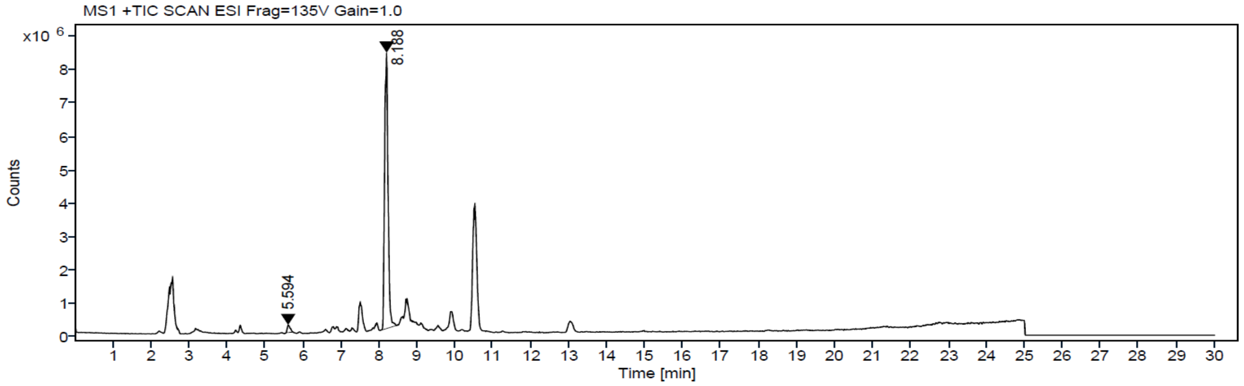

HPLC-UV chromatogram (214 nm) of O-VBX of AMYA-NH_2 (**3i**):

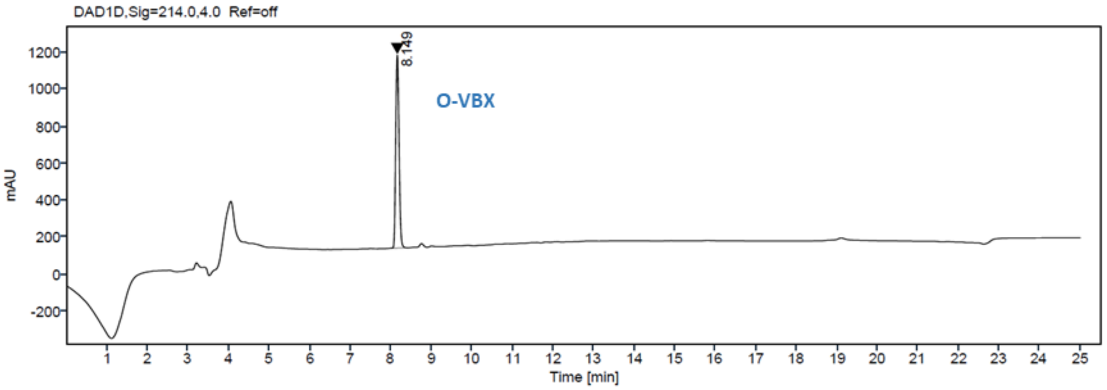

Relative ratio based on reverse phase HPLC-UV chromatogram at 214 nm: > 99%

Relative ratio based on reverse phase HPLC-MS chromatogram: > 99%

O-VBX of ADYA-NH$_2$ (3j)

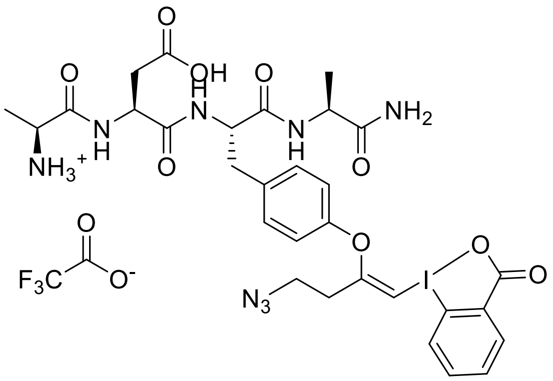

Starting from ADYA-NH$_2$ (**1j**) (11.0 mg, 20.0 μmol), O-VBX of ADYA-NH$_2$ (**3j**) (15.5 mg, 17.4 μmol, 87% yield) was obtained, as a white solid after lyophilization (retention time 7.6 min).

^{1}H NMR (400 MHz, MeOD) δ 8.29 (dd, *J* = 7.4, 1.9 Hz, 1H, Ar*H*), 8.00 (dd, *J* = 8.0, 1.1 Hz, 1H, Ar*H*), 7.84 – 7.70 (m, 2H, Ar*H*), 7.30 – 7.21 (m, 2H, Ar*H*), 7.01 – 6.92 (m, 2H, Ar*H*), 6.51 (s, 1H, C*H*-I), 4.64 (t, *J* = 6.9 Hz, 1H, C*H*-Asp), 4.48 (dd, *J* = 9.1, 5.1 Hz, 1H, C*H*-Tyr), 4.32 (q, *J* = 7.2 Hz, 1H, C*H*-Ala-C-Term), 3.91 – 3.82 (m, 1H, C*H*-Ala-N-Term), 3.61 (t, *J* = 6.3 Hz, 2H, $CH_2CH_2N_3$), 3.21 (dd, *J* = 14.1, 5.1 Hz, 1H, CH_2-Tyr), 2.99 – 2.80 (m, 4H, CH_2-Tyr, CH_2-Asp, $CH_2CH_2N_3$), 2.74 – 2.63 (m, 1H, CH_2-Asp), 1.37 (d, *J* = 7.2 Hz, 3H, CH_3), 1.33 (d, *J* = 7.1 Hz, 3H, CH_3). **^{13}C NMR** (101 MHz, MeOD) δ 177.3, 174.0, 172.5 (*2 carbon signals under this peak*), 171.0, 170.5, 168.3, 153.9, 136.1, 135.9, 133.6, 133.3, 132.3, 132.0, 129.2, 120.4, 114.6, 82.6, 56.3, 51.3, 50.3, 50.1, 49.5, 37.4, 36.5, 32.9, 18.1, 17.6. [21] **HRMS** (ESI/QTOF) m/z: [M + H]$^+$ Calcd for $C_{30}H_{36}IN_8O_9^+$ 779.1644; Found 779.1638.

HPLC-UV chromatogram (214 nm) of ADYA-NH$_2$ (**1j**):

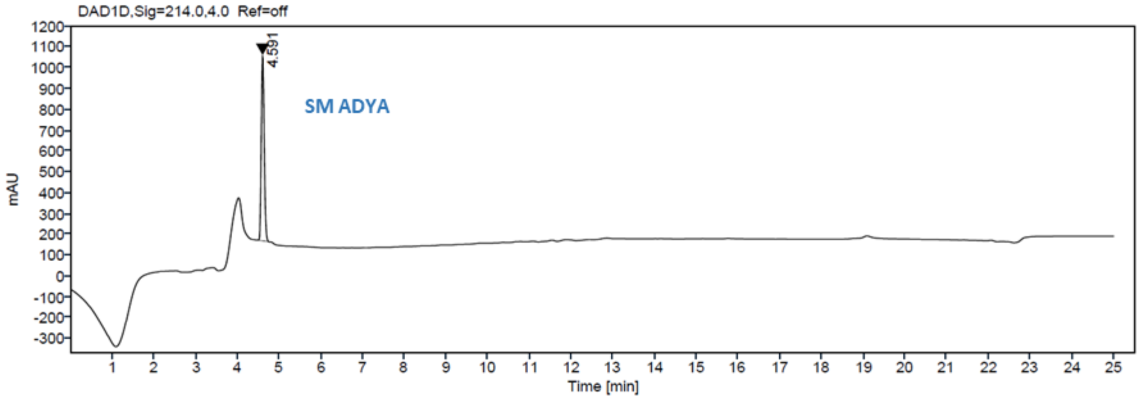

HPLC-UV chromatogram (214 nm) of crude of the reaction:

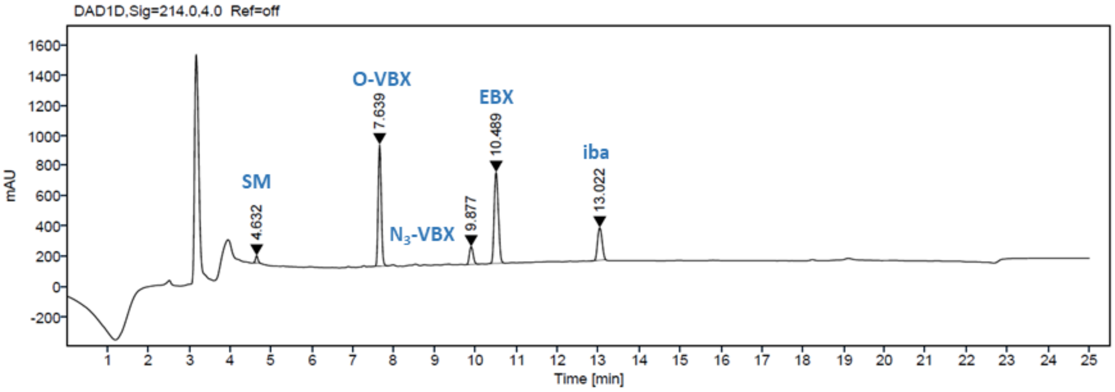

HPLC-MS chromatogram of crude of the reaction:

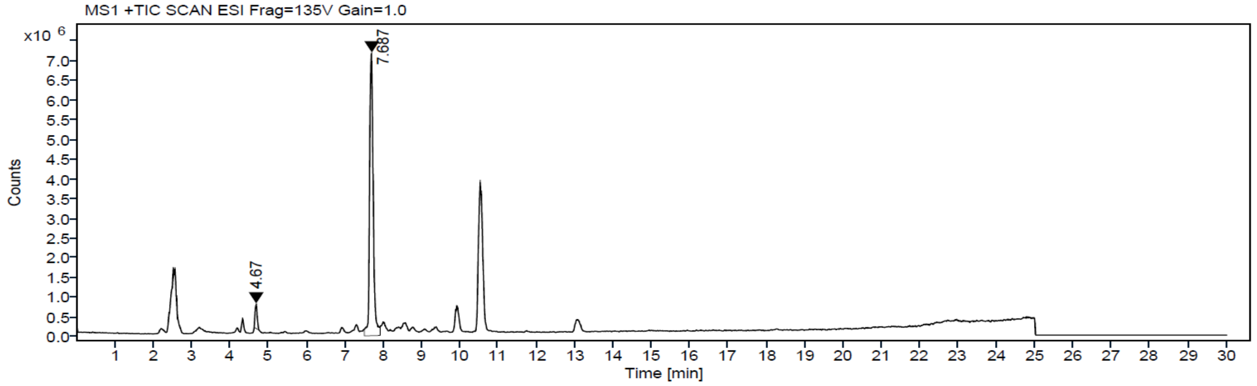

HPLC-UV chromatogram (214 nm) of O-VBX of ADYA-NH$_2$ (**3j**):

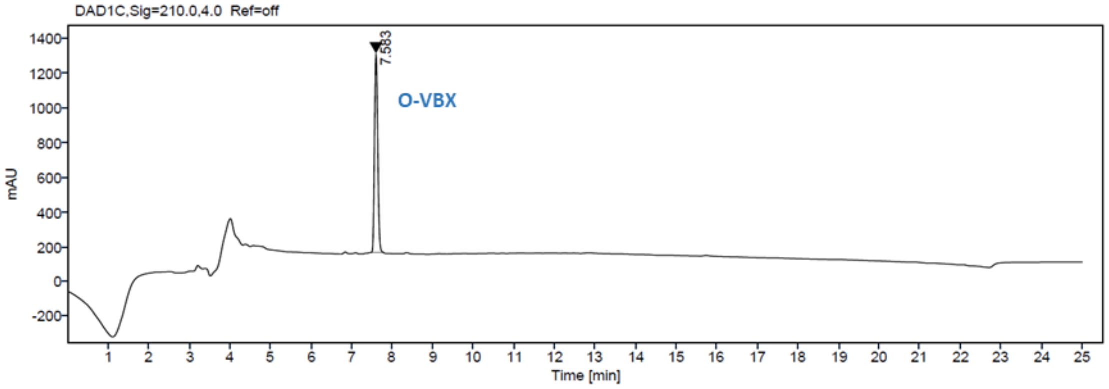

Relative ratio based on reverse phase HPLC-UV chromatograms at 214 nm: > 95%

Relative ratio based on reverse phase HPLC-MS chromatograms: > 95%

O-VBX of ANYA-NH_2 (3k)

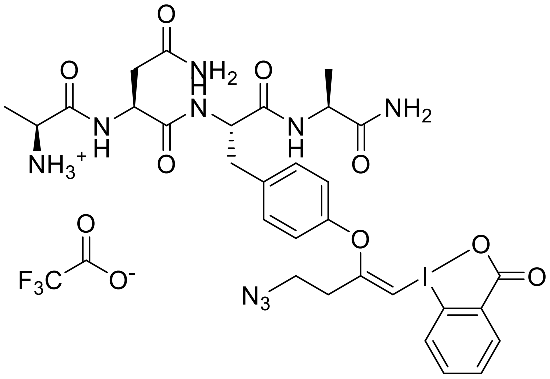

Starting from ANYA-NH_2 (**1k**) (11.0 mg, 20.0 µmol), O-VBX of ANYA-NH_2 (**3k**) (11.3 mg, 12.7 µmol, 63% yield) was obtained, as a white solid after lyophilization (retention time 8.2 min).

^{1}H NMR (400 MHz, MeOD) δ 8.27 (dd, *J* = 7.5, 1.8 Hz, 1H, Ar*H*), 7.99 (dd, *J* = 8.1, 1.0 Hz, 1H, Ar*H*), 7.77 (ddd, *J* = 8.1, 7.2, 1.8 Hz, 1H, Ar*H*), 7.73 (td, *J* = 7.4, 1.0 Hz, 1H, Ar*H*), 7.28 – 7.24 (m, 2H, Ar*H*), 6.98 – 6.94 (m, 2H, Ar*H*), 6.50 (d, *J* = 0.8 Hz, 1H, C*H*-I), 4.65 (t, *J* = 6.9 Hz, 1H, C*H*-Asn), 4.47 (dd, *J* = 9.6, 4.7 Hz, 1H, C*H*-Tyr), 4.33 (q, *J* = 7.2 Hz, 1H, C*H*-Ala-C-Term), 3.83 (q, *J* = 7.1 Hz, 1H, C*H*-Ala-N-Term), 3.61 (t, *J* = 6.4 Hz, 2H, $CH_2CH_2N_3$), 3.26 – 3.21 (m, 1H, CH_2-Tyr), 2.96 – 2.86 (m, 3H, CH_2-Tyr, $CH_2CH_2N_3$), 2.78 (dd, *J* = 15.8, 7.1 Hz, 1H, CH_2-Asn), 2.62 (dd, *J* = 15.8, 6.8 Hz, 1H, CH_2-Asn), 1.39 (d, *J* = 7.2 Hz, 3H, CH_3), 1.29 (d, *J* = 7.0 Hz, 3H, CH_3). **^{13}C NMR** (201 MHz, MeOD) δ 177.5, 174.5, 172.8, 172.6, 170.8, 170.4, 168.0, 154.0, 136.1, 135.6, 134.1, 133.5, 132.2, 131.9, 128.9, 120.3, 114.7, 83.4, 56.4, 51.5, 50.4, 50.1, 49.5, 37.6, 37.3, 33.0, 18.1, 17.6. [21] **HRMS** (ESI/QTOF) m/z: $[M + H]^+$ Calcd for $C_{30}H_{37}IN_9O_8^+$ 778.1804; Found 778.1799.

HPLC-MS chromatogram of ANYA-NH_2 (**1k**):

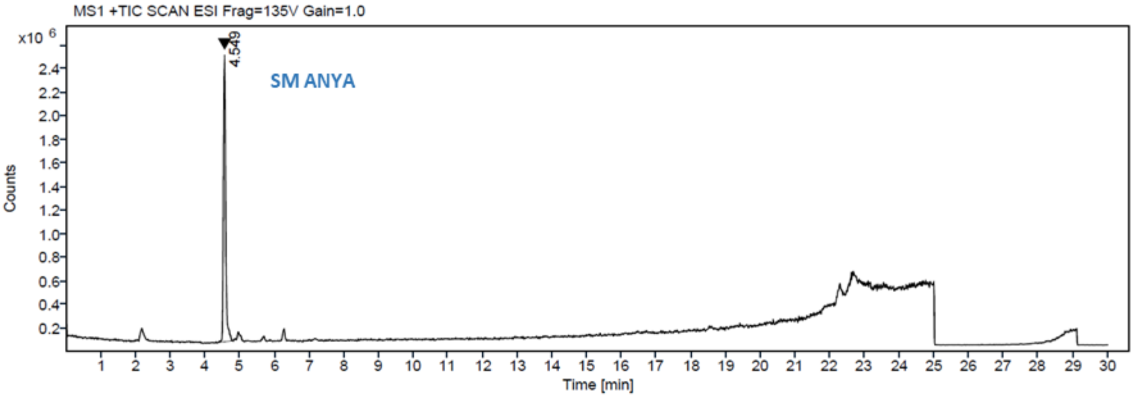

HPLC-UV chromatogram (214 nm) of crude of the reaction:

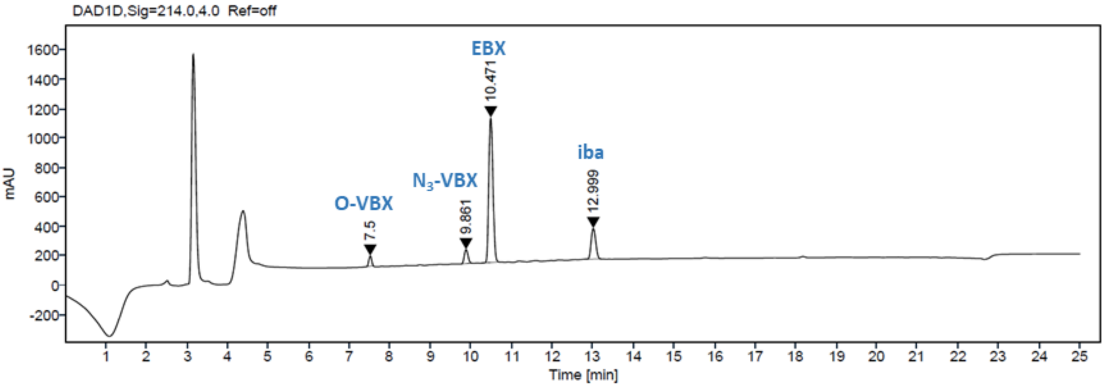

HPLC-MS chromatogram of crude of the reaction:

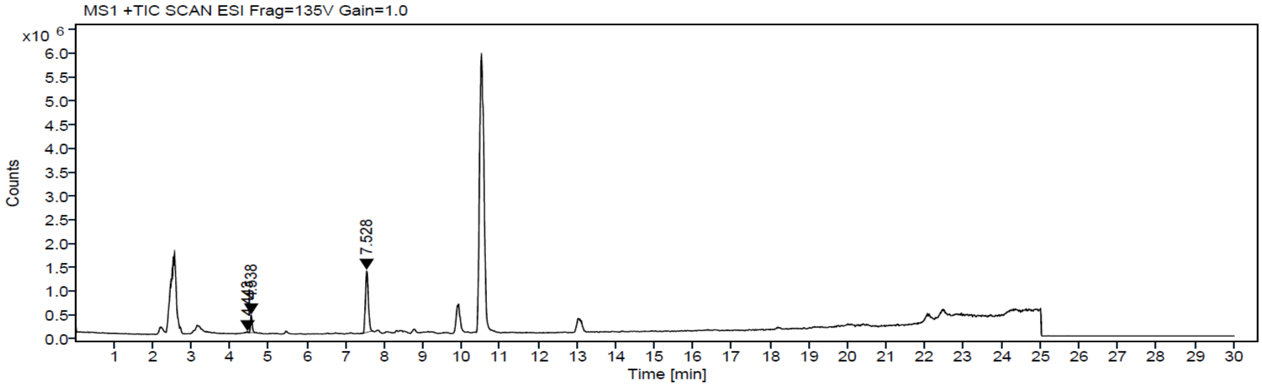

HPLC-UV chromatogram (214 nm) of O-VBX of ANYA-NH$_2$ (**3k**):

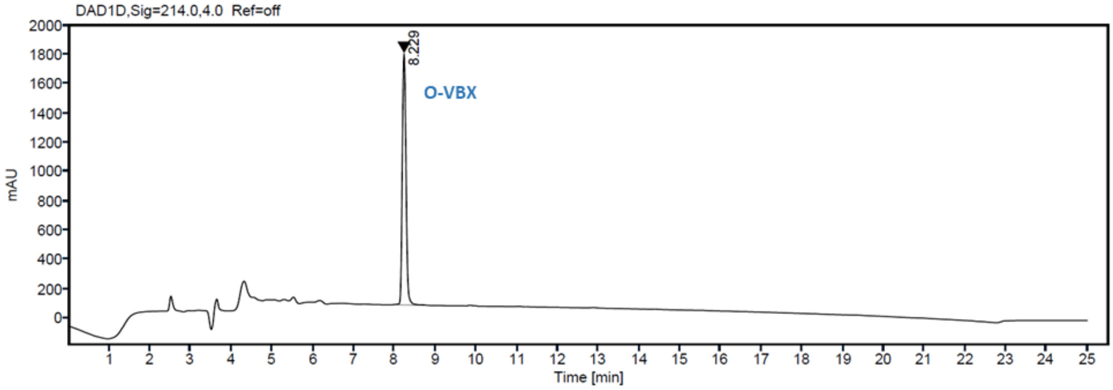

Relative ratio based on reverse phase HPLC-UV chromatogram at 214 nm: > 99%

Relative ratio based on reverse phase HPLC-MS chromatogram: 85%

O-VBX of AYLA-NH_2 (3l)

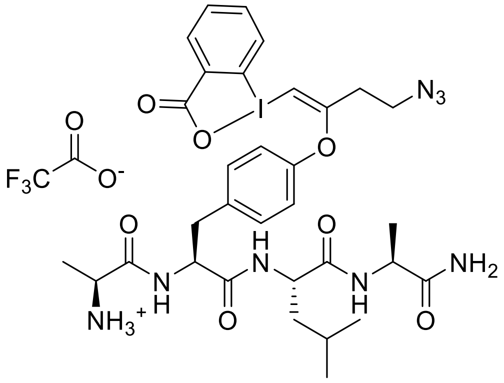

Starting from AYLA-NH_2 (**1l**) (11.0 mg, 20.0 µmol), O-VBX of AYLA-NH_2 (**3l**) (14.2 mg, 15.9 µmol, 80% yield) was obtained, as a white solid after lyophilization (retention time 8.3 min).

^{1}H NMR (400 MHz, MeOD) δ 8.28 (dt, *J* = 7.7, 1.8 Hz, 1H, Ar*H*), 8.00 (dd, *J* = 8.1, 1.1 Hz, 1H, Ar*H*), 7.84 – 7.70 (m, 2H, Ar*H*), 7.32 – 7.24 (m, 2H, Ar*H*), 7.01 – 6.92 (m, 2H, Ar*H*), 6.46 (d, *J* = 0.8 Hz, 1H, C*H*-I), 4.62 (dd, *J* = 9.4, 5.5 Hz, 1H, C*H*-Tyr), 4.42 – 4.34 (m, 1H, C*H*-Ala-C-Term), 4.30 (q, *J* = 7.1 Hz, 1H, C*H*-Leu), 3.88 (p, *J* = 6.8 Hz, 1H, C*H*-Ala-N-Term), 3.65 (t, *J* = 6.3 Hz, 2H, $CH_2CH_2N_3$), 3.21 – 3.12 (m, 1H, C*H_2*-Tyr), 2.95 (t, *J* = 6.2 Hz, 2H, $CH_2CH_2N_3$), 2.92 – 2.81 (m, 1H, C*H_2*-Tyr), 1.66 (dq, *J* = 13.0, 6.5 Hz, 1H, $CH_2CH(CH_3)_2$), 1.59 (dd, *J* = 7.8, 6.2 Hz, 2H, $CH_2CH(CH_3)_2$), 1.46 (d, *J* = 7.0 Hz, 3H, CH_3), 1.34 (d, *J* = 7.2 Hz, 3H, CH_3), 0.94 (d, *J* = 6.5 Hz, 3H, $CH_2CH(CH_3)_2$), 0.90 (d, *J* = 6.4 Hz, 3H, $CH_2CH(CH_3)_2$). **^{13}C NMR** (101 MHz, MeOD) δ 177.3, 174.0, 173.0, 171.1, 170.4, 168.4, 154.1, 135.9 (*2 carbon signals under this peak*), 133.6, 133.1, 132.2, 132.0, 129.1, 120.6, 114.7, 81.1, 56.1, 53.1, 50.0, 49.9, 41.8, 37.8, 33.7, 25.8, 23.5, 21.9, 18.5, 17.6. [21] **HRMS** (ESI/QTOF) m/z: $[M + H]^+$ Calcd for $C_{32}H_{42}IN_8O_7^+$ 777.2216; Found 777.2200.

HPLC-UV chromatogram (214 nm) of AYLA-NH_2 (**1l**):

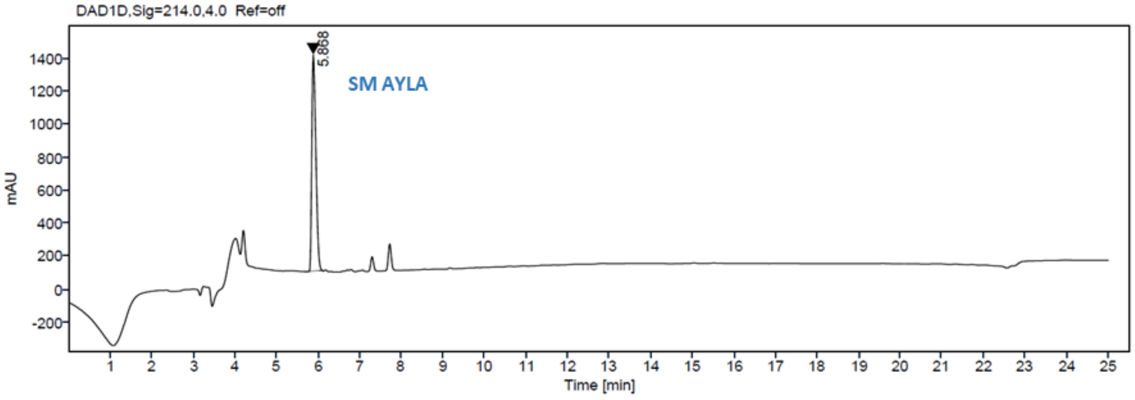

HPLC-UV chromatogram (214 nm) of crude of the reaction:

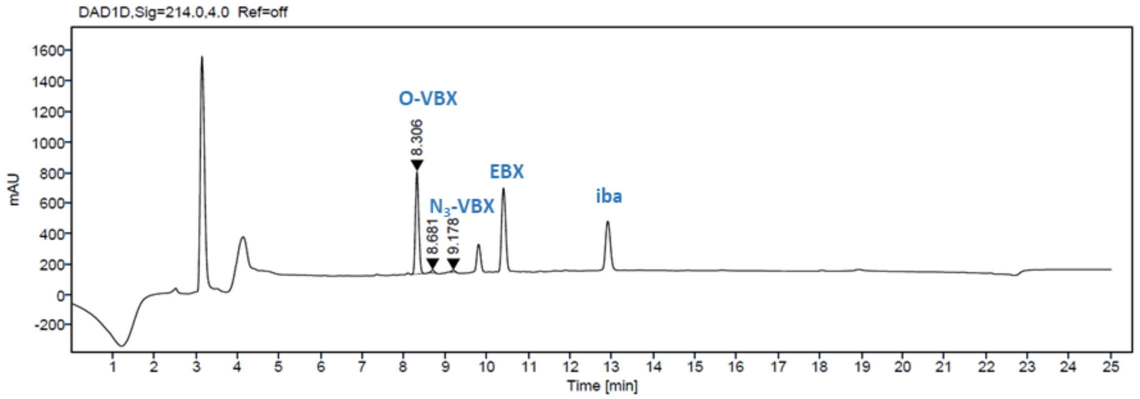

HPLC-MS chromatogram of crude of the reaction:

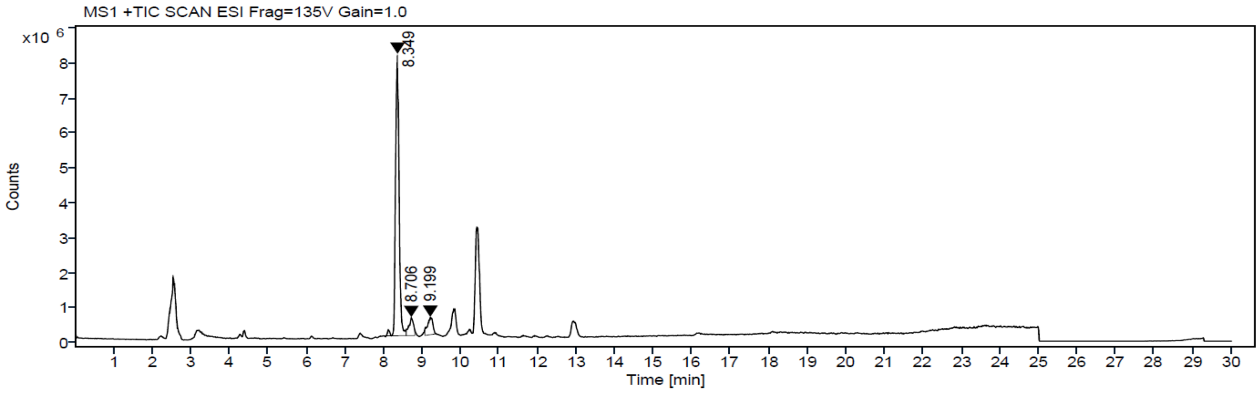

HPLC-UV chromatogram (214 nm) of O-VBX of AYLA-NH_2 (**3l**):

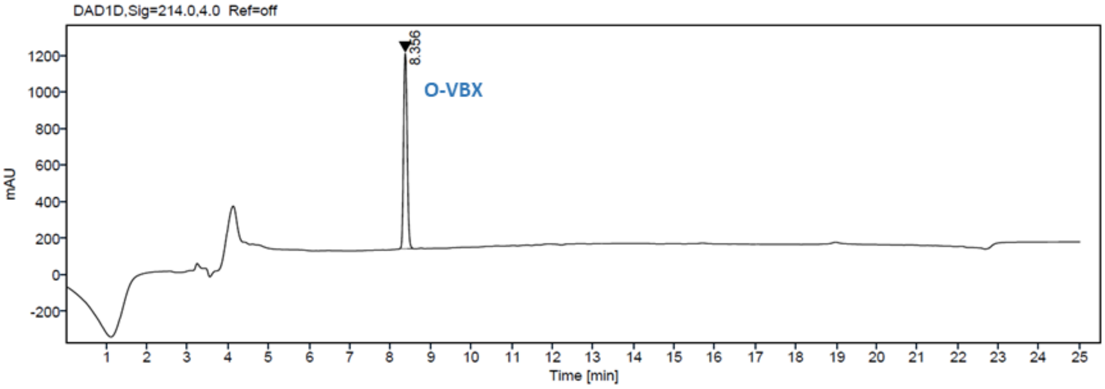

Relative ratio based on reverse phase HPLC-UV chromatograms at 214 nm: > 99%

Relative ratio based on reverse phase HPLC-MS chromatogram: 85%

O-VBX of YALA-NH$_2$ (3m)

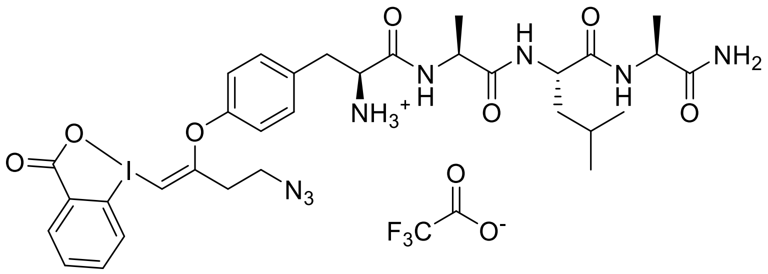

Starting from YALA-NH$_2$ (**1m**) (11.0 mg, 20.0 μmol), O-VBX of YALA-NH$_2$ (**3m**) (8.20 mg, 9.21 μmol, 46% yield) was obtained, as a white solid after lyophilization (retention time 8.3 min).

^{1}H NMR (400 MHz, MeOD) δ 8.29 (dd, *J* = 7.4, 1.9 Hz, 1H, Ar*H*), 8.00 (dd, *J* = 8.0, 1.2 Hz, 1H, Ar*H*), 7.84 – 7.70 (m, 2H, Ar*H*), 7.32 – 7.25 (m, 2H, Ar*H*), 7.05 – 6.98 (m, 2H, Ar*H*), 6.52 (d, *J* = 0.9 Hz, 1H, C*H*-I), 4.42 (q, *J* = 7.1 Hz, 1H, C*H*-Ala), 4.39 – 4.29 (m, 2H, C*H*-Tyr, C*H*-Leu), 4.04 (t, *J* = 7.0 Hz, 1H, C*H*-Ala-C-Term), 3.64 (t, *J* = 6.4 Hz, 2H, $CH_2CH_2N_3$), 3.22 (dd, *J* = 14.2, 6.6 Hz, 1H, CH_2-Tyr), 3.06 – 2.98 (m, 1H, CH_2-Tyr), 2.96 (t, *J* = 6.3 Hz, 2H, $CH_2CH_2N_3$), 1.78 – 1.65 (m, 1H, $CH_2CH(CH_3)_2$), 1.65 – 1.56 (m, 2H, $CH_2CH(CH_3)_2$), 1.38 (t, *J* = 7.3 Hz, 6H, CH_3), 0.93 (d, *J* = 6.5 Hz, 3H, $CH_2CH(CH_3)_2$), 0.87 (d, *J* = 6.4 Hz, 3H, $CH_2CH(CH_3)_2$). **^{13}C NMR** (101 MHz, MeOD) δ 177.3, 174.3, 174.2, 170.3, 169.0, 168.0, 154.7, 135.8, 133.6, 133.5, 133.0, 132.6, 132.0, 129.1, 121.0, 114.9, 82.6, 55.4, 53.2, 50.4, 50.0, 49.7, 41.6, 37.7, 33.2, 25.8, 23.6, 21.9, 18.6, 18.3. [21] **HRMS** (ESI/QTOF) m/z: [M + H]$^+$ Calcd for $C_{32}H_{42}IN_8O_7^+$ 777.2216; Found 777.2244.

HPLC-UV chromatogram (214 nm) of YALA-NH$_2$ (**1m**):

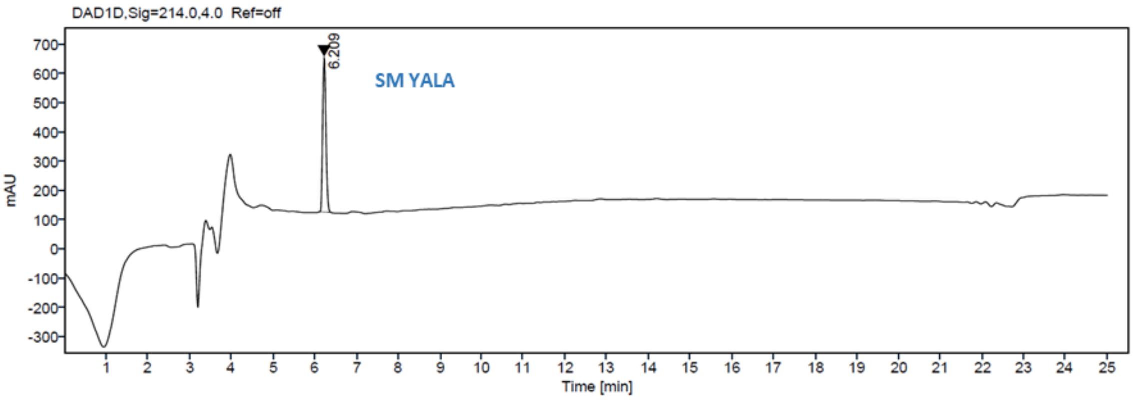

HPLC-UV chromatogram (214 nm) of crude of the reaction:

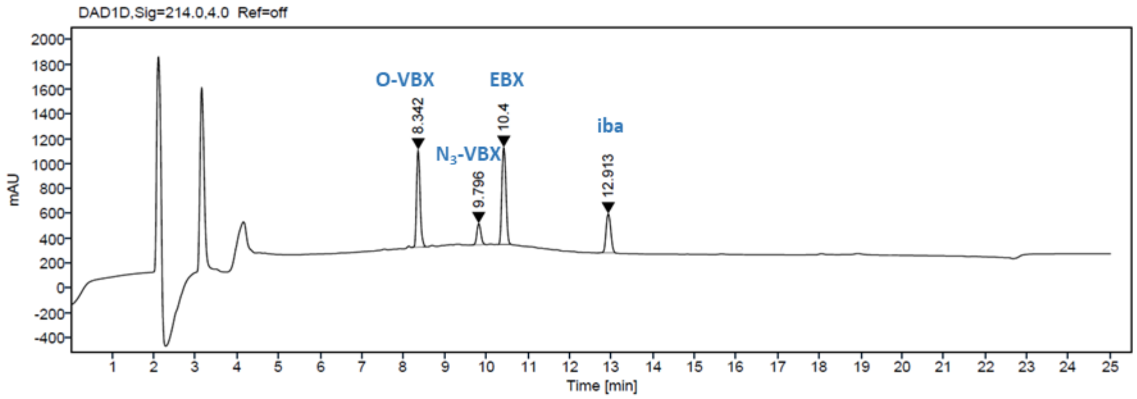

HPLC-MS chromatogram of crude of the reaction:

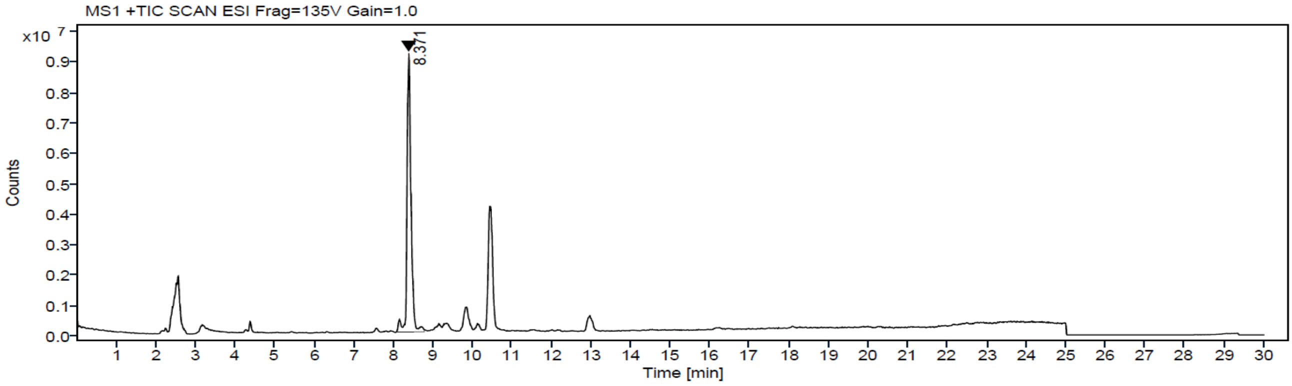

HPLC-UV chromatogram (214 nm) of O-VBX of YALA-NH_2 (**3m**):

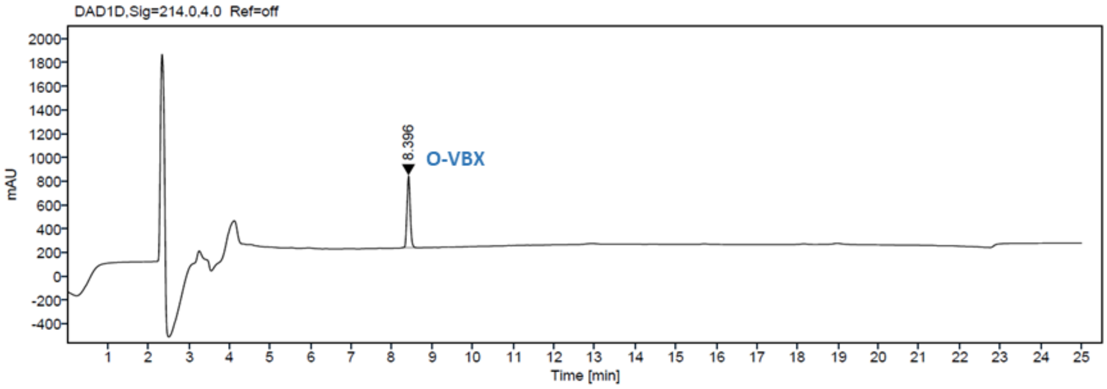

Relative ratio based on reverse phase HPLC-UV chromatogram at 214 nm: > 99%

Relative ratio based on reverse phase HPLC-MS chromatogram: > 99%

O-VBX of Ac-AFGY (3n)

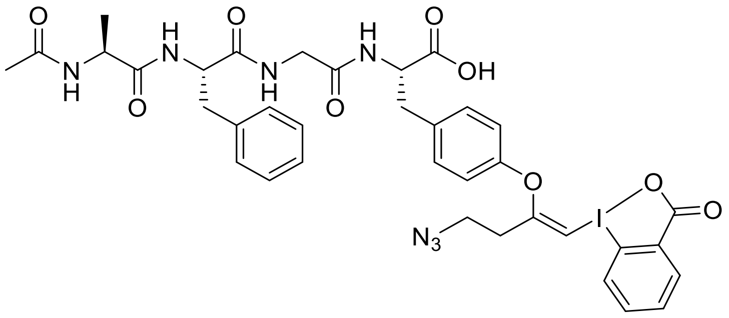

Starting from Ac-AFGY (**1n**) (9.97 mg, 20.0 μmol), O-VBX of Ac-AFGY (**3n**) (9.90 mg, 11.8 μmol, 59% yield) was obtained, as a white solid after lyophilization (retention time 10.5 min).

^{1}H NMR (400 MHz, MeOD) δ 8.33 (dd, *J* = 7.5, 1.8 Hz, 1H, Ar*H*), 8.23 – 8.16 (m, 1H, N*H*), 8.06 (dd, *J* = 8.2, 1.0 Hz, 1H, Ar*H*), 7.97 (d, *J* = 8.4 Hz, 1H, N*H*), 7.88 – 7.80 (m, 1H, Ar*H*), 7.76 (td, *J* = 7.4, 1.0 Hz, 1H, Ar*H*), 7.34 – 7.22 (m, 4H, Ar*H*), 7.19 (ddt, *J* = 6.8, 3.1, 1.3 Hz, 3H, Ar*H*), 7.03 – 6.95 (m, 2H, Ar*H*), 6.51 (s, 1H, C*H*-I), 4.69 – 4.59 (m, 1H, C*H*-Phe), 4.48 – 4.36 (m, 1H, C*H*-Tyr), 4.18 (q, *J* = 7.1 Hz, 1H, C*H*-Ala-N-Term), 3.97 – 3.87 (m, 1H, CH_2-Gly), 3.64 – 3.52 (m, 3H, CH_2-Gly, $CH_2CH_2N_3$), 3.22 (dt, *J* = 13.9, 4.9 Hz, 2H, CH_2-Phe), 3.08 – 2.95 (m, 2H, CH_2-Tyr), 2.93 (t, *J* = 6.4 Hz, 2H, $CH_2CH_2N_3$), 1.93 (s, 3H, NHCOCH_3), 1.19 (d, *J* = 7.2 Hz, 3H, CH_3). **^{13}C NMR** (101 MHz, MeOD) δ 175.4, 174.0, 173.8, 173.6, 171.4, 170.6, 169.1, 153.7, 138.6, 136.8, 136.4, 134.0, 132.4, 132.2, 131.3, 130.3, 129.8, 129.5, 127.8, 120.7, 114.1, 80.5, 56.6, 54.9, 50.9, 49.7, 43.3, 37.7, 37.5, 32.7, 22.5, 17.5. **HRMS** (ESI/QTOF) m/z: [M + H]$^+$ Calcd for $C_{36}H_{39}IN_7O_9^+$ 840.1848; Found 840.1847.

HPLC-UV chromatogram (214 nm) of Ac-AFGY (**1n**):

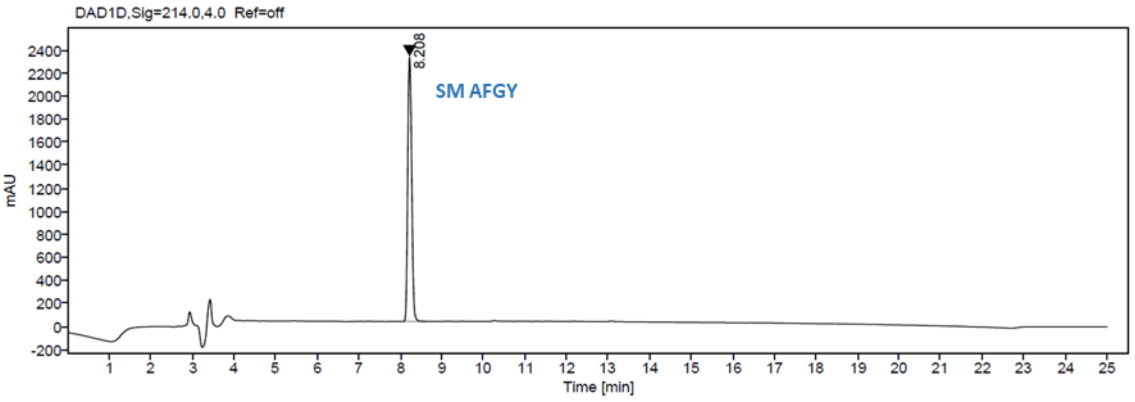

HPLC-UV chromatogram (214 nm) of crude of the reaction:

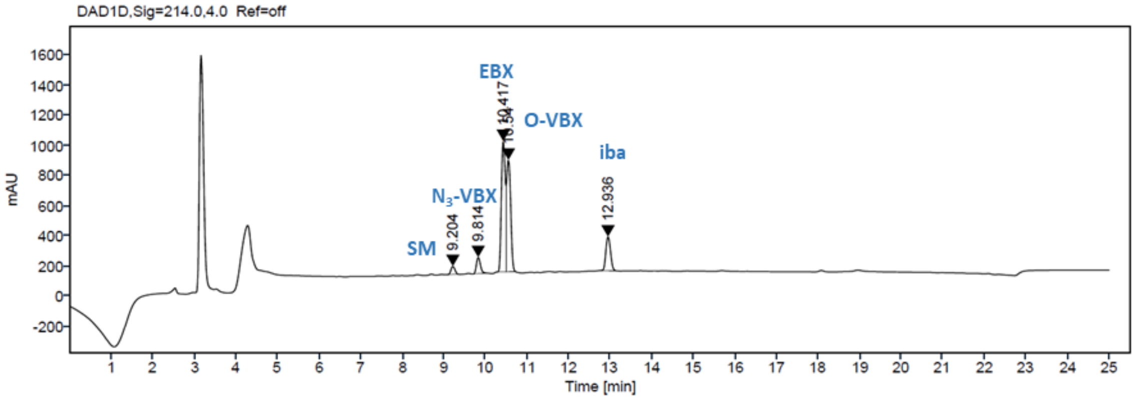

HPLC-MS chromatogram of crude of the reaction:

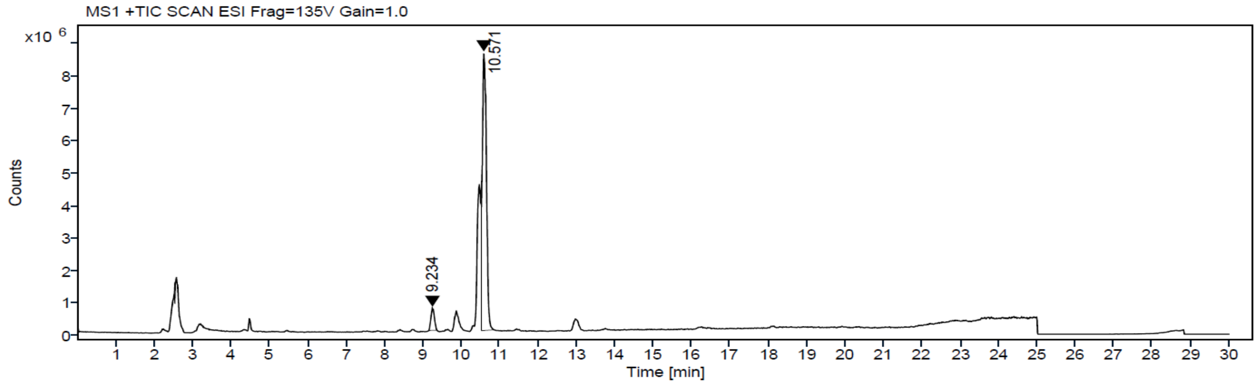

HPLC-UV chromatogram (214 nm) of O-VBX of Ac-AFGY (**3n**):

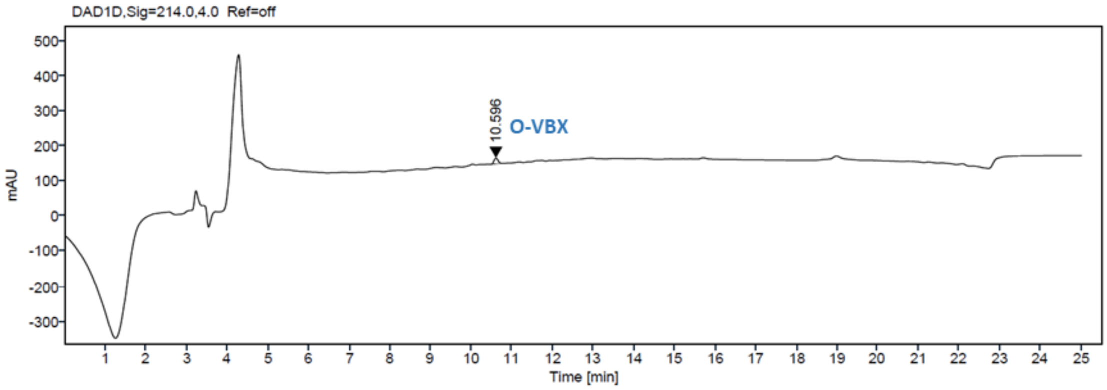

Relative ratio based on reverse phase HPLC-UV chromatogram at 214 nm: 94%

Relative ratio based on reverse phase HPLC-MS chromatogram: 94%

6. Scope of peptides

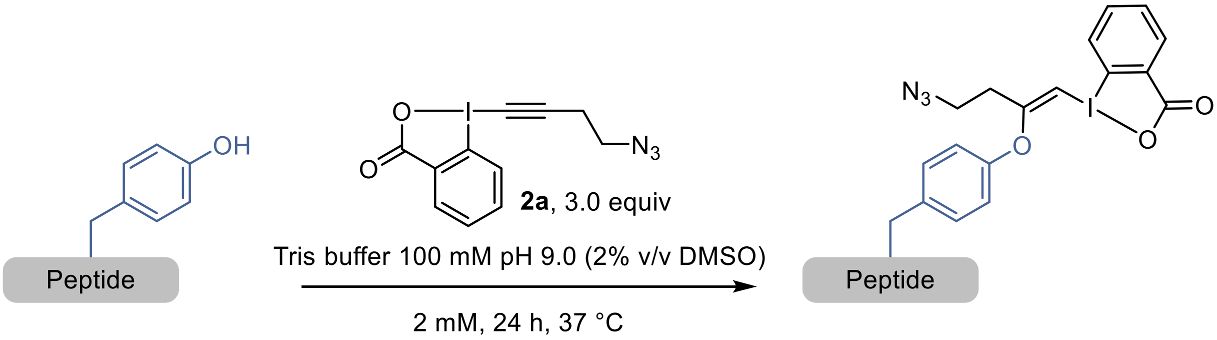

General procedure:

To a solution of peptide **1** (1.00 µmol or 1.00 mg, 1.00 equiv) in 100 mM Tris buffer pH 9.0 (490 µL), in a 0.5 mL Eppendorf Safe-Lock microcentrifuge tube, was added a 300 mM solution of N_3-EBX **2a** in DMSO (10.0 µL, 1.02 mg, 3.00 µmol, 3.00 equiv). The 2.00 mM solution was stirred at 37 °C for 24 h. No effort was made to exclude oxygen. The reaction was analysed by HPLC-MS.

The yields were approximated as the ratio of Aprod/Atotal where Aprod = area in mAU of the product peak and Atotal = area in mAU of all peptides products (product, starting material, and side products if present). The ratio were determined using HPLC-UV at 214 nm and/or HPLC-MS chromatograms.

MS/MS: the fragment map is displayed, with similarities in the isotopic pattern displayed in grey for assigned ions.

O-VBX of Vasopressin (3o)

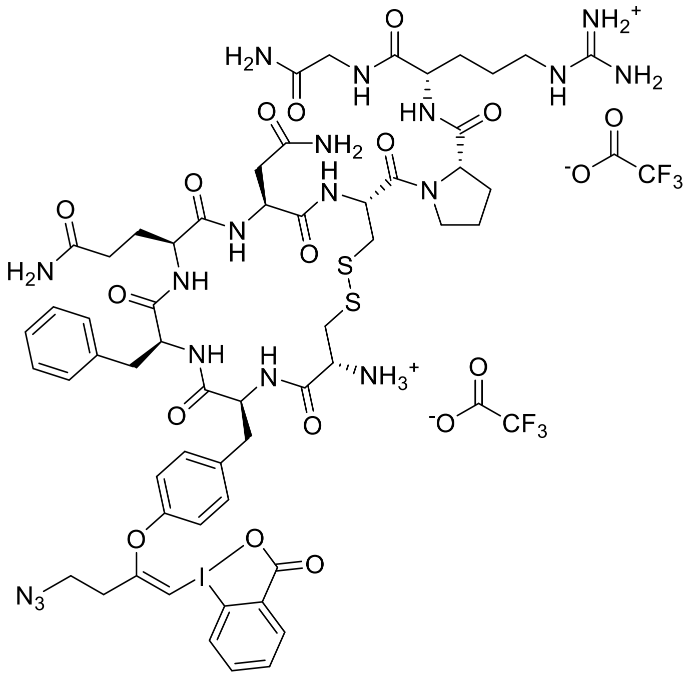

Starting from Vasopressin (**1o**) (1.00 mg, 0.762 µmol), O-VBX (**3o**) was obtained in 85% yield based on HPLC-MS (retention time 7.8 min).

HRMS (nanochip-ESI/LTQ-Orbitrap) m/z: $[M + H]^+$ Calcd for $C_{57}H_{74}IN_{18}O_{14}S_2^+$ 1425.4113; Found 1425.4132.

MS/MS characterization: *The lack of processing tools to assign fragment ions for such cyclic modified peptide did not allow us to confirm its structure based on the fragmentation spectra obtained.*

HPLC-UV chromatogram (214 nm) of a 2.00 mM solution of Vasopressin (**1o**) in 100 mM Tris buffer pH 9.0:

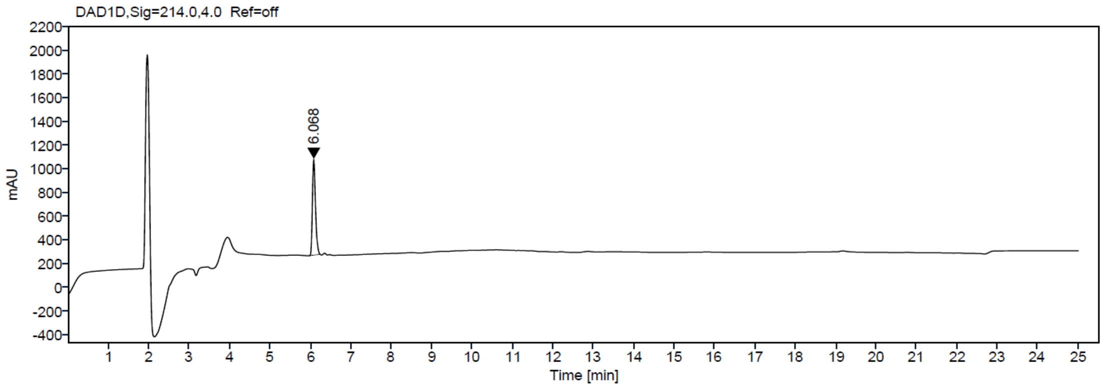

HPLC-UV chromatogram (214 nm) of crude of the reaction:

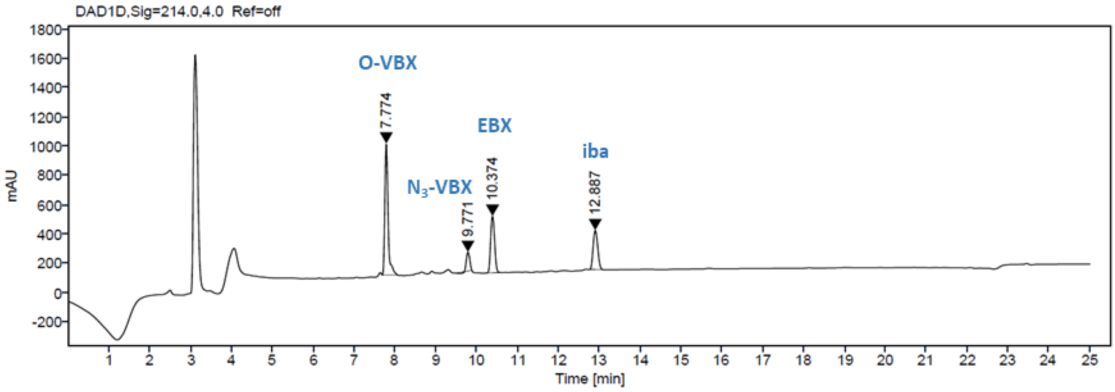

HPLC-MS chromatogram of crude of the reaction:

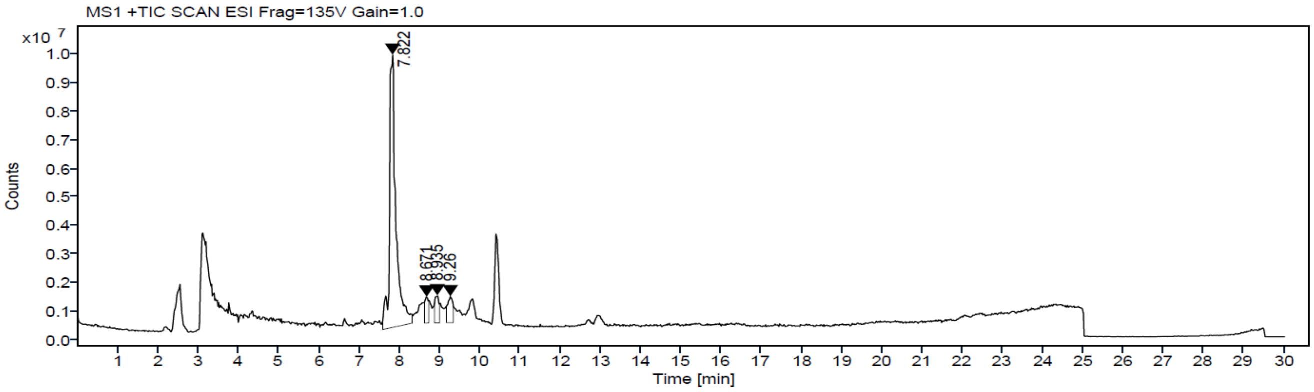

Yield based on HPLC-UV at 214 nm: 95%

Yield based on HPLC-MS: 85%

O-VBX of Oxytocin (3p)

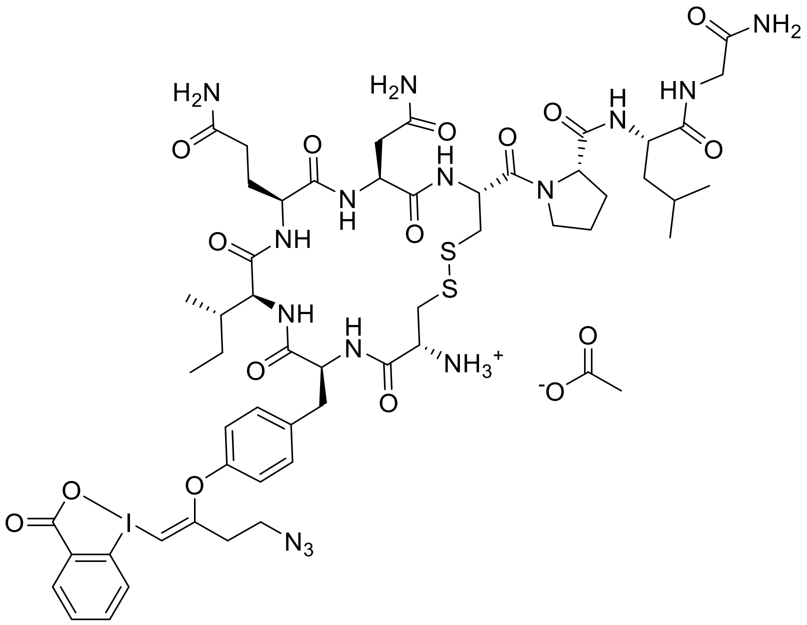

Starting from Oxytocin (**1p**) (1.07 mg, 1.00 µmol), O-VBX (**3p**) was obtained in 72% yield (retention time 8.5 min).

HRMS (nanochip-ESI/LTQ-Orbitrap) m/z: [M + H]$^+$ Calcd for $C_{54}H_{75}IN_{15}O_{14}S_2^+$ 1348.4099; Found 1348.4060.

MS/MS characterization:

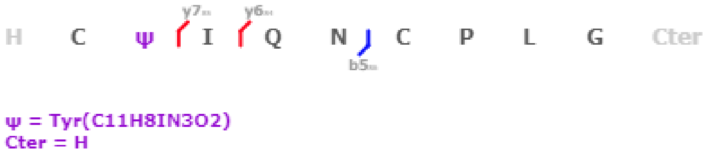

Sequence	Type	MF	m/z	Intensity	Similarity
**CYIQN**	b5	C38H48IN10O10S(+1)	963.232	1.04	86%
IQNCPLG	y7	C31H55N10O9S(+1)	743.3874	0.93	85%
QNCPLG	y6	C25H44N9O8S(+1)	630.3034	0.81	84%

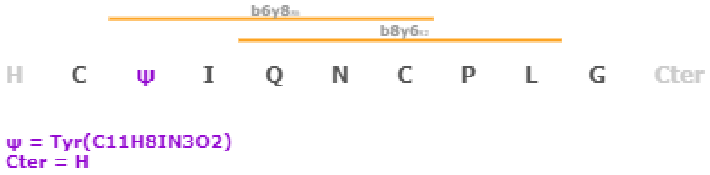

Sequence	Type	MF	m/z	Intensity	Similarity
**YIQNC**	b6y8	C38H48IN10O10S(+1)	901.1868	1.04	86%
QNCPL	b8y6	C23H38N7O7S(+1)	596.2497	0.31	82%

HPLC-UV chromatogram (214 nm) of a 2.00 mM solution of Oxytocin (**1p**) in 100 mM Tris buffer pH 9.0 (with EBX):

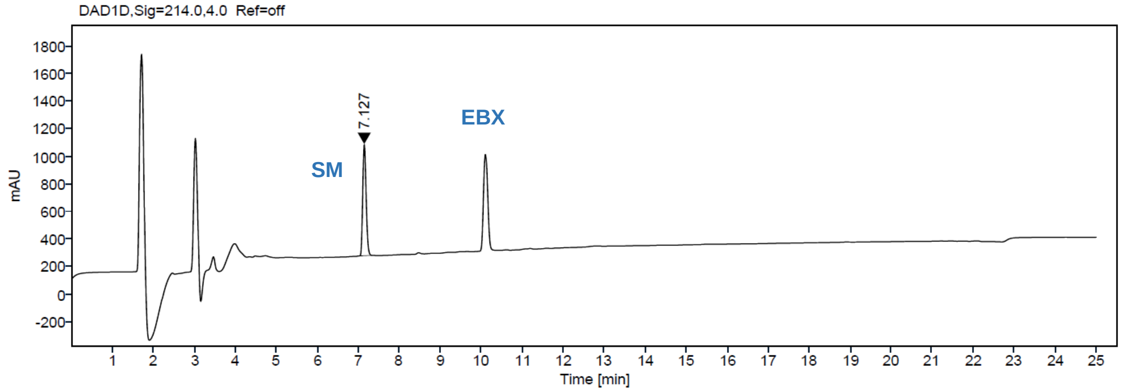

HPLC-UV chromatogram (214 nm) of crude of the reaction:

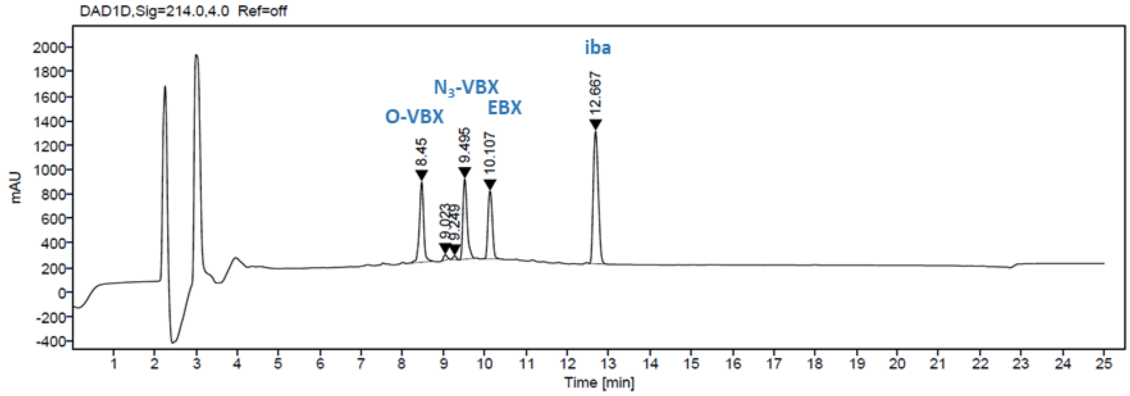

HPLC-MS chromatogram of crude of the reaction:

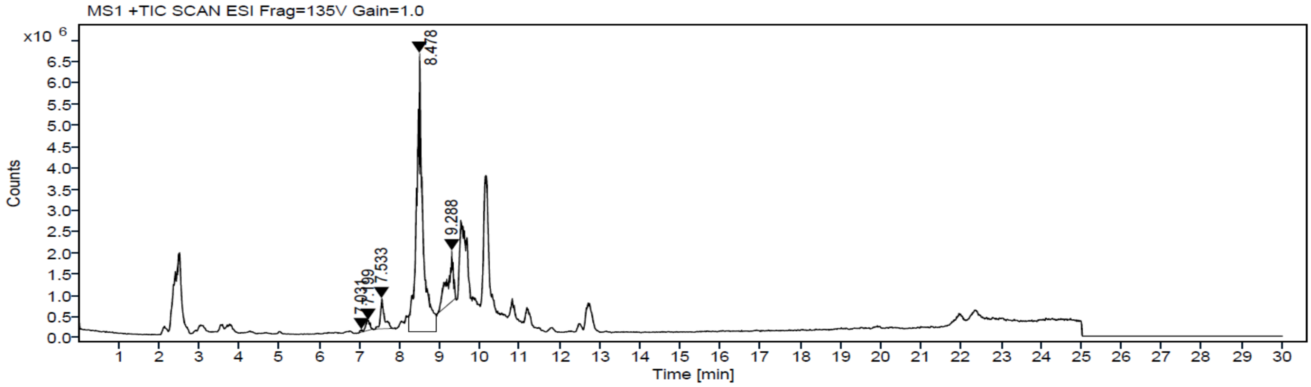

Yield based on HPLC-UV at 214 nm: 84%

Yield based on HPLC-MS: 72%

O-VBX of HIV-1 tat (3q)

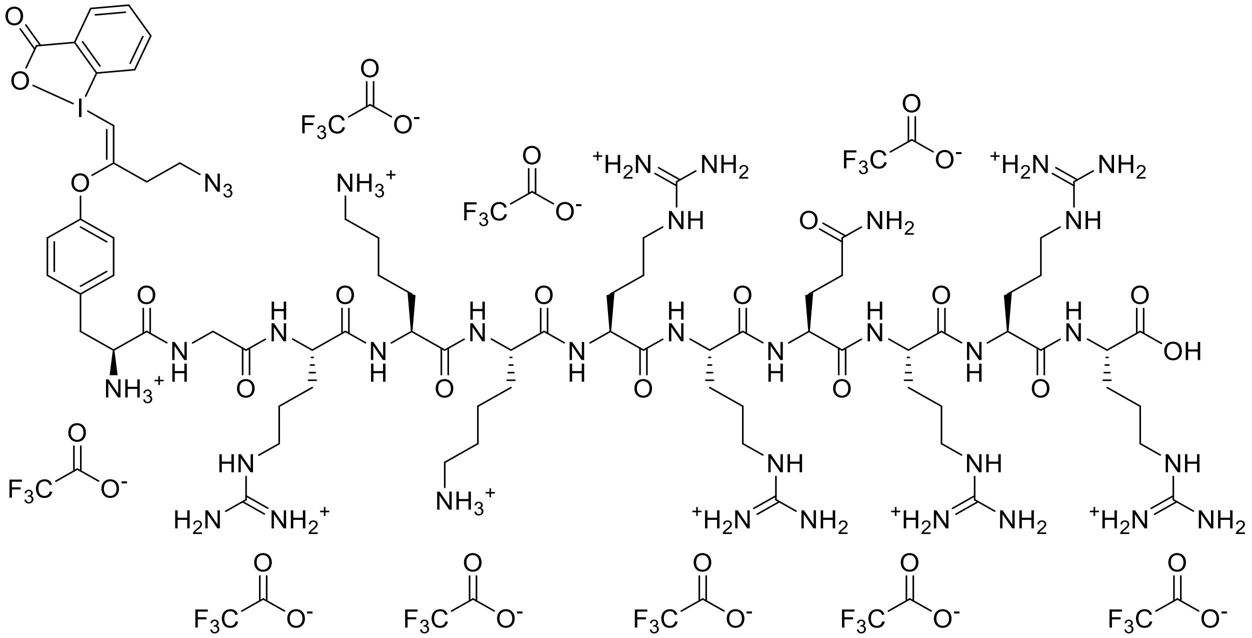

Starting from HIV-1 tat (**1q**) (1.00 mg, 0.387 μmol), O-VBX (**3q**) was obtained in > 90% yield (retention time 4.5 min).

HRMS (nanochip-ESI/LTQ-Orbitrap) m/z: $[M + H_3]^{+3}$ Calcd for $C_{75}H_{129}IN_{35}O_{16}^{+3}$ 634.3128; Found 634.3118.

MS/MS characterization:

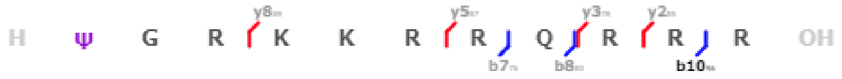

ψ = Tyr(C11H8IN3O2)

Sequence	Type	MF	m/z	Intensity	Similarity
**YGRKKRRQRR**	b10	C69H113IN31O14(+1)	863.9098	5.29	96%
RQRRR	y5	C29H59N18O7(+1)	386.2441	1.69	87%
KKRRQRRR	y8	C47H95N26O10(+1)	592.3896	0.93	89%
**YGRKKRRQRR**	b10	C69H113IN31O14(+1)	576.2756	0.73	95%
RR	y2	C12H27N8O3(+1)	331.2201	0.68	85%
RRR	y3	C18H39N12O4(+1)	487.3212	0.64	78%
**YGRKKRR**	b7	C52H81IN21O10(+1)	643.7794	0.6	76%
**YGRKKRRQ**	b8	C57H89IN23O12(+1)	707.8087	0.54	80%

HPLC-MS chromatogram of a 2.00 mM solution of HIV-1 tat (**1q**) in 100 mM Tris buffer pH 9.0:

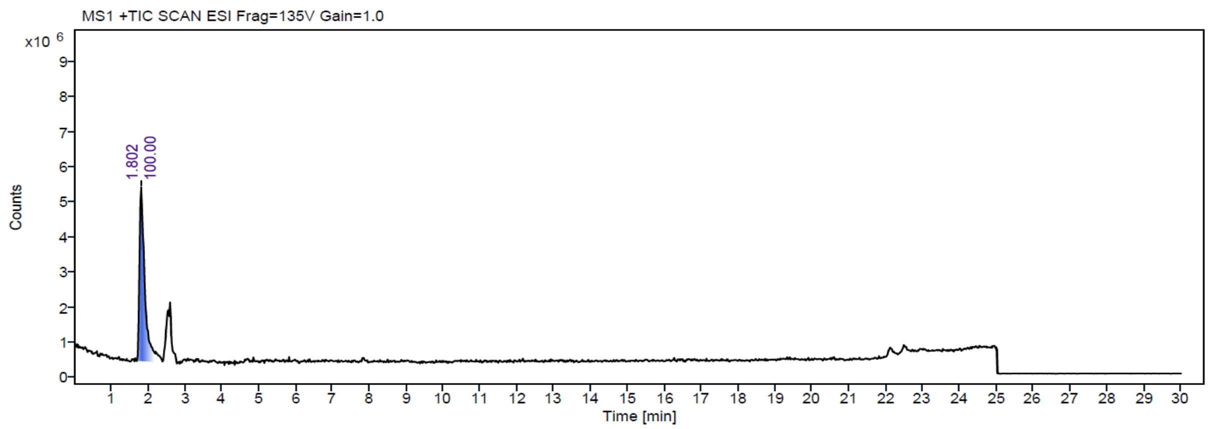

HPLC-UV chromatogram (214 nm) of crude of the reaction:

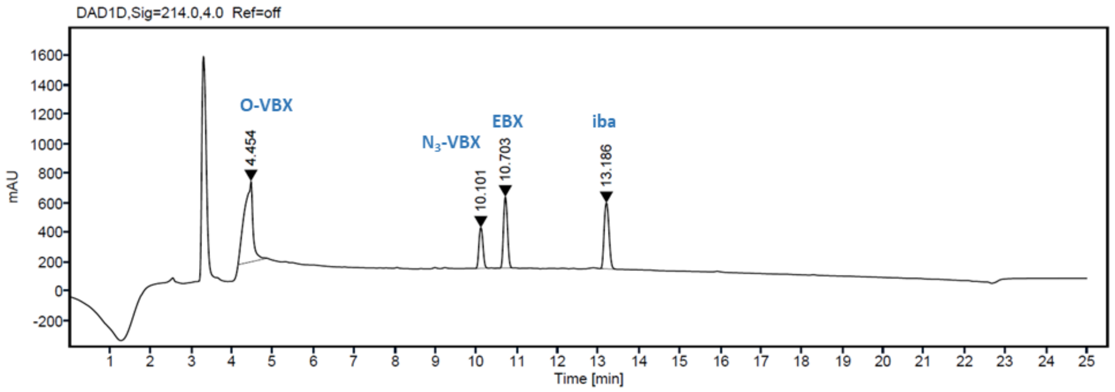

HPLC-MS chromatogram of crude of the reaction:

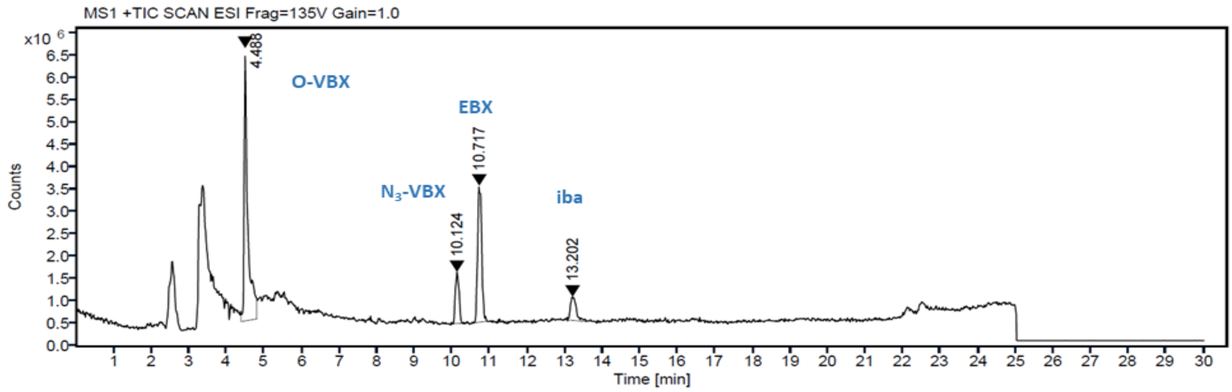

Yield based on HPLC-MS: > 90%

O-VBX of β-Casomorphin (3r)

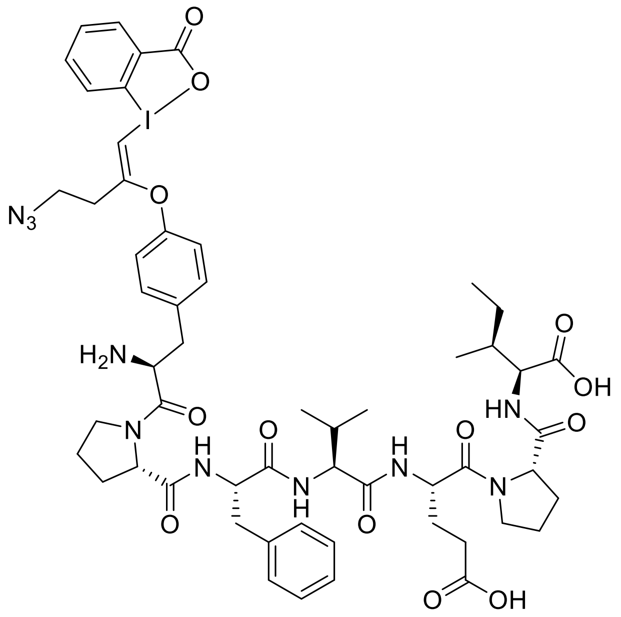

Starting from β-Casomorphin (**1r**) (0.864 mg, 1.00 μmol), O-VBX (**3r**) was obtained in 80% yield (retention time 9.6 min).

HRMS (nanochip-ESI/LTQ-Orbitrap) m/z: $[M + H]^+$ Calcd for $C_{55}H_{70}IN_{10}O_{13}^+$ 1205.4163; Found 1205.4185.

MS/MS characterization:

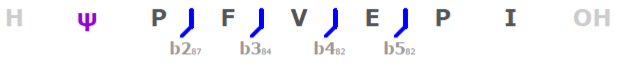

ψ = Tyr(C11H8IN3O2)

Sequence	Type	MF	m/z	Intensity	Similarity
YP	b2	C25H25IN5O5(+1)	602.0889	97.96	87%
YPFVE	b5	C44H50IN8O10(+1)	977.2679	27.41	82%
YPFVE	b5	C44H48IN8O9(+1)	959.2574	9.65	82%
YPFV	b4	C39H43IN7O7(+1)	848.2256	5.42	82%
YPF	b3	C34H34IN6O6(+1)	749.1572	1.66	84%

HPLC-UV chromatogram (214 nm) of a 2.00 mM solution of β-Casomorphin (**1r**) in 100 mM Tris buffer pH 9.0:

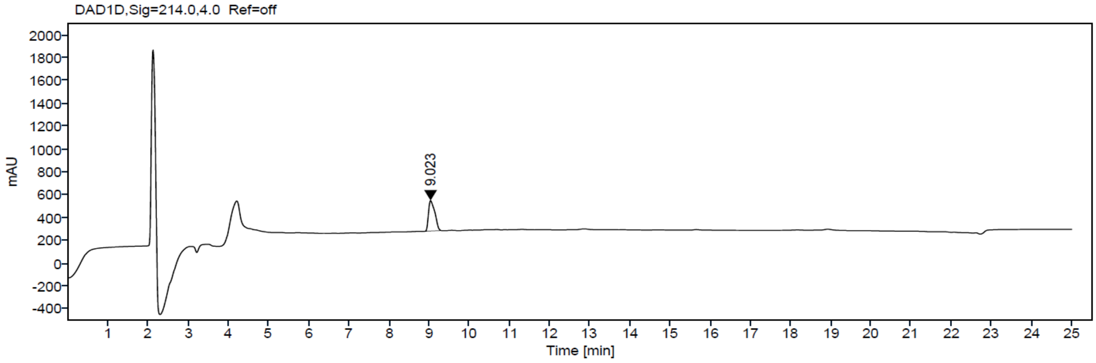

HPLC-UV chromatogram (214 nm) of crude of the reaction:

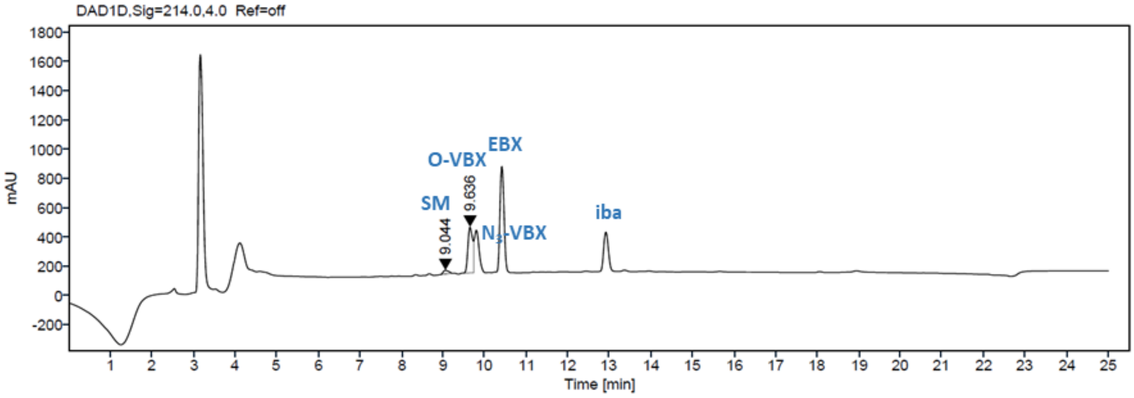

HPLC-UV chromatogram (214 nm) of crude of the reaction with different gradient:

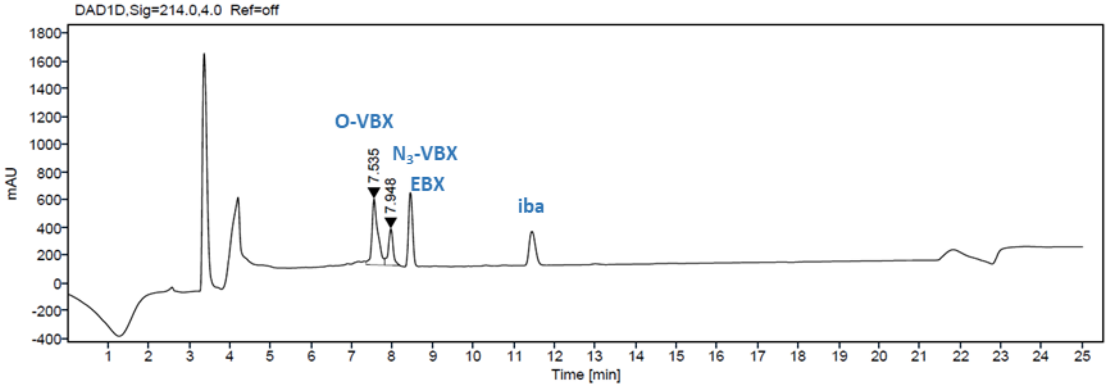

HPLC-MS chromatogram of crude of the reaction with different gradient:

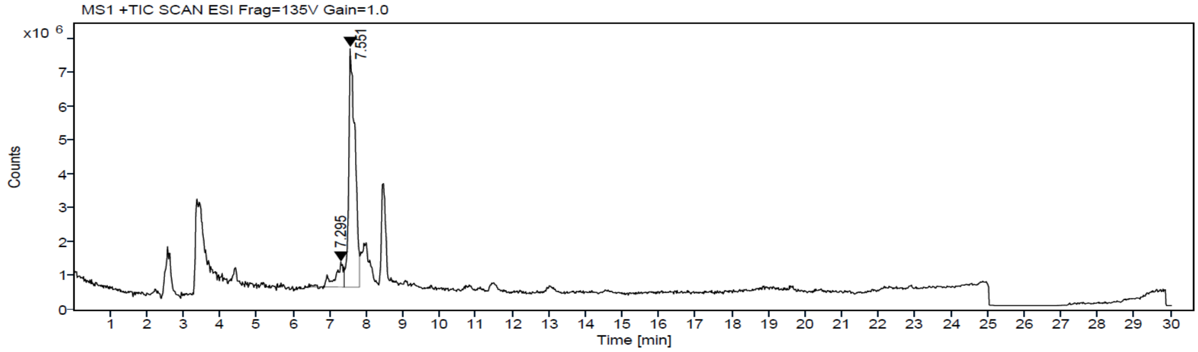

Yield based on HPLC-UV at 214 nm: 89%
Yield based on HPLC-MS: 80%

O-VBX of Angiotensin (3s)

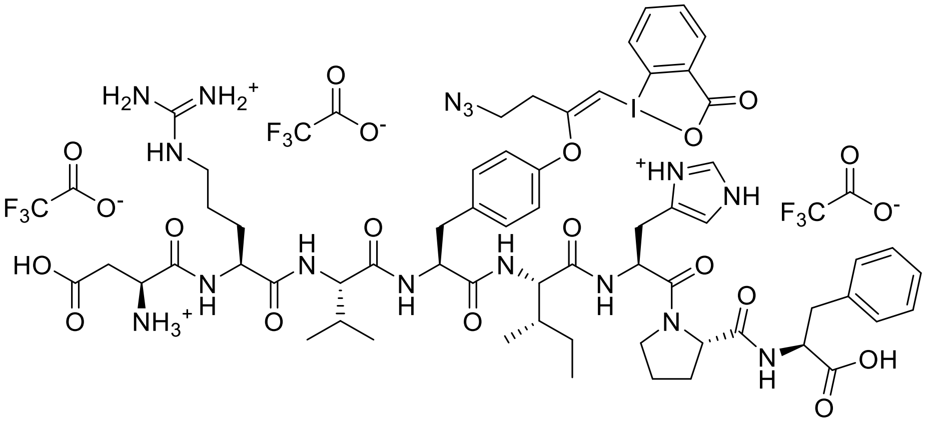

Starting from Angiotensin (**1s**) (1.39 mg, 1.00 µmol), O-VBX (**3s**) was obtained in 55% yield (retention time 8.5 min).

HRMS (nanochip-ESI/LTQ-Orbitrap) m/z: $[M + H_2]^{+2}$ Calcd for $C_{61}H_{81}IN_{16}O_{14}^{+2}$ 694.2576; Found 694.2593.

MS/MS characterization:

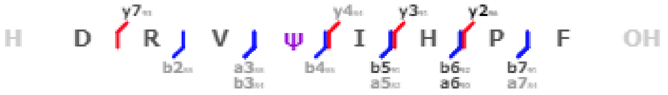

ψ = Tyr(C11H8IN3O2)

Sequence	Type	MF	m/z	Intensity	Similarity
PF	y2	C14H19N2O3(+1)	263.139	54.78	96%
**DRVYIH**	b6	C47H62IN14O11(+1)	1125.3762	28.34	94%
**DRVYIH**	b6	C47H62IN14O11(+1)	563.1917	11.57	91%
**RVYIHPF**	y7	C57H75IN15O11(+1)	636.7441	6.11	93%
**DRVYI**	b5	C41H55IN11O10(+1)	988.3173	3.84	91%
HPF	y3	C20H26N5O4(+1)	400.1979	2.58	91%
**DRVYIHP**	b7	C52H69IN15O12(+1)	611.7181	1.56	91%
**DRVYIH**	a6	C46H62IN14O10(+1)	549.1943	1.06	90%
DR	b2	C10H18N5O4(+1)	272.1353	1.01	88%
DRV	a3	C14H27N6O4(+1)	343.2088	0.78	88%
**DRVY**	b4	C35H44IN10O9(+1)	875.2332	0.66	85%
IHPF	y4	C26H37N6O5(+1)	513.282	0.64	84%
DRV	b3	C15H27N6O5(+1)	371.2037	0.4	84%
**DRVYI**	a5	C40H55IN11O9(+1)	960.3223	0.19	82%
**DRVYIHP**	a7	C51H69IN15O11(+1)	597.7206	0.04	84%

HPLC-UV chromatogram (214 nm) of a 2.00 mM solution of Angiotensin (**1s**) in 100 mM Tris buffer pH 9.0:

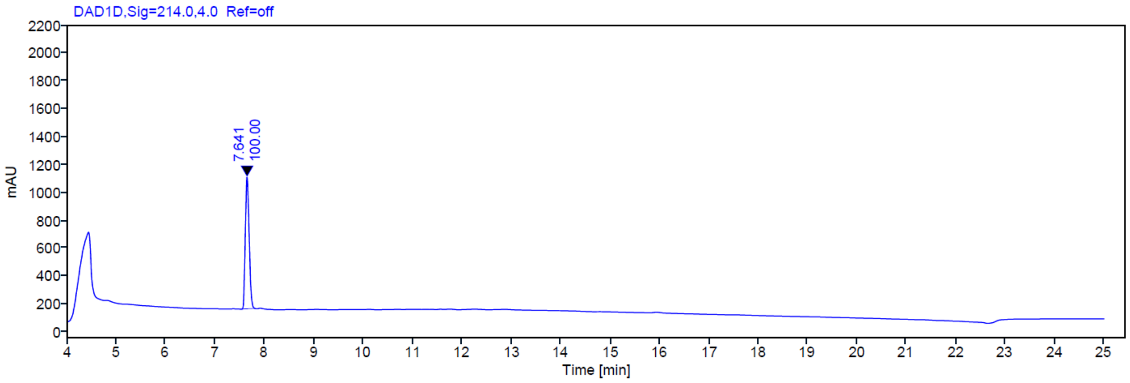

HPLC-UV chromatogram (214 nm) of crude of the reaction:

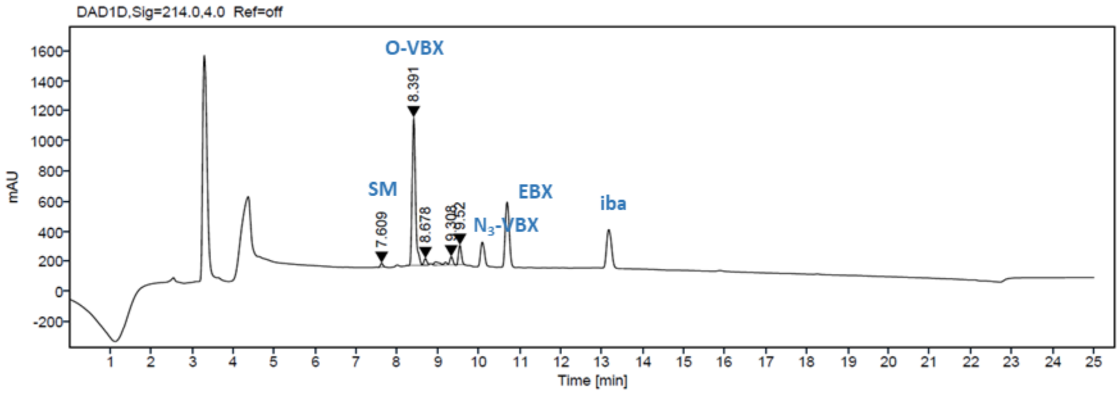

HPLC-MS chromatogram of crude of the reaction:

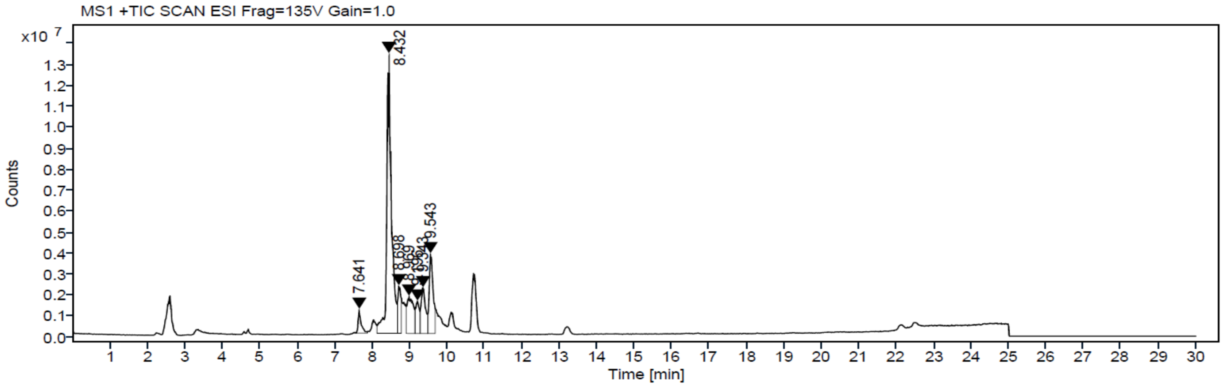

Yield based on HPLC-UV at 214 nm: 78%

Yield based on HPLC-MS: 55%

O-VBX of Amyloid β-Protein (1-15) (3t)

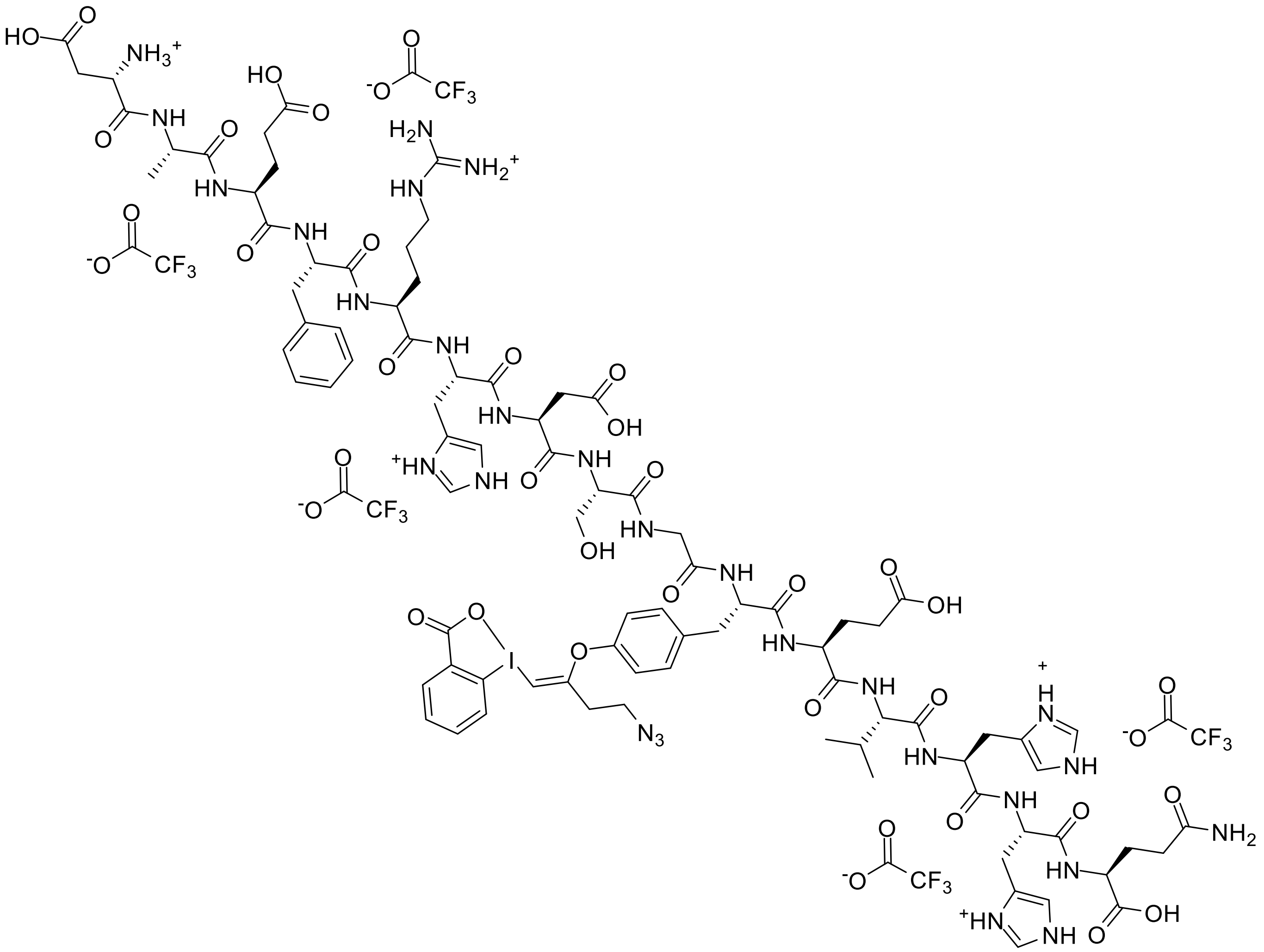

Starting from Amyloid β-Protein (1-15) (**1t**) (1.00 mg, 0.417 μmol), O-VBX (**3t**) was obtained in 61% yield (retention time 6.3 min).

HRMS (nanochip-ESI/LTQ-Orbitrap) m/z: $[M + H_2]^{+2}$ Calcd for $C_{89}H_{117}IN_{28}O_{29}^{+2}$ 1084.3787; Found 1084.3856.

MS/MS characterization:

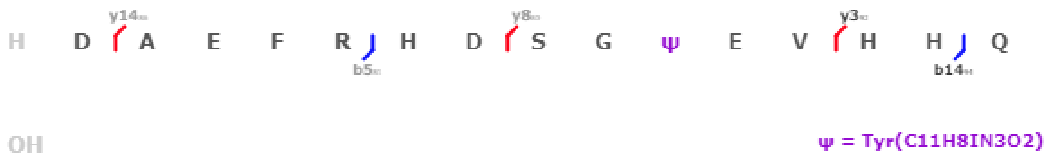

Sequence	Type	MF	m/z	Intensity	Similarity
**SGYEVHHQ**	y8	C52H66IN16O16(+1)	1297.388	6.92	80%
**DAEFRHDSGYEVHH**	b14	C84H106IN26O26(+1)	1011.344	5.21	94%
**AEFRHDSGYEVHHQ**	y14	C85H111IN27O26(+1)	1026.865	4.44	86%
DAEFR	b5	C27H39N8O9(+1)	619.2835	1.88	81%
HHQ	y3	C17H25N8O5(+1)	421.1942	1.56	92%

HPLC-UV chromatogram (214 nm) of a 2.00 mM solution of Amyloid β-Protein (1-15) (**1t**) in 100 mM Tris buffer pH 9.0:

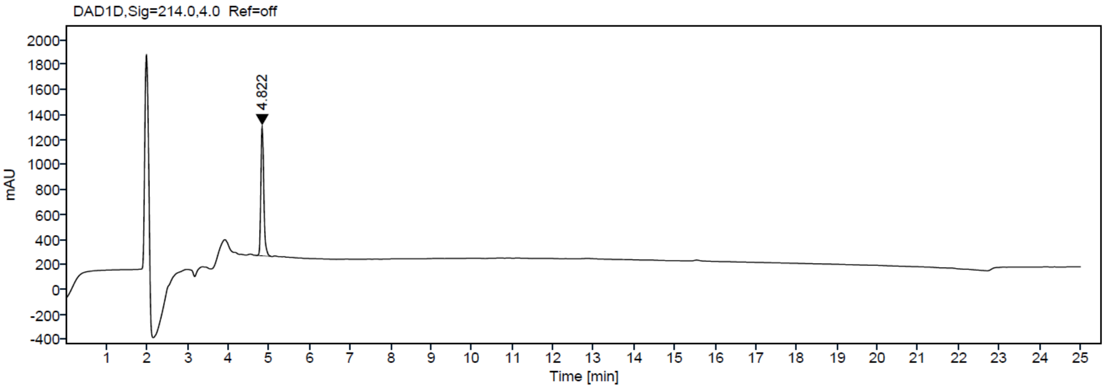

HPLC-UV chromatograms (214 nm) of crude of the reaction:

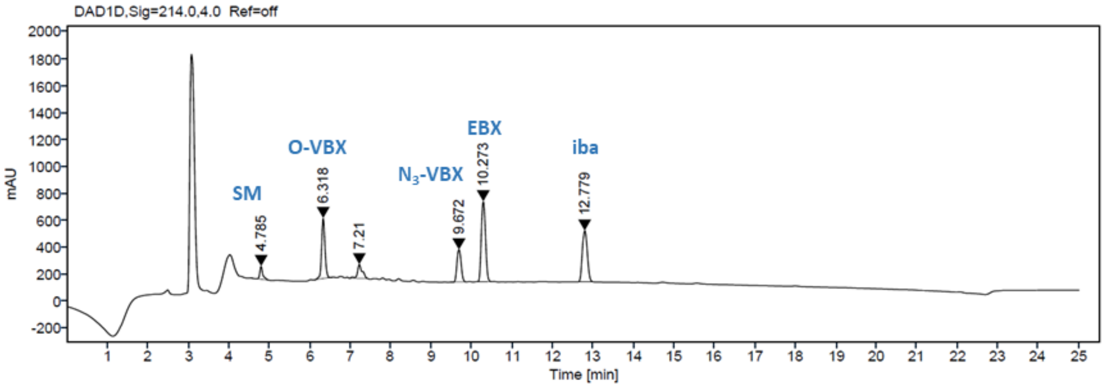

HPLC-MS chromatograms of crude of the reaction:

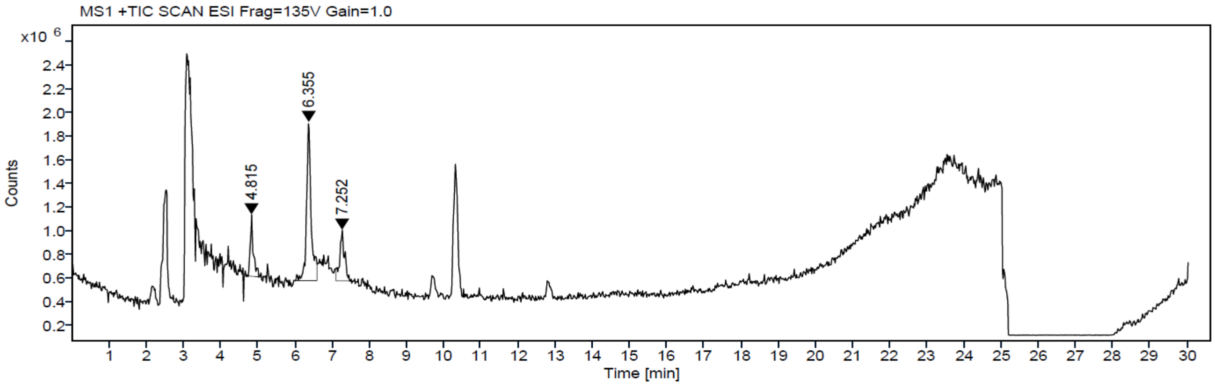

Yield based on HPLC-UV at 214 nm: 64%

Yield based on HPLC-MS: 61%

O-VBX of Amyloid β-Protein (1-24) (3u)

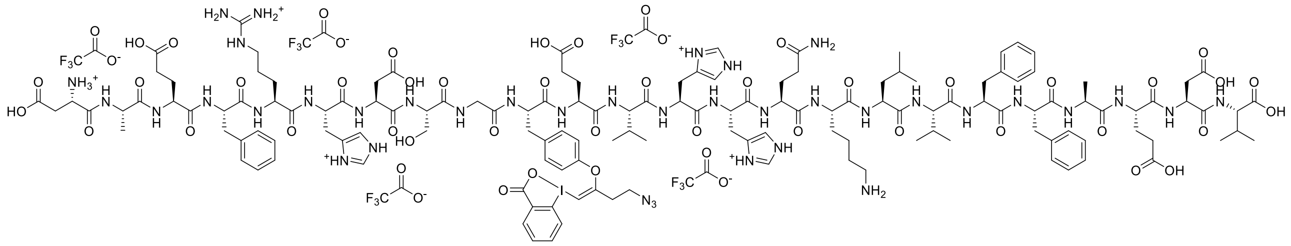

Starting from Amyloid β-Protein (1-24) (**1u**) (0.500 mg, 0.145 µmol), O-VBX (**3u**) was obtained in 40% yield (retention time 8.2 min).

HRMS (nanochip-ESI/LTQ-Orbitrap) m/z: $[M + H_3]^{+3}$ Calcd for $C_{141}H_{194}IN_{38}O_{42}^{+3}$ 1072.7747; Found 1072.7733.

MS/MS characterization:

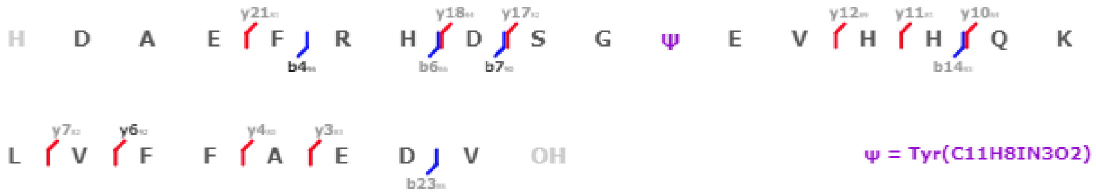

Sequence	Type	MF	m/z	Intensity	Similarity
DAEFRHD	b7	C37H51N12O13(+1)	871.3693	27.32	90%
**DAEFRHDSGYEVHHQKLVFFAED**	b23	C136H181IN37O40(+1)	1033.748	14.09	85%
**SGYEVHHQKLVFFAEDV**	y17	C104H142IN26O29(+1)	1173.477	8.33	82%
HQKLVFFAEDV	y11	C63H94N15O17(+1)	1332.695	6.09	81%
FFAEDV	y6	C35H47N6O11(+1)	727.3297	5.82	92%
DAEFRH	b6	C33H46N11O10(+1)	756.3424	5.26	86%
**FRHDSGYEVHHQKLVFFAEDV**	y21	C129H175IN35O35(+1)	967.7392	2.01	81%
QKLVFFAEDV	y10	C57H87N12O16(+1)	1195.636	2.01	84%
HHQKLVFFAEDV	y12	C69H101N18O18(+1)	735.3804	1.49	89%
**DAEFRHDSGYEVHH**	b14	C84H106IN26O26(+1)	1011.344	1.36	83%
VFFAEDV	y7	C40H56N7O12(+1)	826.3981	1.26	82%
DAEF	b4	C21H27N4O8(+1)	463.1823	1.01	96%
AEDV	y4	C17H29N4O9(+1)	433.1929	0.77	80%
EDV	y3	C14H24N3O8(+1)	362.1558	0.75	83%
**DSGYEVHHQKLVFFAEDV**	y18	C108H147IN27O32(+1)	1230.991	0.71	84%

HPLC-UV chromatogram (214 nm) of a 2.00 mM solution of Amyloid β-Protein (1-24) (**1u**) in 100 mM Tris buffer pH 9.0:

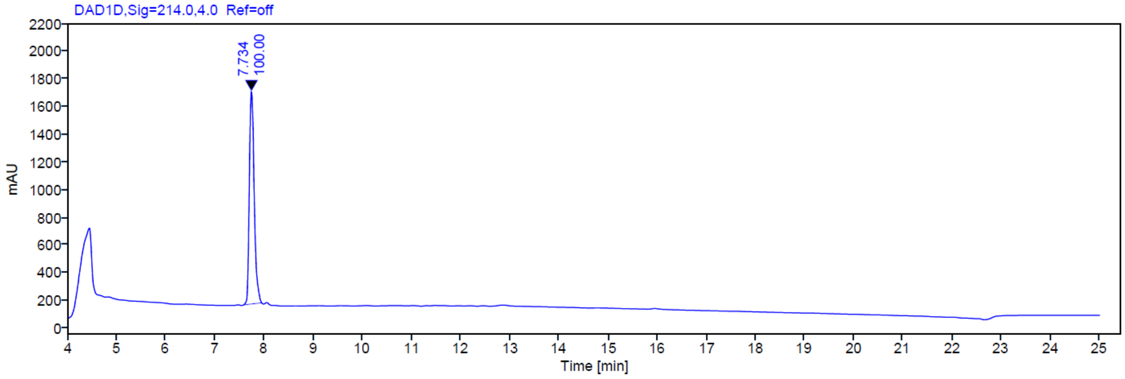

HPLC-UV chromatograms (214 nm) of crude of the reaction:

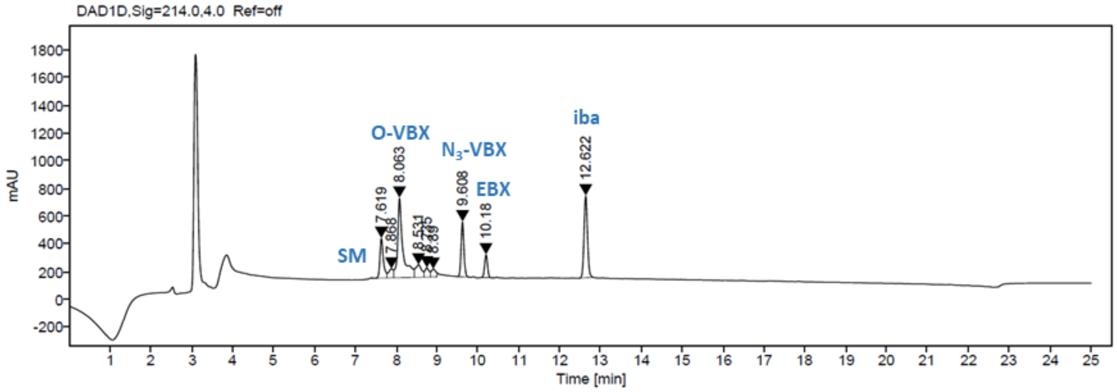

HPLC-MS chromatograms of crude of the reaction:

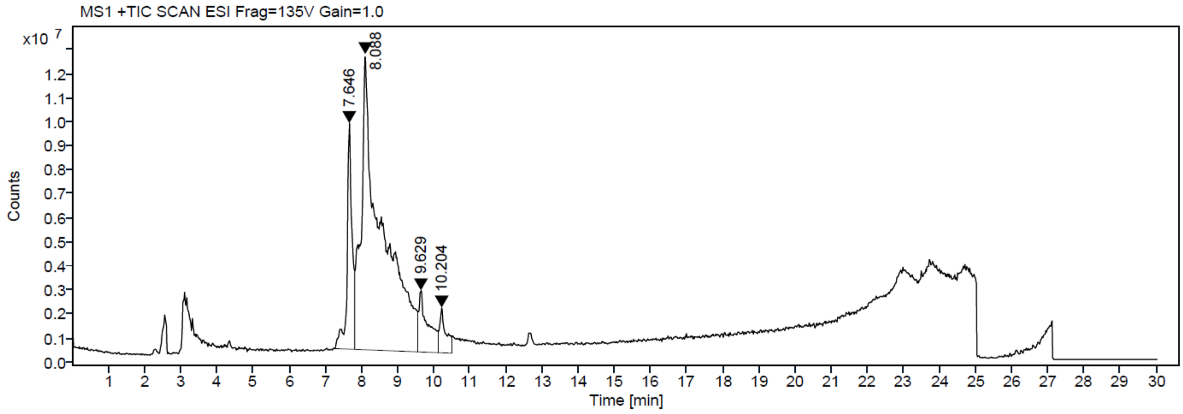

Yield based on HPLC-UV at 214 nm: 41%

Yield based on HPLC-MS: 40%

O-VBX of Cyclic RGD (3v)

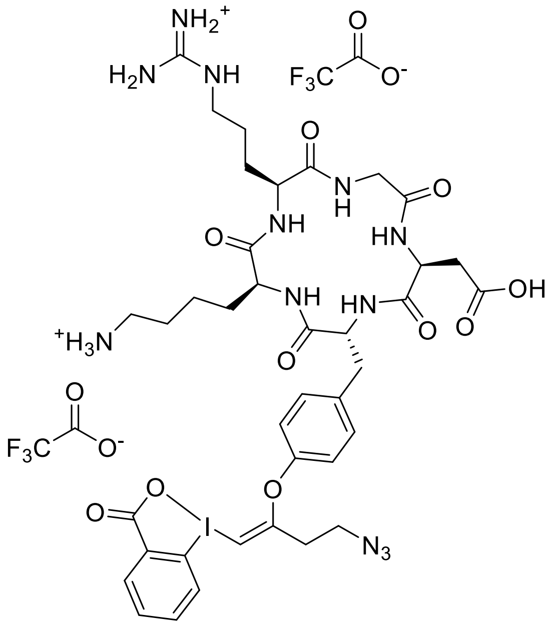

Starting from Cyclic RGD (**1v**) (0.848 mg, 1.00 µmol), O-VBX (**3v**) was obtained in 76% yield (retention time 6.5 min).

HRMS (nanochip-ESI/LTQ-Orbitrap) m/z: [M + H]$^+$ Calcd for $C_{38}H_{50}IN_{12}O_{10}^+$ 961.2812; Found 961.2826.

MS/MS characterization: *The lack of processing tools to assign fragment ions for such cyclic modified peptide did not allow us to confirm its structure based on the fragmentation spectra obtained.*

HPLC-UV chromatogram (214 nm) of a 2.00 mM solution of Cyclic RGD (**1v**) in 100 mM Tris buffer pH 9.0 (with EBX):

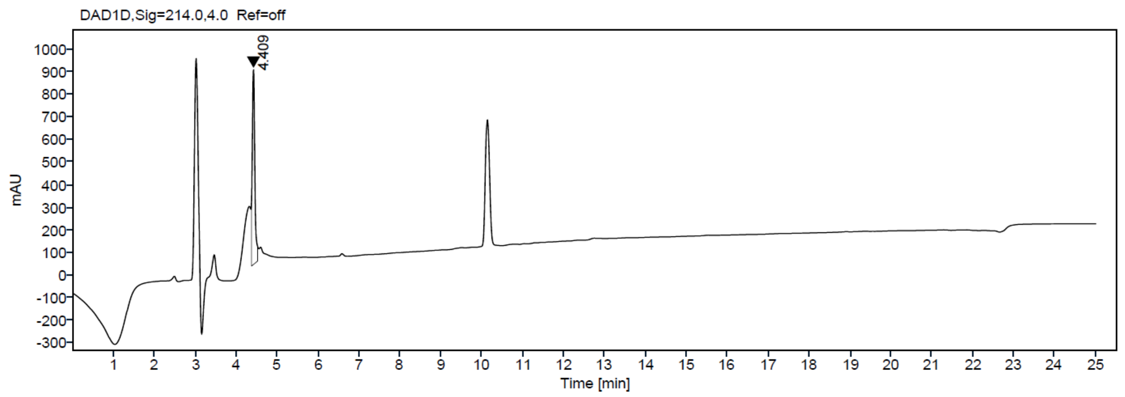

HPLC-MS chromatogram of a 2.00 mM solution of Cyclic RGD (**1v**) in 100 mM Tris buffer pH 9.0:

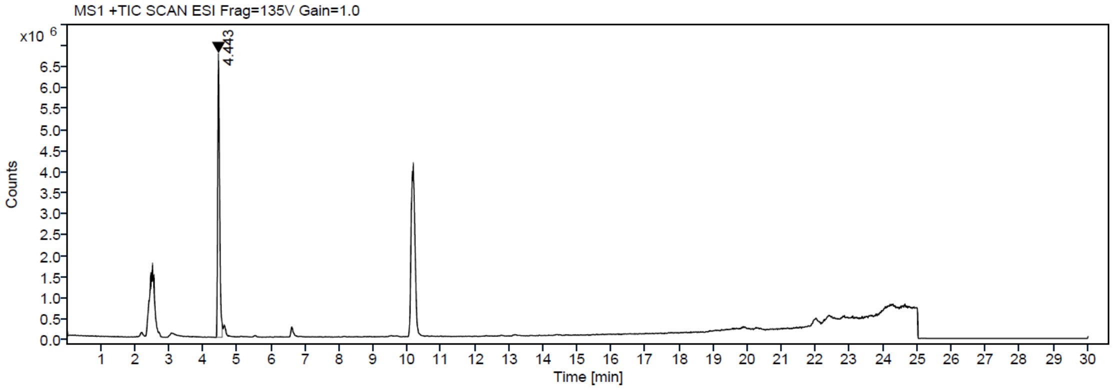

HPLC-UV chromatograms (214 nm) of crude of the reaction:

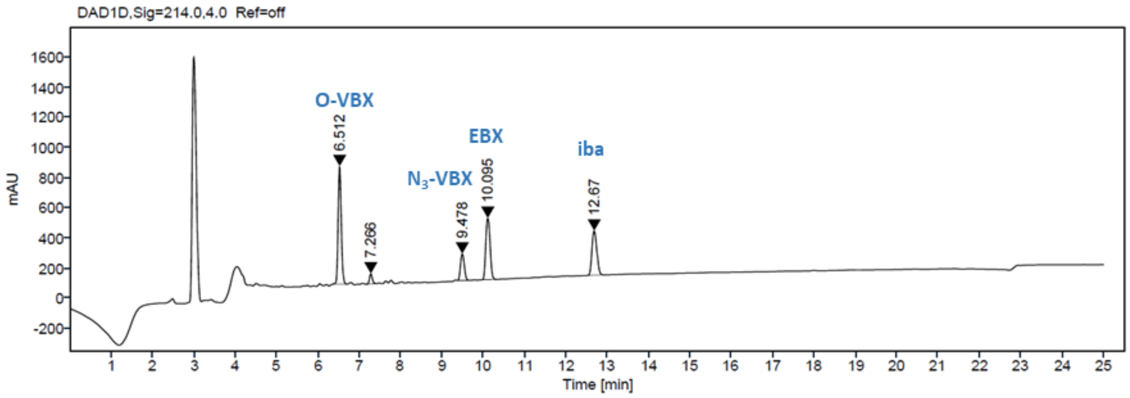

HPLC-MS chromatograms of crude of the reaction:

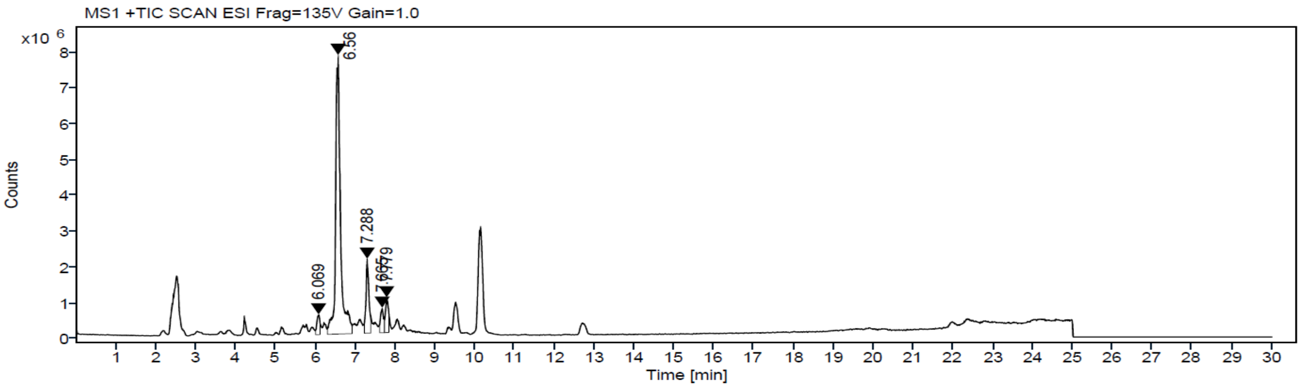

Yield based on HPLC-UV at 214 nm: 88%

Yield based on HPLC-MS: 76%

O-VBX of Jagaricin (3w)

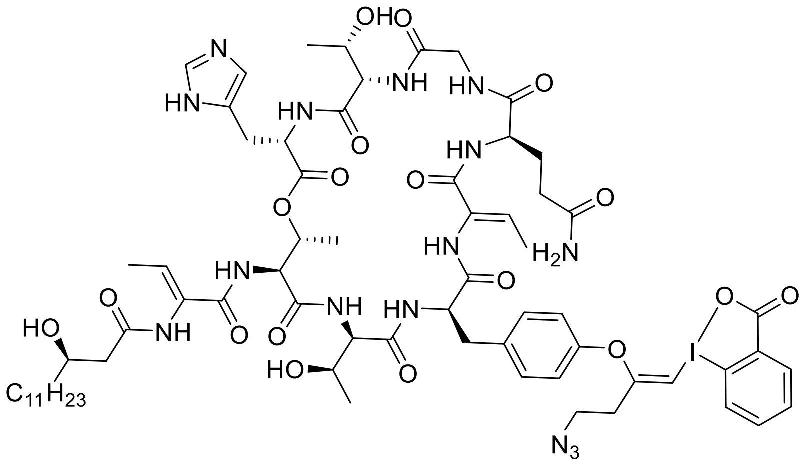

Starting from Jaricin (**3w**, 8.50 µmol), O-VBX (**3wa**) was obtained in 61% yield based on HPLC-MS (retention time 13.3 min) and 30% isolated yield (4.00 mg, 2.60 µmol).

HRMS (HESI/LTQ-Orbitrap) m/z: $[M + H]^+$ Calcd for $C_{67}H_{93}IN_{15}O_{18}^+$ 1522.5862; Found 1522.5825.

MS/MS characterization: *The lack of processing tools to assign fragment ions for such cyclic modified peptide did not allow us to confirm its structure based on the fragmentation spectra obtained.*

HPLC-UV chromatogram (214 nm) of crude of the reaction:

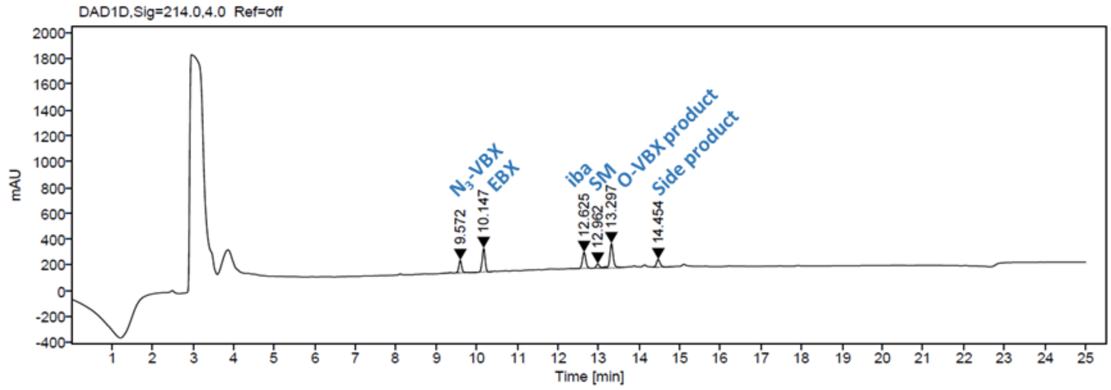

HPLC-MS chromatogram of crude of the reaction:

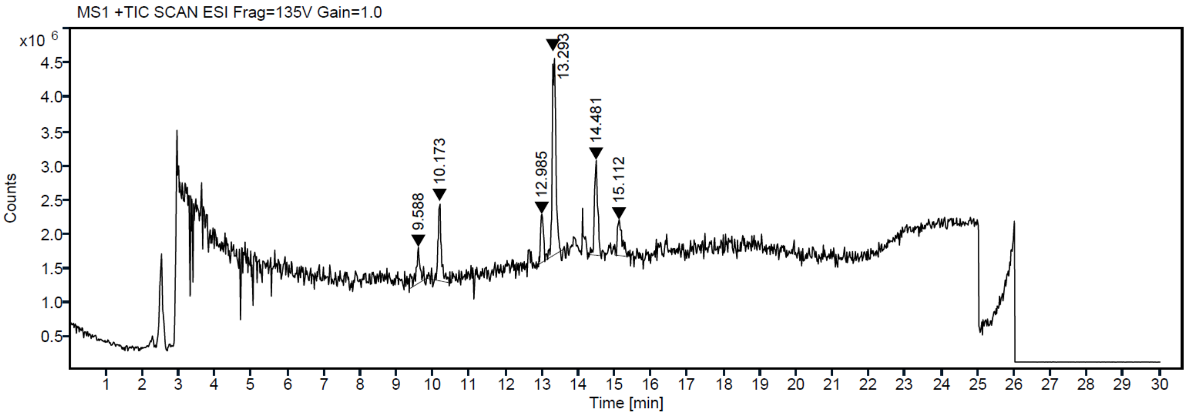

7. Protein bioconjugation

Protein materials and their sequences used in this study:

Ubiquitin: recombinant human protein (Bio-techne, U-100H)
MQIFVKTLTGKTITLEVEPSDTIENVKAKIQDKEGIPPDQQRLIFAGKQLEDGRTLSDYNIQKE
STLHLVLRLRGG

Myoglobin: from equine skeletal muscle (Sigma-Aldrich, M0630)
GLSDGEWQQVLNVWGKVEADIAGHGQEVLIRLFTGHPETLEKFDKFKHLKTEAEMKASEDL
KKHGTVVLTALGGILKKKGHHEAELKPLAQSHATKHKIPIKYLEFISDAIIHVLHSKHPGDFGA
DAQGAMTKALELFRNDIAAKYKELGFQG

Streptavidin: recombinant protein (Lubio Science, OPPA02060)
MAEAGITGTWYNQLGSTFIVTAGADGALTGTYESAVGNAESRYVLTGRYDSAPATDGSGTA
LGWTVAWKNNYRNAHSATTWSGQYVGGAEARINTQWLLTSGTTEANAWKSTLVGHDTFT
KVKPSAAS

Trastuzumab (Herceptin): recombinant antibody (Lubio Science, HY-P9907); $C_{6460}H_{9972}N_{1724}O_{2014}S_{44}$
Light chain: DIQMTQSPSSLSASVGDRVTITCRASQDVNTAVAWYQQKPGKAPKLLIYSASF
LYSGVPSRFSGSRSGTDFTLTISSLQPEDFATYYCQQHYTTPPTFGQGTKVEIKRTVAAPSV
FIFPPSDEQLKSGTASVVCLLNNFYPREAKVQWKVDNALQSGNSQESVTEQDSKDSTYSLS
STLTLSKADYEKHKVYACEVTHQGLSSPVTKSFNRGEC
Heavy chain: EVQLVESGGGLVQPGGSLRLSCAASGFNIKDTYIHWVRQAPGKGLEWVA
RIYPTNGYTRYADSVKGRFTISADTSKNTAYLQMNSLRAEDTAVYYCSRWGGDGFYAMDY
WGQGTLVTVSSASTKGPSVFPLAPSSKSTSGGTAALGCLVKDYFPEPVTVSWNSGALTSG
VHTFPAVLQSSGLYSLSSVVTVPSSSLGTQTYICNVNHKPSNTKVDKKVEPKSCDKTHTCPP
CPAPELLGGPSVFLFPPKPKDTLMISRTPEVTCVVVDVSHEDPEVKFNWYVDGVEVHNAKT
KPREEQYNSTYRVVSVLTVLHQDWLNGKEYKCKVSNKALPAPIEKTISKAKGQPREPQVYT
LPPSREEMTKNQVSLTCLVKGFYPSDIAVEWESNGQPENNYKTTPPVLDSDGSFFLYSKLT
VDKSRWQQGNVFSCSVMHEALHNHYTQKSLSLSPGK

Monoisotopic and average masses of elements used to calculate carbohydrate structures based on elemental composition of glycan species contributing to major trastuzumab glycoforms:[22]

Monosaccharides nomenclature											
GlcNac, Man, Gal, Fuc			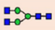	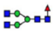	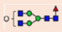	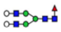	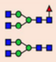	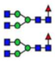	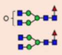	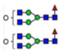	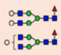
Element	Monoisotopic mass	Average mass	G0	G0F	G1F	G2F	G0/G0F	G0F/G0F	G0F/G1F	G1F/G1F	G1F/G2F
			Elemental Composition								
C	12	12.01079	50	56	62	68	6554	6560	6566	6572	6578
H	1.007825032	1.007968	82	92	102	112	10122	10132	10142	10152	10162
N	14.00307401	14.00669	4	4	4	4	1728	1728	1728	1728	1728
O	15.99491462	15.99937	35	39	44	49	2086	2090	2095	2100	2102
S	31.97207069	32.0639	0	0	0	0	0	0	0	0	0
Monoisotopic mass			1298.47596	1444.533869	1606.58669	1768.63952	2743.00983	2889.067738	3051.120562	3213.1733385	3375.226209
Average mass			1299.19759	1445.339486	1607.48076	1769.62203	2744.537072	2890.678972	3052.820242	3214.961512	3377.102782

[22] The numbers were taken from ThermoFisher website.

General procedure for Tyr-selective bioconjugation to proteins:

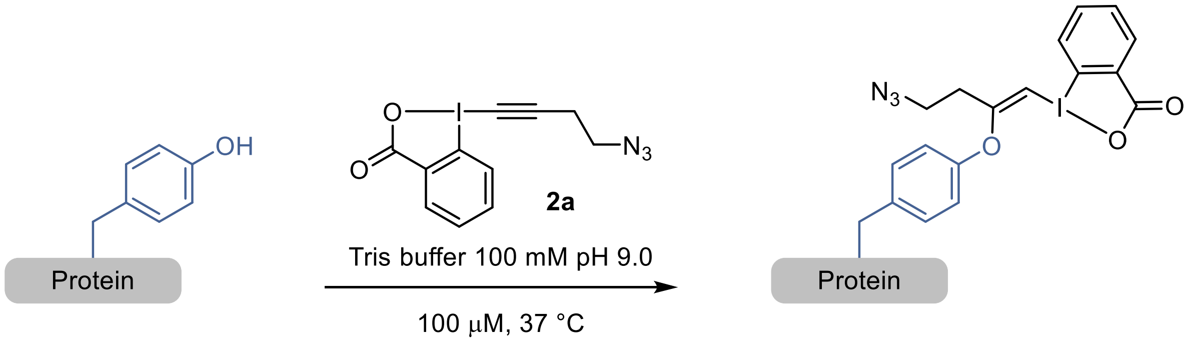

Procedure A: In a 0.5 mL Eppendorf Safe-Lock microcentrifuge tube, Protein (10.0 μL of 1.00 mM stock solution in 100 mM pH 9.0 Tris buffer, 10.0 nmol, 1.00 equiv, 100 μM final concentration) was diluted in Tris buffer (100 mM Tris, pH 9.0, 89.6 μL) or denaturing Tris buffer (6.00 M GdmHCl, 100 mM Tris, pH 9.0, 89.6 μL). Then a 300 mM solution of EBX **2a** in DMSO (0.400 μL, 10.0 nmol, 10 equiv.) was added in one portion and the resulting mixture was shook at 37 °C for 72 h. No effort was made to exclude oxygen.

Procedure B: In a 0.5 mL Eppendorf Safe-Lock microcentrifuge tube, Protein (10.0 μL of 1.00 mM stock solution in 100 mM pH 9.0 Tris buffer, 10.0 nmol, 1.00 equiv, 100 μM final concentration) was diluted in Tris buffer (100 mM Tris, pH 9.0, 88.3 μL) or denaturing Tris buffer (6.00 M GdmHCl, 100 mM Tris, pH 9.0, 88.3 μL). Then a 300 mM solution of EBX **2a** in DMSO (1.70 μL, 50.0 nmol, 50 equiv.) was added in one portion and the resulting mixture was shook at 37 °C for 24 h. No effort was made to exclude oxygen.

The reaction crude was analyzed by high resolution mass spectrometry. The yield was determined using deconvoluted MS area. Peaks with relative abundance under 5% were considered as background signals and were not taken in account.

$$\text{Yield (\%)} = [A(\text{modified protein}) / \sum A(\text{protein components})] \times 100.$$

Procedure trastuzumab: In a 0.5 mL Eppendorf Safe-Lock microcentrifuge tube, Trastuzumab (10.0 μL of 100 μM stock solution in 100 mM pH 9.0 Tris buffer, 1.00 nmol, 1.00 equiv, 10 μM final concentration) was diluted in Tris buffer (100 mM Tris, pH 9.0, 90.0 μL). Then a 3 mM solution of EBX **2a** in 100 mM Tris (pH 9.0) - 1% DMSO (2 μL, 5.00 nmol, 5.00 equiv.) was added in one portion and the resulting mixture was shook at 37 °C for 4 h. No effort was made to exclude oxygen.

<u>Procedure for LC-MS/MS analysis:</u>

Intact protein mass spectrometry analysis

After reaction, samples were diluted to a final protein concentration of 5 uM with milliQ water containing 0.1% formic acid and were injected into an Acquity UPLC Protein column BEH C4 VanGuard (300 Å, 1.7 µm, 2.1 x 5 mm, Waters, Milford, MA, U.S.A.) using a Vanquish analytical LC system (Thermo Fisher Scientific, Germany) coupled to an OptaMax NG ion source (Thermo Fisher Scientific, Bremen, Germany). The sample desalting was performed with a flow rate of 400 µl/min by applying a gradient of solvent B from 15 to 60 % in 2.5 min, followed by column washing and re-equilibration steps. Solvent A was composed of milliQ water with 0.1 % formic acid, while solvent B consisted of acetonitrile with 0.1 % formic acid.

Eluting proteoforms were analyzed on a Exploris 240 Orbitrap-FT-MS benchtop instrument (Thermo Fisher Scientific, Bremen, Germany) using Intact Protein mode with low pressure settings, positive polarity, standard AGC, maximum injection time (IT) set to auto, 240'000 resolution at 200 *m/z* and averaging 5 microscans. Intact mass measurement data were analyzed with BioPharma Finder 4.1 software (Thermo Fisher Scientific, Sunnyvale, CA, U.S.A.) using a Xtract algorithm with 90% fit factor.

Top-down mass spectrometry analysis

To localize the modification sites, samples were submitted to top-down mass spectrometry analysis using higher energy collision induced dissociation (HCD) or collision induced dissociation (CID) fragmentation techniques.

For HCD-based top-down MS experiments, samples were diluted to a final protein concentration of 5 uM with milliQ water containing 0.1% formic acid and were injected into an Acquity UPLC Protein column BEH C4 VanGuard (300 Å, 1.7 µm, 2.1 x 5 mm, Waters, Milford, MA, U.S.A.) using a Vanquish analytical LC system (Thermo Fisher Scientific, Germany) coupled to an OptaMax NG ion source (Thermo Fisher Scientific, Bremen, Germany). The sample desalting was performed with a flow rate of 400 µl/min by applying a gradient of solvent B from 15 to 60 % in 2.5 min, followed by column washing and re-equilibration steps. Solvent A was composed of milliQ water with 0.1 % formic acid, while solvent B consisted of acetonitrile with 0.1 % formic acid.

Eluting proteoforms were analyzed on a Orbitrap Exploris 240 FT-MS benchtop instrument (Thermo Fisher Scientific, Bremen, Germany) using Intact Protein mode with low pressure settings, positive polarity and targeted MS/MS approach with ion multiplexing. For each protein sample 8 precursor ions corresponding to different charge states of the same proteoform carrying one EBX modification were input into the targeted inclusion mass list. Isolation window was set to 1.5 *m/z*, AGC target as standard, maximum IT set as Auto, 240'000 resolution at 200 *m/z* and averaging 10 microscans. Three different values (between 25-50 %) of normalized collision energy (NCE) for HCD fragmentation were set for each sample.

CID-based top-down MS analyses were performed on an LTQ Orbitrap Elite ETD FTMS (Thermo Fisher Scientific, Bremen, Germany) operated in the positive mode coupled with a chip-based nano-ESI source (TriVersa Nanomate, Advion Biosciences, Ithaca, NY, U.S.A.) controlled by the Chipsoft 8.3.1 software (Advion BioScience). Protein samples were desalted using Zip-Tip C4 pipette Tips (Millipore Corporation, MA, U.S.A) with standard protocol provided by the manufacturer and loaded onto a 96-well plate (Eppendorf, Hamburg, Germany). MS survey scans were acquired with 240'000 resolution at 400 *m/z*. Fragmentation of different charge states was carried out using CID with different normalized collision energies (between 30 to 50). Isolation window was set to 3 *m/z* and 10 microscans were averaged for

each MS/MS spectrum at resolution set to 240’000 at 400 *m/z*. The maximum injection time was set to 1000 ms for both MS and MS/MS, and AGC was set to 1e6 for MS scan and 5e4 for MS/MS, respectively.

Top-down MS data were deconvolved using MASH Explorer software (Ge Group, University of Wisconsin) using eTRASH algorithm. Deconvolution results obtained from different NCE values for both HCD and CID values were combined together to create a fragmentation map with assigned b- and y-fragment ions using ProSight Lite software (Kelleher research group, Northwestern University) with 10 ppm mass accuracy tolerance.

Alternatively, certain MS/MS spectra were exported as txt files and processed using the Apm2S web-based application available at ms.epfl.ch (Lee et al., 2018). The group C11H8IN3O2 was either entered as a variable or fixed modification in the sequence of the protein. A similarity cut-off of 85% was applied to generate fragmentation maps mainly composed of b-, y- and yb internal fragments. The length of internal fragment length was set to a minimum of 5 to a maximum of 20 amino acid residues.

Intact antibody samples were analyzed by size exclusion chromatography (SEC) in the native ESI-FT-MS conditions. The SEC separation was carried out using the column MAbPac SEC-1, 5 um, 300 Å, 4x150 mm (Thermo Fisher Scientific) with Ammonium acetate 50 mM as mobile phase. The flow rate was 0.3 mL/min and run time 7 min.

VBX-Ubiquitin 6:

Deconvoluted mass spectrum:

(a) Procedure A in denaturing Tris buffer, 24% yield

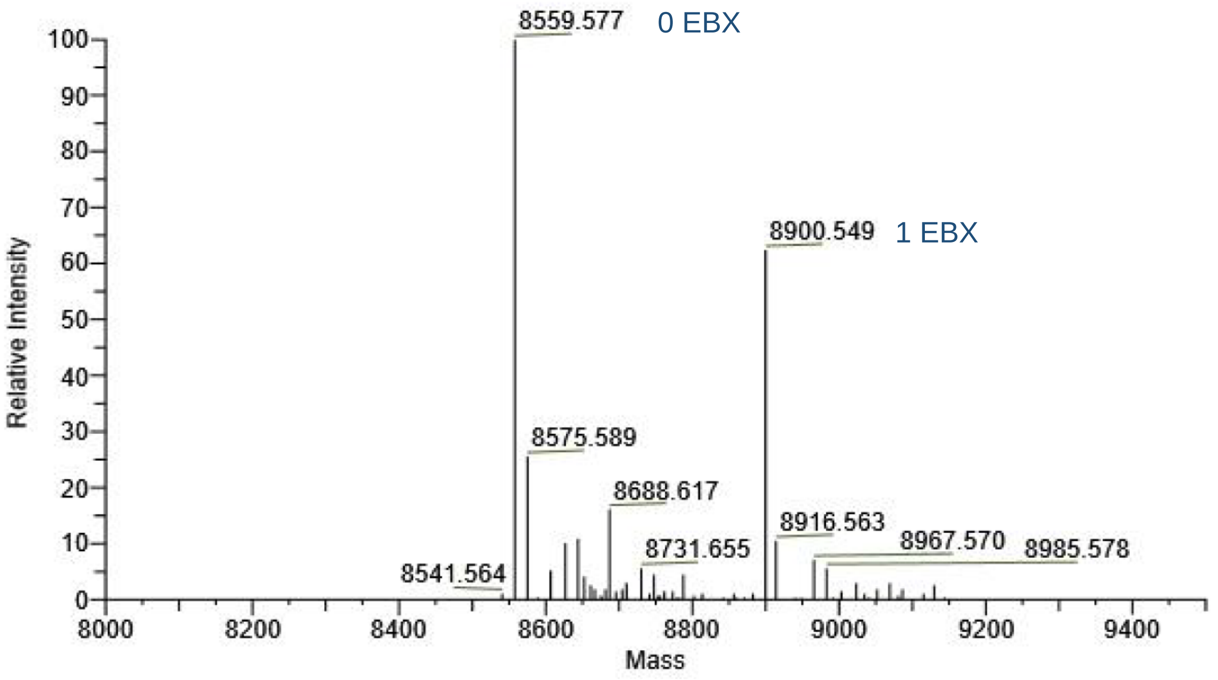

(b) Procedure B in denaturing Tris buffer, 23% yield

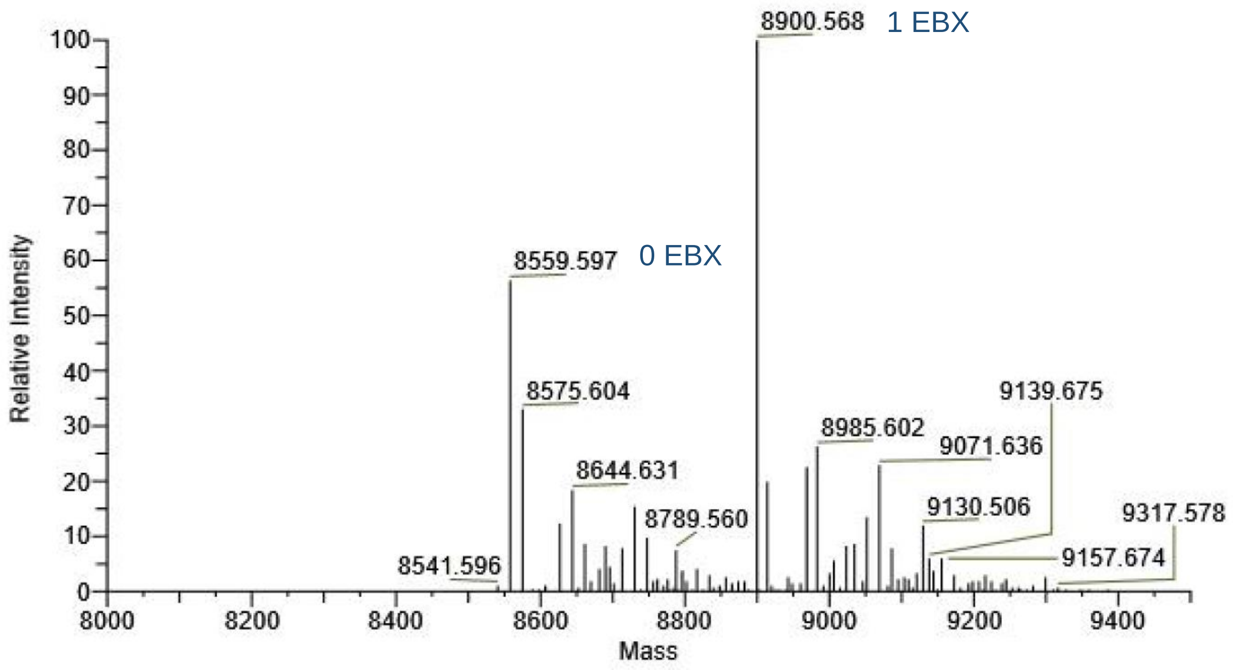

MS/MS analysis:

- 2 activation techniques combines: CID, HCD
- Multiplexing: fragmentation of 3 charge states

Prosight Lite (HCD + CID):

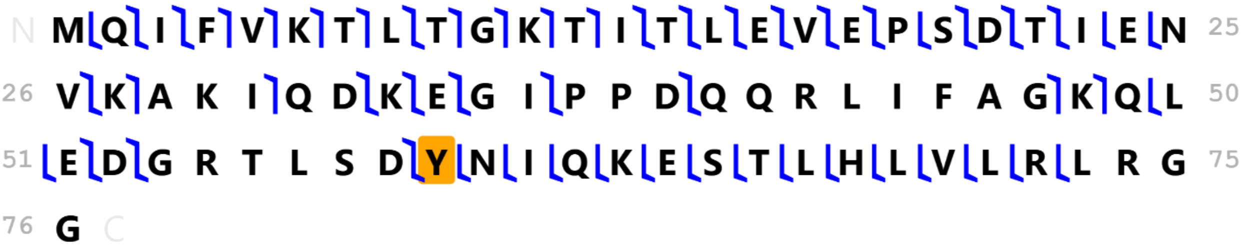

Apm2S, terminal fragments (CID):

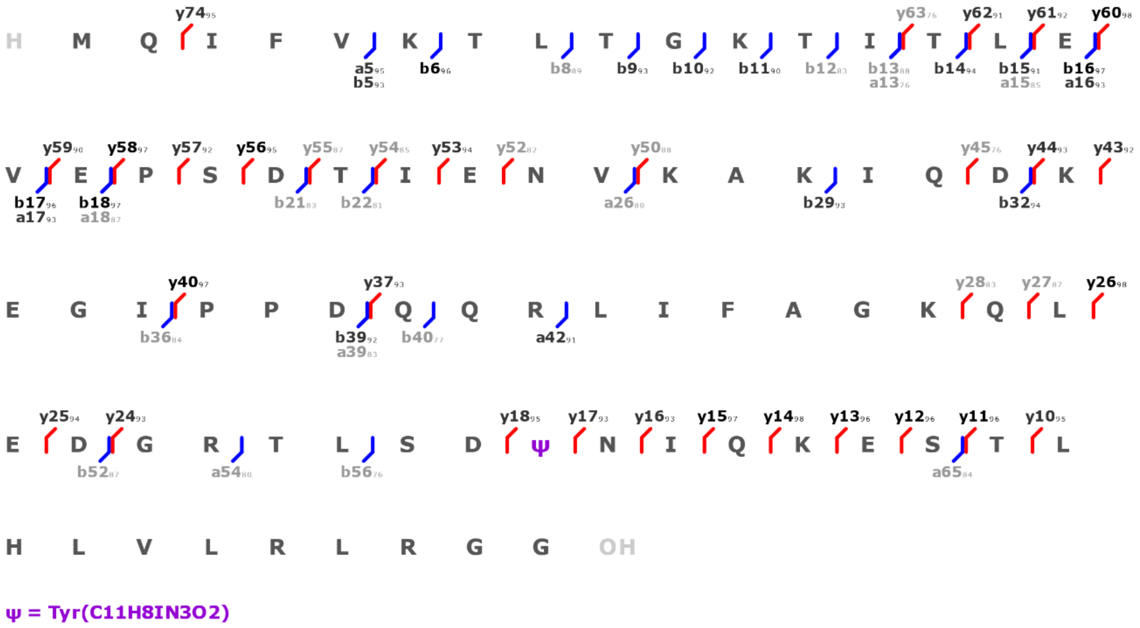

Apm2S, internal fragments (HCD):

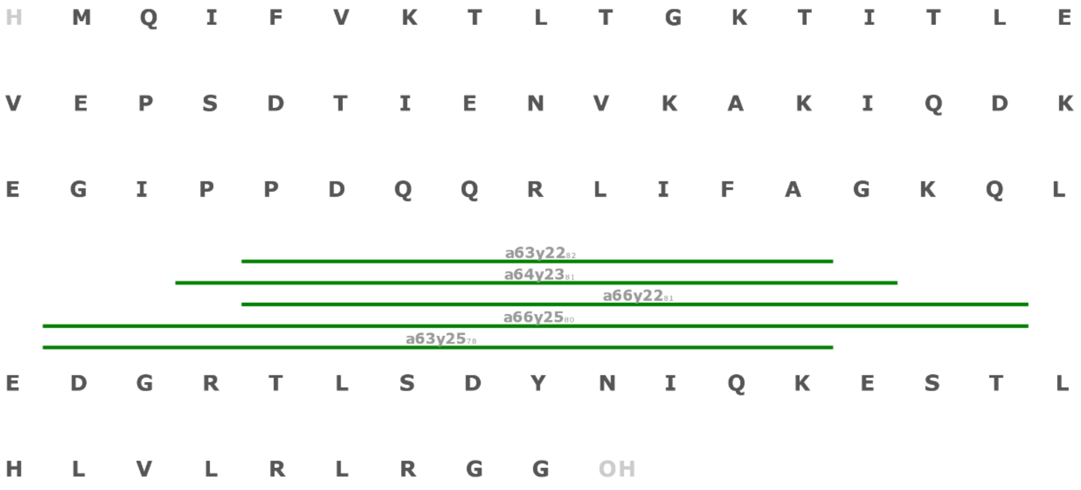

VBX-Myoglobin 7:

Deconvoluted mass spectrum:

(a) Procedure A in denaturing Tris buffer, 19% yield

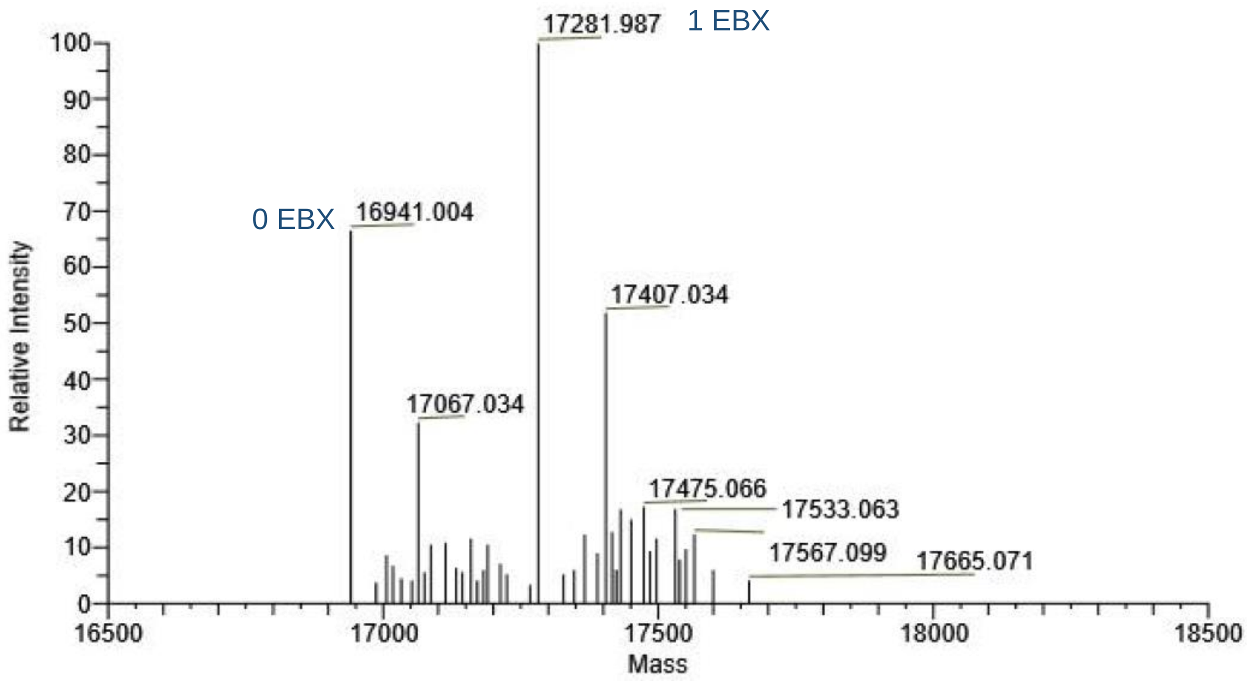

(b) Procedure B in denaturing Tris buffer, 10% yield

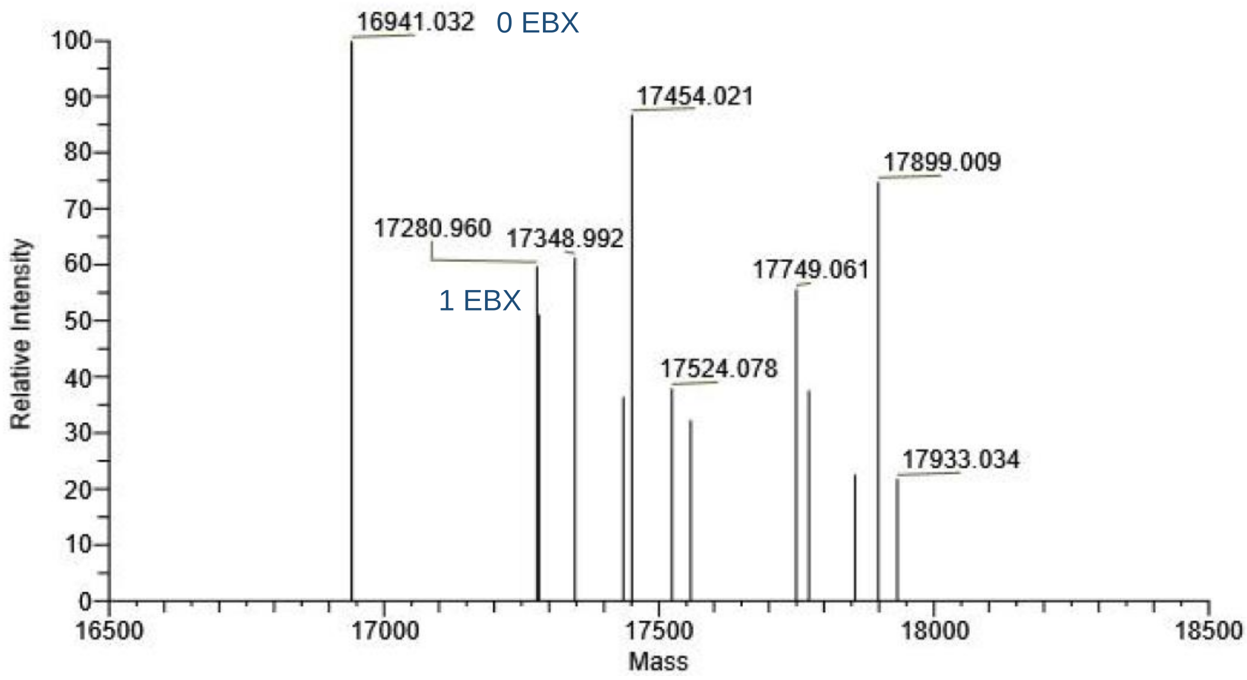

(c) Procedure A in Tris buffer, 6% yield

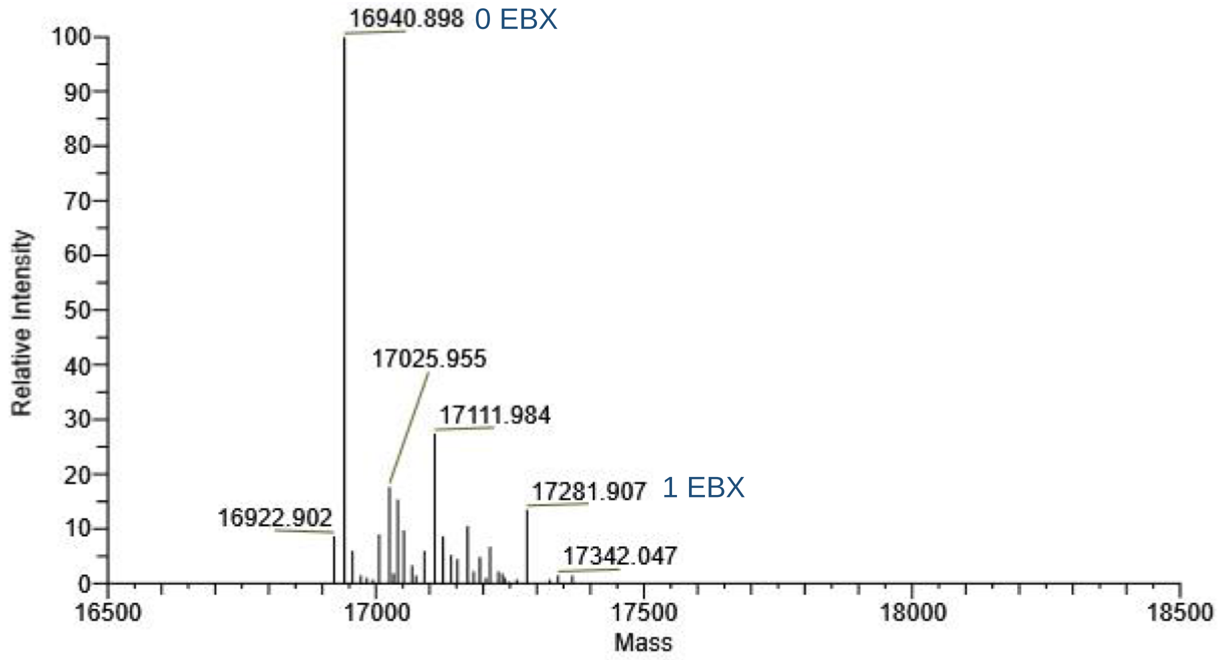

(d) Procedure B in Tris buffer, 2% yield

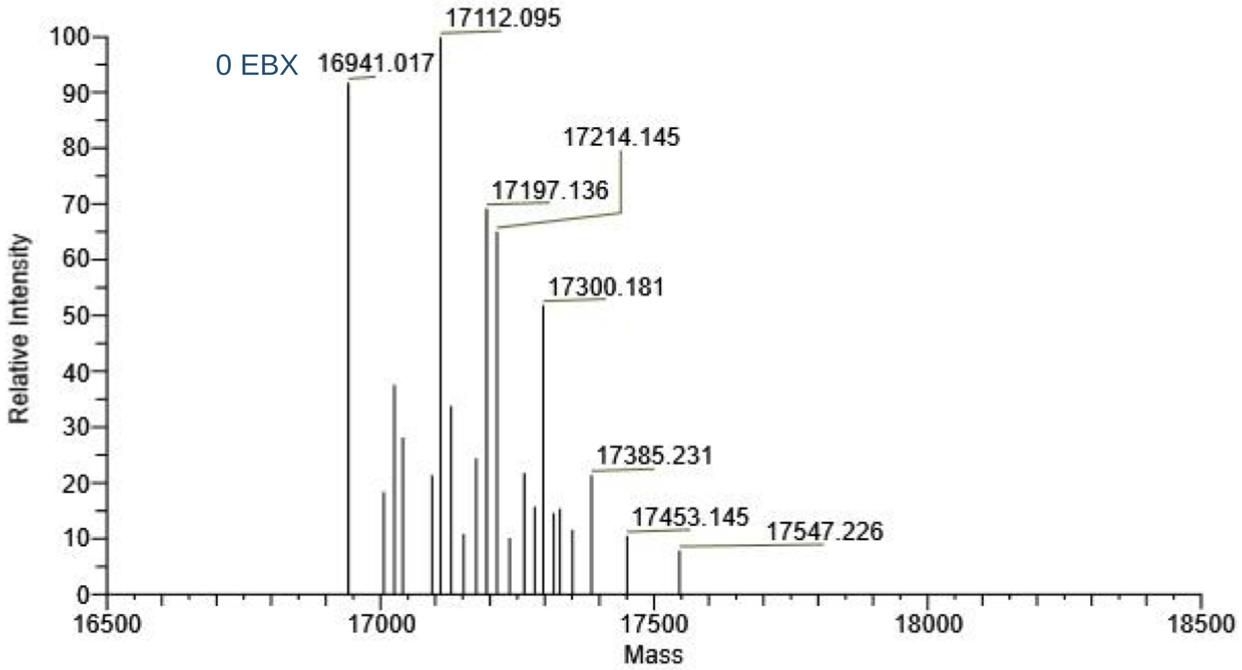

MS/MS analysis:

- 1 activation technique: HCD
- Multiplexing: fragmentation of 8 charge states

Prosight Lite (CID + HCD):

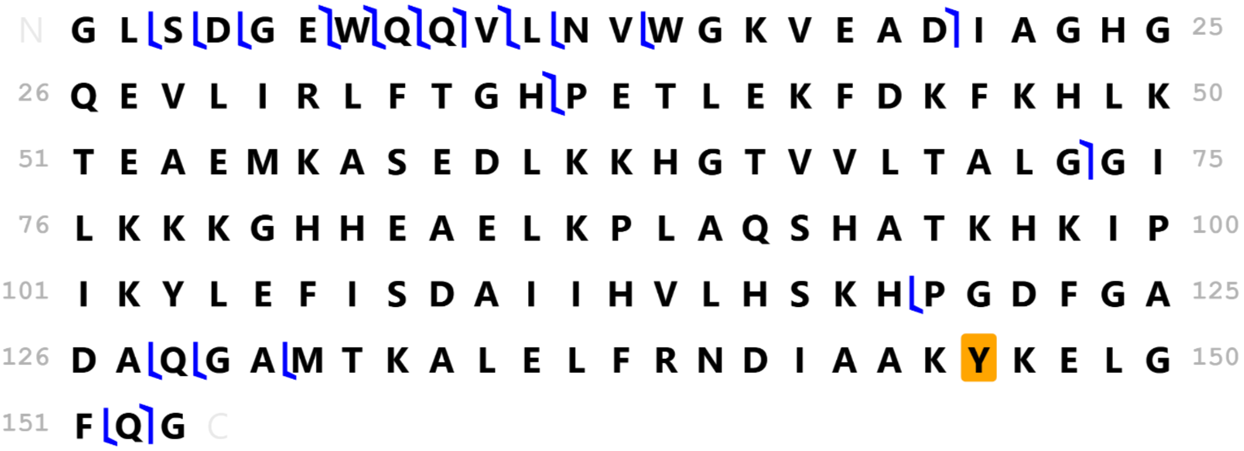

Apm2S, external fragments (HCD):

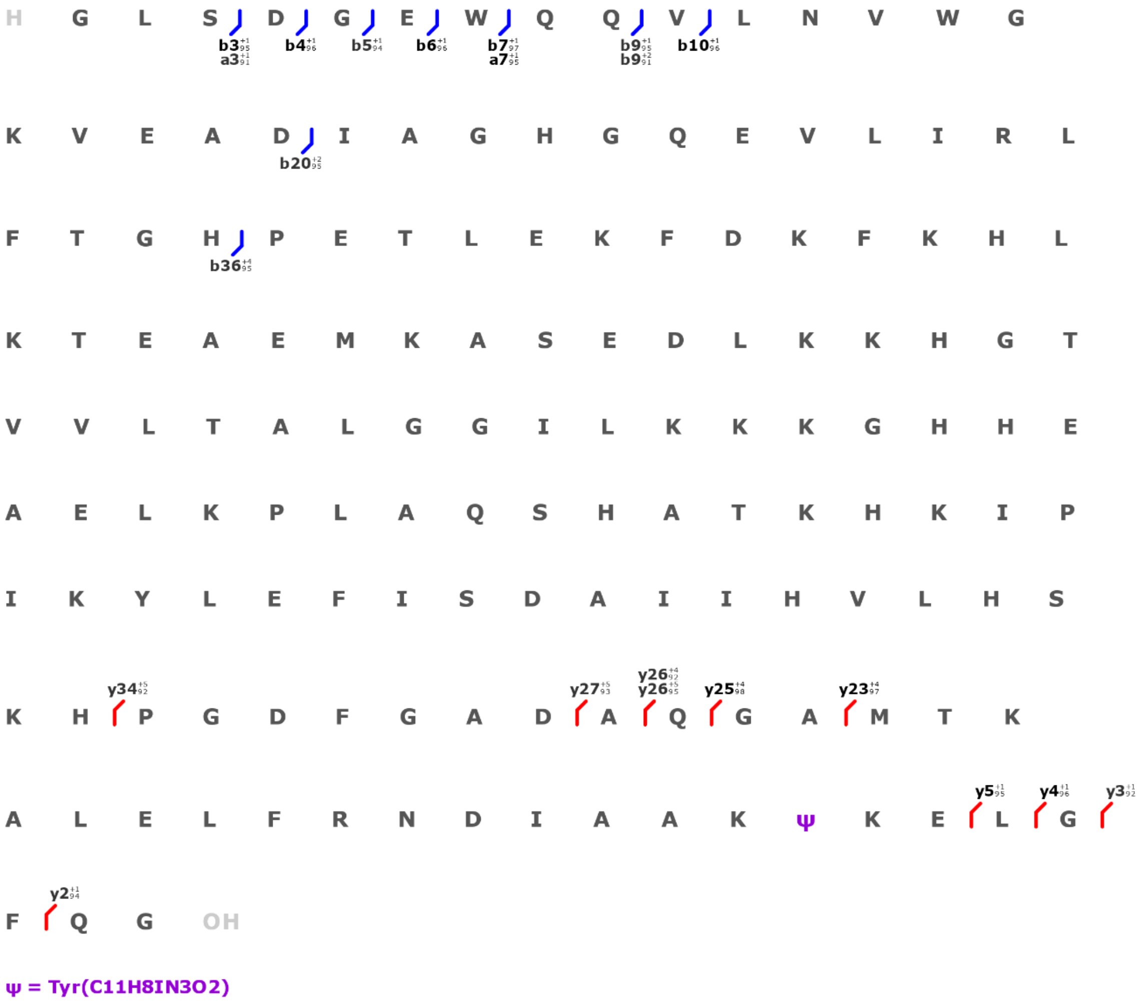

Apm2S, internal fragments (HCD):

H G L S D G E W Q Q V L N V W G

K V E A D I A G H G Q E V L I R L

F T G H P E T L E K F D K F K H L

K T E A E M K A S E D L K K H G T

V V L T A L G G I L K K K G H H E

A E L K P L A Q S H A T K H K I P

I K Y L E F I S D A I I H V L H S

$b147y25_{85}$

$b148y24_{85}$

K H P G D F G A D A Q G A M T K

$b147y25_{85}$

$b152y19_{85}$

$b148y24_{85}$

A L E L F R N D I A A K Y K E L G

$b152y19_{85}$

F Q G OH

VBX-Streptavidin 8:

Deconvoluted spectrum:

(a) Procedure A in Tris buffer, 45% yield

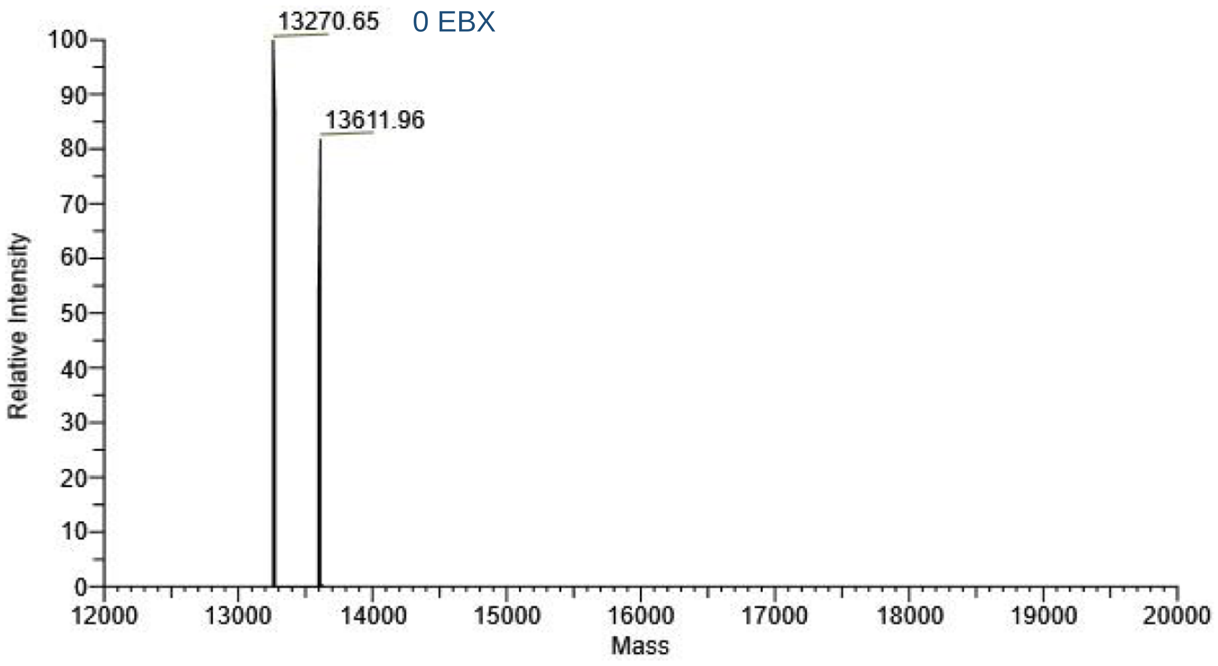

(b) Procedure B in Tris buffer, 65% yield

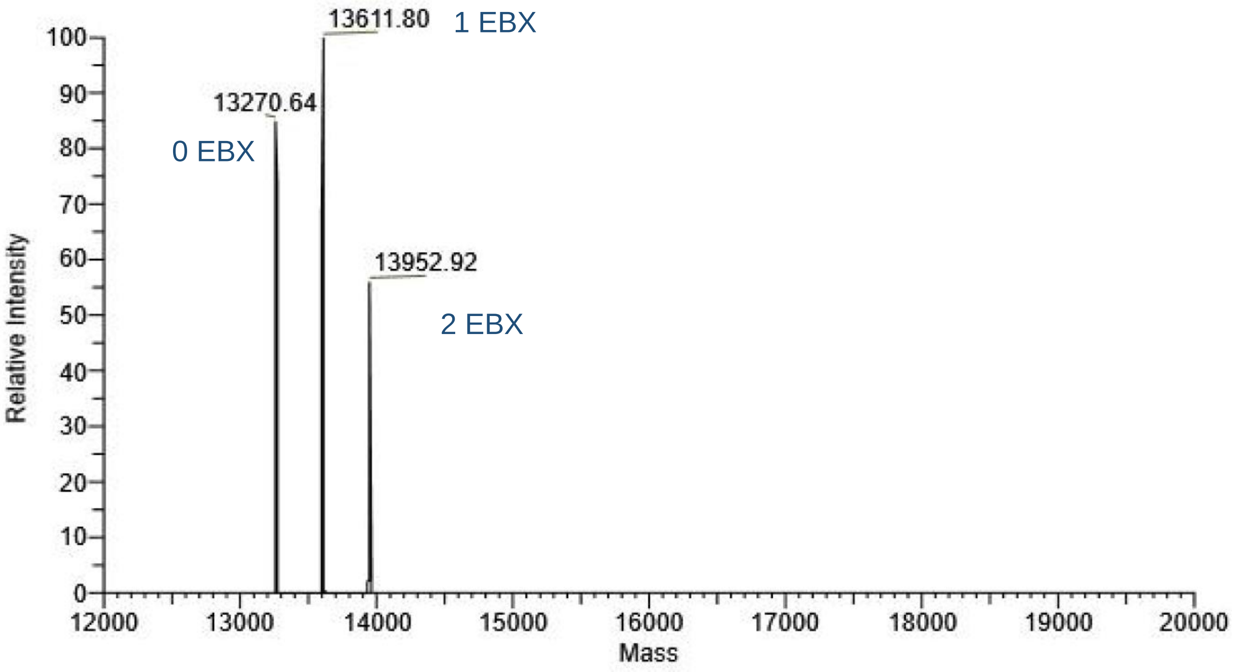

(a) Procedure A in denaturing Tris buffer, 30% yield

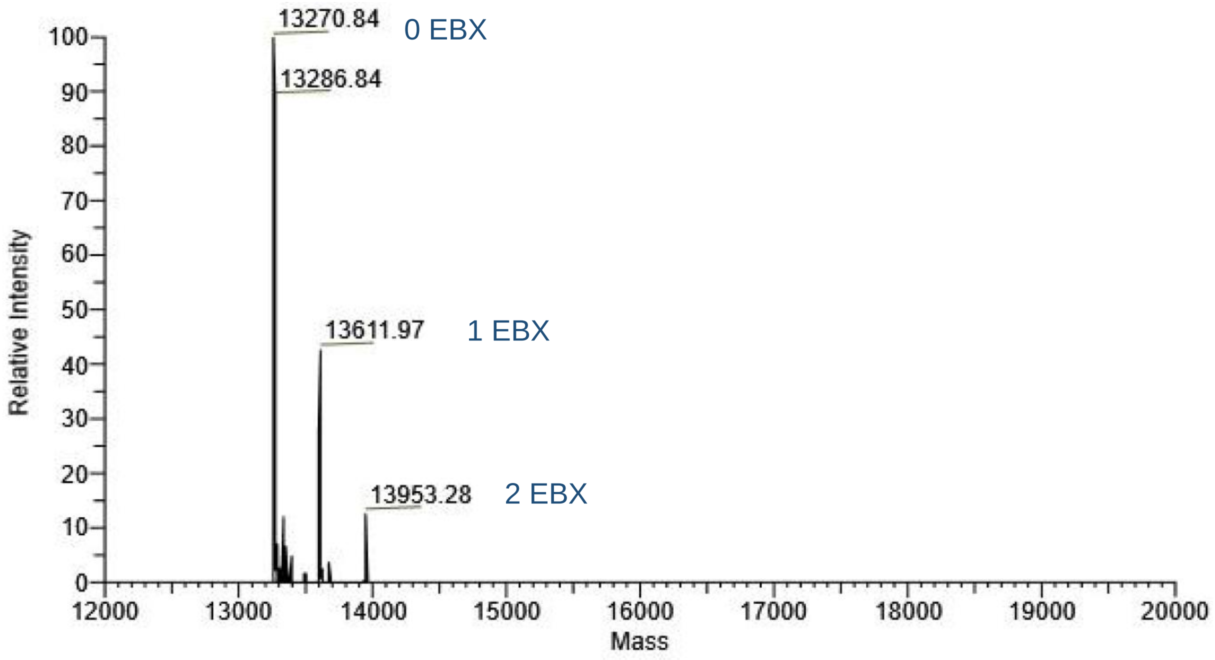

(b) Procedure B in denaturing Tris buffer, 56% yield

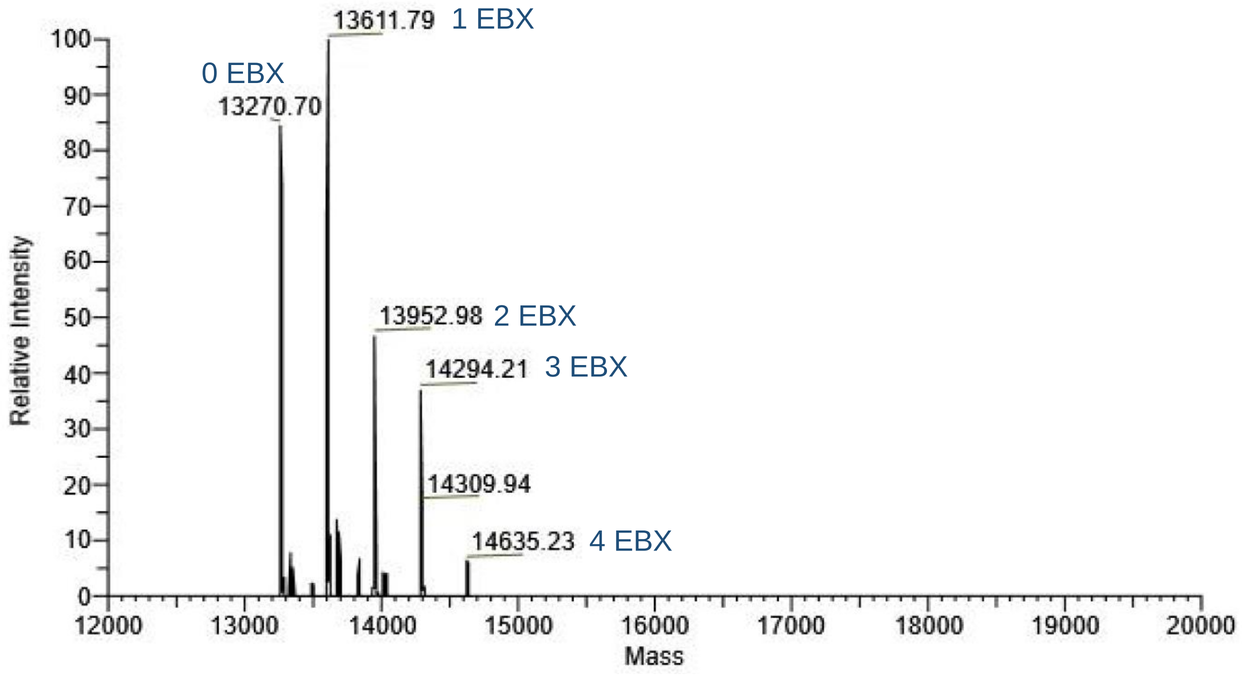

MS/MS analysis of 13611.8:

Prosight Lite (CID):

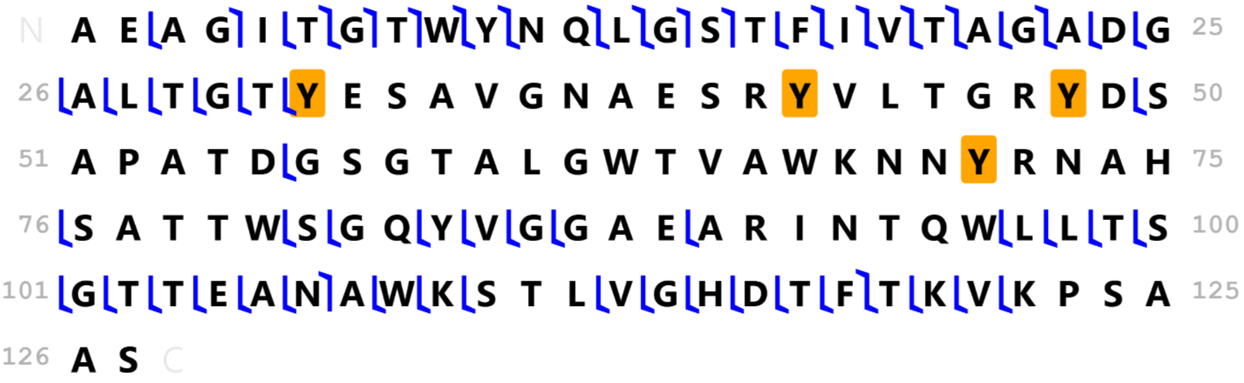

Apm2S, external fragments (CID + HCD):

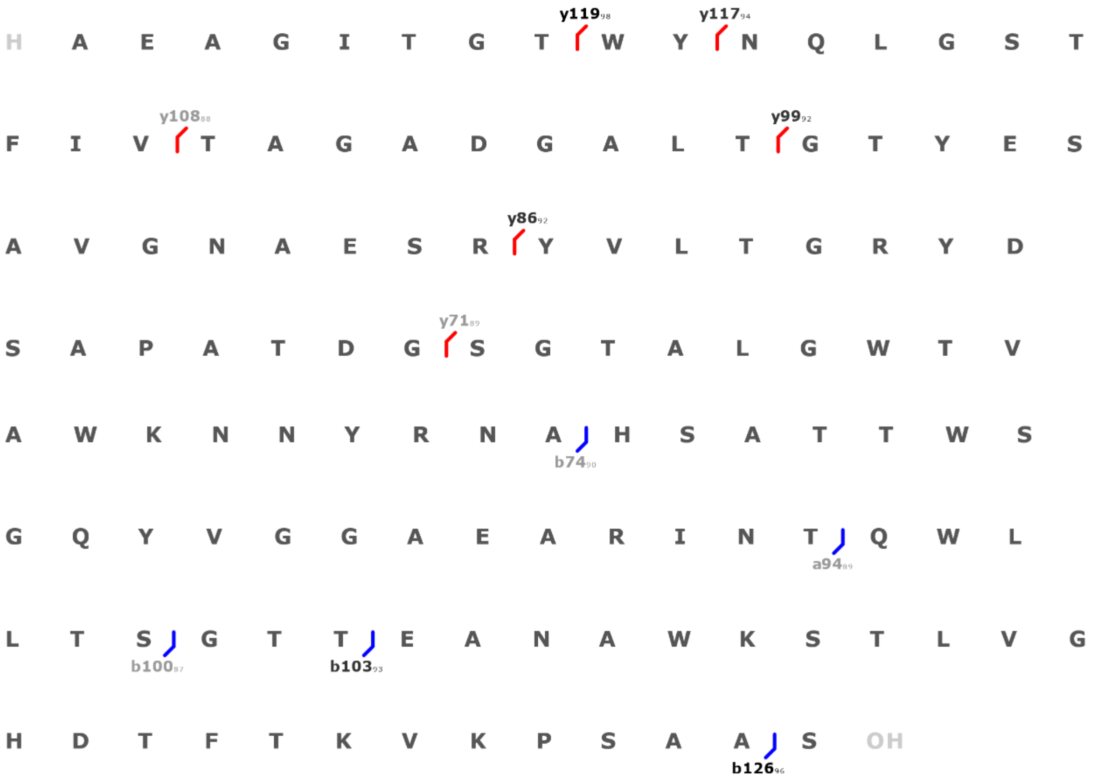

Apm2S, internal fragments:

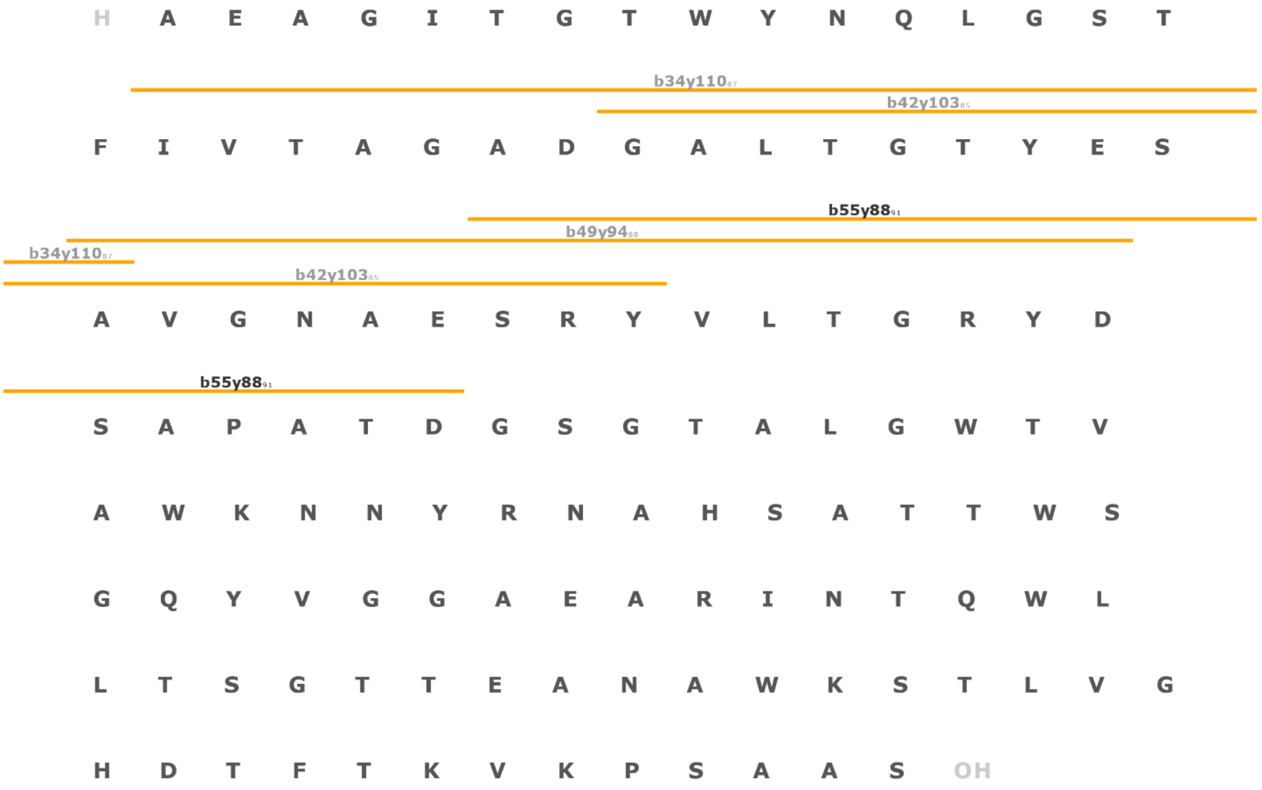

VBX-Trastuzumab 9:

SEC mass and deconvoluted spectrums of native trastuzumab:[23]

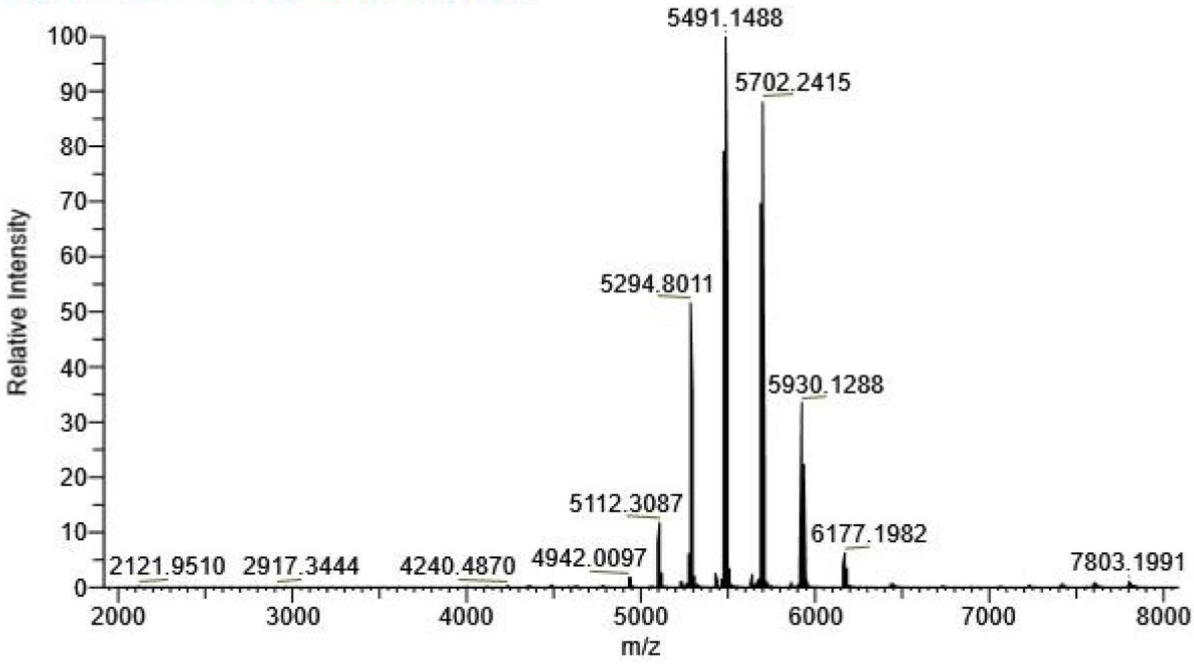

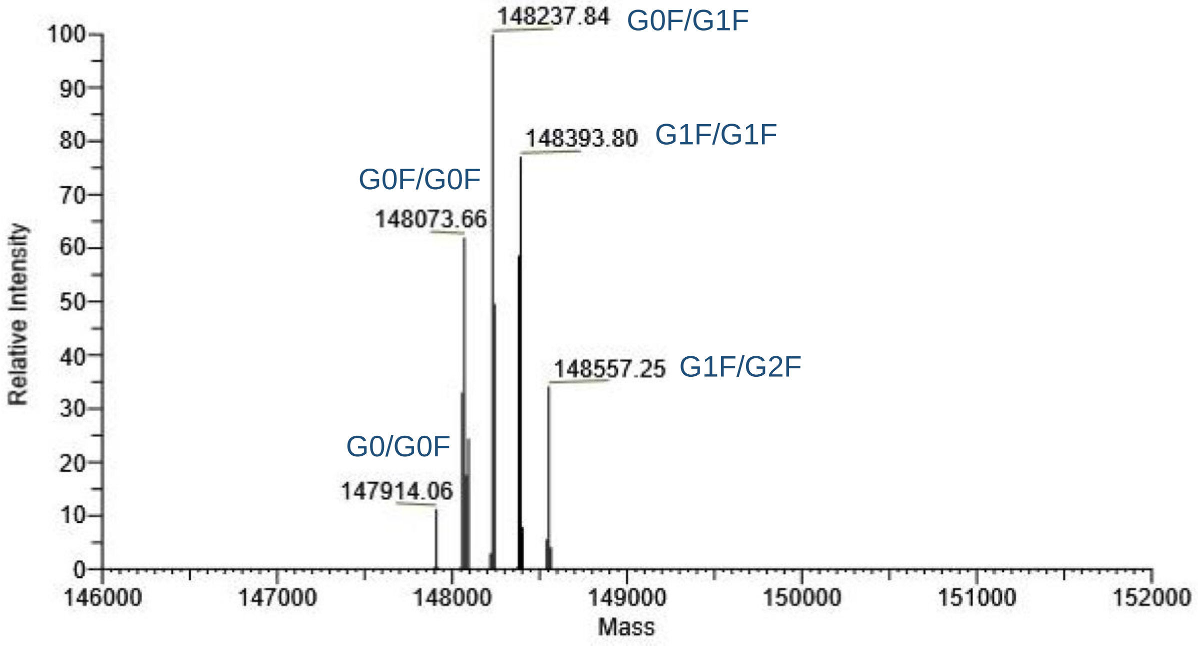

[23] (a) Cruz, E.; Sifniotis, V.; Sumer-Bayraktar, Z.; Reslan, M.; Wilkinson-White, L.; Cordwell, S.; Kayser, V. *Pharmaceutics* **2021**, *13* (11), 1747. (b) Varki, A.; Cummings, R. D.; Aebi, M.; Packer, N. H.; Seeberger, P. H.; Esko, J. D.; Stanley, P.; Hart, G.; Darvill, A.; Kinoshita, T.; Prestegard, J. J.; Schnaar, R. L.; Freeze, H. H.; Marth, J. D.; Bertozzi, C. R.; Etzler, M. E.; Frank, M.; Vliegenthart, J. F.; Lütteke, T.; Perez, S.; Bolton, E.; Rudd, P.; Paulson, J.; Kanehisa, M.; Toukach, P.; Aoki-Kinoshita, K. F.; Dell, A.; Narimatsu, H.; York, W.; Taniguchi, N.; Kornfeld, S. *Glycobiology* **2015**, *25* (12), 1323–1324. (c) Neelamegham, S.; Aoki-Kinoshita, K.; Bolton, E.; Frank, M.; Lisacek, F.; Lütteke, T.; O'Boyle, N.; Packer, N. H.; Stanley, P.; Toukach, P.; Varki, A.; Woods, R. J. *Glycobiology* **2019**, *29* (9), 620–624. (d) https://www.ncbi.nlm.nih.gov/glycans/snfg.html

Average Mass	Intensity	Relative Abundance	
148237.84	2026201.63	100	G0F/G1F
148393.80	1568736.50	77	G1F/G1F
148073.66	1263063.88	62	G0F/G0F
148557.25	696214.75	34	G1F/G2F
147914.33	502451.75	8	G0/G0F

SEC mass and deconvoluted spectrums after 4 hours with 5 equiv EBX **2a**:

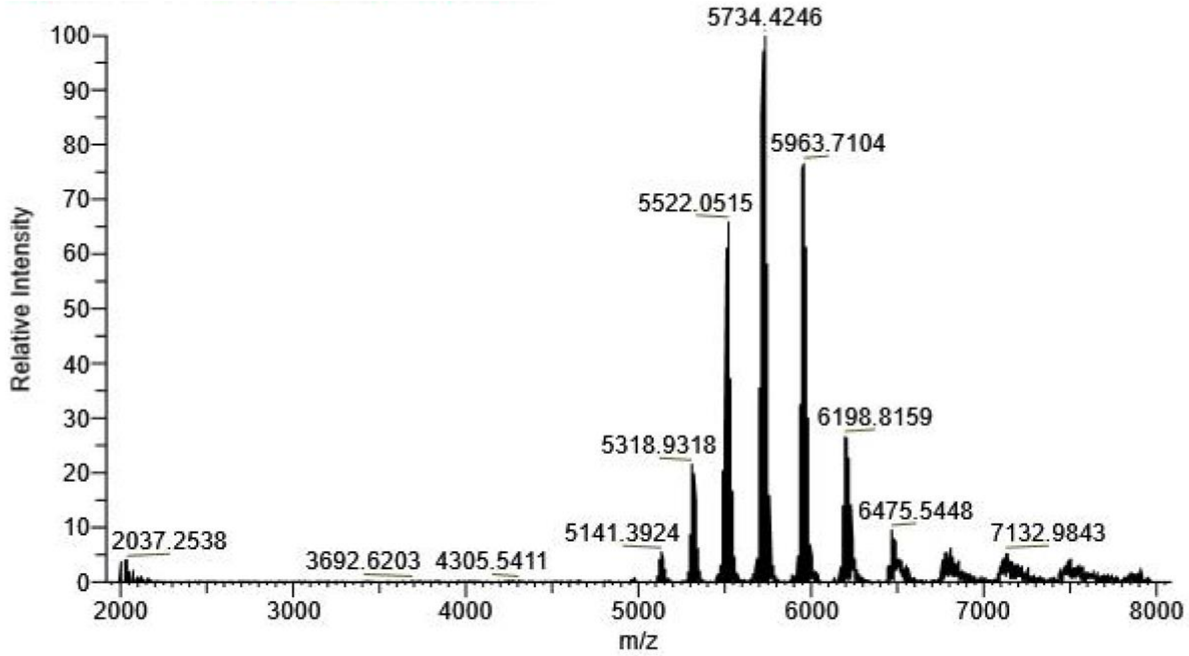

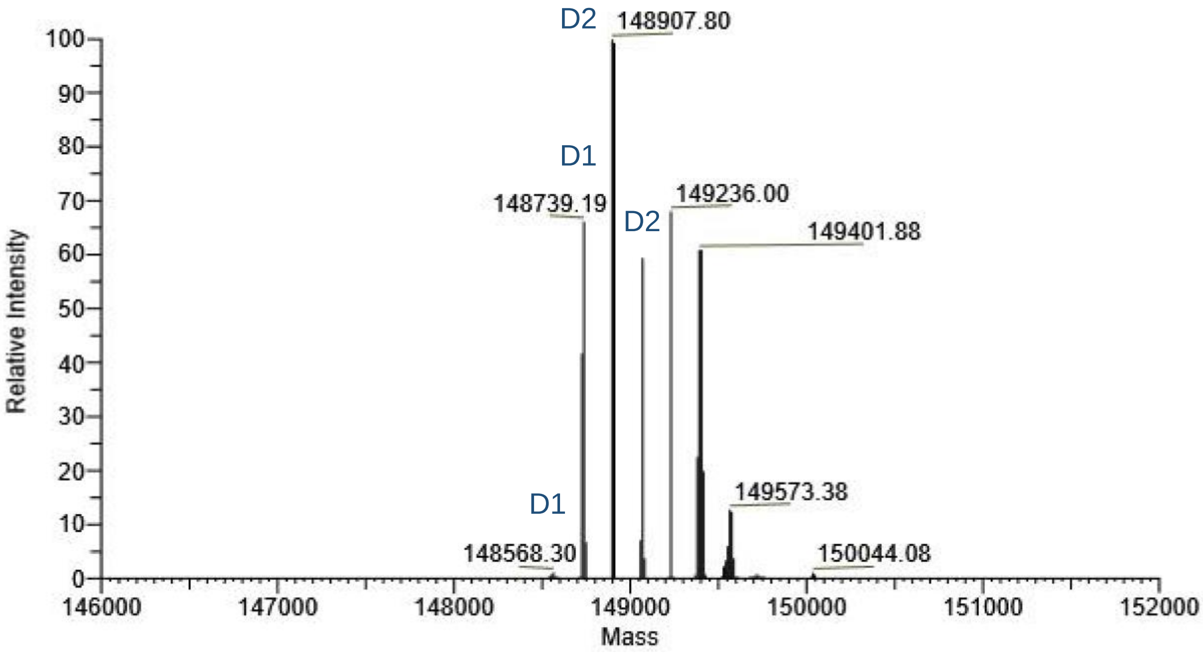

Average Mass	Intensity	Relative Abundance	
148907.80	154738.09	100	D2 = G0F/G1F + 2 EBX ± 6 Da
148739.19	102848.73	67	D1 = G1F/G1F + 1 EBX ± 2 Da
149074.33	92421.86	60	D2 = G1F/G1F + 2 EBX ± 4 Da
148568.30	2016.46	2	D1 = G0F/G1F + 1 EBX ± 4 Da

SEC mass and deconvoluted spectrums after 24 hours with 5 equiv EBX **2a**:

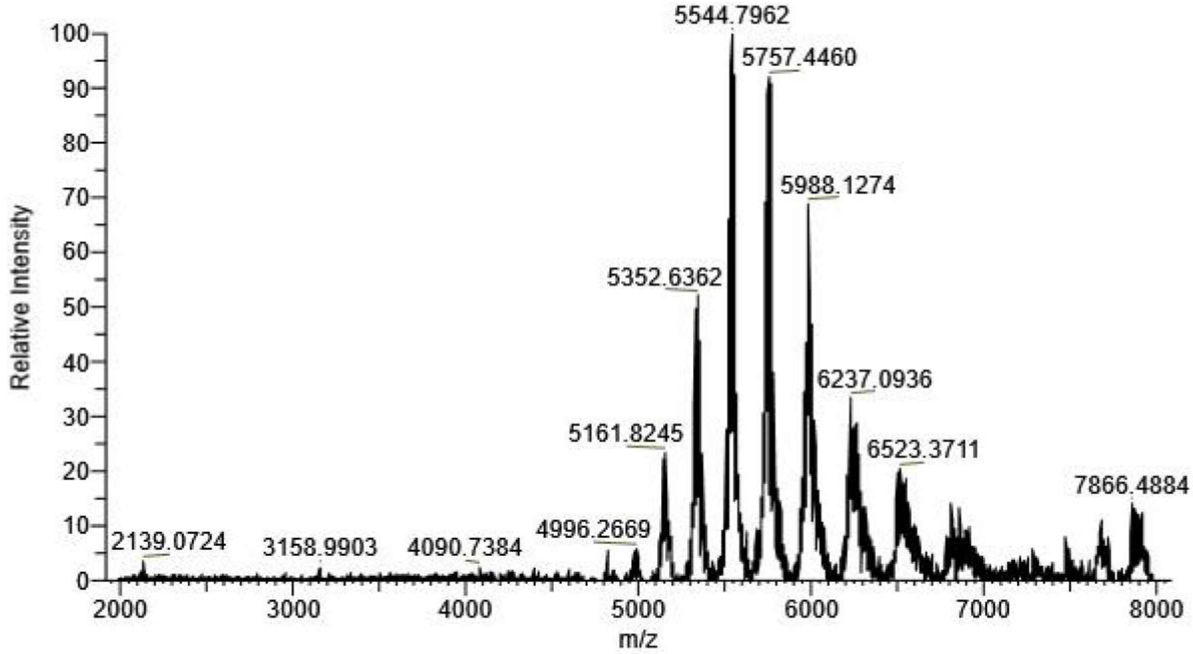

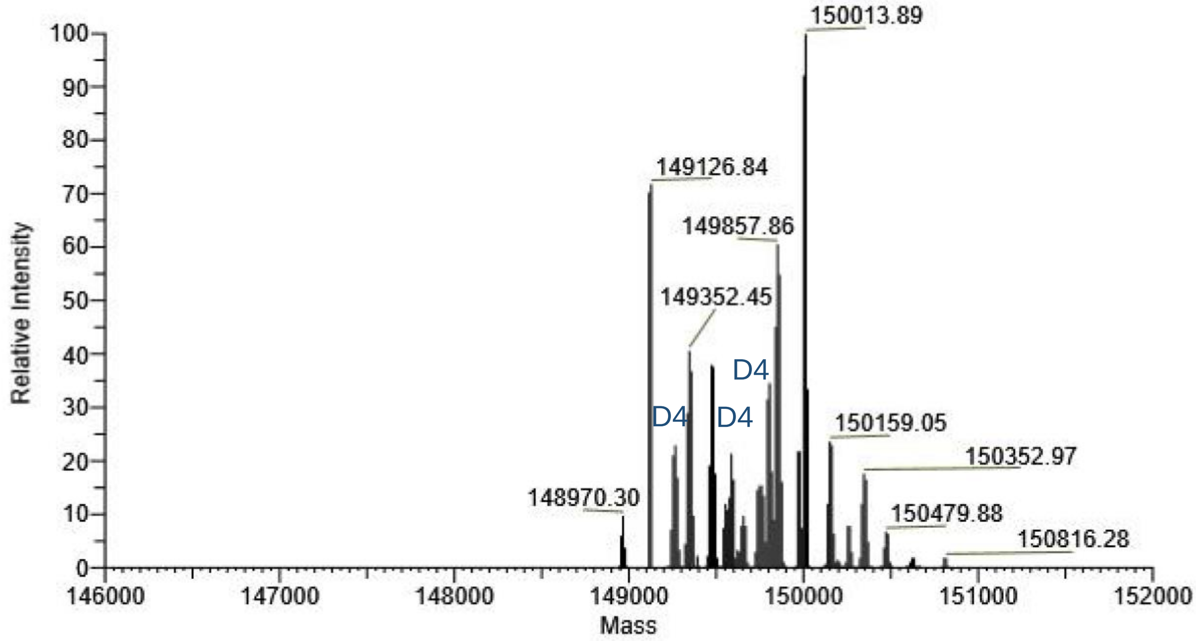

Average Mass	Intensity	Relative Abundance	
149268.13	6488.69	23	D4 = G0/G0F + 4 EBX ± 10 Da
149592.78	6129.92	22	D4 = G0F/G1F + 4 EBX ± 3 Da
149766.88	4153.25	15	D4 = G1F/G1F + 4 EBX ± 7 Da
149748.64	3990.52	14	D4 = G1F/G1F + 4 EBX ± 10 Da

SEC mass and deconvoluted spectrums after 24 hours with 10 equiv EBX **2a**:

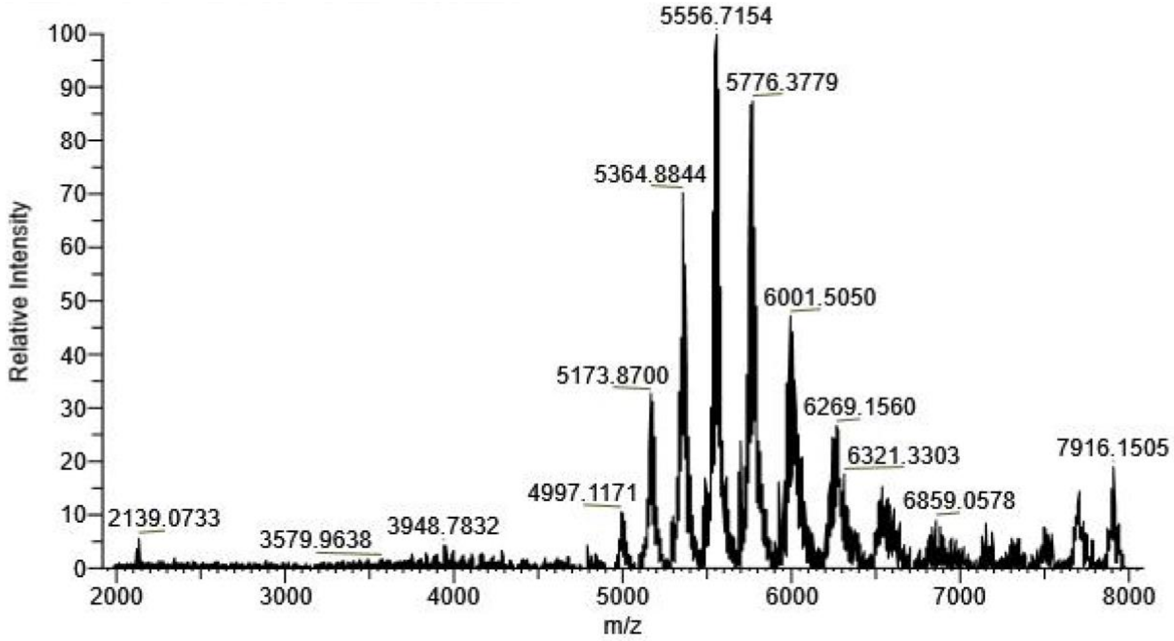

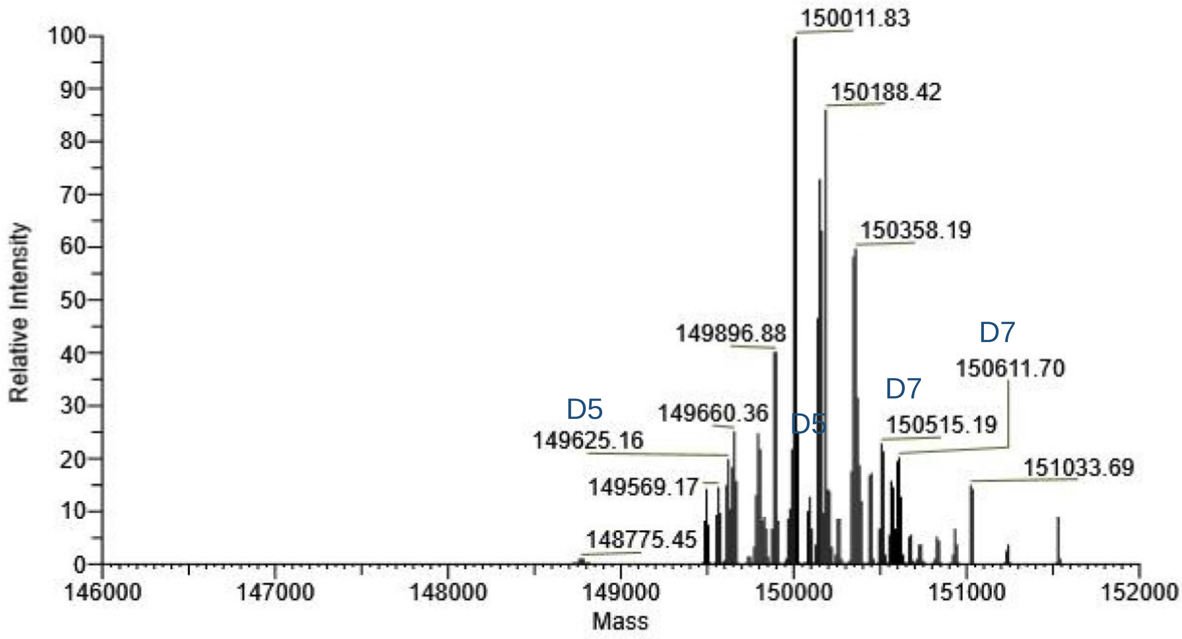

Average Mass	Intensity	Relative Abundance	
150611.70	4584.09	21	D7 = G0F/G1F + 7 EBX ± 7 Da
149625.16	4461.72	20	D5 = G0/G0F + 5 EBX ± 5 Da
150449.19	3900.09	18	D7 = G0F/G1F + 7 EBX ± 9 Da
150096.25	2873.56	13	D5 = G1F/G1F + 5 EBX ± 5 Da

8. Scope of EBX on β-Casomorphin

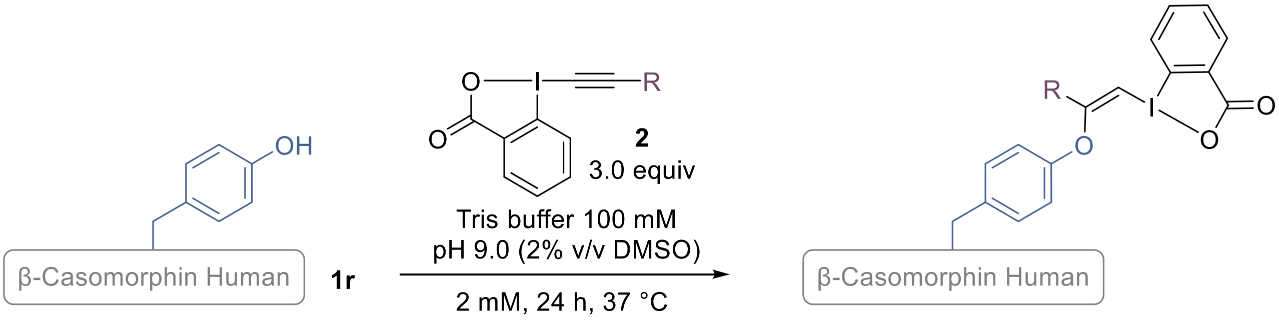

General procedure:

To a solution of β-Casomorphin **1r** (864 µg, 1.00 µmol, 1.00 equiv) in 100 mM Tris buffer pH 9.0 (490 µL), in a 0.5 mL Eppendorf Safe-Lock microcentrifuge tube, was added a 300 mM solution of EBX **2** in DMSO (10.0 µL, 3.00 µmol, 3.00 equiv). The 2.00 mM solution was stirred at 37 °C for 24 h. No effort was made to exclude oxygen. The reaction was analysed by HPLC-MS.

The yields were approximated as the ratio of Aprod/Atotal where Aprod = area in mAU of the product peak and Atotal = area in mAU of all peptides products (product, starting material, and side products if present). The ratio were determined using HPLC-UV at 214 nm and/or HPLC-MS chromatograms.

O-VBX (3rb)

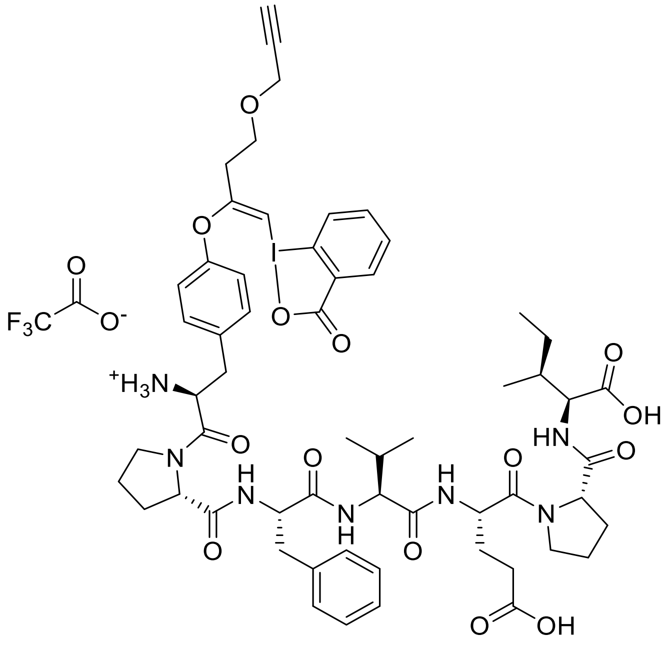

Starting from β-Casomorphin (**1r**) (0.864 mg, 1.00 μmol), O-VBX (**3rb**) was obtained in 66% yield (retention time 9.7 min).

HRMS (nanochip-ESI/LTQ-Orbitrap) m/z: $[M + H_2]^{+2}$ Calcd for $C_{58}H_{74}IN_7O_{14}^{+2}$ 609.7164; Found 609.7147.

MS/MS characterization:

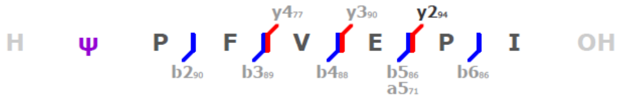

ψ = Tyr(C14H11IO3)

Sequence	Type	MF	m/z	Intensity	Similarity
**YPFVE**	b5	C47H53IN5O11(+1)	990.2781	101.36	0.87
PI	y2	C11H21N2O3(+1)	229.1547	73.07	0.94
**YP**	b2	C28H28IN2O6(+1)	615.0987	24.95	0.90
**YPFVEP**	b6	C52H60IN6O12(+1)	544.1691	10.59	0.86
**YPFVE**	b5	C47H51IN5O10(+1)	972.2675	9.05	0.88
**YPFV**	b4	C42H46IN4O8(+1)	861.2355	8.81	0.90
**YPFVE**	b5	C47H53IN5O11(+1)	495.6427	8.02	0.85
EPI	y3	C16H28N3O6(+1)	358.1973	4.94	0.90
**YPF**	b3	C37H37IN3O7(+1)	762.1671	3.78	0.89
VEPI	y4	C21H37N4O7(+1)	457.2657	0.92	0.77
**YPFVE**	a5	C46H53IN5O10(+1)	481.6452	0.70	0.71

HPLC-UV chromatogram (214 nm) of a 2.00 mM solution of β-Casomorphin (**1r**) in 100 mM Tris buffer pH 9.0:

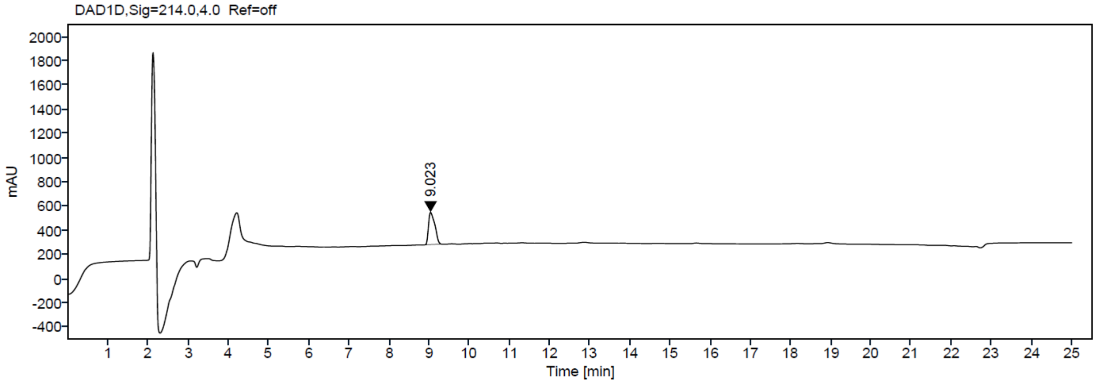

HPLC-UV chromatograms (214 nm) of crude of the reaction:

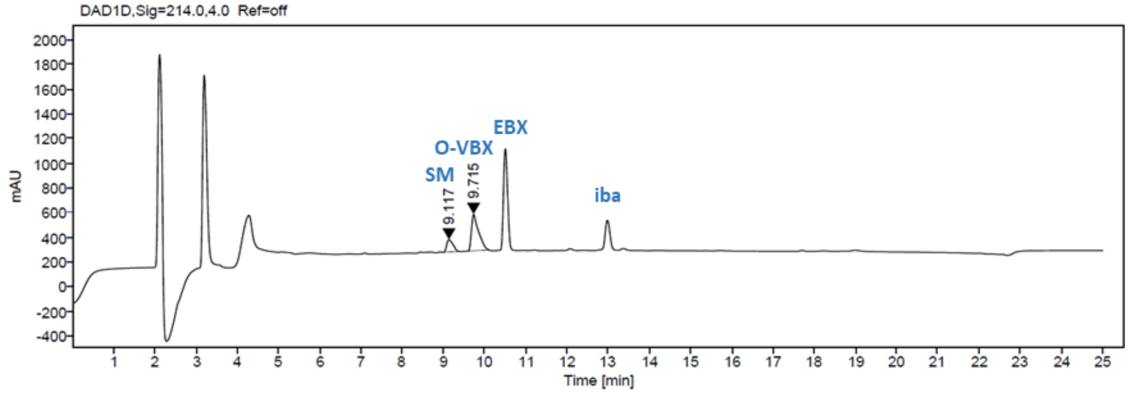

HPLC-MS chromatograms of crude of the reaction:

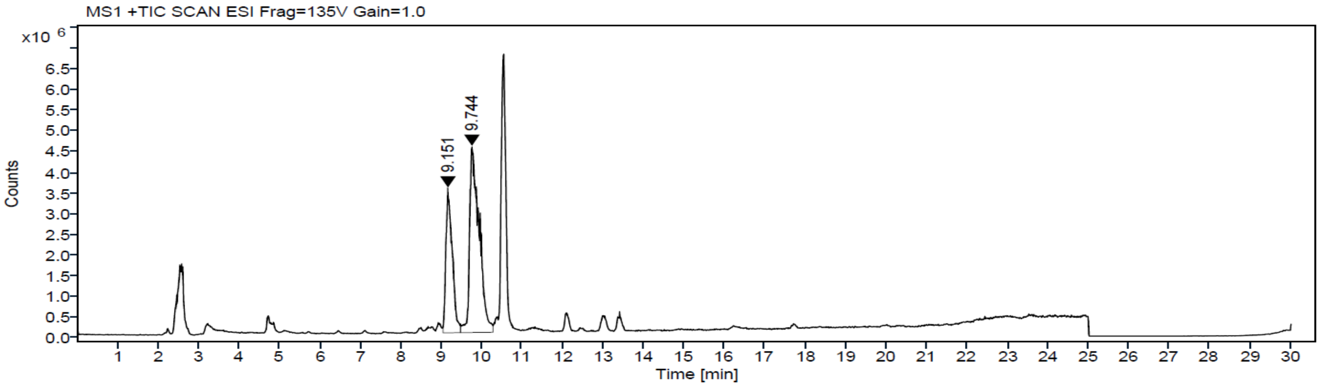

Yield based on HPLC-UV at 214 nm: 77%

Yield based on HPLC-MS: 66%

O-VBX (3rc)

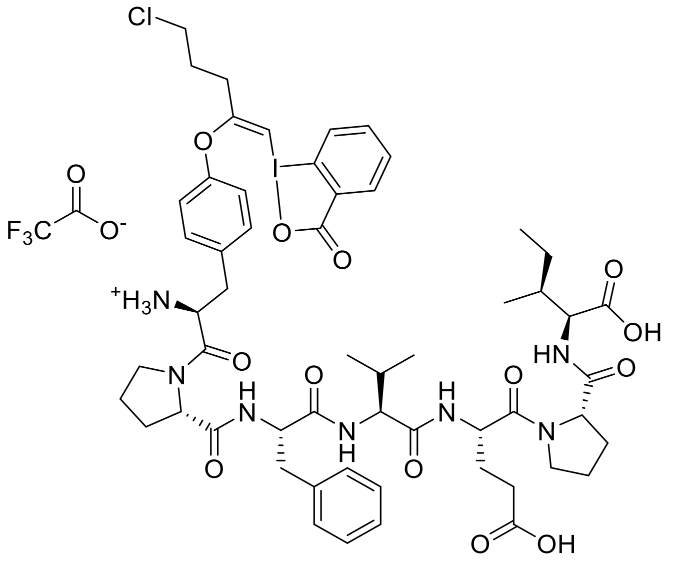

Starting from β-Casomorphin (**1r**) (0.864 mg, 1.00 μmol), O-VBX (**3rc**) was obtained in 78% yield (retention time 10.1 min).

HRMS (LTQ-Orbitrap) m/z: $[M + H]^+$ Calcd for $C_{56}H_{72}ClIN_7O_{13}^+$ 1212.3916; Found 1212.3951.

MS/MS characterization:

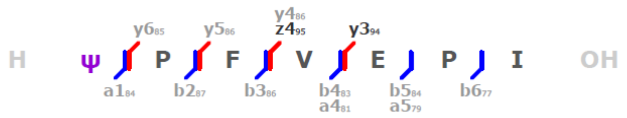

ψ = Tyr(C12H10ClIO2)

Sequence	Type	MF	m/z	Intensity	Similarity
**YP**	b2	C26H27ClIN2O5(+1)	609.0646	100.91	0.87
**YPFVE**	b5	C45H52ClIN5O10(+1)	984.2438	16.80	0.85
**YPFV**	b4	C40H45ClIN4O7(+1)	855.2015	6.08	0.84
EPI	y3	C16H28N3O6(+1)	358.1973	5.35	0.83
PFVEPI	y6	C35H53N6O9(+1)	701.3866	3.89	0.94
**YPFVE**	b5	C45H50ClIN5O9(+1)	966.2335	3.32	0.90
**YPF**	b3	C35H36ClIN3O6(+1)	756.133	2.72	0.84
**Y**	a1	C20H20ClINO3(+1)	484.0169	1.52	0.86
VEPI	y4	C21H37N4O7(+1)	457.2656	1.41	0.84
**YPFV**	a4	C39H45ClIN4O6(+1)	827.2064	1.34	0.91
**YPFVE**	a5	C44H52ClIN5O9(+1)	956.249	1.23	0.81
FVEPI	y5	C30H46N5O8(+1)	604.3339	0.73	0.83

HPLC-UV chromatogram (214 nm) of a 2.00 mM solution of β-Casomorphin (**1r**) in 100 mM Tris buffer pH 9.0:

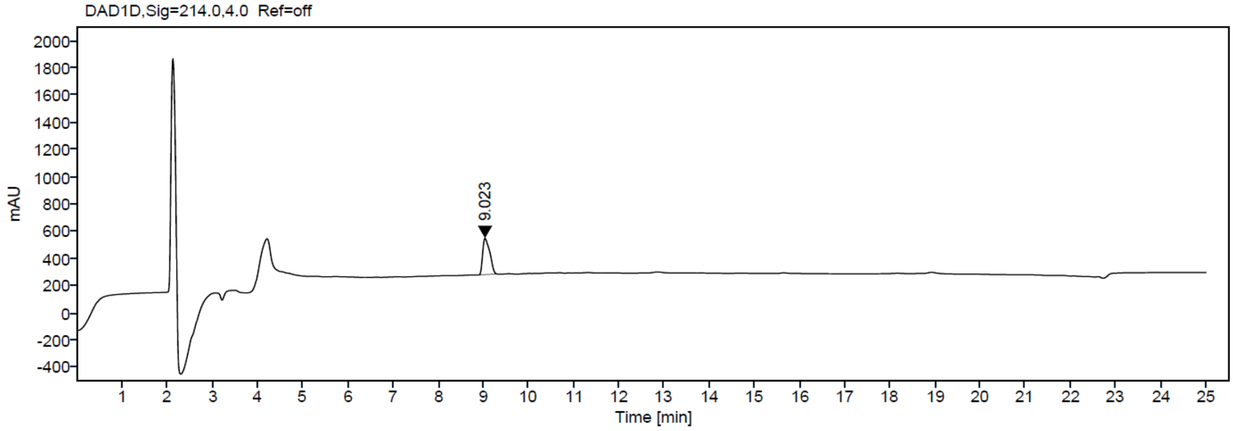

HPLC-UV chromatograms (214 nm) of crude of the reaction:

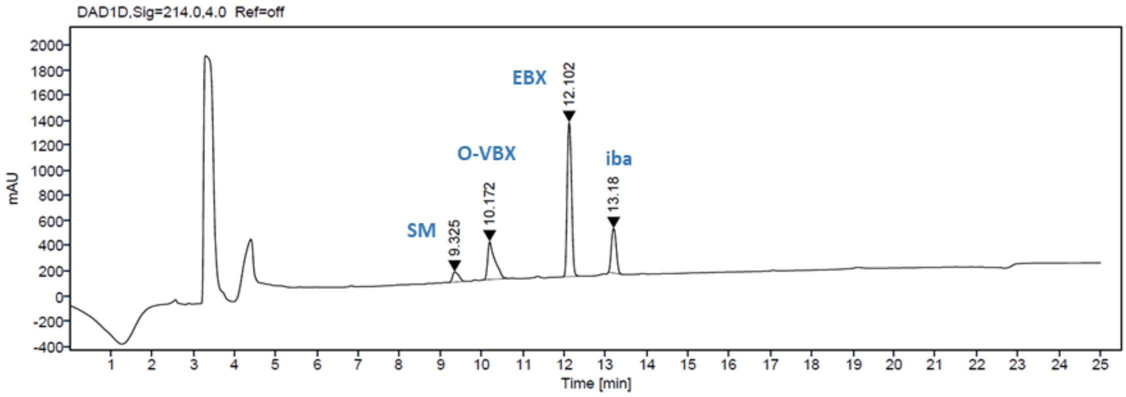

HPLC-MS chromatograms of crude of the reaction:

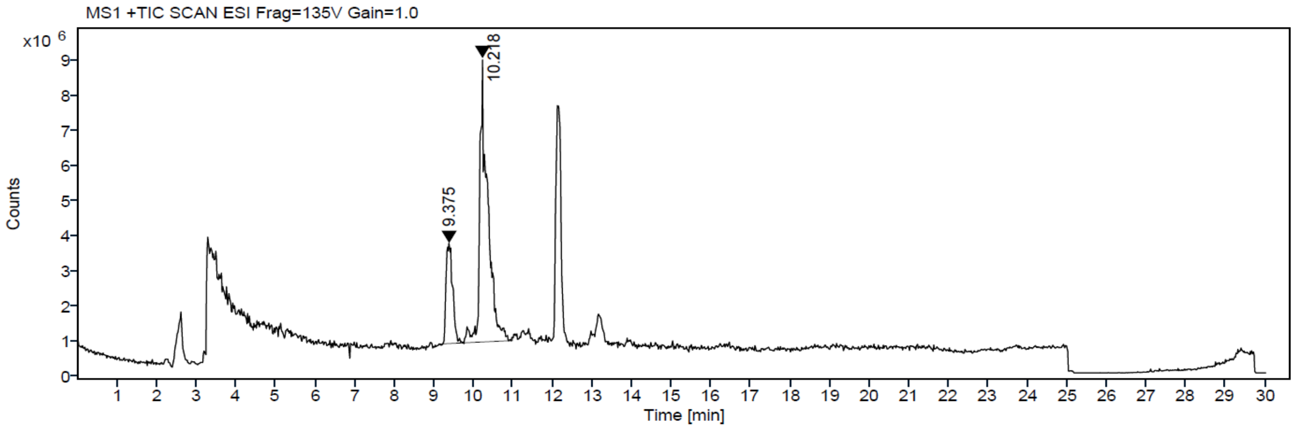

Yield based on HPLC-UV at 214 nm: 81%

Yield based on HPLC-MS: 78%

O-VBX (3rd)

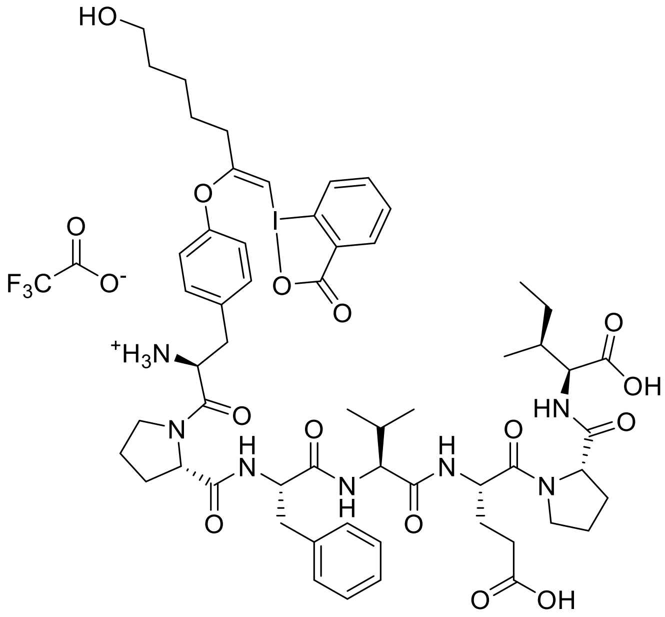

Starting from β-Casomorphin (**1r**) (0.864 mg, 1.00 μmol), O-VBX (**3rd**) was obtained in 45% yield (retention time 7.1 min).

HRMS (nanochip-ESI/LTQ-Orbitrap) m/z: $[M + H]^+$ Calcd for $C_{58}H_{77}IN_7O_{14}^+$ 1222.4568; Found 1222.4544.

MS/MS characterization:

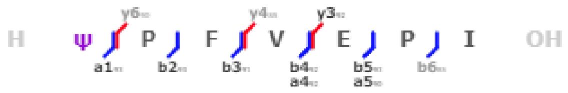

ψ = Tyr(C14H15IO3)

Sequence	Type	MF	m/z	Intensity	Similarity
YP	b2	C28H32IN2O6(+1)	619.13	102	93%
YPFVE	b5	C47H57IN5O11(+1)	994.3094	12.28	93%
PFVEPI	y6	C35H53N6O9(+1)	701.3869	6.38	90%
EPI	y3	C16H28N3O6(+1)	358.1973	6.35	92%
YPFV	b4	C42H50IN4O8(+1)	865.2668	4.95	92%
YPF	b3	C47H55IN5O10(+1)	766.1984	2.87	91%
Y	a1	C37H41IN3O7(+1)	494.0823	2.61	93%
YPFV	a4	C22H25INO4(+1)	837.2719	1.84	92%
YPFVE	a5	C41H50IN4O7(+1)	966.3145	1.56	90%
VEPI	y4	C46H57IN5O10(+1)	457.2657	1.17	85%
YPFVEP	b6	C21H37N4O7(+1)	1091.362	0.21	85%

HPLC-UV chromatogram (214 nm) of a 2.00 mM solution of β-Casomorphin (**1r**) in 100 mM Tris buffer pH 9.0:

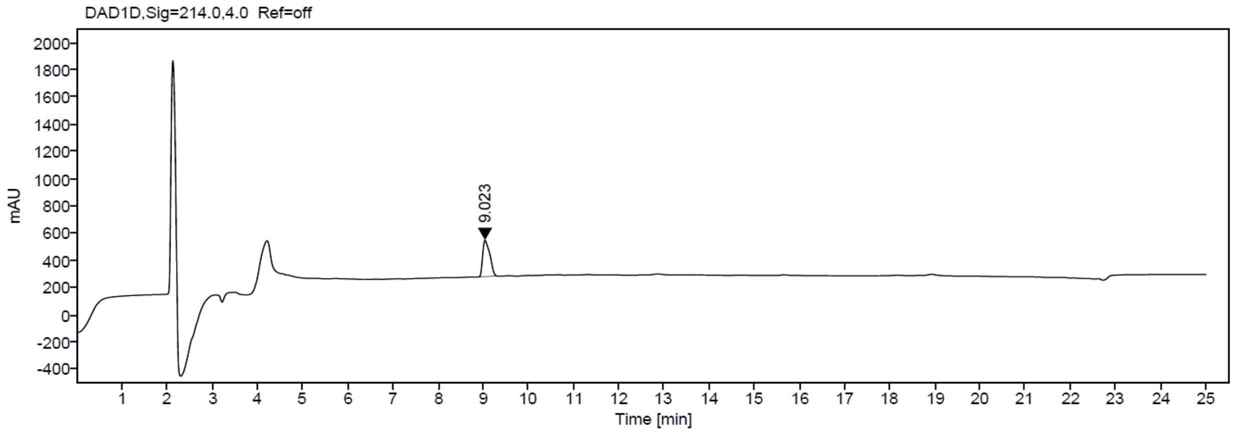

HPLC-UV chromatograms (214 nm) of crude of the reaction:

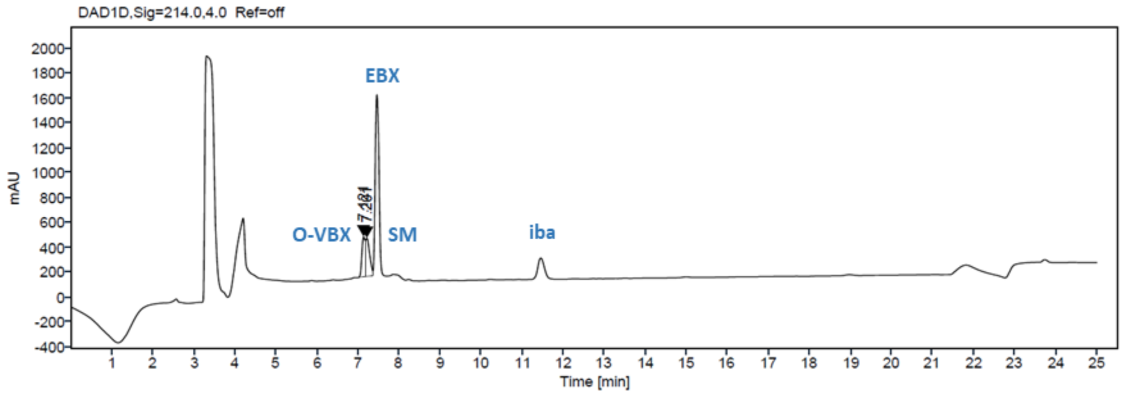

HPLC-MS chromatograms of crude of the reaction:

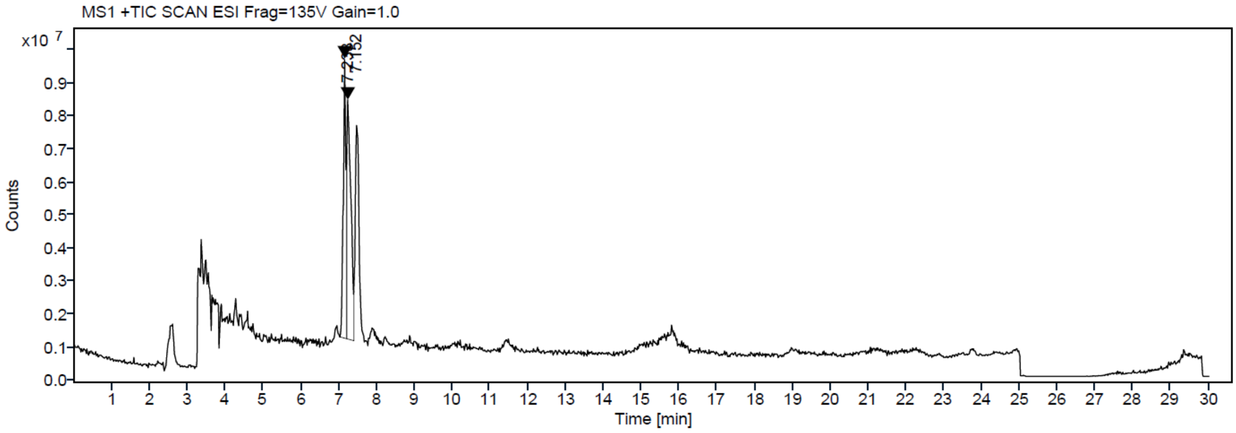

Yield based on HPLC-UV at 214 nm: 46%

Yield based on HPLC-MS: 45%

O-VBX (3re)

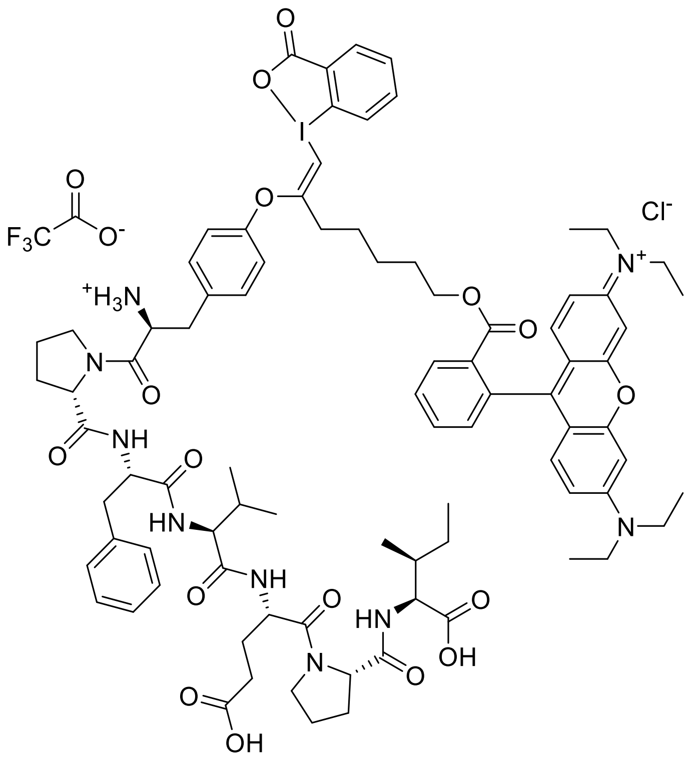

Starting from β-Casomorphin (**1r**) (0.864 mg, 1.00 μmol), O-VBX (**3re**) was obtained in 76% yield (retention time 12.7 min).

HRMS (nanochip-ESI/LTQ-Orbitrap) m/z: $[M + H]^{+2}$ Calcd for $C_{86}H_{106}IN_9O_{16}^{+2}$ 823.8396; Found 823.8379.

MS/MS characterization:

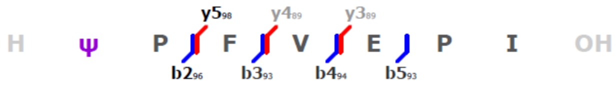

ψ = Tyr(C42H43IN2O5)

Sequence	Type	MF	m/z	Intensity	Similarity
**YP**	b2	C56H60IN4O8(+1)	522.1762	100.95	0.94
**YPFVE**	b5	C75H85IN7O13(+1)	709.7659	28.84	0.93
FVEPI	y5	C30H46N5O8(+1)	604.3341	17.08	0.98
**YP**	b2	C56H60IN4O8(+1)	1043.345	12.77	0.97
**YPFVE**	b5	C75H83IN7O12(+1)	700.7606	5.97	0.93
**YPFV**	b4	C70H78IN6O10(+1)	645.2446	5.61	0.94
**YPF**	b3	C65H69IN5O9(+1)	595.7104	1.53	0.93
EPI	y3	C16H28N3O6(+1)	358.1973	1.38	0.94
EPI	y3	C16H26N3O5(+1)	340.1867	0.64	0.89
VEPI	y4	C21H37N4O7(+1)	457.2657	0.51	0.84

HPLC-UV chromatogram (214 nm) of a 2.00 mM solution of β-Casomorphin (**1r**) in 100 mM Tris buffer pH 9.0:

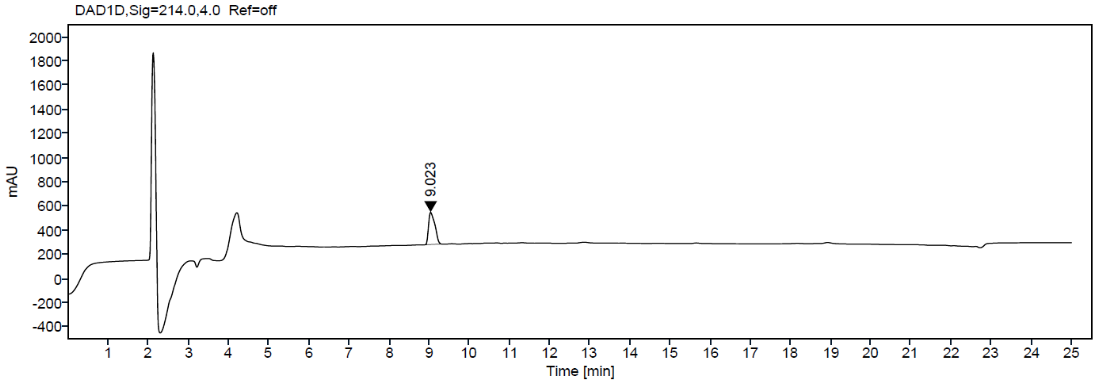

HPLC-UV chromatograms (214 nm) of crude of the reaction:

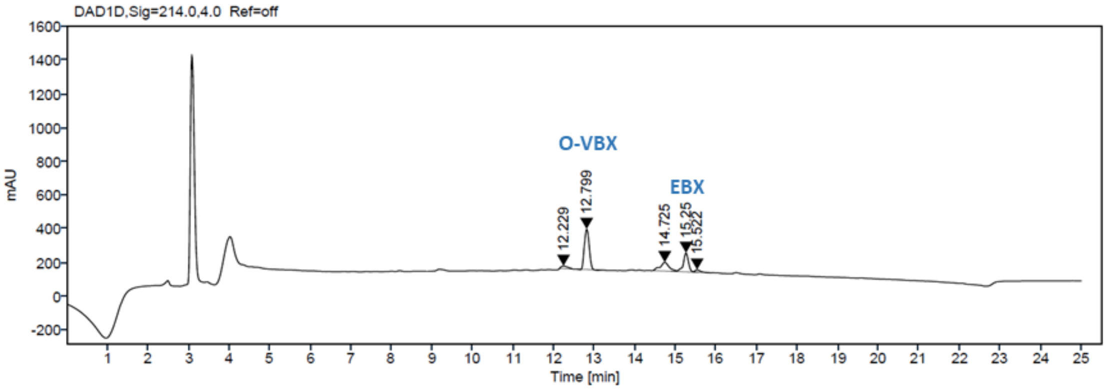

HPLC-MS chromatograms of crude of the reaction:

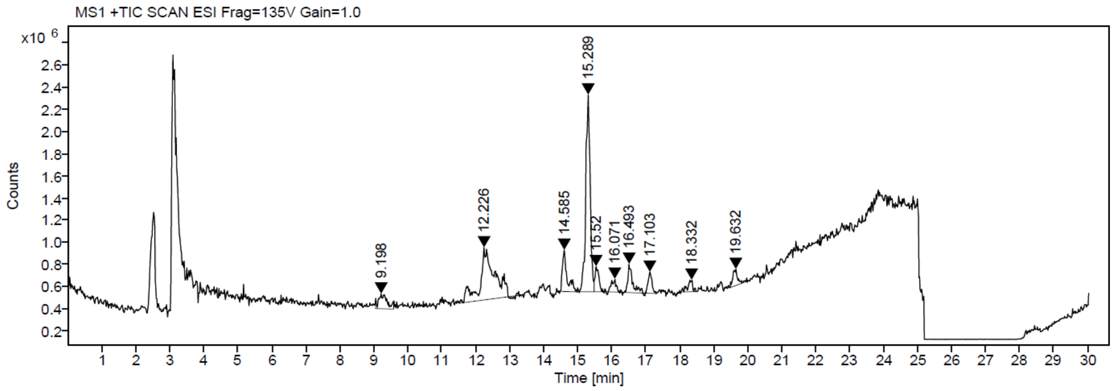

Yield based on HPLC-UV at 214 nm: 82%

Yield based on HPLC-MS: 76%

O-VBX (3rg)

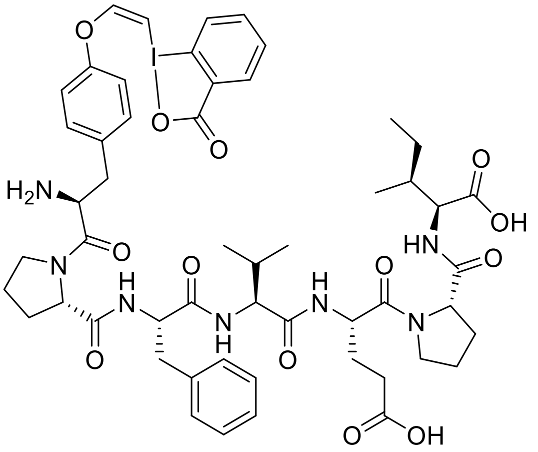

Starting from β-Casomorphin (**1r**) (0.864 mg, 1.00 μmol), O-VBX (**3rg**) was obtained in >76% yield (retention time 8.7 min).

HRMS (nanochip-ESI/LTQ-Orbitrap) m/z: $[M + H_2]^{+2}$ Calcd for $C_{53}H_{68}IN_7O_{13}^{+2}$ 568.6954; Found 568.6931.

MS/MS characterization:

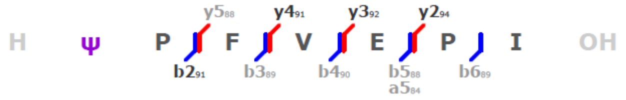

ψ = Tyr(C9H5IO2)

Sequence	Type	MF	m/z	Intensity	Similarity
**YPFVE**	b5	C42H47IN5O10(+1)	908.2362	103.01	0.88
PI	y2	C11H21N2O3(+1)	229.1547	50.61	0.94
**YP**	b2	C23H22IN2O5(+1)	533.0568	15.71	0.91
**YPFVEP**	b6	C47H54IN6O11(+1)	503.1481	12.30	0.89
**YPFV**	b4	C37H40IN4O7(+1)	779.1936	8.79	0.89
**YPFVE**	b5	C42H47IN5O10(+1)	454.6217	8.75	0.89
**YPFVE**	b5	C42H45IN5O9(+1)	890.2257	4.37	0.86
EPI	y3	C16H28N3O6(+1)	358.1973	4.13	0.91
EPI	y3	C16H26N3O5(+1)	340.1867	2.76	0.93
**YPF**	b3	C32H31IN3O6(+1)	680.1252	1.27	0.89
VEPI	y4	C21H37N4O7(+1)	457.2657	0.91	0.91
FVEPI	y5	C30H46N5O8(+1)	604.3341	0.89	0.88
**YPFVE**	a5	C41H47IN5O9(+1)	440.6243	0.74	0.84
**YPFV**	b4	C37H40IN4O7(+1)	390.1004	0.68	0.91

HPLC-UV chromatogram (214 nm) of a 2.00 mM solution of β-Casomorphin (**1r**) in 100 mM Tris buffer pH 9.0:

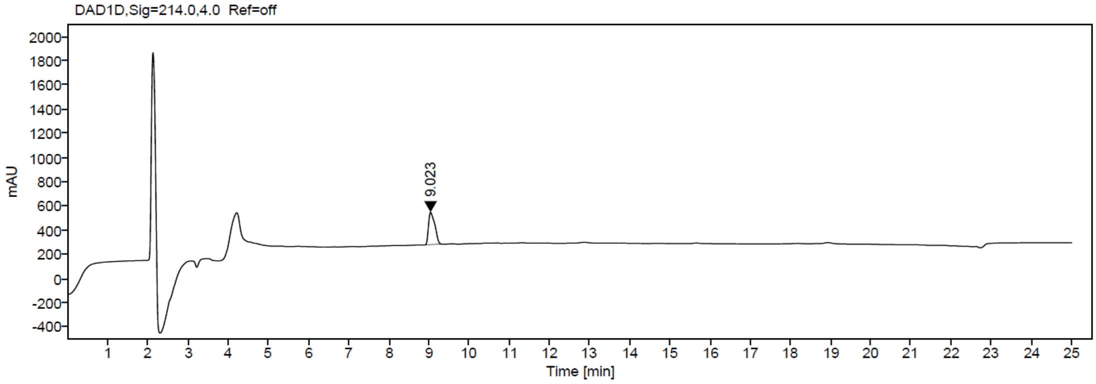

HPLC-UV chromatograms (214 nm) of crude of the reaction:

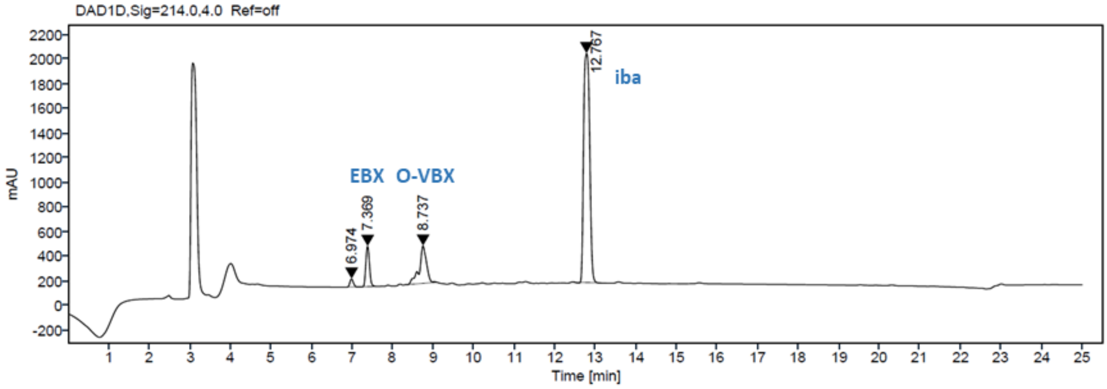

HPLC-MS chromatograms of crude of the reaction:

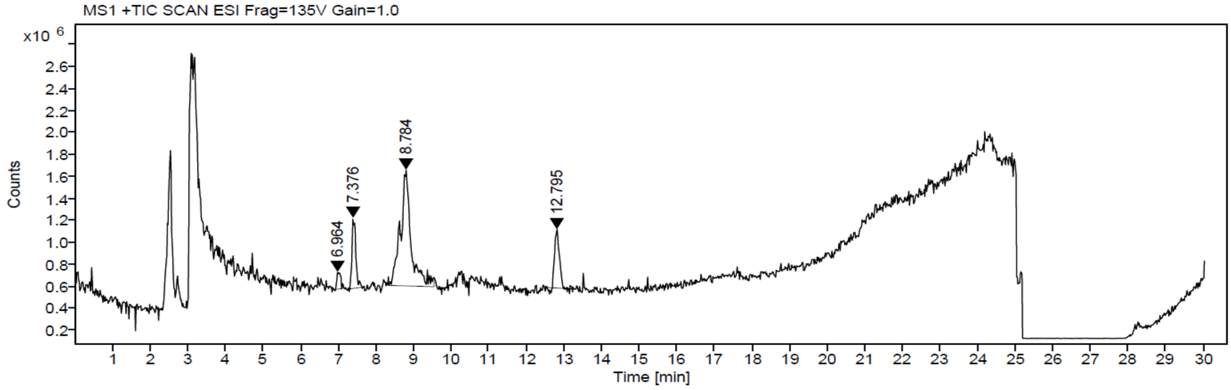

O-VBX (3rj)

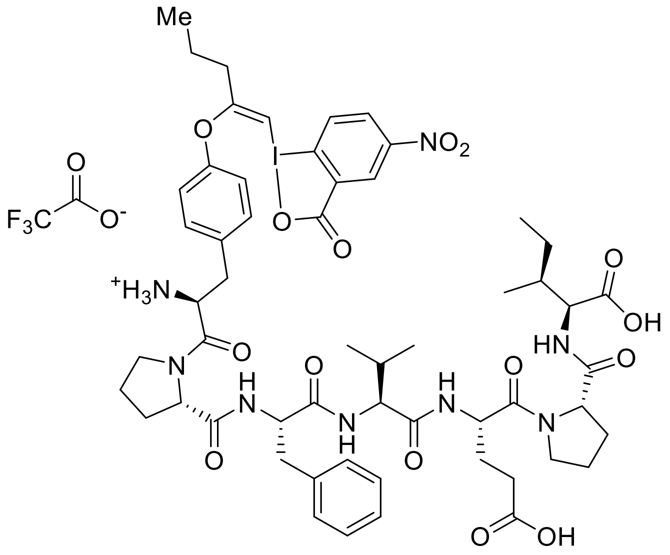

Starting from β-Casomorphin (**1r**) (0.864 mg, 1.00 μmol), O-VBX (**3rj**) was obtained in 11% yield (retention time 10.3 min).

HRMS (ESI/QTOF) m/z: $[M + H]^+$ Calcd for $C_{56}H_{72}IN_8O_{15}^+$ 1223.4156; Found 1223.4230.

HPLC-UV chromatogram (214 nm) of a 2.00 mM solution of β-Casomorphin (**1r**) in 100 mM Tris buffer pH 9.0:

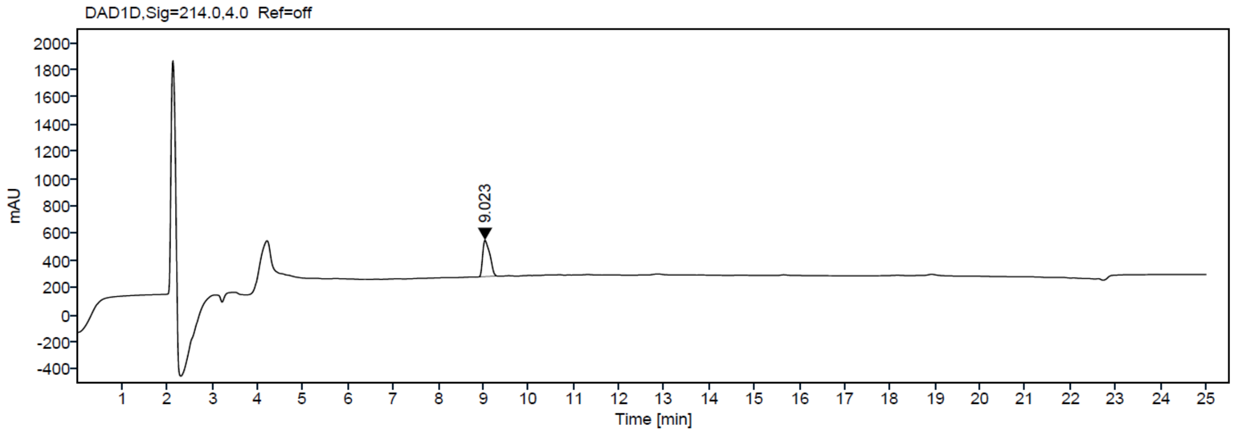

HPLC-UV chromatograms (214 nm) of crude of the reaction:

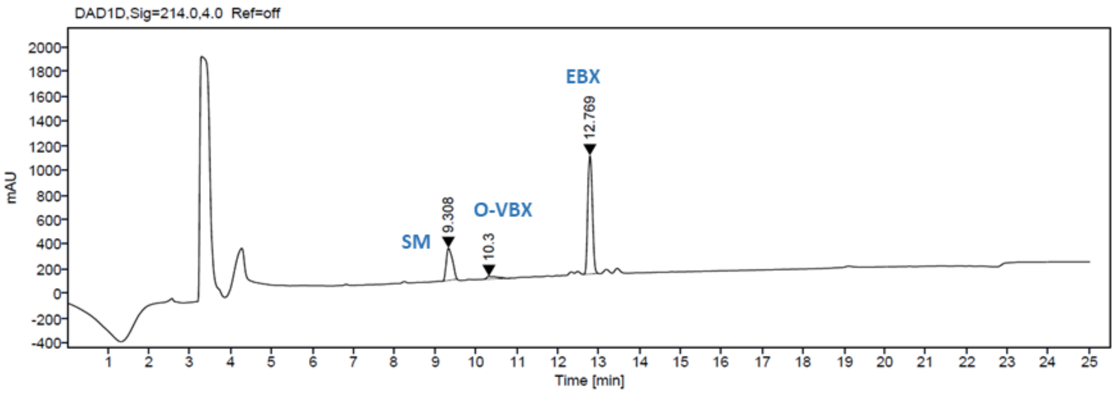

HPLC-MS chromatograms of crude of the reaction:

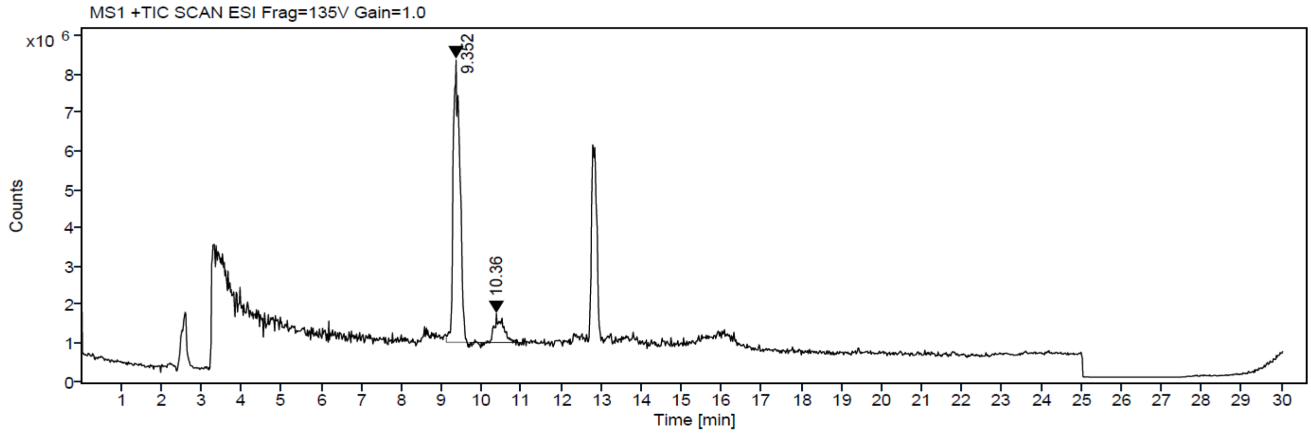

Yield based on HPLC-UV at 214 nm: 12%

Yield based on HPLC-MS: 11%

O-VBX (3rk)

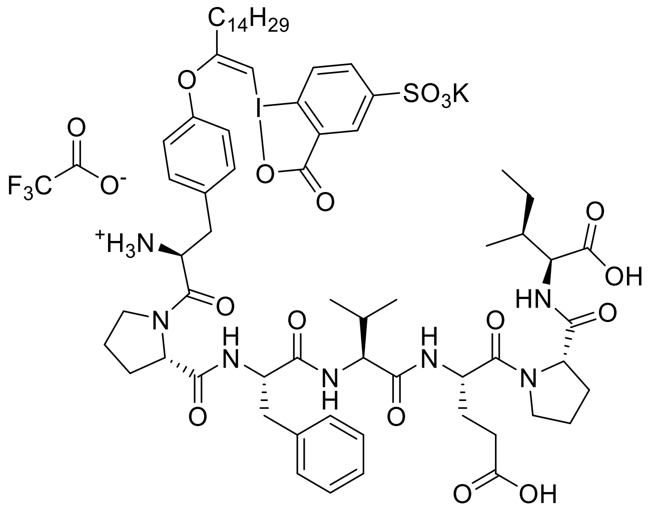

Starting from β-Casomorphin (**1r**) (0.864 mg, 1.00 μmol), O-VBX (**3rk**) was obtained in 3% yield (retention time 13.2 min).

HRMS (ESI/QTOF) m/z: $[M + K_{-1}]^-$ Calcd for $C_{67}H_{93}IN_7O_{16}S^-$ 1410.5450; Found 1410.5444.

HPLC-UV chromatogram (214 nm) of a 2.00 mM solution of β-Casomorphin (**1r**) in 100 mM Tris buffer pH 9.0:

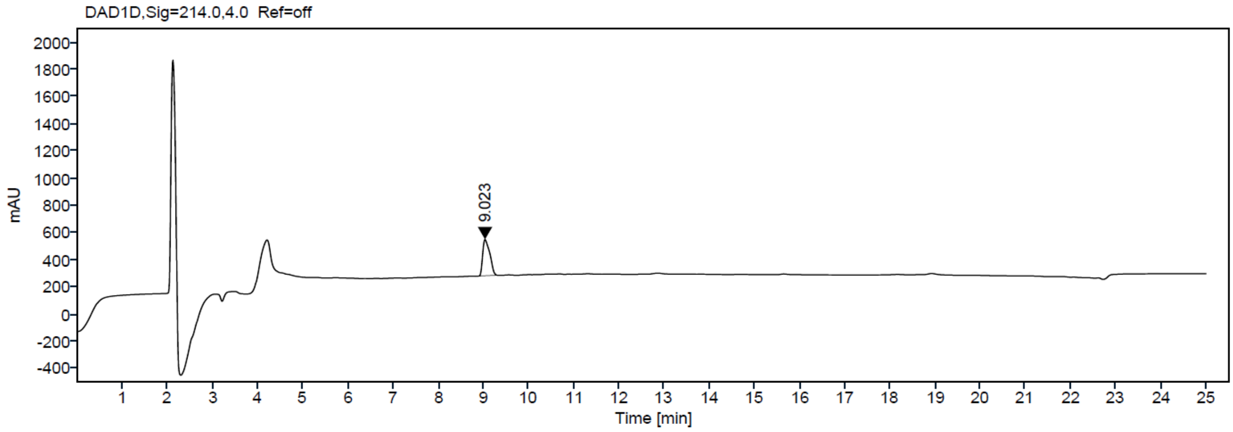

HPLC-UV chromatograms (214 nm) of crude of the reaction:

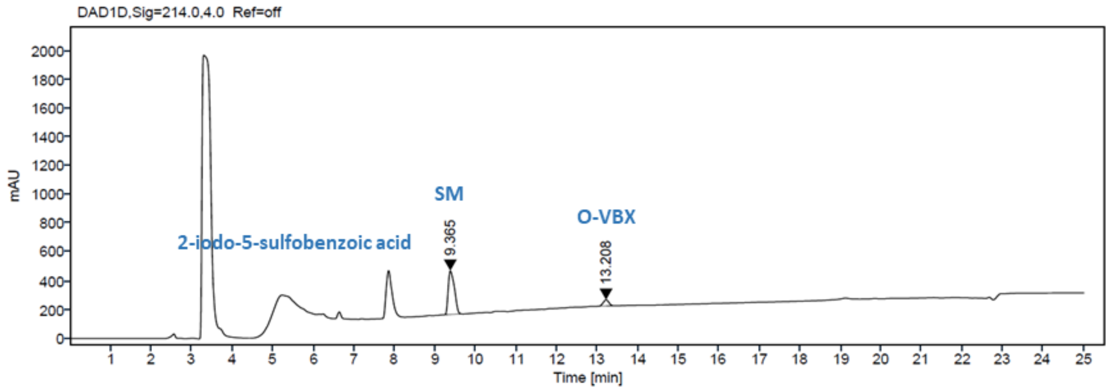

HPLC-MS chromatograms of crude of the reaction:

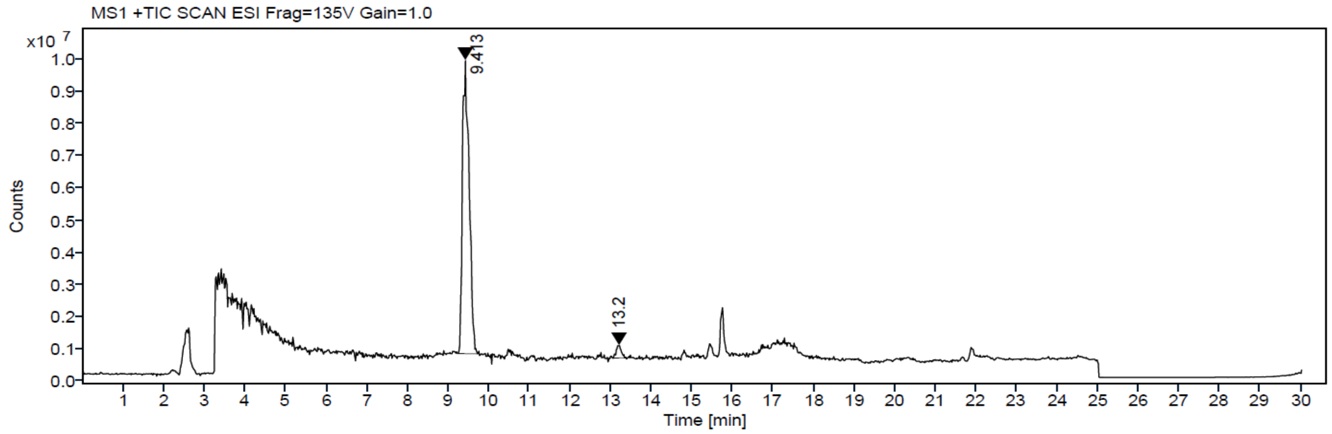

Yield based on HPLC-UV at 214 nm: 10%

Yield based on HPLC-MS: 3%

O-VBX (3we)

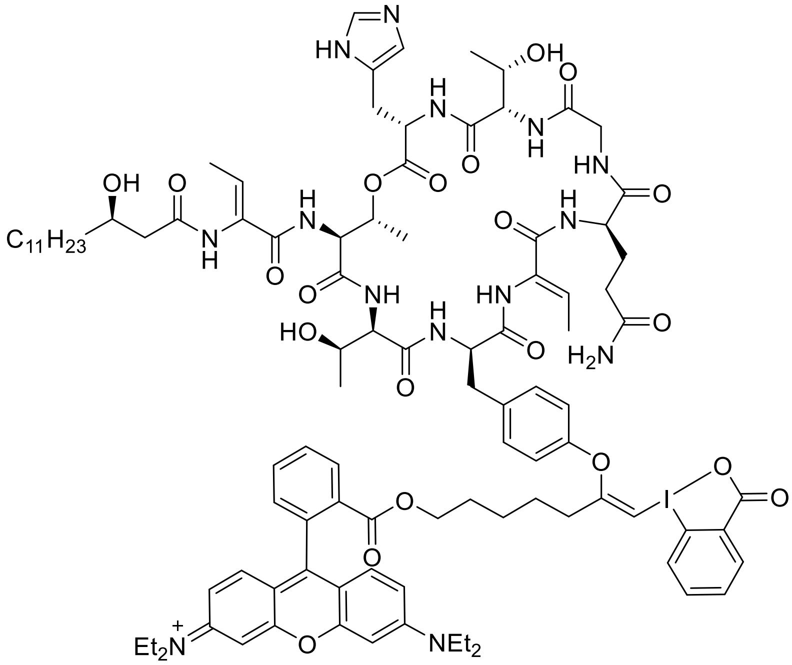

Starting from Jaricin (**3w**, 5.00 µmol), O-VBX (**3wa**) was obtained in 39% yield based on HPLC-MS (retention time 13.3 min) and 20% isolated yield (2.00 mg, 1.00 µmol).

HPLC-UV chromatogram (214 nm) of crude of the reaction:

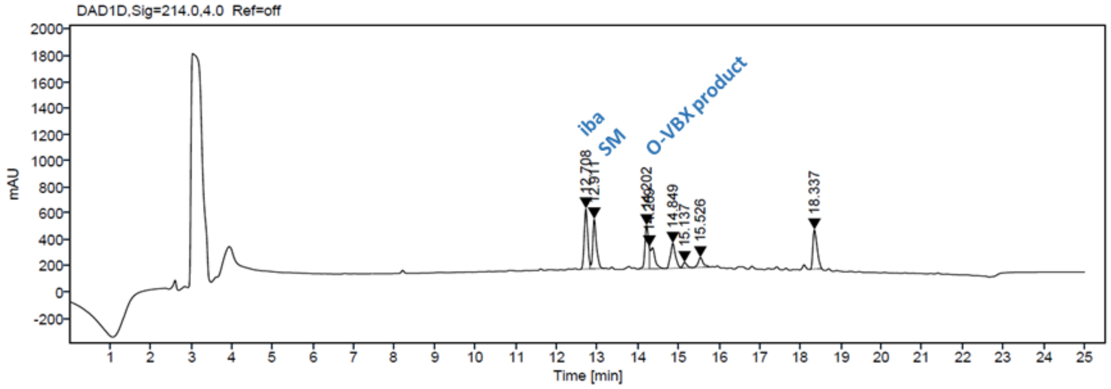

HPLC-MS chromatogram of crude of the reaction:

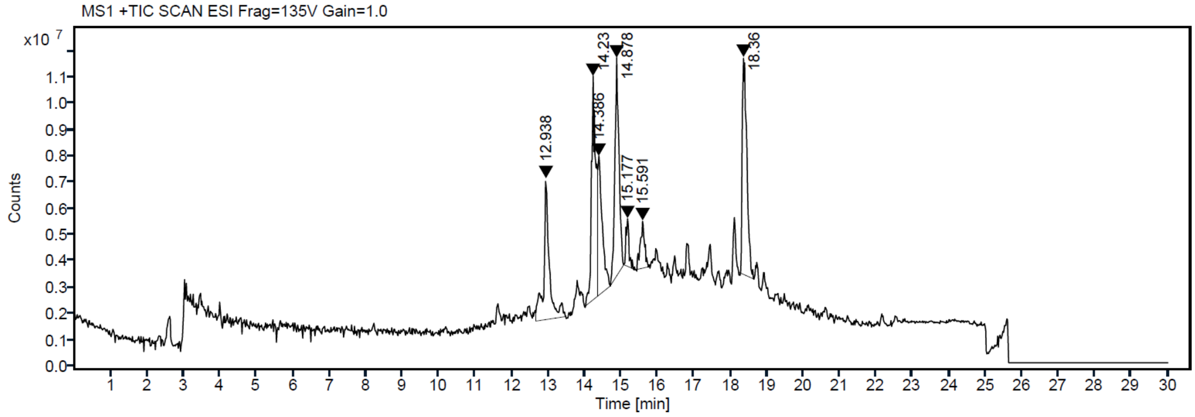

The rhodamine-jagaricin-conjugate was purified using semi-preparative HPLC (HPLC column Luna® 5 µm C18(2), 100 Å, 250 x 10 mm). The following method was used: flow rate of 5 mL per minute; 0-5 min: 10 % (v/v) acetonitrile in water containing 0.1 % formic acid; 5-7 min: linear gradient from 10 % to 45 % (v/v) acetonitrile in water containing 0.1 % formic acid; 7-20 min: isocratic gradient of 45 % acetonitrile in water containing 0.1 % formic acid. The retention time (t_R) of the rhodamine-jagaricin-conjugate was t_R = 12.4 min (0.6 mg, purple solid). The linear rhodamine-jagaricin-conjugate eluted at t_R = 14.9 min (0.1 mg, purple solid).

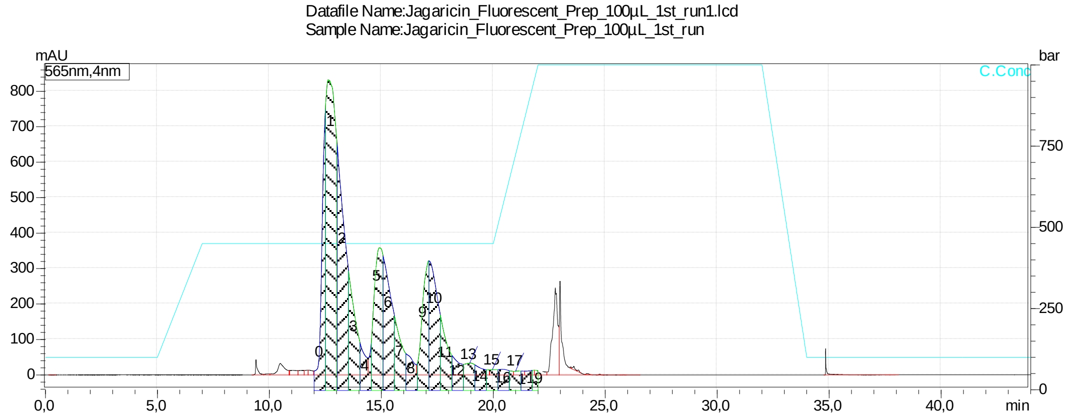

For NMR experiments the rhodamine-jagaricin-conjugate (0.6mg) was dissolved in 600 µL d_4-methanol (Deutero GmbH) and a ^{1}H- as well as a 2D-NMR experiment (COSY) were recorded on a Bruker Avance™ III 500 using standard pulse sequences. Topspin 3.2 (Bruker) was used for the analysis of the NMR data. The solvent peak of d_4-MeOH was used for calibration (3.31 ppm for ^{1}H-NMR spectra). The signal fine structures are described, using the following abbreviations: s (singlet), d (doublet), t (triplet), q (quartet) and m (multiplet) as well as combinations thereof. Coupling constants are given in Hz.

High-resolution mass (HRMS) and tandem mass (HRMS^2) spectrometry data of the pure compounds was obtained by using a LC-ESI-HRMS system (Accela UPLC system by Thermo Scientific) equipped with an Accucore C18 column (100 x 2.1 mm, particle size 2.6 µm) coupled with a QExactive Orbitrap mass spectrometer (Thermo Scientific) with an electrospray ionization (ESI) source (HCD was set to 35% and either the singly or doubly charged m/z values were used for fragmentation). MS data was visualized using Xcalibur. Chemical structures were drawn using ChemDraw version 20. The conjugates yielded complex fragmentation patterns. Crucial fragment ions could be identified and showed only small deviations from the calculated m/z ratios (Δ < 5 ppm).

Analytical data for the rhodamine-jagaricin-conjugate: **^{1}H NMR (500 MHz, d_4-MeOH):** δ 8.70 (bs, 1H), 8.34 (d, 1H, J = 7.9 Hz), 8.26-8.22 (m, 1 H), 7.88 (t, 1H, J = 7.5 Hz), 7.81 (t, 1H, J = 7.7 Hz), 7.73-7.69 (m, 1H), 7.65-7.59 (m, 2H), 7.42 (d, 1H, J = 7.5 Hz), 7.32 (s, 1H) 7.23 (d, 2H, J = 8.2 Hz), 7.10-7.05 (m, 2H), 7.02-6.96 (m, 2H), 6.93 (s, 2H), 6.85 (d, 2H, J = 8.2 Hz), 6.43 (bs, 1H), 6.18 (s, 1H), 5.71 (q, 1H, J = 7.5 Hz), 5.50 (bs, 1H), 4.63 (d, 1H, J = 3.5 Hz), 4.32-4.21 (m, 2H), 4.18-4.06 (m, 4H), 4.04-3.89 (m, 4H), 3.65 (q, 8H, J = 7.0 Hz), 2.59 (t, 2H, J = 7.1 Hz), 2.45-2.38 (m, 3H), 2.34 (dd, 1H, J_1 = 9.2 Hz, J_2 = 14.2 Hz), 2.19-2.10 7(m, 2H), 1.87 (d, 3H, J = 7.3 Hz), 1.63 (d, 3H, J = 5.5 Hz), 1.56-1.08 (m, 47H), 0.89 (t, 3H, J = 6.7 Hz); HRMS (ESI+) m/z 982.9244 $[M+H]^{2+}$ (calcd for $C_{98}H_{129}IN_{14}O_{21}$, 982.9262) and 655.6190 $[M+2H]^{3+}$ (calcd for $C_{98}H_{129}IN_{14}O_{21}$, 655.6199).

Analytical data for the hydrolyzed rhodamine-jagaricin-conjugate: **HRMS** (ESI+) m/z 991.9302 $[M+H]^{2+}$ (calcd for $C_{98}H_{131}IN_{14}O_{22}$, 991.9315) and 661.6226 $[M+2H]^{3+}$ (calcd for $C_{98}H_{129}IN_{14}O_{21}$, 661.6234).

HRMS^2-Analysis of the rhodamine-jagaricin-conjugate

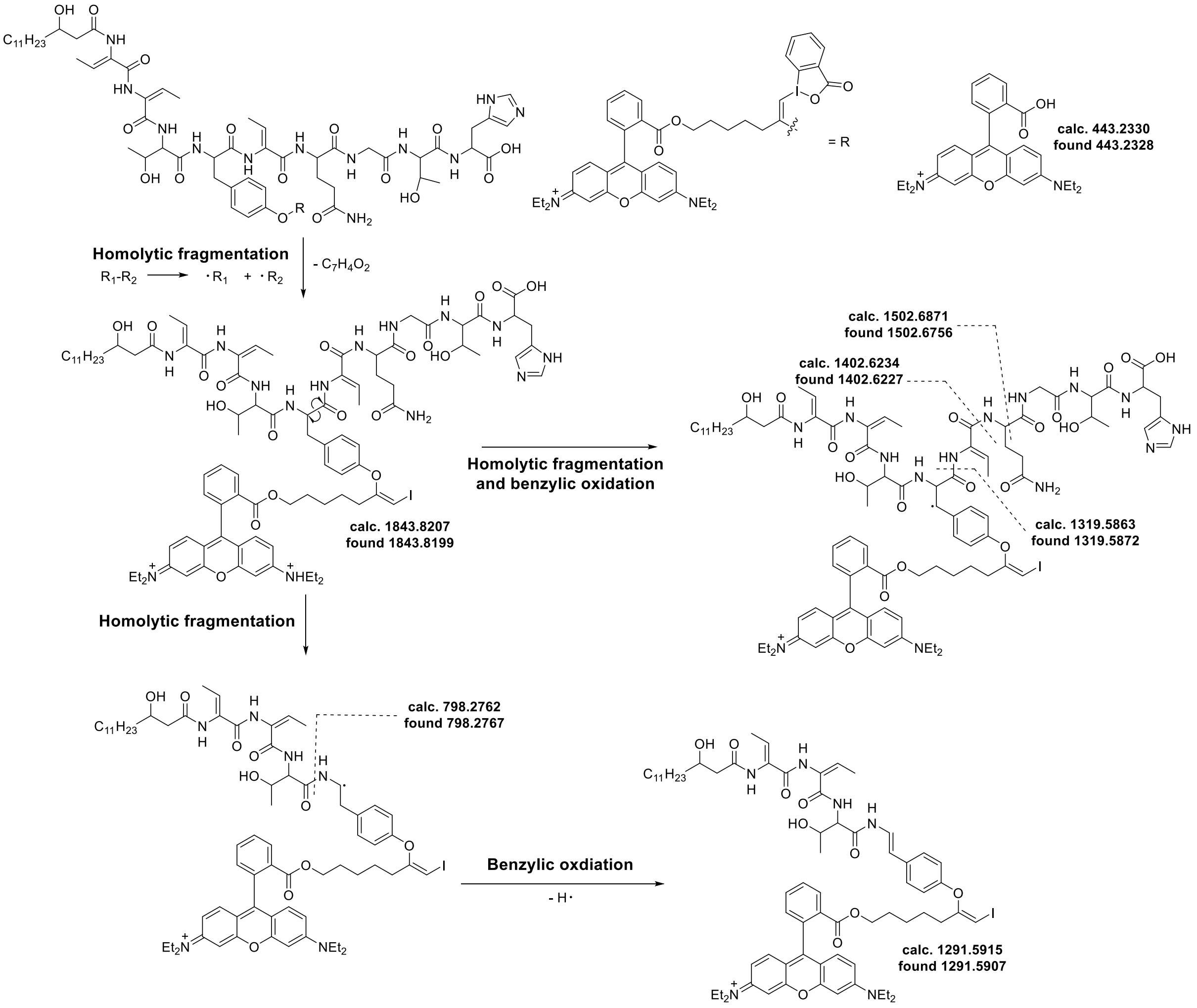

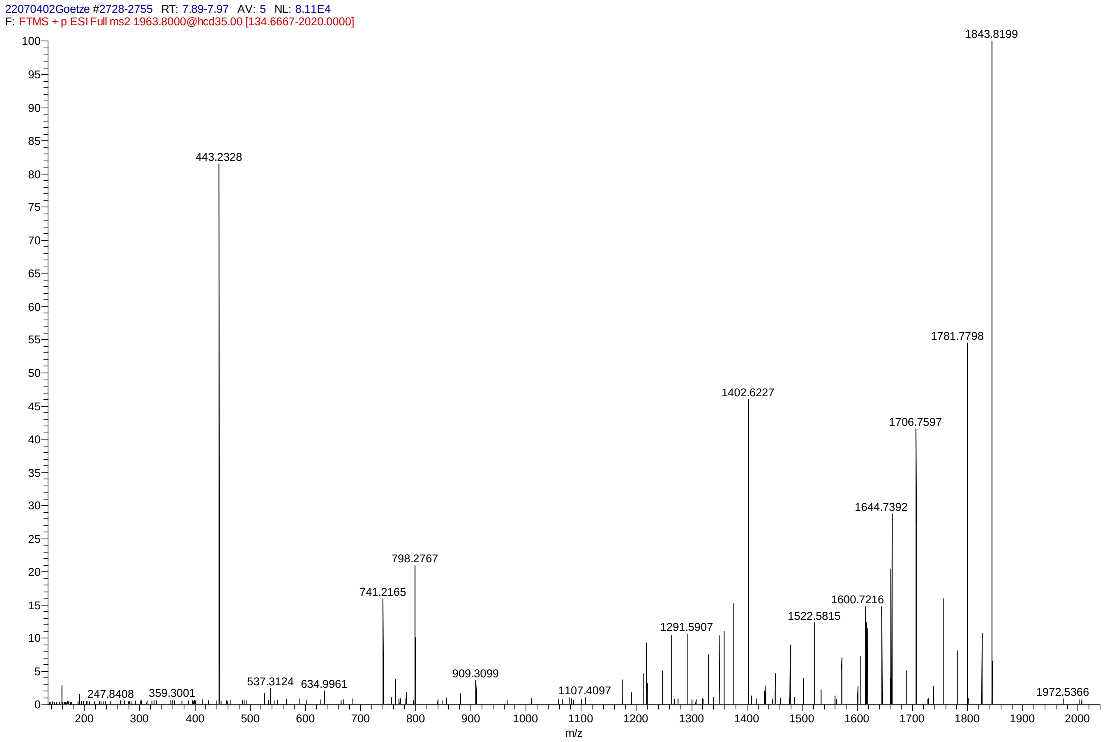

HRMS^2-Analysis of the hydrolysed rhodamine-jagaricin-conjugate

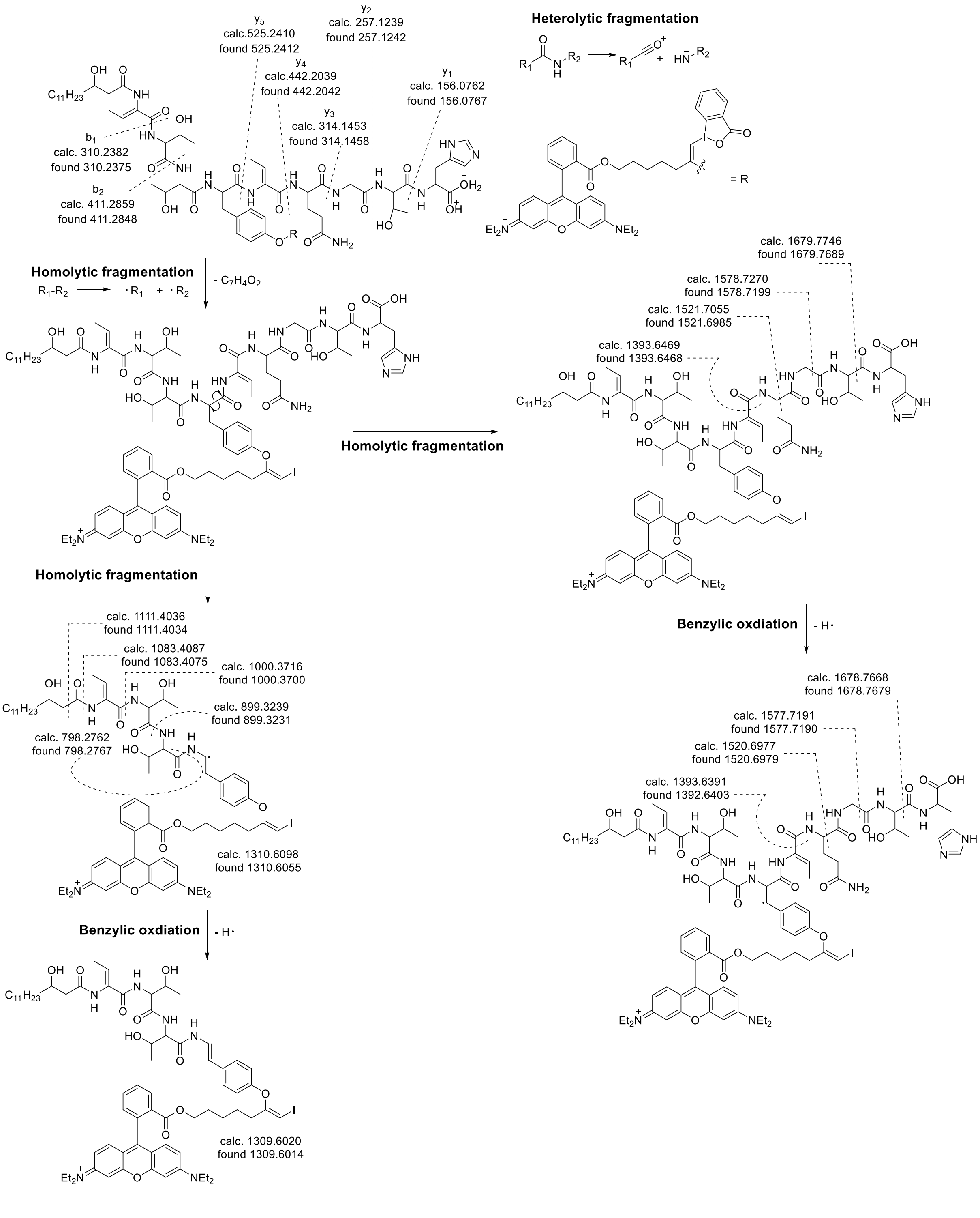

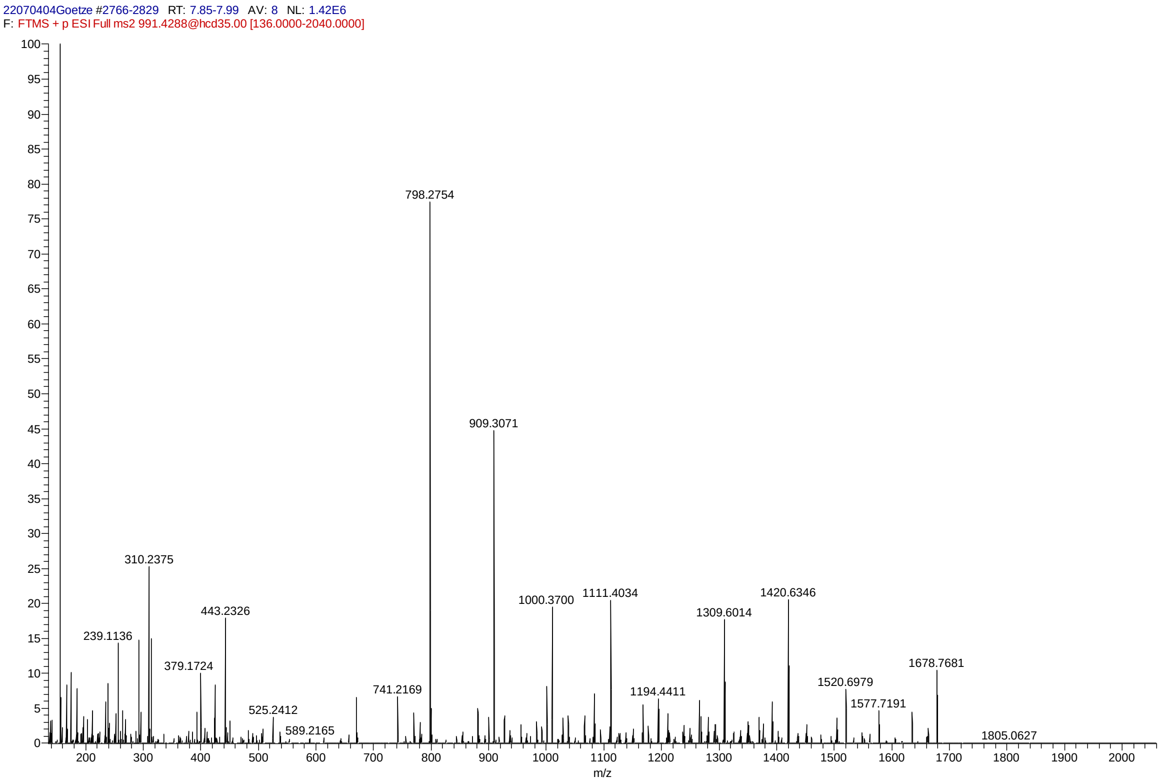
22070404Goetze #2766-2829 RT: 7.85-7.99 AV: 8 NL: 1.42E6
F: FTMS + p ESI Full ms2 991.4288@hcd35.00 [136.0000-2040.0000]
798.2754
909.3071
310.2375
239.1136
443.2326
379.1724
525.2412
589.2165
741.2169
1000.3700
1111.4034
1194.4411
1309.6014
1420.6346
1520.6979
1577.7191
1678.7681
1805.0627
m/z

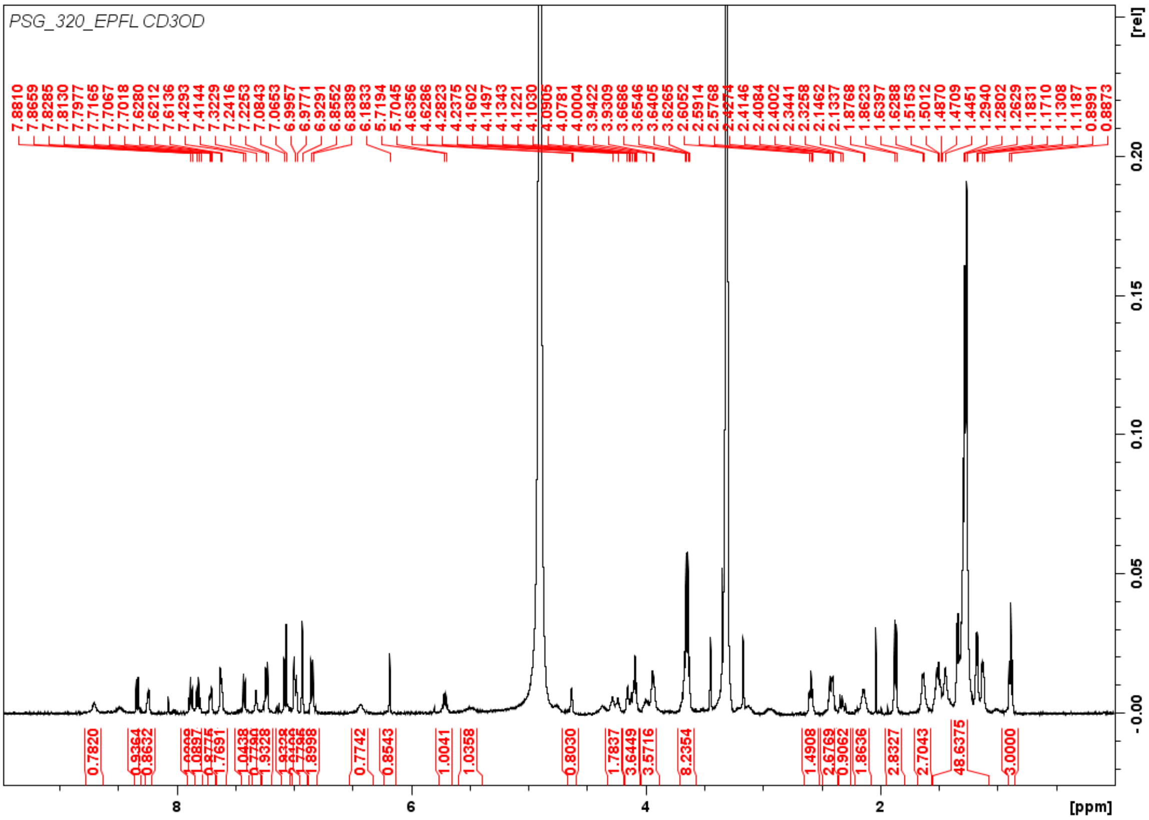
PSG_320_EPFL CD3OD
[rel]
[ppm]

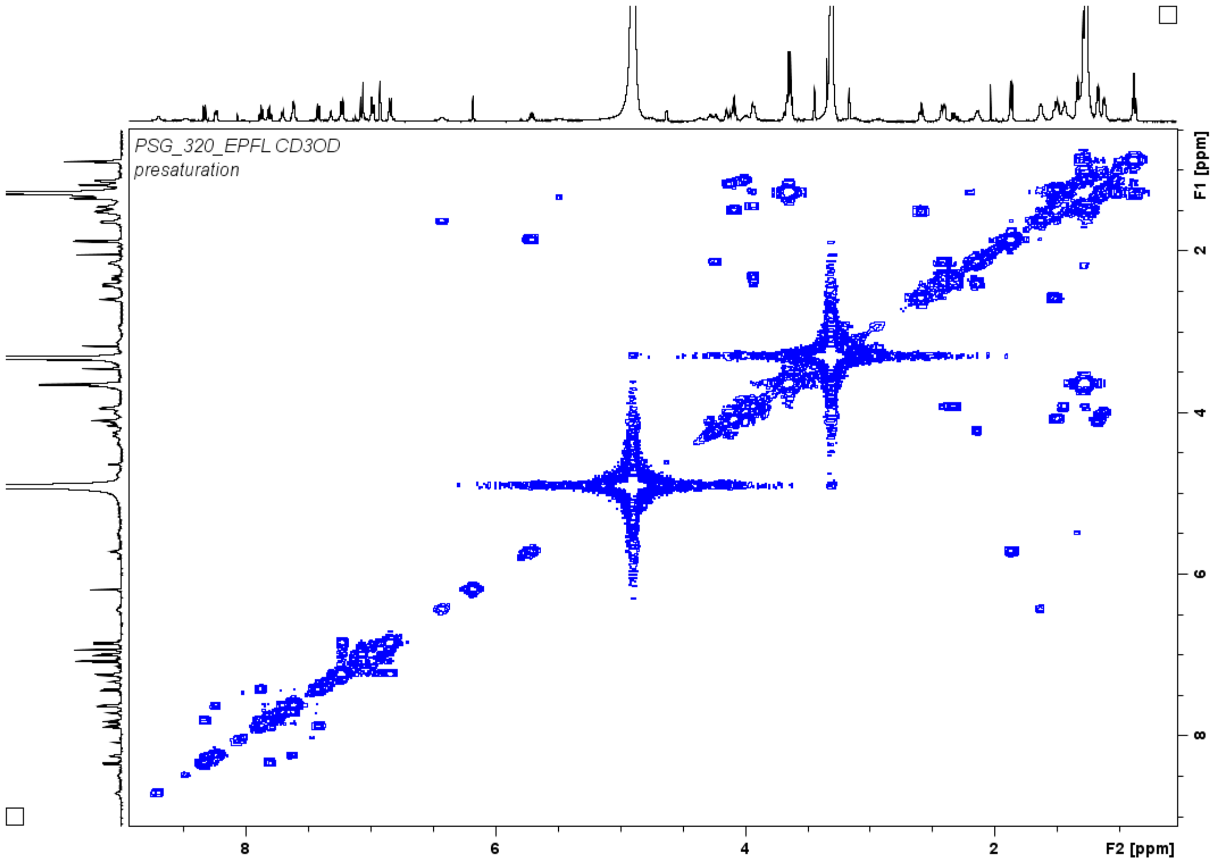
PSG_320_EPFL CD3OD
presaturation
F1 [ppm]
2
4
6
8
F2 [ppm]
8
6
4
2

9. Click chemistry

c. Synthesis of Click reagents

(1*R*,8*S*,9*R*,*Z*)-Ethyl bicyclo[6.1.0]non-4-ene-9-carboxylate (exo-S19), and (1*R*,8*S*,9*S*,*Z*)-ethyl bicyclo[6.1.0]non-4-ene-9-carboxylate (endo-S19)

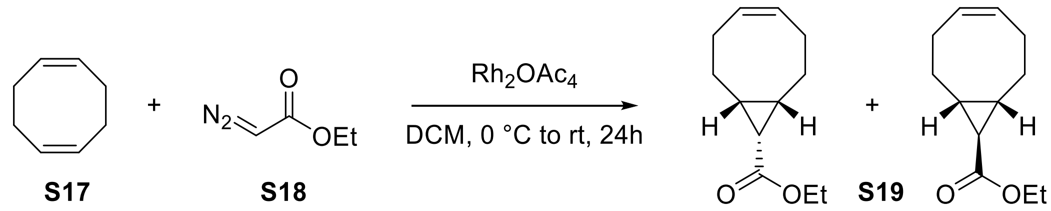

Following a reported procedure,[24] a solution of 1,5-cyclooctadiene (**S17**, 19.6 mL, 160 mmol) and $Rh_2(OAc)_4$ (380 mg, 860 µmol) DCM (10 mL) was added dropwise in 3 h to a solution of ethyl diazoacetate **S18** (2.1 mL, 20 mmol) in DCM (10 mL). This solution was stirred for 40 h at rt. DCM was evaporated and the excess of cyclooctadiene was removed by filtration over a glass filter filled with silica and elution with EtOAc:heptane, 1:200 (400 mL). The filtrate was concentrated in vacuo and the residue was purified by column chromatography on silica gel (EtOAc:heptane, 1:20) to afford exo-**S19** (1.10 g, 5.66 mmol, 28%) and endo-**S19** (2.24 g, 11.5 mmol, 58%) as colorless oils.

exo-**S19**: **^{1}H NMR** ($CDCl_3$, 400 MHz): δ 5.68-5.60 (m, 2H, C*H*-BCN), 4.10 (q, *J* = 7.2 Hz, 2H, C*H₂*-ester), 2.35-2.27 (m, 2H, C*H*-BCN, C*H*$_2$-BCN) 2.24-2.16 (m, 2H, C*H*$_2$-BCN), 2.13-2.04 (m, 2H, C*H*-BCN, C*H*$_2$-BCN) 1.59-1.53 (m, 2H, C*H*-BCN, C*H*$_2$-BCN), 1.53-1.43 (m, 2H, C*H*$_2$-BCN), 1.25 (t, *J* = 7.2 Hz, 3H, C*H*$_3$-ester), 1.18 (t, *J* = 4.8 Hz, 1H, C*H*$_2$-BCN). **^{13}C NMR** ($CDCl_3$, 100 MHz): δ 174.3, 129.8, 60.1, 28.2, 27.8, 27.6, 26.6, 14.2.

endo-**S19**: **^{1}H NMR** ($CDCl_3$, 400 MHz): δ 5.65-5.57 (m, 2H, C*H*-BCN), 4.12 (q, *J* = 7.2 Hz, 2H, C*H*$_2$-ester), 2.53-2.46 (m, 2H, C*H*-BCN, C*H*$_2$-BCN), 2.25-2.16 (m, 2H, C*H*$_2$-BCN), 2.10-2.01 (m, 2H, C*H*$_2$-BCN), 1.87-1.79 (m, 2H, C*H*-BCN, C*H*$_2$-BCN), 1.70 (t, *J* = 8.8 Hz, 1H C*H*-BCN), 1.43-1.34 (m, 2H C*H*$_2$-BCN,), 1.26 (t, *J* = 7.2 Hz, 3H, C*H*$_3$-ester). **^{13}C NMR** ($CDCl_3$, 100 MHz): δ 172.2, 129.4, 59.7, 27.0, 24.1, 22.6, 21.2, 14.4.

Spectroscopic data was consistent with the values reported in literature.[24]

[24] J. Dommerholt, S. Schmidt, R. Temming, L. J. A. Hendriks, F. P. J. T. Rutjes, J. C. M. van Hest, D. J. Lefeber, P. Friedl, F. L. van Delft, *Angew. Chem. Int. Ed.* **2010**, *49*, 9422–9425.

(1*R*,8*S*,9*S*)-Bicyclo[6.1.0]non-4-yn-9-ylmethanol (endo-37)

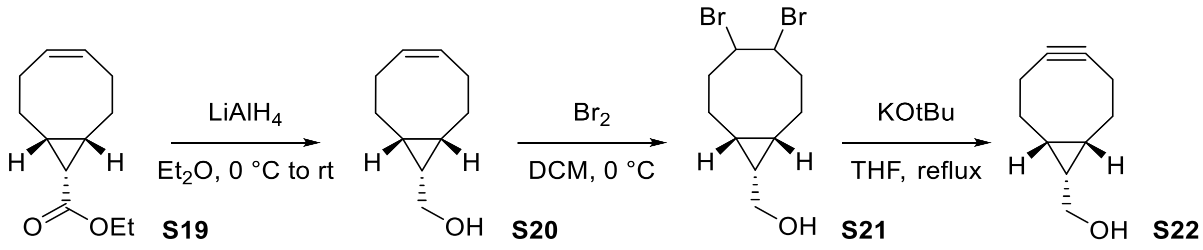

Following a reported procedure,[2424] a suspension of LiAlH4 (90.0 mg, 2.34 mmol) in Et_2O (10 mL) was added dropwise at 0 °C a solution of endo-**S19** (520 mg, 2.68 mmol) in Et_2O (10 mL). This suspension was 3 stirred for 15 min at rt, then cooled down to 0 °C, and water was added carefully until the grey solid had turned into white. Na_2SO_4 (2 g) was added, the solid was filtered off and washed thoroughly with Et_2O (100 mL). The filtrate was concentrated in vacuo.

Without further purification the alcohol was dissolved in DCM (20 mL). At 0 °C a solution of Br_2 (151 µL, 2.94 mmol) in DCM (2 mL) was added dropwise until the yellow color persisted. The reaction mixture was quenched with a 10% $Na_2S_2O_3$-solution (5 mL), and extracted with DCM (2 x 20 mL). The organic layer was dried (Na_2SO_4) and concentrated in vacuo to afford the dibromide **S21** (833 mg, quant.).

Without further purification the dibromide (1.4 mg, 4.49 mmol) was dissolved in THF (30 mL). A solution of KO*t*Bu (1.61 , 14.4 mmol) was added dropwise at 0 °C. Then the solution was refluxed for 2 h. After cooling down to rt the mixture was quenched with saturated NH_4Cl-solution (20 mL), and extracted with DCM (3 x 20 mL). The organic layer was dried (Na_2SO_4) and concentrated in vacuo. The residue was purified by column chromatography on silica gel (EtOAc:pentane, 1:1) to afford endo-**S22** (400 mg, 2.66 mmol, 59%) as a white solid.

^{1}H NMR ($CDCl_3$, 400 MHz): δ 3.73 (d, *J* = 8.0 Hz, 2H, C*H₂*OH), 2.35-2.20 (m, 6H, C*H₂*-BCN), 1.66-1.56 (m, 2H, C*H₂*-BCN), 1.39- 1.30 (m, 1H, C*H*-BCN), 1.18 (bs, 1H, O*H*), 0.99-0.90 (m, 2H C*H*-BCN). **^{13}C NMR** ($CDCl_3$, 100 MHz): δ 98.4, 59.3, 28.5, 21.0, 20.9, 19.5.

Spectroscopic data was consistent with the values reported in literature.[24]

(1*R*,8*S*,9*S*)-Bicyclo[6.1.0]non-4-yn-9-ylmethyl (4-nitrophenyl) carbonate (S23)

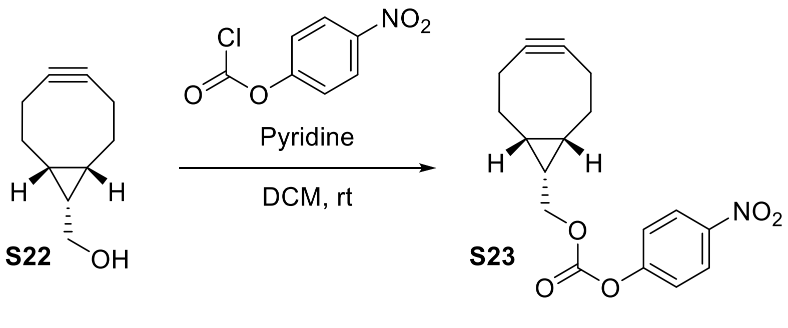

Following a reported procedure,[2424] to a solution of endo-**S22** (350.0 mg, 2.33 mmol) in DCM (54.0 mL) was added pyridine (471 µL, 5.83 mmol) and p-NO_2PhOC(O)Cl (587 mg, 2.91 mmol). After stirring for 15 min at rt, the mixture was quenched with saturated NH_4Cl-solution (50 mL) and extracted with DCM (3 x 5 mL). The organic layer was dried (Na_2SO_4) and concentrated in vacuo. The residue was purified by column chromatography on silica gel (EtOAc:pentane, 1:5) to afford **S23** (320.0 mg, 1.01 mmol, 44%) as a white solid.

^{1}H NMR ($CDCl_3$, 400 MHz): δ 8.28 (d, *J* = 9.2 Hz, 2H, Ar*H*), 7.40 (*J* = 9.6 Hz, 2H, Ar*H*), 4.41 (d, *J* = 8.4 Hz, 2H, C*H_2*O), 2.37-2.22 (m, 6H, C*H_2*-BCN), 1.67-1.57 (m, 2H, C*H_2*-BCN), 1.56-1.47 (m, 1H, C*H*-BCN), 1.11-1.02 (m, 2H, C*H*-BCN). **^{13}C NMR** ($CDCl_3$, 100 MHz): δ 155.6, 152.5, 145.3, 125.3, 121.7, 98.7, 68.0, 29.0, 21.3, 20.5, 17.2.

Spectroscopic data was consistent with the values reported in literature.[24]

(1*R*,8*S*,9*S*)-bicyclo[6.1.0]non-4-yn-9-ylmethyl 3,6,9-trioxa-12-azadodecylcarbamate (10a)

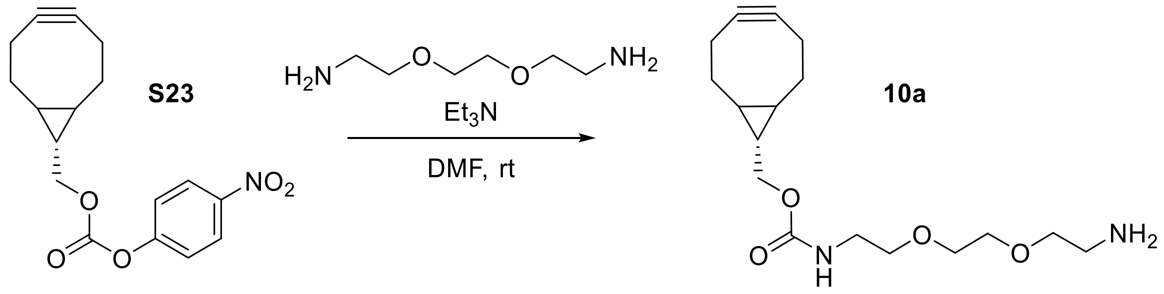

Following a reported procedure,[24] to a solution of **S23** (320 mg, 1.02 mmol) in DMF (28 mL) was added 1,8-diamino-3,6-dioxaoctane (889 µL, 6.09 mmol, 6.00 equiv.) and NEt_3 (424 µL, 3.05 mmol, 3.00 equiv.) and the reaction mixture was stirred at rt for 15 min. The mixture was concentrated under reduced pressure, taken up in DCM and extracted with 1 N NaOH, followed by water. The combined aqueous layers were extracted once with DCM. The combined organic layers were dried over Na_2SO_4 and concentrated in vacuo. Silica gel column chromatography (DCM:MeOH, 10:1, 1% Et_3N) and concentration in vacuo gave compound **10a** (300 mg, 0.924 mmol, 91%) as a slightly yellow oil.

^{1}H NMR ($CDCl_3$, 400 MHz): δ 5.34 (bs, 1H, N*H*), 4.15 (d, 2H, *J* = 8.0 Hz, C*H_2*O), 3.62 (s, 4H, C*H_2*-PEG), 3.58-3.48 (m, 4H, C*H_2*-PEG), 3.38 (m, 2H, C*H_2*-PEG), 2.89 (t, 2H, *J* = 5.2 Hz, C*H_2*-PEG), 2.32-2.19 (m, 6H, C*H_2*-BCN), 1.64-1.51 (m, 2H, C*H*-BCN), 1.40-1.31 (m, 1H, C*H*-BCN), 0.96-0.88 (m, 2H, C*H_2*-BCN). **^{13}C NMR** ($CDCl_3$, 100 MHz): δ 157.0, 98.8, 73.4, 70.3, 70.2, 70.1, 62.7, 41.7, 40.8, 29.1, 21.4, 20.1, 17.8. **HRMS** (ESI^+) m/z calcd for $C_{17}H_{28}N_2O_4$ $(M + H)^+$: 325.2122, found: 325.2120.

Spectroscopic data was consistent with the values reported in literature.[24]

BCN-biotin (10b)

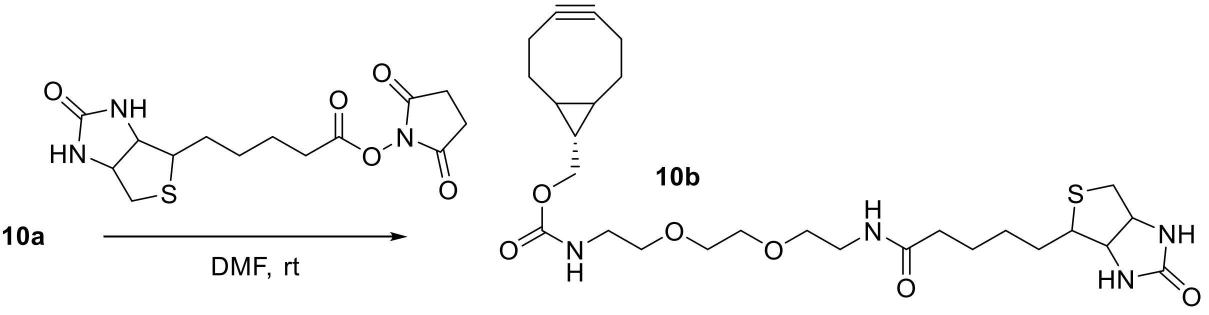

Following a reported procedure,[25] to a solution of **10a** (68.1 mg, 210 µmol, 1.00 equiv.) in DMF (1.00 mL) was added Biotin NHS (143 mg, 420 µmol, 2.00 equiv.). After 16 hours, the reaction was diluted with DCM and evaporated under reduced pressure. The residue was purified by reverse phase preparative HPLC (water 0.1 % TFA to 95:5 ACN:water 0.1 % TFA) to afford **10b** (51.0 mg, 92.6 µmol, 44%) as a white solid after lyophilization.

^{1}**H NMR** ($CDCl_3$, 400 MHz) δ 6.66 (s, 2H, N*H*), 5.77 (s, 1H, N*H*), 5.40 (s, 1H, N*H*), 4.48 (t, *J* = 6.0 Hz, 1H, C*H*-Biotin), 4.29 (t, *J* = 5.9 Hz, 1H, C*H*-Biotin), 4.13 (d, *J* = 7.8 Hz, 2H, CH_2-BCN) 3.59 (s, 4H, CH_2-PEG), 3.55 (m, 4H, CH_2-PEG), 3.43 (m, 3H, CH_2-PEG, C*H*-Biotin), 3.35 (m, 2H, CH_2-PEG), 3.12 (dd, *J* = 6.9, 11.6 Hz, 1H, CH_2-Biotin), 2.88 (dd, *J* = 4.7, 12.7 Hz, 1H, CH_2-Biotin), 2.72 (d, *J* = 12.8 Hz, 1H, C*H*-BCN), 2.11-2.34 (m, 6H, CH_2-Biotin, CH_2-BCN), 1.48-1.76 (m, 6H, CH_2-Biotin, CH_2-BCN), 1.28-1.48 (m, 4H, CH_2-Biotin, C*H*-BCN), 1.23 (s, 1H, CH_2-Biotin), 0.92 (m, 1H, CH_2-Biotin). ^{13}**C NMR** ($CDCl_3$, 100 MHz): δ 173.3, 164.1, 156.8, 98.7, 70.0, 62.7, 61.7, 60.1, 55.6, 40.7, 40.1, 39.0, 35.9, 33.2, 29.6, 29.0, 28.2, 28.0, 25.5, 22.8, 21.3, 20.0, 17.7; **HRMS** (ESI-TOF MS) m/z 551.2903 $(M+H)^+$ calculated for $C_{27}H_{43}N_4O_6$, measured 551.2900.

Spectroscopic data was consistent with the values reported in literature.[25]

BCN-fluorescein (10c)

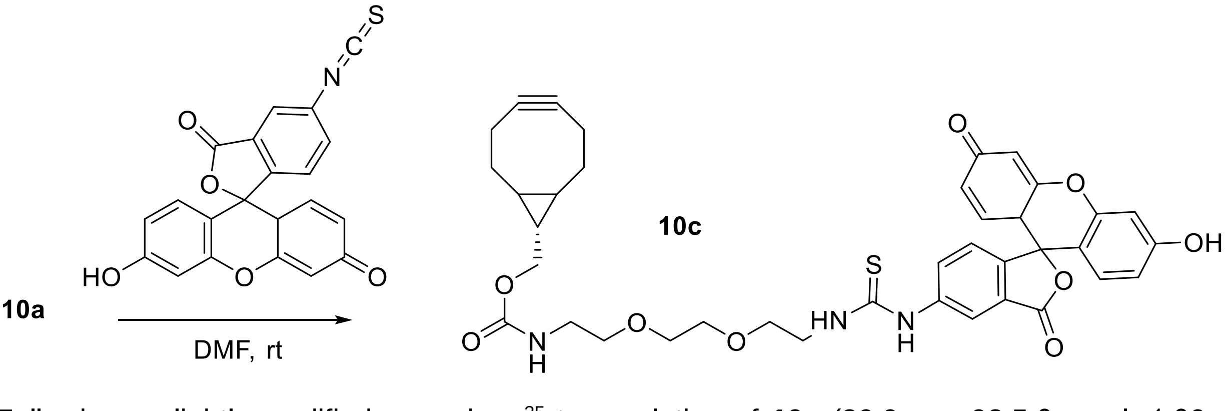

Following a slightly modified procedure,[25] to a solution of **10a** (30.0 mg, 92.5.0 µmol, 1.00 equiv) in DMF (2.10 mL) was added Fluorescein isocyanate (39.6 mg, 102 µmol, 1.10 equiv). After 16 hours, the reaction was dilute with DCM and evaporated under reduced pressure. The

[25] S. M. DeGuire, D. C. Earl, Y. Du, B. A. Crews, A. T. Jacobs, A. Ustione, C. Daniel, K. M. Chong, L. J. Marnett, D. W. Piston, B. O. Bachmann, G. A. Sulikowski, *Angewandte Chemie International Edition* **2015**, *54*, 961–964.

residue was purified by reverse phase preparative HPLC (water 0.1 % TFA to 95:5 ACN:water 0.1 % TFA) to afford **10c** (46.0 mg, 64.4 μmol, 70%) as an orange solid after lyophilization.

^{1}H NMR (CD_3OD, 400 MHz) δ 8.19 (d, *J* = 1.8 Hz, 1H, Ar*H*), 7.78 (dd, *J* = 7.8, 1.2 Hz, 1H, Ar*H*), 7.15 (d, *J* = 8.1 Hz, 1H, Ar*H*), 6.70 (s, 1H, Ar*H*), 6.68 – 6.67 (m, 2H, Ar*H*), 6.67 (s, 1H, Ar*H*), 6.56 (d, *J* = 2.4 Hz, 1H, Ar*H*), 6.53 (d, *J* = 2.4 Hz, 1H, Ar*H*), 4.09 (d, *J* = 8.1 Hz, 2H, CH_2-BCN), 3.86 – 3.77 (m, 2H, CH_2-PEG), 3.71 (t, *J* = 5.1 Hz, 2H, CH_2-PEG), 3.68 – 3.63 (m, 4H, CH_2-PEG), 3.53 (t, *J* = 5.6 Hz, 2H, CH_2-PEG), 3.27 (t, *J* = 5.6 Hz, 2H, CH_2-PEG), 2.24 – 2.18 (m, 2H, CH_2-BCN), 2.10 – 2.06 (m, 1H, C*H*-BCN), 1.65 – 1.46 (m, 2H, CH_2-BCN), 1.40 – 1.23 (m, 4H, CH_2-BCN, C*H*-BCN), 0.97 – 0.81 (m, 2H, CH_2-BCN). **HRMS** (ESI): m/z calculated $[M+H]^+$ = 714.0, found $[M+H]^+$ = 714.2.

Spectroscopic data was consistent with the values reported in literature.[26]

BCN-sugar (9d)

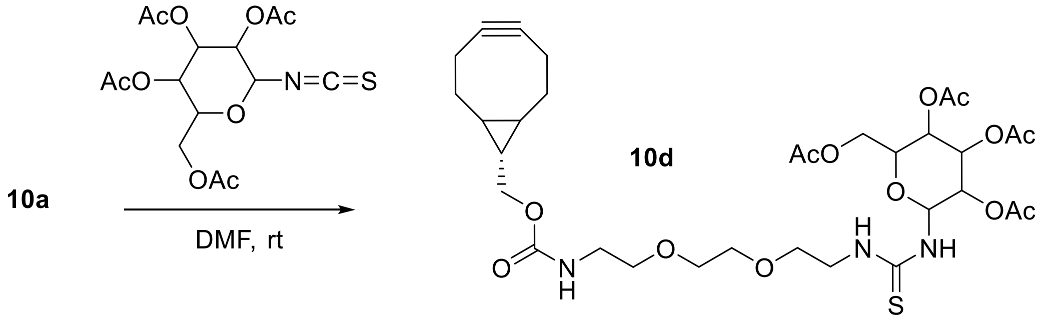

Following a slightly modified procedure,[25] to a solution of **10a** (15.0 mg, 46.0 μmol) in DMF (1.05 mL) was added Sugar isocyanate (18.0 mg, 46.0 μmol). After 16 hours, the reaction was dilute with DCM and evaporated under reduced pressure. The residue was purified by reverse phase preparative HPLC (water 0.1 % TFA to 95:5 ACN:water 0.1 % TFA) to afford **10d** (19.9 mg, 27.9 μmol, 61%) as a white sticky solid after lyophilization.

^{1}H NMR (400 MHz, MeOD) δ 5.83 (d, *J* = 8.4 Hz, 1H, CSNHC*H*O), 5.32 (q, *J* = 9.2 Hz, 1H, C*H*OAc), 5.04 (m, 2H, C*H*OAc), 4.28 (dd, *J* = 12.0, 4.3 Hz, 1H, OCHCH_2OAC), 4.16 (d, *J* = 8.0 Hz, 2H, NHCOOCH_2), 4.10 (dd, *J* = 12.4, 2.4 Hz, 1H, OCHCH_2OAC), 3.96 – 3.86 (m, 1H, OC*H*CH_2OAC), 3.59 (m, 12H, CH_2-PEG), 2.32 – 2.13 (m, 5H, CH_2-BCN, C*H*-BCN), 2.04 (s, 3H, CH_3-Ac), 2.01 (d, *J* = 1.8 Hz, 6H, CH_3-Ac), 1.98 (s, 3H, CH_3-Ac), 1.62 (m, 2H, CH_2-BCN), 1.47 – 1.26 (m, 2H, CH_2-BCN), 0.93 (m, 2H, C*H*-BCN). **^{13}C NMR** (101 MHz, MeOD) δ 172.3, 171.6 (*4 C under this peak*), 171.5, 171.3, 99.5, 74.8, 74.4, 72.0, 71.4, 71.3, 71.1, 70.2, 69.7, 63.8, 63.1, 57.7, 57.5, 57.3, 41.7, 30.2, 21.9, 21.4, 20.7, 20.6, 19.0, 17.3. **HRMS** (nanochip-ESI/LTQ-Orbitrap) m/z: $[M + Na]^+$ Calcd for $C_{32}H_{47}N_3NaO_{13}S^+$ 736.2722; Found 736.2694.

[26] P. M. S. D. Cal, R. F. M. Frade, C. Cordeiro, P. M. P. Gois, *Chem. Eur. J.* **2015**, *21*, 8182–8187.

d. SPAAC on isolated O-VBX

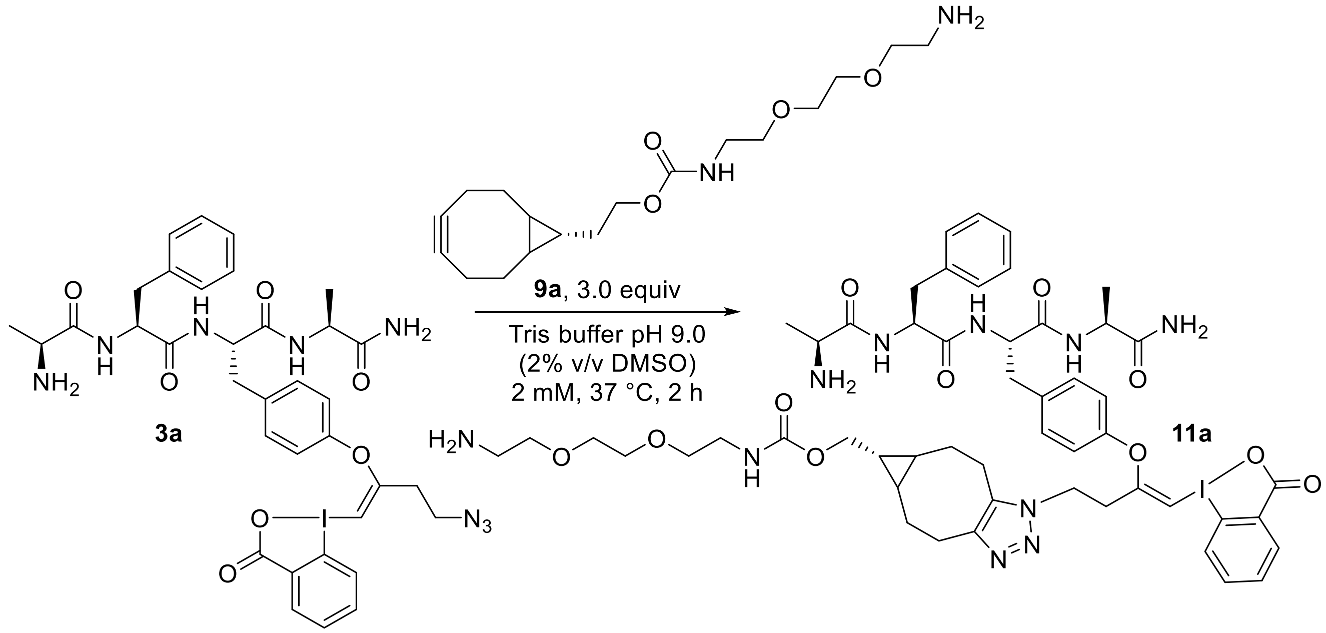

To a 2.0 mM solution of O-VBX AFYA (**3a**) (16.2 mg, 20.0 μmol, 20.0 equiv) in 100 mM Tris buffer pH 9.0 (10.0 mL) was added a 30.0 mM solution of Click reagent, 9-bicyclo[6.1.0]non-4-ynylmethyl N-[2-[2-(2-aminoethoxy)ethoxy]ethyl]carbamate (**10a**) (9.73 mg, 30.0 μmol, 1.50 equiv in 1.00 mL DMSO). The resulting solution was shaken at room temperature for 2 hours. No effort was made to exclude oxygen. The reaction was analyzed by HPLC-MS. The crude was lyophilized and purified by preparative RP-HPLC (water to ACN 95:5 in 20 min). Fractions containing the desired product were lyophilized to afford bioconjugate **11a** (15.7 mg, 10.3 μmol, 51% yield), as a white solid (retention time 6.8 min).

^{1}H NMR (400 MHz, MeOD) δ 8.26 (dd, *J* = 7.6, 1.7 Hz, 1H, Ar*H*), 7.88 – 7.79 (m, 1H, Ar*H*), 7.74 (dd, *J* = 7.4, 1.1 Hz, 1H, Ar*H*), 7.61 (d, *J* = 8.2 Hz, 1H, Ar*H*), 7.29 – 7.13 (m, 7H, Ar*H*), 6.98 – 6.91 (m, 2H, Ar*H*), 6.37 (s, 1H, C*H*-I), 4.60 – 4.50 (m, 3H, $CH_2CH_2N_3$, C*H*-Tyr), 4.45 (dd, *J* = 8.4, 5.7 Hz, 1H, C*H*-Phe), 4.31 (q, *J* = 7.2 Hz, 1H, C*H*-Ala), 4.12 (dd, *J* = 18.5, 8.6 Hz, 2H, CHC*H*$_2$OCONH-PEG), 3.82 (q, *J* = 7.0 Hz, 1H, C*H*-Ala), 3.72 – 3.68 (m, 2H, C*H*$_2$-PEG), 3.68 – 3.63 (m, 4H, C*H*$_2$-PEG), 3.54 (t, *J* = 5.7 Hz, 2H, C*H*$_2$-PEG), 3.31 – 3.22 (m, 4H, C*H*$_2$-PEG), 3.20 – 3.05 (m, 5H, C*H*$_2$-Phe, C*H*$_2$CH$_2$N$_3$, C*H*$_2$-BCN), 2.99 – 2.82 (m, 4H, C*H*$_2$-BCN, C*H*$_2$-Tyr), 2.68 (dtd, *J* = 10.4, 6.6, 3.5 Hz, 1H, C*H*$_2$-Tyr), 2.29 – 2.13 (m, 2H, C*H*$_2$-BCN), 1.58 (d, *J* = 14.9 Hz, 2H, C*H*$_2$-BCN), 1.36 (dd, *J* = 7.1, 3.8 Hz, 6H, C*H*$_3$), 1.23 (d, *J* = 8.4 Hz, 1H, C*H*-BCN), 1.04 (d, *J* = 9.5 Hz, 2H, C*H*-BCN). **^{13}C NMR** (101 MHz, MeOD) δ 177.1, 172.9, 172.2, 171.0, 170.5, 167.6, 159.0, 153.7, 146.2, 138.2, 136.3, 135.8, 133.6, 132.5, 132.0, 130.2, 129.5, 129.5, 129.5, 127.9, 120.7, 119.0, 114.5, 101.4, 81.2, 71.3, 71.3, 71.1, 67.9, 63.5, 56.5, 56.0, 50.0, 46.2, 41.5, 40.7, 38.5, 37.8, 33.5, 26.6, 23.7, 23.6, 23.0, 21.1, 20.3, 19.0, 18.4, 17.7. **HRMS** (ESI/QTOF) m/z: $[M + H_2]^{+2}$ Calcd for $C_{52}H_{69}IN_{10}O_{11}^{+2}$ 568.2091; Found 568.2102.

HPLC-UV chromatogram (214 nm) of crude of the reaction:

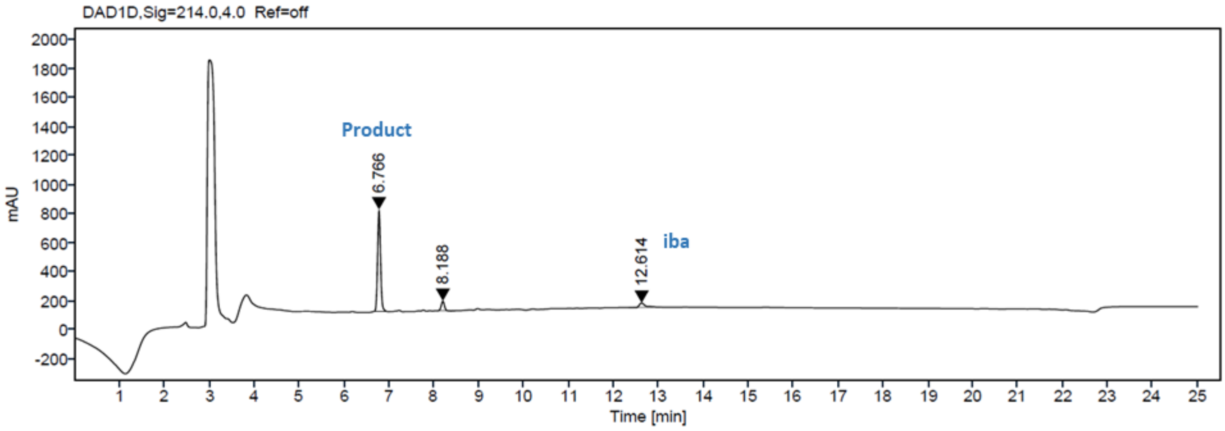

HPLC-UV chromatograms (214 nm) of isolated **11a**:

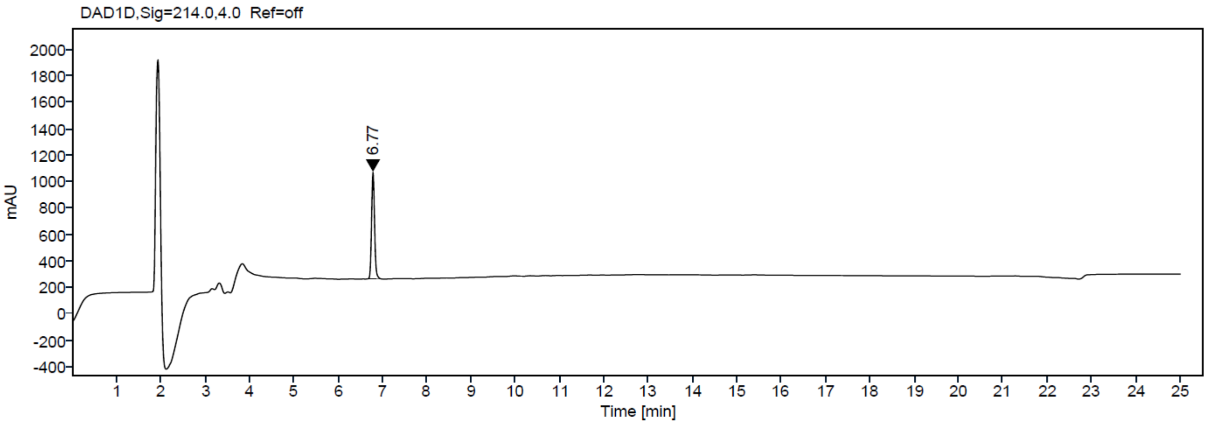

e. One pot-two steps SPAAC:

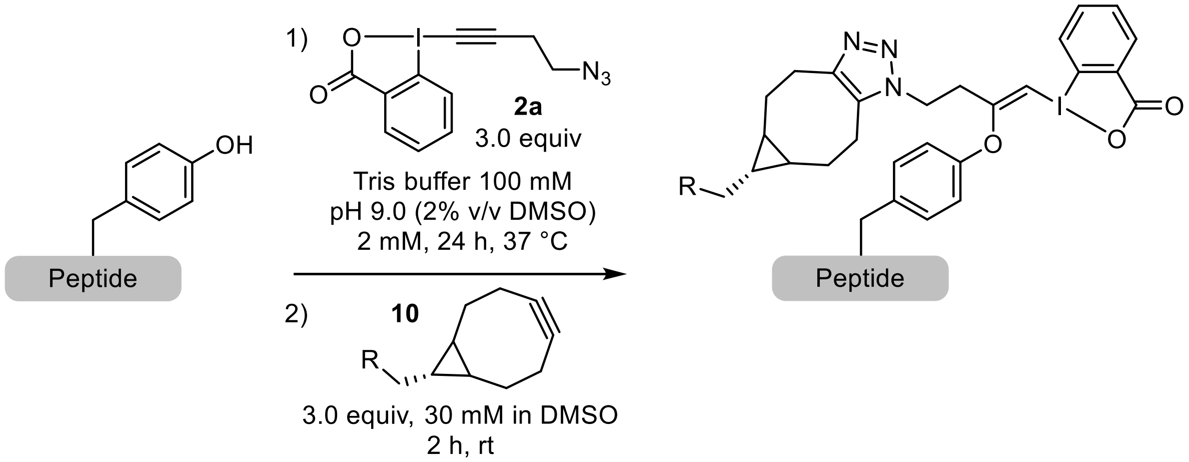

General procedure:

To a solution of β-Casomorphin **1r** (864 µg, 1.00 µmol, 1.00 equiv) or Vasopressin **1o** (1.00 mg, 0.762 µmol, 1.00 equiv) in 100 mM Tris buffer pH 9.0 (490 µL), in a 0.5 mL Eppendorf Safe-Lock microcentrifuge tube, was added a 300 mM solution of N_3-EBX (1.50 µmol, 1.50 equiv) in DMSO. The 2.00 mM solution was shaken at 37 °C for 24 h. No effort was made to exclude oxygen. Then, a 30.0 mM solution of cyclooctyne reagent (**10a-d**) in DMSO (100 µL, 3.00 µmol, 3.00 equiv.) was added. The resulting mixture was shaken at room temperature for 2 hours. No effort was made to exclude oxygen. The reaction was analyzed by HPLC-UV.

The yields are given over two steps by HPLC-UV. The yields were approximated as the ratio of Aprod/Atotal where Aprod = area in mAU of the product peak and Atotal = area in mAU of all peptides products (product, starting material, and side products if present).

Bioconjugate (12a)

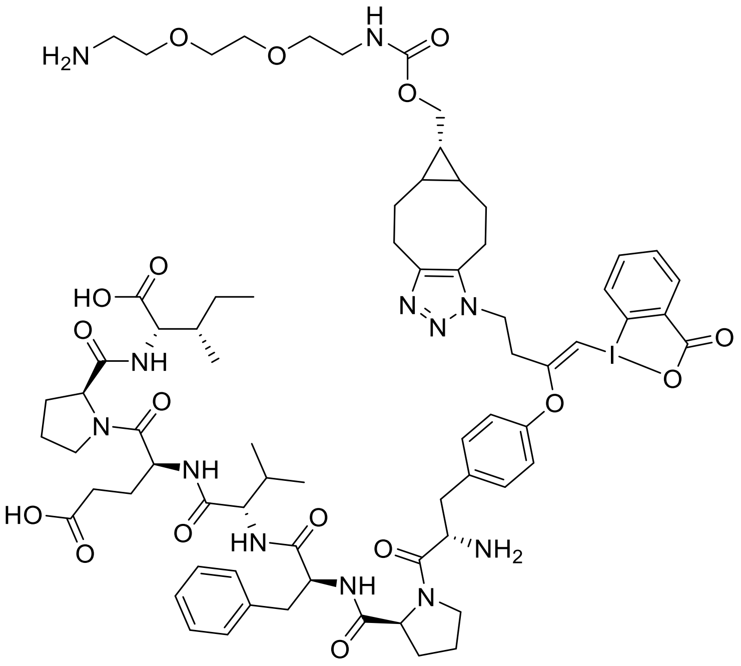

Following the general procedure on β-Casomorphin (**1r**), the *N*-[(1*R*,8*S*,9*S*)-Bicyclo[6.1.0]non-4-yn-9-ylmethyloxycarbonyl]-1,8-diamino-3,6-dioxaoctane (1.02 mg, 3.00 µmol) (**10a**) afforded the O-VBX bioconjugate (**12a**) in 86% yield based on HPLC-UV (retention time 8.1 min).

HRMS (nanochip-ESI/LTQ-Orbitrap) m/z: [M + H_2]$^{+2}$ Calcd for $C_{72}H_{99}IN_{12}O_{17}^{+2}$ 765.3142; Found 765.3136.

MS/MS characterization:

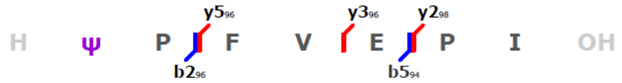

ψ = Tyr(C28H36IN5O6)

Sequence	Type	MF	m/z	Intensity	Similarity
**YP**	b2	C42H53IN7O9(+1)	926.2944	5.06	0.96
FVEPI	y5	C30H46N5O8(+1)	604.3341	3.50	0.96
PI	y2	C11H21N2O3(+1)	229.1547	2.33	0.98
**YPFVE**	b5	C61H78IN10O14(+1)	1301.4738	1.83	0.93
**YPFVE**	b5	C61H78IN10O14(+1)	651.2405	1.32	0.95
EPI	y3	C16H28N3O6(+1)	358.1973	0.60	0.96

HPLC-UV chromatograms (214 nm) of crude of the reaction:

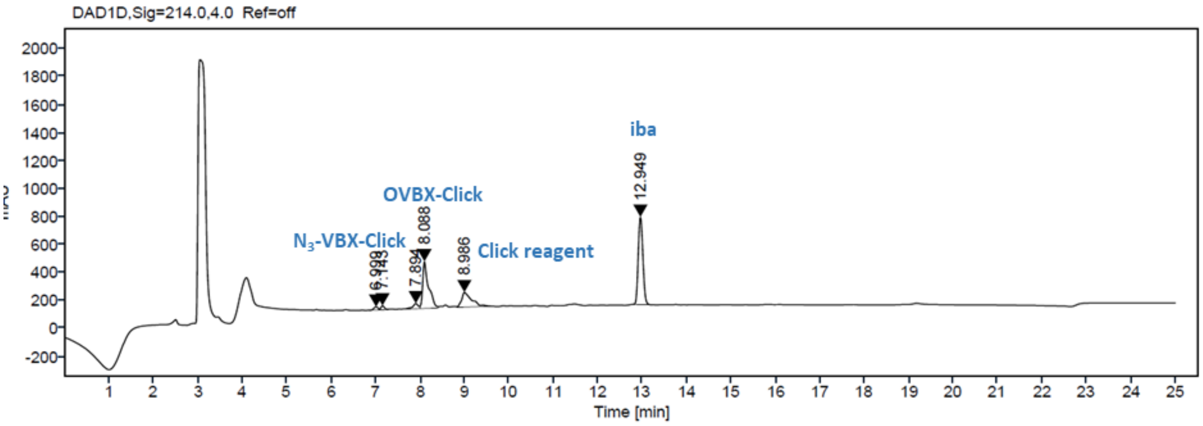

HPLC-MS chromatograms of crude of the reaction:

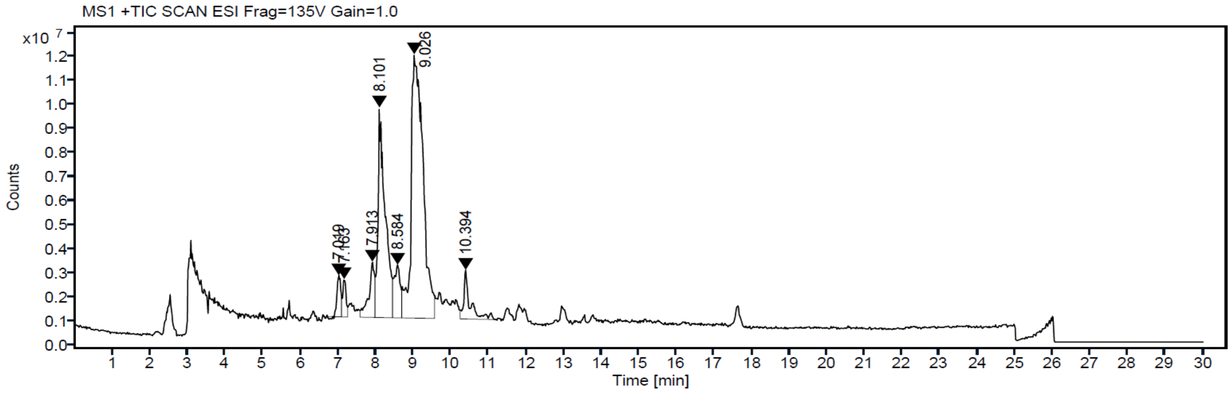

Bioconjugate (12b)

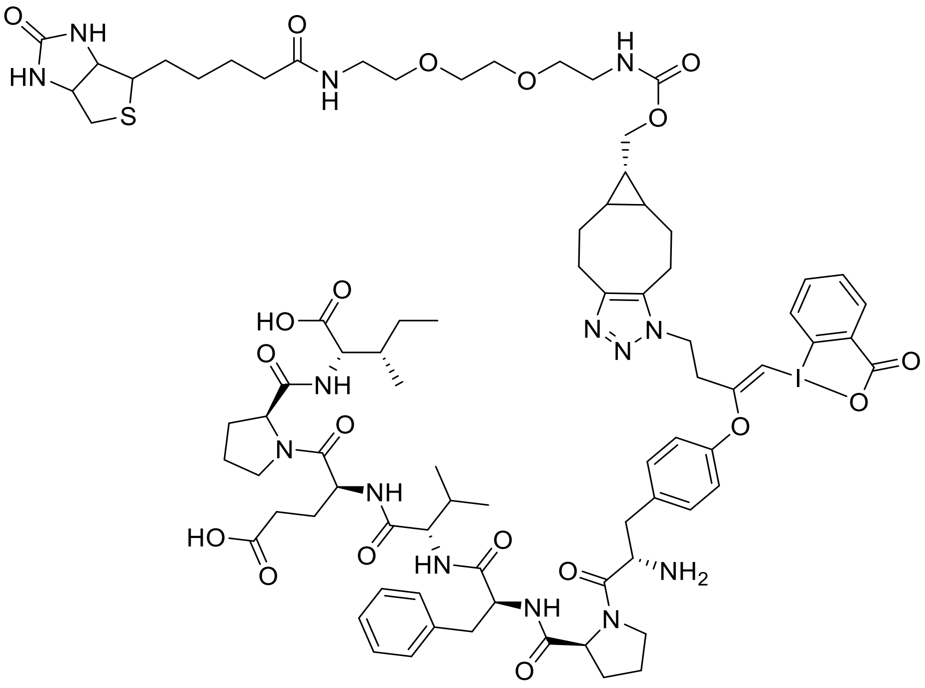

Following the general procedure on β-Casomorphin (**1r**), the Biotin derivate (1.65 mg, 3.00 µmol) (**10b**) afforded the O-VBX bioconjugate (**12b**) in 70% yield based on HPLC-UV (retention time 9.2 min).

HRMS (nanochip-ESI/LTQ-Orbitrap) m/z: $[M + H]^+$ Calcd for $C_{82}H_{112}IN_{14}O_{19}S^+$ 1755.6988; Found 1755.6980.

MS/MS characterization:

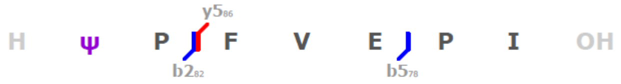

ψ = Tyr(C38H50IN7O8S)

Sequence	Type	MF	m/z	Intensity	Similarity
**YPFVE**	b5	C71H92IN12O16S(+1)	764.2793	1.71	0.80
**YPFVE**	b5	C71H92IN12O16S(+1)	1527.5514	1.34	0.76
**YP**	b2	C52H67IN9O11S(+1)	1152.3720	0.81	0.82
FVEPI	y5	C30H46N5O8(+1)	604.3341	0.51	0.86

HPLC-UV chromatograms (214 nm) of crude of the reaction:

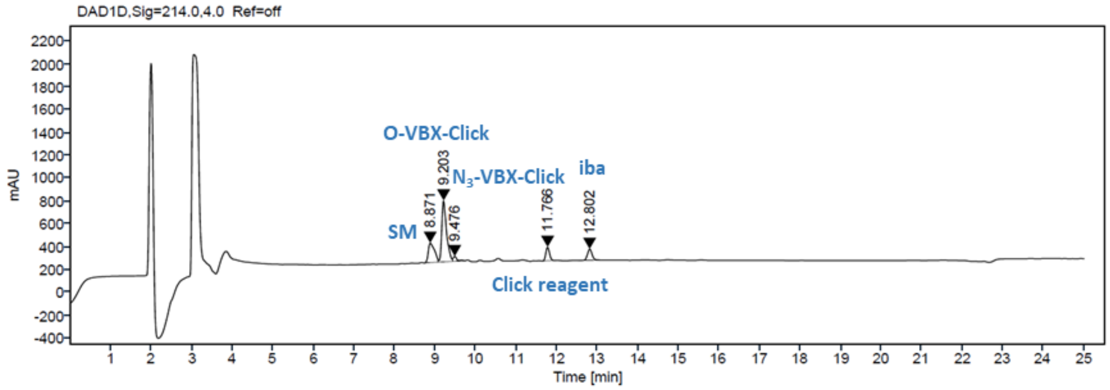

HPLC-MS chromatograms of crude of the reaction:

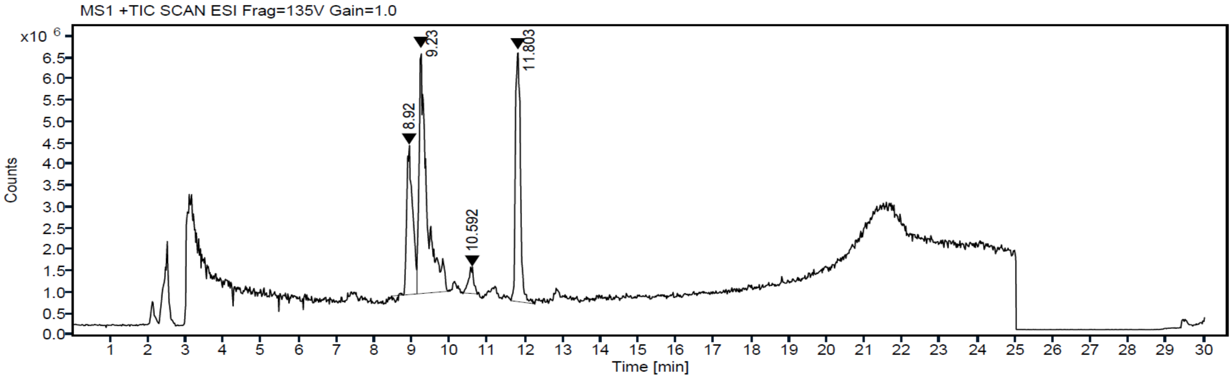

Bioconjugate (12c)

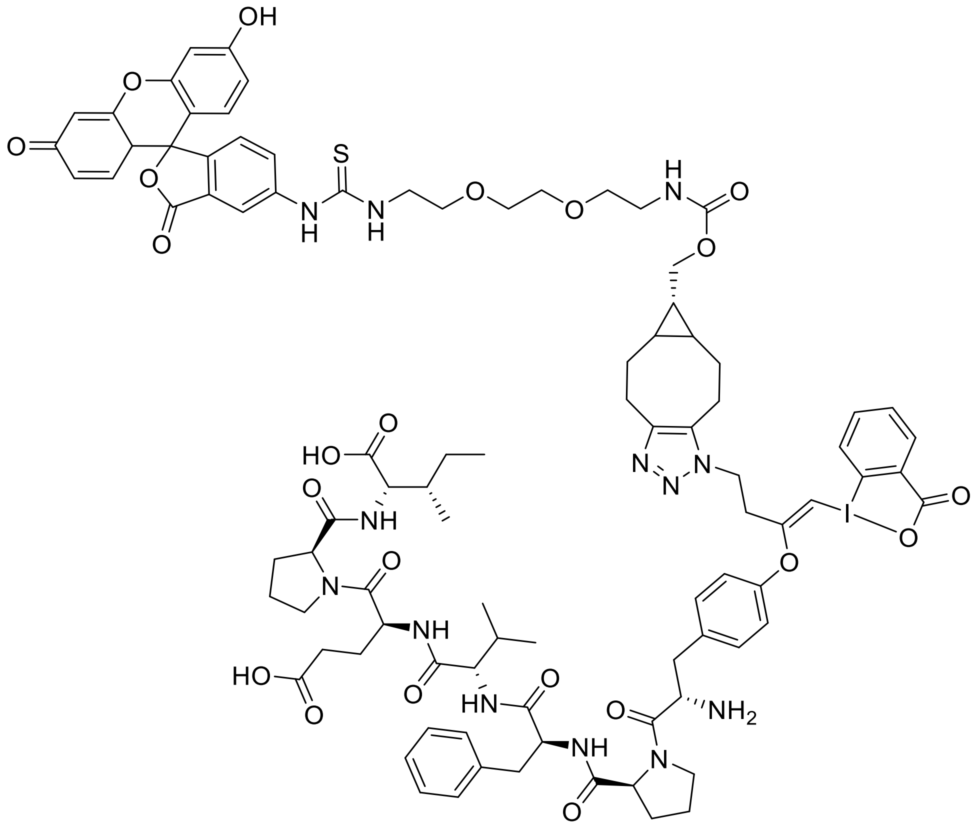

Following the general procedure on β-Casomorphin (**1r**), the Fluorophore derivate (2.14 mg, 3.00 μmol) (**10c**) afforded the O-VBX bioconjugate (**12c**) in 51% yield based on HPLC-UV (retention time 10.9 min).

HRMS (nanochip-ESI/LTQ-Orbitrap) m/z: $[M + H_2]^{+2}$ Calcd for $C_{93}H_{110}IN_{13}O_{22}S^{+2}$ 959.8321; Found 959.8312.

MS/MS characterization:

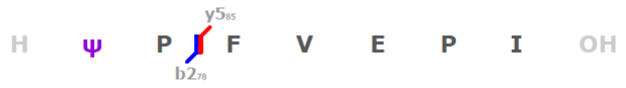

ψ = Tyr(C49H47IN6O11S)

Sequence	Type	MF	m/z	Intensity	Similarity
**YP**	b2	C63H64IN8O14S(+1)	1315.3302	1.36	0.78
FVEPI	y5	C30H46N5O8(+1)	604.3341	0.98	0.85

HPLC-UV chromatograms (214 nm) of crude of the reaction:

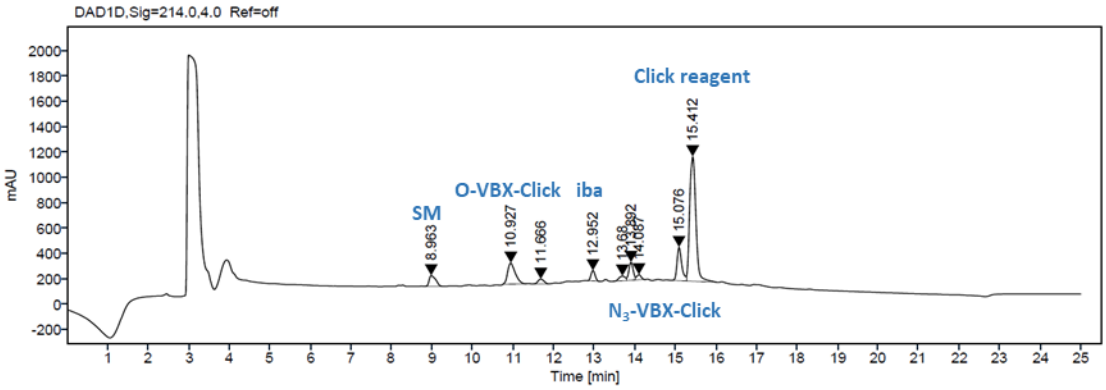

HPLC-MS chromatograms of crude of the reaction:

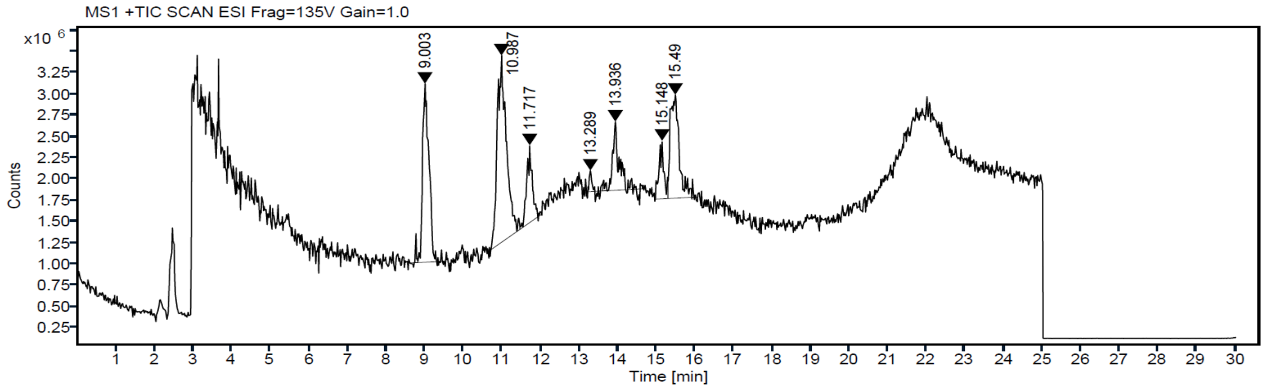

Bioconjugate (12d)

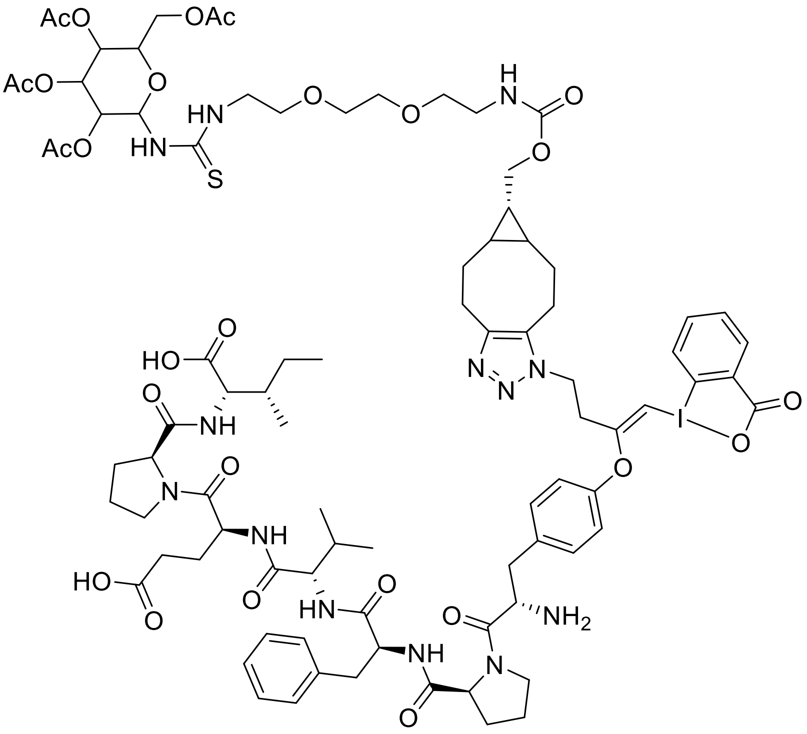

Following the general procedure on β-Casomorphin (**1r**), the Sugar derivate (2.14 mg, 3.00 µmol) (**10d**) afforded the O-VBX biocnjugate (**12d**) in 58% yield based on HPLC-UV (retention time 10.99 min).

HRMS (nanochip-ESI/LTQ-Orbitrap) m/z: $[M + H_2]^{+2}$ Calcd for $C_{87}H_{118}IN_{13}O_{26}S^{+2}$ 959.8533; Found 959.8519.

MS/MS characterization:

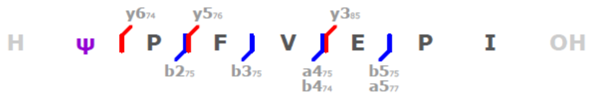

ψ = Tyr(C43H55IN6O15S)

Sequence	Type	MF	m/z	Intensity	Similarity
**YPFVE**	b5	C76H97IN11O23S(+1)	845.7796	19.75	0.72
PFVEPI	y6	C35H53N6O9(+1)	701.3869	2.28	0.74
FVEPI	y5	C30H46N5O8(+1)	604.3341	2.06	0.76
**YPFV**	b4	C71H90IN10O20S(+1)	781.2583	1.97	0.74
**YPFVE**	b5	C76H95IN11O22S(+1)	836.7743	1.60	0.78
**YP**	b2	C57H72IN8O18S(+1)	658.1899	1.29	0.75
**YPF**	b3	C66H81IN9O19S(+1)	731.7241	1.27	0.75
EPI	y3	C16H28N3O6(+1)	358.1973	1.25	0.83
**YPFVE**	a5	C75H97IN11O22S(+1)	831.7821	1.17	0.77
EPI	y3	C16H26N3O5(+1)	340.1867	0.76	0.82
**YPFV**	a4	C70H90IN10O19S(+1)	767.2608	0.64	0.86

HPLC-UV chromatograms (214 nm) of crude of the reaction:

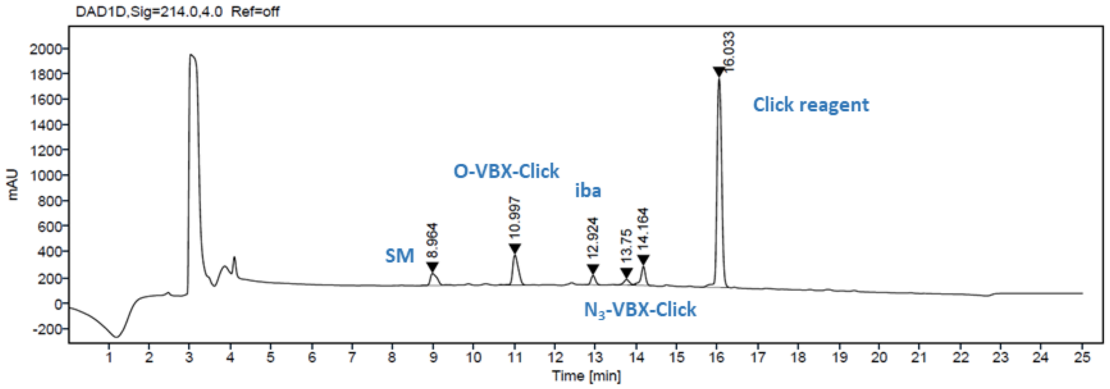

HPLC-MS chromatograms of crude of the reaction:

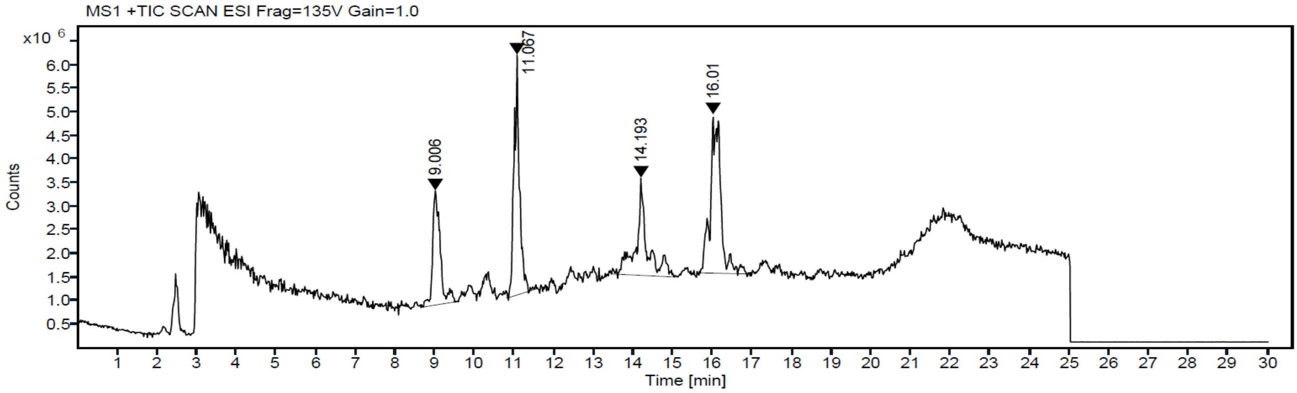

Bioconjugate (13b)

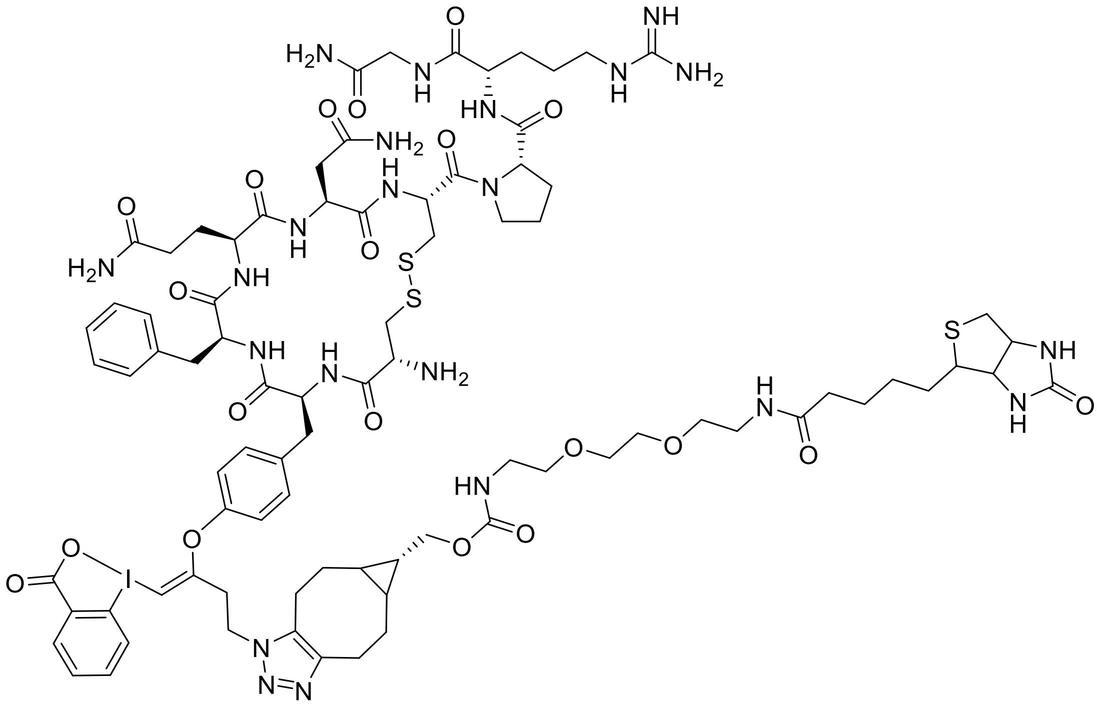

Following the general procedure on Vasopressin (**1o**), Biotine derivate (1.26 mg, 3.00 µmol) (**10b**) afforded the O-VBX bioconjugate (**13b**) in 93% yield based on HPLC-UV (retention time 7.9 min).

HRMS (nanochip-ESI/LTQ-Orbitrap) m/z: $[M + H_2]^{+2}$ Calcd for $C_{84}H_{117}IN_{22}O_{20}S_3^{+2}$ 988.3505; Found 988.3508.

MS/MS characterization: *The lack of processing tools to assign fragment ions for such cyclic modified peptide did not allow us to confirm its structure based on the fragmentation spectra obtained.*

HPLC-UV chromatograms (214 nm) of crude of the reaction:

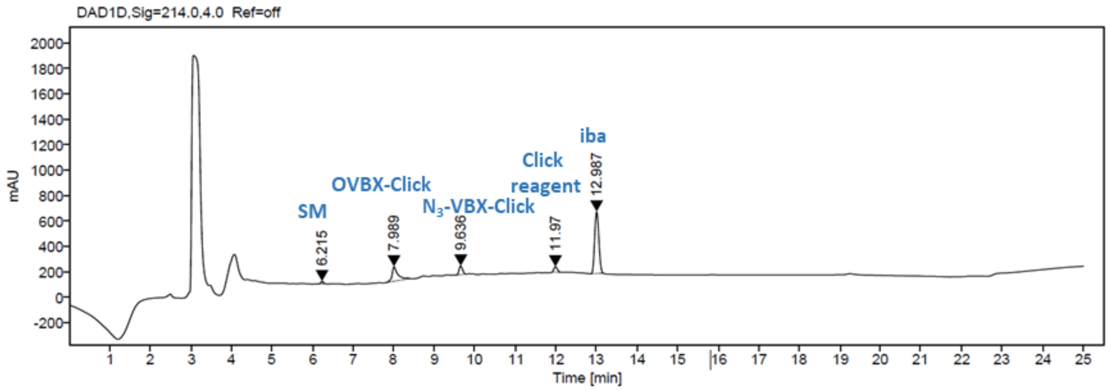

HPLC-MS chromatograms of crude of the reaction:

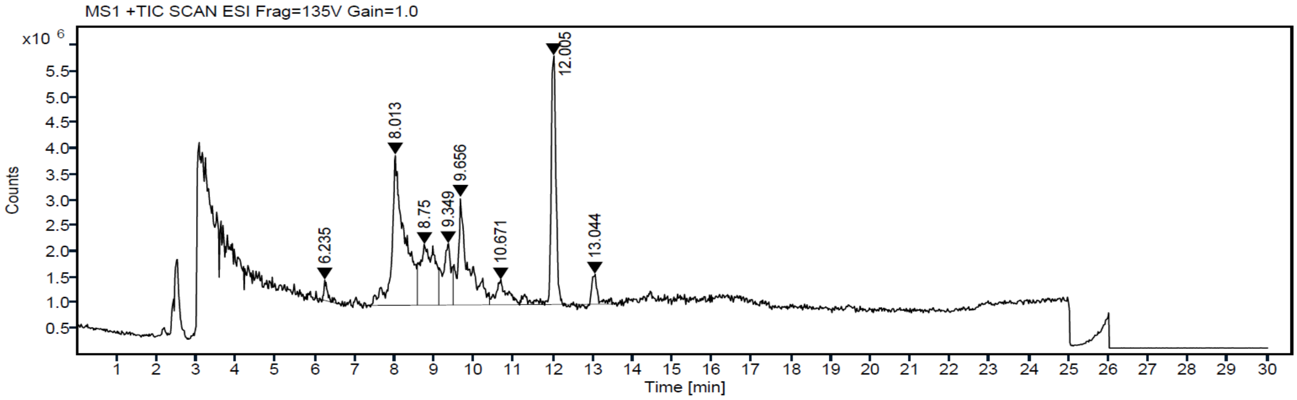

Bioconjugate (13c)

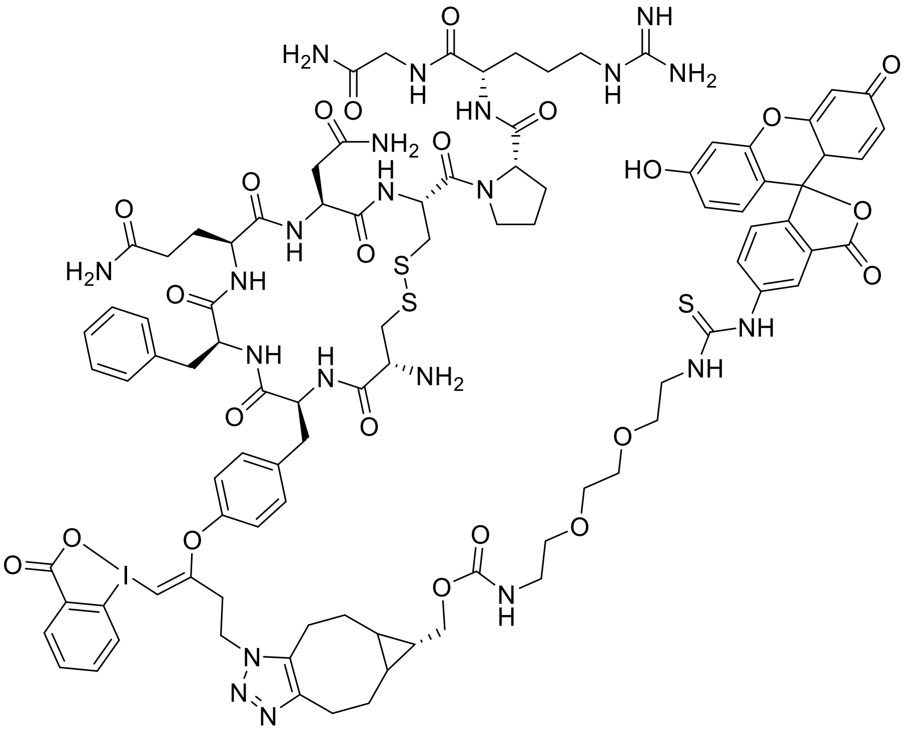

Following the general procedure on Vasopressin (**1o**), Fluorophore derivate (1.63 mg, 3.00 µmol) (**10c**) afforded the O-VBX bioconjugate (**13c**) in 67% yield based on HPLC-UV (retention time 9.2 min).

HRMS (nanochip-ESI/LTQ-Orbitrap) m/z: $[M + H_2]^{+2}$ Calcd for $C_{95}H_{114}IN_{21}O_{23}S_3^{+2}$ 1069.8296; Found 1069.8291.

MS/MS characterization: *The lack of processing tools to assign fragment ions for such cyclic modified peptide did not allow us to confirm its structure based on the fragmentation spectra obtained.*

HPLC-UV chromatograms (214 nm) of crude of the reaction:

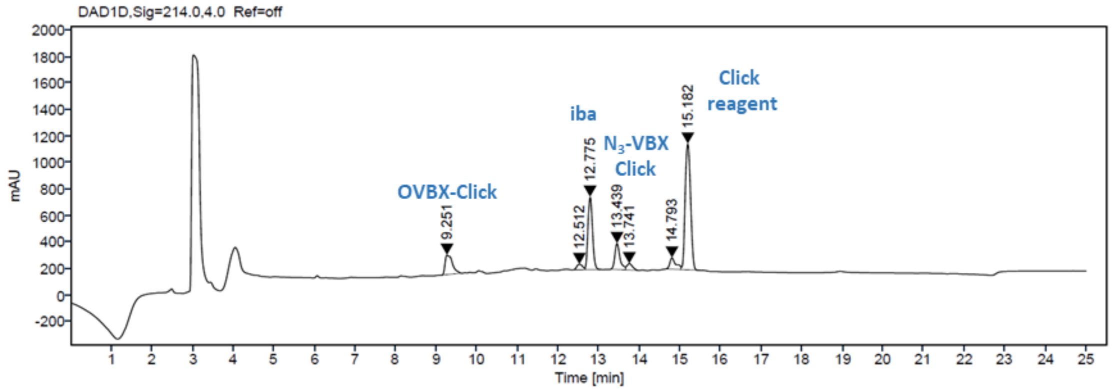

HPLC-MS chromatograms of crude of the reaction:

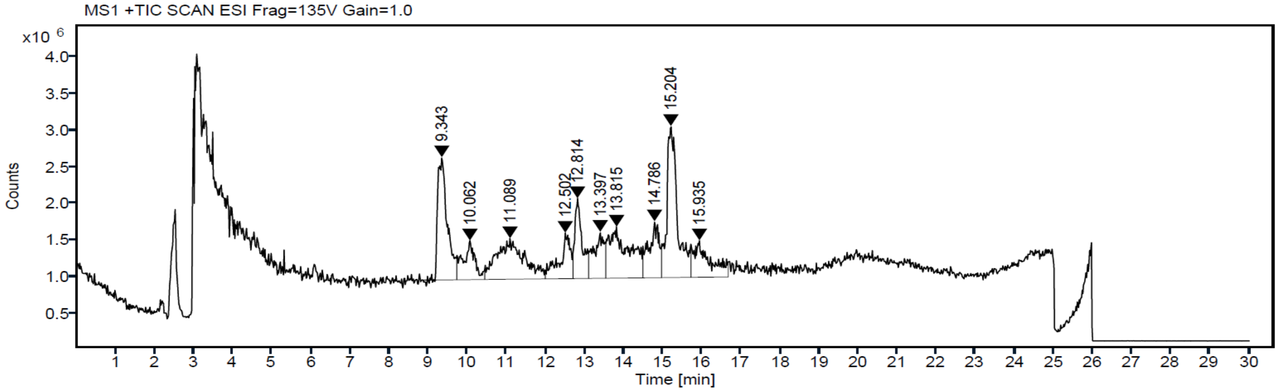

Bioconjugate (13d)

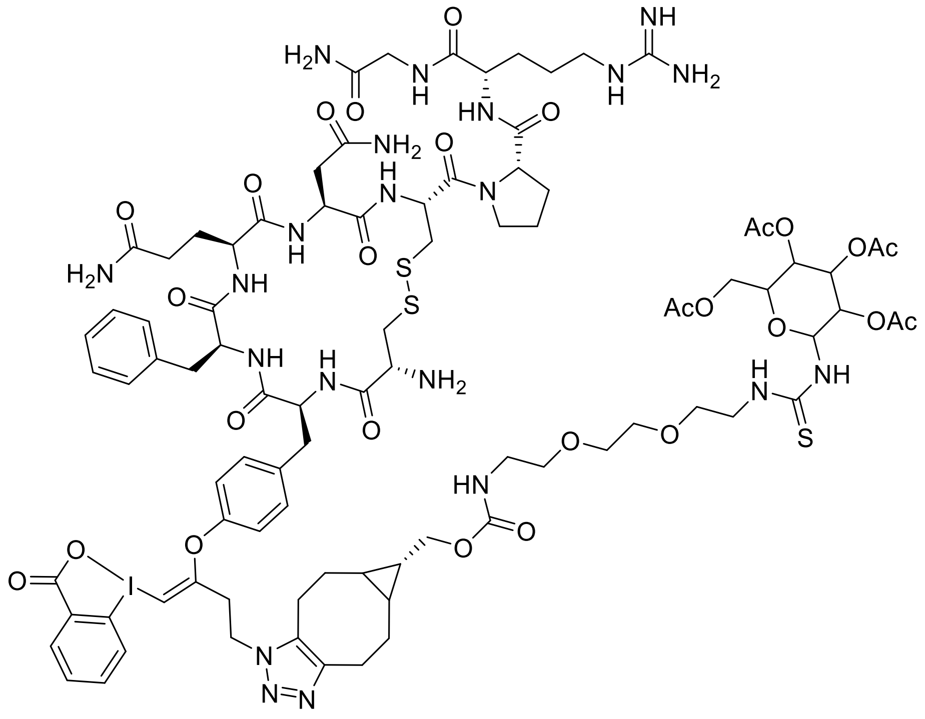

Following the general procedure on Vasopressin (**1o**) and Sugar derivate (1.63 mg, 3.00 µmol) (**10d**) afforded the O-VBX bioconjugate (**13d**) in 54% yield based on HPLC-UV (retention time 9.3 min).

HRMS (nanochip-ESI/LTQ-Orbitrap) m/z: $[M + H_2]^{+2}$ Calcd for $C_{89}H_{122}IN_{21}O_{27}S_3^{+2}$ 1069.8507; Found 1069.8291.

MS/MS characterization: *The lack of processing tools to assign fragment ions for such cyclic modified peptide did not allow us to confirm its structure based on the fragmentation spectra obtained.*

HPLC-UV chromatograms (214 nm) of crude of the reaction:

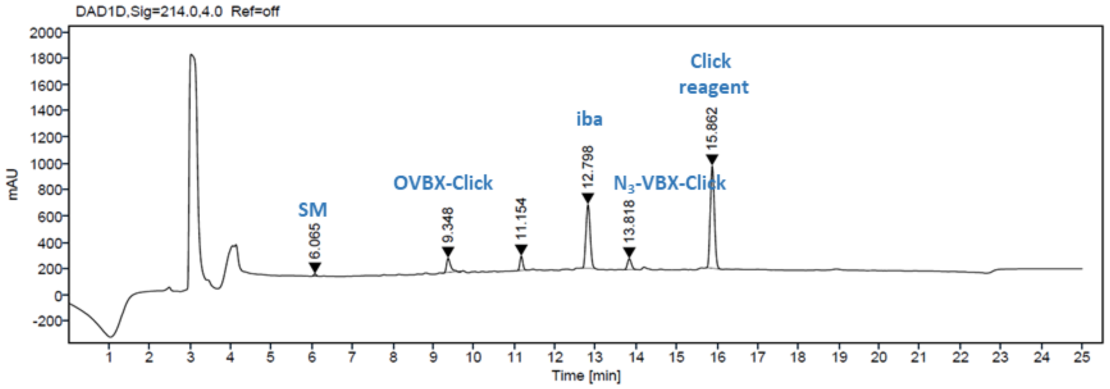

HPLC-MS chromatograms of crude of the reaction:

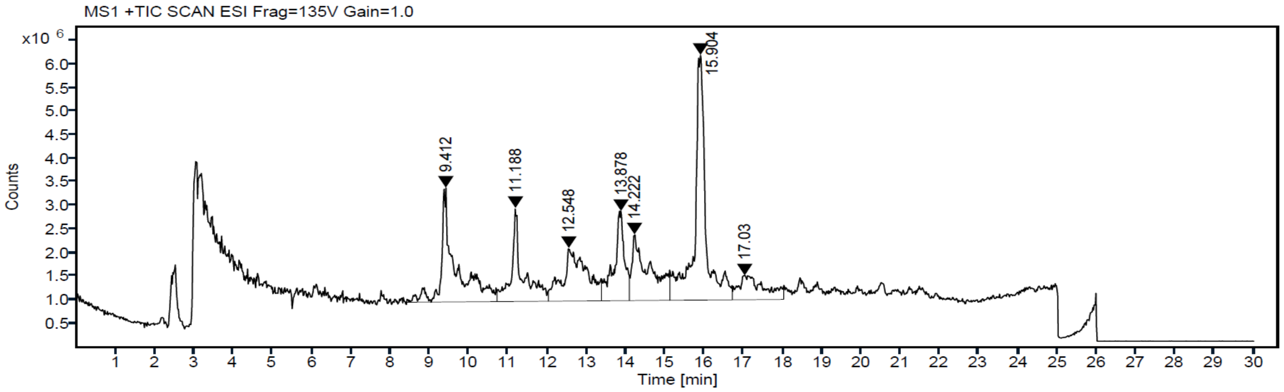

10. Suzuki reaction

a. Synthesis of ligands and preparation of palladium complexes

2-(Dimethylamino)pyrimidine-4,6-diol (S24)

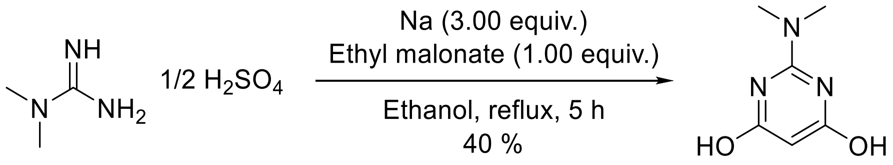

S24 **S25**

Following a reported procedure,[i] to a solution of sodium (Na, 93.0 mg, 4.04 mmol, 1.10 equiv.) in ethanol (1.9 mL, 2.12 M), dimethyl guanidine sulfate (**S24**) (1.00 g, 3.67 mmol, 1.00 equiv.) was added. The resulting solution was added to another solution of sodium (Na, 0.160 g, 6.98 mmol, 1.90 equiv.) and diethyl malonate (0.560 mL, 3.67 mmol, 1.00 equiv.) in ethanol (1.96 mL, 1.87 M). The combined solution was refluxed for 5 hours. The reaction was then evaporated to dryness, dissolved in water (5 mL) and taken to pH 6 with acetic acid. Collection of the white solid formed by filtration under vacuum afforded product **S25** (0.225 g, 1.45 mmol, 40%).

^{1}H NMR (400 MHz, $CDCl_3$) δ 10.50 (s, 2H, O*H*), 4.65 (s, 1H, Ar*H*), 3.00 (s, 6H,-N(C*H*$_3$)$_2$). **^{13}C NMR** (101 MHz, $CDCl_3$) δ 168.0, 155.0, 78.3, 37.1. **HRMS** (ESI/QTOF) m/z: $[M + H]^+$ Calcd for $C_6H_{10}N_3O_2^+$ 156.0768; Found 156.0766.

Spectroscopic data was consistent with that previously reported in the literature.[27]

Pyrimidine-4,6-diol-based Palladium complex:

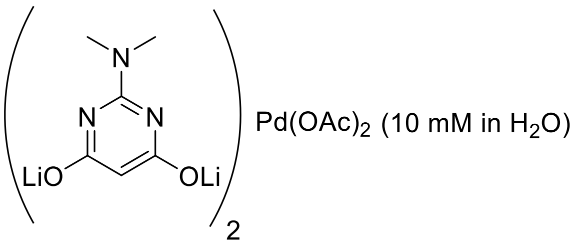

Palladium catalyst **Pd-1** was synthesized following reported procedure.[28] 2-amino-4,6-dihydroxypyrimidine based ligand (**S25**) (20.0 µmol) was dissolved in an aqueous solution of LiOH (0.4 mL, 0.1m) in an ultrasonic bath for 2 minutes. The palladium source (10.0 µmol) was added and the mixture was magnetically stirred at 65 °C for 30 minutes, deionized water (0.6 mL) was then added to afford a 10.0 mM catalyst solution of **Pd-1**. The 40.0 mM catalyst solution was achieved with a 0.2 M solution of LiOH.

[27] Latham, J.; Henry, J.-M.; Sharif, H.; Menon, B.; Shepherd, S.; Greaney, M.; Micklefield, J. *Nat. Comm.* **2016**, *7*, 11873.
[28] Chalker, J.; Wood, C.; Davis, B. *J. Am. Chem. Soc.* **2009**, *131*, 16346.

b. Synthesis of boronic acid

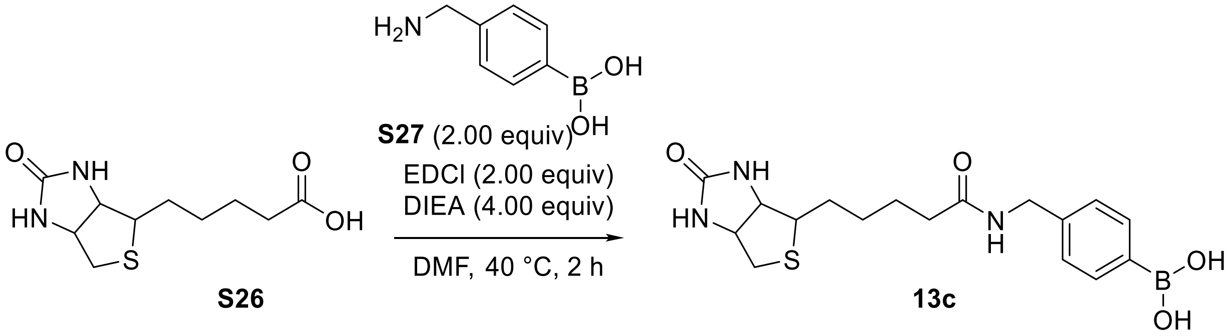

Following a reported procedure,[29] D-Biotin **S26** (122 mg, 0.500 mmol) and *p*-(aminomethyl)phenylboronic hydrochloride **S27** (187 mg, 1.00 mmol, 2.00 equiv) were dissolved in 5 mL of DMF. EDCI (191 mg, 1.00 mmol, 2.00 equiv) and DIEA (259 mg, 2.00 mmol, 4.00 equiv) were added to the mixture, which was then stirred at 40 °C for 2 h. After the solvent was removed under vacuum, the crude product was purified by column chromatography (10% MeOH in CH_2Cl_2), affording product **13c** as white solid (110 mg, 0.292 mmol, 58%):

^{1}H NMR (400 MHz, DMSO) δ 8.28 (t, *J* = 6.0 Hz, 1H, N*H*), 7.96 (s, 2H, Ar*H*), 7.72 (d, *J* = 7.8 Hz, 2H, Ar*H*), 7.19 (d, *J* = 7.8 Hz, 2H, O*H*), 6.42 (s, 1H, N*H*), 6.35 (s, 1H, N*H*), 4.30 (dd, *J* = 7.8, 5.1 Hz, 1H, C*H*), 4.25 (d, *J* = 5.5 Hz, 2H, NC*H₂*), 4.12 (dd, J = 7,8, 5.9 Hz, 1H, C*H*), 3.14 – 3.04 (m, 1H, SC*H*), 2.83 (dd, *J* = 12.4, 5.1 Hz, 1H, SC*H₂*), 2.58 (d, *J* = 12.4 Hz, 1H, SC*H₂*), 2.14 (t, *J* = 7.4 Hz, 2H, C*H₂*), 1.68 – 1.42 (m, 4H, C*H₂*), 1.39 – 1.18 (m, 2H, C*H₂*). **HRMS** (ESI/QTOF) m/z: $[M + H]^+$ Calcd for $C_{17}H_{25}BN_3O_4S^+$ 378.1653; Found 378.1655.

Spectroscopic data was consistent with that previously reported in the literature.[29]

[29] Ma, X.; Wang, H.; Chen, W. *J. Org. Chem.* **2014**, *79* (18), 8652–8658.

c. Suzuki on isolated O-VBX:

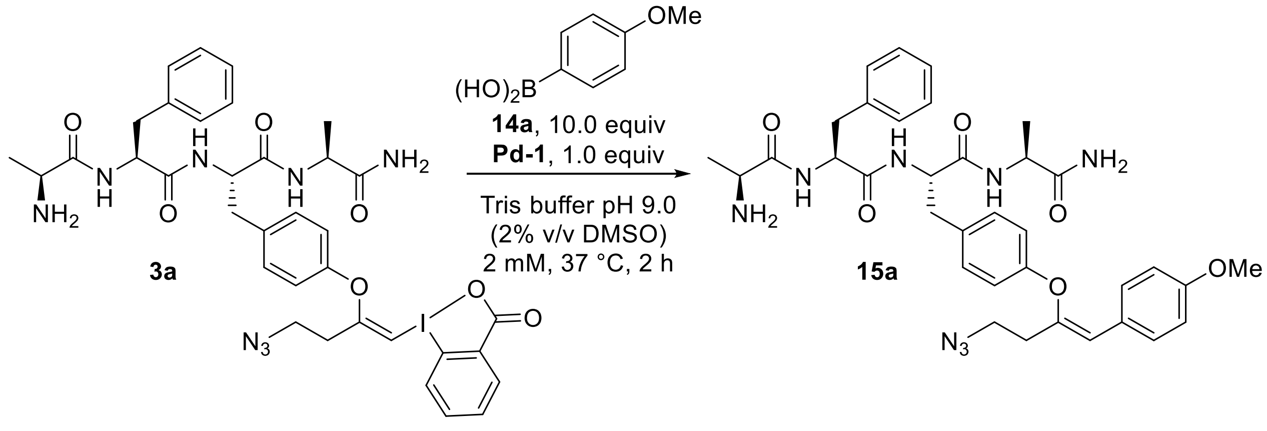

A 25 mL one neck round bottom flask was charged with (4-methoxyphenyl)boronic acid (**14a**) (30.4 mg, 200 µmol, 10.0 equiv), Tris buffer (100 mM, pH 9.0, 5.80 mL), DMSO (200 µL) and stirred at 37 °C, upon complete dissolution. Then a solution of OVBX (**3a**) (16.2 mg, 20.0 µmol, 1.00 equiv) in Tris buffer (100 mM, pH 9.0, 2.00 mL) was added and the mixture was stirred at 37 °C for 10 minutes. Then, a 10.0 mM solution of palladium catalyst (**Pd-1**) in water (2.0 mL, 20 µmol, 1.00 equiv.) was added and the reaction mixture was stirred at 37 °C for 2 hours. The resulting reaction mixture was quenched with a 570 mM solution of 3-mercaptopropionic acid in water (0.20 mL, 114 µmol, 5.70 equiv.) and stirred for an additional 10 minutes at 37 °C. The crude mixture was lyophilized to afford an orange solid. The solid was washed with ethanol (10 mL) and centrifuged. The mother liquid was removed and collected in a round bottom flask. The procedure was repeated once. The orange solid was dissolved in water and analysed by HPLC. The collected mother liquids were evaporated under reduce pressure to give a yellow oil. Then water was added to the yellow oil and a white solid precipitated. The white solid was purified by preparative RP-HPLC (water to ACN 95:5 in 20 min). Fractions containing the desired product were lyophilized to afford bioconjugate **15a** (7.20 mg, 10.7 µmol, 54% yield) as a white solid (retention time 11.5 min).

^{1}H NMR ^{1}H NMR (400 MHz, MeOD) δ 7.49 (d, *J* = 8.8 Hz, 1H, N*H*), 7.43 (d, *J* = 8.8 Hz, 2H, Ar*H*), 7.30 – 7.12 (m, 7H, Ar*H*), 6.94 (d, *J* = 8.6 Hz, 2H, Ar*H*), 6.78 – 6.72 (m, 2H, Ar*H*), 6.06 (s, 1H, C*H*-Ar), 4.61 (dd, *J* = 9.4, 5.6 Hz, 1H, C*H*-Phe), 4.58 – 4.48 (m, 1H, C*H*-Tyr), 4.36 – 4.23 (m, 1H, C*H*-Ala), 3.82 (q, *J* = 7.0 Hz, 1H, C*H*-Ala), 3.73 (s, 3H, OCH_3), 3.40 (t, *J* = 6.7 Hz, 2H, $CH_2CH_2N_3$), 3.15 – 3.03 (m, 2H, CH_2-Tyr, CH_2-Phe), 2.98 – 2.79 (m, 2H, CH_2-Tyr, CH_2-Phe), 2.52 (t, *J* = 6.7 Hz, 2H, $CH_2CH_2N_3$), 1.43 (d, *J* = 7.1 Hz, 3H, CH_3), 1.33 (dd, *J* = 7.2, 2.6 Hz, 3H, CH_3). **^{13}C NMR** (101 MHz, MeOD) δ 177.1, 173.0, 172.6, 170.9, 160.1, 155.5, 148.0, 138.2, 132.3, 132.0, 131.0, 131.5, 130.2, 129.5, 127.9, 118.4, 117.6, 114.7, 57.7, 56.0, 55.6, 50.1, 50.0, 49.8, 38.6, 38.0, 34.0, 18.4, 17.7. **HRMS** (ESI/QTOF) m/z: $[M + H]^+$ Calcd for $C_{35}H_{43}N_8O_6^+$ 671.3300; Found 671.3301.

HPLC-UV chromatogram (214 nm) of crude of the reaction:

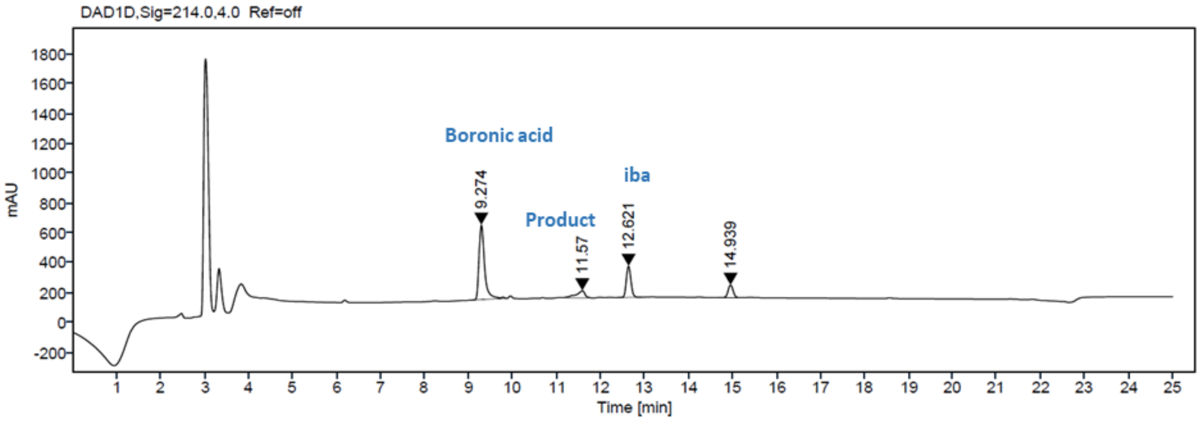

HPLC-UV chromatograms (214 nm) of isolated **15a**:

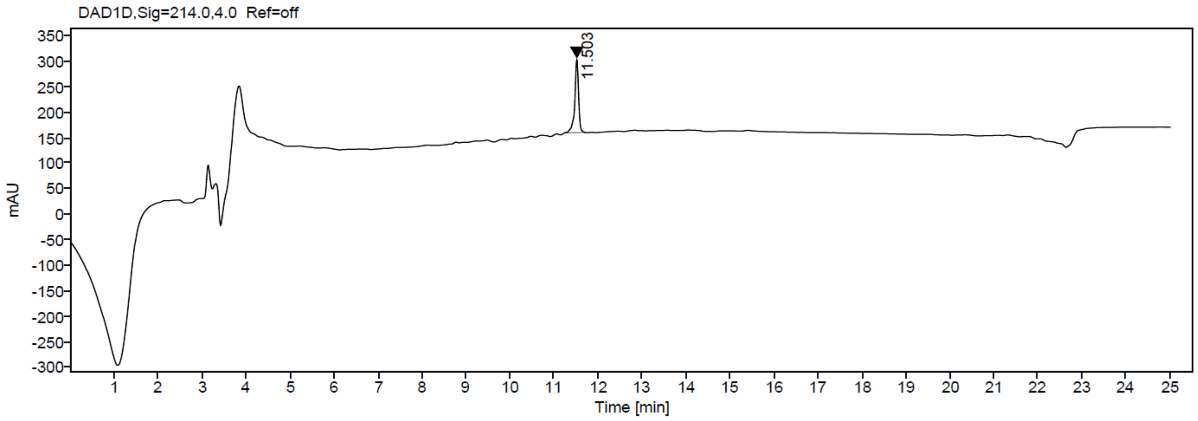

d. One pot-two steps Suzuki:

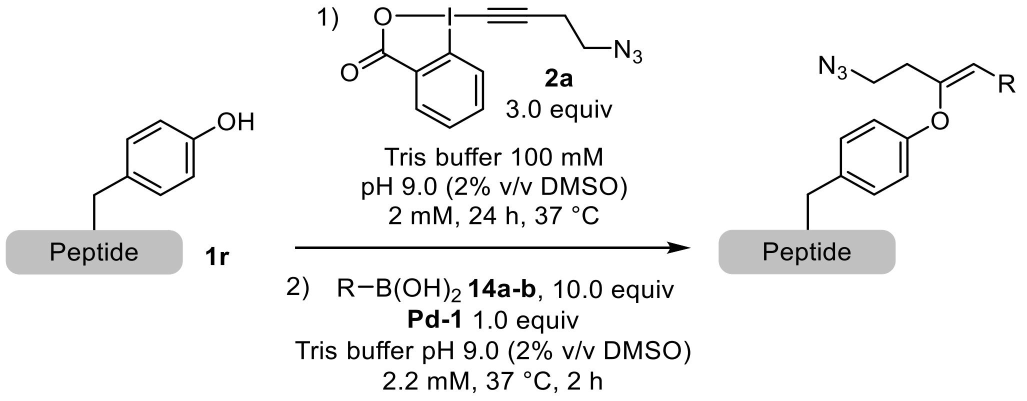

General procedure:

To a solution of β-Casomorphin (**1r**) (864 µg, 1.00 µmol, 1.00 equiv) in 100 mM Tris buffer pH 9.0 (490 µL), in a 1.5 mL Eppendorf Safe-Lock microcentrifuge tube, was added a 300 mM solution of N_3-EBX (**2a**) (512 µg, 1.50 µmol, 1.50 equiv) in DMSO (10 µL). The 2.00 mM solution was shaken at 37 °C for 24 h. No effort was made to exclude oxygen. The reaction O-VBX formation was analyzed by HPLC-MS. Separately, a 0.5 mL Eppendorf Safe-Lock microcentrifuge tube was charged with boronic acid (**14a-b**) (10.0 µmol, 10.0 equiv.), Tris buffer (100 mM, pH 9.0, 290 µL) and DMSO (10 µL). The resulting mixture was shaken at 37 °C until complete dissolution. The 33.3 mM solution of phenyl boronic acid was then added to the crude containing the O-VBX of β-Casomorphin. The resulting solution was vortexed few seconds to ensure proper reagent mixing and incubated at 37 °C over 10 minutes. Then, a 10.0 mM solution of palladium complexe (**Pd-1**) in water (100 µL, 1.00 µmol, 1.00 equiv.) was added in one portion. The resulting solution was vortexed few seconds to ensure proper reagent mixing and incubated at 37 °C for 2 hours. The reaction was then quenched with a 570 mM solution of 3-mercaptopropionic acid in water (2.00 µL, 1.14 µmol, 1.14 equiv. per equiv. of palladium) and shaken at room temperature for 10 minutes. The reaction was analyzed by HPLC-MS.

The yields are given over two steps by HPLC-UV. The yields were approximated as the ratio of Aprod/Atotal where Aprod = area in mAU of the product peak and Atotal = area in mAU of all peptides products (product, starting material, and side products if present).

Bioconjugate (16a)

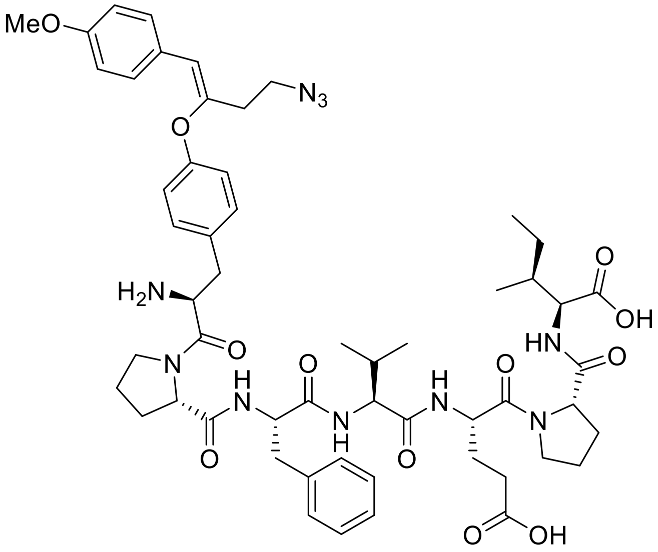

Following the general procedure, (para-methoxyphenyl)boronic acid (**14a**) (1.52 mg, 10.0 μmol, 10.0 equiv.) afforded bioconjugate (**16a**) in 78% yield based on HPLC-UV (retention time 12.5 min).

HRMS (nanochip-ESI/LTQ-Orbitrap) m/z: $[M + H]^+$ Calcd for $C_{55}H_{73}N_{10}O_{12}^+$ 1065.5404; Found 1065.5393.

MS/MS characterization:

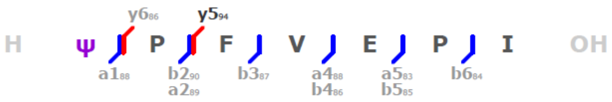

ψ = Tyr(C11H11N3O)

Sequence	Type	MF	m/z	Intensity	Similarity
**YPFVE**	b5	C44H53N8O9(+1)	837.3907	100.64	0.85
**YPFVE**	a5	C43H53N8O8(+1)	809.3978	14.26	0.85
**YPFV**	b4	C39H46N7O6(+1)	708.3485	12.93	0.86
**YPF**	b3	C34H37N6O5(+1)	609.2802	9.43	0.87
**YPFV**	a4	C38H46N7O5(+1)	680.3536	5.89	0.88
**YPFVE**	b5	C44H51N8O8(+1)	819.3802	2.58	0.84
**YPFVEP**	b6	C49H60N9O10(+1)	934.4432	2.35	0.84
PFVEPI	y6	C35H53N6O9(+1)	701.3849	2.01	0.86
**YP**	a2	C24H28N5O3(+1)	434.2174	1.81	0.89
**YP**	b2	C25H28N5O4(+1)	462.2124	1.54	0.90
**YPFVE**	a5	C43H51N8O7(+1)	791.3868	1.11	0.80
**Y**	a1	C19H21N4O2(+1)	337.1651	0.96	0.88
FVEPI	y5	C30H46N5O8(+1)	604.3324	0.52	0.94

HPLC-UV chromatograms (214 nm) of crude of the reaction:

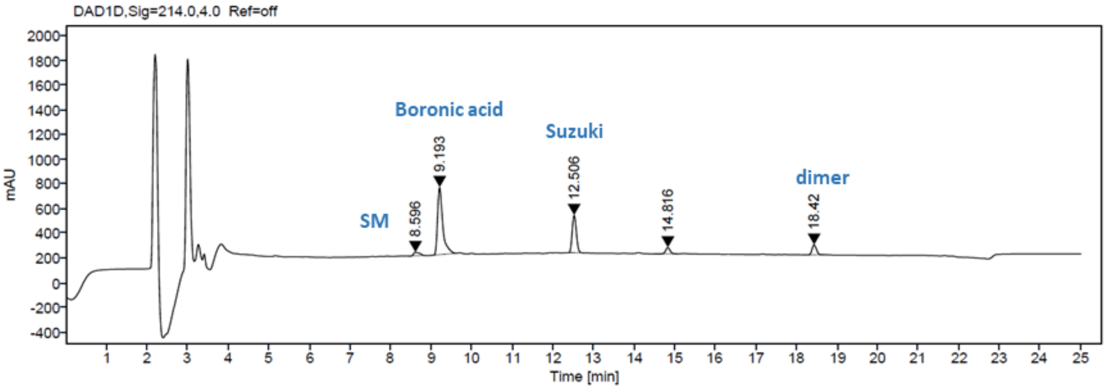

Bioconjugate (16b)

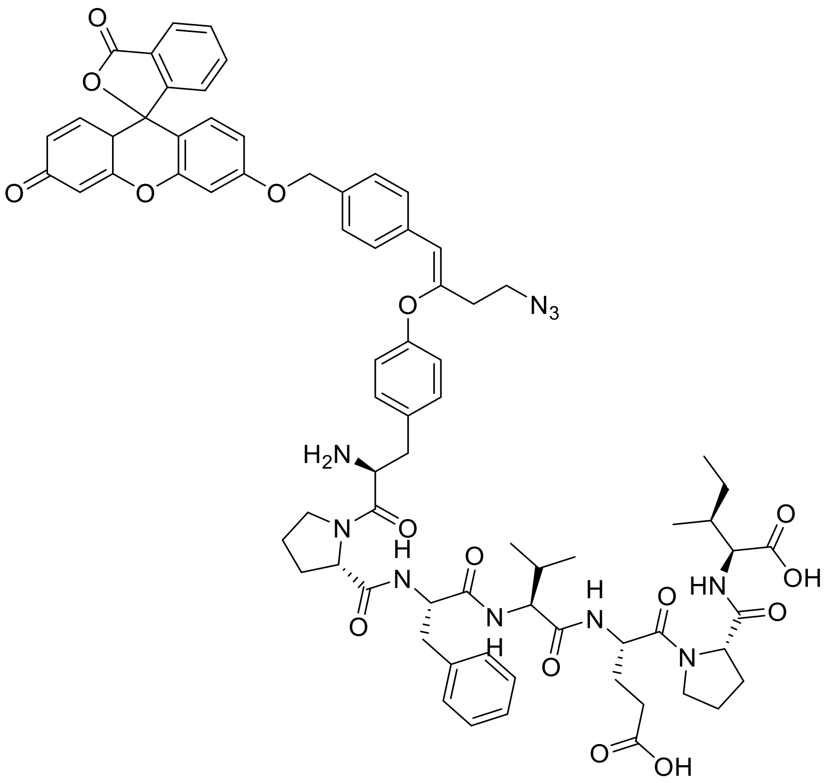

Following the general procedure, with 10.0 equiv of **Pd-1**, 20% DMSO and 4 hours of reaction time, Fluorescein boronic acid (**14b**) (4.66 mg, 10.0 µmol, 10.0 equiv.) afforded bioconjugate (**16b**) in 30% yield based on HPLC-UV (retention time 13.3 min).

HRMS (nanochip-ESI/LTQ-Orbitrap) m/z: $[M + H]^+$ Calcd for $C_{75}H_{83}N_{10}O_{16}^+$ 1379.5983; Found 1379.5978.

MS/MS characterization:

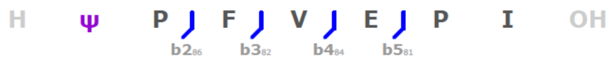

ψ = Tyr(C31H21N3O5)

Sequence	Type	MF	m/z	Intensity	Similarity
**YPFVE**	b5	C64H63N8O13(+1)	1151.4509	63.61	0.80
**YP**	b2	C45H38N5O8(+1)	776.2715	28.90	0.86
**YPFV**	b4	C59H56N7O10(+1)	1022.4083	11.65	0.84
**YPFVE**	b5	C64H61N8O12(+1)	1133.4403	4.79	0.82
**YPF**	b3	C54H47N6O9(+1)	923.3399	2.62	0.82

HPLC-UV chromatograms (214 nm) of crude of the reaction:

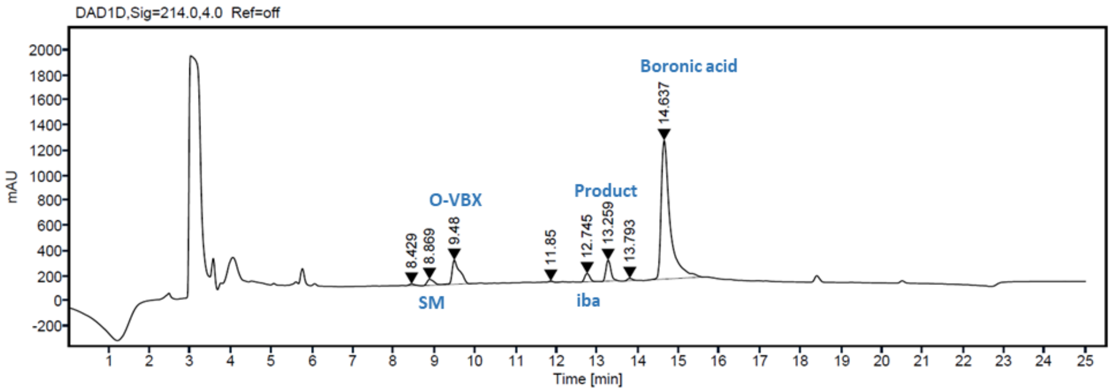

11. Labelling/SPAAC/Suzuki

Bioconjugate (17)

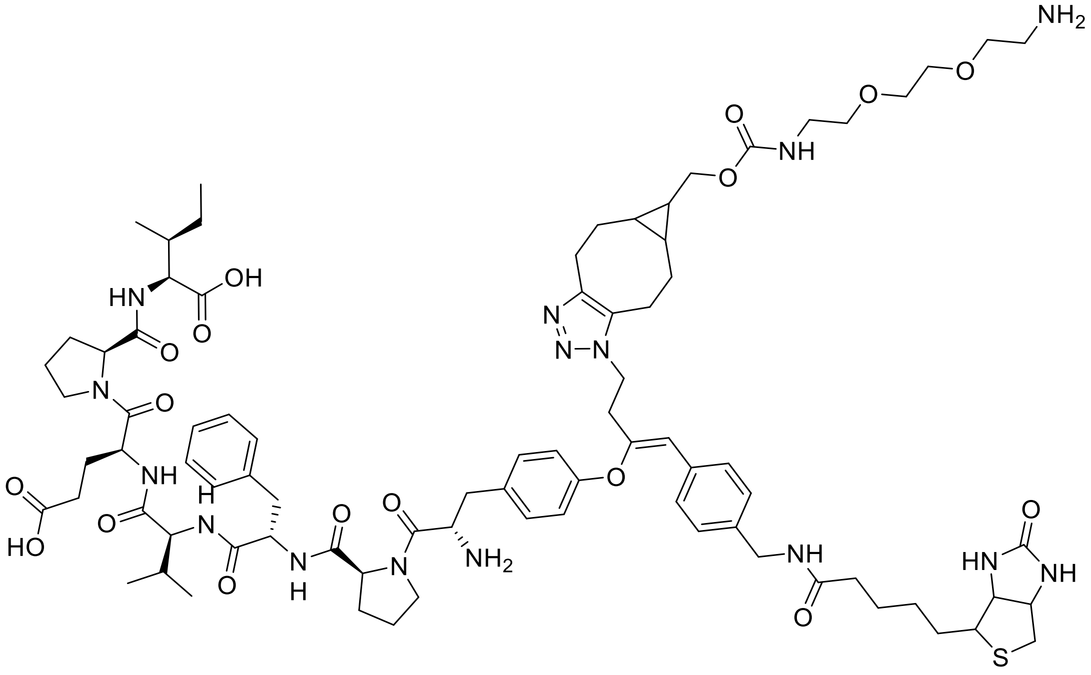

To a solution of β-Casomorphin (**1r**) (864 µg, 1.00 µmol, 1.00 equiv) in 100 mM Tris buffer pH 9.0 (490 µL), in a 1.5 mL Eppendorf Safe-Lock microcentrifuge tube, was added a 300 mM solution of N_3-EBX (**2a**) (512 µg, 1.50 µmol, 1.50 equiv) in DMSO (10 µL). The 2.00 mM solution was shaken at 37 °C for 24 h. No effort was made to exclude oxygen. The for reaction O-VBX formation was analyzed by HPLC-MS. Separately, a 0.5 mL Eppendorf Safe-Lock microcentrifuge tube was charged with boronic acid (**14c**) (3.78 mg, 10.0 µmol, 10.0 equiv.), Tris buffer (100 mM, pH 9.0, 290 µL) and DMSO (10 µL). The resulting mixture was shaken at 37 °C until complete dissolution. The 33.3 mM solution of phenyl boronic acid was then added to the crude containing the O-VBX of β-Casomorphin. The resulting solution was vortexed few seconds to ensure proper reagent mixing and incubated at 37 °C over 10 minutes. Then, a 10.0 mM solution of palladium complexe (**Pd-1**) in water (100 µL, 1.00 µmol, 1.00 equiv.) was added in one portion. The resulting solution was vortexed few seconds to ensure proper reagent mixing and incubated at 37 °C for 5 hours. Then, a 30.0 mM solution of cyclooctyne reagent (**10a**) in DMSO (100 µL, 3.00 µmol, 3.00 equiv.) was added. The resulting mixture was shaken at room temperature for 2 hours. No effort was made to exclude oxygen. The reaction was analyzed by HPLC-MS.

The yield is given over three steps by HPLC-UV. The yields were approximated as the ratio of Aprod/Atotal where Aprod = area in mAU of the product peak and Atotal = area in mAU of all peptides products (product, starting material, and side products if present).

Following the procedure on β-Casomorphin (**1r**), Suzuki reaction with boronic acid (**14c**) and SPAAC with BCN (**10a**) afforded bioconjugate (**17**) in 71% yield based on HPLC-UV (retention time 9.2 min).

HRMS (ESI/QTOF) m/z: $[M + H_2]^{+2}$ Calcd for $C_{82}H_{117}N_{15}O_{17}S^{+2}$ 807.9231; Found 807.9191.

MS/MS characterization:

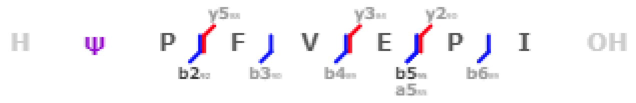

ψ = Tyr(C38H54N8O6S)

Sequence	Type	MF	m/z	Intensity	Similarity
**YPFVE**	**b5**	**C71H96N13O14S(+1)**	**693.8494**	**48.63**	**94%**
**YPFVE**	**b5**	**C71H96N13O14S(+1)**	**1386.692**	**28.7**	**96%**
**YP**	**b2**	**C52H71N10O9S(+1)**	**1011.512**	**13.04**	**92%**
**YPFV**	**b4**	**C66H89N12O11S(+1)**	**1257.649**	**9.76**	**92%**
FVEPI	y5	C30H46N5O8(+1)	604.3341	4.22	88%
**YPFVEP**	**b6**	**C76H103N14O15S(+1)**	**742.3758**	**3.44**	**89%**
**YPF**	**b3**	**C61H80N11O10S(+1)**	**1158.581**	**2.93**	**89%**
**YPFV**	**b4**	**C66H89N12O11S(+1)**	**629.3281**	**2.81**	**85%**
PI	y2	C11H21N2O3(+1)	229.1547	2.51	90%
EPI	y3	C16H28N3O6(+1)	358.1973	2.44	84%
**YPF**	**b3**	**C61H80N11O10S(+1)**	**579.7939**	**0.9**	**90%**
**YPFVE**	**a5**	**C70H96N13O13S(+1)**	**679.8519**	**0.81**	**85%**

HPLC-UV chromatograms (214 nm) of crude of the reaction:

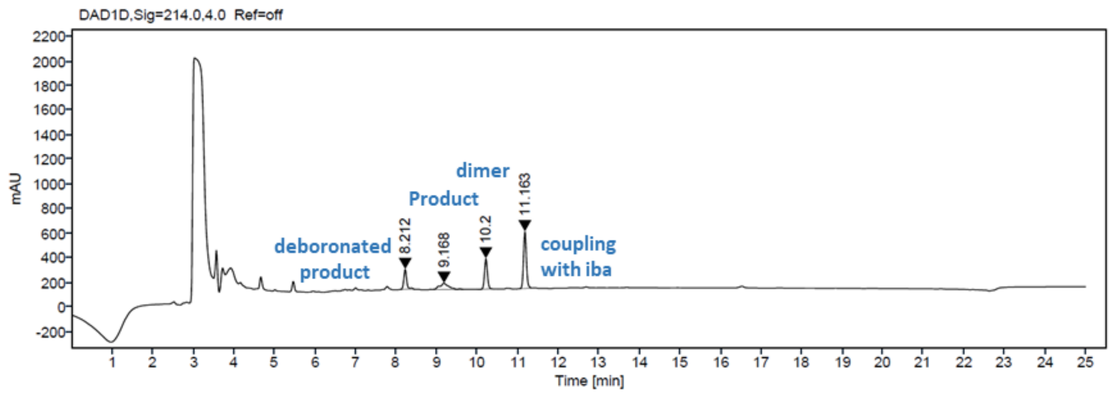

HPLC-MS chromatograms of crude of the reaction:

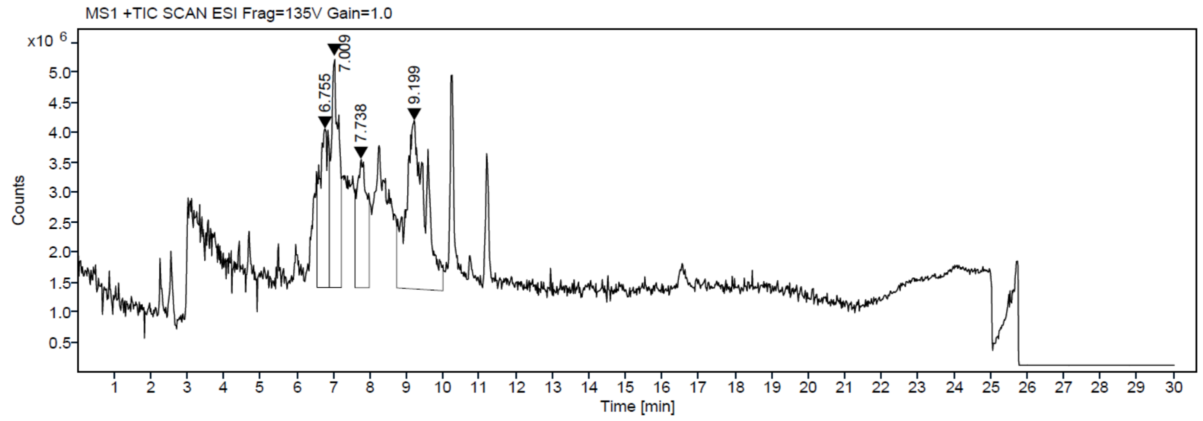

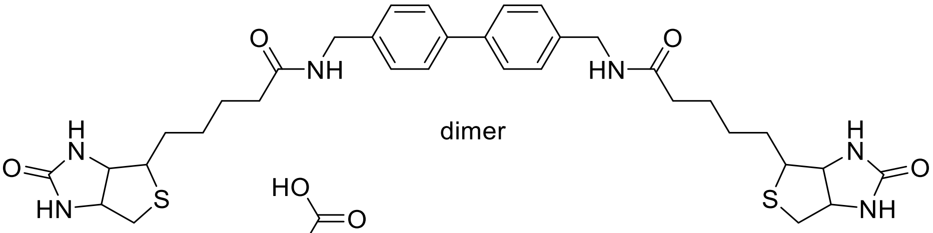

dimer

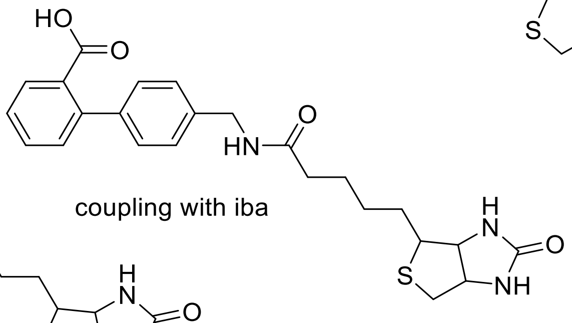

coupling with iba

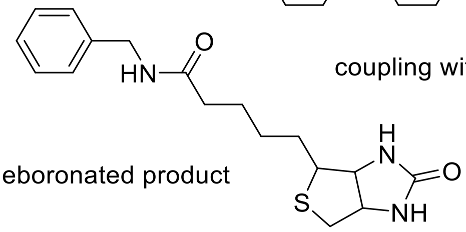

deboronated product

12. Jagaricin fluorescent microscopy experiment

Candida albicans (SC5314) was inoculated into YPD liquid medium (1% yeast extract, 2% peptone, 2% D-glucose) and incubated overnight at 30°C with shaking. The overnight culture was washed 2× with PBS and 1×10^4 cells were seeded in an 8-chambered microscopy slide (ibidi, Germany) in RPMI (ThermoFisher Scientific). The cells were incubated for 3 h at 37°C with 5% CO_2 to induce hypha formation. The cells were then washed 3× with PBS and stained for 30 min at room temperature with jagaricin conjugated to rhodamine (10µg/mL in PBS). The cells were washed 3× with PBS and fixed with Histofix 4% formaldehyde (Roth, Germany) for 30 min at room temperature. The cells were washed 3× with PBS and stained with calcofluor white (10µg/mL in 0.1M TrisHCl [pH 9]; Sigma-Aldrich) for 30 min at room temperature to visualize the cell wall. The cells were washed 3× with water and imaged with a Zeiss Axio Observer (Zeiss, Germany).

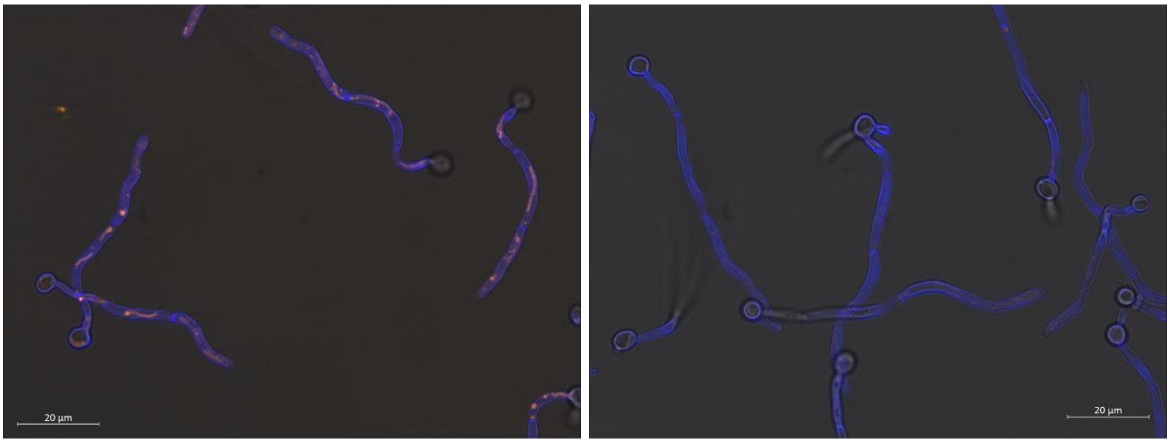

Figure S1: Brightfield images of *C. albicans* incubated with jagaricin-conjugate/calcofluor white (left) and rhodamine B/calcofluor white (right) overlayed with the fluorescence signals for calcofluor white stain (blue) and rhodamine B (orange). Microscope parameters are identical for both experiments.

The jagaricin-conjugate shows a distinct and specific staining pattern in comparison to rhodamine B, which shows little to no fluorescent signal. The jagaricin-conjugate does not bind the surface of the fungal cells, as it does not co-localize with the chitin in the cell wall (stained with calcofluor white). Instead the jagaricin-conjugate seems to localize within both hypha and yeast cells.

13. Cellular uptake experiments

Wild-type Streptavidin (66 kDa) was a generous gift provided by Prof. Thomas R. Ward (University of Basel). HeLa MZ cells were kindly provided by ACCESS Geneva. Phosphate buffered saline (PBS, pH = 7.4), DMEM (GlutaMAX, 4.5 g/L D-Glucose, with phenol red) medium, FluoroBrite DMEM (high D-Glucose) medium, Penicillin-Streptomycin, Fetal Bovine Serum, TrypLE Express Enzyme and Pierce™ Coomassie Plus (Bradford) Assay Reagent were obtained from Thermo Fisher Scientific. Hoechst 33342 (HOE, 10 mg/mL solution in water) was obtained from Invitrogen by Thermo Fisher Scientific. µ-Plate 96-Well Black were obtained from Ibidi. MALDI MS analyses of functionalised streptavidin were performed with SA as a matrix using Bruker MALDI Autoflex Speed TOF/TOF. Fluorescence cellular imaging was performed using an IXM-C automated microscope from ImageXpress equipped with a Lumencor Aura III with 5 independently selectable solid-state light sources, bandpass filters and 5 objectives (4x to 60x). Sample preparation and washing on µ-Plate 96-well black was performed using a Plate washer Biotek EL406®.

Abbreviations: BTTAA: 2-(4-((Bis((1-(tert-butyl)-1H-1,2,3-triazol-4- yl)methyl)amino)methyl)-1H-1,2,3-triazol-1-yl)acetic acid; DMEM: Dulbecco's modified eagle medium; DMSO: Dimethyl sulfoxide; FBS: Fetal bovine serum; HOE: Hoechst 33342; MALDI: Matrix-assisted laser desorption/ionization; PBS: Phosphate buffered saline; SA: Sinapinic acid; TAMRA: 5-Carboxytetramethylrhodamine.

The streptavidin mutants were expressed using an *E. coli* expression strain and purified subsequently.

Streptavidin monomer sequence:
MASMTGGQQMGRDQAGITGTWYNQLGSTFIVTAGADGALTGTYESAVGNAESRYVLTGRY
DSAPATDGSGTALGWTVAWKNNYRNAHSATTWSGQYVGGAEARINTQWLLTSGTTEANA
WKSTLVGHDTFTKVKPSAASIDAAKKAGVNNGNPLDAVQQ

Synthesis of transporters

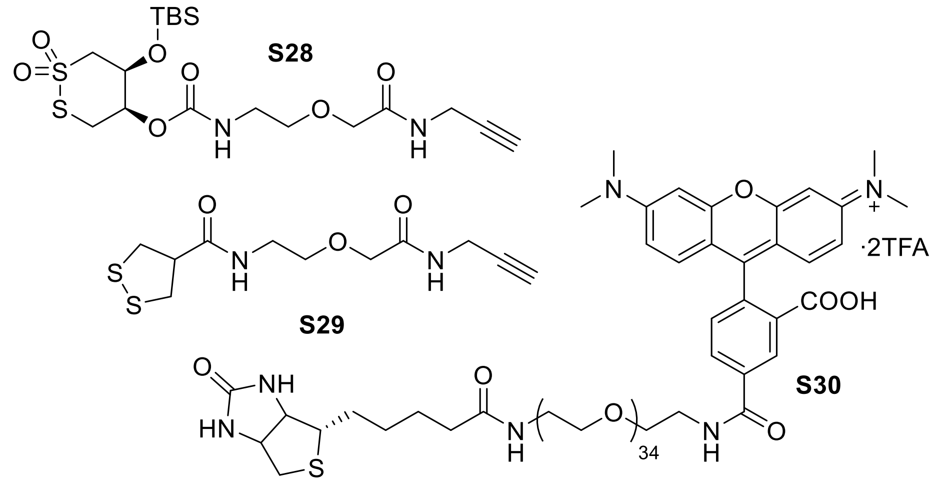

The synthesis of **TAMRA-biotin S30** was prepared according to the literature procedure.[30]

Synthesis of transporter **S28** and **S29**:

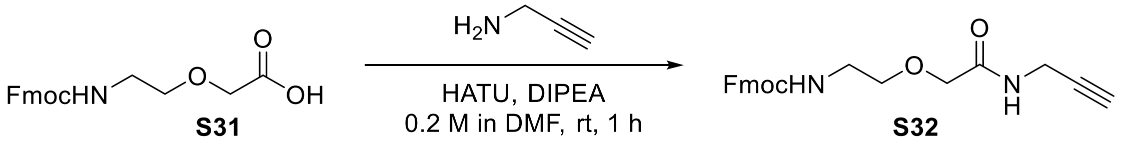

To a solution of 2-(2-aminoethoxy)-N-(prop-2-yn-1-yl)acetamide **S31** (362 mg, 1.06 mmol, 1.00 eq.) in dry DMF (5.30 mL) was added HATU (443 mg, 1.17 mmol, 1.10 eq.), followed by DIPEA (203 µL, 1.17 mmol, 1.10 eq.). The mixture was stirred for 1 min, then propargylamine (81.4 µL, 1.27 mmol, 1.20 eq.) was added, and the reaction was stirred for 1 h. At this point, the reaction was diluted with EtOAc, and washed with $NaHCO_3$ (x3), 10% citric acid (x3), 5% LiCl (x2) and brine (x1). The organics were dried over Na_2SO_4, filtered, and concentrated *in vacuo*. Flash chromatography (0 to 10% MeOH in CH_2Cl_2) gave **S32** (380 mg, 1.00 mmol, 95%) as a white foam.

^{1}H NMR (300 MHz, $CDCl_3$) δ 7.77 (d, *J* = 7.7 Hz, 2H, Ar*H*), 7.59 (d, *J* = 7.4 Hz, 2H, Ar*H*), 7.41 (t, *J* = 7.1 Hz, 2H, Ar*H*), 7.31 (td, *J* = 7.4, 1.3 Hz, 2H, Ar*H*), 6.81 (s, 1H, N*H*), 5.13 (s, 1H, N*H*), 4.45 (d, *J* = 6.7 Hz, 2H, C*H₂*O), 4.22 (t, *J* = 6.7 Hz, 1H, C*H*), 4.07 (dd, *J* = 5.6, 2.6 Hz, 2H, C*H₂*C), 4.01 – 3.95 (m, 2H, OC*H₂*CO), 3.59 (t, *J* = 5.1 Hz, 2H, NCH_2C*H₂*O), 3.43 (q, *J* = 5.5 Hz, 2H, NC*H₂*CH_2O), 2.19 (t, *J* = 2.6 Hz, 1H, CC*H*). **^{13}C NMR** (75 MHz, $CDCl_3$) δ 169.1, 156.7, 144.0, 141.5, 127.9, 128.0, 125.1, 120.2, 79.4, 71.8, 71.0, 70.5, 66.9, 47.4, 40.9, 28.6. **LR-ESI-MS (+ve)** m/z: 401.2 $[M+Na]^+$

[30] López-Andarias, J.; Saarbach, J.; Moreau, D.; Cheng, Y.; Derivery, E.; Laurent, Q.; González-Gaitán, M.; Winssinger, N.; Sakai, N.; Matile, S. *J. Am. Chem. Soc.* **2020**, *142* (10), 4784–4792.

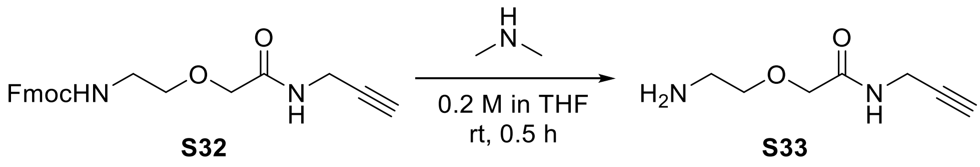

S32 (480 mg, 1.27 mmol, 1.00 eq.) was dissolved in 2 M dimethylamine in THF (6.34 mL) and stirred for 30 min. At this point, the reaction was concentrated *in vacuo*, and the product dissolved in a minimal volume of CH_2Cl_2. The product was then precipitated in pentane and filtered over a bed of Na_2SO_4, washing with additional pentane. The product was isolated by washing the bed of Na_2SO_4 with CH_2Cl_2, and the precipitation procedure was repeated twice more. The product was then used in the next step without further purification. **S33** (174 mg, 1.11 mmol, 88%) was obtained as a yellow oil.

^{1}H NMR (300 MHz, MeOD) δ 4.02 (d, *J* = 2.5 Hz, 2H, C*H₂*C), 4.00 (s, 2H, N*H₂*), 3.57 (t, *J* = 5.1 Hz, 2H, NCH_2CH_2O), 2.88 (t, *J* = 5.1 Hz, 2H, NCH_2CH_2O), 2.59 (t, *J* = 2.6 Hz, 1H, CC*H*).**^{13}C NMR** (75 MHz, MeOD) δ 172.2, 80.6, 73.4, 72.0, 71.1, 41.9, 28.9. **LR-ESI-MS (+ve)** m/z: 157.1 $[M+H]^+$

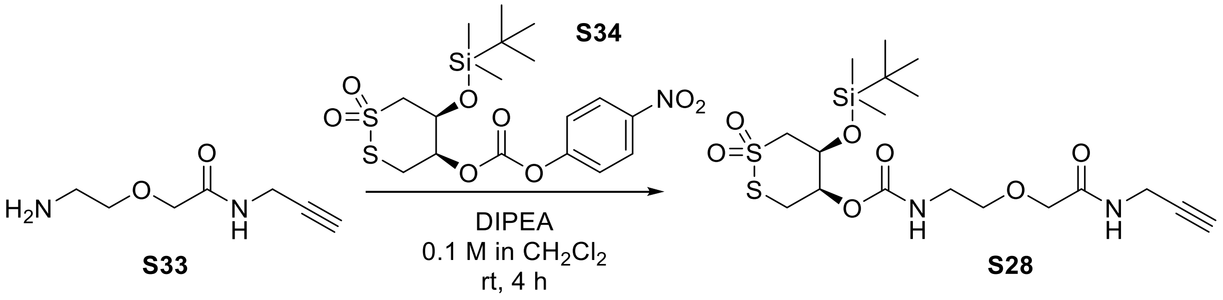

A solution of **S33** (14.2 mg, 91.1 µmol, 1.20 eq.) and DIPEA (15.9 µL, 91.1 µmol, 1.20 eq.) was prepared in CH_2Cl_2 (759 µL) and added to a flask containing **CTO-PNP ester S34** (35.2 mg, 75.9 µmol, 1.00 eq.).[31] The reaction was stirred for 4 h at ambient temperature, and the reaction was then concentrated *in vacuo*. The crude residue was redissolved in EtOAC, and washed with $NaHCO_3$ (x3), 10% citric acid (x3), and brine. The combined organics were dried over Na_2SO_4, filtered, and concentrated *in vacuo*. Flash chromatography (0 to 50% EtOAc in CH_2Cl_2) gave **S28** (25.0 mg, 51.6 µmol, 68%) as a colourless oil.

^{1}H NMR (400 MHz, $CDCl_3$) δ 6.78 (s, 1H, N*H*), 5.35 (t, *J* = 6.0 Hz, 1H, N*H*), 5.27 (q, *J* = 3.0 Hz, 1H, C*H*O), 4.35 (dt, *J* = 10.7, 3.4 Hz, 1H, C*H*O), 4.11 (dd, *J* = 5.5, 2.6 Hz, 2H, CH_2C), 4.00 (s, 2H, OCH_2CO), 3.76 – 3.56 (m, 3H, NCH_2CH_2O, SCH_2C), 3.55 – 3.40 (m, 4H, NCH_2CH_2O, SO_2CH_2C), 3.35 (dd, *J* = 13.0, 3.8 Hz, 1H, SCH_2C), 2.27 (t, *J* = 2.6 Hz, 1H, CC*H*), 0.88 (s, 9H, CH_3), 0.13 (d, *J* = 1.0 Hz, 6H, CH_3). **^{13}C NMR** (101 MHz, $CDCl_3$) δ 168.9, 155.39, 79.3, 71.9, 71.8, 70.6, 70.3, 69.9, 67.1, 61.9, 40.8, 35.2, 28.6, 28.6, 25.6, 25.5, 17.9, -4.9, -4.99. **HR-ESI-MS (+ve)** m/z: $[M+Na]^+$ calc. for $C_{18}H_{32}N_2O_7S_2Si$ 503.1313; Found 503.1298.

[31] CTO-PNP ester **S34** was prepared according to the literature procedure: Kato, T.; Lim, B.; Cheng, Y.; Pham, A.-T.; Maynard, J.; Moreau, D.; Poblador-Bahamonde, A. I.; Sakai, N.; Matile, S. *JACS Au* **2022**, *2* (4), 839–852.

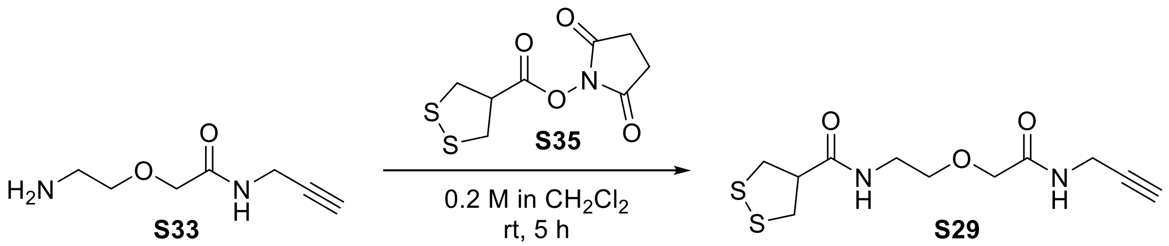

To a solution of **S33** (12.0 mg, 76.8 μmol, 1.00 eq.) in CH_2Cl_2 (384 μL) was added **AspA-NHS S35** (19.0 mg, 76.8 μmol, 1.00 eq.),[32] and the solution was stirred at ambient temperature for 5 h. At this point, the crude mixture was loaded directly onto silica and purified by flash chromatography (0 to 40% MeCN in CH_2Cl_2), giving **S29** (8.30 mg, 28.7 μmol, 37%) as a colourless oil.

^{1}H NMR (400 MHz, $CDCl_3$) δ 6.71 (s, 1H, N*H*), 6.32 (s, 1H, N*H*), 4.12 (dd, *J* = 5.5, 2.6 Hz, 2H, C*H₂*C), 4.01 (s, 2H, OC*H₂*CO), 3.63 (dd, *J* = 5.7, 4.7 Hz, 2H, $NCH_2C\mathit{H_2}O$), 3.54 (td, *J* = 5.6, 4.4 Hz, 2H, $NC\mathit{H_2}CH_2O$), 3.46 – 3.34 (m, 4H, $SC\mathit{H_2}CHC\mathit{H_2}S$), 3.34 – 3.24 (m, 1H, $SCH_2C\mathit{H}CH_2S$), 2.28 (t, *J* = 2.6 Hz, 1H, CC*H*). **^{13}C NMR** (101 MHz, $CDCl_3$) δ 172.2, 169.0, 79.4, 72.0, 70.5, 70.4, 52.6, 42.8, 39.6, 28.7. **HR-ESI-MS (+ve)** m/z: $[M+Na]^+$ calc. for $C_{11}H_{16}N_2O_3S_2$ 311.0495; Found 311.0483.

[32] Aspa-NHS **S35** was prepared according to the literature procedure: Sakai, N.; Lista, M.; Kel, O.; Sakurai, S.; Emery, D.; Mareda, J.; Vauthey, E.; Matile, S. *J. Am. Chem. Soc.* **2011**, *133* (39), 15224–15227.

EBX functionalization of streptavidin

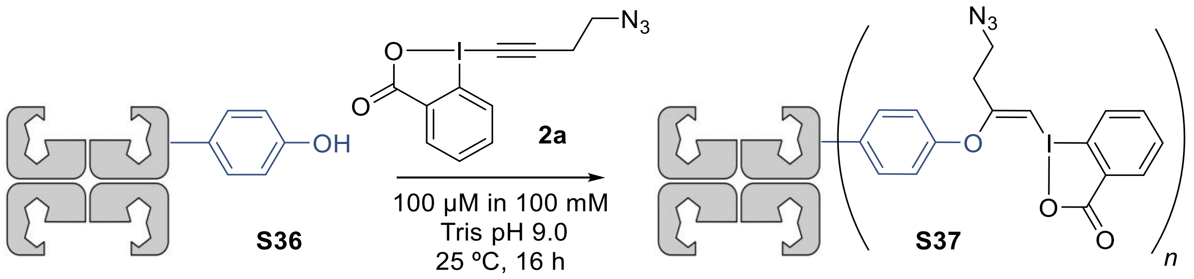

Tyrosine functionalisation with EBX **2a** was performed as per previously optimised conditions B. Samples were purified following functionalisation by passing through a desalting column (PD midiTrap G-25, cytiva), followed by concentration in a centrifugal cutoff filter (Amicon® Ultra 2 mL, 30K) prior to the subsequent reaction.

SEC analysis of streptavidin monomer (z = 8):

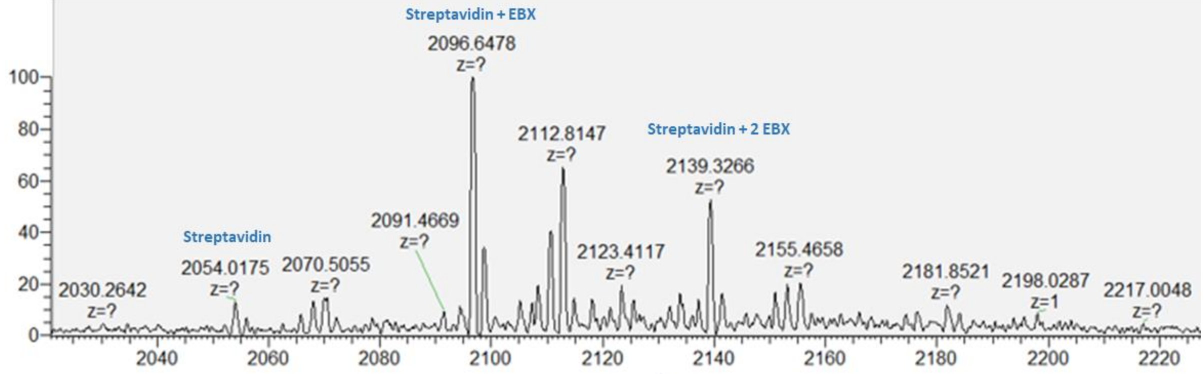

Suzuki coupling on Streptavidin

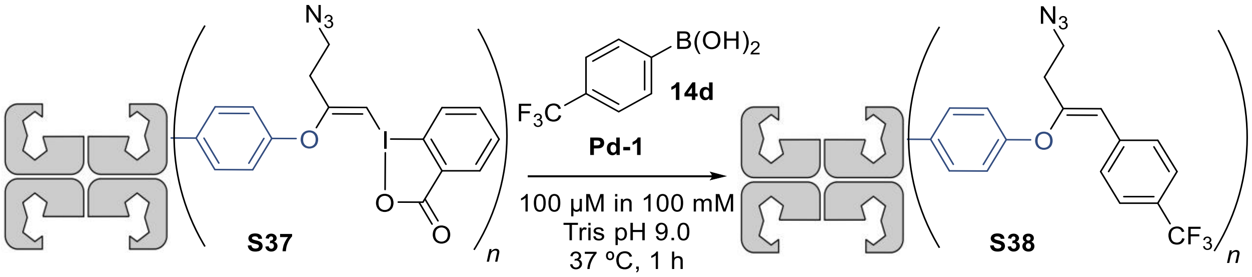

Suzuki coupling on the freshly prepared streptavidin O-VBX conjugate **S37** was performed according to a previously described procedure.[33] To a solution of streptavidin O-VBX conjugate **S37** (100 µM, 1.0 equiv) in Tris buffer (100 mM, pH 9.0) was added a freshly prepared solution of 4-(trifluoromethyl)phenylboronic acid **14d** (25 mM, 50 equiv) in 9:1 Tris buffer (100 mM, pH 9.0) – DMSO, followed by addition of the palladium catalyst (**Pd-1**) in water (20 equiv). The reaction vessel was sealed and heated at 37 ºC for 1 hour whilst shaking at 750 rpm. At this point, the reaction was purified by desalting column (PD midiTrap G-25, cytiva) and concentrated in a centrifugal cutoff filter (Amicon® Ultra 2 mL, 30K) prior to the subsequent reaction.

[33] Tessier, R.; Ceballos, J.; Guidotti, N.; Simonet-Davin, R.; Fierz, B.; Waser, J. *Chem* **2019**, *5* (8), 2243–2263.

CuAAC functionalization

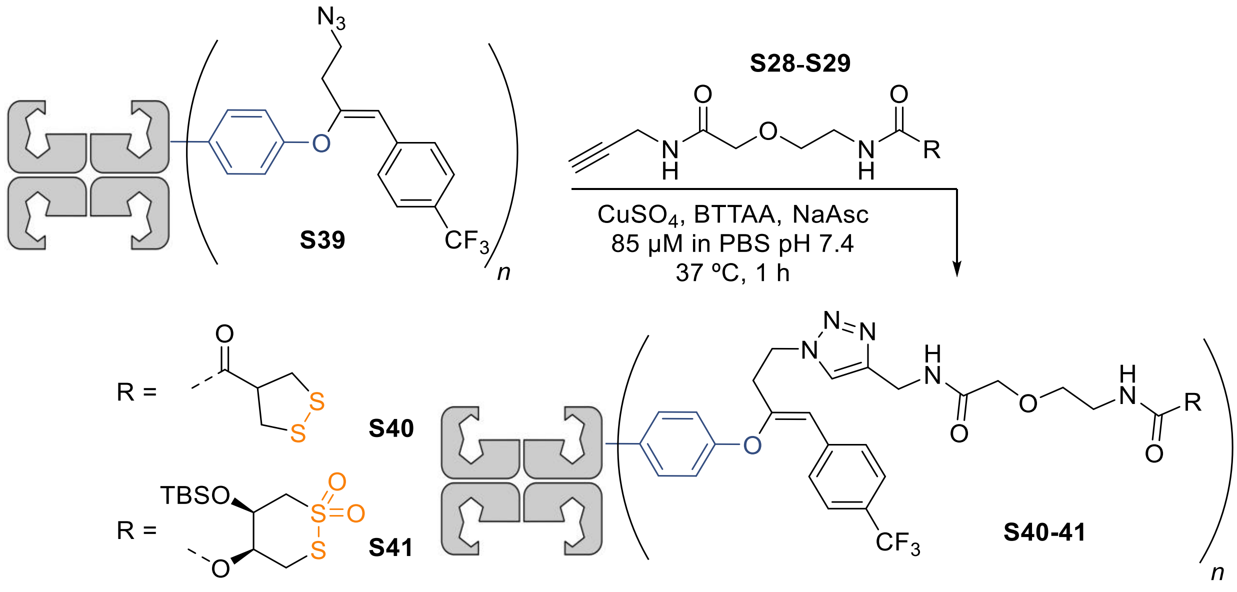

To a solution of the previously prepared streptavidin conjugate **S39** (100 µM in PBS pH 7.4, 1.00 equiv) was added the transporter-alkyne **S28**-**S29** (10 mM in DMSO, 30.0 equiv), followed by a pre-mixed solution of BTTAA ligand (50 mM in H_2O, 40.0 equiv) and $CuSO_4$ (20 mM in H_2O, 10.0 equiv). Finally, a freshly prepared solution of sodium ascorbate (100 mM in water, 400.0 equiv) was added, the reaction vessel was sealed, and shaken at 37 ºC, 750 rpm for 1 hour. At this point, the functionalised protein was purified by first passing through a desalting column (PD midiTrap G-25, cytiva), then concentrating (3 times) in a centrifugal cutoff filter (Amicon® Ultra 2 mL, 30K). The concentrations of the resulting solutions were determined by Pierce Coomassie Plus assay (Thermo Scientific™).

Biotin complexation

The functionalised streptavidin samples (8 µM in PBS pH 7.4) were complexed with 2 equivalents of fluorescently labelled biotin **S30** (2 mM in H_2O) as previously reported,[30] before concentration with a centrifugal cutoff filter (Amicon® Ultra 0.5 mL, 30K) to give 100 µM stock solutions in PBS buffer (pH 7.4). All samples were filtered prior to uptake experiments.

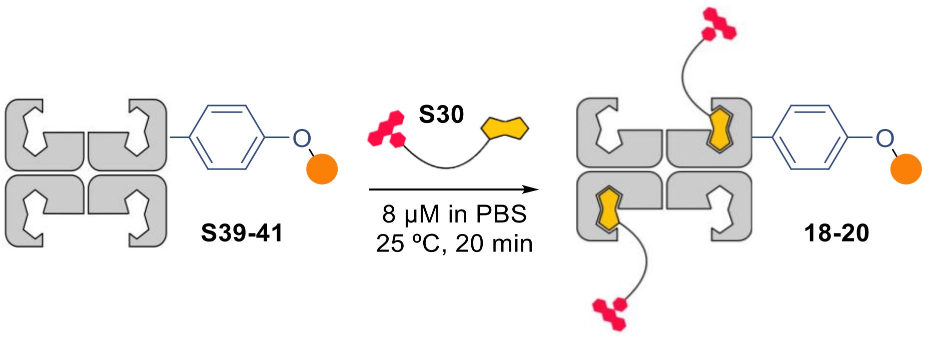

MALDI-MS analysis of functionalised proteins

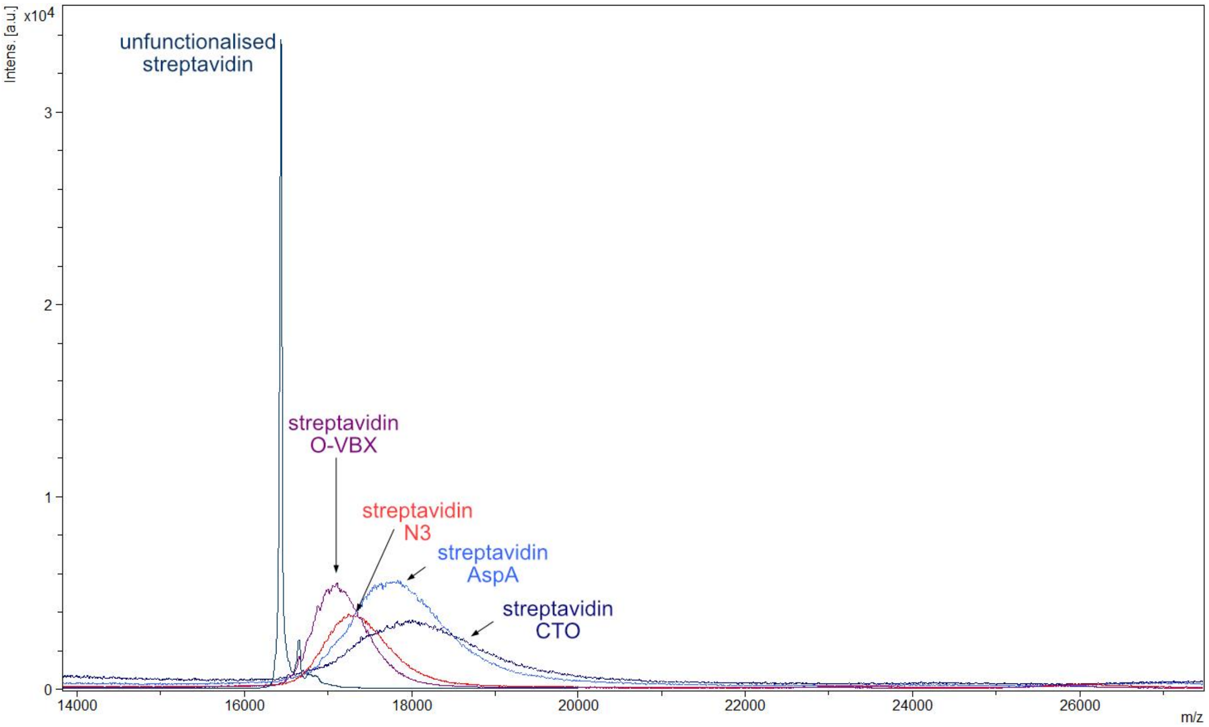

Uptake experiments

HeLa MZ cells were plated at a density of 8x10^4 cells/mL in FluoroBrite DMEM + 10% FBS on µ-Plate 96-well Black ibiTreat sterile and incubated at 37ºC with 5% CO_2 for 24 hours prior to performing uptake experiments. Then, the medium was removed and cells were washed with PBS (3 x 3 mL/well) and fresh FluoroBrite DMEM medium (4 x 150 µL/well) was added into the cells using a plate washer (Biotek EL406®), keeping a final volume of 135 µL/well. Functionalised streptavidin-biotin complexes were added to the wells as 10x stock solutions in PBS (15 µL) to give final concentrations of 10 µM. Cells were incubated with the functionalised proteins for 6 h at 37 ºC with 5% CO_2, before again washing with FluoroBrite DMEM using the plate washer and incubating with a freshly prepared solution of Hoechst 33342 in PBS (15 µL) to give a final volume of 150 µL/well. After 25 minutes, the cells were washed and kept in DMEM FluoroBrite using a plate washer. For live cell imaging, samples were kept at 37 ºC with 5% CO_2 during the microscope imaging. The distribution of fluorescence stained cells was analysed without fixing using an IXM-C automated microscope. 9 images per well were recorded using a 20x objective lens, and the fluorescent images were acquired with two channels, blue for Hoechst 33342 with 377/50 nm excitation filter and 477/60 nm emission filter, and red for TAMRA with 531/40 nm excitation filter and 593/40 nm emission filter. Experiments were performed in triplicate, and data were analysed following a reported procedure.[34]

[34] Kato, T.; Lim, B.; Cheng, Y.; Pham, A.-T.; Maynard, J.; Moreau, D.; Poblador-Bahamonde, A. I.; Sakai, N.; Matile, S. Cyclic Thiosulfonates for Thiol-Mediated Uptake: Cascade Exchangers, Transporters, Inhibitors. *JACS Au* **2022**, *2* (4), 839–852.

Original SDCM images following 6 h incubation with functionalized streptavidin-biotin complexes

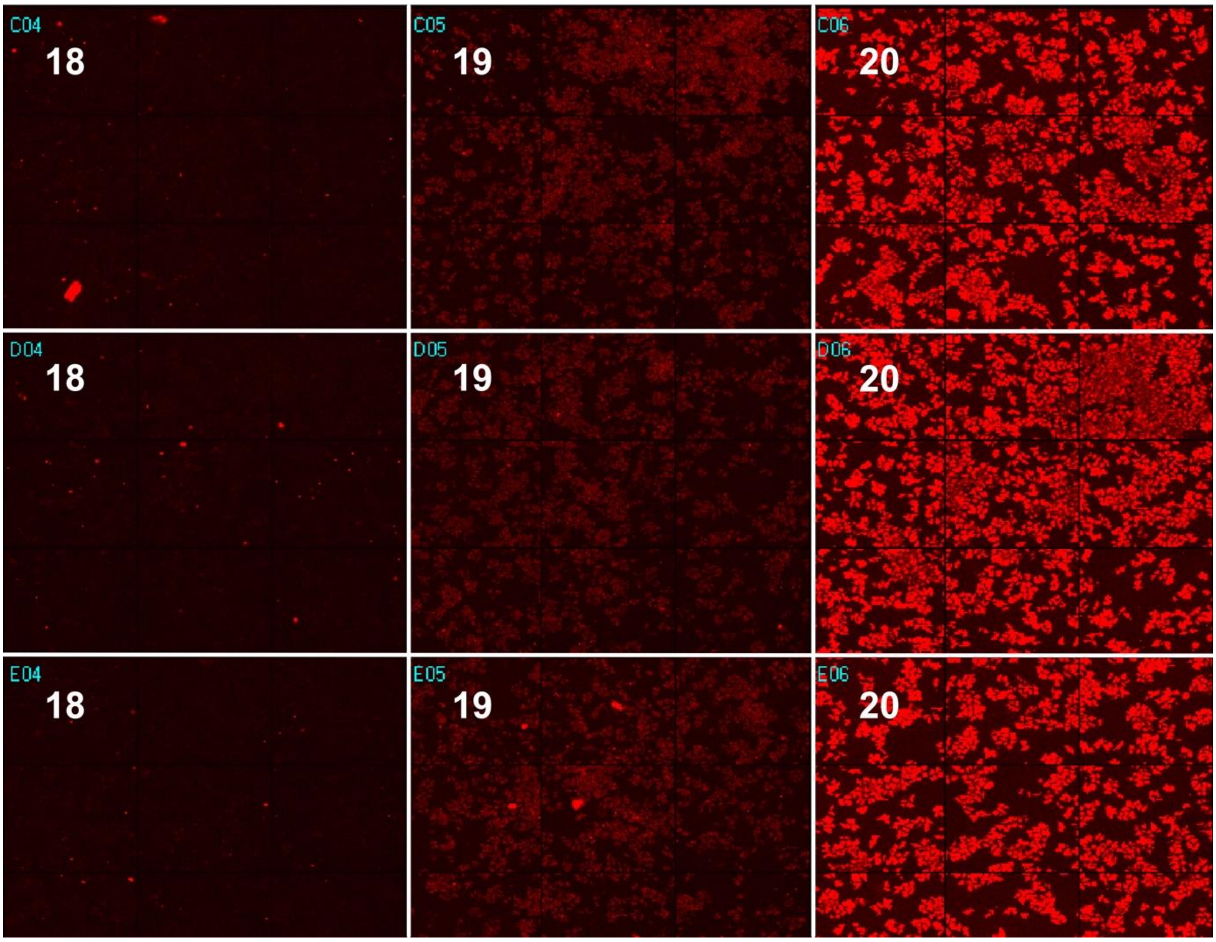

<u>Enlarged image of HeLa MZ cells following 6 h incubation with 10 µM **18**</u>

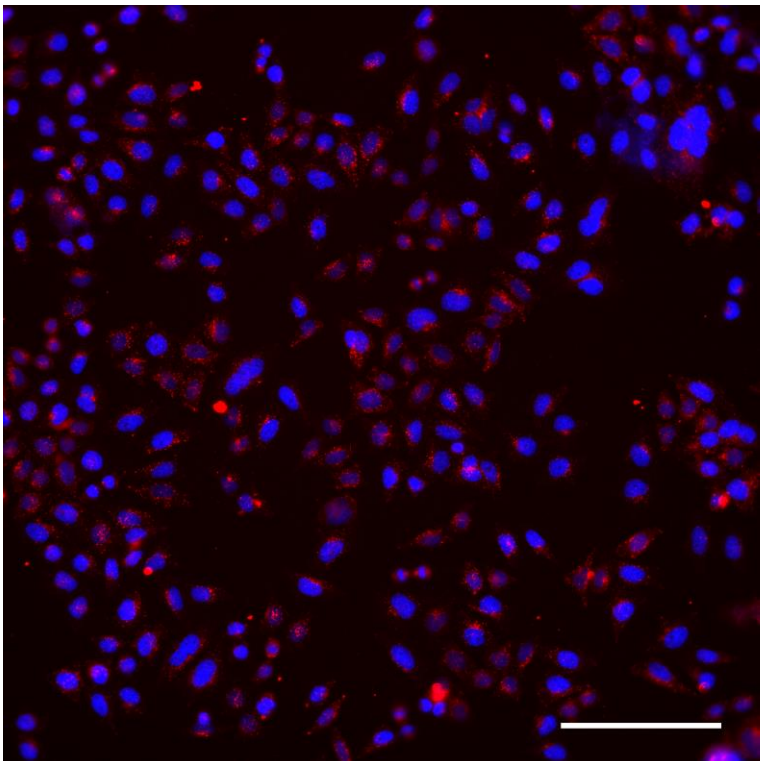

<u>Enlarged image of HeLa MZ cells following 6 h incubation with 10 µM **19**</u>

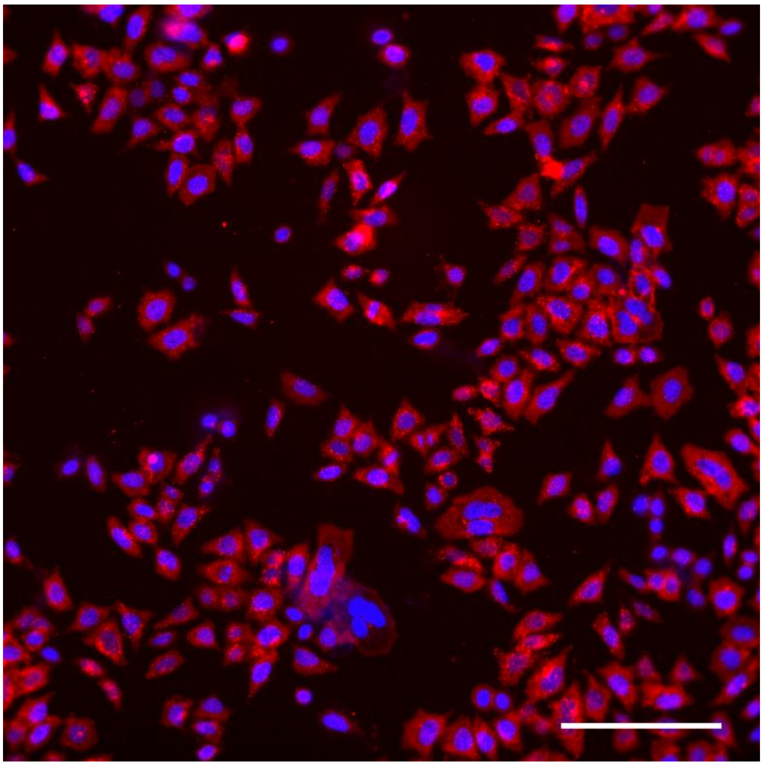

Enlarged image of HeLa MZ cells following 6 h incubation with 10 μM **20**

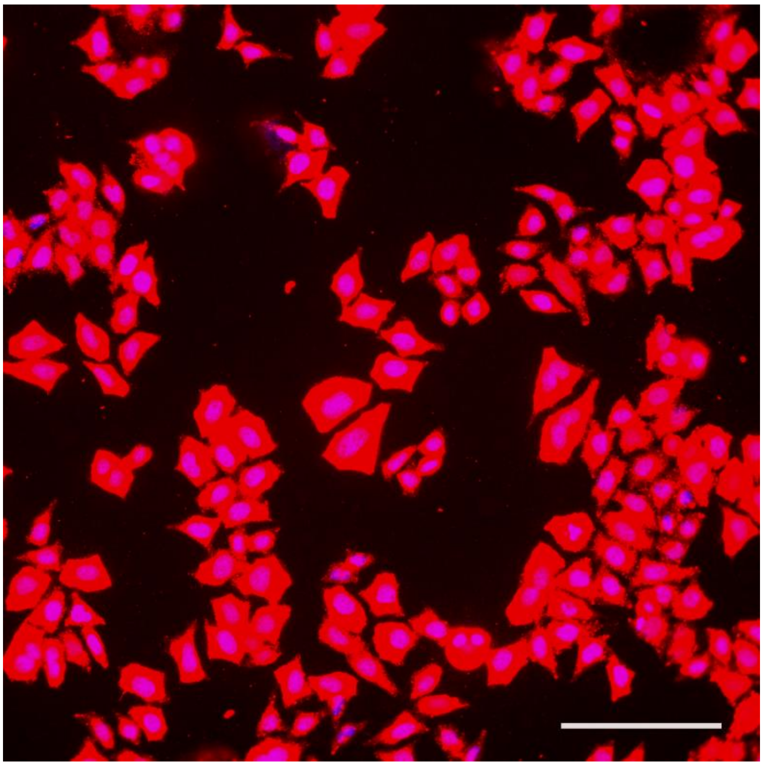

14. Stability evaluation of O-VBX

This structure being reported for the first time, some stability evaluation was performed. The degradation evolution was followed by HPLC-MS.

a. Stability evaluation at low pH

In a 1.5 mL Eppendorf Safe-Lock microcentrifuge tube, O-VBX of AFYA-NH_2 (**3a**) (1.85 mg, 2.00 μmol) was dissolved in an acetic acid buffer (1.0 M, pH 4.0, 1000 μL) and shaken at room temperature. After 2 days, O-VBX of AFYA-NH_2 (**3a**) remained intact.

b. Stability evaluation at high pH

In a 1.5 mL Eppendorf Safe-Lock microcentrifuge tube, O-VBX of AFYA-NH_2 (**3a**) (1.85 mg, 2.00 μmol) was dissolved in a CAPS buffer (10.0 mM, pH 11.0, 1000 μL) and shaken at room temperature. After 2 days, O-VBX of AFYA-NH_2 (**3a**) remained intact.

c. Stability evaluation in presence of glutathione

In a 1.5 mL Eppendorf Safe-Lock microcentrifuge tube, O-VBX of AFYA-NH_2 (**3a**) (1.85 mg, 2.00 μmol) was dissolved in water (1000 μL). Then glutathione (0.614 mg, 2.00 μmol, 1.00 equiv) was added in one portion and the solution was shaken at room temperature. After 2 days, 95% of the O-VBX of AFYA-NH_2 (**3a**) remained intact.

In a 1.5 mL Eppendorf Safe-Lock microcentrifuge tube, O-VBX of AFYA-NH_2 (**3a**) (1.85 mg, 2.00 μmol) was dissolved in milliQ water (1000 μL). Then glutathione (6.14 mg, 20.0 μmol, 10.0 equiv) was added in one portion and the solution was shaken at room temperature. After 2 days, 90% of the O-VBX of AFYA-NH_2 (**3a**) remained intact.

d. Stability evaluation towards temperature

In a 1.5 mL Eppendorf Safe-Lock microcentrifuge tube, O-VBX of AFYA-NH_2 (**3a**) (1.85 mg, 2.00 μmol) was dissolved in milliQ water (1000 μL). The solution was sampled after 10 minutes shaking at 37 °C, 50 °C, 80 °C and 100 °C. Only a minor degradation was noticed at 100 °C.

15. Full mass spectrum for proteins

Ubiquitin, deconvoluted mass spectrum:

Procedure A in denaturing Tris buffer

Xtract Masses Table											
Row Number	Monoisotopic Mass	Average Mass	Sum Intensity	Relative Abundance	Fractional Abundance	Number of Charge States	Charge State Distribution	Average Charge	Delta Mass	Start Time (min)	Stop Time (min)
1	8559.577	8565.04	36063564.51	100.00	30.63	10	5 - 14	10.43	0.000	2.187	2.187
2	8900.549	8906.17	22630462.22	62.75	19.22	10	5 - 14	10.71	340.971	2.187	2.187
3	8575.589	8581.06	9338292.33	25.89	7.93	9	6 - 14	10.44	16.011	2.187	2.187
4	8688.617	8694.14	5888409.00	16.33	5.00	7	7 - 13	10.58	129.039	2.187	2.187
5	8644.603	8650.11	3974873.76	11.02	3.38	8	7 - 14	10.45	85.026	2.187	2.187
6	8916.563	8922.19	3899513.84	10.81	3.31	7	8 - 14	10.73	356.985	2.187	2.187
7	8627.601	8633.10	3763662.97	10.44	3.20	7	7 - 13	10.55	68.024	2.187	2.187
8	8967.570	8973.22	2586783.70	7.17	2.20	8	7 - 14	10.75	407.993	2.187	2.187
9	8731.655	8737.20	2126097.66	5.90	1.81	6	8 - 13	10.54	172.077	2.187	2.187
10	8985.578	8991.23	2099780.99	5.82	1.78	7	8 - 14	10.86	426.000	2.187	2.187
11	8608.613	8614.10	1960545.95	5.44	1.67	7	7 - 13	10.22	49.035	2.187	2.187
12	8747.634	8753.18	1749453.25	4.85	1.49	7	8 - 14	10.78	188.057	2.187	2.187
13	8789.516	8795.08	1683106.00	4.67	1.43	6	8 - 13	10.32	229.938	2.187	2.187
14	8652.613	8658.12	1523071.74	4.22	1.29	6	8 - 13	10.50	93.036	2.187	2.187
15	9025.595	9031.27	1176124.68	3.26	1.00	7	8 - 14	10.80	466.017	2.187	2.187
16	9071.613	9077.32	1159943.75	3.22	0.99	7	8 - 14	10.97	512.035	2.187	2.187
17	8712.636	8718.17	1109872.93	3.08	0.94	6	8 - 13	10.57	153.058	2.187	2.187
18	8661.616	8667.13	1076149.23	2.98	0.91	6	8 - 13	10.64	102.039	2.187	2.187
19	9130.470	9136.21	1022071.07	2.83	0.87	7	8 - 14	10.66	570.893	2.187	2.187
20	8705.639	8711.17	801048.00	2.22	0.68	6	8 - 13	10.46	146.062	2.187	2.187
21	8683.628	8689.15	760257.94	2.11	0.65	4	8 - 12	9.87	124.050	2.187	2.187
22	8668.625	8674.14	755165.21	2.09	0.64	6	8 - 13	10.42	109.048	2.187	2.187
23	9088.598	9094.31	737257.99	2.04	0.63	7	8 - 14	11.12	529.021	2.187	2.187
24	9052.610	9058.30	712526.15	1.98	0.61	6	9 - 14	11.12	493.032	2.187	2.187
25	8774.667	8780.23	630244.29	1.75	0.54	7	8 - 14	10.64	215.089	2.187	2.187
26	8696.624	8702.15	611471.91	1.70	0.52	4	9 - 13	10.31	137.047	2.187	2.187
27	8763.635	8769.19	577844.23	1.60	0.49	6	9 - 14	10.87	204.058	2.187	2.187
28	9005.583	9011.25	554126.50	1.54	0.47	5	9 - 13	10.84	446.005	2.187	2.187
29	8857.525	8863.12	512527.45	1.42	0.44	5	9 - 13	10.83	297.948	2.187	2.187
30	9115.663	9121.39	511346.81	1.42	0.43	4	8 - 11	9.64	556.085	2.187	2.187
31	8541.564	8547.02	494662.17	1.37	0.42	4	8 - 13	11.49	-18.014	2.187	2.187
32	8741.662	8747.21	452860.86	1.26	0.38	4	9 - 12	10.61	182.085	2.187	2.187
33	9035.587	9041.27	449978.46	1.25	0.38	5	9 - 13	10.83	476.010	2.187	2.187
34	8882.547	8888.16	432348.89	1.20	0.37	5	9 - 13	10.88	322.969	2.187	2.187
35	8813.670	8819.25	414231.34	1.15	0.35	5	9 - 13	10.77	254.093	2.187	2.187
36	8754.634	8760.19	346610.80	0.96	0.29	4	9 - 13	11.08	195.057	2.187	2.187
37	9082.616	9088.33	310717.72	0.86	0.26	5	10 -14	11.41	523.039	2.187	2.187
38	8675.620	8681.14	297587.29	0.83	0.25	5	8 - 13	10.95	116.043	2.187	2.187
39	8804.513	8810.09	296041.31	0.82	0.25	4	9 - 12	10.57	244.936	2.187	2.187

40	8757.650	8763.20	284539.88	0.79	0.24	3	10 -12	10.81	198.073	2.187	2.187
41	8993.584	8999.24	239203.87	0.66	0.20	4	9 - 12	10.46	434.006	2.187	2.187
42	8949.572	8955.21	223721.76	0.62	0.19	4	9 - 13	11.37	389.994	2.187	2.187
43	8589.584	8595.06	218493.57	0.61	0.19	5	9 - 13	11.03	30.006	2.187	2.187
44	9145.474	9151.22	211547.47	0.59	0.18	5	9 - 13	10.92	585.897	2.187	2.187
45	8781.663	8787.23	209803.31	0.58	0.18	4	9 - 12	10.53	222.086	2.187	2.187
46	8861.705	8867.30	186921.88	0.52	0.16	4	10 -13	11.30	302.127	2.187	2.187
47	8779.507	8785.07	179320.40	0.50	0.15	4	10 -13	11.38	219.930	2.187	2.187
48	8872.528	8878.13	146988.02	0.41	0.12	4	10 -13	11.24	312.951	2.187	2.187
49	8842.660	8848.25	125786.93	0.35	0.11	3	10 - 12	11.04	283.083	2.187	2.187
50	8941.534	8947.17	116570.57	0.32	0.10	3	10 - 12	11.08	381.957	2.187	2.187
51	9041.595	9047.28	94536.53	0.26	0.08	3	10 - 13	11.48	482.018	2.187	2.187

Procedure B in denaturing Tris buffer

Xtract Masses Table											
Row Number	Monoisotopic Mass	Average Mass	Sum Intensity	Relative Abundance	Fractional Abundance	Number of Charge States	Charge State Distribution	Average Charge	Delta Mass	Start Time (min)	Stop Time (min)
1	8900.547	8906.16	9356730.06	100.00	14.15	9	6 - 14	10.65	0.000	2.322	2.322
2	8559.575	8565.04	4743469.54	50.70	7.17	9	6 - 14	10.54	-340.972	2.322	2.322
3	8575.577	8581.05	3444962.51	36.82	5.21	8	7 - 14	10.52	-324.970	2.322	2.322
4	9071.622	9077.33	3076126.55	32.88	4.65	8	7 - 14	10.69	171.075	2.322	2.322
5	8985.575	8991.23	3056571.41	32.67	4.62	8	7 - 14	10.65	85.028	2.322	2.322
6	8968.585	8974.23	2484633.05	26.55	3.76	8	7 - 14	10.68	68.038	2.322	2.322
7	8916.556	8922.18	2455510.62	26.24	3.71	8	7 - 14	10.61	16.009	2.322	2.322
8	8644.606	8650.11	1865335.56	19.94	2.82	8	7 - 14	10.45	-255.941	2.322	2.322
9	8730.646	8736.19	1816584.82	19.41	2.75	7	7 - 13	10.51	-169.902	2.322	2.322
10	9053.618	9059.31	1751555.62	18.72	2.65	8	7 - 14	10.67	153.071	2.322	2.322
11	8747.654	8753.20	1391164.39	14.87	2.10	8	7 - 14	10.55	-152.894	2.322	2.322
12	8627.600	8633.09	1286408.04	13.75	1.95	8	7 - 14	10.38	-272.948	2.322	2.322
13	9130.491	9136.23	1220328.52	13.04	1.85	8	7 - 14	10.39	229.944	2.322	2.322
14	9036.610	9042.29	1219498.50	13.03	1.84	7	8 - 14	10.72	136.062	2.322	2.322
15	9088.622	9094.34	1126408.59	12.04	1.70	7	8 - 14	10.64	188.075	2.322	2.322
16	9025.610	9031.29	1100485.13	11.76	1.66	8	7 - 14	10.64	125.063	2.322	2.322
17	8661.613	8667.12	1029460.42	11.00	1.56	7	8 - 14	10.57	-238.934	2.322	2.322
18	9139.654	9145.40	994816.35	10.63	1.50	7	8 - 14	10.63	239.107	2.322	2.322
19	8712.643	8718.18	991249.17	10.59	1.50	7	8 - 14	10.56	-187.905	2.322	2.322
20	9157.655	9163.41	967463.72	10.34	1.46	7	8 - 14	10.83	257.108	2.322	2.322
21	9002.600	9008.26	854707.59	9.13	1.29	7	8 - 14	10.60	102.053	2.322	2.322
22	8688.646	8694.17	839633.66	8.97	1.27	6	8 - 13	10.56	-211.901	2.322	2.322
23	8816.684	8822.26	687453.20	7.35	1.04	7	8 - 14	10.54	-83.864	2.322	2.322
24	8696.625	8702.15	640826.91	6.85	0.97	6	8 - 13	10.43	-203.922	2.322	2.322
25	8789.529	8795.10	638266.20	6.82	0.97	6	8 - 13	10.31	-111.019	2.322	2.322
26	9121.655	9127.39	621881.10	6.65	0.94	7	8 - 14	10.62	221.107	2.322	2.322
27	8798.677	8804.25	562645.60	6.01	0.85	6	8 - 13	10.55	-101.870	2.322	2.322
28	9173.662	9179.43	526843.00	5.63	0.80	7	8 - 14	10.74	273.114	2.322	2.322
29	9243.698	9249.51	490408.64	5.24	0.74	7	8 - 14	10.75	343.151	2.322	2.322

30	9008.595	9014.26	452046.53	4.83	0.68	6	8 - 13	10.39	108.047	2.322	2.322
31	9225.694	9231.49	447545.26	4.78	0.68	6	8 - 13	10.74	325.147	2.322	2.322
32	8833.696	8839.28	439033.72	4.69	0.66	7	8 - 14	10.75	-66.852	2.322	2.322
33	8682.633	8688.15	438682.27	4.69	0.66	6	8 - 13	10.51	-217.914	2.322	2.322
34	9145.494	9151.24	418980.44	4.48	0.63	6	8 - 13	10.47	244.946	2.322	2.322
35	8764.658	8770.21	388949.04	4.16	0.59	7	8 - 14	10.69	-135.890	2.322	2.322
36	9103.632	9109.36	385019.51	4.11	0.58	6	8 - 13	10.71	203.085	2.322	2.322
37	9301.565	9307.41	384942.32	4.11	0.58	6	8 - 13	10.44	401.017	2.322	2.322
38	9215.529	9221.32	375622.14	4.01	0.57	6	8 - 13	10.45	314.981	2.322	2.322
39	9111.639	9117.37	367429.88	3.93	0.56	5	9 - 13	10.77	211.091	2.322	2.322
40	9207.679	9213.47	364037.52	3.89	0.55	6	9 - 14	10.96	307.132	2.322	2.322
41	9095.631	9101.35	356177.44	3.81	0.54	5	9 - 13	10.77	195.083	2.322	2.322
42	8941.562	8947.20	325350.89	3.48	0.49	6	8 - 13	10.50	41.014	2.322	2.322
43	8781.665	8787.23	318231.96	3.40	0.48	5	9 - 13	10.87	-118.882	2.322	2.322
44	9196.680	9202.46	312802.42	3.34	0.47	6	8 - 13	10.61	296.133	2.322	2.322
45	8774.685	8780.25	270474.91	2.89	0.41	5	9 - 13	10.81	-125.862	2.322	2.322
46	8856.557	8862.15	257857.98	2.76	0.39	6	9 - 14	10.97	-43.990	2.322	2.322
47	9179.667	9185.44	252812.59	2.70	0.38	5	9 - 13	11.00	279.119	2.322	2.322
48	9189.676	9195.45	246950.06	2.64	0.37	5	9 - 13	10.97	289.129	2.322	2.322
49	8702.630	8708.16	239065.81	2.56	0.36	5	9 - 13	10.77	-197.917	2.322	2.322
50	9261.702	9267.52	237420.35	2.54	0.36	6	9 - 14	11.22	361.155	2.322	2.322
51	9198.521	9204.30	233248.92	2.49	0.35	6	8 - 13	10.45	297.973	2.322	2.322
52	8884.713	8890.32	232310.77	2.48	0.35	5	9 - 13	10.90	-15.835	2.322	2.322
53	8862.695	8868.29	230987.25	2.47	0.35	5	9 - 13	10.97	-37.853	2.322	2.322
54	9047.621	9053.31	230301.28	2.46	0.35	5	10 -14	11.36	147.074	2.322	2.322
55	8919.724	8925.35	230025.59	2.46	0.35	7	8 - 14	10.72	19.176	2.322	2.322
56	8960.585	8966.23	223609.68	2.39	0.34	4	9 - 12	10.39	60.038	2.322	2.322
57	8755.655	8761.21	214138.28	2.29	0.32	6	9 - 14	10.86	-144.892	2.322	2.322
58	8873.546	8879.15	210467.29	2.25	0.32	6	8 - 13	10.40	-27.001	2.322	2.322
59	8994.598	9000.26	207695.89	2.22	0.31	5	9 - 13	10.76	94.051	2.322	2.322
60	8668.623	8674.14	207340.58	2.22	0.31	5	9 - 13	10.50	-231.925	2.322	2.322
61	8855.711	8861.31	206150.95	2.20	0.31	6	9 - 14	10.88	-44.836	2.322	2.322
62	8882.548	8888.16	205834.90	2.20	0.31	4	8 - 11	9.72	-17.999	2.322	2.322
63	8846.712	8852.30	200664.25	2.14	0.30	5	8 - 12	10.39	-53.836	2.322	2.322
64	9283.554	9289.39	200463.05	2.14	0.30	6	8 - 13	10.49	383.006	2.322	2.322
65	9081.630	9087.34	187430.20	2.00	0.28	4	10 -13	11.22	181.083	2.322	2.322
66	8902.724	8908.34	178829.14	1.91	0.27	5	10 -14	11.33	2.177	2.322	2.322
67	8871.713	8877.32	165032.04	1.76	0.25	5	9 - 13	10.87	-28.834	2.322	2.322
68	9165.664	9171.43	164769.83	1.76	0.25	5	9 - 13	10.88	265.117	2.322	2.322
69	9148.648	9154.40	164459.30	1.76	0.25	4	9 - 12	10.46	248.100	2.322	2.322
70	8591.578	8597.06	161459.25	1.73	0.24	5	9 - 13	10.80	-308.969	2.322	2.322
71	9317.564	9323.42	156053.47	1.67	0.24	5	9 - 13	10.66	417.016	2.322	2.322
72	8804.520	8810.09	152159.96	1.63	0.23	4	9 - 12	10.53	-96.028	2.322	2.322
73	8826.698	8832.28	148575.98	1.59	0.22	5	9 - 13	10.85	-73.849	2.322	2.322
74	8951.590	8957.23	148047.49	1.58	0.22	5	9 - 14	11.22	51.043	2.322	2.322
75	9240.542	9246.35	146186.20	1.56	0.22	6	9 - 14	10.78	339.994	2.322	2.322
76	9131.656	9137.40	140006.42	1.50	0.21	4	9 - 13	11.04	231.108	2.322	2.322

77	8811.680	8817.26	123917.99	1.32	0.19	5	9 - 13	10.90	-88.868	2.322	2.322
78	9062.616	9068.32	113819.19	1.22	0.17	3	9 - 11	10.06	162.069	2.322	2.322
79	9312.714	9318.56	108878.32	1.16	0.16	4	10 -13	11.37	412.167	2.322	2.322
80	9255.540	9261.36	106306.29	1.14	0.16	5	9 - 13	10.78	354.993	2.322	2.322
81	9255.706	9261.52	103665.66	1.11	0.16	4	10 -13	11.28	355.159	2.322	2.322
82	9266.547	9272.37	101835.84	1.09	0.15	5	9 - 13	10.78	366.000	2.322	2.322
83	8788.663	8794.23	101719.25	1.09	0.15	3	10 -12	11.09	-111.885	2.322	2.322
84	9387.602	9393.49	101043.39	1.08	0.15	5	9 - 13	10.72	487.055	2.322	2.322
85	9272.716	9278.54	99730.01	1.07	0.15	4	10 -13	11.28	372.168	2.322	2.322
86	9231.533	9237.33	94951.14	1.01	0.14	5	9 - 13	10.62	330.986	2.322	2.322
87	9236.707	9242.51	94059.64	1.01	0.14	4	10 -13	11.43	336.160	2.322	2.322
88	8977.577	8983.23	93916.51	1.00	0.14	3	9 - 12	10.54	77.029	2.322	2.322
89	8935.728	8941.36	90322.76	0.97	0.14	5	9 - 13	11.07	35.180	2.322	2.322
90	8542.570	8548.03	89824.28	0.96	0.14	5	9 - 13	10.92	-357.977	2.322	2.322
91	8675.621	8681.14	89774.77	0.96	0.14	3	10 -12	10.98	-224.926	2.322	2.322
92	8610.600	8616.09	83546.36	0.89	0.13	3	9 - 13	10.43	-289.947	2.322	2.322
93	9369.593	9375.48	82316.58	0.88	0.12	4	9 - 12	10.48	469.046	2.322	2.322
94	8510.563	8516.01	78441.84	0.84	0.12	5	10 -14	11.91	-389.984	2.322	2.322
95	8925.566	8931.19	76553.42	0.82	0.12	3	10 -12	10.98	25.018	2.322	2.322
96	8839.694	8845.28	72957.40	0.78	0.11	3	9 - 13	10.55	-60.853	2.322	2.322
97	8598.600	8604.08	71385.54	0.76	0.11	4	9 - 13	11.06	-301.948	2.322	2.322
98	8934.558	8940.19	67432.70	0.72	0.10	3	9 - 12	10.86	34.011	2.322	2.322
99	8722.652	8728.19	65874.95	0.70	0.10	3	10 -13	10.91	-177.895	2.322	2.322
100	8759.649	8765.20	60100.11	0.64	0.09	3	11 -13	11.74	-140.898	2.322	2.322
101	9331.726	9337.59	56545.94	0.60	0.09	3	10 -13	10.95	431.179	2.322	2.322
102	8949.759	8955.40	54458.36	0.58	0.08	4	11 -14	11.90	49.212	2.322	2.322
103	9285.722	9291.56	52487.97	0.56	0.08	3	9 - 12	11.00	385.175	2.322	2.322
104	9403.605	9409.51	45555.49	0.49	0.07	4	9 - 12	10.61	503.058	2.322	2.322
105	9309.560	9315.41	42559.71	0.45	0.06	4	9 - 12	10.54	409.012	2.322	2.322
106	8925.728	8931.36	41631.04	0.44	0.06	3	9 - 13	10.41	25.181	2.322	2.322
107	8943.732	8949.37	39022.43	0.42	0.06	4	10 -14	11.54	43.185	2.322	2.322
108	9005.757	9011.42	38427.83	0.41	0.06	3	10 -13	11.56	105.209	2.322	2.322
109	9360.427	9366.30	37913.19	0.41	0.06	4	9 - 12	10.36	459.880	2.322	2.322
110	9348.585	9354.46	36681.13	0.39	0.06	4	10 -13	11.14	448.038	2.322	2.322
111	8929.578	8935.21	30339.99	0.32	0.05	3	9 - 14	11.08	29.030	2.322	2.322

Myoglobin, Deconvoluted mass spectrum:

Procedure A in denaturing Tris buffer

Row Number	Monoisotopic Mass	Average Mass	Sum Intensity	Relative Abundance	Fractional Abundance	Number of Charge States	Charge State Distribution	Average Charge	Delta Mass	Start Time (min)	Stop Time (min)
1	17281.987	17292.84	52622.71	100.00	18.12	16	10 - 25	18.94	0.000	2.701	2.701
2	16941.004	16951.69	35108.17	66.72	12.09	12	14 - 25	19.94	-340.983	2.701	2.701
3	17407.034	17417.95	27354.56	51.98	9.42	12	14 - 25	19.59	125.047	2.701	2.701
4	17067.034	17077.79	17193.46	32.67	5.92	9	17 - 25	20.75	-214.953	2.701	2.701
5	17475.066	17486.01	9246.79	17.57	3.18	8	18 - 25	21.14	193.079	2.701	2.701
6	17433.053	17443.98	8924.31	16.96	3.07	6	19 - 24	21.28	151.066	2.701	2.701
7	17533.063	17544.04	8920.59	16.95	3.07	6	18 - 23	20.39	251.076	2.701	2.701
8	17453.065	17464.00	7975.67	15.16	2.75	6	20 - 25	22.03	171.078	2.701	2.701
9	17416.032	17426.95	6829.67	12.98	2.35	6	19 - 25	21.96	134.045	2.701	2.701
10	17567.099	17578.10	6725.62	12.78	2.32	6	18 - 24	20.40	285.112	2.701	2.701
11	17367.009	17377.91	6610.76	12.56	2.28	6	19 - 25	22.22	85.023	2.701	2.701
12	17160.065	17170.87	6289.47	11.95	2.17	7	19 - 25	21.67	-121.922	2.701	2.701
13	17498.069	17509.03	6148.81	11.68	2.12	5	19 - 24	21.19	216.083	2.701	2.701
14	17115.067	17125.85	5742.11	10.91	1.98	5	19 - 24	21.35	-166.920	2.701	2.701
15	17193.125	17203.94	5696.28	10.82	1.96	5	19 - 23	20.70	-88.862	2.701	2.701
16	17089.069	17099.84	5661.30	10.76	1.95	6	20 - 25	22.26	-192.918	2.701	2.701
17	17553.058	17564.05	5253.20	9.98	1.81	4	20 - 23	21.23	271.071	2.701	2.701
18	17487.042	17497.99	5025.67	9.55	1.73	4	20 - 24	22.12	205.055	2.701	2.701
19	17389.021	17399.93	4904.72	9.32	1.69	5	20 - 25	22.03	107.034	2.701	2.701
20	17007.093	17017.82	4679.44	8.89	1.61	5	21 - 25	22.80	-274.893	2.701	2.701
21	17541.072	17552.05	4232.87	8.04	1.46	5	20 - 25	21.96	259.086	2.701	2.701
22	17216.124	17226.95	3781.44	7.19	1.30	4	17 - 22	19.50	-65.863	2.701	2.701
23	17021.031	17031.77	3621.04	6.88	1.25	5	19 - 25	21.64	-260.956	2.701	2.701
24	17134.111	17144.90	3560.82	6.77	1.23	4	19 - 24	21.49	-147.876	2.701	2.701
25	17348.057	17358.94	3320.15	6.31	1.14	3	22 - 25	23.13	66.070	2.701	2.701
26	17183.089	17193.90	3249.58	6.18	1.12	4	20 - 24	21.93	-98.898	2.701	2.701
27	17424.026	17434.95	3218.03	6.12	1.11	3	20 - 23	21.18	142.039	2.701	2.701
28	17601.057	17612.07	3190.33	6.06	1.10	4	19 - 25	22.25	319.070	2.701	2.701
29	17078.040	17088.80	3096.58	5.88	1.07	4	22 - 25	23.36	-203.946	2.701	2.701
30	17146.094	17156.89	3010.20	5.72	1.04	3	21 - 24	22.24	-135.893	2.701	2.701
31	17226.066	17236.90	2958.52	5.62	1.02	4	19 - 25	21.53	-55.921	2.701	2.701
32	17330.978	17341.86	2801.88	5.32	0.96	3	22 - 24	22.83	48.991	2.701	2.701
33	17034.005	17044.75	2568.79	4.88	0.88	3	20 - 24	22.16	-247.981	2.701	2.701
34	17665.071	17676.12	2347.30	4.46	0.81	4	20 - 24	22.22	383.085	2.701	2.701
35	17171.048	17181.85	2261.80	4.30	0.78	3	20 - 24	21.19	-110.939	2.701	2.701
36	17052.085	17062.84	2215.04	4.21	0.76	3	22 - 25	23.28	-229.902	2.701	2.701
37	16990.040	17000.76	2167.37	4.12	0.75	3	20 - 22	20.95	-291.946	2.701	2.701
38	17269.137	17279.99	1906.34	3.62	0.66	3	21 - 24	22.47	-12.850	2.701	2.701

Procedure B in denaturing Tris buffer

Xtract Masses Table											
Row Number	Monoisotopic Mass	Average Mass	Sum Intensity	Relative Abundance	Fractional Abundance	Number of Charge States	Charge State Distribution	Average Charge	Delta Mass	Start Time (min)	Stop Time (min)
1	16941.032	16951.72	5550.80	100.00	14.70	6	20 - 25	22.36	0.000	2.876	2.876
2	17454.021	17464.96	4834.26	87.09	12.81	5	10 - 24	15.46	512.988	2.876	2.876
3	17899.009	17910.20	4158.81	74.92	11.02	3	10 - 22	12.07	957.977	2.876	2.876
4	17348.992	17359.88	3412.45	61.48	9.04	6	19 - 25	21.53	407.960	2.876	2.876
5	17280.960	17291.82	3334.90	60.08	8.83	3	19 - 21	20.03	339.928	2.876	2.876
6	17749.061	17760.16	3092.49	55.71	8.19	4	10 - 25	17.62	808.029	2.876	2.876
7	17281.967	17292.82	2841.04	51.18	7.53	3	23 - 25	23.66	340.935	2.876	2.876
8	17524.078	17535.05	2121.17	38.21	5.62	4	19 - 25	21.51	583.046	2.876	2.876
9	17774.015	17785.13	2107.27	37.96	5.58	3	22 - 24	23.07	832.983	2.876	2.876
10	17437.058	17447.99	2027.08	36.52	5.37	4	22 - 25	23.35	496.026	2.876	2.876
11	17559.043	17570.03	1805.40	32.53	4.78	3	19 - 23	21.09	618.011	2.876	2.876
12	17860.052	17871.22	1254.48	22.60	3.32	3	23 - 25	23.84	919.019	2.876	2.876
13	17933.034	17944.24	1211.05	21.82	3.21	3	22 - 25	23.33	992.001	2.876	2.876

Streptavidin, Deconvoluted spectrum:

Procedure A in Tris buffer

Row Number	Average Mass	Intensity	Relative Abundance	Fractional Abundance	Score	Number of Charge States	Charge State Distribution	Mass Std Dev	PPM Std Dev	Delta Mass	Start Time (min)	Stop Time (min)
1	13270.65	3035654.75	100.00	54.99	40.00	7	7 - 13	0.14	10.80	0.00	2.992	2.992
2	13611.96	2484628.50	81.85	45.01	35.58	6	8 - 13	0.22	16.38	341.31	2.992	2.992

Procedure B in Tris buffer

ReSpect Masses Table												
Row Number	Average Mass	Intensity	Relative Abundance	Fractional Abundance	Score	Number of Charge States	Charge State Distribution	Mass Std Dev	PPM Std Dev	Delta Mass	Start Time (min)	Stop Time (min)
1	13611.80	6167011.00	100.00	41.52	35.24	6	8 - 13	0.24	17.66	0.00	2.338	2.338
2	13270.64	5238021.50	84.94	35.27	40.24	7	7 - 13	0.22	16.70	-341.15	2.338	2.338
3	13952.92	3446388.00	55.88	23.21	35.29	6	8 - 13	0.24	16.96	341.13	2.338	2.338

Procedure A in denaturing Tris buffer

ReSpect Masses Table												
Row Number	Average Mass	Intensity	Relative Abundance	Fractional Abundance	Score	Number of Charge States	Charge State Distribution	Mass Std Dev	PPM Std Dev	Delta Mass	Start Time (min)	Stop Time (min)
1	13270.84	27795738.00	100.00	50.91	40.08	7	7 - 13	0.26	19.45	0.00	2.308	2.308
2	13611.97	11833453.00	42.57	21.67	39.80	7	7 - 13	0.22	16.49	341.13	2.308	2.308
3	13953.28	3488843.25	12.55	6.39	34.99	6	8 - 13	0.22	15.77	682.44	2.308	2.308
4	13338.85	3346659.75	12.04	6.13	34.12	6	8 - 13	0.29	21.85	68.01	2.308	2.308
5	13286.84	2046008.50	7.36	3.75	34.60	6	8 - 13	0.18	13.87	16.01	2.308	2.308
6	13356.59	1843207.88	6.63	3.38	34.90	6	8 - 13	0.22	16.66	85.76	2.308	2.308

7	13399.57	1340645.38	4.82	2.46	35.60	6	8 - 13	0.24	17.98	128.73	2.308	2.308
8	13679.88	1016020.63	3.66	1.86	36.23	6	8 - 13	0.15	10.91	409.05	2.308	2.308
9	13313.64	740265.31	2.66	1.36	35.02	6	8 - 13	0.40	29.72	42.80	2.308	2.308
10	13628.09	687151.75	2.47	1.26	34.82	6	8 - 13	0.33	24.16	357.25	2.308	2.308
11	13501.09	459253.00	1.65	0.84	36.30	6	8 - 13	0.25	18.32	230.26	2.308	2.308

Procedure B in denaturing Tris buffer

ReSpect Masses Table												
Row Number	Average Mass	Intensity	Relative Abundance	Fractional Abundance	Score	Number of Charge States	Charge State Distribution	Mass Std Dev	PPM Std Dev	Delta Mass	Start Time (min)	Stop Time (min)
1	13611.79	4620764.50	100.00	28.21	40.35	7	7 - 13	0.22	16.12	0.00	2.337	2.337
2	13270.70	3907269.50	84.56	23.85	40.57	7	7 - 13	0.23	17.41	-341.09	2.337	2.337
3	13952.98	2154346.50	46.62	13.15	39.29	7	7 - 13	0.21	14.77	341.19	2.337	2.337
4	14294.21	1706775.00	36.94	10.42	36.25	7	8 - 14	0.54	37.95	682.42	2.337	2.337
5	13679.77	634905.44	13.74	3.88	31.20	6	8 - 13	0.44	32.51	67.98	2.337	2.337
6	13697.67	531412.25	11.50	3.24	33.23	6	8 - 13	0.40	29.06	85.88	2.337	2.337
7	13627.84	507880.19	10.99	3.10	32.68	6	8 - 13	0.25	18.15	16.05	2.337	2.337
8	13338.58	360499.72	7.80	2.20	33.04	6	8 - 13	0.42	31.31	-273.20	2.337	2.337
9	13969.24	314324.69	6.80	1.92	30.76	6	8 - 13	0.41	29.28	357.45	2.337	2.337
10	13842.03	310928.25	6.73	1.90	27.69	6	8 - 13	0.40	29.16	230.24	2.337	2.337
11	14635.23	295285.59	6.39	1.80	36.87	7	8 - 14	0.42	28.47	1023.44	2.337	2.337
12	13356.35	235676.69	5.10	1.44	31.32	6	8 - 13	0.42	31.11	-255.44	2.337	2.337
13	14021.09	197106.42	4.27	1.20	38.33	7	8 - 14	0.55	39.35	409.30	2.337	2.337
14	14038.79	186927.53	4.05	1.14	37.03	7	8 - 14	0.58	41.26	427.00	2.337	2.337
15	14309.94	156825.34	3.39	0.96	37.96	7	8 - 14	0.51	35.97	698.15	2.337	2.337
16	13287.35	154672.28	3.35	0.94	32.48	6	8 - 13	0.43	32.53	-324.43	2.337	2.337
17	13500.59	106594.87	2.31	0.65	34.79	6	8 - 13	0.29	21.59	-111.20	2.337	2.337

16. NMR spectrum of the synthesized compounds.

EBX (2e)

^1H NMR (400 MHz, $CDCl_3$)

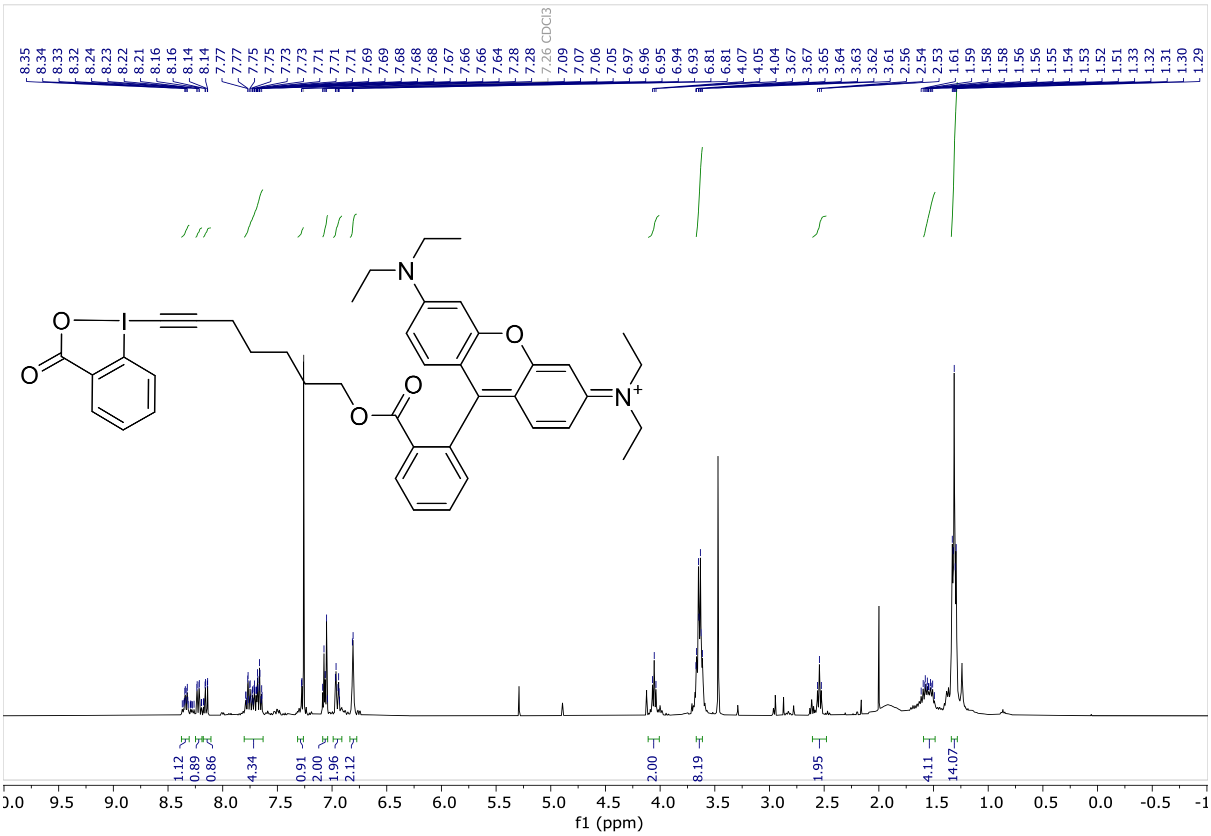

^{13}C NMR (101 MHz, DMSO)

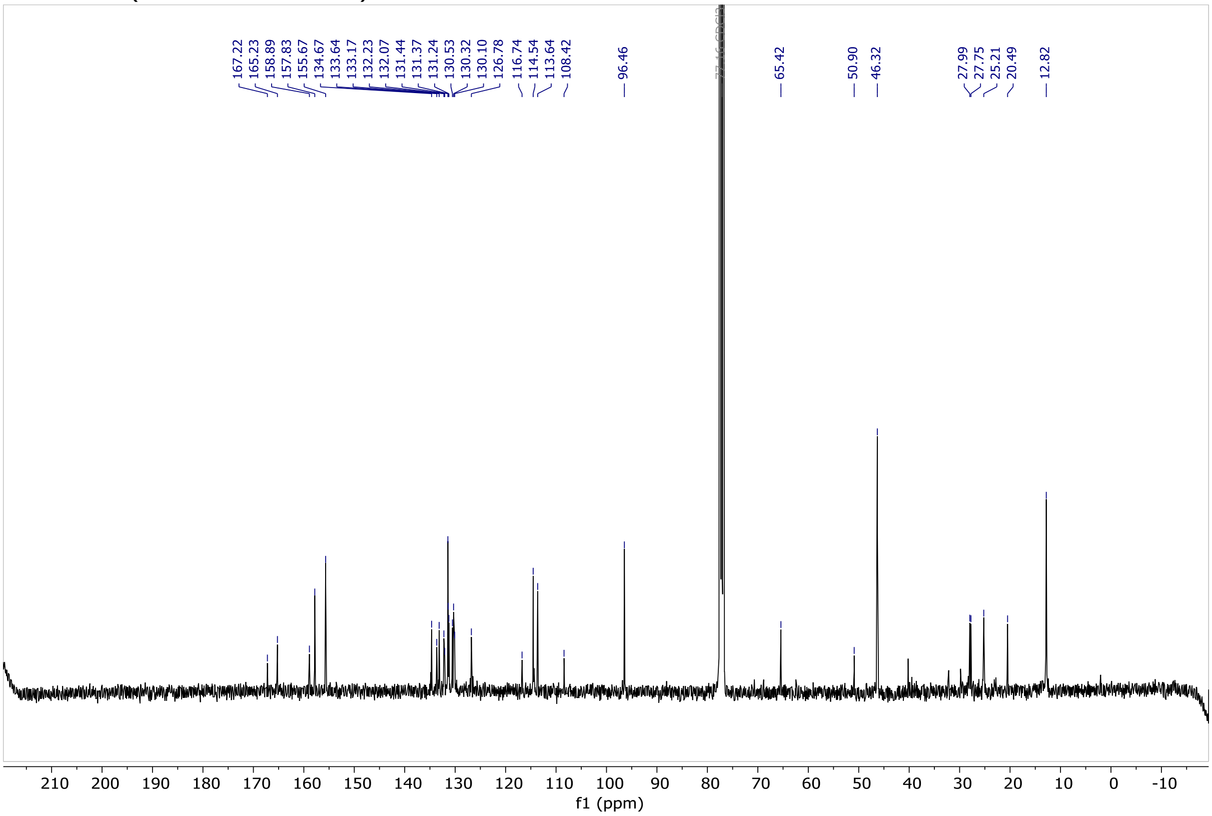

EBX (2j)

^{1}H NMR (400 MHz, DMSO)

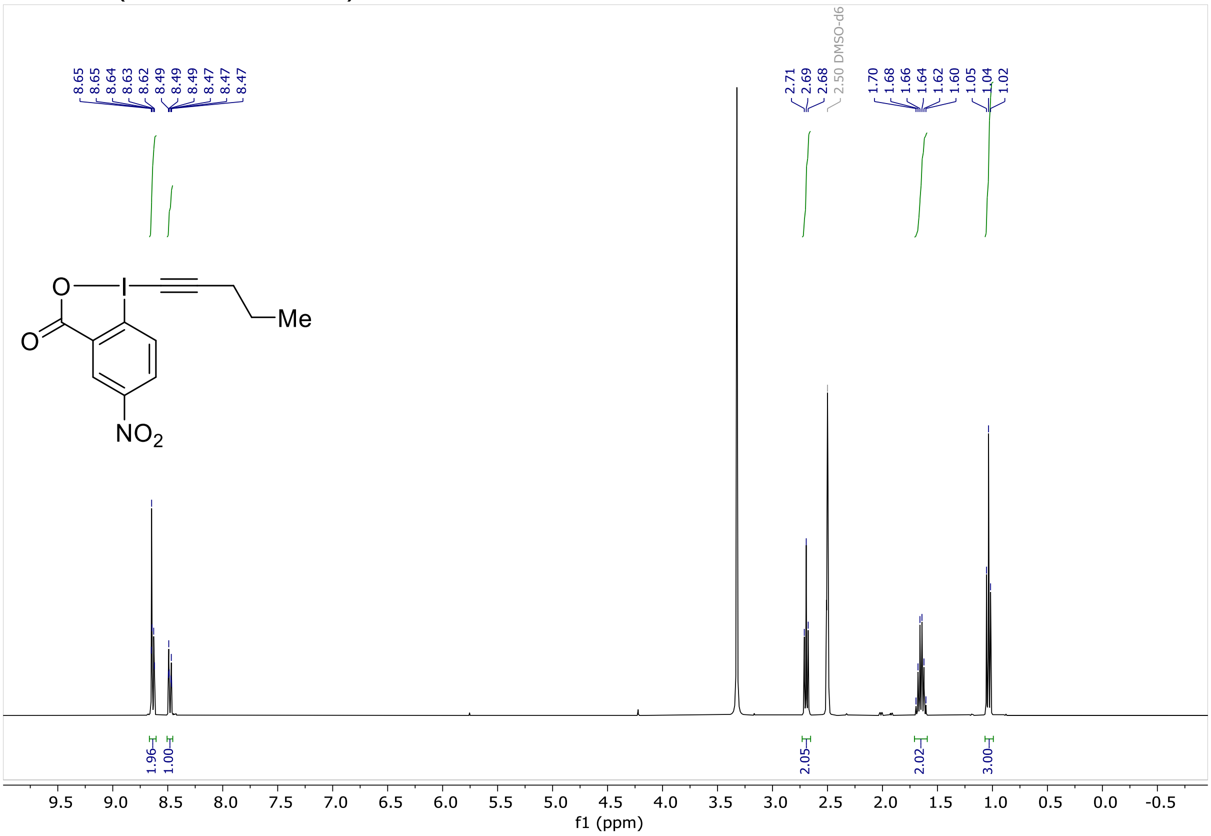

^{13}C NMR (101 MHz, DMSO)

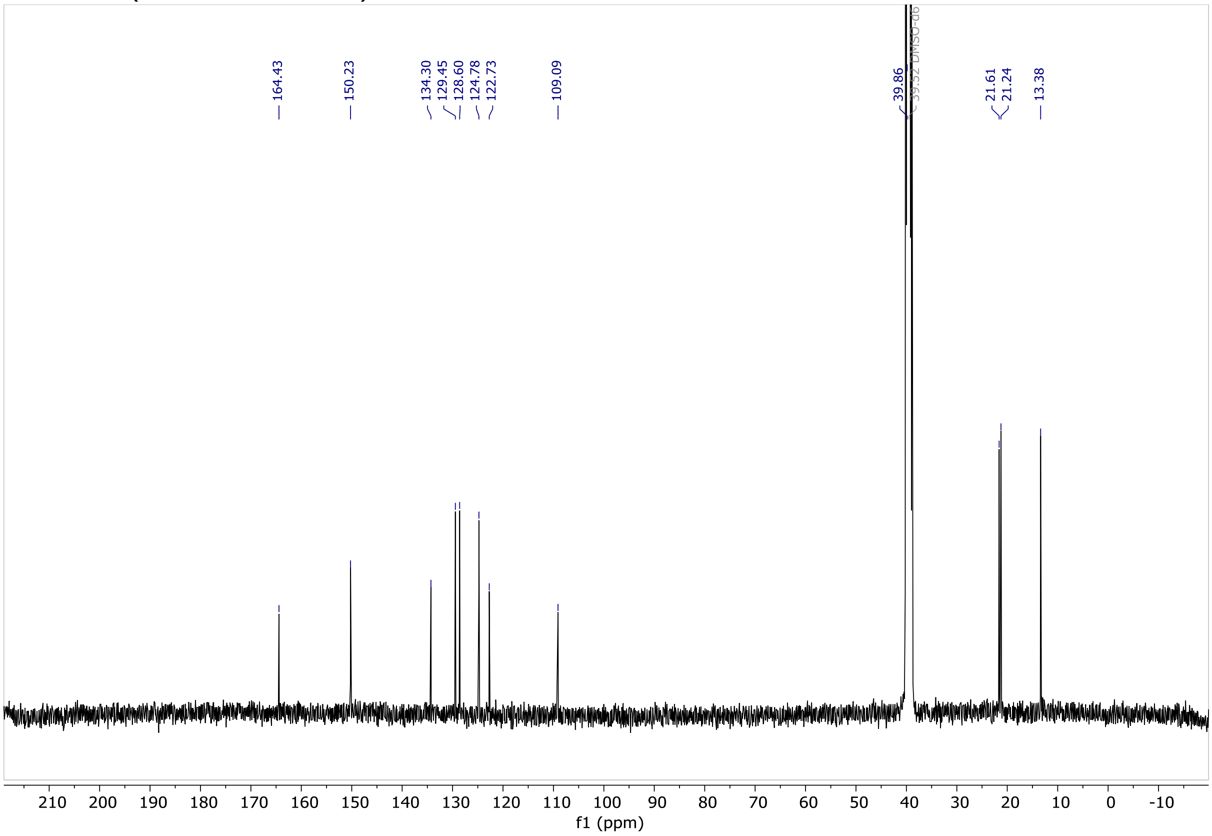

AFYA-NH$_2$ (1a)

^{1}H NMR (400 MHz, CD_3OD)

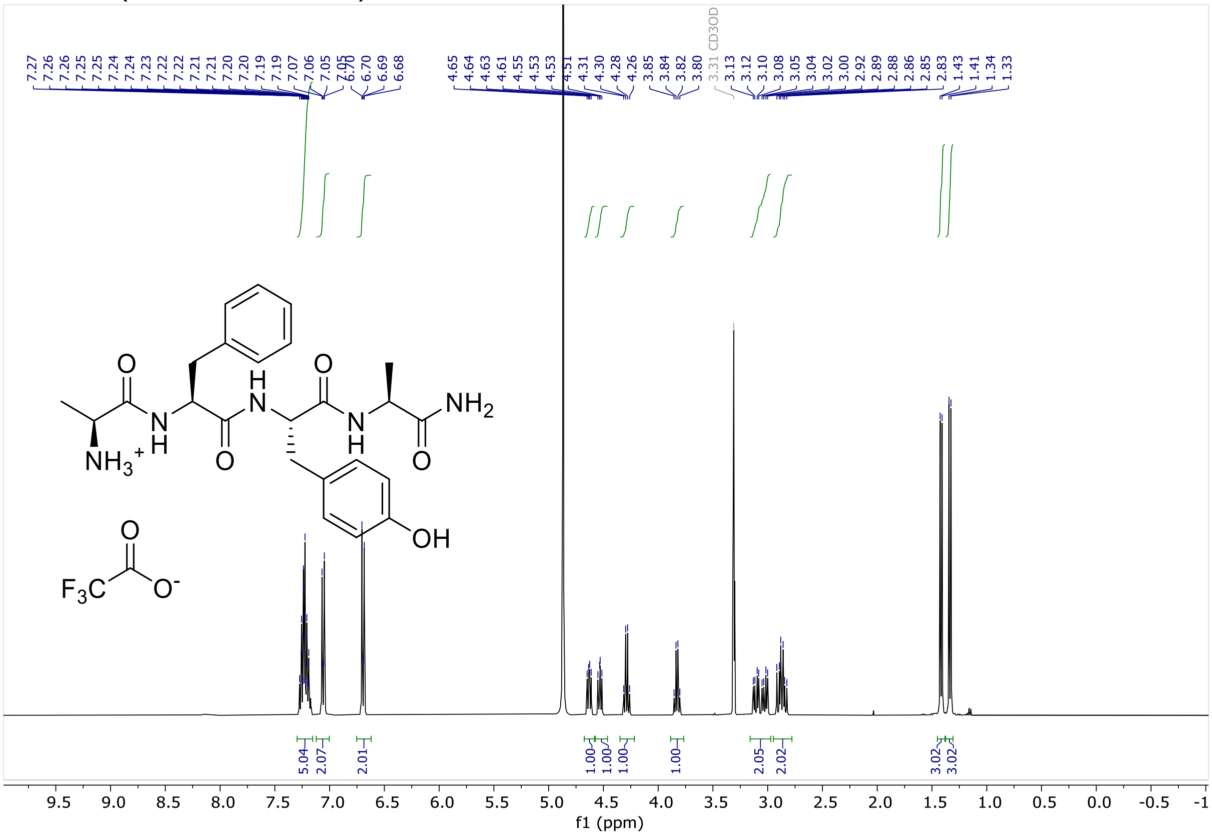

^{13}C NMR (101 MHz, CD_3OD)

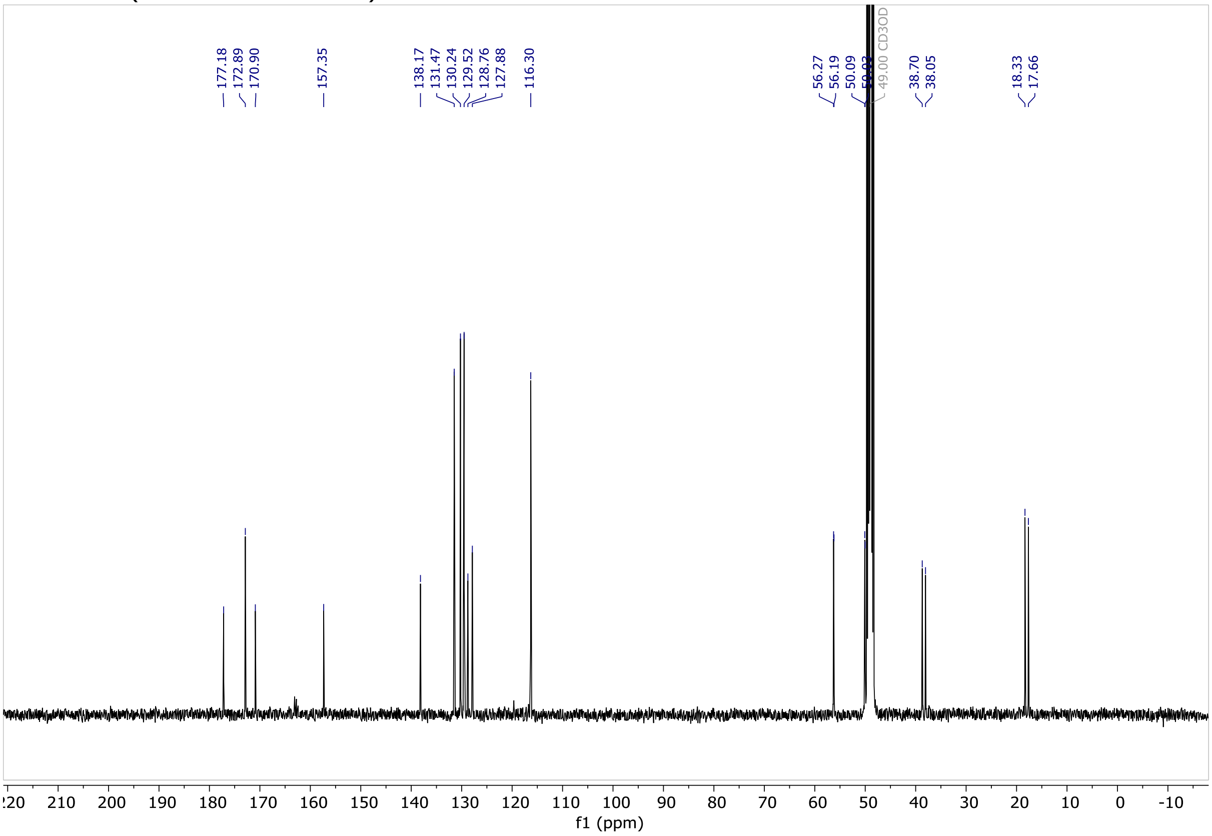

ALYA-NH$_2$ (1b)

^{1}H NMR (400 MHz, CD_3OD)

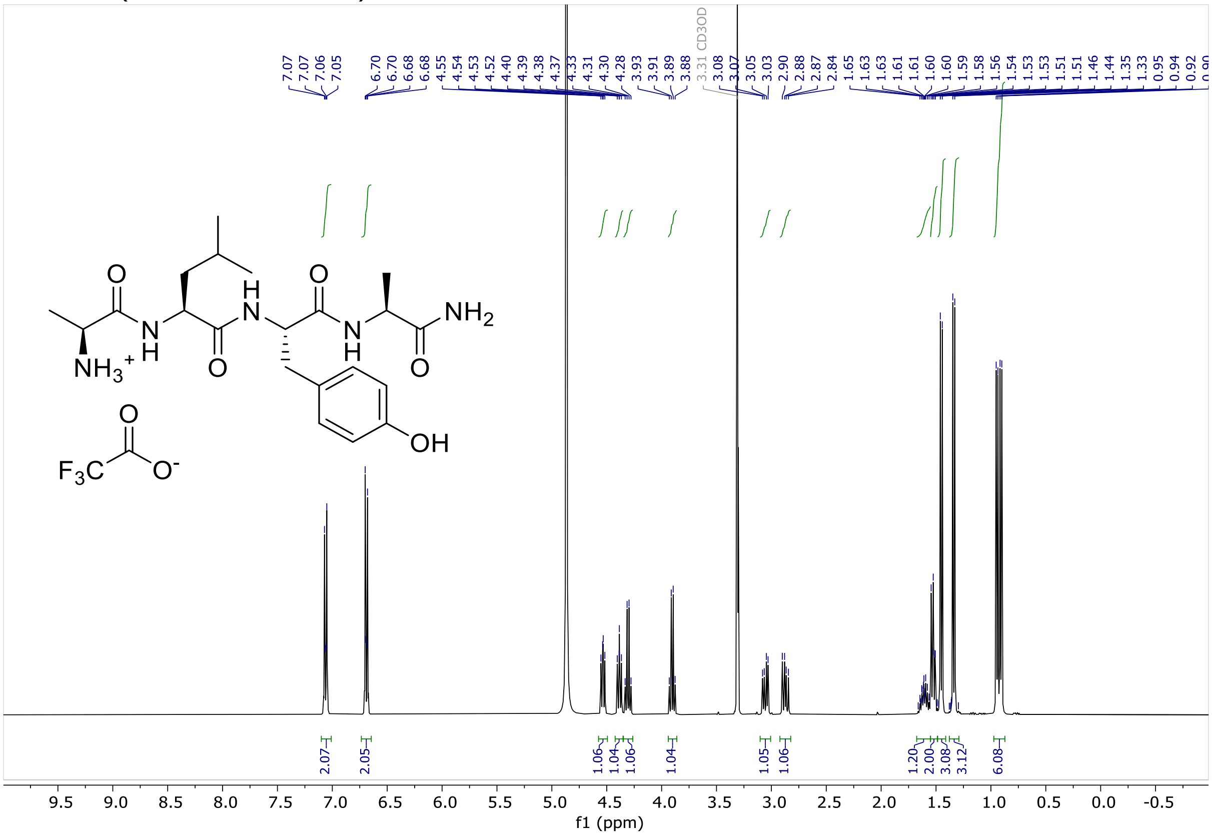

^{13}C NMR (101 MHz, CD_3OD)

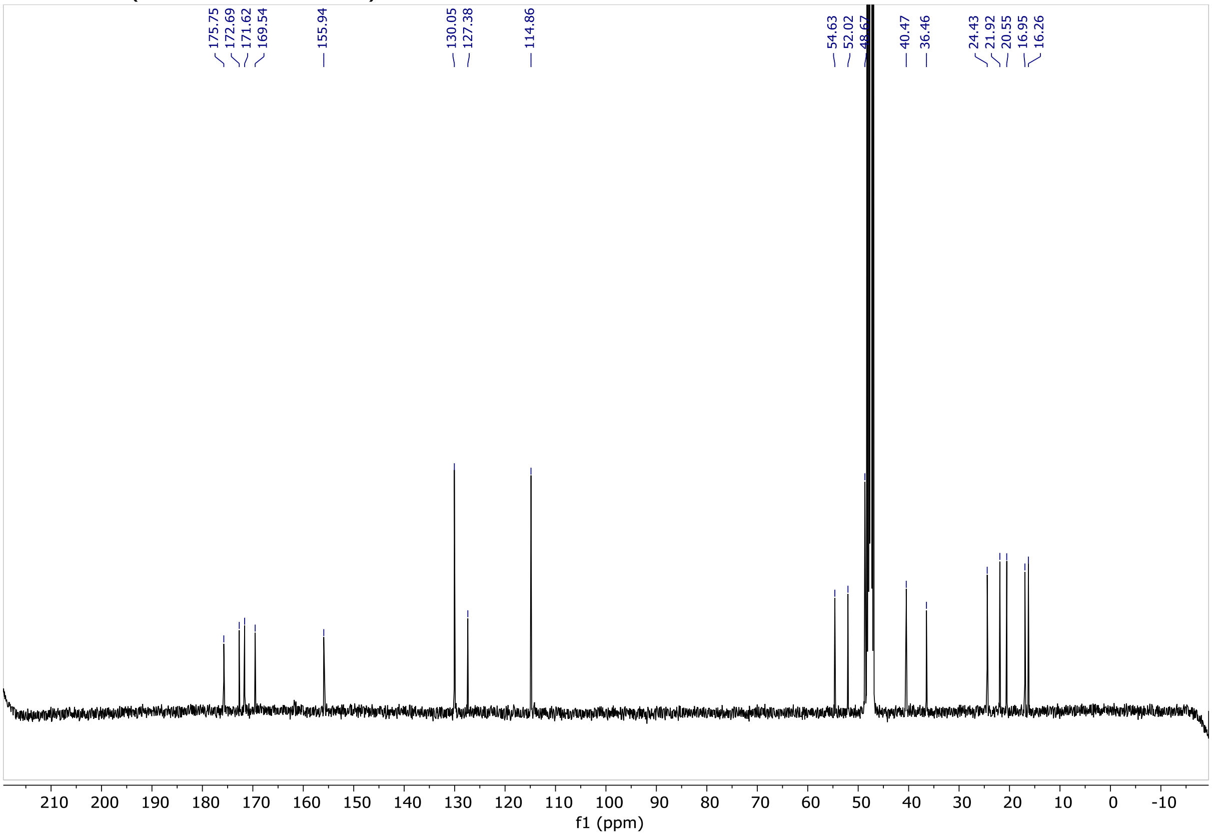

AWYA-NH$_2$ (1c)

^{1}H NMR (400 MHz, CD_3OD)

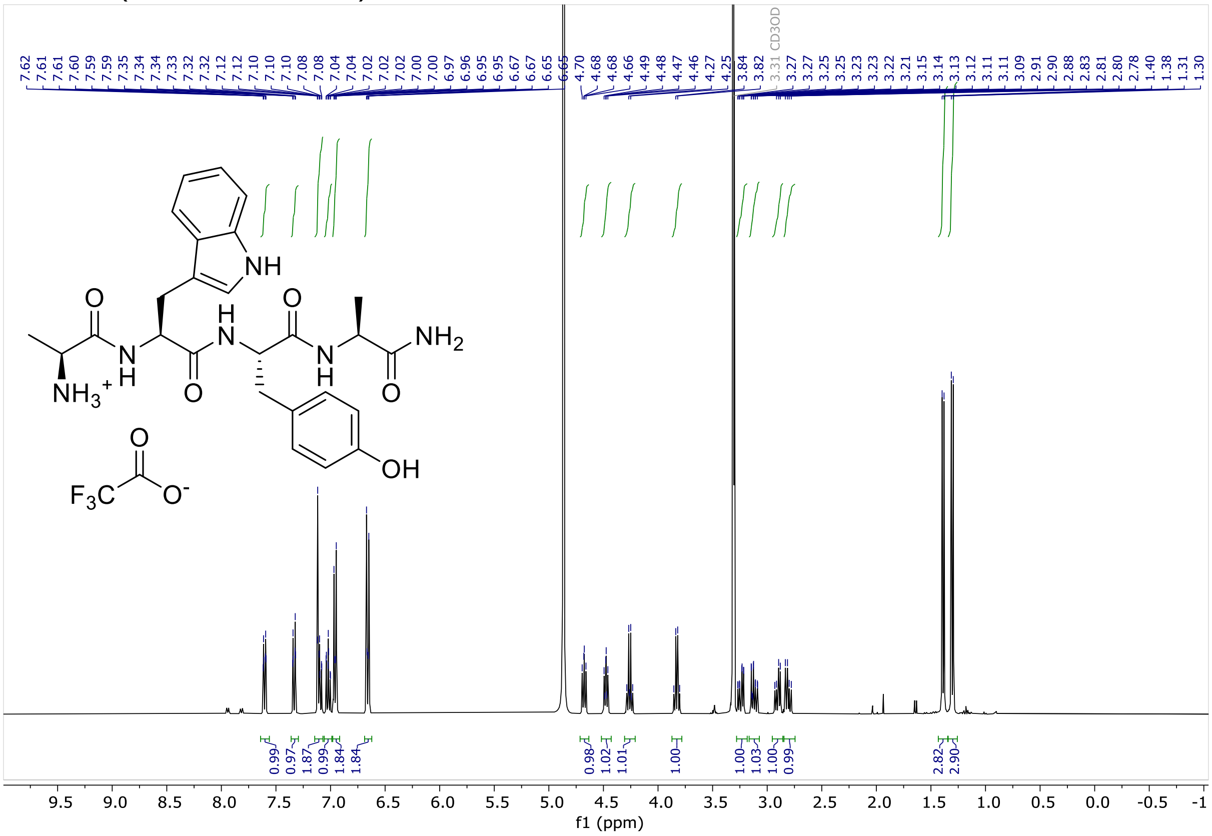

^{13}C NMR (101 MHz, CD_3OD)

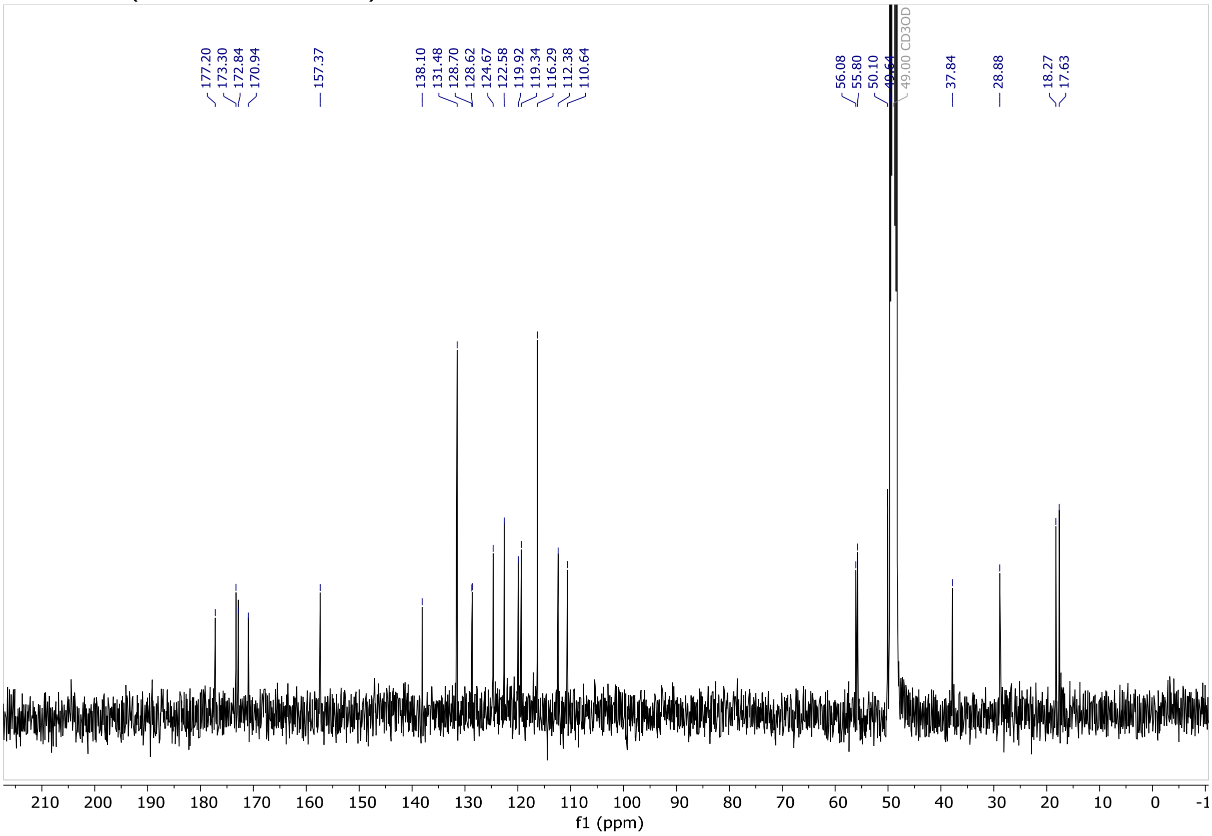

ARYA-NH$_2$ (1d)

^{1}H NMR (400 MHz, CD_3OD)

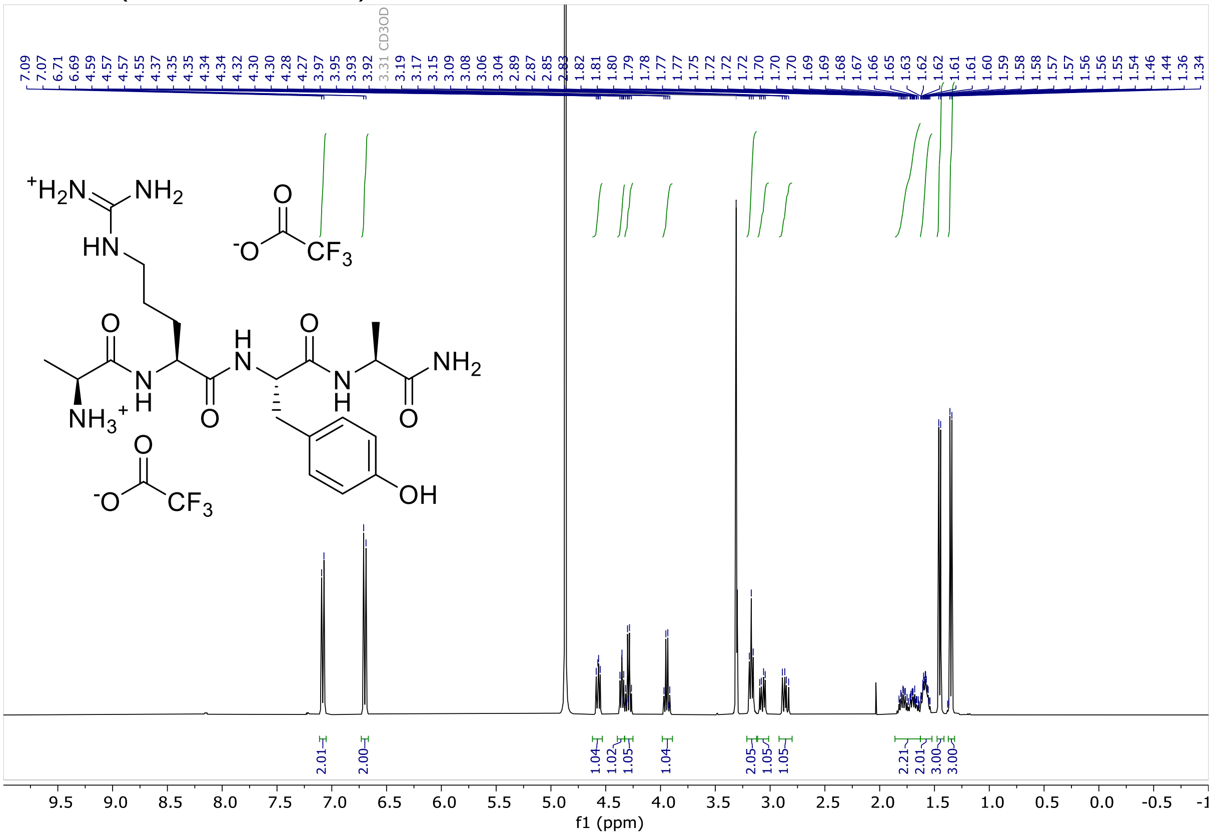

^{13}C NMR (101 MHz, CD_3OD)

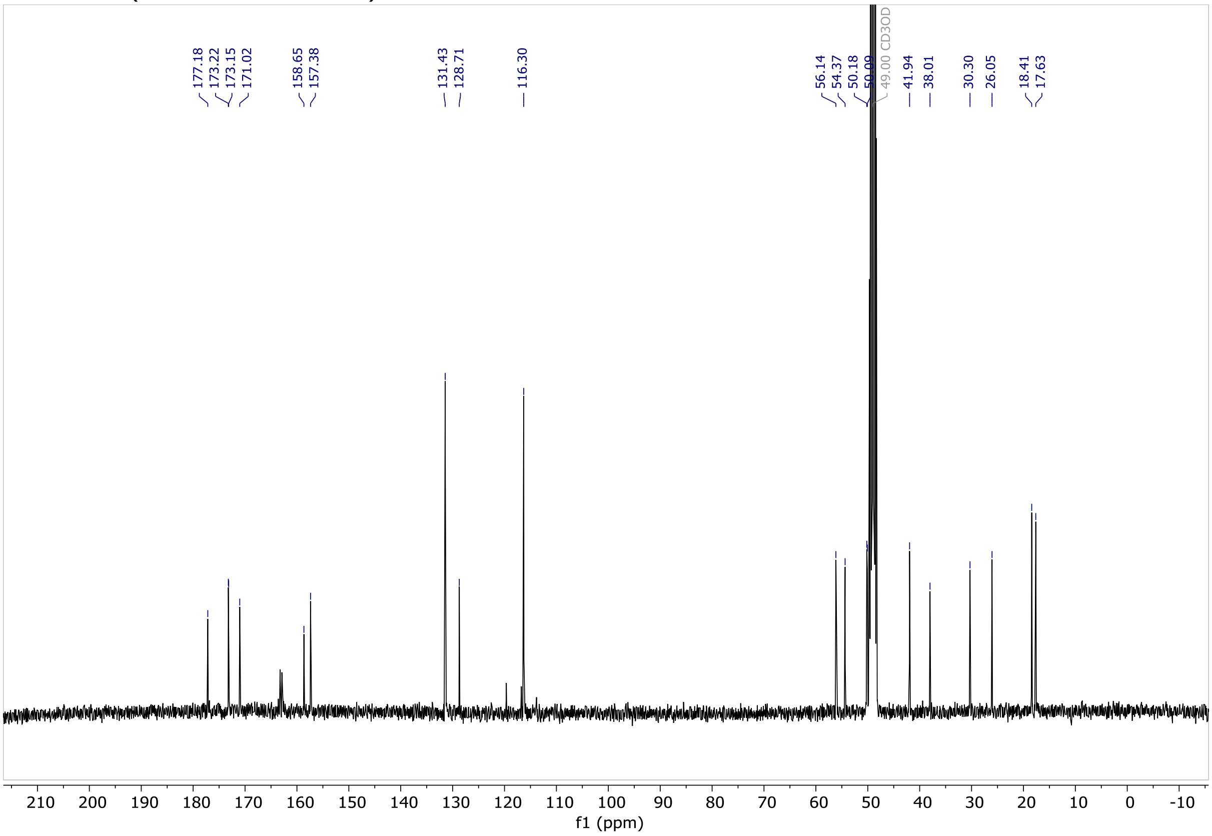

AHYA-NH$_2$ (1e)

^{1}H NMR (400 MHz, CD_3OD)

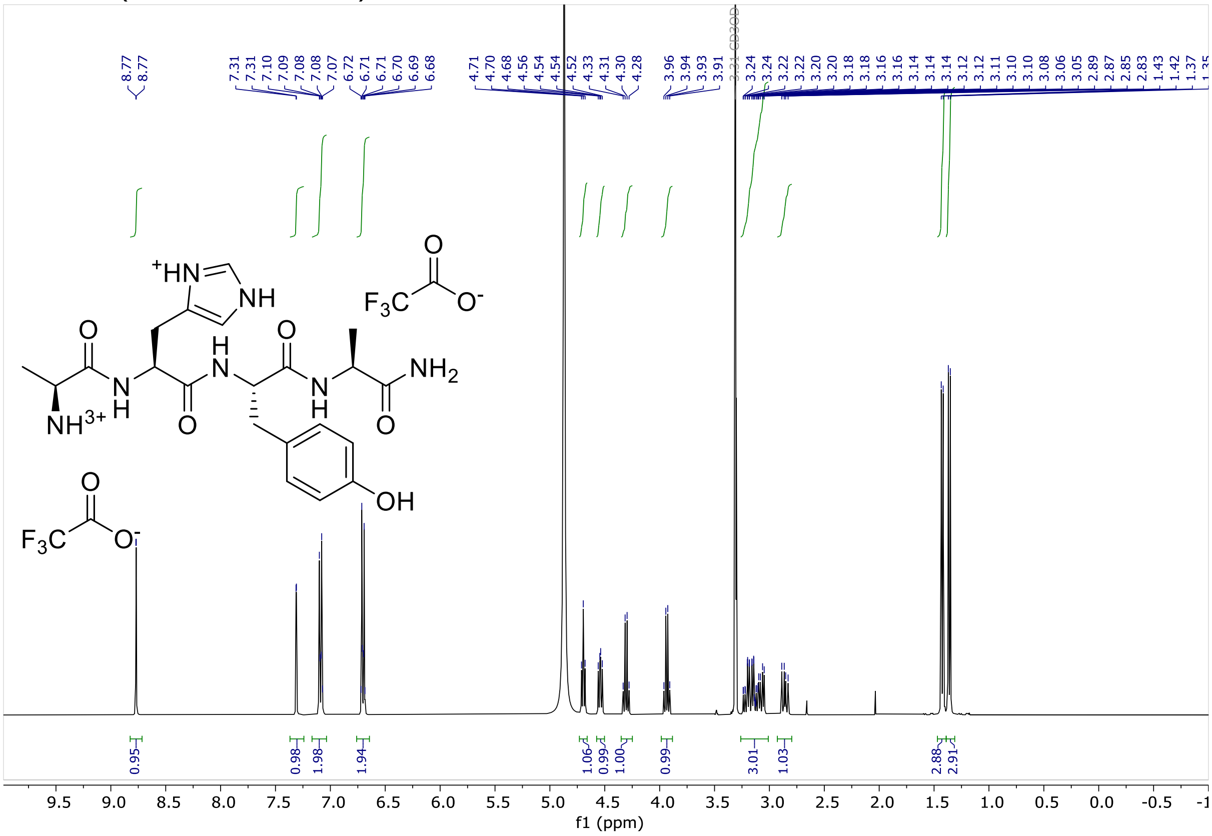

^{13}C NMR (101 MHz, CD_3OD)

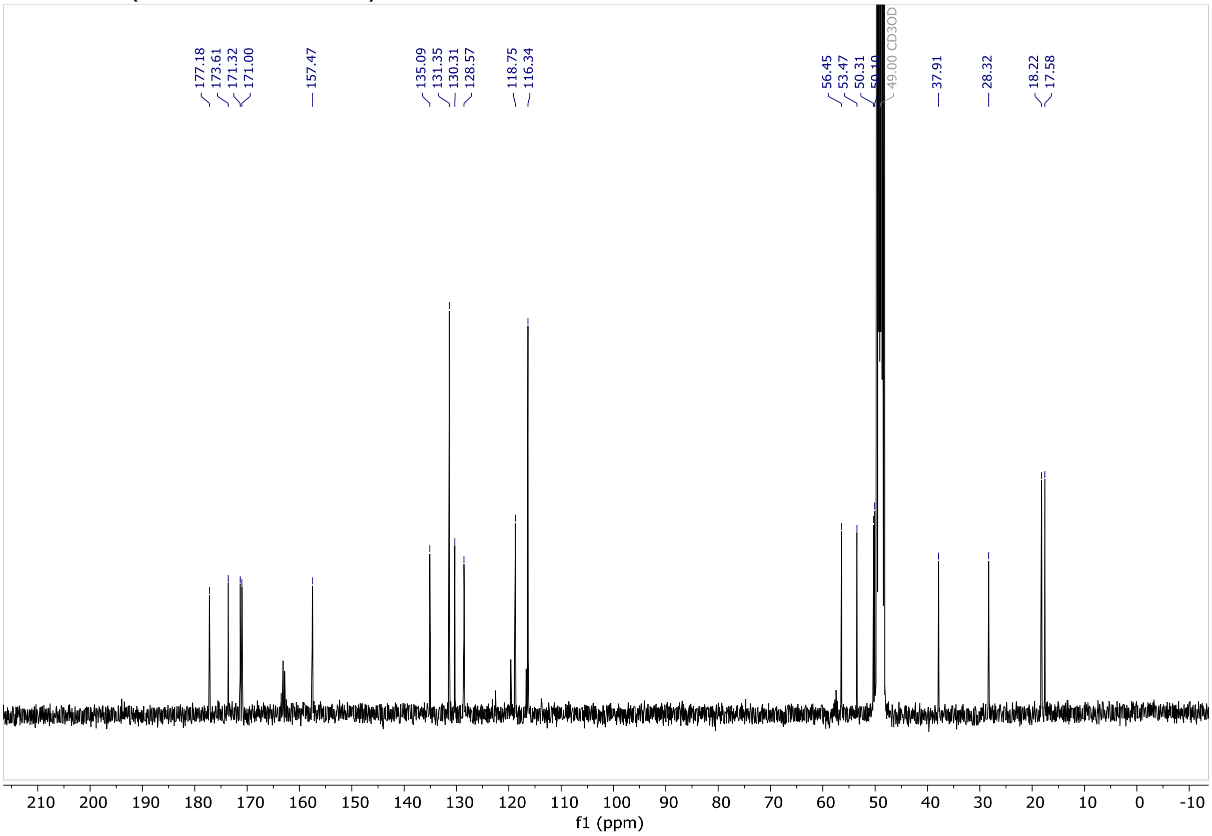

AKYA-NH$_2$ (1f)

^{1}H NMR (400 MHz, CD_3OD)

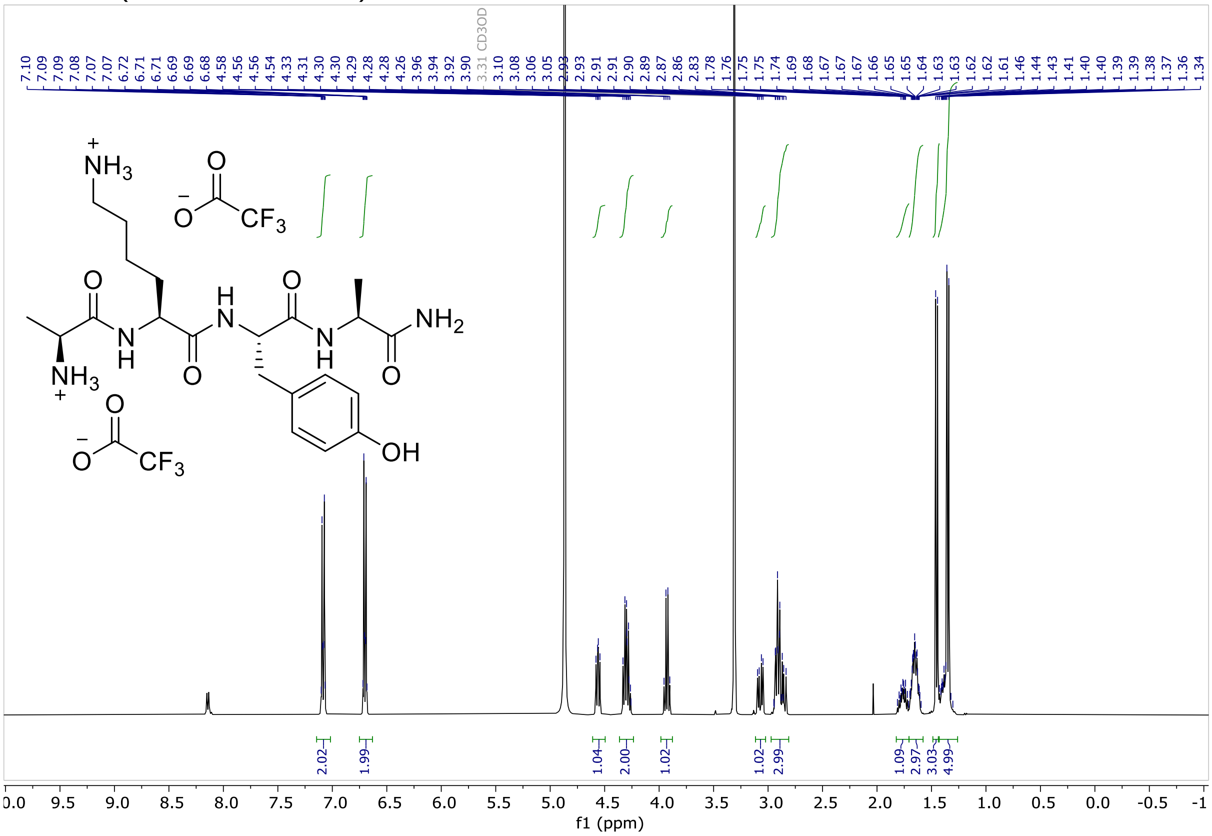

^{13}C NMR (101 MHz, CD_3OD)

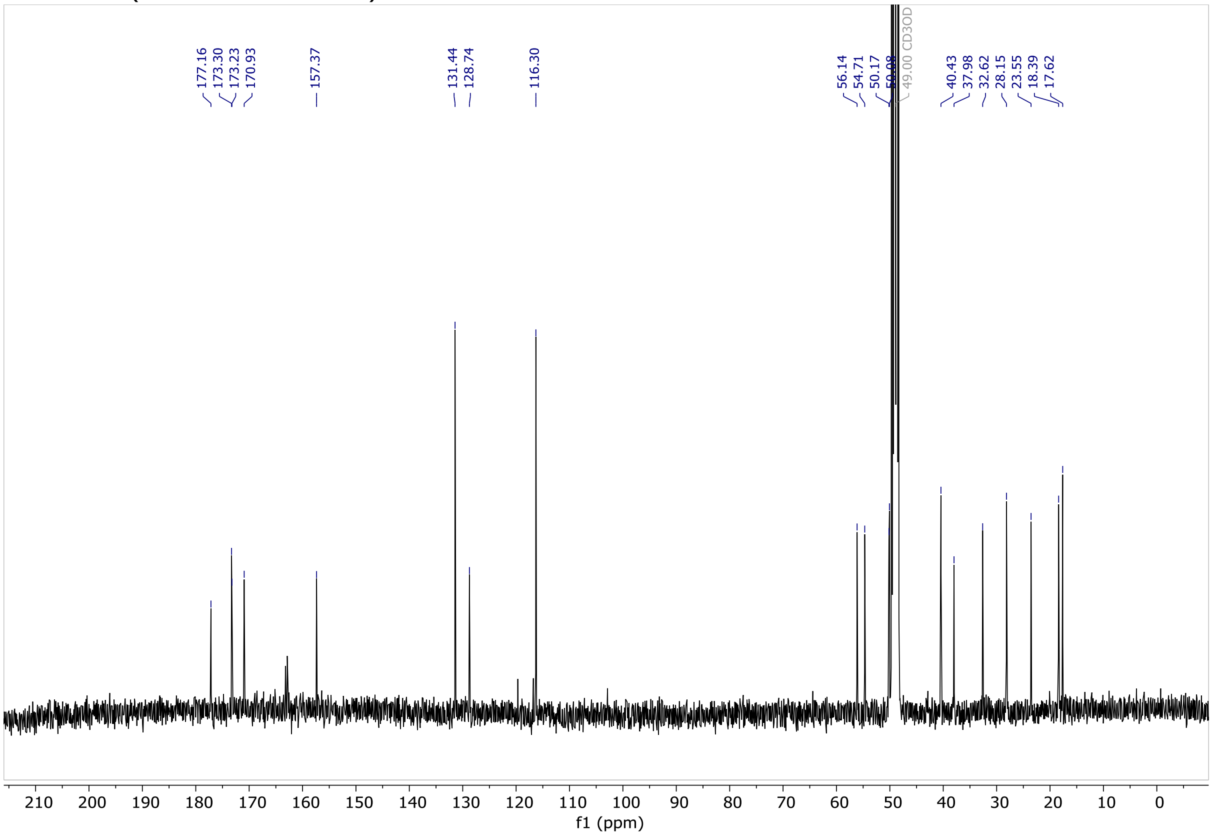

ALYA-NH₂ (1g)

^{1}H NMR (400 MHz, CD_3OD)

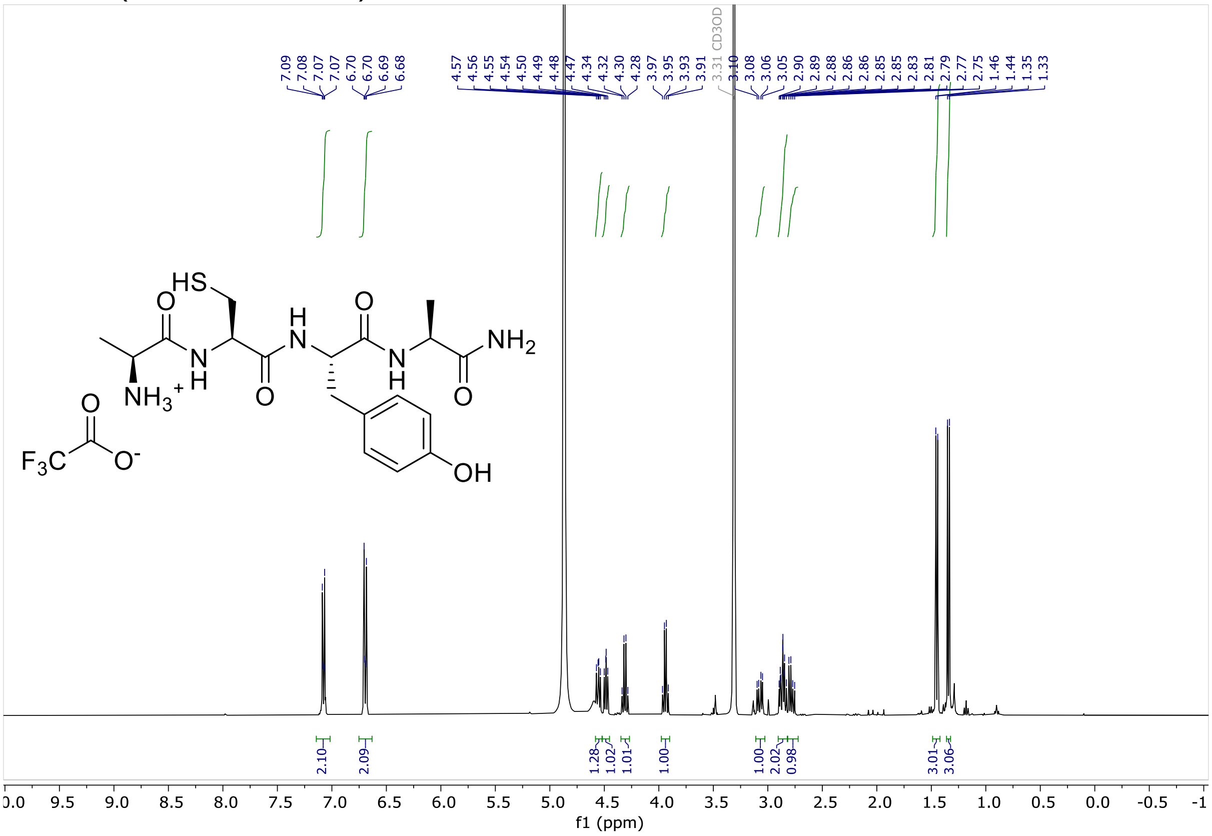

^{13}C NMR (101 MHz, CD_3OD)

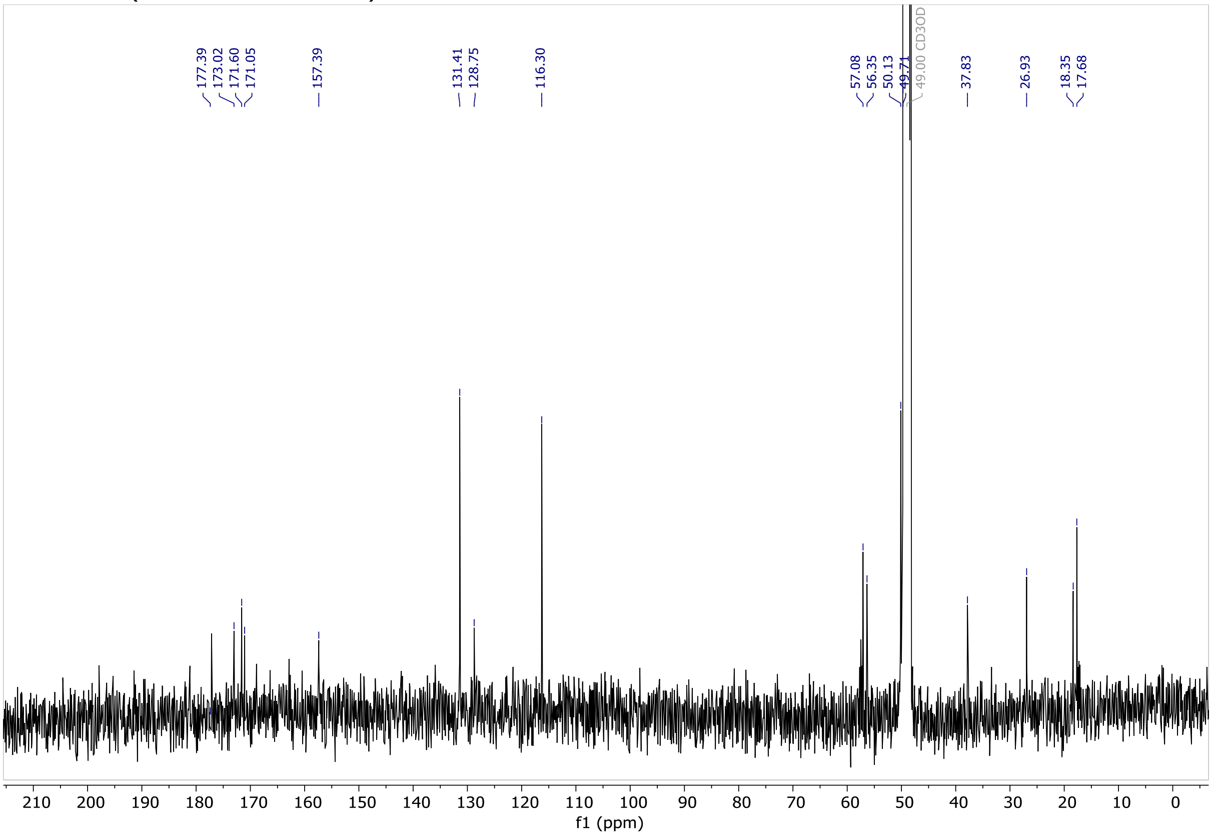

ASYA-NH$_2$ (1h)

^{1}H NMR (400 MHz, CD_3OD)

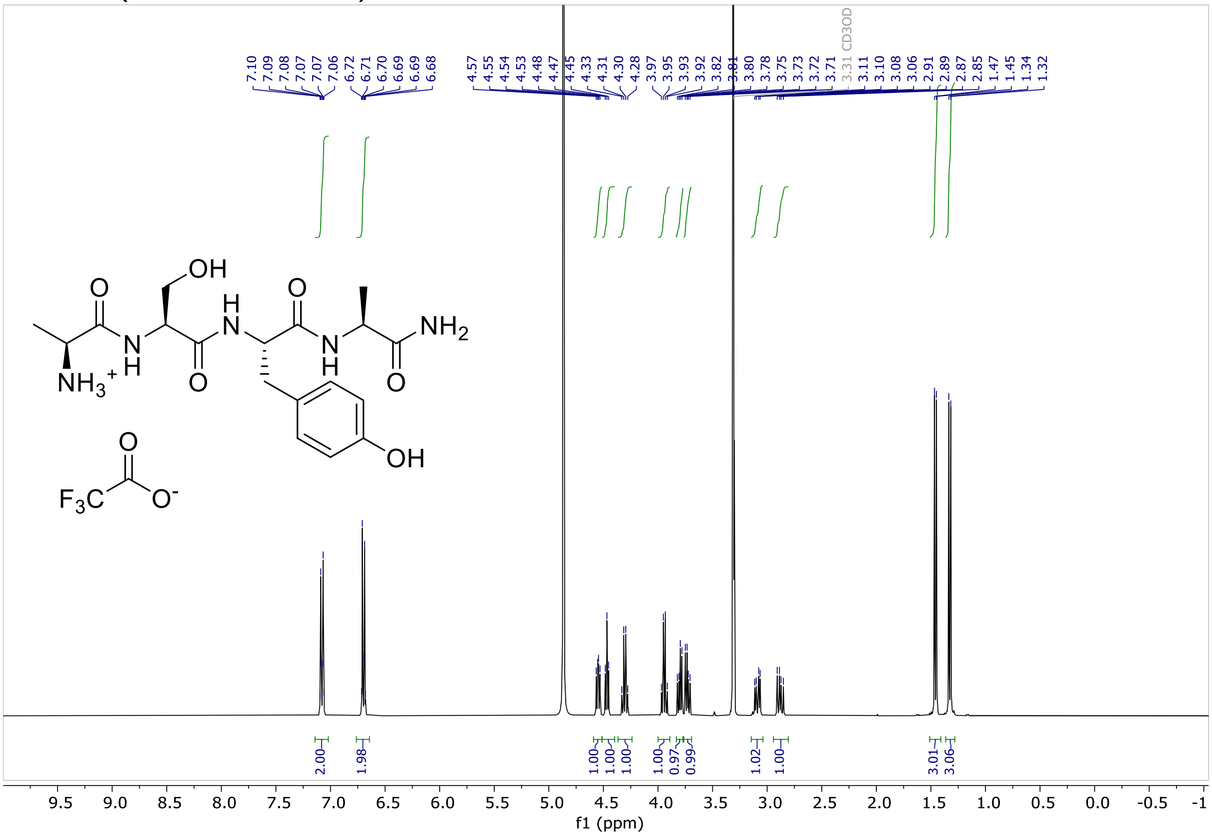

^{13}C NMR (101 MHz, CD_3OD)

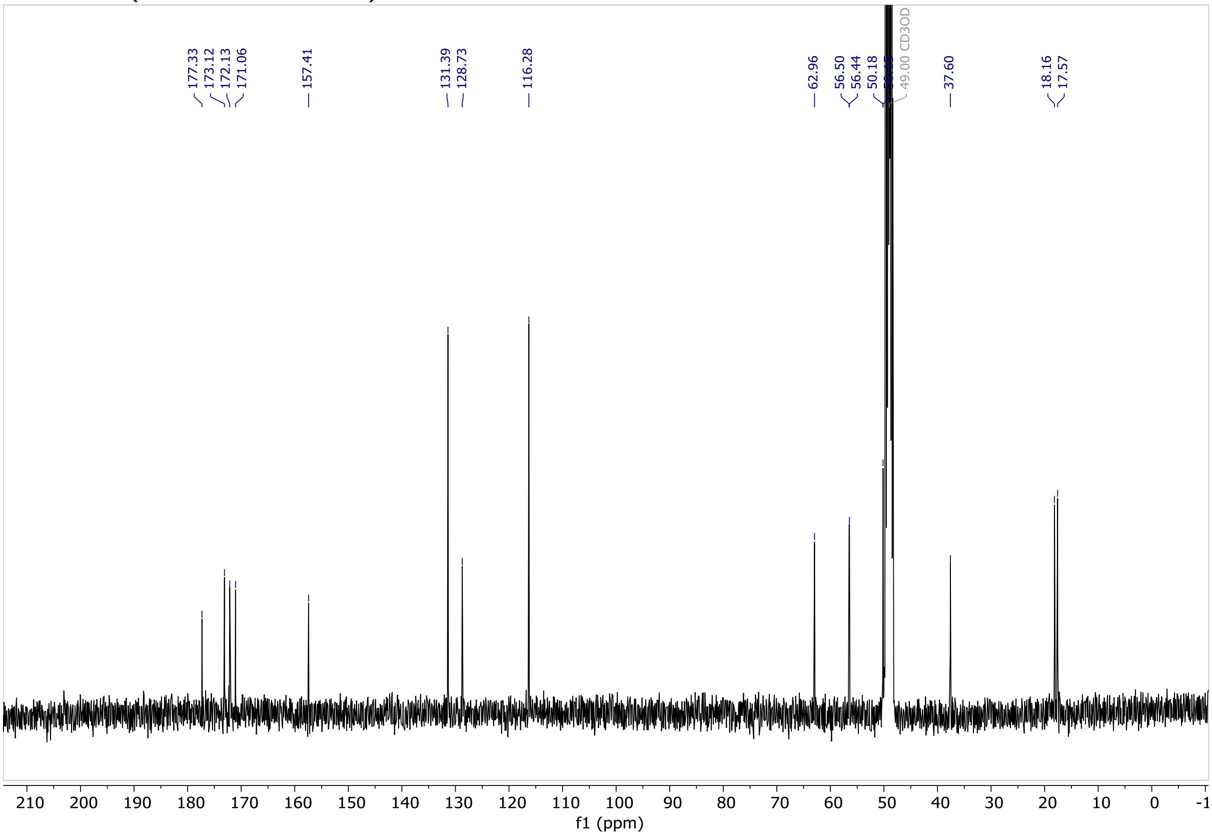

AMYA-NH$_2$ (1i)

^{1}H NMR (400 MHz, CD_3OD)

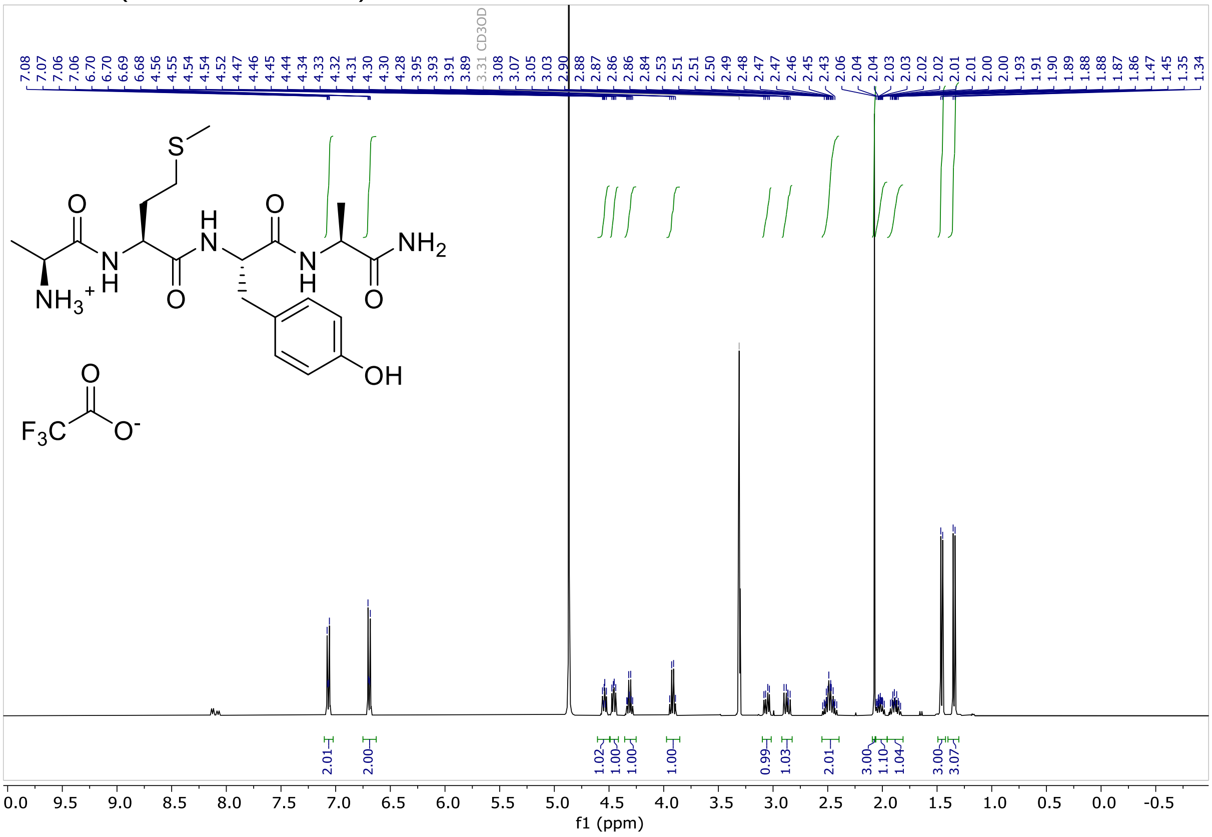

^{13}C NMR (101 MHz, CD_3OD)

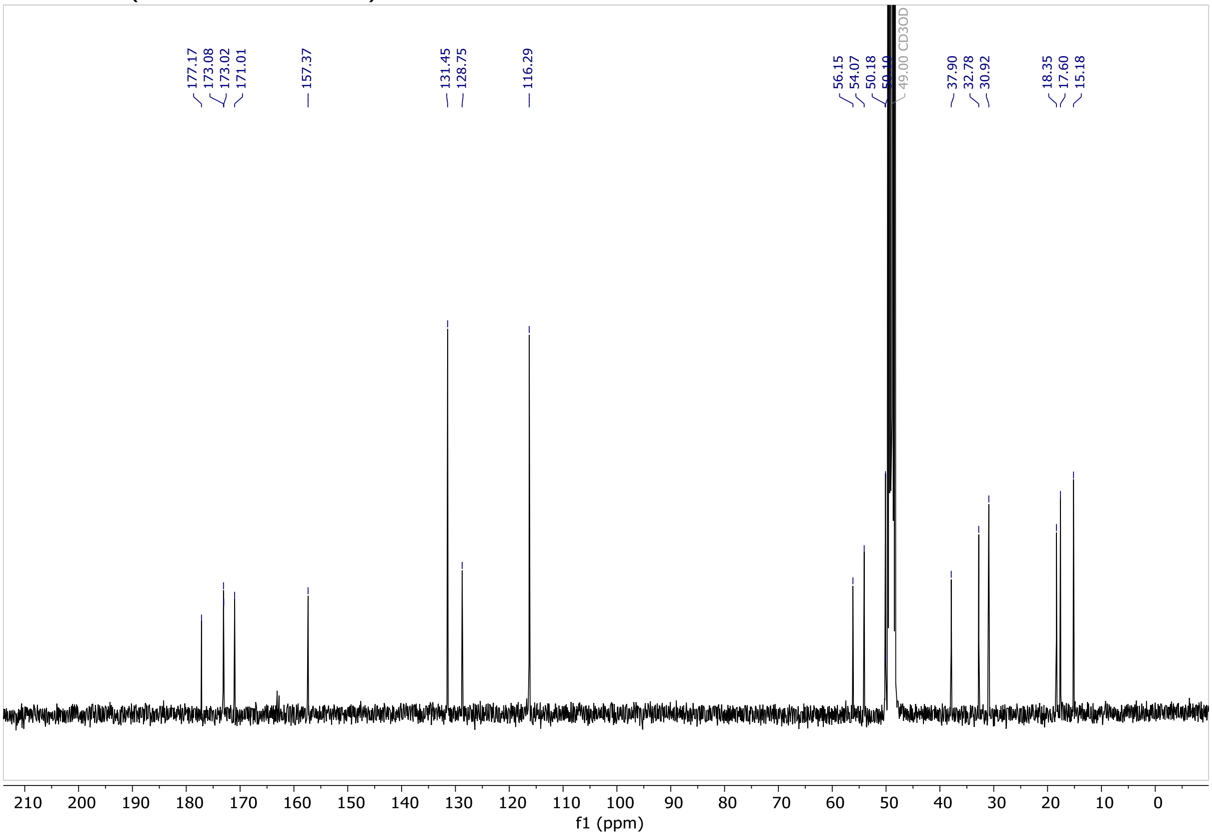

ADYA-NH₂ (1j)

^{1}H NMR (400 MHz, CD_3OD)

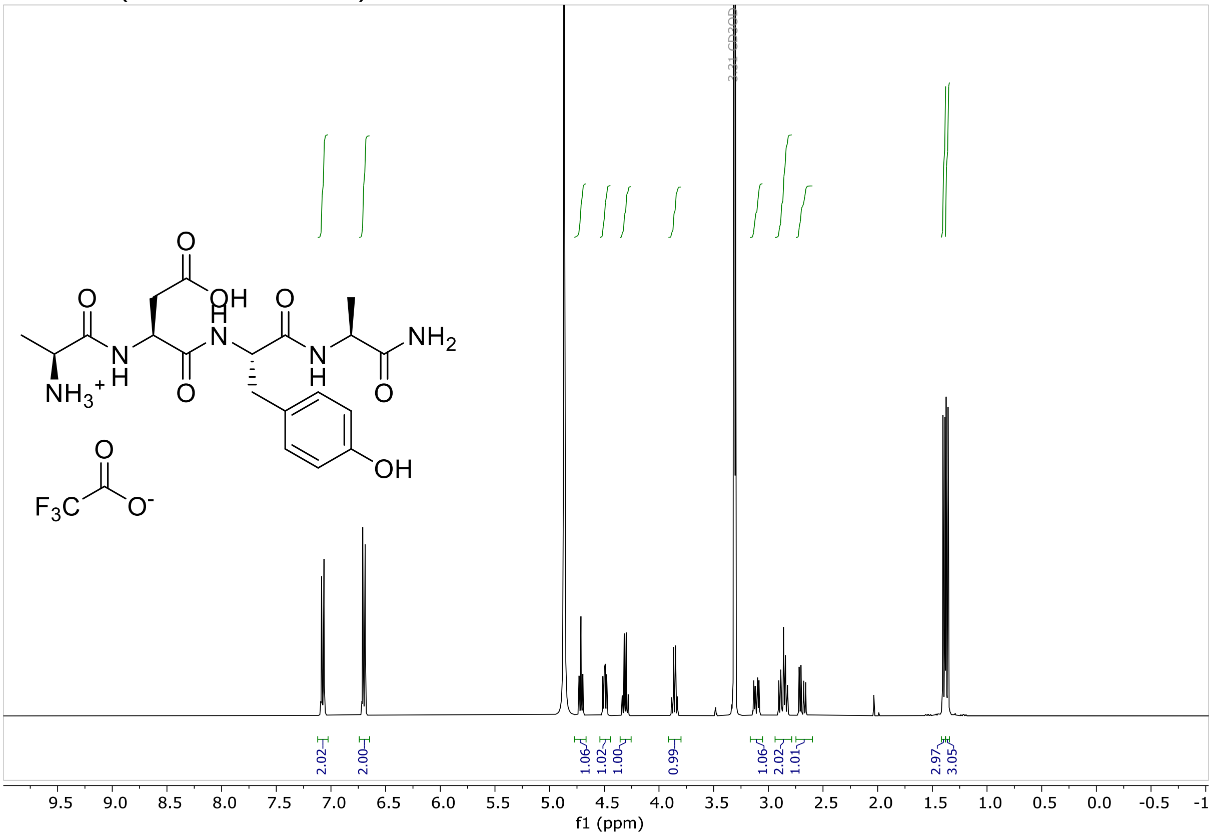

^{13}C NMR (101 MHz, CD_3OD)

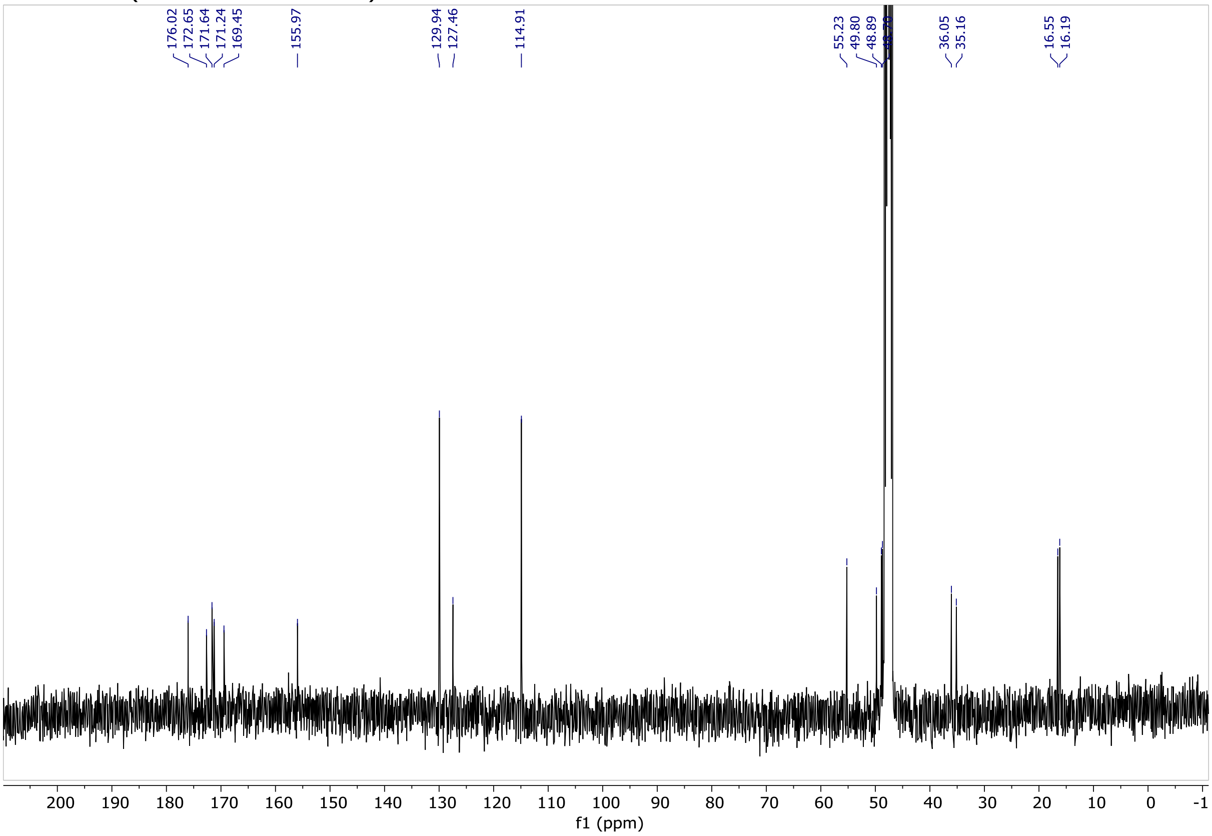

ANYA-NH$_2$ (1k)

^{1}H NMR (400 MHz, CD_3OD)

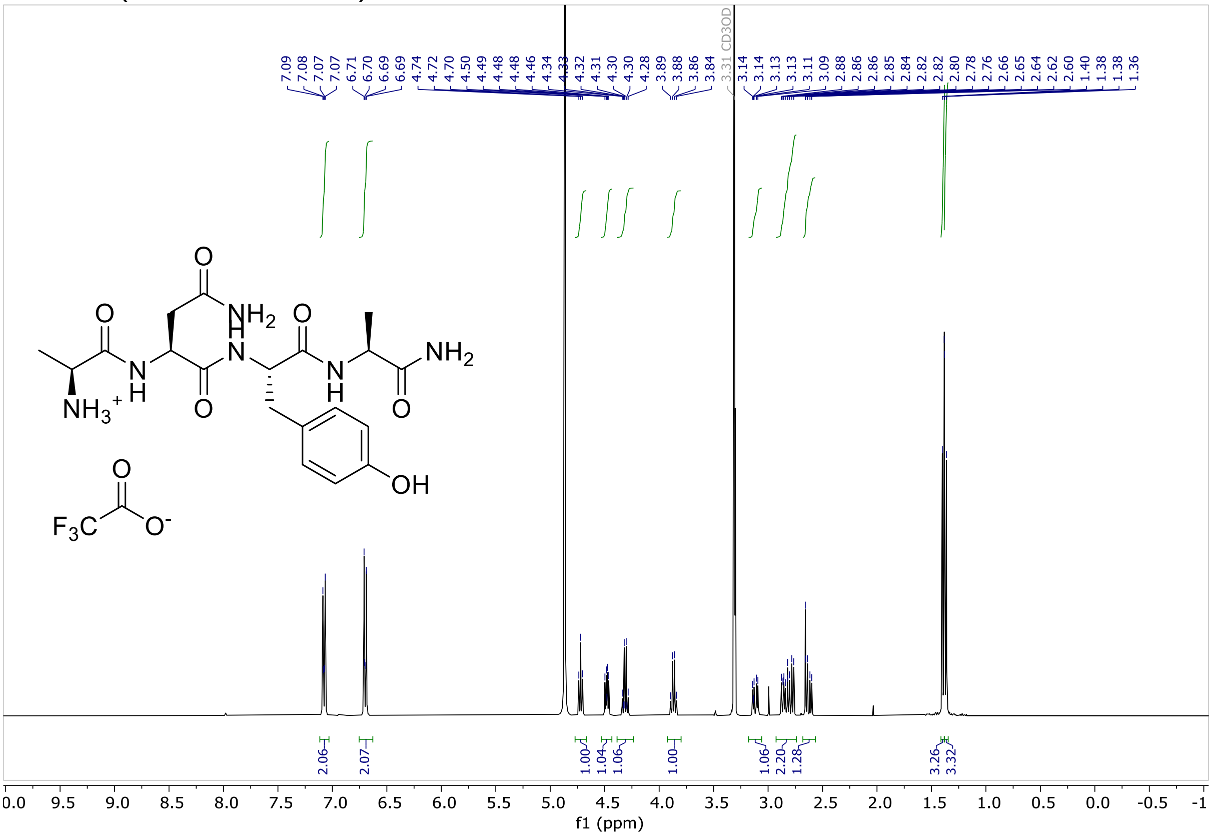

^{13}C NMR (101 MHz, CD_3OD)

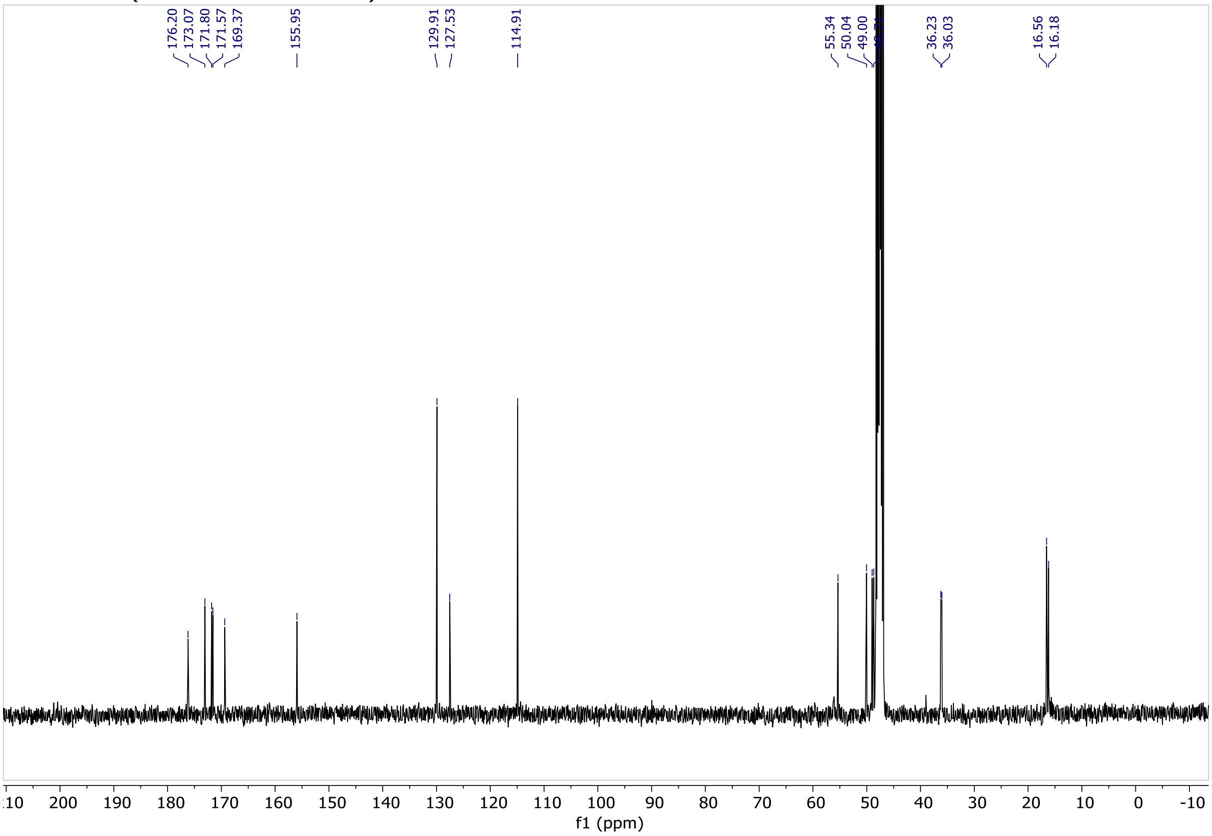

AYLA-NH_2 (1l)

^{1}H NMR (400 MHz, CD_3OD)

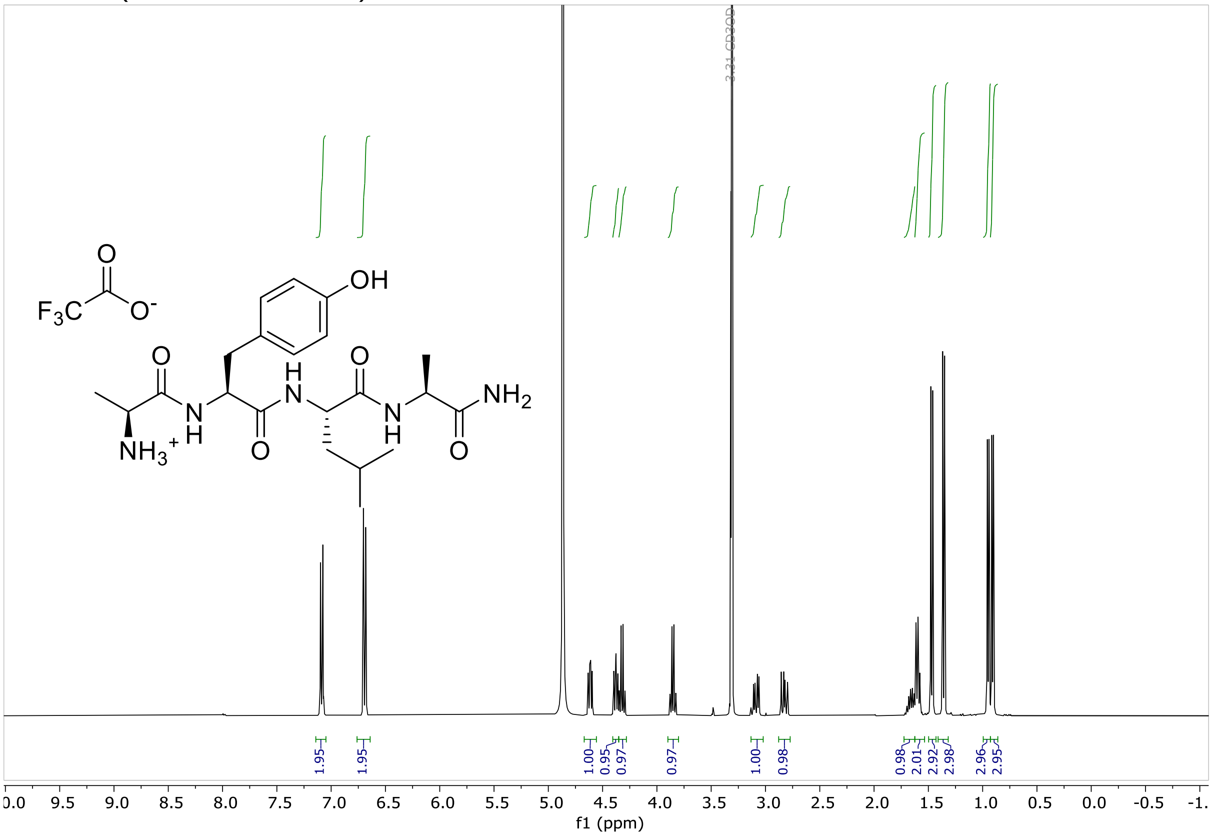

^{13}C NMR (101 MHz, CD_3OD)

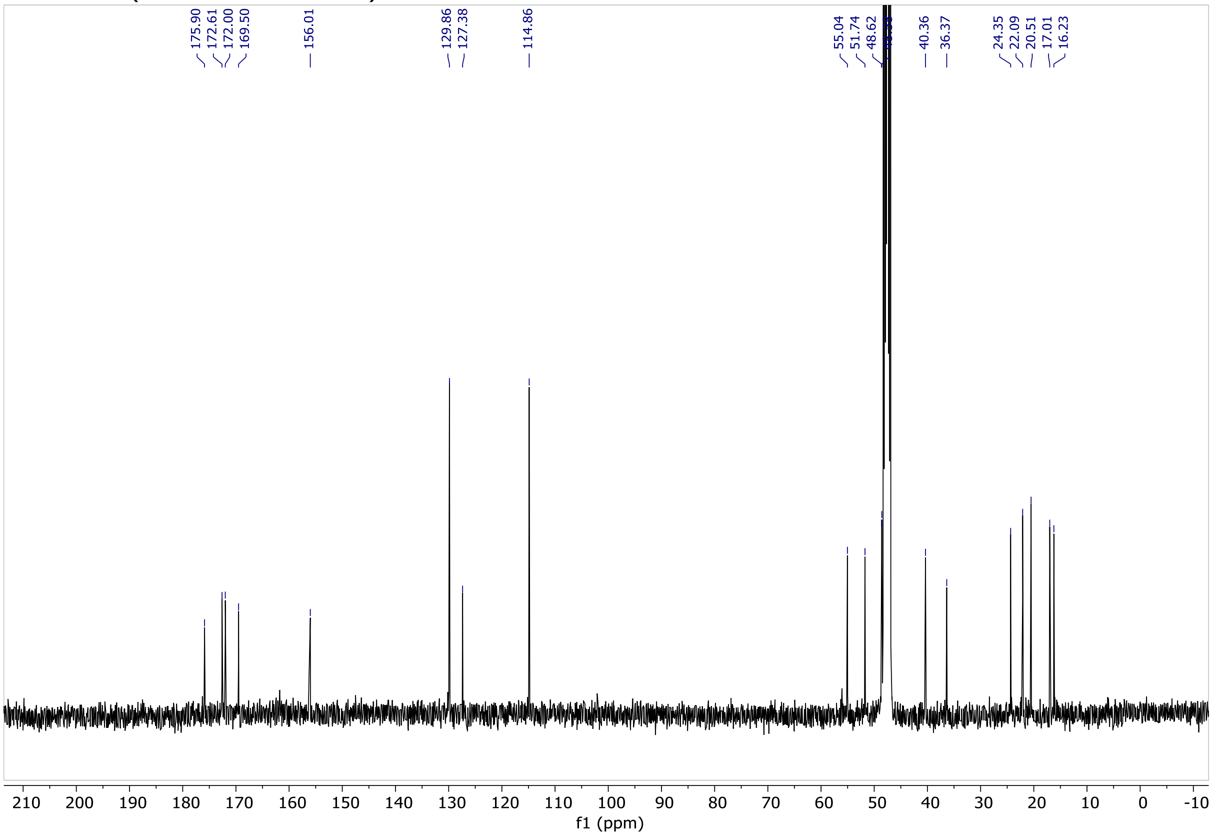

YALA-NH_2 (1m)

^1H NMR (400 MHz, CD_3OD)

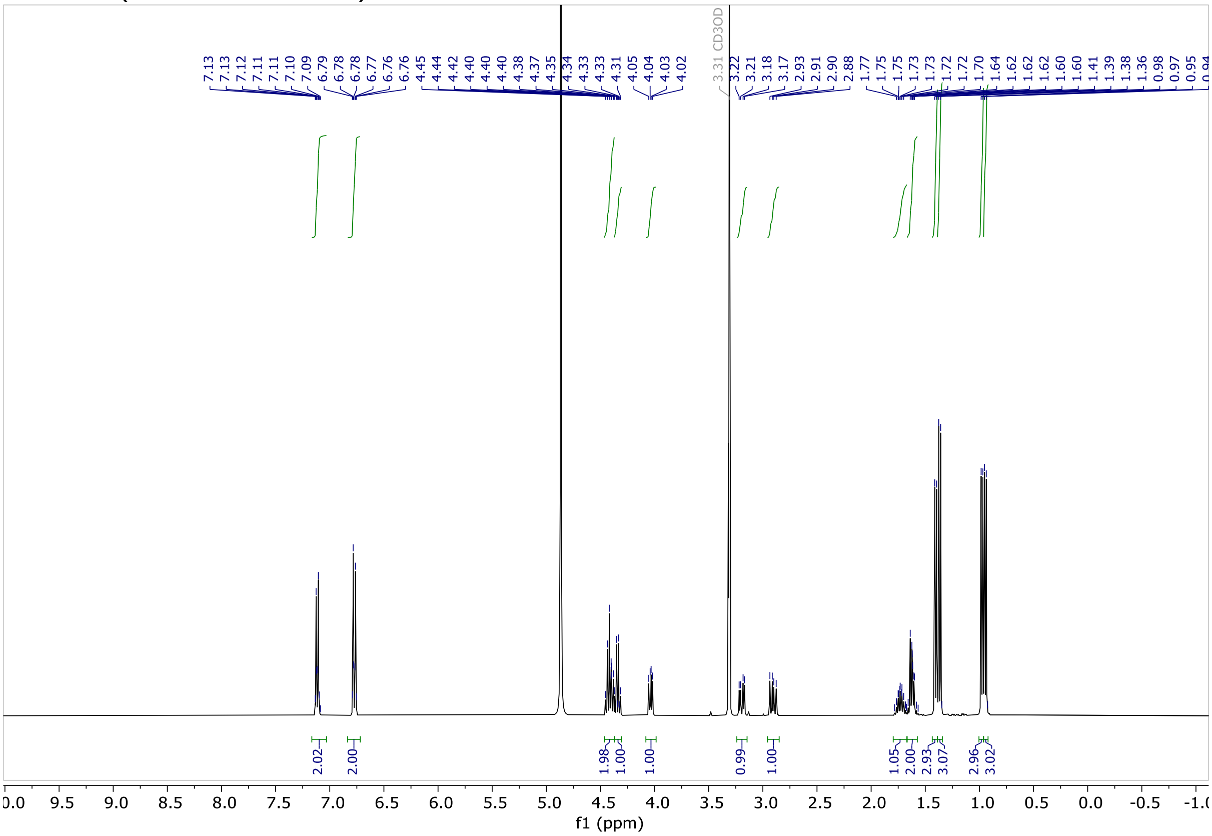

^{13}C NMR (101 MHz, CD_3OD)

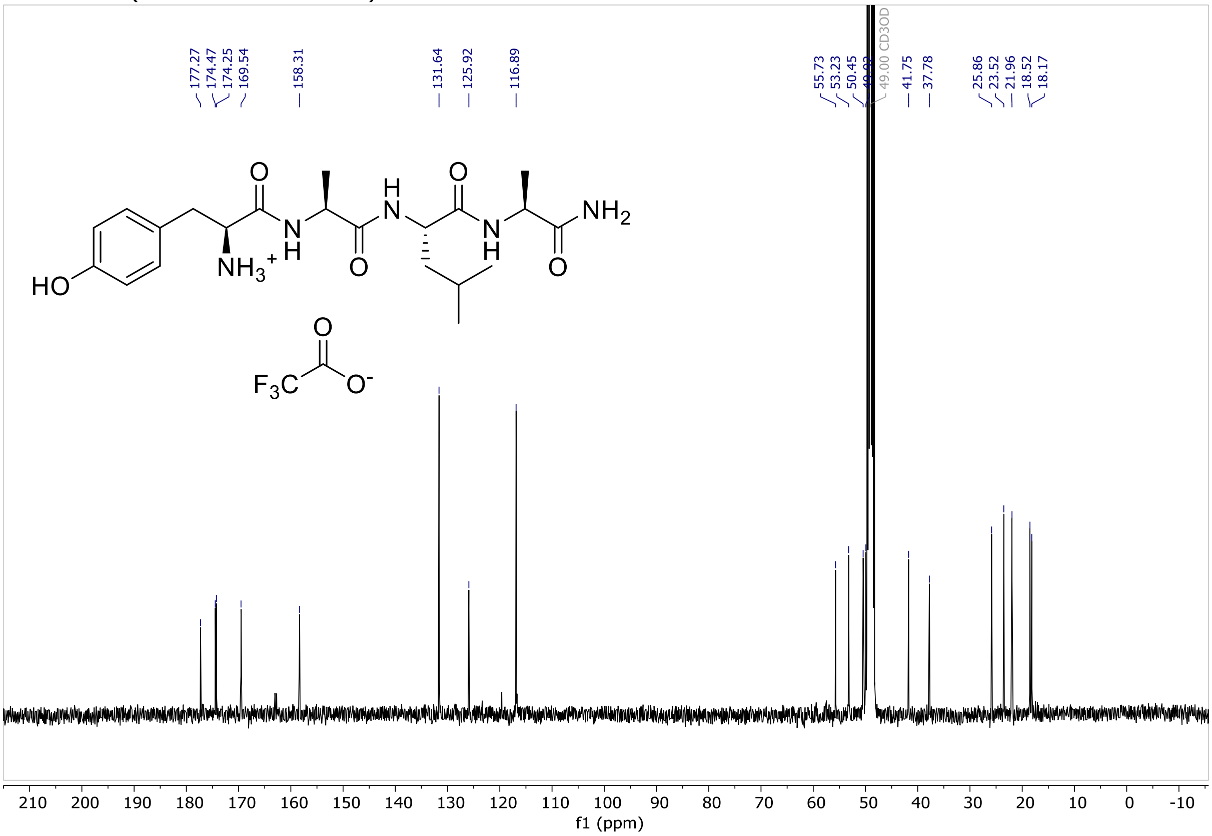

Ac-AFGY (1n)

^{1}H NMR (400 MHz, CD_3OD)

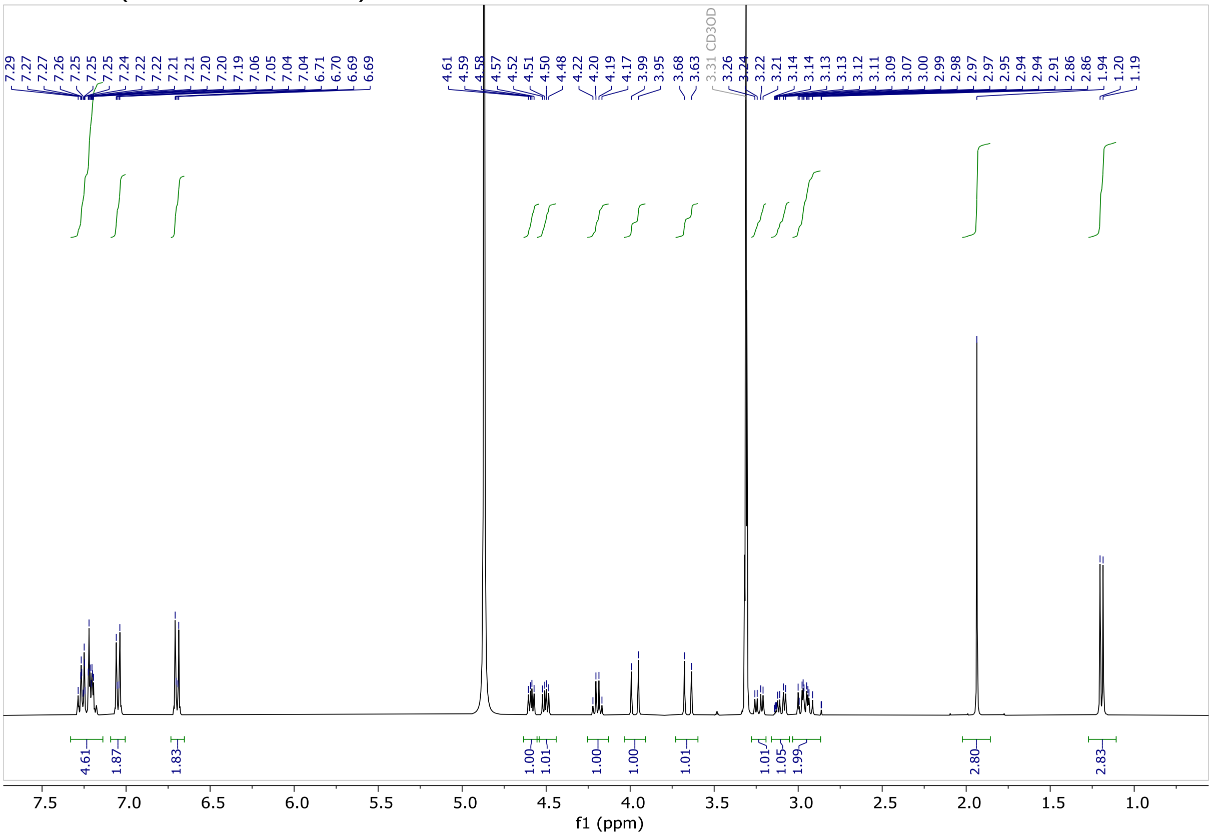

^{13}C NMR (101 MHz, CD_3OD)

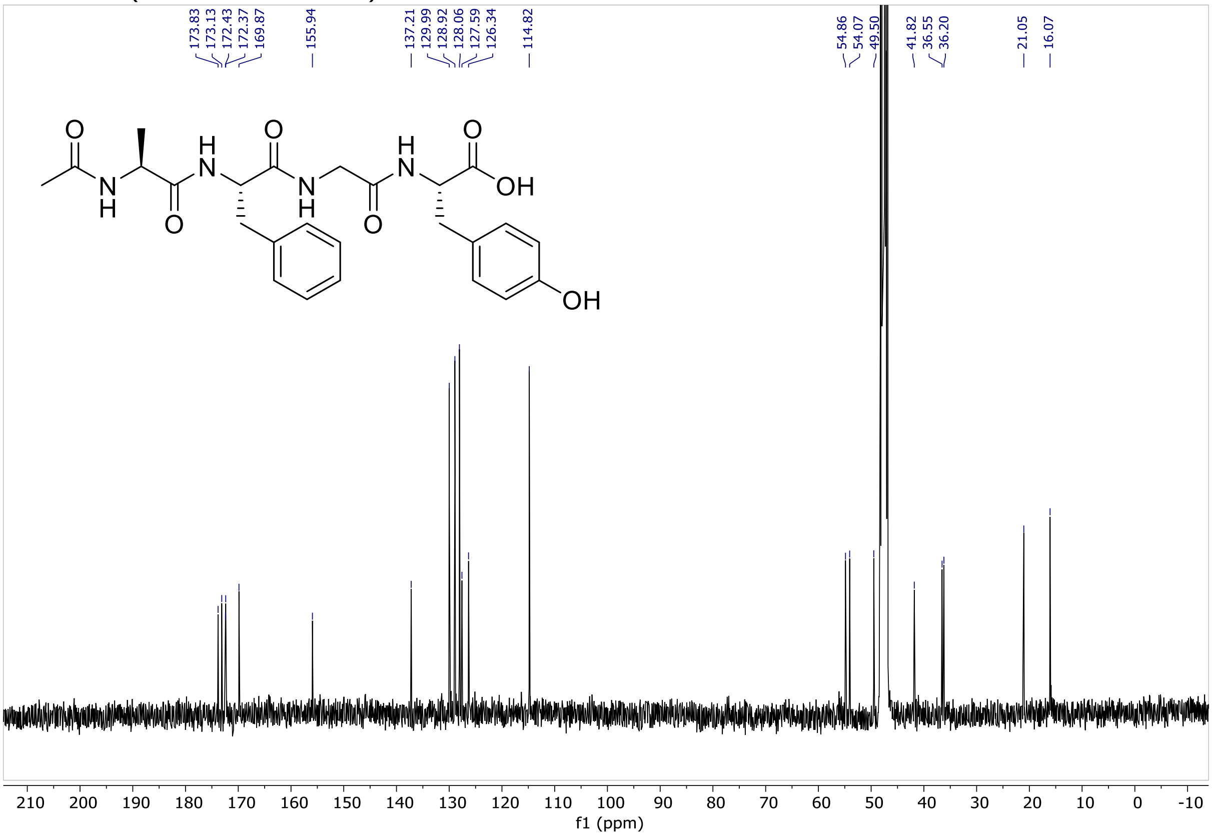

O-VBX AFYA-NH₂ (3a)

^{1}H NMR (400 MHz, CD_3OD)

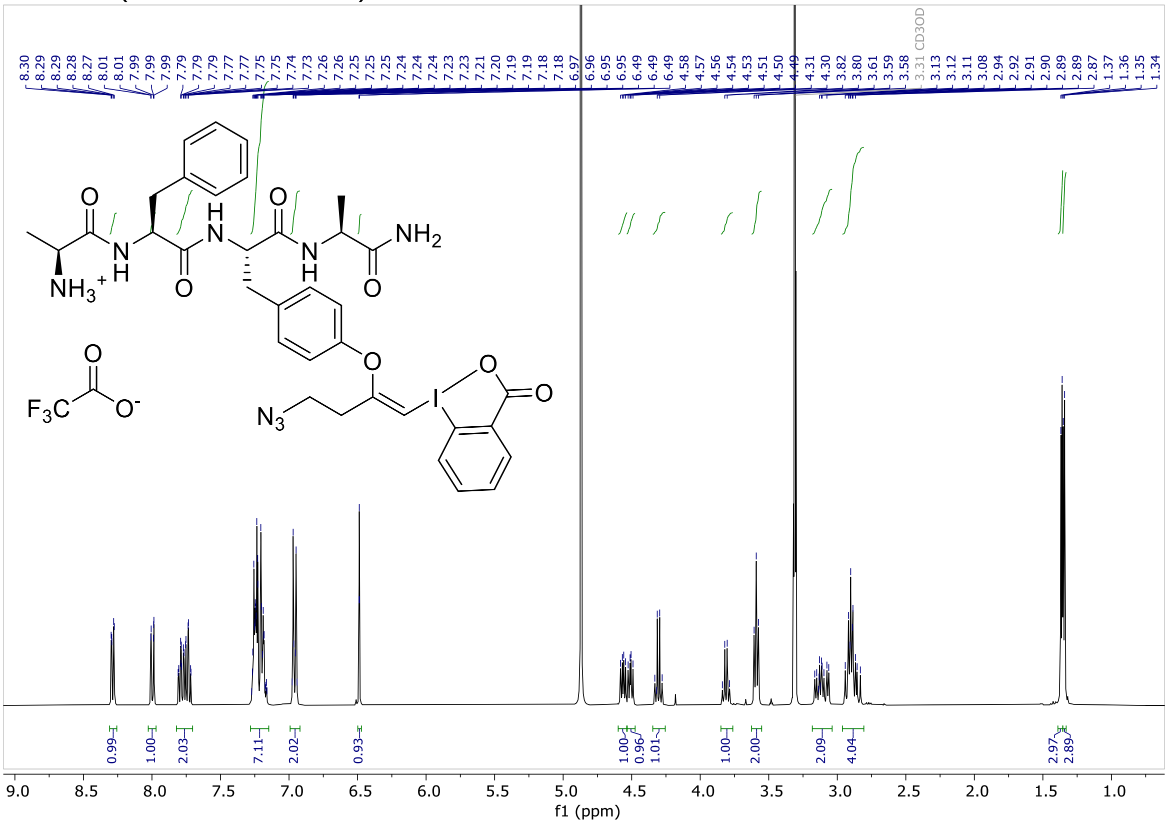

^{13}C NMR (101 MHz, CD_3OD)

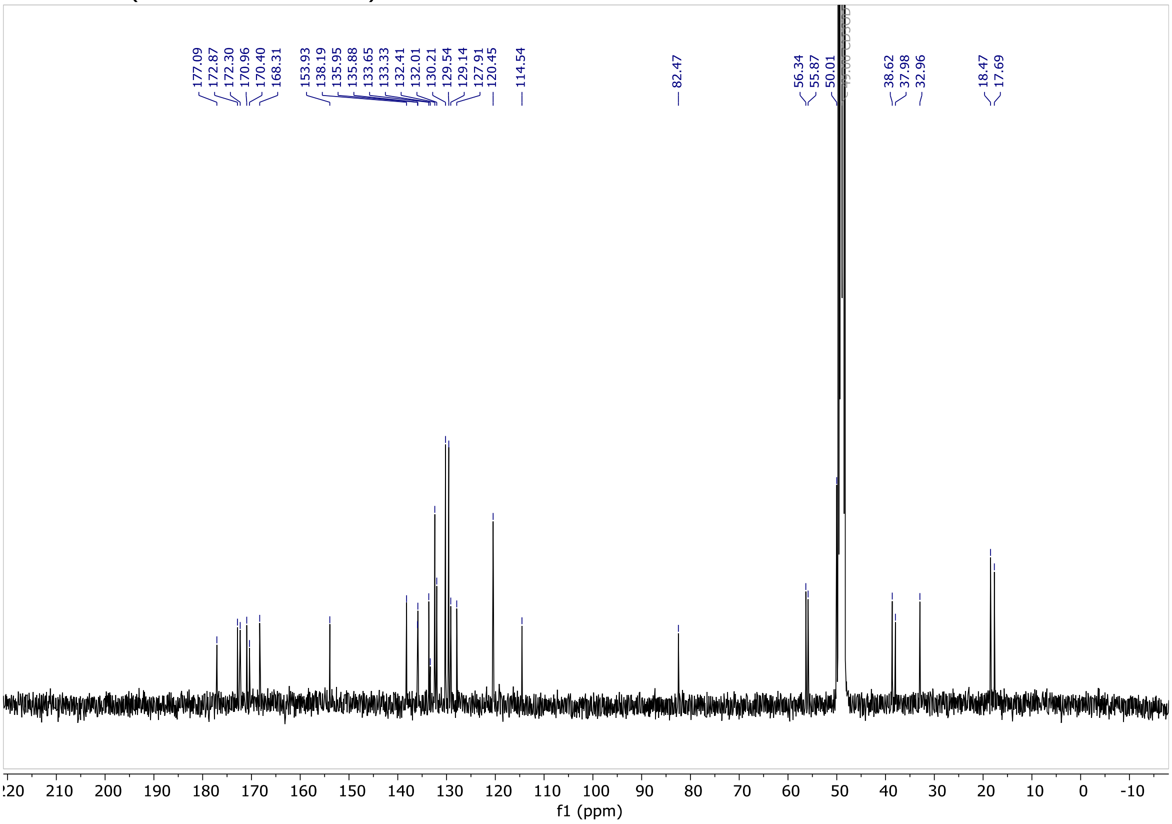

O-VBX ALYA-NH$_2$ (3b)

^{1}H NMR (400 MHz, CD_3OD)

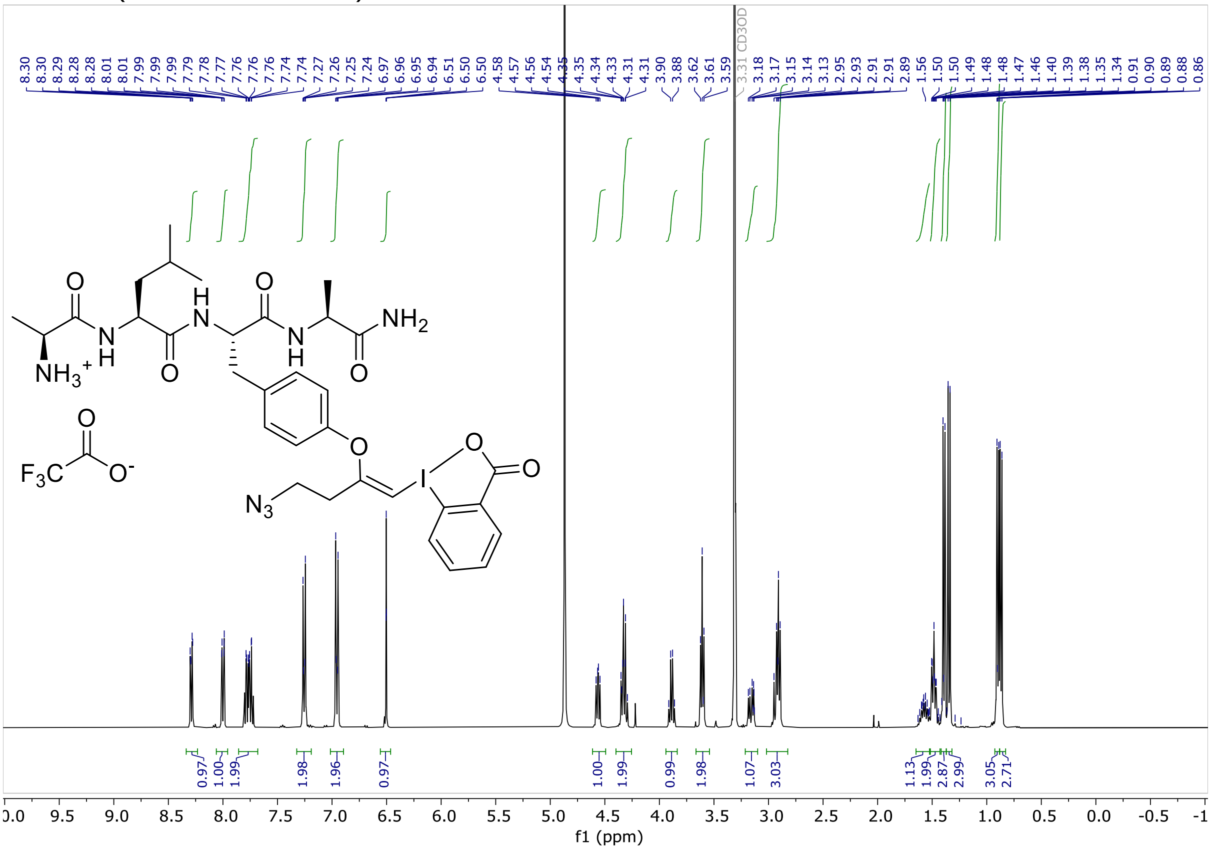

^{13}C NMR (101 MHz, CD_3OD)

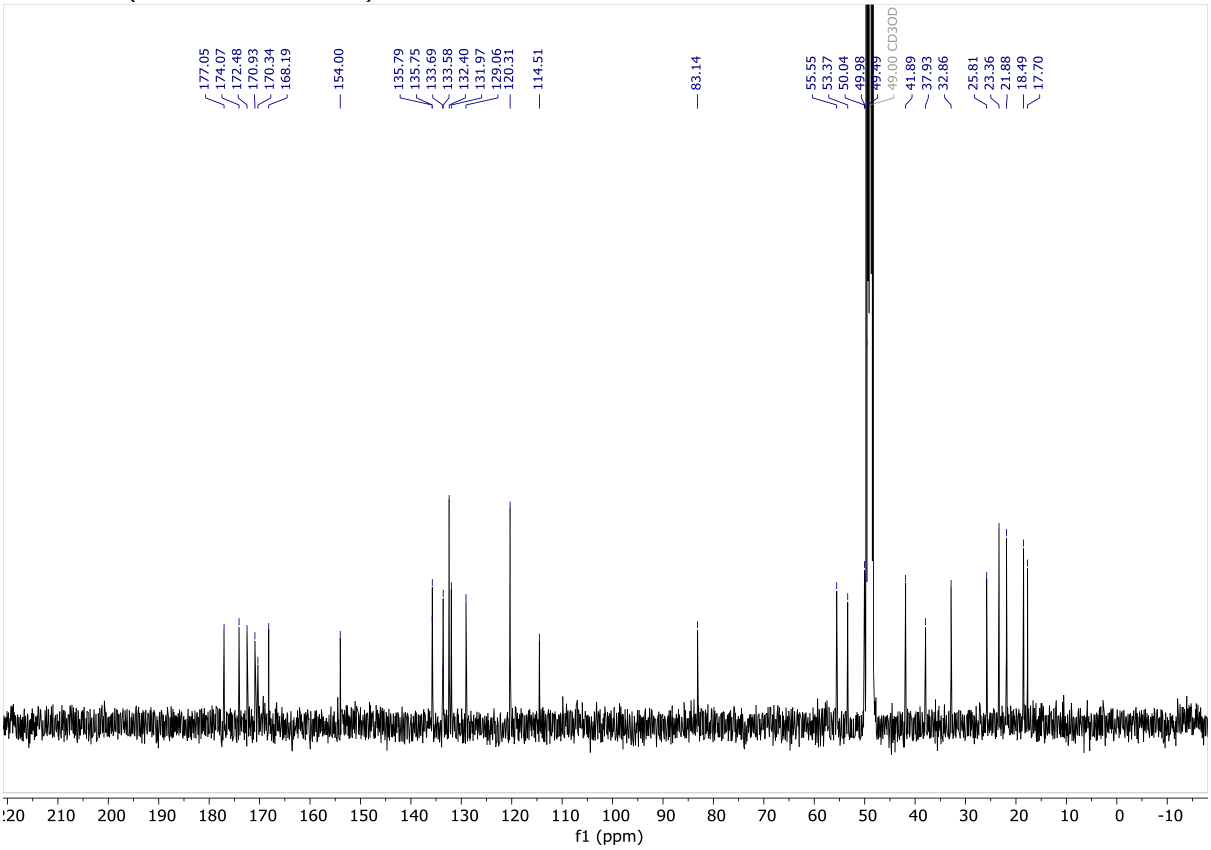

O-VBX AWYA-NH₂ (3c)

^{1}H NMR (400 MHz, CD_3OD)

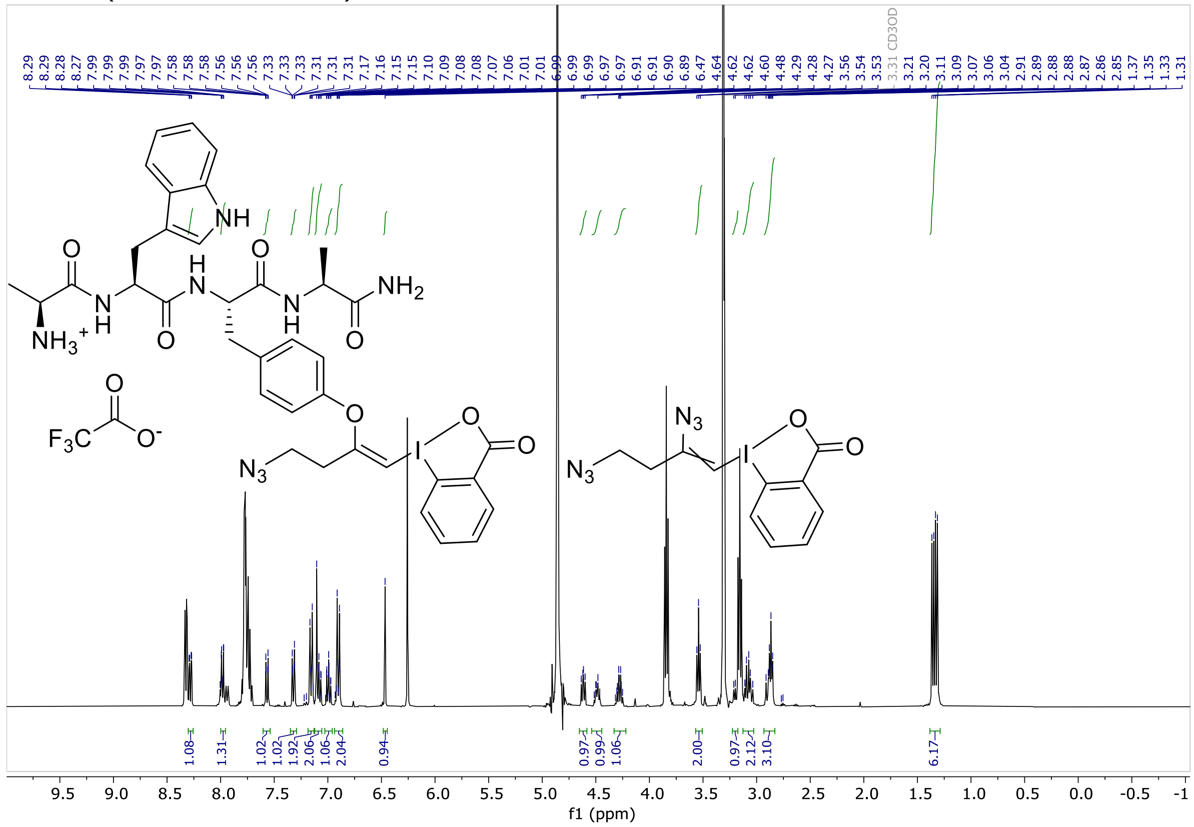

^{13}C NMR (101 MHz, CD_3OD)

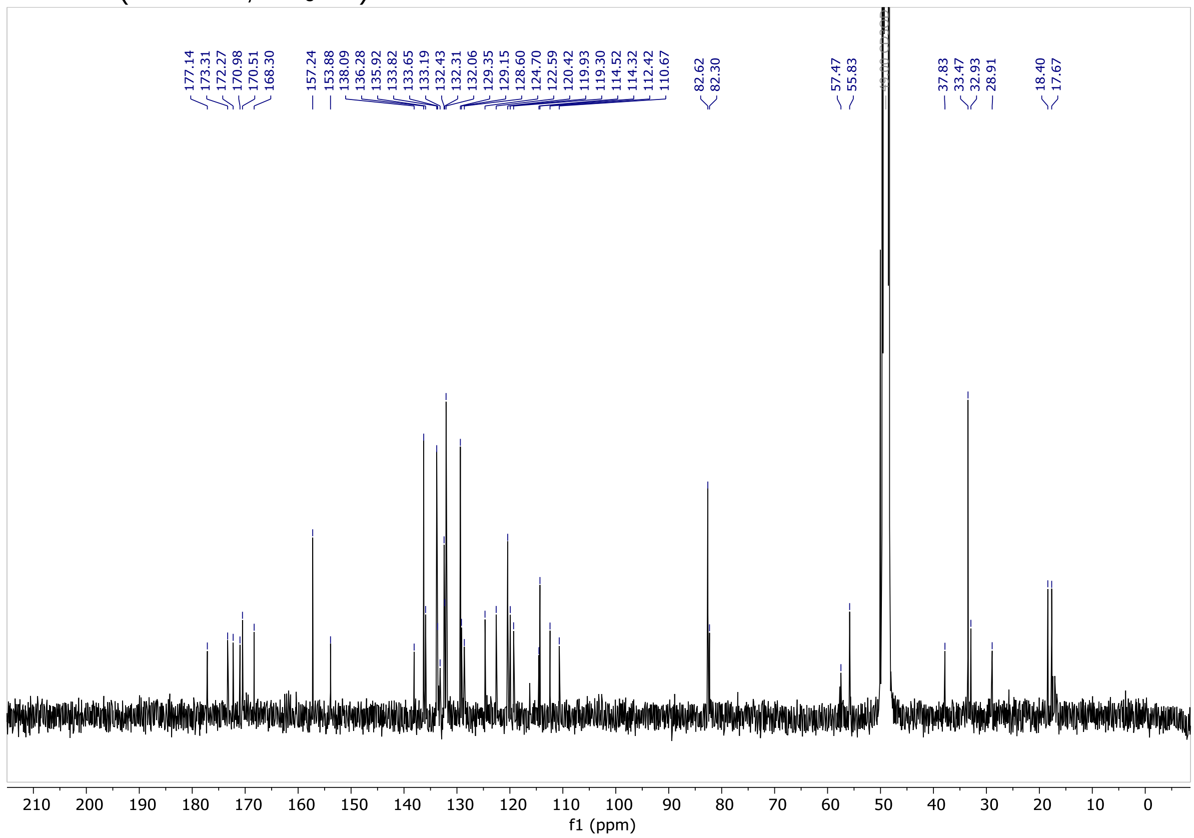

N_3-VBX (4)

^1H NMR (400 MHz, DMSO-d_6)

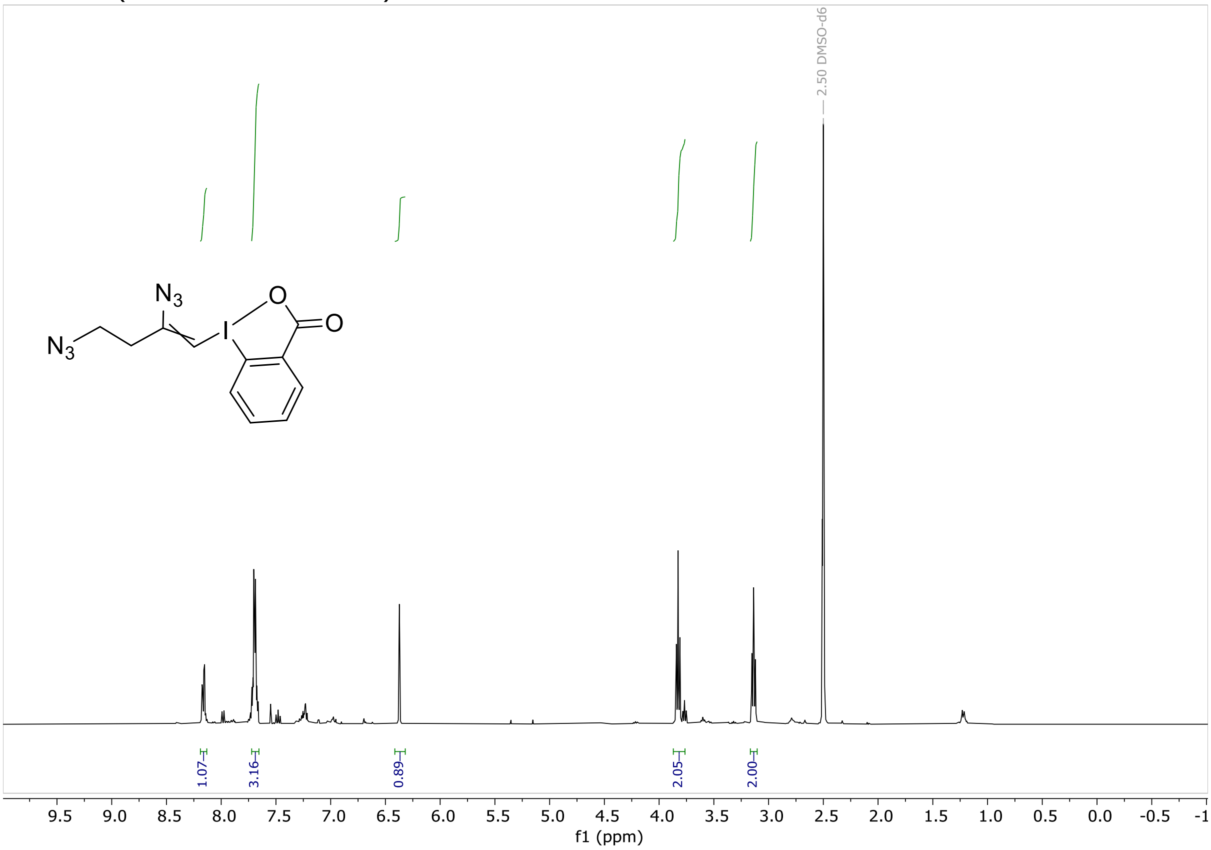

^{13}C NMR (101 MHz, DMSO-d_6)

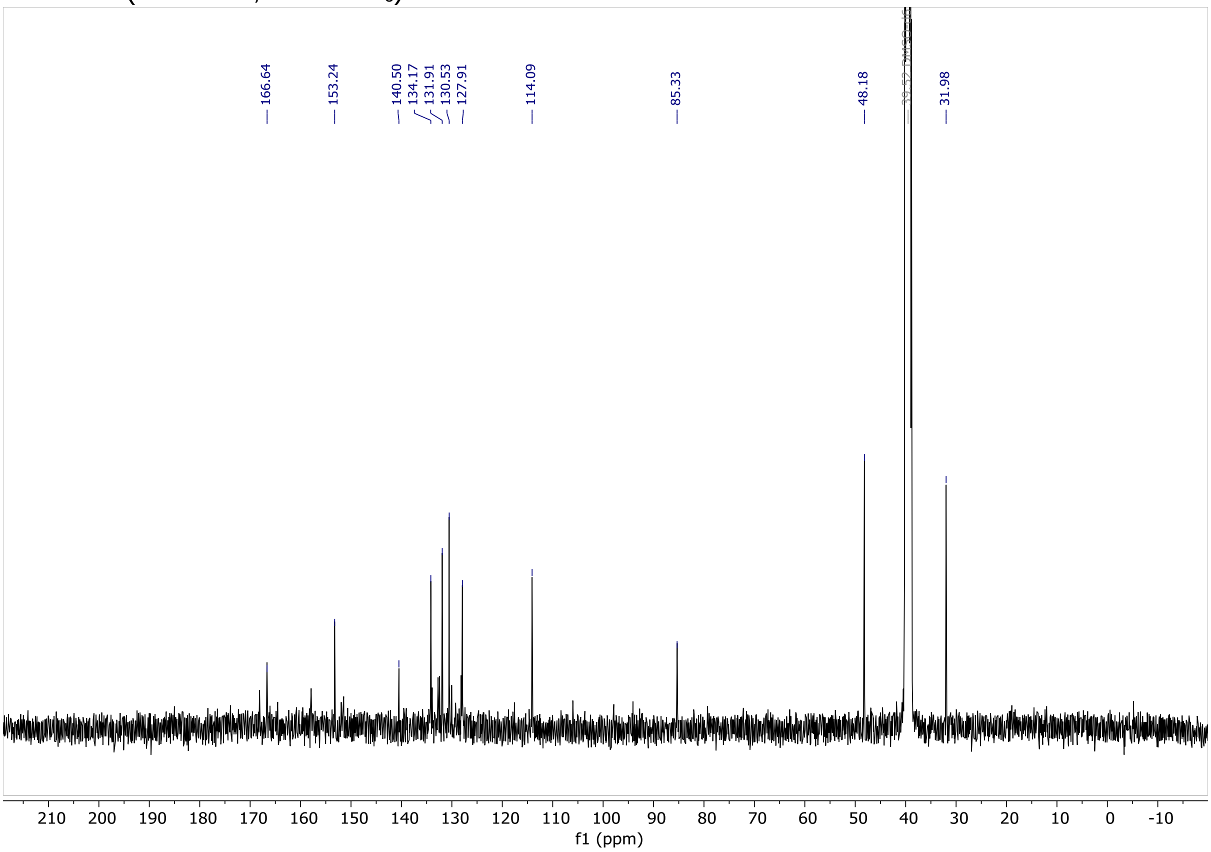

O-VBX ARYA-NH₂ (3d)

^{1}H NMR (400 MHz, CD_3OD)

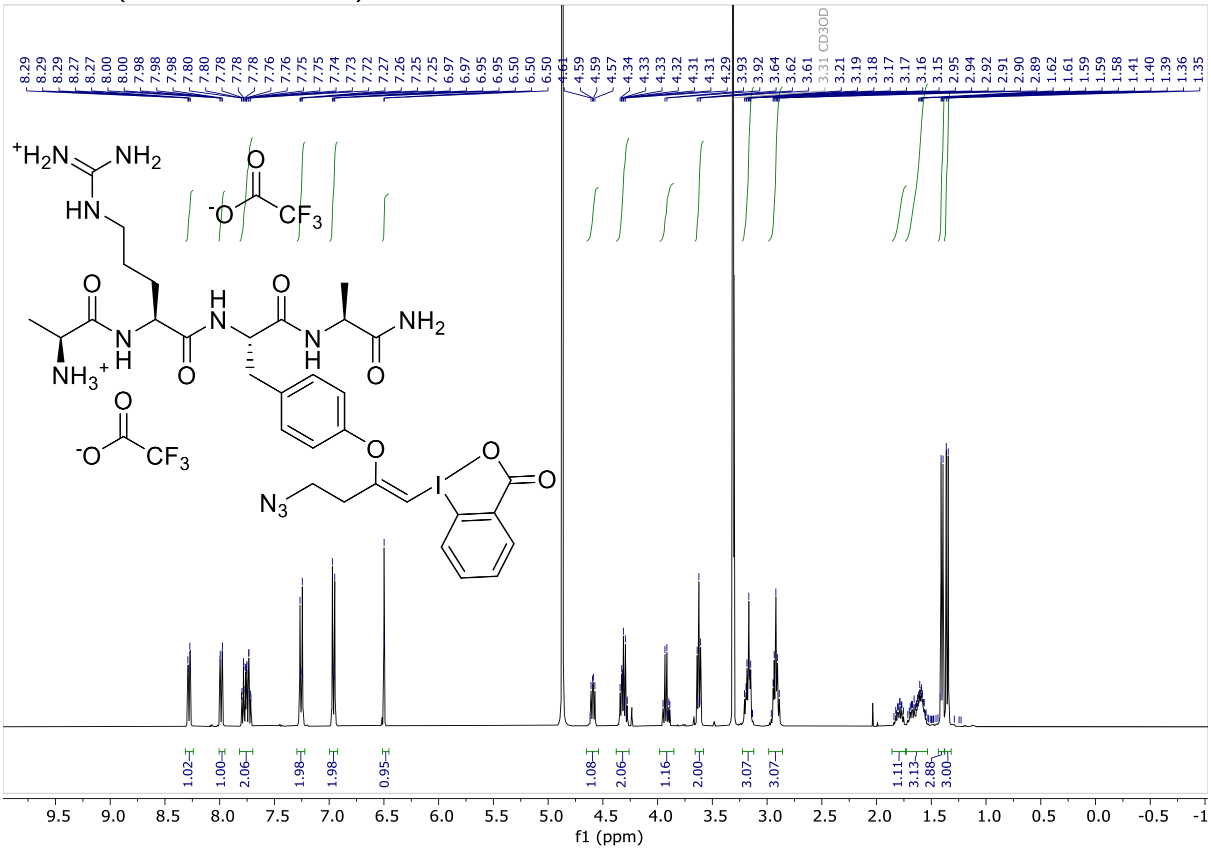

^{13}C NMR (101 MHz, CD_3OD)

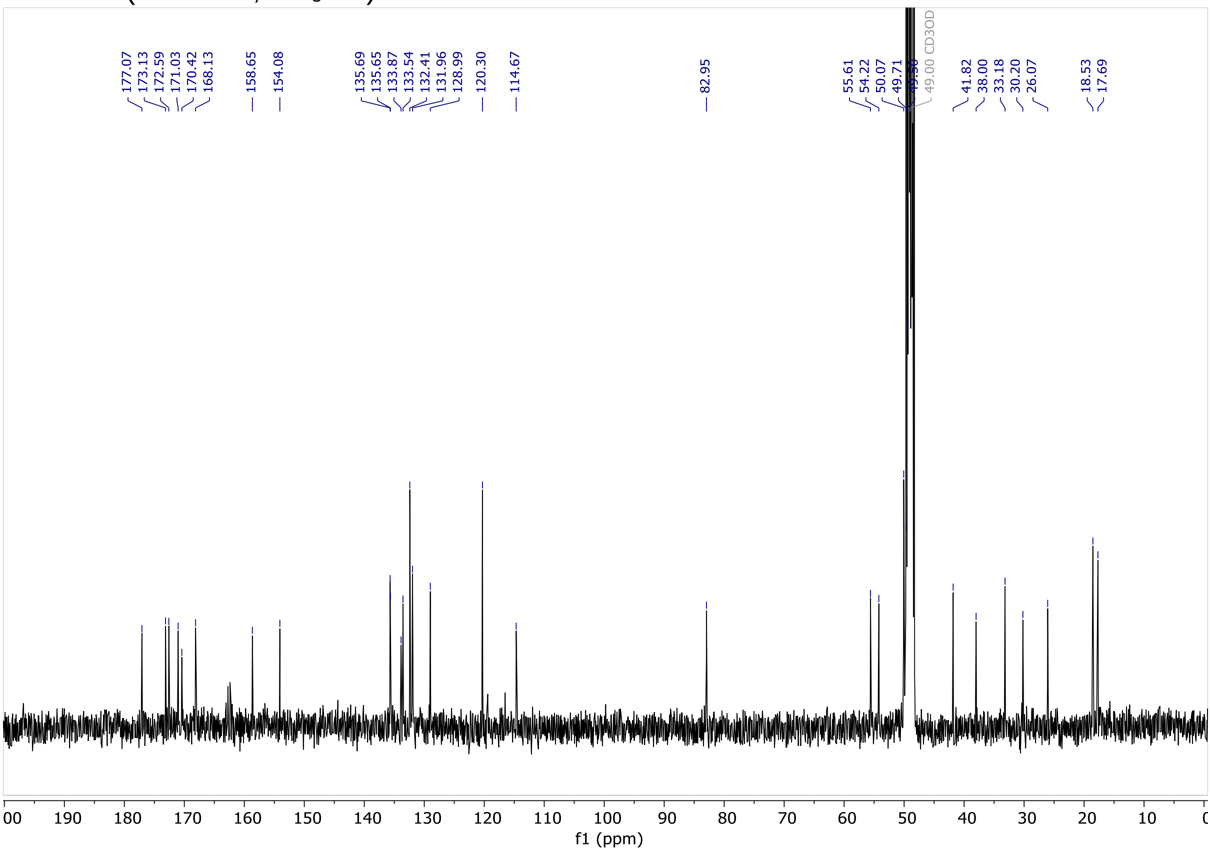

O-VBX AHYA-NH₂ (3e)

^{1}H NMR (400 MHz, CD_3OD)

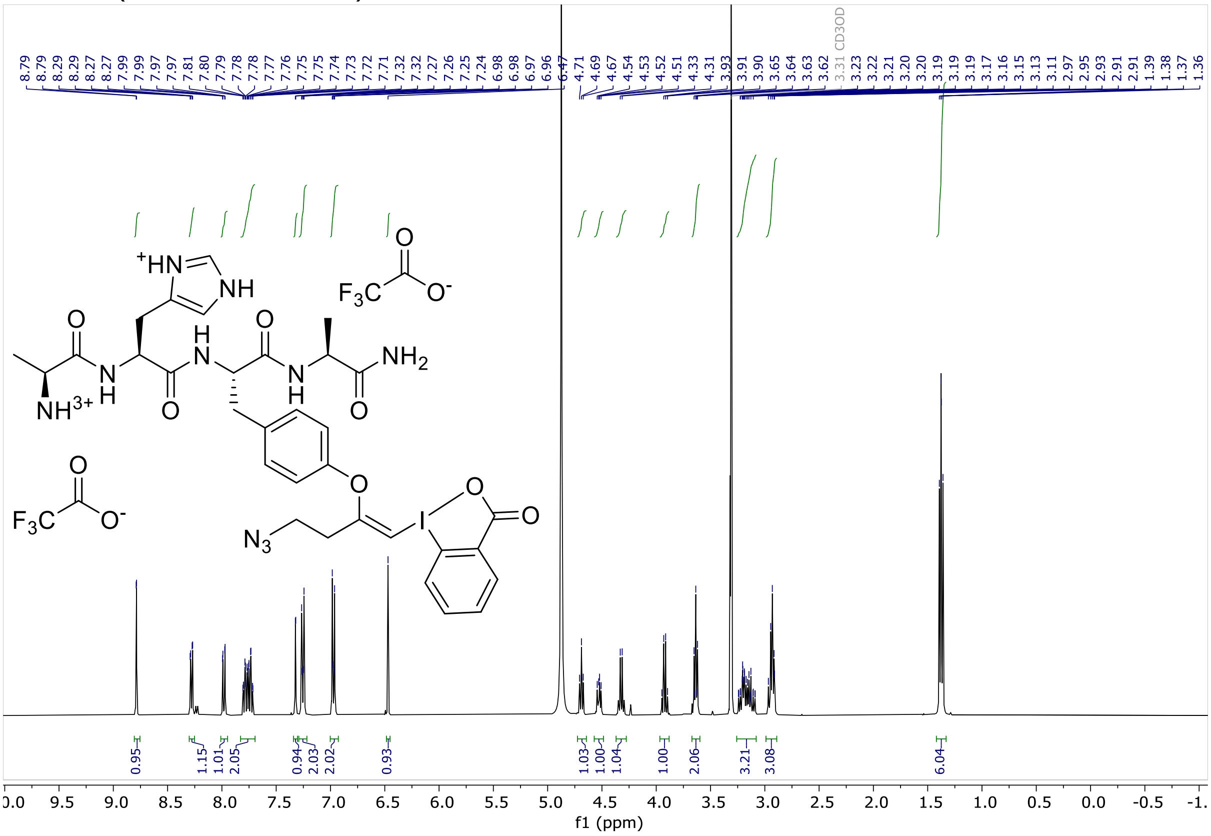

^{13}C NMR (101 MHz, CD_3OD)

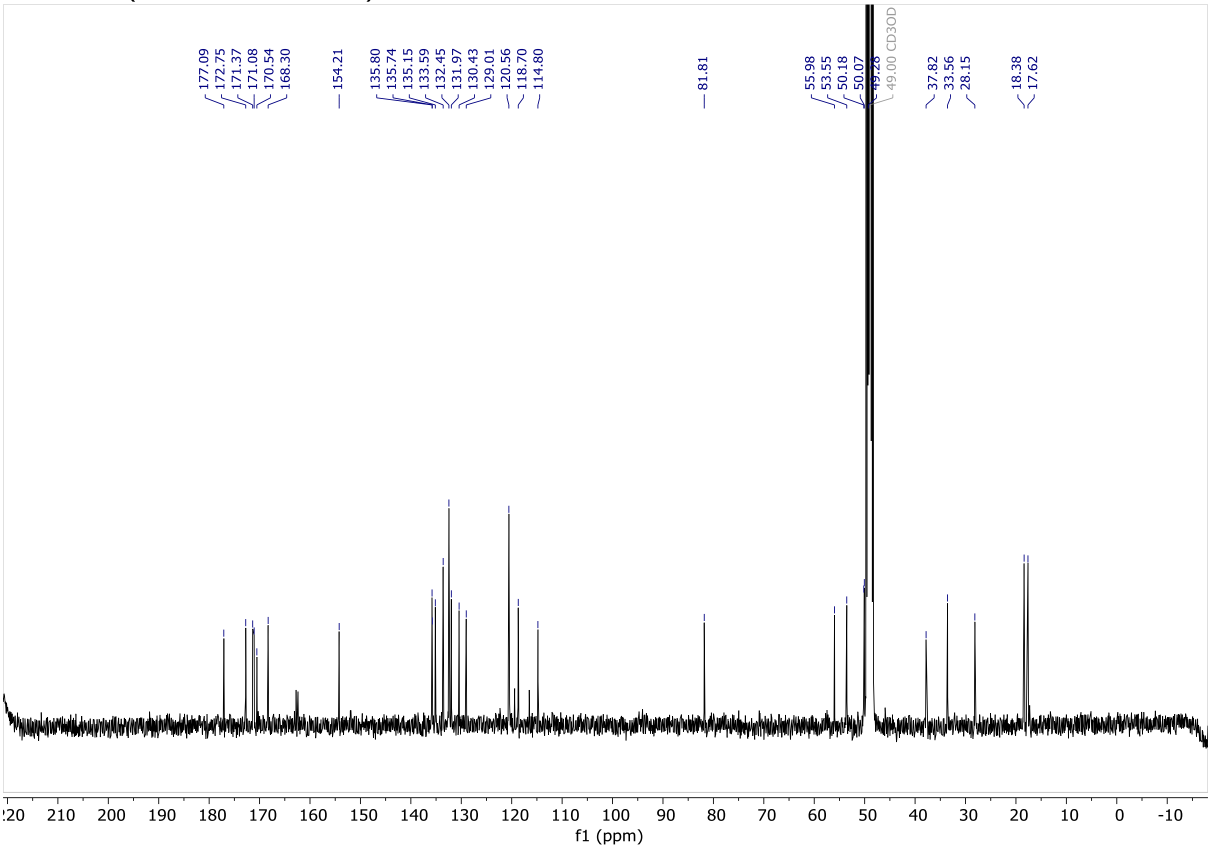

O-VBX AKYA-NH2 (3f)

^{1}H NMR (400 MHz, CD_3OD)

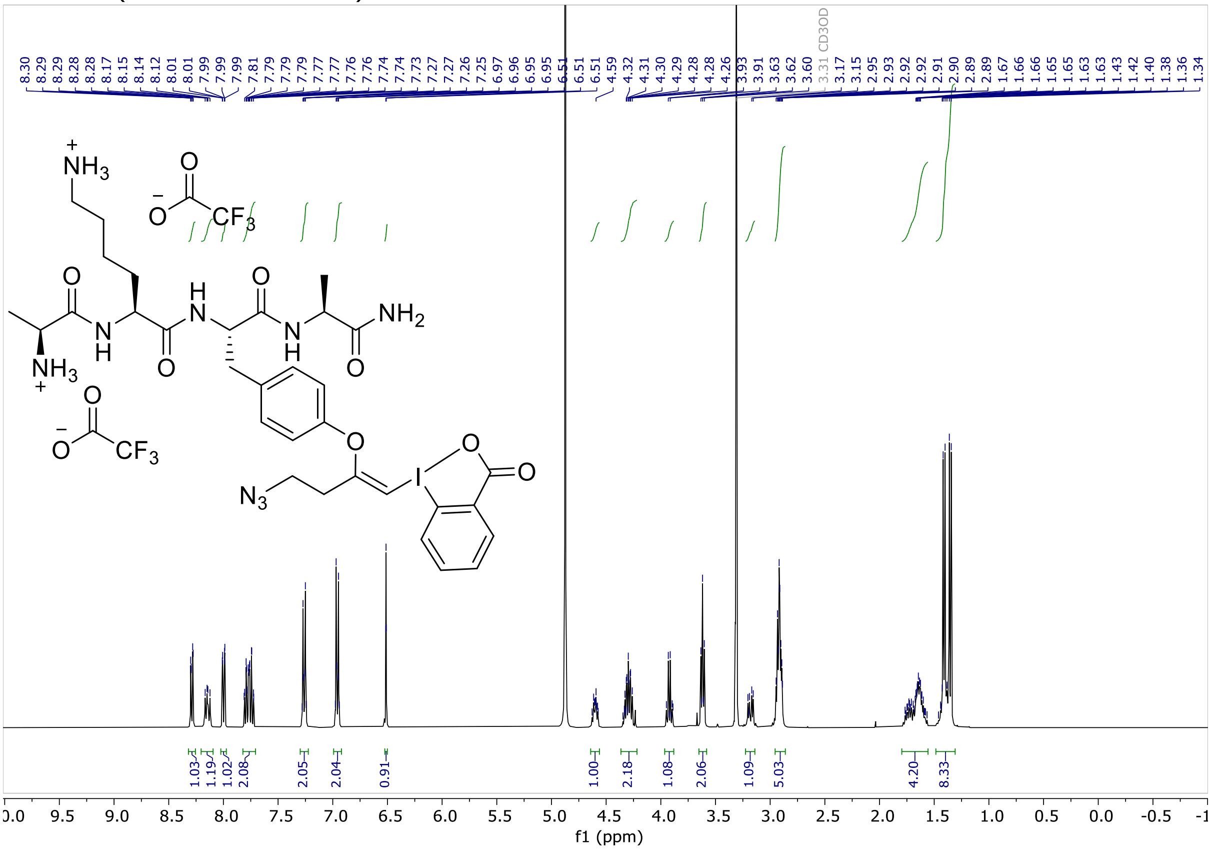

^{13}C NMR (101 MHz, CD_3OD)

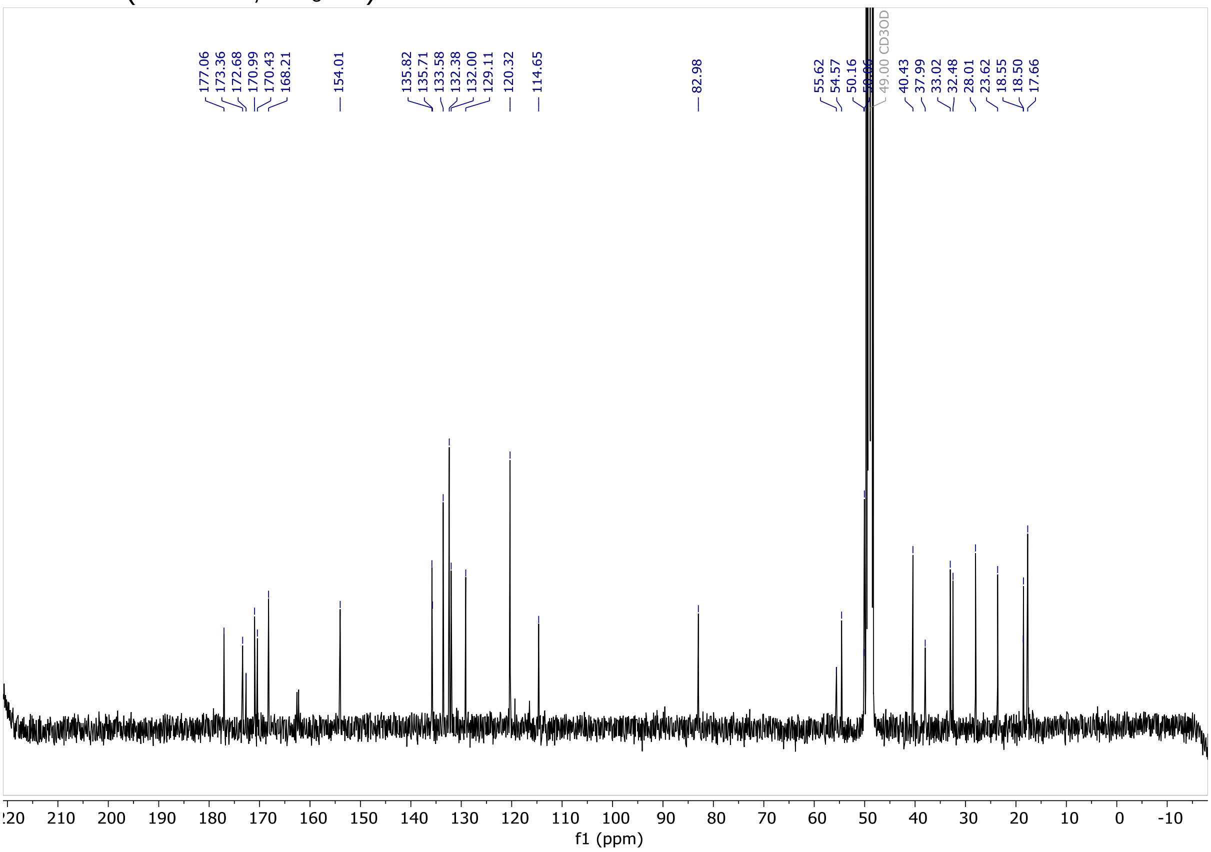

O-VBX ACYA-NH$_2$ (3g)

^{1}H NMR (400 MHz, CD_3OD)

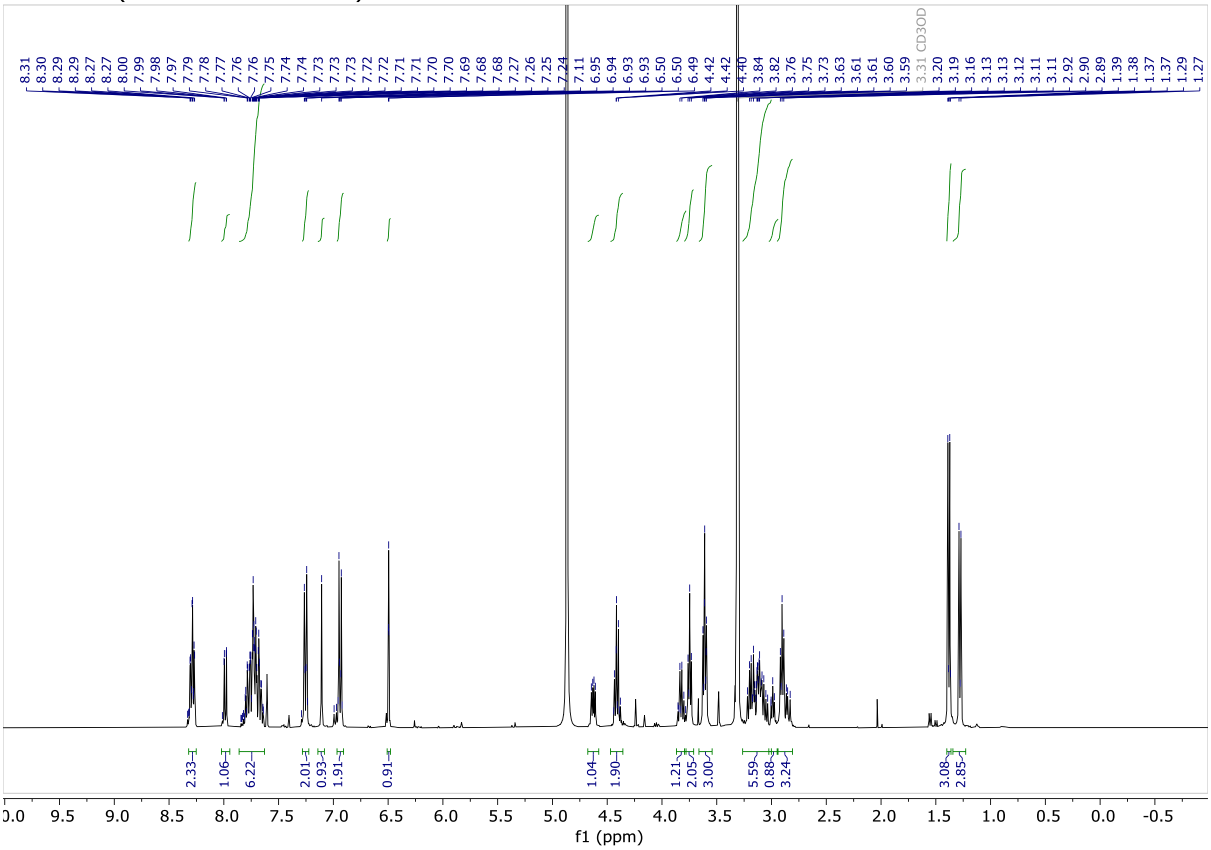

^{13}C NMR (101 MHz, CD_3OD)

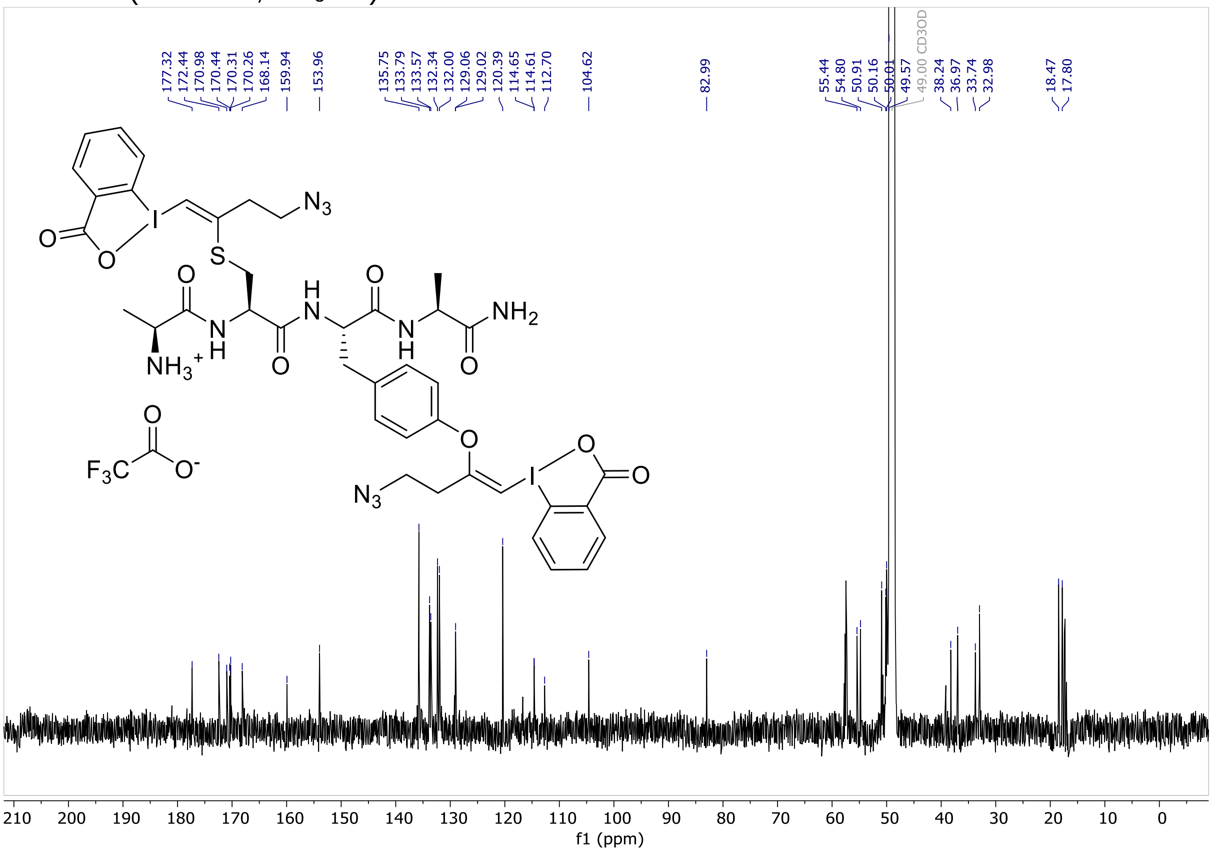

O-VBX ASYA-NH₂ (3h)

^{1}H NMR (400 MHz, CD_3OD)

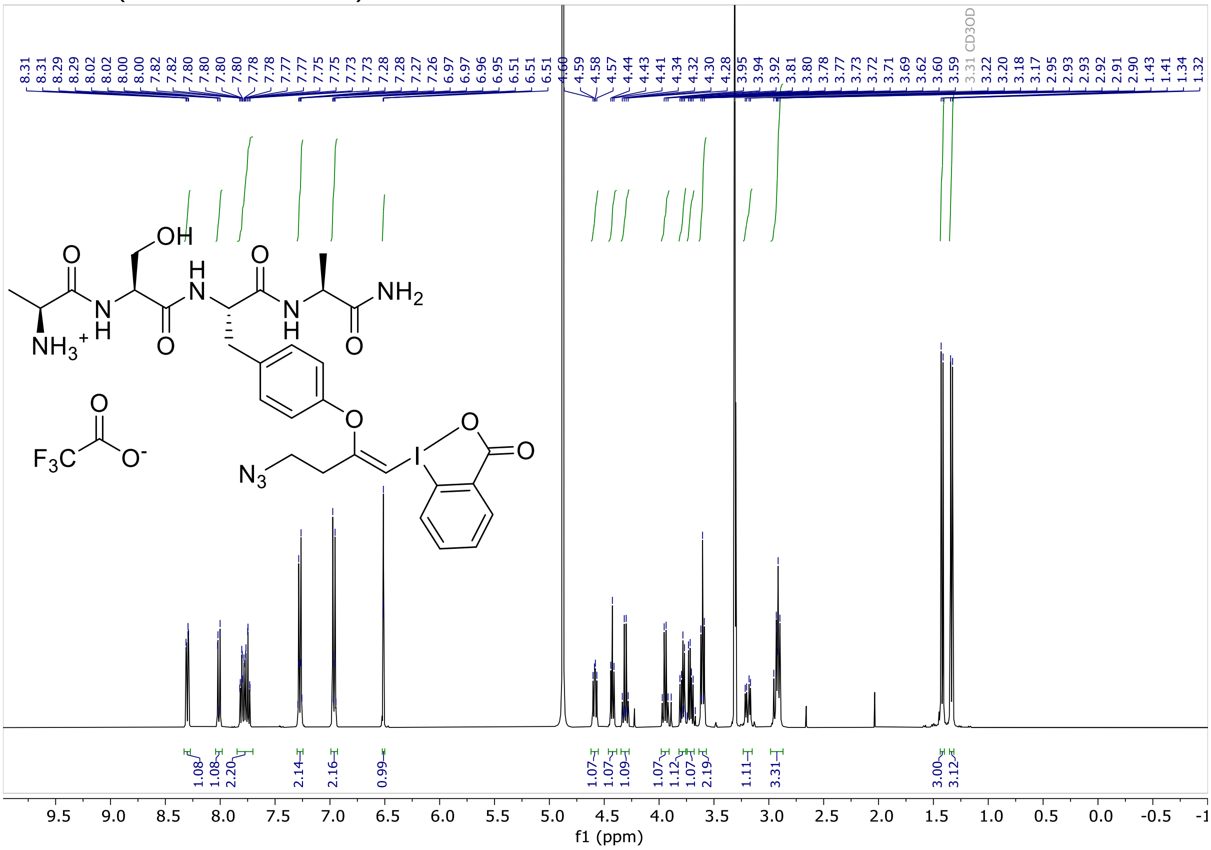

^{13}C NMR (101 MHz, CD_3OD)

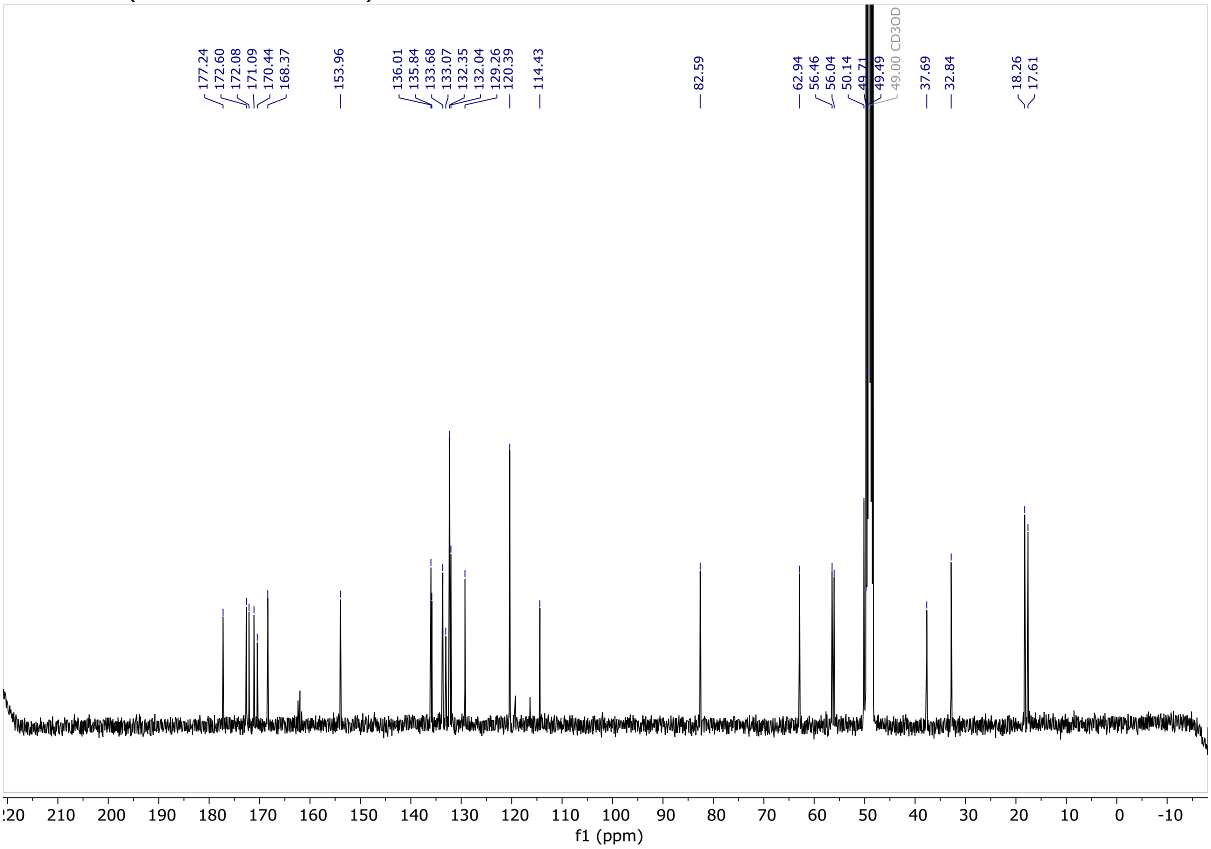

O-VBX AMYA-NH₂ (3i)

^{1}H NMR (400 MHz, CD_3OD)

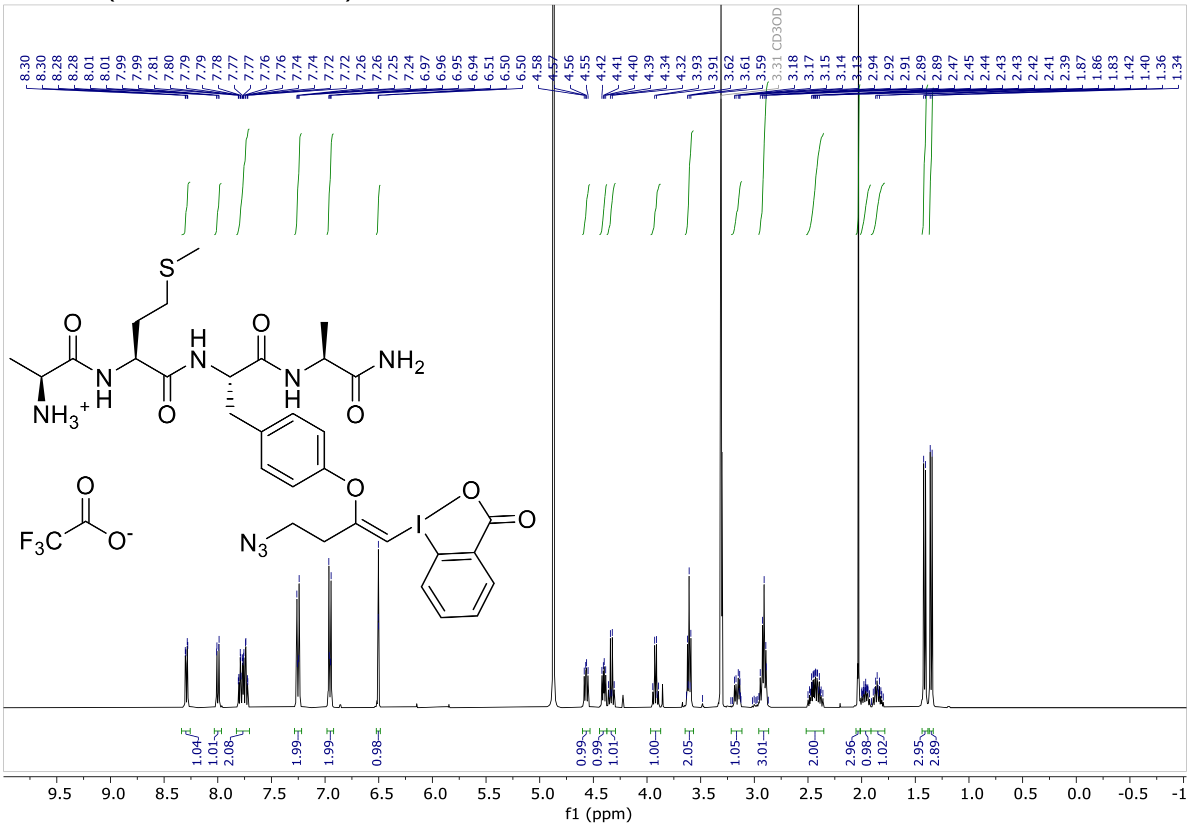

^{13}C NMR (101 MHz, CD_3OD)

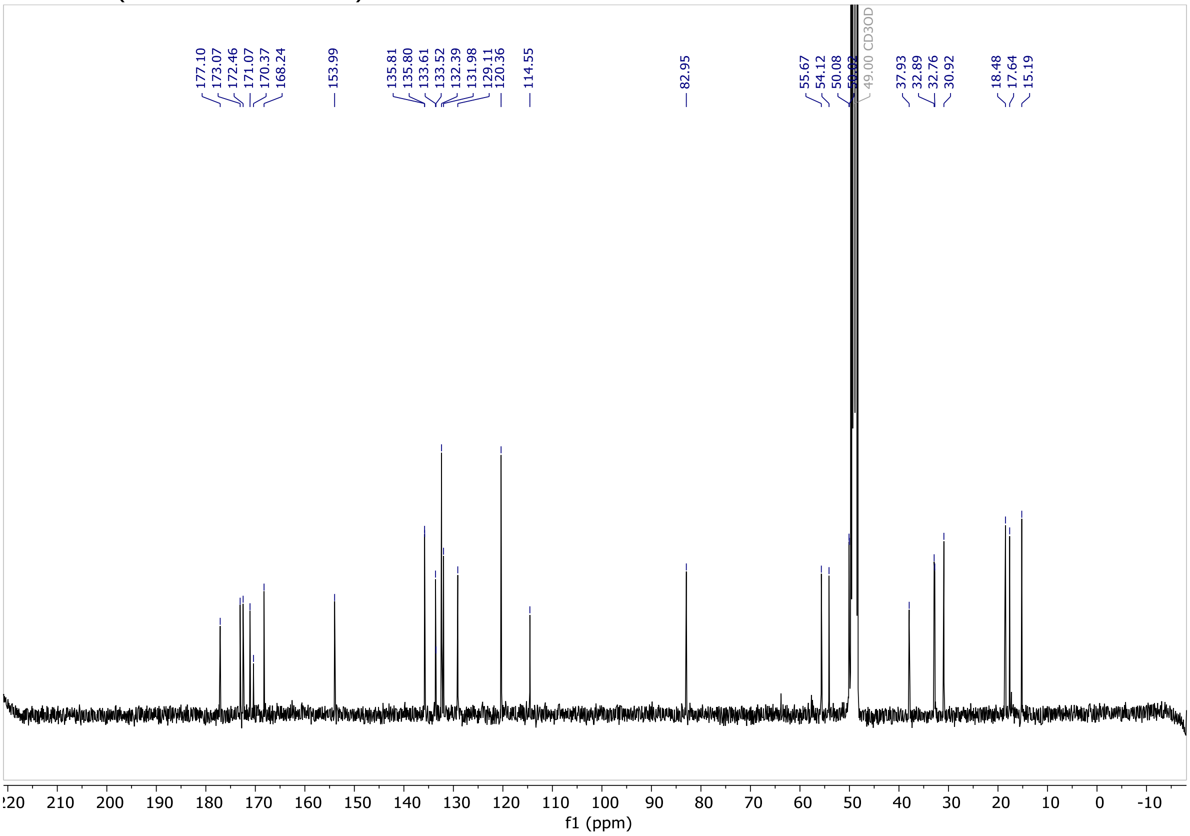

O-VBX ADYA-NH_2 (3j)

^{1}H NMR (400 MHz, CD_3OD)

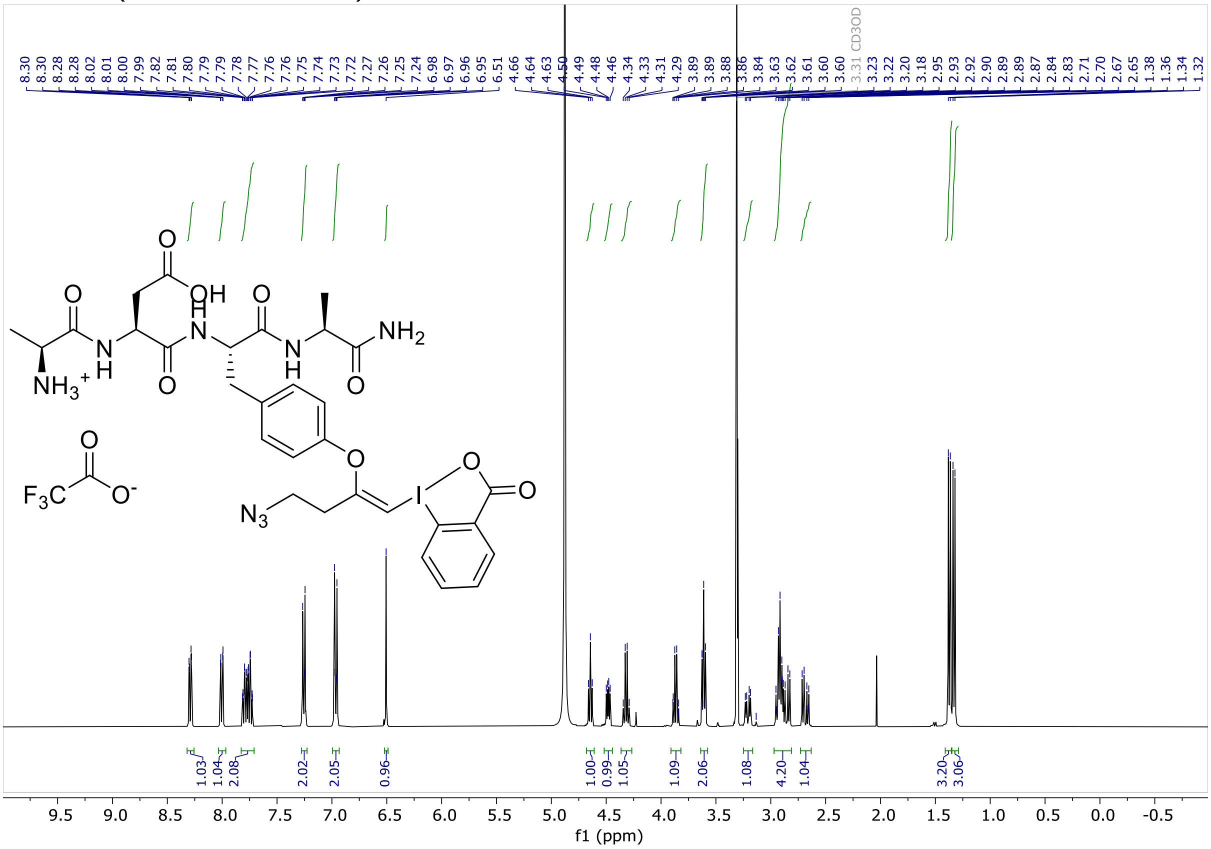

^{13}C NMR (101 MHz, CD_3OD)

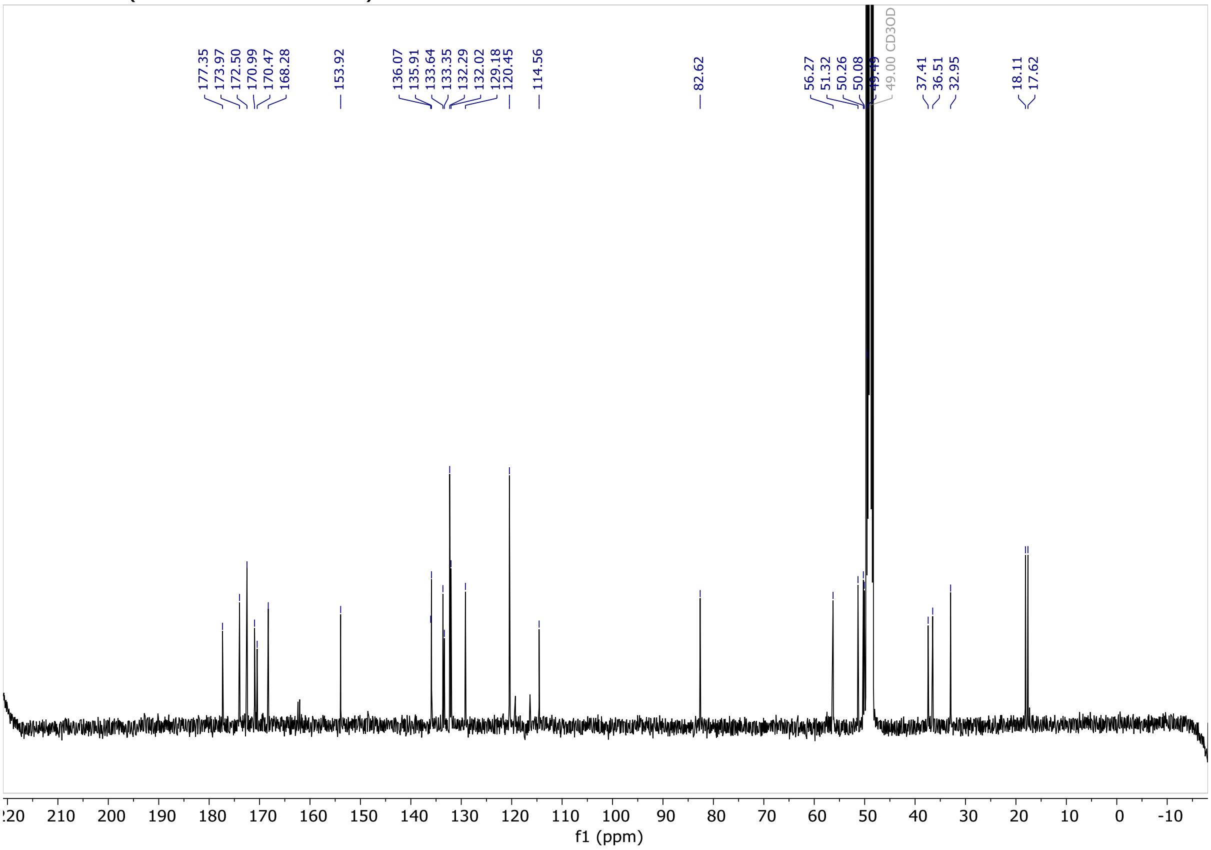

O-VBX ANYA-NH₂ (3k)

^{1}H NMR (400 MHz, CD_3OD)

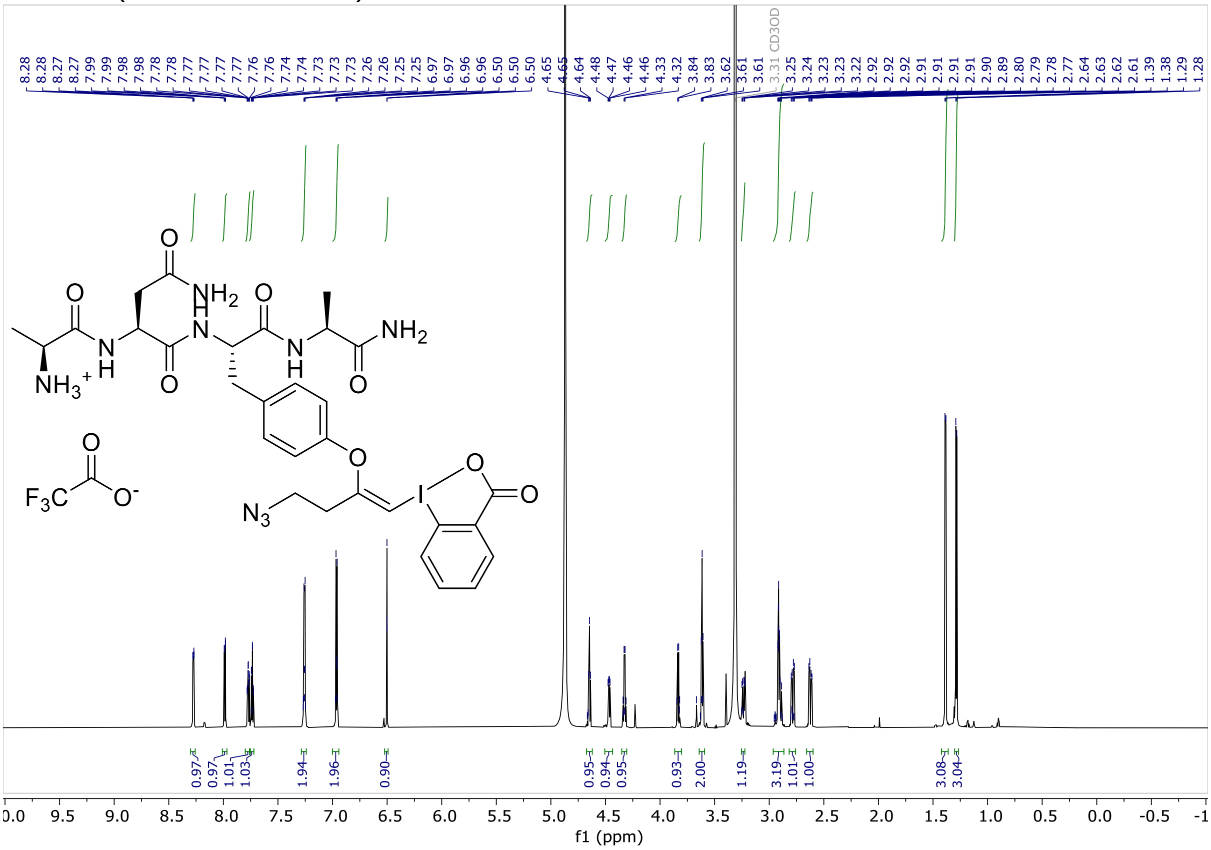

^{13}C NMR (101 MHz, CD_3OD)

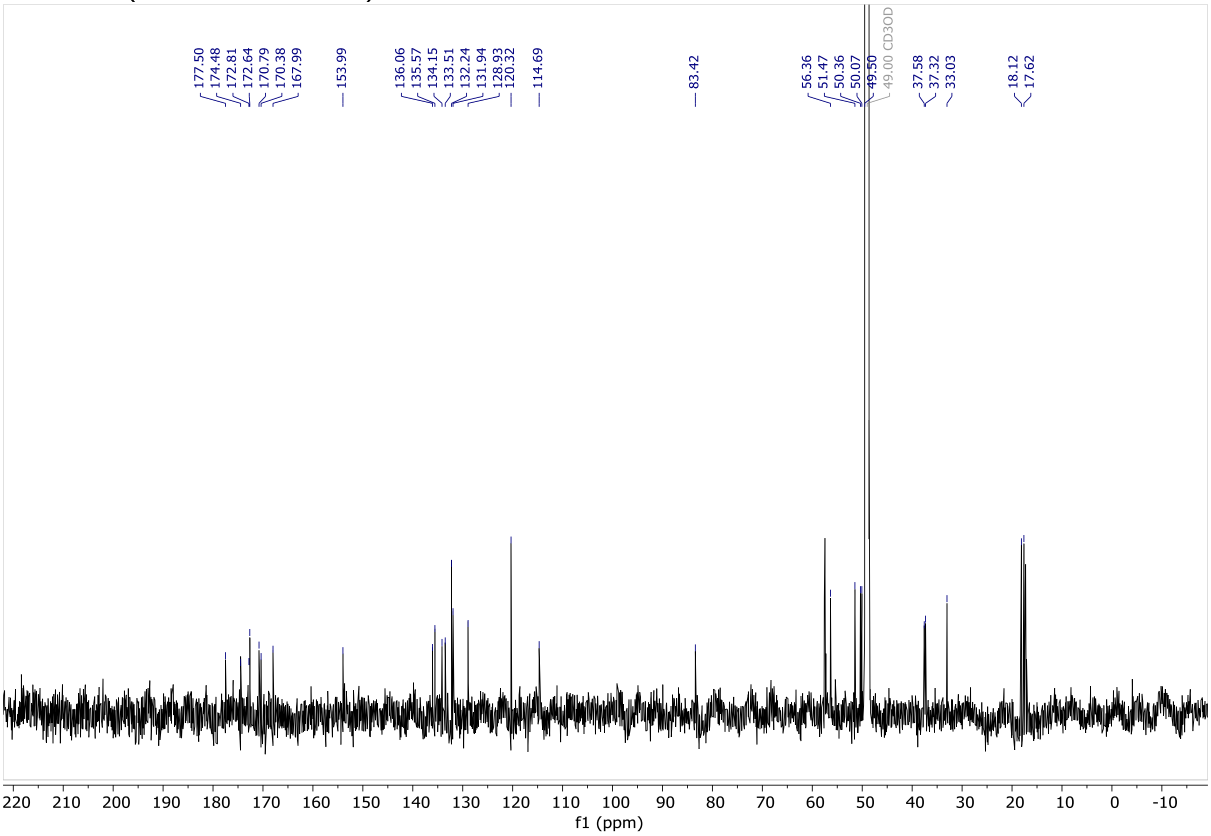

O-VBX AYLA-NH$_2$ (3l)

^{1}H NMR (400 MHz, CD_3OD)

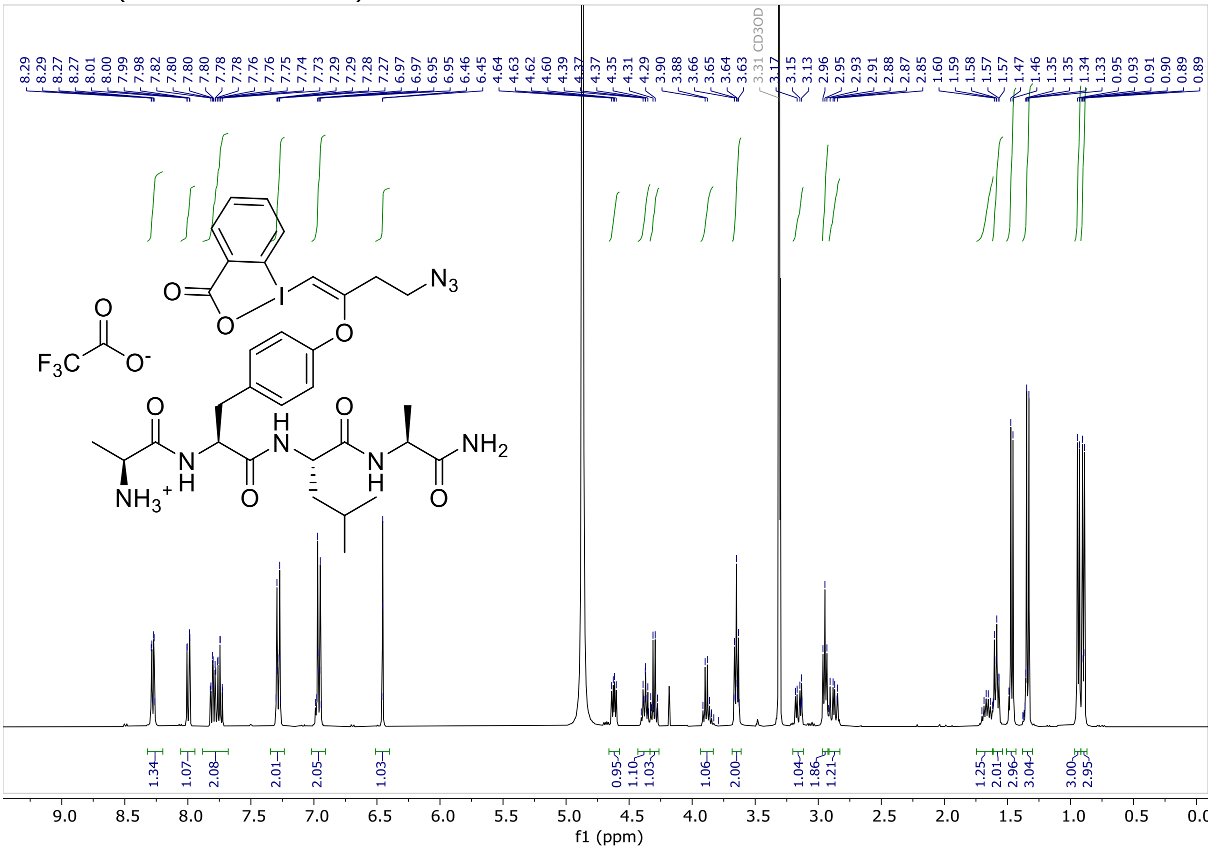

^{13}C NMR (101 MHz, CD_3OD)

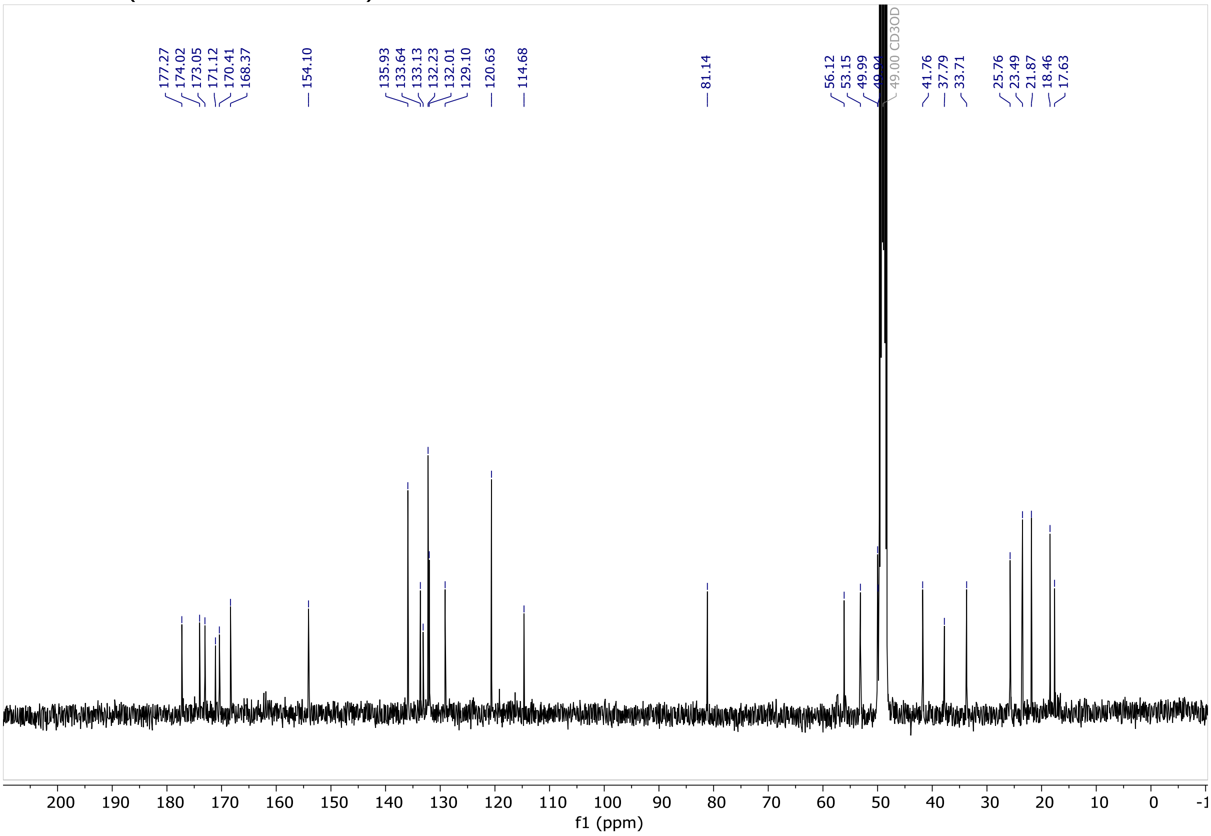

O-VBX YALA-NH₂ (3m)

^1H NMR (400 MHz, CD_3OD)

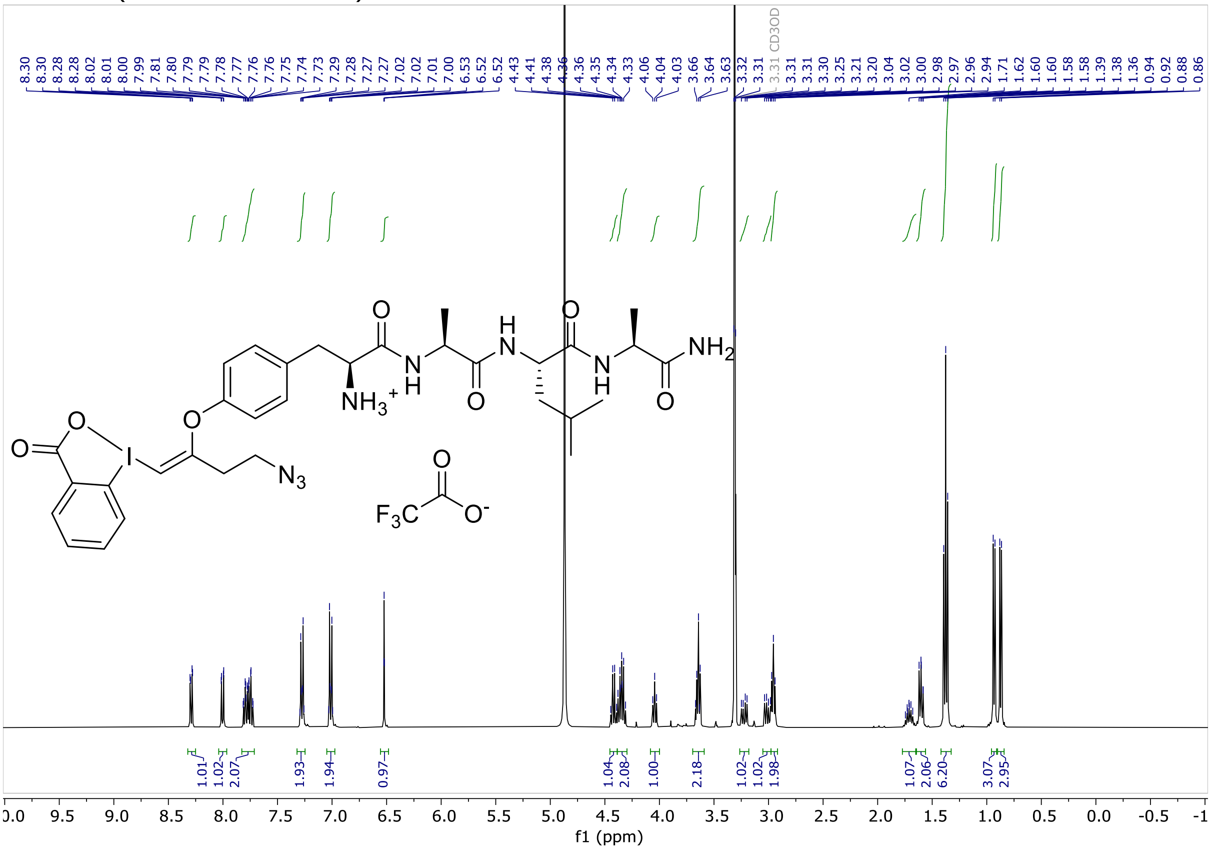

^{13}C NMR (101 MHz, CD_3OD)

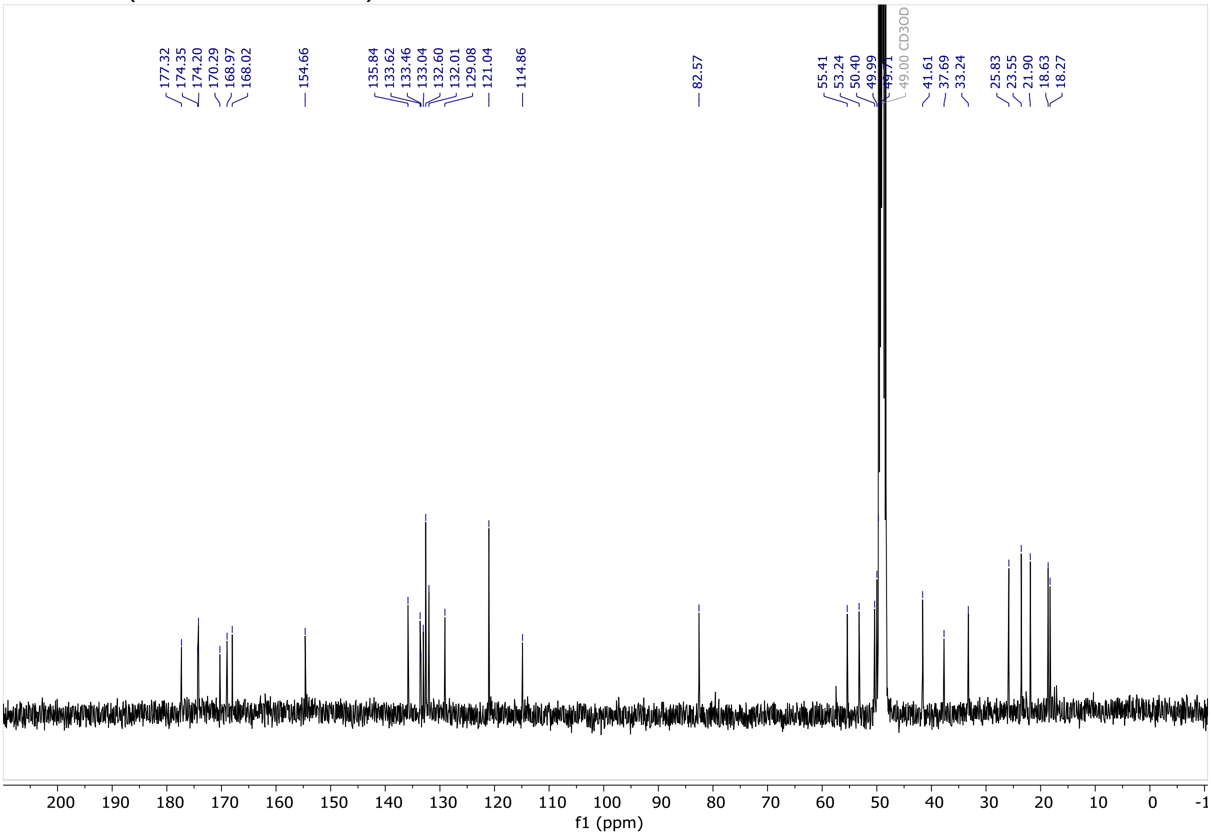

O-VBX Ac-AFGY (3n)

^{1}H NMR (400 MHz, CD_3OD)

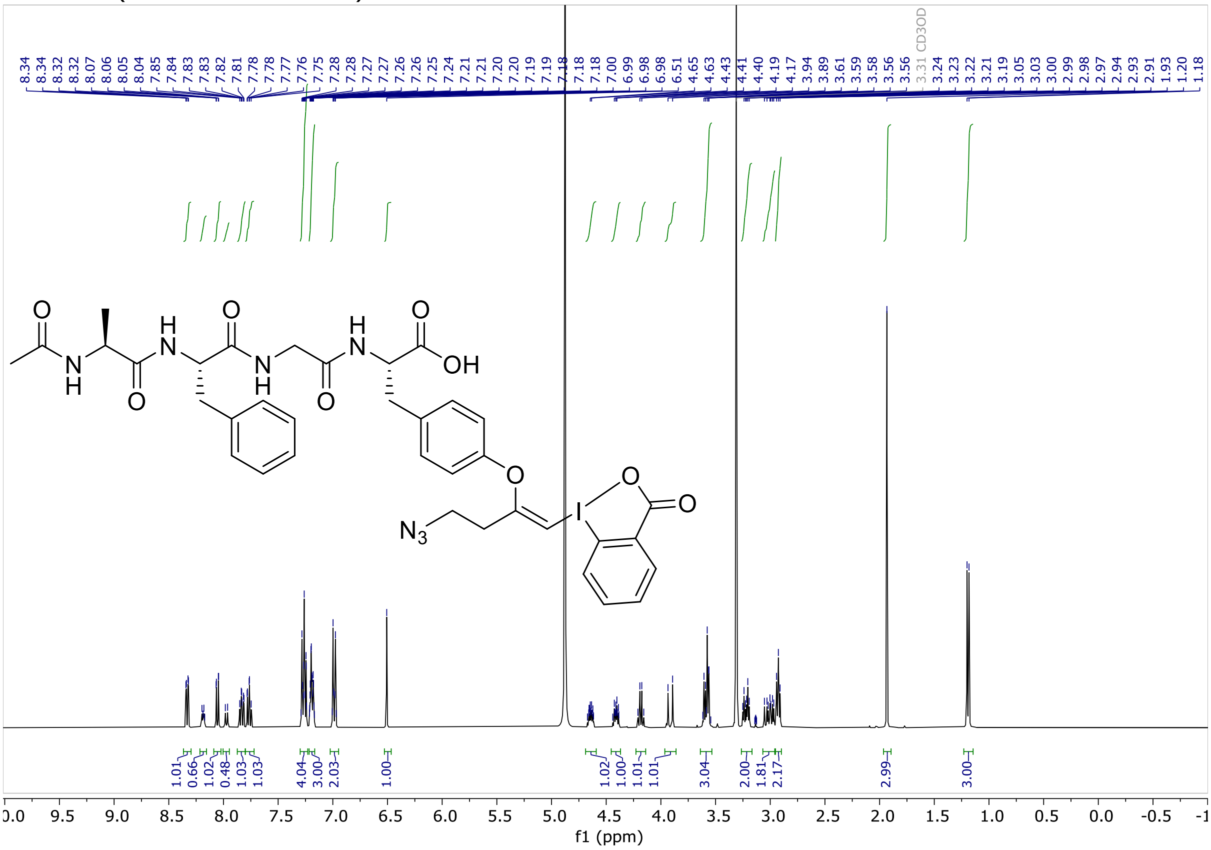

^{13}C NMR (101 MHz, CD_3OD)

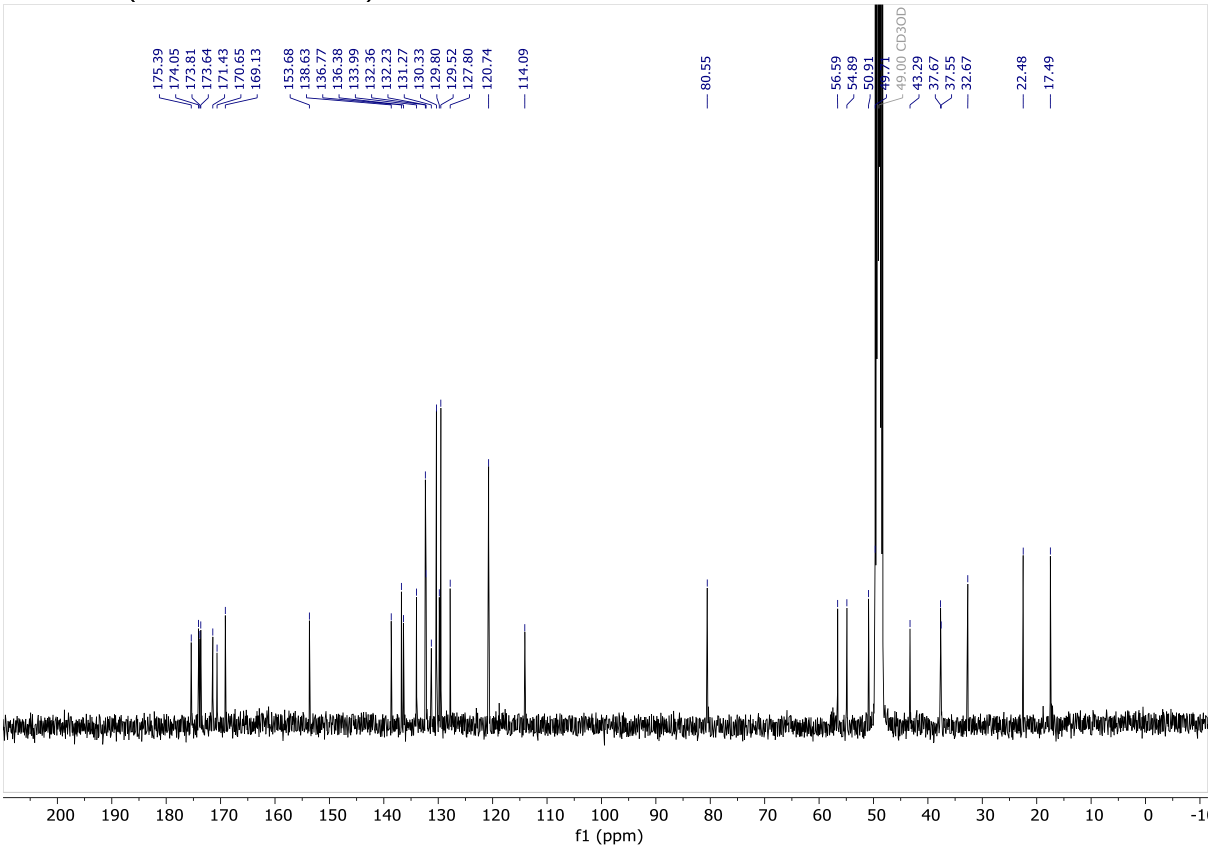

BCN (9d)

^{1}H NMR (400 MHz, CD_3OD)

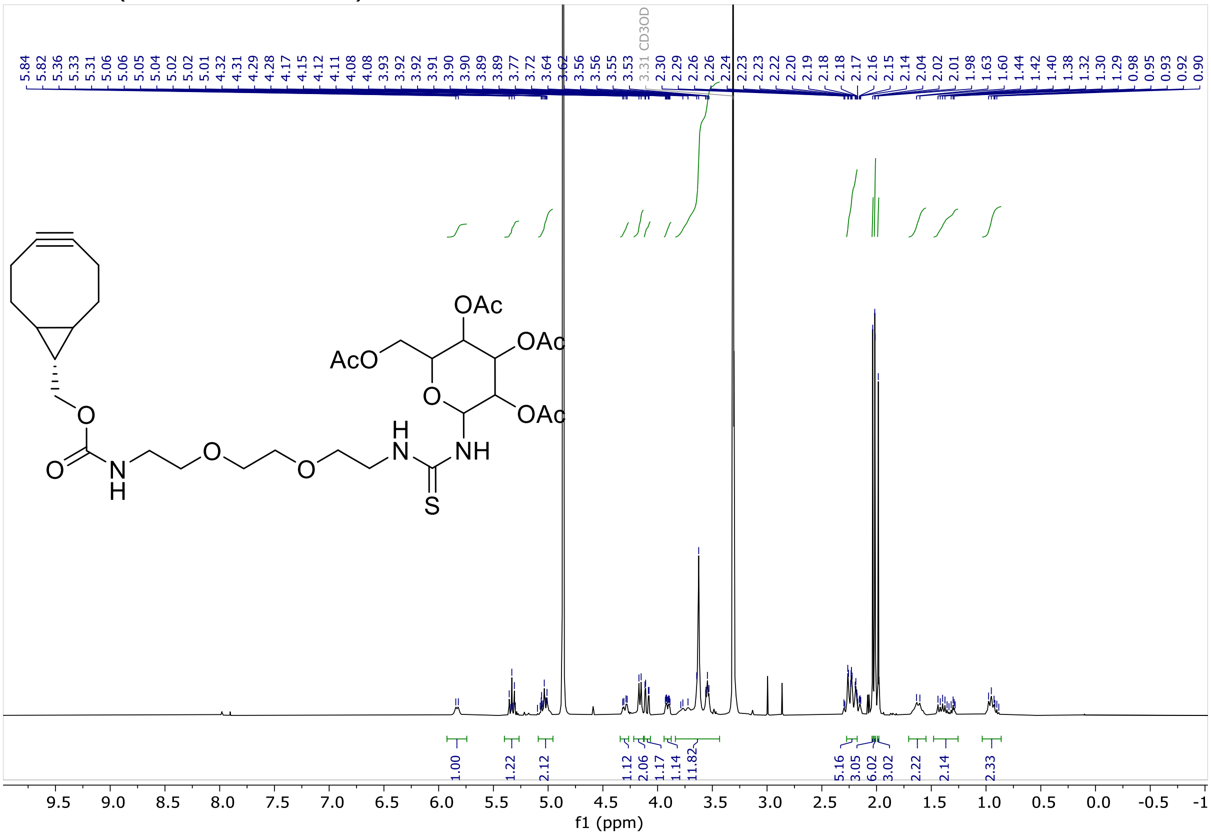

^{13}C NMR (101 MHz, CD_3OD)

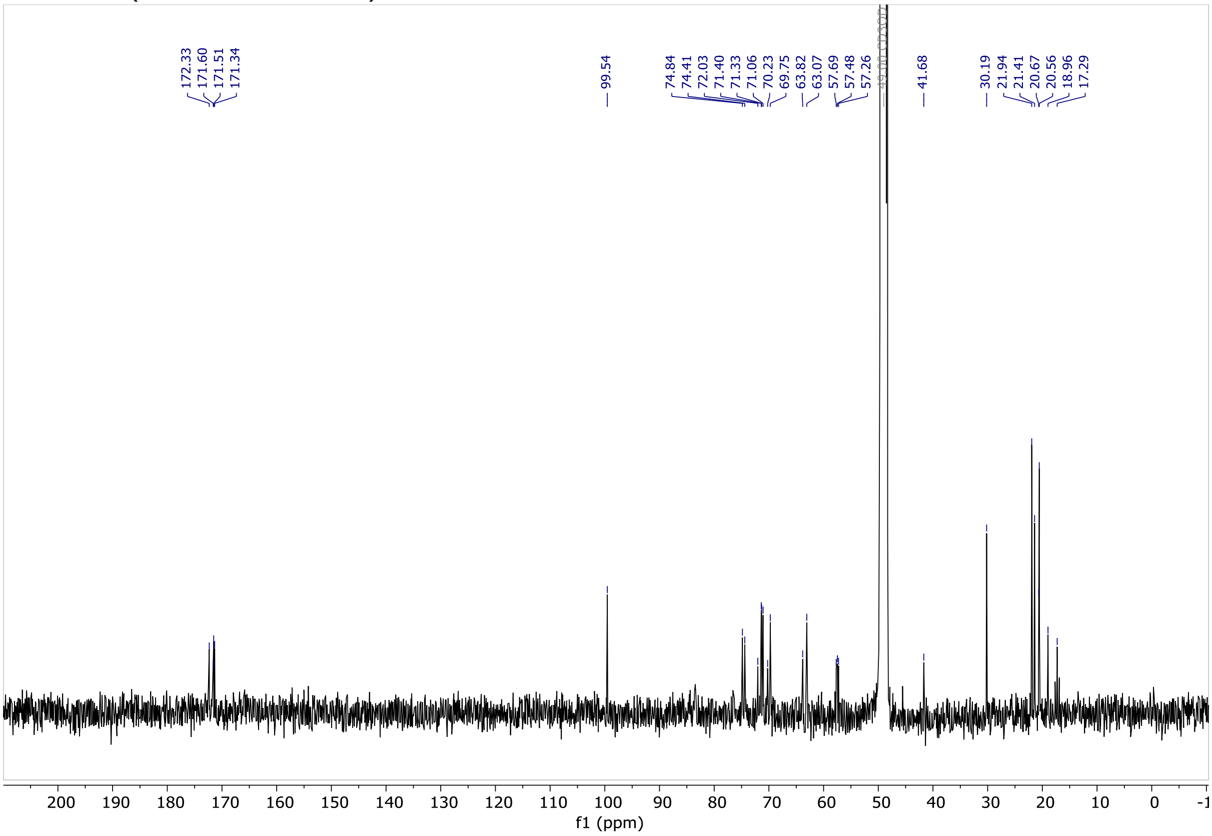

SPAAC (10a)

^{1}H NMR (400 MHz, CD_3OD)

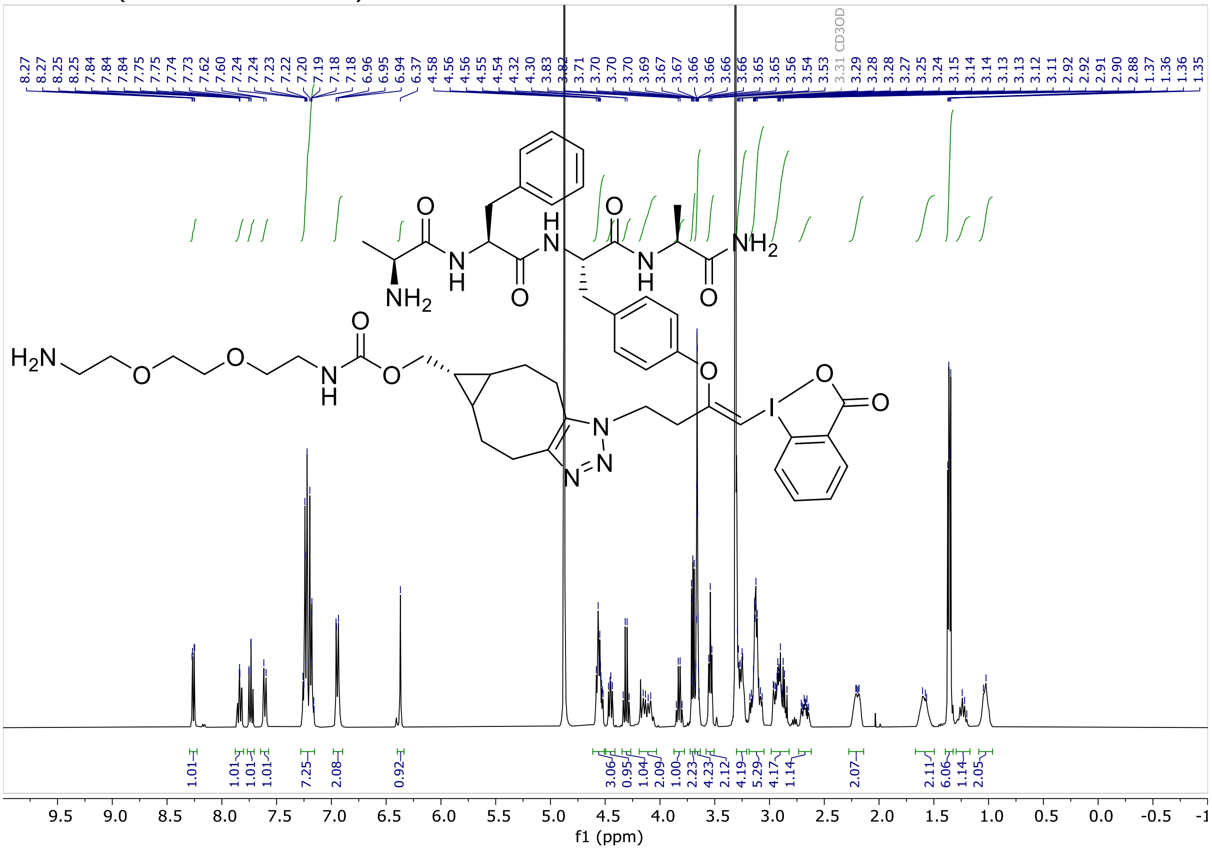

^{13}C NMR (101 MHz, CD_3OD)

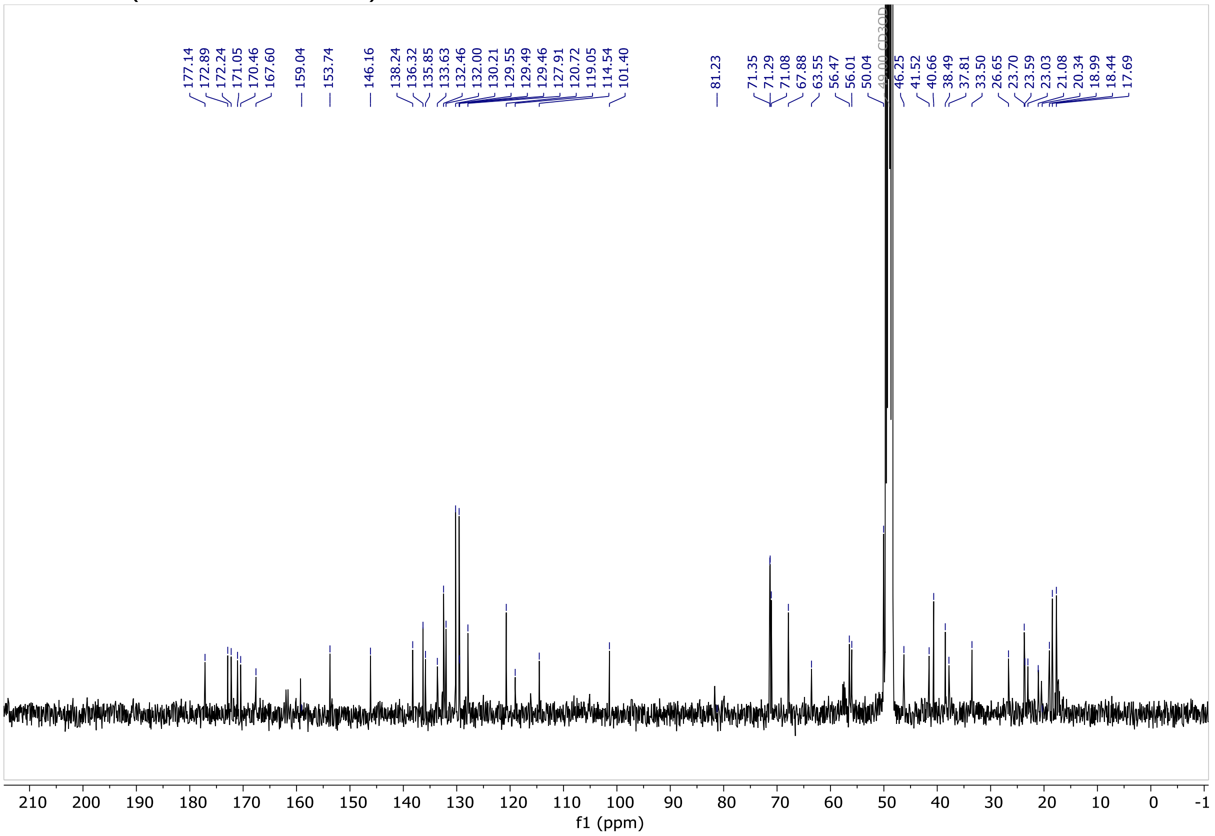

Suzuki (15a)

^{1}H NMR (400 MHz, CD_3OD)

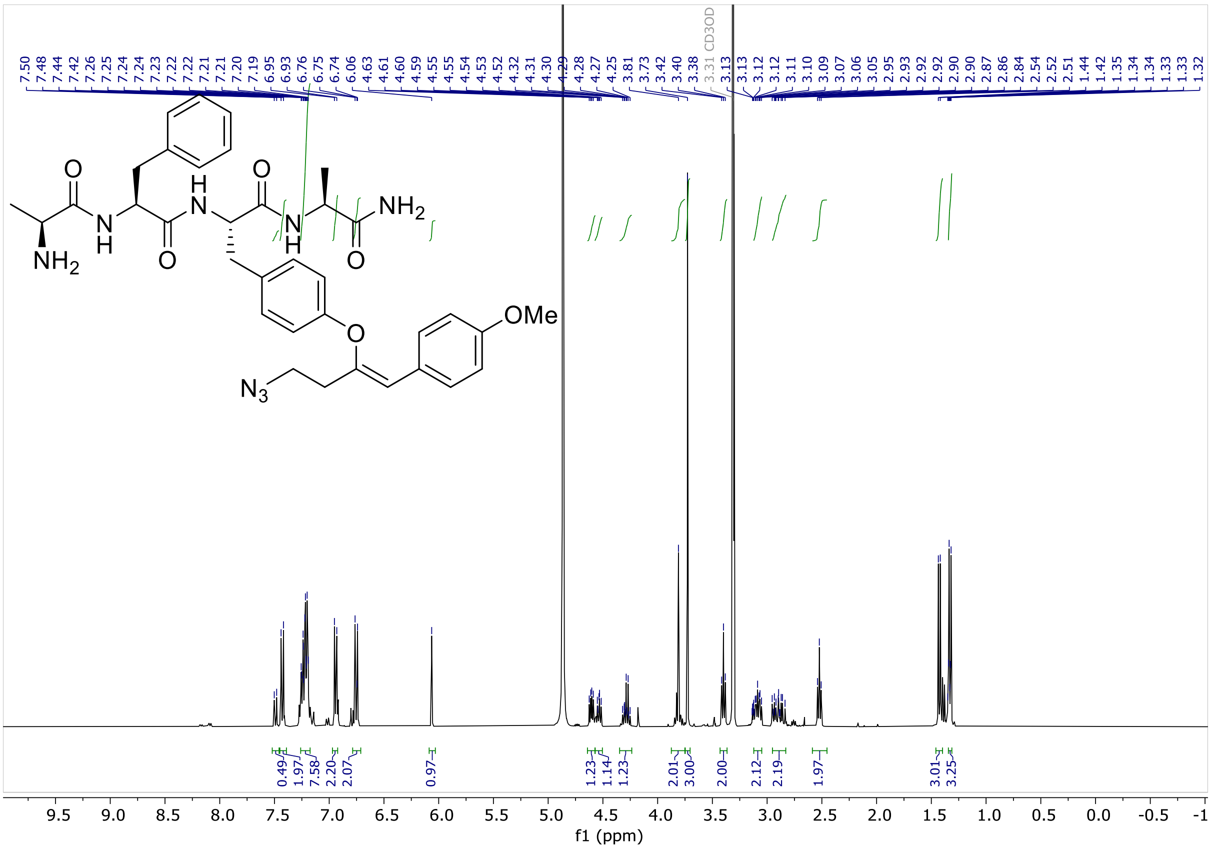

^{13}C NMR (101 MHz, CD_3OD)

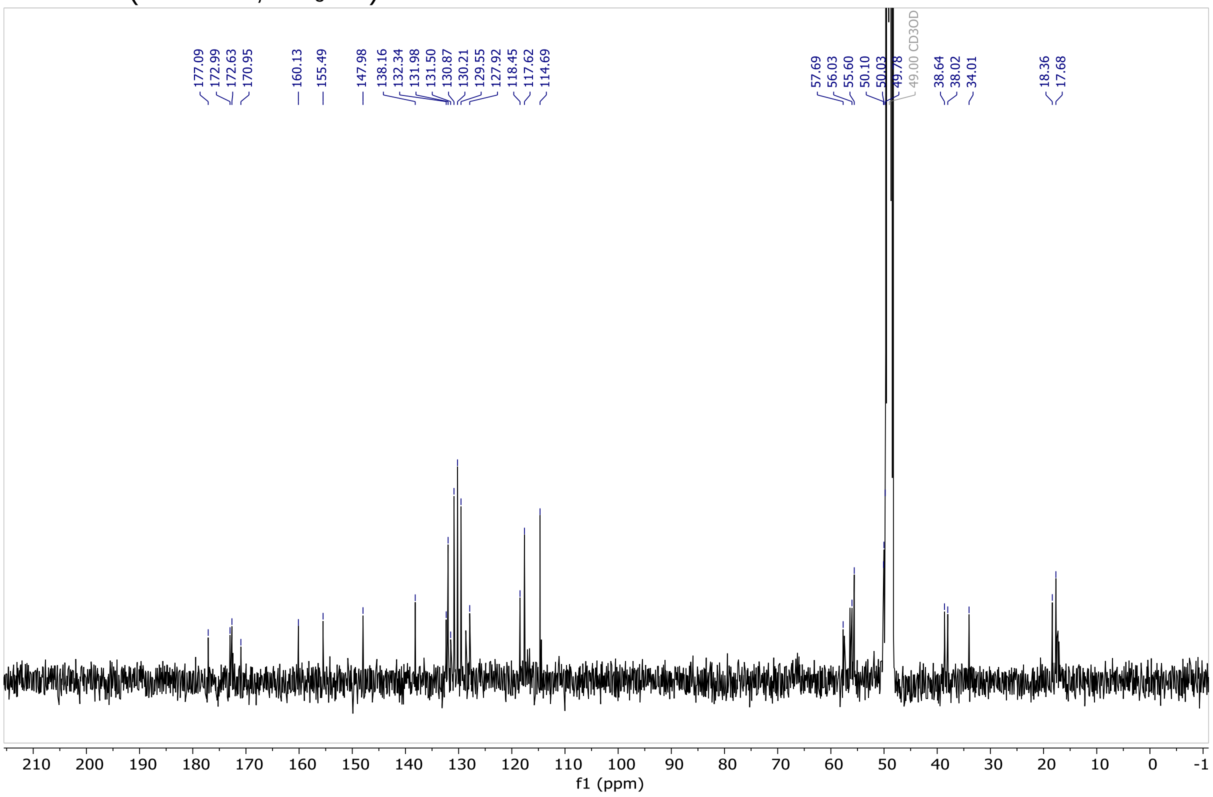

Transporter (S32)

^{1}H NMR (300 MHz, $CDCl_3$)

JM-05-69-FULL.10.fid
MP_PROTON CDCl3 /opt/topspin3.6.2/data/matile maynard 14

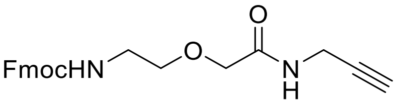

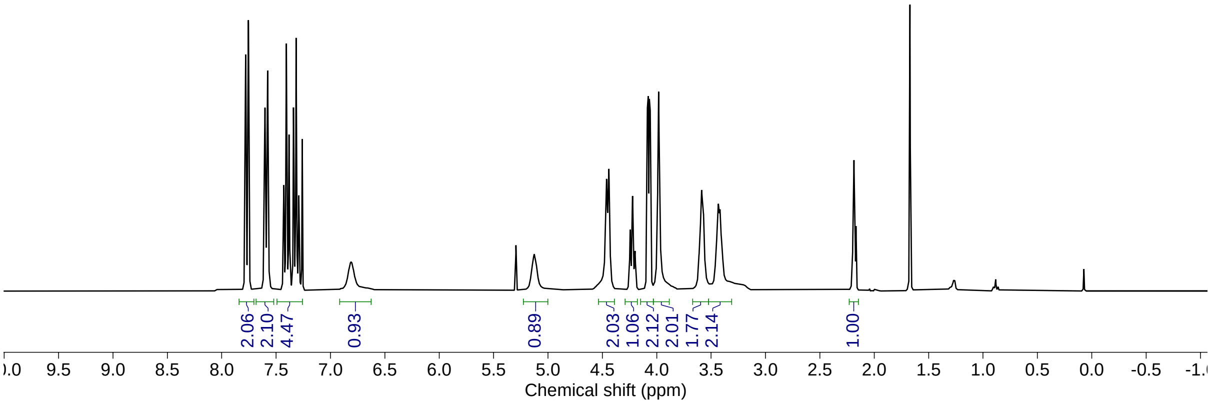

^{13}C NMR (75 MHz, $CDCl_3$)

JM-05-69-FULL.11.fid
C13CPD CDCl3 /opt/topspin3.6.2/data/matile maynard 14

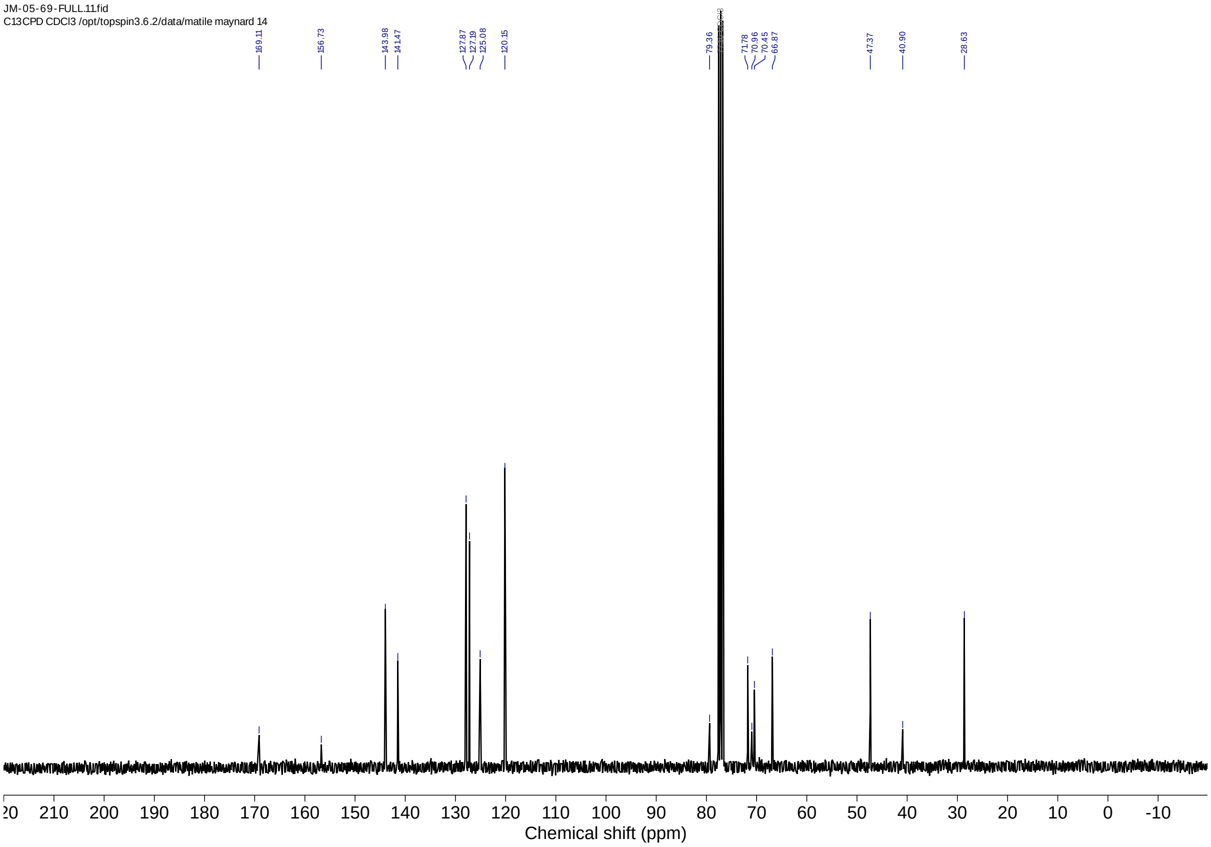

Transporter (S33)

^{1}H NMR (300 MHz, CD_3OD)

JM-05-71-FULL.10.fid
MP_PROTON MeOD /opt/topspin3.6.2/data/matile maynard 18

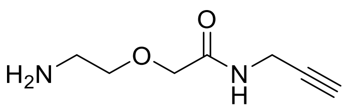

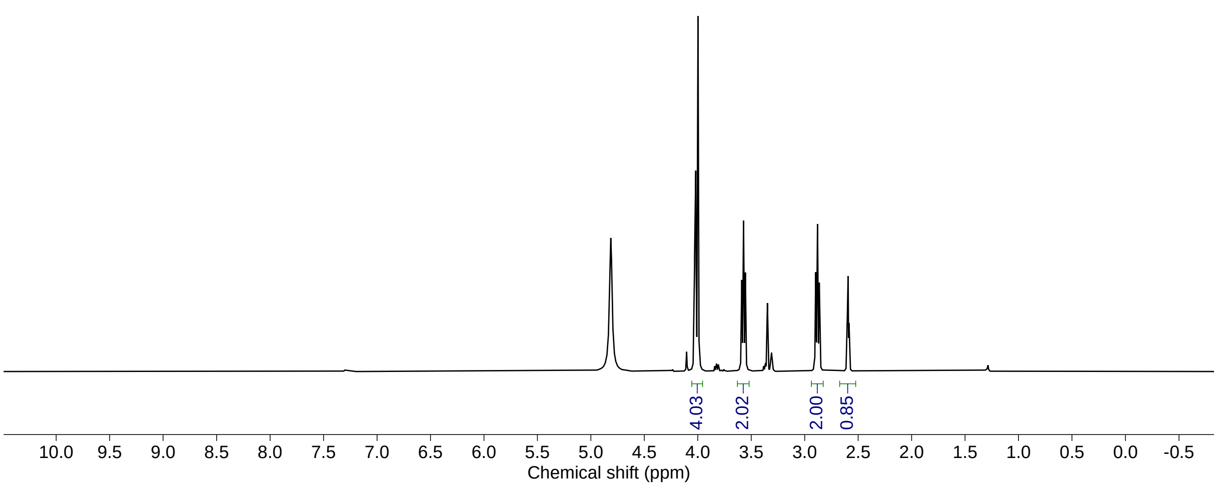

^{13}C NMR (75 MHz, CD_3OD)

JM-05-71-FULL.11.fid
C13CPD MeOD /opt/topspin3.6.2/data/matile maynard 18

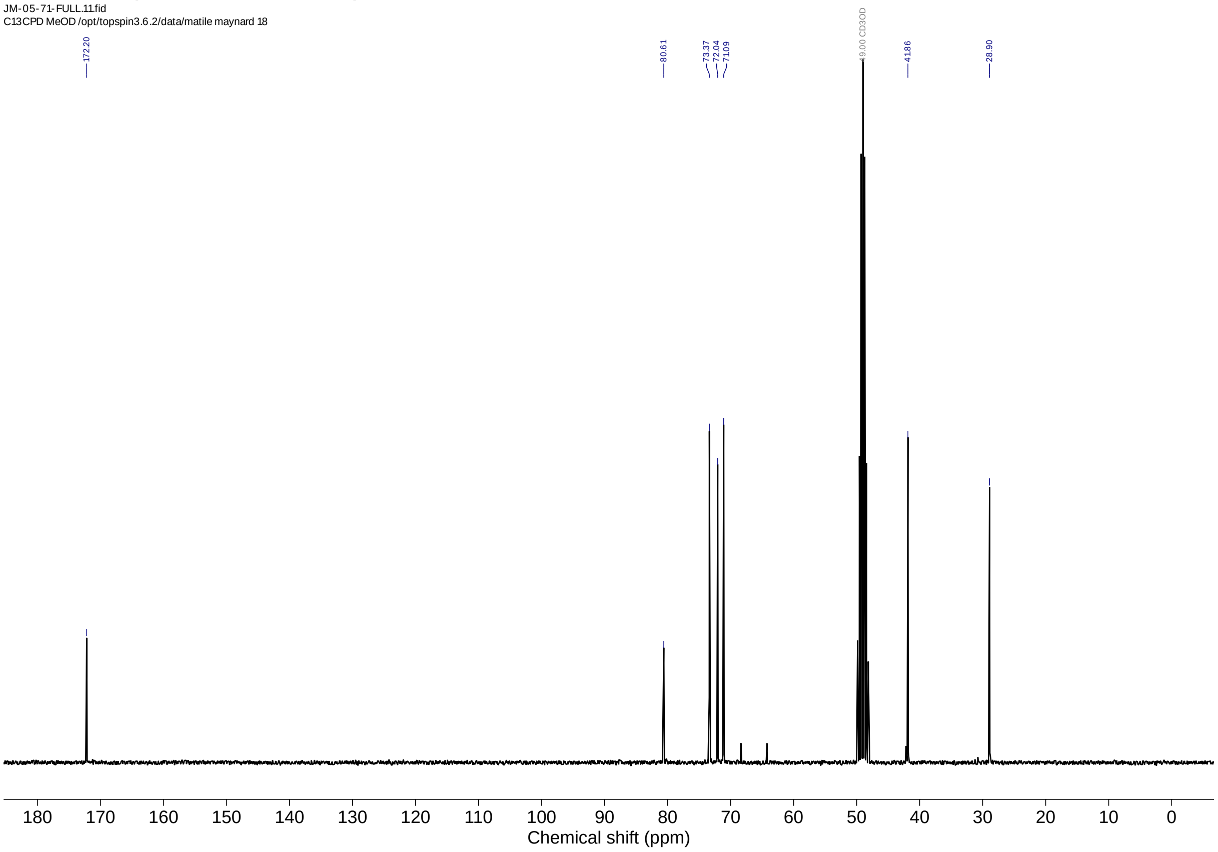

Transporter (S28)

^{1}H NMR (400 MHz, $CDCl_3$)

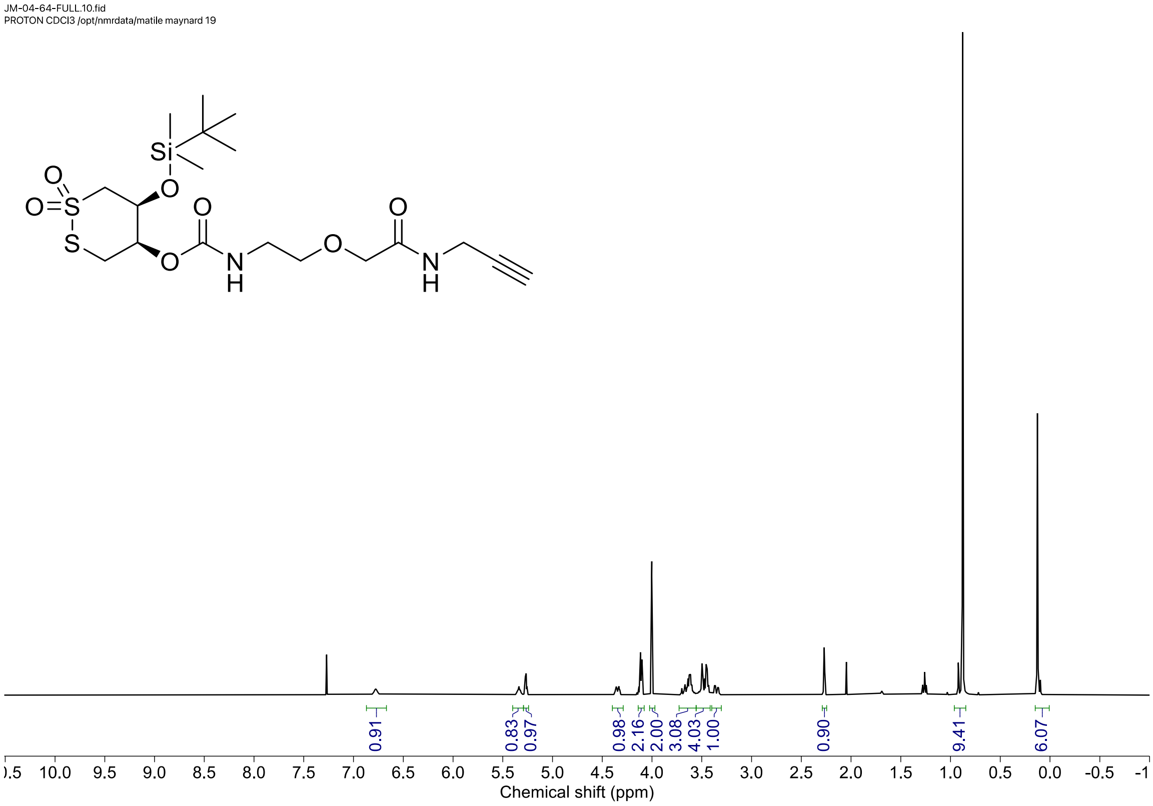

^{13}C NMR (101 MHz, $CDCl_3$)

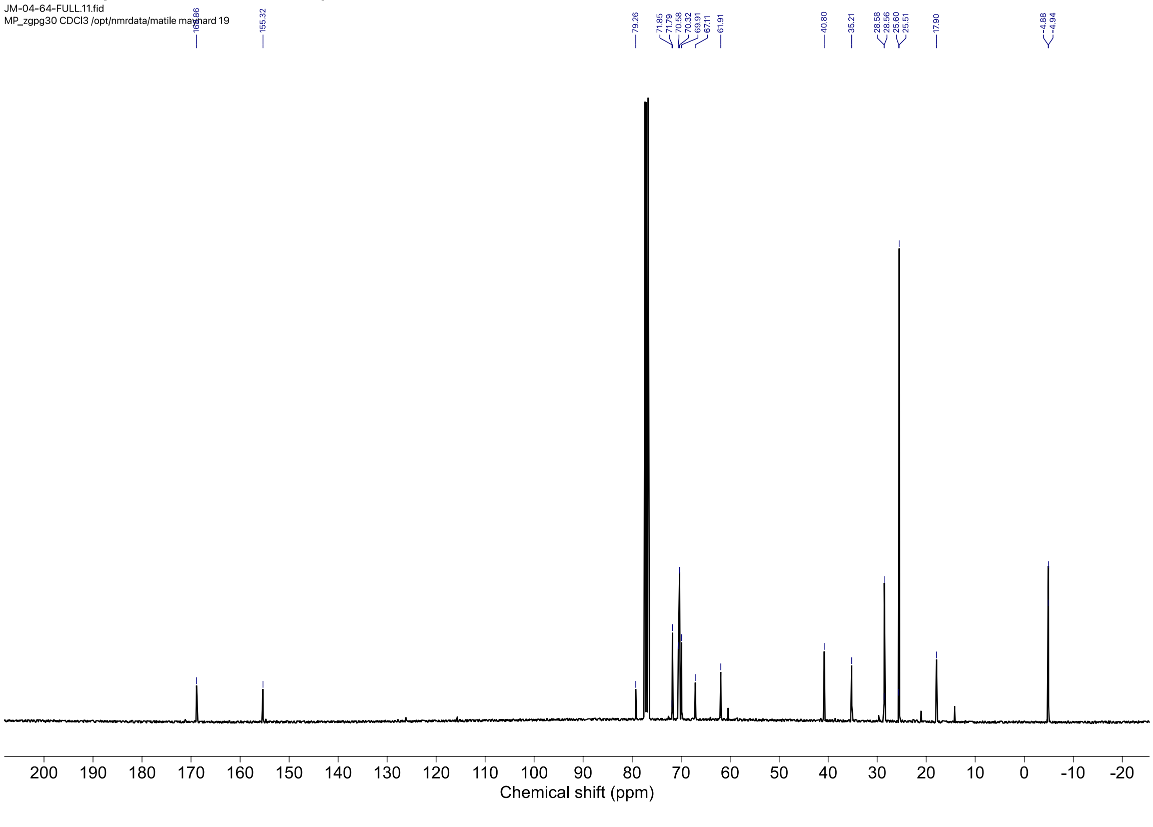

Transporter (S29)

^1H NMR (400 MHz, $CDCl_3$)

JM-04-15-FULL.10.fid
PROTON CDCl3 /opt/nmrdata/matile maynard 8

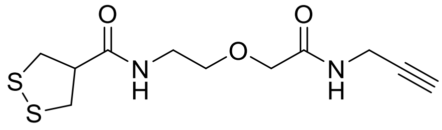

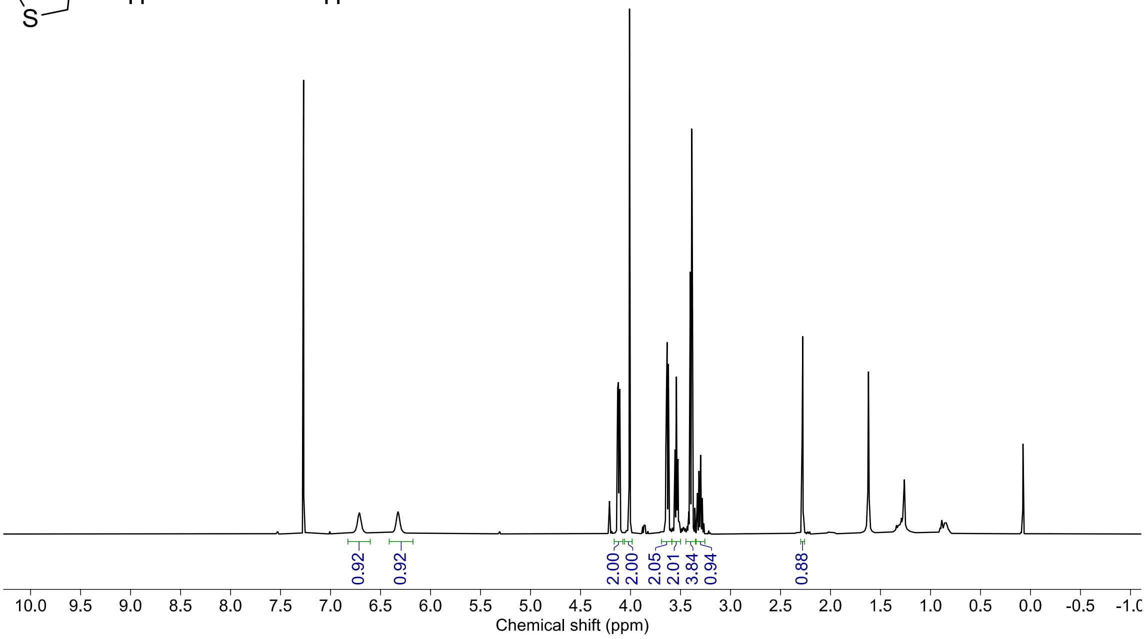

^{13}C NMR (101 MHz, $CDCl_3$)

JM-04-15-FULL.11.fid
MP_zgpg30 CDCl3 /opt/nmrdata/matile maynard 8

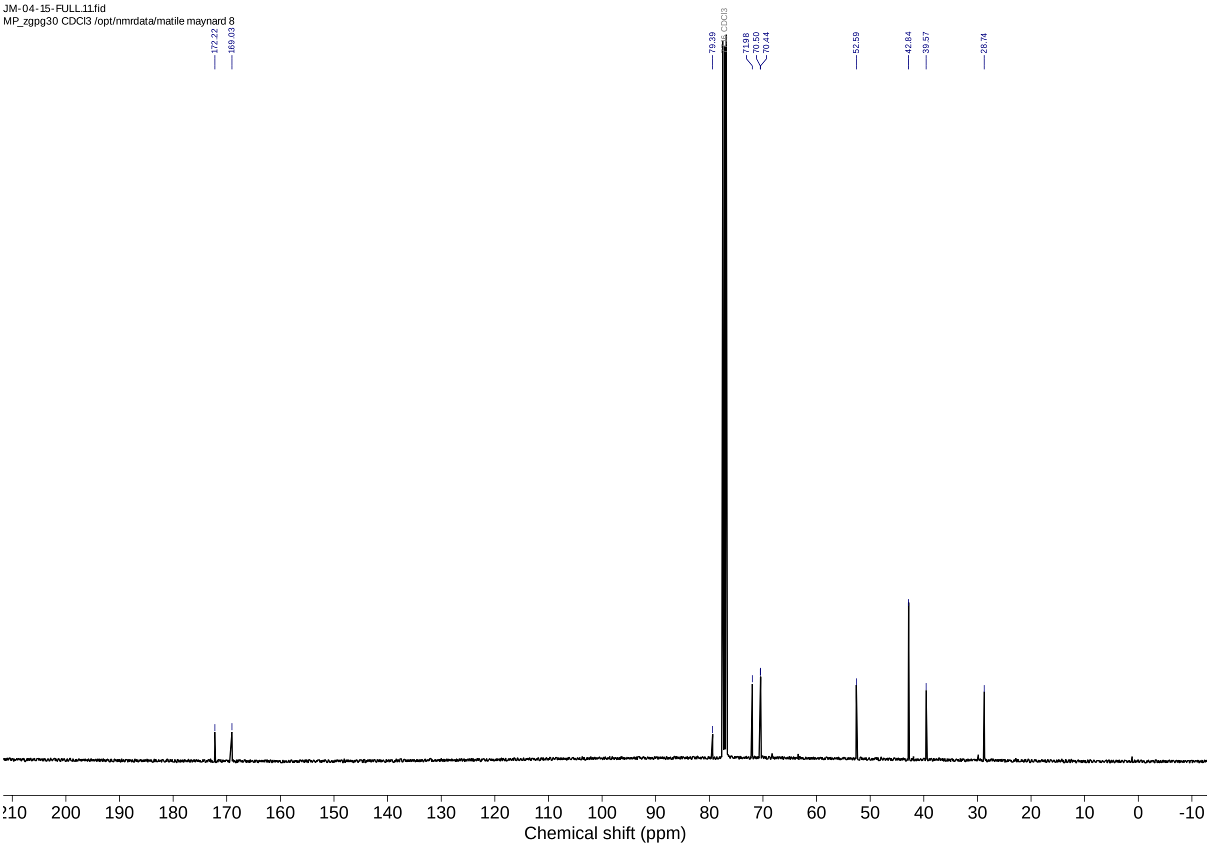

Supplement: SC-013-D2SC04558C-s001 [file SC-013-D2SC04558C-s001.pdf]
